# Molecular activity of bioactive phytocompounds for inhibiting host cell attachment and membrane fusion interacting with West Nile Virus envelope glycoprotein

**DOI:** 10.1371/journal.pone.0321902

**Published:** 2025-04-24

**Authors:** Noimul Hasan Siddiquee, Shanjida Akter Joyoti, Bushra Binte Zaker, Mansura Akter Eva, Alif Islam Nava, Nusrat Jahan Mridu, Al Amin Shawon, Sanjida Rahman, Tasnuva Jamil Chowdhury, Susmita Sarkar Katha, Md. Rafiul Islam, Mohammad Sharif Uddin

**Affiliations:** 1 Department of Microbiology, Noakhali Science and Technology University, Noakhali, Bangladesh; 2 Bioinformatics Laboratory (BioLab), Noakhali, Bangladesh; 3 Department of Fisheries, University of Chittagong, Chittagong, Bangladesh; 4 Department of Biotechnology, BRAC University, Dhaka, Bangladesh; 5 Department of Biochemistry and Molecular Biology, University of Dhaka, Dhaka, Bangladesh; 6 Department of Pharmacy, Bangladesh University, Dhaka, Bangladesh; 7 Department of Biochemistry and Molecular Biology, Noakhali Science and Technology University Noakhali, Noakhali, Bangladesh; 8 Department of Biotechnology & Genetic Engineering, Noakhali Science and Technology University, Noakhali, Bangladesh; 9 Department of Microbiology, University of Chittagong, Chittagong, Bangladesh; Debre Markos University, ETHIOPIA

## Abstract

West Nile virus is an arbovirus primarily spread by mosquitoes, which are the principal carriers and belong to the Flaviviridae category. This widespread disease lacks specific treatments despite its potential lethality, urgently demanding novel pharmaceutical research and development aims to prevent severe or long-term complications and improve overall outcomes. Pandemic awareness, increasing global incidence, fatal illness effects, expenses associated with outbreaks, reducing suffering, and other broader implications highlight the study’s wider significance. Drug design as a novel treatment approach to reduce the risk of resistance to the virus resulting from overuse of broad-spectrum antiviral therapies for unrelated viral diseases has been evaluated using computational techniques. Initially, molecular docking targeted the envelope glycoprotein of the WNV, utilizing a set of 5375 phytochemicals found in the IMPPAT database. Their binding affinities were −7.464, −5.802, −5.617, and −4.92, kcal/mol for CID: 359 (Phloroglucinol), 9064 (Cianidanol), 25310 (L-Rhamnose), and 492405 (Favipiravir), respectively. The lead compounds and the control ligand both bind at the common active site of the macro-molecule, as evidenced by their interactions with the same amino acid residues at LEU281, ASN47, THR282, SER29, MET48, MET46, and MET45, correspondingly. In post-docking MM-GBSA the negative binding energy of the P-L complex for the compounds CIDs: 359, 9064, 25310, and 492405 (control) were −29.16, −33.45, −32.02, and −3.16 kcal/mol, correspondingly. The selected compounds are secure and efficient since they demonstrate excellent toxicological and Pk characteristics. The compounds were further evaluated to confirm their stability and binding affinity to the target protein by molecular dynamics simulation (RMSD, RMSF, Rg, SASA, H-bond, P-L, and L-P contact). Following this, principal component analysis (PCA) and dynamic cross-correlation matrix (DCCM) studies were conducted using the MD trajectory data. The ligands evaluated in this study demonstrated considerable stability of the proteins’ binding site when complexed with CID: 9064 and CID: 25310, respectively, in the MD simulation, which also revealed a high negative binding free energy value, indicating a robust interaction between the target and lead compounds. The three principal components (PC1, PC2, PC3) for the lead compounds corresponding to CID: 9064 (40.37%, 23.02%, and 8.82%) and CID: 25310 (73.04%, 10.06%, and 3.77%), respectively, indicate that their complexes are more stable than the other L-P complexes. Consequently, both the compounds derived from the plants *Tamarindus indica* and *Plantago ovate*, respectively, may potentially impede the viral activity of the WNV envelope glycoprotein, indicating the possibility of these compounds as prospective phytochemical therapeutic candidates. This preclinical study can be used in further drug development processes, including *in vivo* studies and animal trials.

## 1. Introduction

One of roughly 75 viral species in the Flaviviridae family, West Nile virus (WNV) is a zoonotic flavivirus transmitted by mosquitoes [1]. WNV, Alfuy virus (ALFV), St. Louis encephalitis virus (SLEV), and Murray Valley encephalitis virus (MVEV) are members of the same serocomplex as the Japanese encephalitis virus [2,3]. It was originally identified from a febrile patient in 1937 in Uganda’s West Nile Province [4–6]. Since the virus only caused mild, subclinical infections, it was once thought to be of less importance to humans [7].

The WNV is an enclosed virion with a positive-sense, single-stranded RNA genome. The genome is made up of a single, roughly 11 kb open reading frame that lacks a polyadenylation tail at the 3′ end. The genome’s 5′ and 3′ noncoding regions combine to produce stem-loop structures, which facilitate transcription, translation, packaging, replication, and other processes [8–10]. The positive-strand RNA genome of flaviviruses is packaged into particles with an underlying lipid membrane and a stiff outer protein shell. The outer shell is made up of the tiny membrane protein M and the large envelope glycoprotein E. The α-helical hairpins at the C-terminus secure E and M within the lipid membrane. E is the main envelope component and is in charge of binding to receptors. αVβ3 integrin is a potential host cell receptor for WNV [11,12].

However, similar to dengue virus E, E may also attach itself initially by engaging glycosaminoglycans [13] or by binding a carbohydrate recognition protein via a glycan on the viral surface [11,14]. In fact, it has recently been discovered that the C-type lectin DC-SIGNR selectively binds the glycan on the WNV to facilitate cellular attachment. The virion is guided to the endocytic route via receptor binding. To transfer the viral genome into the cytoplasm for replication, flaviviruses need to fuse their lipid membrane with the membrane of the host cell once they have reached an endosome. The endosome’s lower pH causes a conformational shift in E, which bends the two apposed membranes toward one another and causes them to merge, providing the energy needed for membrane fusion, molecular diagram showing these viruses’ fusion process. The structurally preserved “class II” fusion proteins, which are also present in alphaviruses, include the flavivirus E proteins. We have a comprehensive molecular understanding of the fusion mechanism of three class II fusion proteins: dengue virus E, tick-borne encephalitis (TBE) virus E, and Semliki Forest virus E1. These crystal structures show us the viruses’ prior and subsequent fusogenic conformational rearrangements. The hydrophobic anchor known as the fusion loop is first inserted by E into the host cell membrane’s outer bilayer leaflet. Second, E folds back on itself, pointing the transmembrane anchor at its C-terminal in the direction of the fusion loop. The viral membrane, held by the C-terminal transmembrane anchor, and the host cell membrane, held by the fusion loop, are forced against one another by this fold-back, causing the two membranes to fuse. We now present the structure of a soluble portion (residues 1–406) of the prefusion conformation of the WNV’s E protein (sE). All but about 50 residues of the E ectodomain are present in the sE segment [13–16]. It takes more than ten years, a significant sum of money, and numerous resources to create a novel medication candidate [12,17].

Viruses are characterized by their RNA or DNA genomes and protein envelopes. Their reproduction and survival are reliant on the host’s metabolism and surroundings. They spread throughout the body by taking advantage of the host’s cellular machinery [12,17]. WNV is transmitted to vertebrate hosts by an infected mosquito vector during the probing stage of blood feeding. In order to inject pharmacologically active saliva proteins and find a blood supply, mosquitoes use their proboscis to probe the skin of their hosts. Blood feeding effectiveness is severely limited, even though many hematophagous insects may receive a blood meal without functioning salivary glands [18–20]. To combat the hemostatic system of their host, hematophagous mosquitoes inject a minimum of one coagulation blocker, one platelet blocker, and one vasodilator. Saliva frequently contains additional immunomodulatory, digestive, and antibacterial proteins [20–22]. Conventional therapy may demonstrate significant adverse effects or decreased efficacy; for instance, some individuals are experiencing serious side effects of conventional medications, including β-interferon and cortisone. Additionally, there is a scarcity of standard therapy alternatives for a certain medical condition [23].

One major obstacle in the development of antivirals is the ability of viruses to enter and evolve evasion techniques [20]. The plants’ diversely characterized metabolites and compounds may be assessed and employed to mitigate the evasion and drug-resistance challenges associated with antivirals, hence impeding the spread of the virus [21]. The antiviral action of phytochemicals is regulated by many mechanisms [24]. Any synthetic medication derived only from plant sources, including aerial and non-aerial components, juices, resins, and oil, whether in its raw or pharmaceutical form, is referred to as a phytochemical therapy or herbal medicine [25]. IMPPAT, a large online library on the phytochemistry of Indian medicinal plant species, would enable computational tools for plant-based drug development. The goal of the analysis was to determine how well the medication provided by the website combats WNV. This study set out to investigate the potential of phytochemicals to combat the WNV E-glycoprotein by using 5375 compounds from 32 distinct medicinal plants [21,24].

There is currently no specific treatment for WNV infections; however, activities such as compound inspecting and high-throughput database screening are being carried out under the 7th EU Framework grant agreement number 260644 in an effort to find suitable antagonists of the WNV virus [26]. During the pre-antibiotic period, horse serum administration for meningitis induced by *Neisseria meningitidis* remarkably diminished fatality rates. High titer anti-WNV immunoglobulin, ribavirin, and interferon (IFN)-α2b are presently considered possible cures for severe WNV infections [27]. Numerous vaccine candidates for WNV prevention in humans have been developed over the past 20 years. Several innovations (Hydrovax-001, ChimeriVax-WN02, and VRC WNV) have progressed to clinical trials; however, these techniques have not yet resulted in a licensed medication [28]. WNV Drug Discovery is challenging due to its complicated viral lifecycle, lack of licensed antiviral drugs, and funding limitations as a neglected tropical illness. The erratic nature of WNV illness prevalence has rendered it difficult to evaluate the efficiency of therapeutic immunoglobulins, which generates a major limitation to the testing and licensing of promising WNV vaccines and therapeutics [29]. Numerous phytochemicals have shown promise in recent studies against a variety of viral targets, lowering the potential of resistance. When compared to synthesized medications, naturally derived compounds are frequently less toxic, improving their safety profiles. Plant-based chemicals are affordable and easily available, particularly in environments with low resources.

In recent years, important advances have been made in the study of WNV through the application of computational tools like molecular docking, bioinformatics-based approaches, and molecular dynamics simulations [30]. Using molecular docking investigations, possible inhibitors of the WNV NS3 protease, a crucial enzyme for viral replication, have been screened and found, offering hope for therapeutic interventions. With a good ratio of binding affinity versus molecular weight (ligand efficiency of 0.33 kcal/mol per non-hydrogen atom), the inhibitor [4-(carbamimidoylsulfanylmethyl)-2,5-dimethylphenyl]-methylsulfanylmethanimidamide] has good potential as a lead compound for further development to combat WNV infections [31]. Researchers can gain a deeper understanding of WNV’s molecular mechanisms and develop more potent therapeutic approaches by utilizing computational methodologies. In this inquiry, a number of potential natural compounds that have the ability to suppress WNV *in silico* models have been investigated; however, none of these candidates have advanced to the point of clinical assessment [32].

Here, Favipiravir has been used as a control ligand. Fifty distinct influenza virus types are inhibited by Favipiravir, including the A (H1N1) pandemic virus, seasonal strains A (H1N1), A (H3N2), and influenza B; highly pathogenic avian influenza virus A (H5N1) isolated from humans; A (H1N1) and A (H1N2) isolated from pigs; and A (H2N2), A (H4N2), and A (H7N2) [33]. Apart from its ability to combat influenza and Ebola viruses, Favipiravir has demonstrated therapeutic efficacy against agents that cause viral hemorrhagic fevers and encephalitis in both mouse and cell culture models of arenavirus, bunyavirus, filovirus, WNV, yellow fever virus, foot-and-mouth disease virus, and Lassa virus [34].

**Fig 1:**
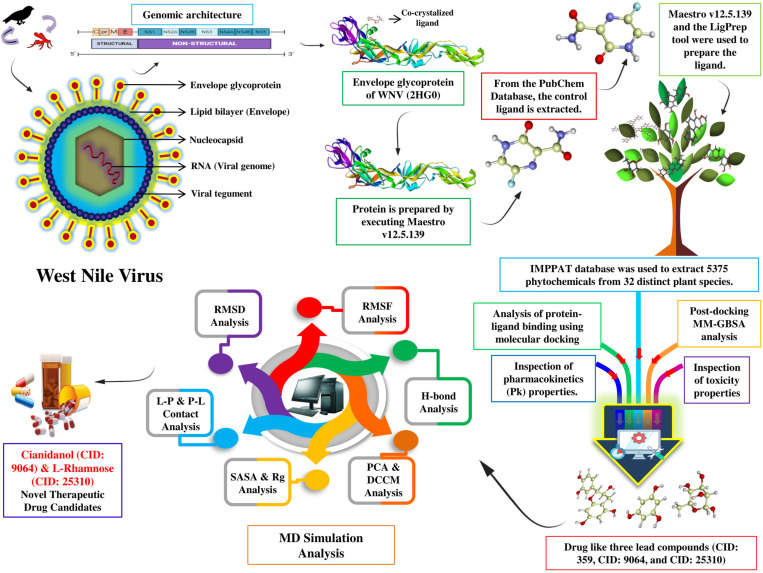
A graphical representation of the study conducted here to find potential phyto-compounds inhibiting West Nile Virus envelope glycoprotein.

This investigation employs a variety of bioinformatics programs, algorithms, and statistical methods to examine and define the binding affinity and interactions between WNV E-glycoprotein and molecules of the anti-viral medication Favipiravir along with other drug-like phytochemical compounds. Further evaluation of the antiviral drugs was carried out using Pk and toxicity tests to assess their potential. Molecular modeling and *in-silico* chemistry have become increasingly popular in Computer Aided Drug Design (CADD). Notably, the procedures required by traditional approaches to discover a novel medicine are costly and time-consuming. On the other hand, computer modeling reduces the need for animal testing and provides a speedier, more affordable, and more dependable substitute for current drug development methods [35]. By using CADD, it is possible to virtually screen large chemical libraries for interesting drug candidates, computationally identify potential pharmacological targets, undertake an *in-silico* investigation of their potential toxicity, and further optimize potential drug prospects [36]. To find the most effective medication, we also evaluated the consistency of the bound drugs to the E glycoprotein of the WNV during physiological settings using molecular docking, post-docking MM-GBSA, ADMET, MD simulation (RMSD, RMSF, hydrogen bond interaction, SASA, Rg, P-L contact, PCA, and DCCM) **Fig 1**.

Although computational approaches have their positive aspects, they do have drawbacks when it comes to drug discovery. Building comprehensive pre-computed databases of three-dimensional structures for protein or binding sites is a major limitation of inverse docking [37]. Problems with the accuracy of data and the simplifying of biological relationships are obstacles to Quantitative Structure-Activity Relationship (QSAR) modelling. There are limitations in simulating solvent effects in molecular docking and simulations, which need significant computer resources and accurate input structures. The computational cost and sensitivity to starting conditions of MD simulations are high. The diversity of data sources makes synthesis difficult, and errors in interpretation might result from relying too much on computational predictions without first validating them experimentally [38].

## 2. Materials and methods

### 2.1 Extraction and purification of the desired protein

**Fig 2:**
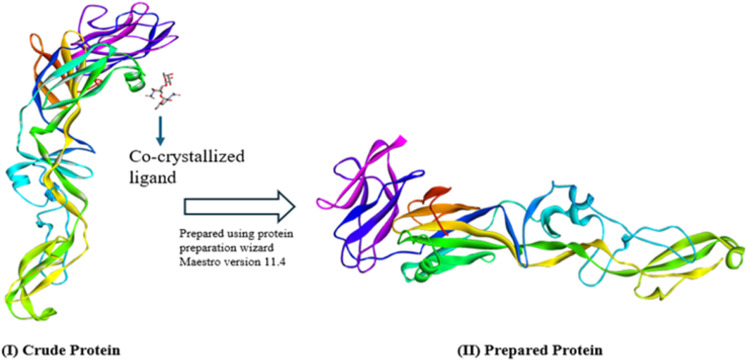
(Ⅰ) The WNV envelope glycoprotein’s (PDB ID: 2HG0) structure, comprising co-crystallized ligands, side chains, water molecules, and hetatm molecules, (Ⅱ) E-glycoprotein’s structure (Chain-A); applying the removal of water molecules, hetatm molecules, and co-crystallized ligands.

Using the RCSB PDB (https://www.rcsb.org/), the 3D configuration of the E-glycoprotein (2HG0) of the WNV was obtained. It is made up of a single chain (chain A), and the sequence length is 408 AA long [39]. In order to optimize subsequent analysis, we utilized the protein preparation tool Maestro v12.5.139 to prepare the protein structure, this involved eliminating the side chains, hetatm molecules, water molecules, and co-crystallized ligand [40]. Consequently, the default setting for protein preparation has been used to add hydrogen atoms and side chain residues that are absent, as well as to assign the protein’s bond ordering. The OPLS-3e force field was utilized in the protein preparation process (Fig 2) [41,42].

### 2.2 Extraction and processing of the ligand

Favipiravir, an antiviral medication, has been taken as a control ligand for the target protein. From PubChem (https://pubchem.ncbi.nlm.nih.gov), the SDF file of the 3D conformer of Favipiravir (CID: 492405) was extracted. This study investigated 5375 phytochemicals from 32 plant species with antiviral properties that are available in the Indian subcontinent to find potential inhibitory compounds. The phytochemical compounds were obtained from the IMPPAT (https://cb.imsc.res.in/imppat) database in SDF format [43]. To prepare ligands, Maestro v12.5.139’s LigPrep module was applied and with the help of the OPLS-3e force field, protein-molecule architectures were optimized [42].

### 2.3 Analysis of molecular docking

Molecular docking is an impressive screening technique and it aims to establish an ideal binding affinity and attachment configuration between the ligand and the target [44]. This approach can yield significant concepts for the advancement of new pharmaceuticals, the associations between L-P, and other physiological activities [45]. Via guidance of Schrödinger-Desmond tools through Maestro v12.5.139 and Glide v8.8, 5375 phytochemicals, together with the control, were docked with the target protein. OPLS-3e in standard precision mode was used as the force field throughout the docking process [46]. In this research, the target protein’s entire surface was incorporated in the receptor grid for blind docking, and the box range found X = -8.603 **Å**, Y = 108.039 **Å**, Z = 49.728 **Å**. In this work, the binding affinity between the ligand and target macromolecule was calculated, and the Maestro viewer showed the many kinds of chemical bonds and residues that are capable of binding to ligands.

### 2.4 Inspection of pharmacokinetics (Pk) properties

Pharmacokinetics is derived from two words: *Pharmaco*, which means ‘drug’, and *kinesis*, which means ‘movement’. The fate of foreign compounds, such as drugs, can be interpreted, understood, and even predicted by studying their dynamic movement along the body [47,48]. Hence, the quantitative research that involves the rate of drug ADME, uses mathematical equations in order to understand the basic principles and even estimate the nature and the range of toxic or therapeutic effects (biological effects) [11,48]. Furthermore, this plays a preliminary and crucial part in drug design, affecting the drug’s safety and efficacy. In our study, we checked the Pk properties of our intended compounds by deploying the server of the Swiss Institute called SwissADME (http://www.swissadme.ch/) in order to assess the behavior of each compound of the plants [49].

### 2.5 Inspection of toxicity properties

The optimization of drug-like properties of the lead compounds and improving their safety, pharmacokinetics, and efficacy by assessing their toxicity properties during the drug development process dictates advancement, success, and therapeutic effectiveness [50]. This is accustomed to utilizing advanced computational tools and predictive models using a platform called ProTox-3.0 (https://tox.charite.de/protox3/), prediction of toxicity of chemicals, and a proven server with enhanced and widely used methodology [51].

### 2.6 Post-docking MM-GBSA Analysis

MM-GBSA is a computational approach used to evaluate the unbound energy of binding ligands and proteins. When the ligand is free from the protein complex, the difference in energy levels between the two substances is known as the free energy of binding [11]. Glide v-8.8 and Maestro v-12.5.139 were used to analyze and visualize the chosen compounds in order to display the lowest energy binding [52]. This process was used as a post-docking verification tool and also used in many types of protein-ligand interactions like protein-protein interactions, drug-receptor binding, and enzyme-substrate binding. After determining the residues using the natural ligand active pocket, a grid box size X = −8.603 **Å**, Y = 108.039 **Å**, Z = 49.728 **Å** was created to represent the precise place where the protein interacts. Using the docking value and the MM-GBSA score as control variables, the result was compared to recently screened drugs [53].

### 2.7 MD Simulation

MD simulation is a computational tool [54] to facilitate the understanding of chemical and physical phenomena of dynamic systems such as a receptor or P-L complex [32]. The phenomena may include protein characterization, new binding site analysis, free energy calculations, and so forth. Generally, we aimed for the three best-docked compounds whose PubChem Compound identification includes CID: 359, 9064, 25310, and 492405 (control) for MD simulation lasting 100 ns targeting the West Nile virus using the PDB Identifier 2HG0. This was executed using the Desmond package available within the Schrödinger suite. To maintain constant system volumes, an orthorhombic periodic simulation boundary box was constructed upon every complex of the simple point charge (SPC) water model, unraveling its complex molecular behavior with a dimension of (10×10×10 **Å**^3^). To sustain neutrality within the system of 0.15M concentration, Na^+^, and Cl^-^ ions were promiscuously introduced and spread across a solvation process. Performing MD simulation facilitated the observation of the system energy, which was optimized using the OPLS-3e force field, the steepest viable method to assess protein-ligand binding trajectory output for 100 ns. An equilibration was then executed on every complete system to ensure the system’s robustness by NPT ensemble performance at a controlled pressure and temperature of 1.01325 bar and 300K followed by energy 1.2 recorded intervals of 100 ps. The trajectory allows researchers to imitate the dynamics of molecular systems and observe how they are involved over time. The course in protein structure dynamics aided components in this system stability investigation include- RMSF, followed by RMSD, hydrogen bond interaction, SASA, Rg, and lastly, P-L contact using simulation interaction diagram (SID) tool provided within the Schrödinger software package. Principal Component Analysis (PCA) and dynamic cross-correlation matrix (DCCM) from the MD trajectory were conducted utilizing the Bio3D package in R programming [32]. Computational approaches can speed up the process of discovering new hit compounds by predicting the binding affinity of small molecules to certain biological targets [55]. Molecular dynamics studies were carried out to assess the stability of the receptor-ligand complexes by analyzing RMSD. RMSF analysis of each system was conducted to assess the displacement and stability of each residue throughout the simulation time. A protein complex’s stability is directly related to the amount of hydrogen bonds between its molecules [56]. The Rg reflects the compactness of a structure and a system with less fluctuation over the simulation is more robust and compact [57].

## 3. Result

### 3.1 Analysis of molecular docking and protein-ligand interaction

Molecular docking was performed using Favipiravir and many drug-like compounds. Out of these compounds, three had more exceptional docking scores than Favipiravir (control). We have selected three compounds including CID: 359 (IMPHY003265), 9064 (IMPHY014854), and 25310 (IMPHY015056) for further analysis as they have a greater binding affinity with docking scores −7.464, −5.802, and −5.617 kcal/mol, respectively than the control CID: 492405 (−4.932 kcal/mol) (**Table 1**).

**Table 1:** Compounds identification according to PubChem CID, name, and docking value of the top three compounds, as well as Favipiravir (control), with therapeutic uses.

PubChem CID	Compound Name	Docking Value (Kcal/ mol)	Source (Plant)	Therapeutic Use
CID: 359	Phloroglucinol	−7.464	*Phyllanthus emblica* [58], [59]	Antibacterial, Antifungal, Antiviral [60] Antioxidant [61] Anti-inflammatory, Anticancer [62]
CID: 9064	Cianidanol/ (+)-catechin	−5.802	*Tamarindus indica* [63]	Breast cancer [64] Parkinson’s disease [65]
CID: 25310	L-Rhamnose	−5.617	*Plantago ovate* [66]	Antitumour, Anticancer [67]
CID: 492405 (control)	Favipiravir	−4.932	N/A	Anti-influenza [68] Antiviral [69], [70]

**Fig 3:**
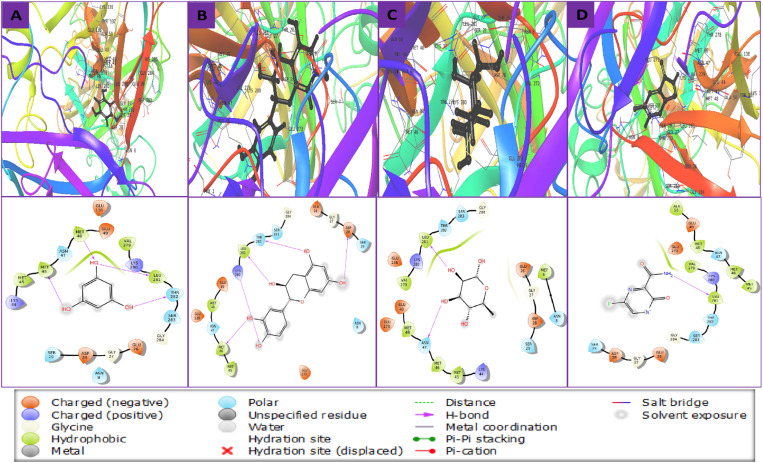
The relationship between the WNV’s E glycoprotein (2HG0) and selected three compounds in 3D and 2D formats, with compounds (A) CID: 359, (B) CID: 9064, (C) CID: 25310, and (D) CID: 492405 in the protein’s active pocket.

In order to do further analysis, the Maestro module of the Schrödinger suite was utilized to show the molecular interactions of the top docking result with the selected three compounds that were collected from plants. The top three selected compounds and the control ligand cooperated with the protein’s shared AA residues during molecular docking. The amino acids MET45, MET46, MET48, and LEU281 are commonly found in hydrophobic bonds. Each of the three selected compounds worked in parallel with a different AA, which is documented in **Table 2** and illustrated and explained in **Fig 3**.

**Table 2:** Highlighting the amino acid residues associated with H-bonds, polar bonds, and hydrophobic bonds that form between the target protein and selected three compounds, as well as the control.

PubChem CID	Hydrogen Bonds	Polar Bonds	Hydrophobic Bonds
CID: 359	MET46, MET48, LEU281, THR282	ASN47, THR282, SER283, ASN8, SER29	MET45, MET46, MET48, VAL279, LEU281
CID: 9064	LYS280, LEU281, THR282, SER283, ASP28	THR282, SER283, SER29, ASN8, ASN47	MET45, MET46, MET48, LEU281
CID: 25310	LEU281, ASN47	THR282, SER283, ASN47, SER29, ASN8	MET48, MET46, MET45, MET6, VAL279, LEU281
CID: 492405 (control)	LEU281	ASN47, THR282, SER283, SER29	MET48, MET46, MET45, VAL279, ALA50, LEU281

### 3.2 Post-docking MM-GBSA analysis

**Fig 4:**
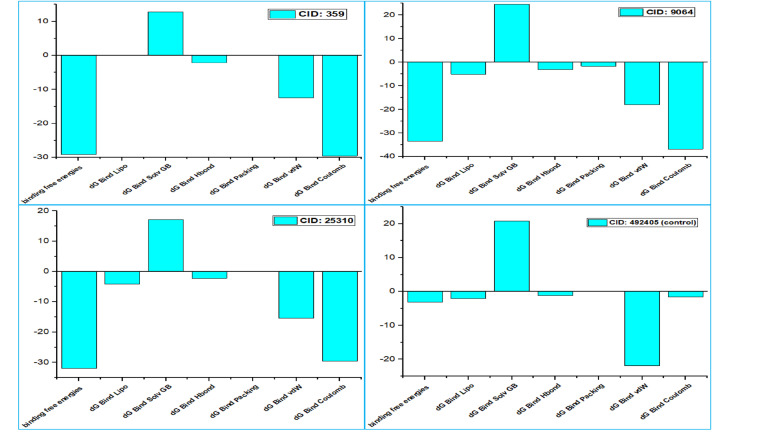
The bar diagram shows MM-GBSA of the compounds CID: 359, 9064, 25310, and the control 492405 demonstrating negative binding free energies.

We have utilized the MM-GBSA methodology to calculate the free energy of binding at the P-L complex endpoint. The three chosen compounds (CIDs: 359, 9064, and 25310) and the control (CID: 492405) interacted with the target protein and displayed increased negative binding free energy scores and a net negative pattern, according to an analysis of MM-GBSA. Upon nearing the end of the molecular docking procedure, a complicated examination of MM−GBSA found the compounds CID: 359, 9064, 25310, and the control 492405 demonstrated negative binding free energies of −29.16, −33.45, −32.02, and −3.16 kcal/mol, corresponding to that order. The selected compounds (CIDs: 359, 9064, and 25310) exhibited a more consistent interaction with the protein in comparison with the control (CID: 492405), and among these compounds, CID: 9064 and 25310 showed the highest negative binding free energy (−33.45 and −32.02 kcal/mol). Additional analysis of these three compounds showed noteworthy activity of ΔBind Coulomb, ΔG Bind Lipo, ΔG Bind Solv GB, ΔG Bind Hbond, ΔG Bind Packing, and ΔBindvdW. Based on the molecular docking result, the comprehensive examination of MM−GBSA also calculated −29.5, −36.79, −29.62, and −1.58 kcal/mol of ΔBind Coulomb; 0, −5.06, −4.23, and −2.05 kcal/mol of ΔG Bind Lipo; 12.8, 24.63, 17.14, and 20.81 kcal/mol of ΔG Bind Solv GB; −2.16, −3.12, −2.31, and −1.19 kcal/mol of ΔG Bind Hbond; 0, −1.66, 0, and 0 kcal/mol of ΔG Bind Packing; −12.46, −18, −15.43, and −21.85 kcal/mol of ΔBindvdW for the molecules CID: 359, 9064, 25310, and the control 492405, respectively (**Fig 4**). According to the information above, the three compounds selected have the ability to attach to the E−glycoprotein binding pocket (PDB ID: 2HG0) for a long period of time, hence hindering the target macromolecule.

### 3.3 Assessment and evaluation of pharmacokinetics (Pk) properties

The drug’s pharmacokinetic profile can be enhanced using ADME qualities, including processes that are integral to understanding the overall pharmacokinetics of a drug [51]. We investigated the ADME qualities of three phytochemicals among 5375 docked compounds found in the IMPPAT database against Favipiravir based on their high docking score and negative binding free energy. Consequently, the SwissADME server has been employed in the study to analyze the Pk characteristics of the three selected drug-like molecules, including CID: 359, 9064, 25310, and control (CID: 492405) which are listed in **Table 3**. The analysis indicated that the above-selected compounds had efficient and druggable Pk characteristics.

**Table 3:** List of Pk characteristics of three lead compounds with the control (Favipiravir).

Properties		CID: 359	CID: 9064	CID: 25310	CID: 492405 (control)
**Physicochemical Properties**	MW (g/mol)	126.11	290.27	164.16	157.1
	Heavy atoms	9	21	11	11
	Arom. Heavy atoms	6	12	0	6
	Rotatable bonds	0	1	0	1
	H bond acceptor	3	6	5	4
	H bond donor	3	5	4	2
**Lipophilicity**	consensus (log P_o/w_)	0.45	0.83	−1.36	−0.27
**Water Solubility**	Log S (ESOL)	−1.22	−2.22	0.46	−0.8
**Pharmacokinetics**	GI absorption	High	High	High	High
	BBB permeant	Yes	no	no	No
**Drug likeness**	Lipinski violation	Yes; 0 violation	Yes; 0 violation	Yes; 0 violation	Yes; 0 violation
**Medicinal Chemistry**	Synthetic accessibility	1	3.5	4.05	2.08

### 3.4 Assessment and evaluation of toxicity properties

Toxicology assesses the level of a particular substance’s toxicity to individuals and potential organ damage. Identifying a molecule’s toxicity is an essential phase of *in-silico* drug development, and the ProTox-3.0 server can help achieve this. The properties studied are classified into toxicity endpoints and organ toxicity of the three selected drug-like compounds and the control (Favipiravir). The three lead compounds (CID: 359, 9064, and 25310) have “**Inactive**” outcomes in the majority of organ toxicity and toxicity endpoints, exhibiting “**Active**” status just in Nephrotoxicity and Cardiotoxicity results (**Table 4**). Nephrotoxicity can cause renal dysfunction or failure, whereas cardiotoxicity harms cardiac muscle due to harmful substances. Mitigating nephrotoxicity and cardiotoxicity in the future could render the compounds effective medicinal agents. Overall, the three lead compounds (CID: 359, 9064, and 25310), and control (CID: 492405) exhibit impressive safety profiles.

**Table 4:** List of toxicity characteristics of three lead compounds with the control (Favipiravir).

Classification	Target		CID: 359	CID: 9064	CID: 25310	CID: 492405 (control)
**Organ toxicity**	Hepatotoxicity	Prediction	Inactive	Inactive	Inactive	Inactive
		Probability	0.78	0.72	0.85	0.66
	Neurotoxicity	Prediction	Inactive	Inactive	Inactive	Active
		Probability	0.75	0.90	0.93	0.80
	Nephrotoxicity	Prediction	Active	Active	Active	Inactive
		Probability	0.61	0.62	0.66	0.53
	Cardiotoxicity	Prediction	Active	Inactive	Active	Inactive
		Probability	0.85	0.99	0.62	0.80
**Toxicity end points**	Carcinogenicity	Prediction	Inactive	Inactive	Inactive	Active
		Probability	0.73	0.51	0.61	0.53
	Immunotoxicity	Prediction	Inactive	Inactive	Inactive	Inactive
		Probability	0.99	0.96	0.97	0.99
	Mutagenicity	Prediction	Inactive	Inactive	Inactive	Inactive
		Probability	0.97	0.55	0.73	0.76
	Cytotoxicity	Prediction	Inactive	Inactive	Inactive	Inactive
		Probability	0.95	0.84	0.78	0.84

### 3.5 MD Simulation analysis

A 100 ns MD simulation was performed in this research to gain a better understanding of the protein’s conformational shifts in response to a particular ligand. Before examining intermolecular interactions, the final snapshots were extracted using the 100 ns MD trajectories, which were analyzed. The MD simulation trajectory was assessed based on the RMSD, RMSF, SASA, Rg, H-bond, PCA, DCCM, P-L, and L-P interactions.

#### 3.5.1 RMSD analysis.

**Fig 5:**
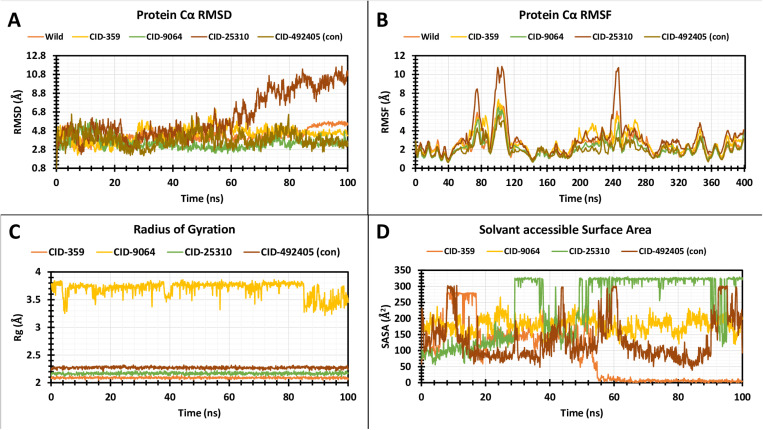
The apoprotein’s RMSD, RMSF, Rg, and SASA values are displayed complexed with the three lead compounds and control chosen and extracted from 100 ns MD trajectory complex system’s C α atoms.

The RMSD in MD simulations is the average distance generated by the displacement of a chosen atom over a certain time interval relative to a reference time. In the initial stage, RMSD of the structural atoms of the protein, such as Cα, back-bone, side chain, and heavy atoms, is determined. Subsequently, the RMSD of the protein fit ligand atoms from all the time frames is aligned and calculated and then compared to the reference time [71]. Consequently, a 100 ns MD simulation was conducted to examine the conformation shift of the target macromolecule in the complex of the specified compounds comprising CID: 492405 (control), 359, 9064, and 25310, shown in **Fig 5A**. The average RMSD scores for CID: 359, 9064, 25310, and 492405 (control) were 4.42 **Å**, 3.48 **Å**, 6.28 **Å**, and 3.87 **Å**. The greatest RMSD scores of the selected three compounds, CID: 359, 9064, 25310, and control (CID: 492405), were 7.047 **Å**, 6.027 **Å**, 11.679 **Å**, and 6.584 **Å**, correspondingly and the smallest were 2.192 **Å**, 1.594 **Å**, 1.763 **Å**, and 2.145 **Å**. The compound CID: 492405 (control) exhibited relatively excessive fluctuation during the 60–100 ns simulation time range, whereas it maintained the lowest continuous fluctuation from 0–60 ns with the apoprotein. Compared to the other two molecules (CID: 359, and 25310) and control (CID: 492405); CID: 9064 produced excellent outcomes when complexed with apoprotein until the last step of the simulation period and typically maintained consistent fluctuation.

#### 3.5.2 RMSF analysis.

The dynamic interactions of the P-L complex are quantified using RMSF analysis, depicting the variability of each atomic localization from their original coordinates in a protein. In essence, this metric underscores the constant stability of the ligand concerning the reference protein computed to visualize the amino acid residue position, which is generally denoted utilizing the α carbon’s spatial coordinates [72,73]. Unlike RMSD, which quantifies the deviation between the atomic positions of a reference structure and those of a target structure, RMSF measures the average fluctuations of atomic coordinates between an ensemble of structures or a single structure [74]. Therefore, the average RMSF values range closer around 2–3 **Å** [74] reflecting the regions of lower or higher flexibility across a protein structure for the selected molecules CID: 359, 9064, 25310, and 492405 (control) were 2.76 **Å**, 2.14 **Å**, 3.12 **Å**, and 2.05 **Å**, correspondingly. Upon keen observation of the result, the ligand structure CID: 25310 has the highest RMSF average value of 3.12 **Å**, also higher than 2.05 **Å** of the control CID: 492405 molecules, demonstrating an immense amount of instability and flexibility within the protein. A peak region of the protein was seen in all selected compounds at residual positions that showed the maximum fluctuation during the simulation period: THR76, TRP101, GLY106, ALA200, TRP217, THR248, LYS280, ASP348, and ASP381 (**Fig 5B**). The stiffest secondary structural elements had residues ranging from a minimum of 0–70 AA residues, 120–185 AA residues, and 280–340 AA residues, according to the RMSF (Fig 5B). The rigidity of the protein structure is demonstrated by the very small variance of the residues in comparison to the native functional components in the complex architecture. The presence of the β-sheet, α-helix, C- and N- terminal domains causes maximum fluctuation to be seen at the beginning and end of the protein. As a result, for CID: 9064 under study, the probability of an atom shifting under real-life conditions has been shown to be minimal.

#### 3.5.3 The radius of gyration (Rg) analysis.

The Rg functions as an insightful tool in interpreting the folding and compactness properties of proteins and P-L complexes [75]. By describing the RMSD of the atoms from the common point of gravity of a particular protein molecule, the average Rg statistically predicts the biomolecule’s dimensions, reflecting the system’s molecular compactness in its form. The following equation provides specific details with respect to the estimation of Rg:


$$r^{2}gyr=\frac{\sum_{i-1}^{n}W_{i}{\left(r_{i}–\bar{r}\right)}^{2}}{\sum_{i-1}^{n}W_{i}}$$

The variables in the equations comprise the position of the *i*^th^ atom (*r*_*i*_), the mass/weight of each atom (*W*), and the center mass of atom *i*(*r*). The mean value is obtained by averaging the Rg values over the frames in each trajectory [76].

A larger Rg score implies the protein molecules are weakly packed, while a smaller Rg value implies that the protein structures are firmly packed [77]. Therefore, a 100 ns simulation time was used for assessing the firmness of CID: 359, 9064, 25310, and 492405 (control) complexed with target protein with regards to Rg, shown in **Fig 5C**. The average Rg for the molecules CID: 359, 25310, and 492405 (control) was calculated to be 2.089 **Å**, 2.168 **Å**, and 2.272 **Å**, respectively, and they exhibited the lowest continuous fluctuation during a 100 ns MD simulation. Consequently, all the compounds that indicate the active site of the protein have no effect on significant conformational alterations after binding, except for CID: 9064, which exhibits the highest fluctuations from 4–8 ns and 84–100 ns over the simulation period, and the average Rg value for this compound was determined to be 3.693 **Å**.

#### 3.5.4 Solvent accessible surface area (SASA) analysis.

The term SASA refers to the surface area where the protein or ligand comes into contact with solvent molecules. It exhibits a correlation with the solvent-complex interactions that transpire during the simulation analysis [78,79]. When a ligand binds to a receptor, it changes the arrangement of the protein, and the SASA value is used to figure out how massive and significant these changes are [80]. A higher SASA value signifies the enlargement of the protein volume, and a low fluctuation is anticipated throughout the simulation period [57]. Therefore, the average SASA scores of the protein in association with the compounds CID: 359, 9064, 25310, and 492405 (control) were calculated, which respectively were 89.24 **Å**^2^, 179.86 **Å**^2^, 234.25 **Å**^2^, and 131.75 **Å**^2^, displayed in **Fig 5D**. The average SASA value for the complex mechanism was determined to range from 80 to 240 **Å**^2^, which implies that an AA residue in the complex mechanism was highly accessible to the specified molecule.

#### 3.5.5 Protein-ligand contact analysis.

**Fig 6:**
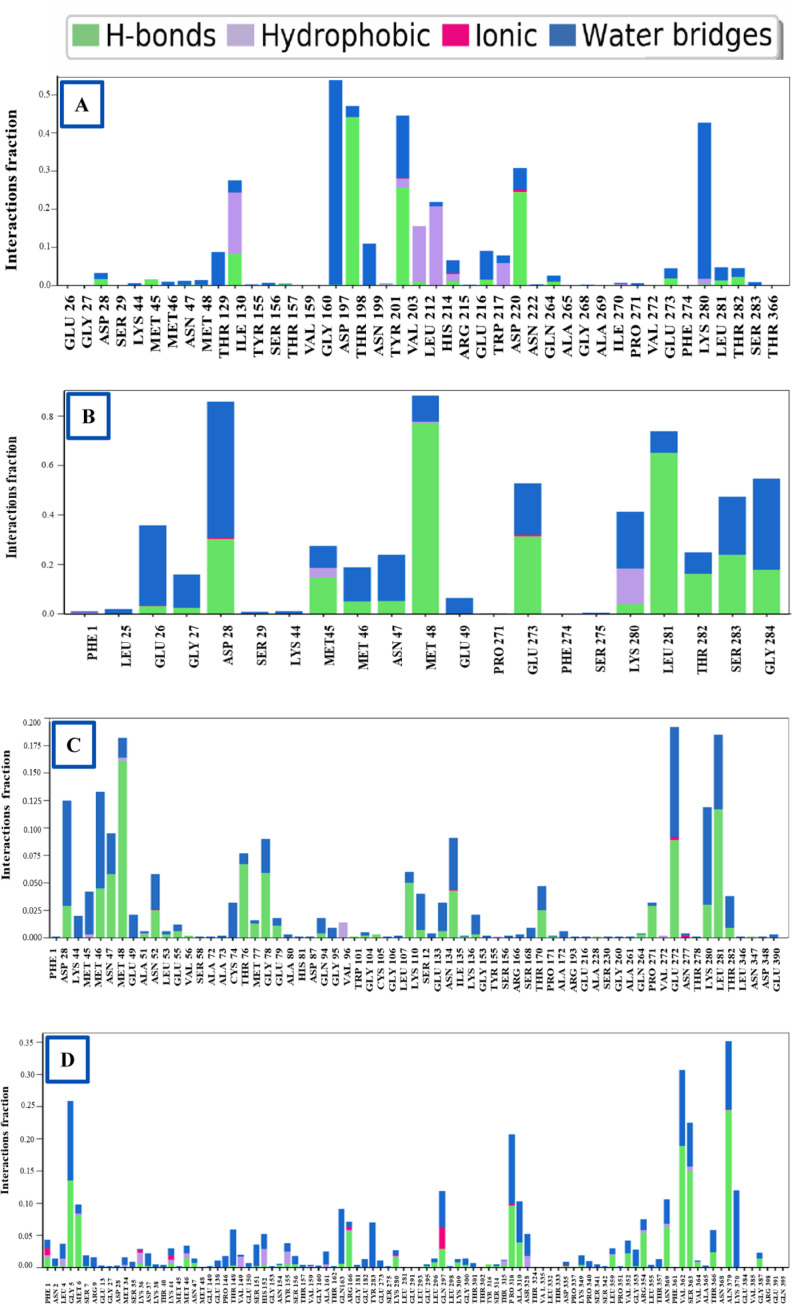
The bar charts display the P-L interactions determined during the 100 ns simulation period. In this figure, the 2HG0 protein of the WNV shows interaction with CID: 359 **(A)**, 9064 **(B)**, 25310 **(C)**, and 492405 (control) **(D)**.

During a 100 ns simulation period, the complex structure of a protein, along with its specified ligands and their intermolecular relationships, were analyzed utilizing the simulation interactions diagram (SID). Several parameters, including H-bonds, ionic bonds, hydrophobic bonds, and water bridge bonds, have been used to assess and present the protein-selected compounds (CIDs 359, 9064, 25310, and 492405, the control) interaction. The results are displayed in **Fig 6**. Throughout the 100 ns simulation, every molecule displayed a different interaction, which finally resulted in the formation of a stable binding with the target macromolecule. The molecules CID: 359 generated several interactions at the residues ILEU130, TYR20, HIS214, GLU216, ASP220, and LYS280 with interaction fractions (IF) of 0.28, 0.45, 0.07, 0.09, 0.32, and 0.45, respectively. The persistent specific association occurs during the simulation because the same subtype repeatedly makes contact with the ligand, as seen in **Fig 6A**. Multiple contacts between the compound CID: 9064 and the residues PHE1 (0.02), GLU26 (0.36), ASP28 (0.88), MET45 (0.3), MET48 (0.9), GLU273 (0.57), LYS280 (0.42) were generated, with the interactions occurring in accordance with the simulation duration (**Fig 6B**). MET48 (0.185), ALA51 (0.0052), ASN52 (0.061), ILE135 (0.085), ASN277 (0.19) residues were the sites of several contacts that the molecule CID: 25310 established, all of which were sustained in accordance with simulation time **(Fig 6C)**. According to simulation time, the molecule CID: 492405 (control) formed numerous contacts at residues of PHE1 (0.04), LEU4 (0.03), MET6 (0.09), LYS36 (0.025), LYS44 (0.025), MET46 (0.03), HIS152 (0.05), TYR155 (0.035), ARG166 (0.075), LYS280 (0.03), GLN297 (0.13), THR315 (0.02), PRO316 (0.022), LEU359 (0.04), ARG354 (0.07), ASN369 (0.12), SER363 (0.23), and ALN379 (0.37) (Fig 6D).

#### 3.5.6 Ligand-protein contact analysis.

Ligand-protein contact or interaction involves the study of how small molecules such as ligands bind in the course of developing effective drugs. This interaction can be looked into in the simulation interactions diagram (SID), which helps to understand and visualize several interactions between the complex of four selected ligands among which, one is a control ligand, altogether with apoprotein (2HG0) throughout the trajectory and extract interaction data, providing insights into key contact points and the binding stability.

**Fig 7:**
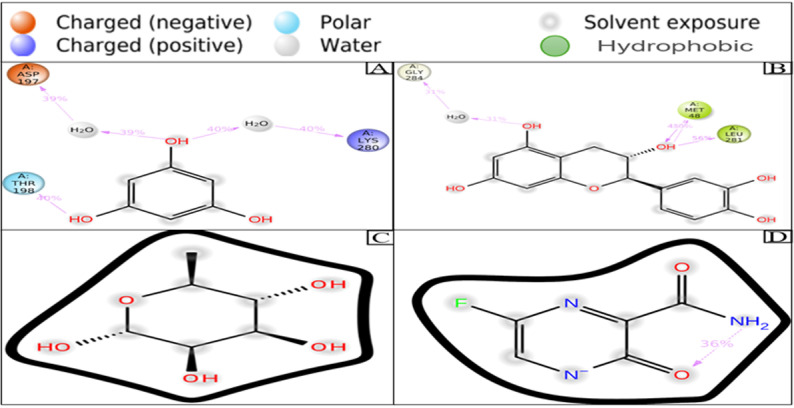
The interaction profiles between the ligand and the apoprotein are presented using the ligand-apoprotein detailed schematic diagram where CID: 359 (A), 9064 (B), 25310 (C), and 492405 (control) (D) illustrate the conformational isomerism of the ligands binding to wild type protein.

The three specific ligands, CID: 359, 9064, 25310, and 492405 (control ligand), are shown in **Fig 7**. During the simulation time, these compounds established a substantial amount of interactions of the same subtype among the ligands (CID: 359 and 9064) and the apoprotein, each showing more than two interactions (**Fig 7**). CID: 359 ligand structure shows three amino acid residues, which are Aspartic acid 197, Lysine 280, and Threonine 198, and two water molecules are bonded to it, respectively. On the other hand, ligand CID: 9064 is bonded to Glycine 284, Methionine 48, Leucine 28 amino acid residues, and water molecule, and based on the findings, ligand CID: 359 and CID: 9064 showed more stability than the control CID: 492405, respectively.

#### 3.5.7 Hydrogen bond analysis.

**Fig 8:**
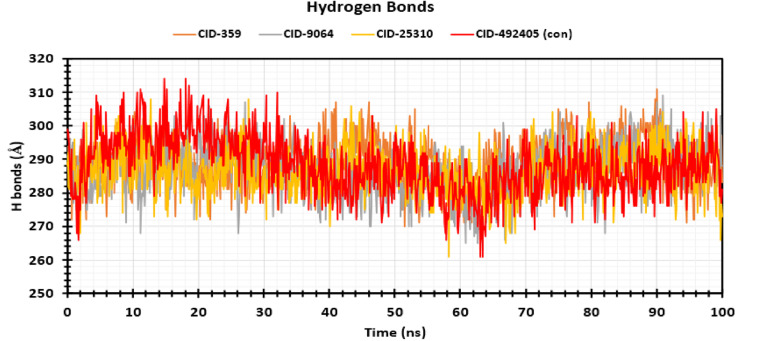
Reflecting the number of H-bonds established by the four selected compounds with the target macromolecule during the 100 ns MD simulation period. The ordinate of the Y-axis represents the number of hydrogen bonds in the P-L complex, while the ordinate of the X-axis represents time in ns. The colors orange, grey, yellow, and red symbolize CID: 359, 9064, 25310, and 492405 (control), correspondingly.

Hydrogen bond (HB), a type of non-covalent interaction, performs a vital role in biomolecule behavior and 3D structure [81]. The mechanism of biological recognition relies on hydrogen bonding, and the rapid formation and breakdown of hydrogen bonds are strongly connected to the molecular movement required for biological processes [82]. In addition to influencing drug specificity, metabolism acceleration, and adsorption, hydrogen bonds serve as essential when stabilizing the ligand with the target protein [71], [83,84]. Consequently, the number of H-bonds created during the interaction between the P-L complex was noticed over the 100 ns simulation period, illustrated in **Fig 8**. The average H-bond values for the compounds CID: 359, 9064, 25310, and 492405 (control) were calculated to be 289.07, 286.77, 286.57, and 288.33, correspondingly. Each of the compounds created numerous hydrogen bonds ranging from 260 to 315 simultaneously during the simulation period. As a result, every compound will significantly boost and stabilize the ligand-receptor association.

#### 3.5.8 Principal component analysis (PCA).

**Fig 9:**
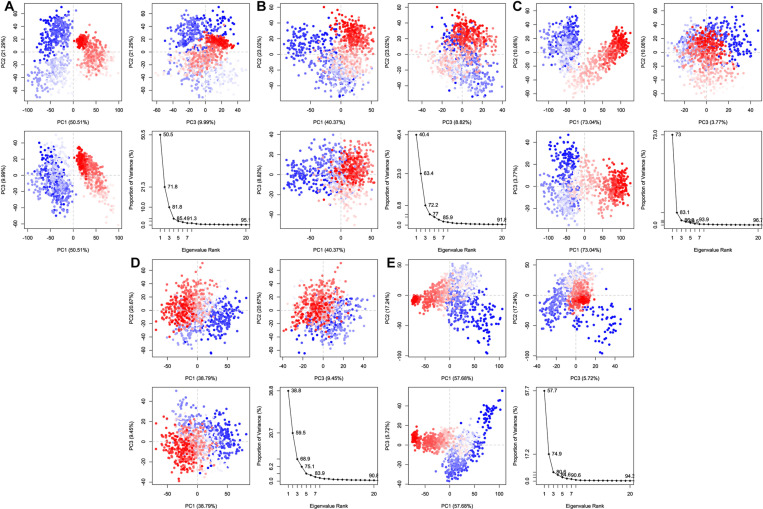
Eigenvalue versus proportion of variance in Principal Component Analysis, depicted across three unique panels representing different areas. Here, **(A)** CID: 25310, **(B)** CID: 9064, **(C)** CID: 359, **(D)** CID: 492405 (control), and **(E)** Apoprotein (2HG0) are referenced.

Principal component analysis, (PCA) in MD simulations is a powerful statistical technique used to simplify the dimensionality of complex data sets, identifying the dominant collective motions in a ligand-protein complex [11]. The collective trajectory data output during the MD simulations is projected onto sets of PCs (principal components) known as PC1, PC2, and PC3 to chart all the changes in the system. During the first 20 modes of motion, a graph of protein eigenvalues versus eigenvector indexes is plotted to show significant stability shown in **Fig 9**. Here, the eigenvalues and eigenvectors are obtained by decomposing the covariance metrics completed on the basis of atomic fluctuations around the main position of the system. This includes eigenvectors, which represent the directions of principal components in multi-dimension whereas eigenvalues represent the magnitude variance of the principal components in a PCA. Based on the simulation analysis, eigenvectors with higher eigenvalues appear to be accountable for the target protein-ligand complex’s overall motion whereas the top 5 eigenvectors in used systems showed dominant motion and had higher eigenvalues (73%-93.9%). The figure also illustrated cluster 2HG0-CID: 25310, where the PC1 cluster displayed the highest variability (73.04%), the PC2 cluster exhibited variability (10.06%) and the PC3 cluster showed the lowest variability (3.77%). Because PC3’s structure was the least variable and hence the smallest, it was believed to be the most stable ligand-protein binding system.

In the case 2HG0-CID: 9064, complex (**Fig 9B**), there is greater variability in PC1, PC2, and PC3 than in the 2HG0-CID: 492405 (control) complex (**Fig 9D**). In comparison to the other 3 complexes, here, the 2HG0-CID: 359 complex (**Fig 9C**) exhibits higher variability. However, 2HG0 (**Fig 9E**) has PC1, PC2, and PC3 values of 57.68%, 17.24%, 5.72%. Due to its simpler and more compact structure than PC1 and PC2, PC3 was believed to be the most stable protein. As we proceed down the principal components, variance tends to decrease, indicating more limited and localized motions where PC1 generally has the most variation. Simple clustering in the PC subspace revealed conformational variations where the color blue indicates the greatest degree of mobility, whereas the colors white and red indicate moderate and low mobility, respectively.

#### 3.5.9 Dynamic cross-correlation matrix analysis (DCCM).

A protein’s structural sections move in different ways depending on the situation: some are very dynamic but uncorrelated, others are strongly correlated or anti-correlated, and some are comparatively static [85].

**Fig 10:**
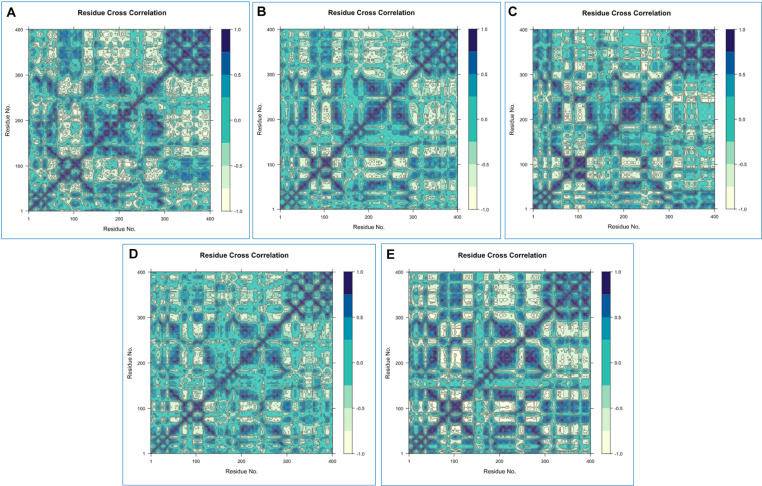
A comprehensive dynamic cross-correlation map where black indicates positive residue correlations and sea green denotes negative residue correlations. In this context, **(A)** CID: 359, **(B)** CID: 9064, **(C)** CID: 25310, **(D)** CID: 492405 (control), and **(E)** Apoprotein (2HG0) are referenced.

Now, information can be transferred via the residues with correlated or anti-correlated motions, which can create a network known as a dynamic cross-correlation network [11,85]. There are several time scales at which protein motions take place, from femtoseconds to seconds. In this analysis, we used inter-residue DCCM analysis, targeting the protein (2HG0) and its docked complexes’ correlated and anti-correlated motions with (A) CID: 359, (B) CID: 9064, (C) CID: 25310, (D) CID: 492405 (control), and (E) Apoprotein (2HG0), respectively. Furthermore, there are both positive and negative amino acid correlation effects shown in the ensuing dynamical cross-correlation graphs for the target protein 2HG0 distribution across the population. DCCM showed that the overall correlation fell between -1.0 and 1.0 (From sea green to dark blue) [85]. Diverse colors were also employed to represent different levels of linkage among residues, with deeper hues signifying greater relationships. A considerable association was identified between the target protein (2HG0) and the selected ligands CIDs: 9064 (B) and 25310 (D) as seen by the elevated pairwise cross-correlation coefficient on the cross-correlation map (**Fig 10**) relative to other complexes. As visualized, the illustration showed that the correlations approaching 1 signified that the residues were traveling in the same direction, but correlations nearing -1 suggested that the residues were migrating in the opposite direction [32]. All map movements had minor differences, suggesting that 2HG0 may possess analogous global dynamics both before and during compound binding. The correlation patterns across all plots are predominantly weakly distinct, suggesting that the motions of free 2HG0 and its ligand-bound complexes exhibit stability.

## 4. Discussion

WNV is a flavivirus transmitted by mosquitos that causes neuropathies in humans, horses, and birds. The majority of WNV illnesses in humans are subclinical, but they can also be clinical and range in severity from ordinary WN fever to deadly meningoencephalitis; the risk of severe neuroinvasive illness and mortality rises with age [86].

Currently, there is no effective drug that can prevent or cure WNV, despite the virus’s widespread propagation and epidemic character [87,88]. In comparison to HCV or DENV, the effort required for WNV medication development is significantly lower and this might be a result of the widespread belief that antivirals are not urgently needed to treat this disease [89]. In this study, we analyze the present status of developing drugs for WNV and various strategies have been performed to explore WNV antagonists, comprising structure-based virtual screening, and structure-based rationale design [89]. In this context, phytochemicals extracted from IMPPAT present a promising approach that could result in the identification of strong anti-WNV therapeutic compounds.

The discovery and development of new, potentially useful compounds have benefited greatly through the combination of computational and experimental approaches. By connecting computational predictions with real-world applicability, the revolutionization of drug discovery could be achieved by converting the results of *in silico* drug design into experimental validation. When compared to more conventional approaches, it allows for the rapid and cost-effective discovery of new medicinal candidates. Docking methods are widely employed in current drug design to study ligand conformations at macromolecular target binding sites. Important intermolecular recognition phenomena are evaluated to estimate the ligand-receptor binding free energy [90]. It is possible to aid researchers in early compound selection by using ADMET models to forecast the pharmacokinetic and toxicological properties of therapeutic candidates. MD simulations are useful for optimizing protein-receptor structure and flexibleness, refining docked complexes, calculating binding free energies, and ranking potential ligands which are useful for further experimental validation [91].

When it comes to finding new drugs, *in-silico* methods have some advantages but also limitations. Building comprehensive pre-computed databases of three-dimensional structures for protein or binding sites is a major limitation of inverse docking [37]. Due to the requirement for substantial computer resources and precise input structures, molecular docking and simulations have limits when it comes to mimicking solvent effects. MD simulations are sensitive to initial conditions and have a high computational cost. It is challenging to synthesise data due to the variety of sources, and interpreting results may be flawed due to an over-reliance on computer predictions without sufficient experimental validation [38].

In rational drug design, molecular docking is a standard process based on a structure that analyzes and predicts the attachment behaviors and binding qualities between the L-P compounds [92]. As previously reported, we used molecular docking to investigate 5375 phytochemicals derived from 32 medicinal plants against the WNV envelope glycoprotein (2HG0). Favipiravir, the control ligand, was utilized as a reference to compare against, and out of the compounds that were selected, three appeared to have a higher inclination to bind to the target protein in comparison to Favipiravir. The PubChem IDs for the three lead compounds, such as CID: 359, 9064, and 25310, with docking values of –7.464, –5.802, and -5.617 kcal/mol, correspondingly. These values notably exceeded the scores for the control ligand (CID: 492405), which exhibited a docking value of –4.932 kcal/mol, demonstrating the more promising binding potential of the discovered candidates. The post-docking MM-GBSA is an essential technique for measuring the binding energy of the complex due to the optimal combination of accuracy and efficiency [93]. Subsequently, we implemented post-docking MM-GBSA to calculate the negative binding energy of the P-L complex. According to the complex analysis, the negative binding free energies of the selected three compounds CID: 359, 9064, 25310, and 492405 (control) were -29.16, −33.45, −32.02, and -3.16 kcal/mol, correspondingly. Based on the information provided above, it can be concluded that the three compounds that were selected had the potential to bind to the envelope glycoprotein binding site (PDB ID: 2HG0) for longer periods than the control compound (CID: 492405).

Pk is the study of how drug concentrations change over time in different bodily fluids, taking into account the features of the drug in terms of its ADME. During the drug advancement process, it is imperative to balance the Pk properties in order to satisfy the necessary criteria set by clinical trials, hence determining the therapeutic candidate’s effectiveness. According to Lipinski’s rule, an orally active drug must satisfy the following criteria: MW < 500 g/mol, consensus logP < 5, H-bond-donating atoms < 5, and H-bond-accepting atoms < 10 [94]. The log P_o/w_, which reflects the partition coefficient of n-octanol and water, is a commonly used parameter in the study of lipophilicity [95]. The MW, consensus logP, H−bond−donating atoms, and H−bond−accepting atoms for CID: 359 are 126.11 g/mol, 0.45, 3, and 3, respectively. The values of 290.27 g/mol, 0.83, 5, and 6 are the MW, consensus logP, H−bond−donating atoms, and H−bond−accepting atoms for CID: 9064, respectively. In the case of CID: 25310, the MW, consensus logP, H-bond−donating atoms, and H-bond−accepting atoms are 164.16 g/mol, −1.36, 4, and 5, respectively. For CID: 492405, the MW is 157.1 g/mol, the consensus logP is −0.27, the number of H-bond-donating atoms is 2, and the number of H−bond−accepting atoms is 4. In this study, we can see that the top three selected phytochemical compounds (CID: 359, 9064, and 25310) reflect favorable absorption or accessibility according to the Lipinski rule. The drug’s solubility in n−octanol, a substance that resembles the lipid bilayer found in cell membranes, as well as in water, the fluid that is present both inside and outside cells, is fundamentally determined by this indicator. The solubility of a substance is greatly affected by the solvent that is employed combined with environmental factors such as temperature and pressure. The saturation concentration, or the point at which the solute’s concentration in the solution stays constant while more solute is added, is known as the range of solubility. Log S values for water solubility for three selected compounds (CID: 359, 9064, and 25310) and 492405 (control) are −1.22, −2.22, 0.46, and −0.8, accordingly. For CID 9064 and 25310, the BBB permeant findings are “No,” and for 492405 (the control), the result is “No” as well, indicating an excellent outcome and three lead compounds (CID: 359, 9064, and 25310) gastrointestinal absorption is “High”. The synthetic accessibility scores for CID: 359, 9064, 25310, and 492405 (control) are 1, 3.5, 4.05, and 2.08, correspondingly. This investigation indicates that the top selected compounds (CID: 359, 9064, and 25310) exhibit satisfactory responses.

In the drug design process, *in-silico* toxicity forecast is a crucial part of regulatory decision-making and the selection of leads, as *in vitro* or *in vivo* methods are frequently constrained by ethics, time, budget, and other resources. Taking advantage of technical advancements such as ProTox-3.0, which enables the acquisition of data from toxicity screening tools, serves as a method of conducting *in-silico* toxicity analysis. The organ toxicities for CID: 359 exhibit inactive hepatotoxicity and neurotoxicity, with probability rates of 0.78 and 0.75, respectively, while the chance of the nephrotoxicity (0.61), and cardiotoxicity (0.85) effects is minimal. If we lower the toxicity, it may be the ideal drug. Substances known as carcinogens have the capacity to cause cancer or raise the risk of tumor growth, the probability is 0.73 and the carcinogenicity prediction is inactive. Immunotoxicity is a term used to describe adverse consequences on the immune system that remain inactive with a likelihood of 0.99. Mutagens are agents that cause abnormal genetic modifications, it is inactive, and the mutagenicity probability is 0.97. When examining substances for the possibility of both intended and undesired cell damage, it is imperative to forecast their cytotoxicity, the probability is 0.95, and the forecast is inactive. CID: 9064 showed most of the inactive organ toxicities, which is an impressive result for finding a drug and the probability rates are as follows: hepatotoxicity (0.72), neurotoxicity (0.90), nephrotoxicity (0.62), and cardiotoxicity (0.99). For carcinogenicity, immunotoxicity, mutagenicity, and cytotoxicity, the probability of the toxicological endpoints being inactive is 0.51, 0.96, 0.55, and 0.84, respectively. CID: 25310 demonstrated the majority of inactive organ toxicities and hepatotoxicity (0.85), neurotoxicity (0.93), nephrotoxicity (0.66), and cardiotoxicity (0.62) are the probability rates. The toxicological endpoints including carcinogenicity, immunotoxicity, mutagenicity, and cytotoxicity all have a likelihood of being inactive: 0.61, 0.97, 0.73, and 0.78. The organ toxicity likelihood rates for CID: 492405 (control) are as follows: neurotoxicity (0.80), nephrotoxicity (0.53), cardiotoxicity (0.80), and hepatotoxicity (0.66). For immunotoxicity, mutagenicity, and cytotoxicity, the probability of the toxicological endpoints being inactive is 0.99, 0.76, and 0.84, respectively. Here, we found that the carcinogenicity (0.53) probability is active, meaning that it may cause cancer or increase the risk of tumor growth, which is not suitable for a perfect medication. This computational approach accurately predicted the selected compounds’ minimum probability and intrinsic security than the control.

To help identify the most promising compounds for further research, MD simulations were utilized to examine the protein-ligand interactions, stability, and conformational changes at several time intervals [96]. The three selected compounds, together with the ligand, have been evaluated in terms of, RMSD, RMSF, Rg, SASA values, P-L interaction, and L-P interaction. The ideal sustainability of the compounds is shown by the RMSD values of the complex systems. The average RMSD values for CID: 359, 9064, 25310, and 492405 (control) were 4.42 **Å**, 3.48 **Å**, 6.28 **Å**, and 3.87 **Å**, respectively. According to the research, CID: 9064 surpassed the other two molecules (CID: 359 and 25310) and the control (CID: 492405) in terms of providing satisfactory outcomes when complexed with apoprotein through the last step of the simulation period and maintained consistent fluctuation most of the time. In contrast, the RMSF scores evaluate the average fluctuation that indicates the robustness of the molecules to the target macromolecule and characterizes the uniformity of the protein-ligand association [97]. The average RMSF scores for the chosen compounds CID: 359, 9064, 25310, and 492405 (control) were 2.76 **Å**, 2.14 **Å**, 3.12 **Å**, and 2.05 **Å**, respectively. According to these findings, the binding of CID: 9064 appears to cause very slight modifications in protein structure. During the MD simulation, three selected compounds were analyzed, and it was observed that the compound with CID: 9064 exhibited the smallest RMSD and RMSF scores, as well as the smallest fluctuation when associated with the apoprotein. The three lead compounds (CID: 359, 9064, and 25310) were evaluated based on SASA values, P-L, and L-P interaction. The Rg is a fruitful technique for understanding the folding and packing properties of proteins and ligand-protein complexes. Higher compactness is symbolized by a lower Rg value, whereas compound disengagement from the protein is indicated by a higher value [98]. The compounds with the following Rg values were determined: 2.089 **Å**, 3.693 **Å**, 2.168 **Å**, and 2.272 **Å** for CID: 359, 9064, 25310, and 492405 (control). Strongly condensed mixtures of amino acid residues and water molecules are indicated by a lower SASA value, whereas a greater value is reflective of a weak structure [11,99]. The protein’s average SASA values when associated with the compounds CID: 359, 9064, 25310, and 492405 (control) were 89.24 **Å**^2^, 179.86 **Å**^2^, 234.25 **Å**^2^, and 131.75 **Å**^2^, correspondingly. The statistical technique of principal component analysis (PCA) was used to reduce the dimensionality of a complicated dataset while preserving the most important information [100]. During our investigation, we looked at each and every collective trajectory that the MD simulation produced as output files. According to the results of the PCA, sets of PCs (PC1, PC2, and PC3) were charted where the complex 2HG0-CID: 359 had the highest PC3 value, which was 9.99%, indicating that it was less stable than the other complexes. In contrast to the control and other complexes, the PC3 values of 2HG0-CID: 9064 and 2HG0-CID: 25310 were found to be 8.82% and 3.77%, respectively. Their PC3 values were found to be lower showing their stability and had a compact structure than PC1 and PC2. A PC3 score of 5.72% was also observed for the Apoprotein, which indicated a high level of stability. On the other hand, DCCM assessed the correlation coefficient between the atom or residue displacement vectors, identifying how different parts of the system are coordinated or differentially moving with respect to each other. Hence, the analyses provided a substantial correlation between the target protein and the phytocompounds CID: 9064 and CID: 25310, as demonstrated by the elevated pairwise cross-correlation coefficient value on the cross-correlation map in comparison to other complexes. According to the findings of the PCA and DCCM analyses, the 2HG0-CID: 25310 and 2HG0-CID: 9064 complexes exhibited less variability and better robustness, even better than the control ligand 2HG0-CID: 492405 (Favipiravir) and other protein-ligand interacting complexes.

In this research, three compounds have been examined using several computational evaluations: CID: 359 (Phloroglucinol), 9064 (Cianidanol), and 25310 (L-Rhamnose) in relation to the control drug, Favipiravir. Cianidanol (CID: 9064) and L-Rhamnose (CID: 25310) demonstrate prominent findings in docking, post-docking MM-GBSA, and MD simulation (RMSD, RMSF, Rg, SASA, H-bond, P-L contact, L-P contact, PCA, and DCCM) analysis. Based on the above findings, these two phytochemical therapeutic candidates, which are bioactive compounds derived from plants, have the greatest potential to be effective against WNV infection, as they can suppress the WNV glycoprotein and prevent WNV replication during the initial stages of infection.

The phytochemical therapeutic candidate, Cianidanol, is found in the *Tamarindus indica* (63) plant along with *Abutilon theophrasti, Acacia catechu, Acacia decurrens, Acacia farnesiana, Acacia nilotica, Acacia planifrons, Acacia pubescens, Albizia lebbeck, Alhagi maurorum, Anacardium occidentale, Annona squamosa, Arachis hypogaea, Arctostaphylos uva-ursi, Areca catechu, Arnica Montana, Artocarpus heterophyllus, Azadirachta indica, Bergenia ciliate, Bergenia purpurascens, Caesalpinia coriaria, Camellia sinensis, Cassia fistula, Cassia roxburghii, Casuarina equisetifolia, Catha edulis, Catharanthus roseus, Ceratonia siliqua, Ceriops decandra, Cinnamomum cassia, Cleistanthus collinus, Cola nitida, Colophospermum mopane, Corymbia calophylla, Crataegus rhipidophylla, Crataegus songarica, Cryptomeria japonica, Dendrophthoe falcate, Elaeagnus angustifolia, Ephedra gerardiana, Eriobotrya japonica, Eucalyptus alba, Eucalyptus globules, Eucalyptus hybrid, Eucalyptus tereticornis, Fagopyrum esculentum, Flacourtia indica, Fragaria vesca, Garcinia mangostana, Geranium pretense, Geum urbanum, Ginkgo biloba, Gleditsia triacanthos, Glycine max, Grewia asiatica, Hardwickia binata, Hordeum vulgare, Hypericum japonicum, Hypericum perforatum, Juniperus communis, Kandelia candel, Kandelia rheedii, Krameria lappacea, Laurus nobilis, Leonurus cardiac, Lepisanthes rubiginosa, Magnolia champaca, Mangifera indica, Manilkara zapota, Melia azedarach, Metasequoia glyptostroboides, Moringa oleifera, Myrica esculenta, Ocimum tenuiflorum, Pinus massoniana, Pinus sylvestris, Pinus wallichiana, Platycladus orientalis, Polygonum aviculare, Polypodium vulgare, Pseudotsuga menziesii, Psidium guajava, Quercus glauca, Quercus ilex, Quercus robur, Rheum officinale, Rheum palmatum, Rhododendron ponticum, Ribes nigrum, Robinia pseudoacacia, Rosa canina, Rosa cymosa, Rosa multiflora, Rotheca serrata, Rumex acetosella, Rumex crispus, Salix alba, Salix caprea, Santolina chamaecyparissus, Saraca asoca, Senna auriculata, Terminalia catappa, Uncaria gambir, Viburnum opulus, Vigna angularis, Vitis vinifera* and L-Rhamnose is present in the *Plantago ovate* (66) plant along with *Abelmoschus esculentus, Acacia auriculiformis, Acacia catechu, Acacia chundra, Acacia concinna, Acacia decurrens, Acacia nilotica, Aconitum violaceum, Actinidia chinensis, Aegle marmelos, Agave americana, Agave cantala, Albizia amara, Albizia julibrissin, Albizia lebbeck, Albizia odoratissima, Albizia procera, Allium ascalonicum, Allium cepa, Allium sativum, Aloe vera, Alternanthera philoxeroides, Ambroma augusta, Ammi visnaga, Amorphophallus paeoniifolius, Anchusa strigosa, Anogeissus latifolia, Arachis hypogaea, Araucaria columnaris, Asparagus adscendens, Asparagus racemosus, Avena sativa, Azadirachta indica, Balanites aegyptiaca, Barringtonia racemosa, Bellis perennis, Benincasa hispida, Blighia sapida, Boerhavia diffusa, Bombax ceiba, Brassica rapa, Calotropis procera, Canavalia ensiformis, Canavalia gladiate, Carthamus tinctorius, Cassia javanica, Catunaregam spinosa, Ceiba pentandra, Centella asiatica, Cheilocostus speciosus, Cinchona calisaya, Citrus mitis, Clitoria mariana, Coffea arabica, Convolvulus microphyllus, Cordia dichotoma, Cordia sinensis, Coriandrum sativum, Cyclea peltata, Cynanchum auriculatum, Delonix regia, Dianthus barbatus, Dicliptera chinensis, Diospyros discolor, Elaeagnus rhamnoides, Euphorbia heterophylla, Euphorbia hirta, Flagellaria indica, Ginkgo biloba, Gmelina arborea, Gossypium barbadense, Helianthus annuus, Herniaria glabra, Heterophragma quadriloculare, Hibiscus rosa-sinensis, Hibiscus syriacus, Indigofera arrecta, Ipomoea alba, Ipomoea indica, Ipomoea purpurea, Ipomoea quamoclit, Jatropha curcas, Jatropha gossypiifolia, Lagenaria siceraria, Lallemantia royleana, Lannea coromandelica, Lepidium sativum, Lepidium virginicum, Luffa cylindrical, Luffa echinata, Madhuca longifolia, Magnolia grandiflora, Malva sylvestris, Mangifera indica, Matricaria chamomilla, Mimusops elengi, Moringa oleifera, Nerium oleander, Nicotiana tabacum, Ochrosia oppositifolia, Ocimum americanum, Ocimum filamentosum, Oldenlandia diffusa, Operculina turpethum, Panax ginseng, Paris polyphylla, Persicaria pulchra, Phoenix dactylifera, Plantago lanceolata, Plantago ovate, Prosopis cineraria, Prunus domestica, Psidium guajava, Pterospermum acerifolium, Pterospermum canescens, Punica granatum, Rhamnus virgata, Saccharum bengalense, Salix tetrasperma, Salvia aegyptiaca, Scindapsus officinalis, Sesbania grandiflora, Seseli diffusum, Spilanthes acmella, Syzygium aromaticum, Terminalia alata, Terminalia bellirica, Tribulus pentandrus, Tribulus terrestris, Ulmus wallichiana, Vallisneria spiralis, Viola odorata, Vitis vinifera, Ziziphus jujube*. Isolation, synthesis, and modification of the two compounds, Cianidanol and L-Rhamnose, pose many challenges. It is hard to extract large quantities because the compounds are usually available in minute amounts in the environment. Production costs may be high because the raw materials used to synthesize Cianidanol are expensive or require intensive pretreatment. The synthesis of L-rhamnose requires a complex procedure that is expensive, time-consuming, and uses hazardous chemicals that can affect the environment. Previous research has shown the potential of Cianidanol in the prevention and treatment of breast cancer [64] and Parkinson’s disease [65] while L-Rhamnose has demonstrated antitumor and anticancer [67] promise but we found their antiviral efficacy in our investigation.

The investigations both *in vivo* and *in vitro* are essential for confirming that potential drugs act efficiently against target proteins. Since there are limited funds, insufficient equipment, and safety precautions for validating antiviral drugs, further research is required to surely affirm compound-protein relationships. Our *in-silico* modeling promotes the *in vitro* research conducted on different diseases, bolstering our knowledge and eventually facilitating the advancement of treatments.

## 5 Conclusion

The underlying cause of WN fever and neuroinvasive illness among individuals is WNV, and its capacity to infect vulnerable cells is facilitated by E glycoprotein. At this moment, no such drugs may inhibit virus attachment and prevent further replication. Our research’s objective was to identify and screen novel candidates for naturally occurring inhibitors that can block the WNV envelope glycoprotein and eradicate the WNV infection. A range of *in-silico* methods were performed, such as ADMET, molecular docking, post-docking MM-GBSA, MD simulation, PCA, and DCCM, were used for several compounds, with the most promising treatment candidates being identified as L-Rhamnose (CID: 25310) and Cianidanol (CID: 9064). This suggests that *in-vivo* testing in animal models (such as mice models) of the disease is essential to validate therapeutic promise, while *in-vitro* testing in illness-relevant cell lines or 3D organoids is required to replicate the physiological setting for efficacy evaluation. As a result, in order to establish them (Cianidanol and L-Rhamnose) as a possible drug candidate for future use, more experimental research needs to be carried out to ascertain their usefulness.

Keywords

**Table:** 

Terms	Detailed Information
OPLS-3e force field	The OPLS-3e force field is a sophisticated computer model which provides exceptional performance in protein simulations and accurately predicts protein-ligand binding affinities across a diverse array of targets and ligands [46].
SPC water model	The simple point charge extended model for water is a reliable model in MD simulations that predicts a rigid water molecule with an O-H distance of 0.1 nm and an angle of 109.47° between the O-H bonds.
NPT ensemble	In MD simulations, the NPT ensemble (fixed number of particles, temperature, and pressure) is frequently used to examine systems at equilibrium under experimentally-like circumstances, such as atmospheric pressure and a particular temperature.
SID tool	In MD simulations, the Simulation Interactions Diagram (SID) is a tool used to examine and illustrate relationships within a system, particularly those between ligands and proteins.

## Supporting information

S1 file List of plants, Phytochemicals along with CID, IMPPAT identifier. (DOCX)

S2 file Docking results. (CSV)

S3 file Post-docking MM-GBSA scores. (XLSX)

S4 file Protein RMSD. (XLSX)

S5 file Protein RMSF. (XLSX)

S6 file H-bond analysis. (XLSX)

S7 file Ligand properties. (XLSX)
