# Supplementary material for: Molecular activity of bioactive phytocompounds for inhibiting host cell attachment and membrane fusion interacting with West Nile Virus envelope glycoprotein
Source: PLoS One. 2025 Apr 24;20(4):e0321902. doi: 10.1371/journal.pone.0321902 (PMC12021142; doi:10.1371/journal.pone.0321902)
Supplement: S1 file — (DOCX) [file pone.0321902.s001.docx]

**Molecular Activity of Bioactive Phytocompounds for Inhibiting Host Cell Attachment and Membrane Fusion Interacting with West Nile Virus Envelope Glycoprotein**

**Supplementary file:** List of plants, Phytochemicals along with CID, IMPPAT identifier

|  |  |  | **Plant Name: *Panax quinquefolius*** |  |
| --- | --- | --- | --- | --- |
| **Serial No** | **IMPPAT Phytochemical Identifier** | **Phytochemical Name** | **SMILES** | **CID** |
| 1 | IMPHY000309 | Dotriacontane | CCCCCCCCCCCCCCCCCCCCCCCCCCCCCCCC | 11008 |
| 2 | IMPHY000399 | beta-Bisabolene | CC(=CCCC(=C)[C@H]1CCC(=CC1)C)C | 10104370 |
| 3 | IMPHY001896 | Heptacosane | CCCCCCCCCCCCCCCCCCCCCCCCCCC | 11636 |
| 4 | IMPHY001915 | Octadecane | CCCCCCCCCCCCCCCCCC | 11635 |
| 5 | IMPHY003983 | Ginsenoside rb1 | OC[C@H]1O[C@@H](O[C@H]2CC[C@]3([C@H](C2(C)C)CC[C@@]2([C@@H]3C[C@@H](O)[C@H]3[C@@]2(C)CC[C@@H]3[C@@](O[C@@H]2O[C@H](CO[C@@H]3O[C@H](CO)[C@H]([C@@H]([C@H]3O)O)O)[C@H]([C@@H]([C@H]2O)O)O)(CCC=C(C)C)C)C)C)[C@@H]([C@H]([C@@H]1O)O)O[C@@H]1O[C@H](CO)[C@H]([C@@H]([C@H]1O)O)O | 9898279 |
| 6 | IMPHY004273 | Ginsenoside RG1 | OC[C@H]1O[C@@H](O[C@H]2C[C@]3(C)[C@@H]([C@@]4([C@@H]2C(C)(C)[C@@H](O)CC4)C)C[C@H]([C@H]2[C@@]3(C)CC[C@@H]2[C@@](O[C@@H]2O[C@H](CO)[C@H]([C@@H]([C@H]2O)O)O)(CCC=C(C)C)C)O)[C@@H]([C@H]([C@@H]1O)O)O | 441923 |
| 7 | IMPHY006736 | 8-[(2R,3S)-3-heptyloxiran-2-yl]octa-4,6-diyn-3-yl acetate | CCCCCCC[C@@H]1O[C@@H]1CC#CC#CC(OC(=O)C)CC | 101618813 |
| 8 | IMPHY006901 | (3S,5R,6S,8R,9R,10R,12R,13R,14R,17S)-4,4,8,10,14-pentamethyl-17-[(2R)-2,6,6-trimethyloxan-2-yl]-2,3,5,6,7,9,11,12,13,15,16,17-dodecahydro-1H-cyclopenta[a]phenanthrene-3,6,12-triol | O[C@H]1C[C@]2(C)[C@@H]([C@@]3([C@@H]1C(C)(C)[C@@H](O)CC3)C)C[C@H]([C@H]1[C@@]2(C)CC[C@@H]1[C@@]1(C)CCCC(O1)(C)C)O | 12313935 |
| 9 | IMPHY006951 | Eicosane | CCCCCCCCCCCCCCCCCCCC | 8222 |
| 10 | IMPHY006971 | Methyl palmitate | CCCCCCCCCCCCCCCC(=O)OC | 8181 |
| 11 | IMPHY007012 | Panaxadiol | O[C@@H]1C[C@@H]2[C@@]3(C)CC[C@@H](C([C@@H]3CC[C@]2([C@]2([C@H]1[C@H](CC2)[C@@]1(C)CCCC(O1)(C)C)C)C)(C)C)O | 73498 |
| 12 | IMPHY007327 | Palmitic acid | CCCCCCCCCCCCCCCC(=O)O | 985 |
| 13 | IMPHY007520 | Viridiflorene | C[C@@H]1CCC2=C(C)CC[C@@H]3[C@H]([C@H]12)C3(C)C | 10910653 |
| 14 | IMPHY008835 | Ginsenoside rb3 | CC/C(=C/CC[C@](C1CC[C@]2(C1[C@@H](O)CC1C2CCC2[C@]1(C)CCC(C2(C)C)O[C@@H]1O[C@H](CO)[C@H]([C@@H]([C@H]1O[C@@H]1O[C@H](CO)[C@H]([C@@H]([C@H]1O)O)O)O)O)C)(O[C@@H]1O[C@H](CO[C@@H]2OC[C@H]([C@@H]([C@H]2O)O)O)[C@H]([C@H]([C@H]1O)O)O)C)/C |  |
| 15 | IMPHY009355 | Tetracosane | CCCCCCCCCCCCCCCCCCCCCCCC | 12592 |
| 16 | IMPHY009359 | Hexacosane | CCCCCCCCCCCCCCCCCCCCCCCCCC | 12407 |
| 17 | IMPHY009369 | Nonadecane | CCCCCCCCCCCCCCCCCCC | 12401 |
| 18 | IMPHY009370 | Ginsenoside Rc | OC[C@H]1O[C@@H](O[C@H]2CC[C@]3([C@H](C2(C)C)CC[C@@]2([C@@H]3C[C@@H](O)[C@H]3[C@@]2(C)CC[C@@H]3[C@@](O[C@@H]2O[C@H](CO[C@@H]3O[C@H]([C@@H]([C@H]3O)O)CO)[C@H]([C@@H]([C@H]2O)O)O)(CCC=C(C)C)C)C)C)[C@@H]([C@H]([C@@H]1O)O)O[C@@H]1O[C@H](CO)[C@H]([C@@H]([C@H]1O)O)O | 12855889 |
| 19 | IMPHY009375 | Docosane | CCCCCCCCCCCCCCCCCCCCCC | 12405 |
| 20 | IMPHY009377 | Pentacosane | CCCCCCCCCCCCCCCCCCCCCCCCC | 12406 |
| 21 | IMPHY009382 | Heneicosane | CCCCCCCCCCCCCCCCCCCCC | 12403 |
| 22 | IMPHY009413 | Triacontane | CCCCCCCCCCCCCCCCCCCCCCCCCCCCCC | 12535 |
| 23 | IMPHY009481 | Octacosane | CCCCCCCCCCCCCCCCCCCCCCCCCCCC | 12408 |
| 24 | IMPHY009482 | Nonacosane | CCCCCCCCCCCCCCCCCCCCCCCCCCCCC | 12409 |
| 25 | IMPHY009490 | Tricosane | CCCCCCCCCCCCCCCCCCCCCCC | 12534 |
| 26 | IMPHY009624 | Ethyl palmitate | CCCCCCCCCCCCCCCC(=O)OCC | 12366 |
| 27 | IMPHY009706 | Isoledene | CC1CCC2C(C3=C1CCC3C)C2(C)C | 530426 |
| 28 | IMPHY010080 | beta-Elemene | C=C[C@]1(C)CC[C@H](C[C@H]1C(=C)C)C(=C)C | 6918391 |
| 29 | IMPHY010585 | (1aR,7R,7aR,7bS)-1,1,7,7a-tetramethyl-2,3,5,6,7,7b-hexahydro-1aH-cyclopropa[a]naphthalene | C[C@@H]1CCC=C2[C@@]1(C)[C@H]1[C@H](C1(C)C)CC2 | 15560278 |
| 30 | IMPHY010906 | Pseudoginsenoside FII | OC[C@H]1O[C@@H](O[C@H]2C[C@]3(C)[C@@H]([C@@]4([C@@H]2C(C)(C)[C@@H](O)CC4)C)C[C@H]([C@H]2[C@@]3(C)CC[C@@H]2[C@]2(C)CC[C@@H](O2)C(O)(C)C)O)[C@@H]([C@H]([C@@H]1O)O)O[C@@H]1O[C@@H](C)[C@@H]([C@H]([C@H]1O)O)O | 21633072 |
| 31 | IMPHY010932 | (3S,4S,5S,6R)-2-[(3R,4S,5S,6R)-2-[[(3S,6S,8R,9R,10R,12R,17S)-3,12-dihydroxy-4,4,8,10,14-pentamethyl-17-[(2S)-6-methyl-2-[(3R,4S,5S,6R)-3,4,5-trihydroxy-6-(hydroxymethyl)oxan-2-yl]oxyhept-5-en-2-yl]-2, | OC[C@H]1OC(O[C@H]2C[C@]3(C)[C@@H]([C@@]4(C2C(C)(C)[C@@H](O)CC4)C)C[C@H](C2C3(C)CCC2[C@@](OC2O[C@H](CO)[C@H]([C@@H]([C@H]2O)O)O)(CCC=C(C)C)C)O)[C@@H]([C@H]([C@@H]1O)O)OC1O[C@H](C)[C@H]([C@@H]([C@@H]1O)O)O | 122130479 |
| 32 | IMPHY011084 | (1S,6S,7R)-2,6,8,8-Tetramethyltricyclo[5.2.2.01,6]undec-2-ene | CC1=CCC[C@@]2([C@@]31CC[C@@H]2C(C3)(C)C)C | 15973057 |
| 33 | IMPHY011454 | Gypenoside VIII | OC[C@H]1O[C@@H](O[C@H]2CC[C@]3([C@H](C2(C)C)CC[C@@]2([C@@H]3C[C@@H](O)[C@H]3[C@@]2(C)CC[C@@H]3[C@@](O[C@@H]2O[C@H](CO)[C@H]([C@@H]([C@H]2O)O)O)(CCC=C(C)C)C)C)C)[C@@H]([C@H]([C@@H]1O)O)O[C@@H]1O[C@H](CO)[C@H]([C@@H]([C@H]1O)O)O | 11679800 |
| 34 | IMPHY011548 | Falcarinol | CCCCCCC/C=CCC#CC#C[C@@H](C=C)O | 5281149 |
| 35 | IMPHY011658 | beta-Farnesene | C=CC(=C)CC/C=C(/CCC=C(C)C)C | 5281517 |
| 36 | IMPHY011667 | alpha-Gurjunene | C[C@@H]1CC[C@@H]2[C@H](C3=C(CC[C@H]13)C)C2(C)C | 15560276 |
| 37 | IMPHY011749 | Humulene epoxide II | C/C/1=CCC(C)(C)/C=C/C[C@@]2([C@@H](CC1)O2)C | 10704181 |
| 38 | IMPHY011761 | Humulene | C/C/1=CCC(C)(C)/C=C/C/C(=C/CC1)/C | 5281520 |
| 39 | IMPHY011793 | (+)-gamma-Cadinene | CC1=C[C@@H]2[C@@H](CC1)C(=C)CC[C@H]2C(C)C | 6432404 |
| 40 | IMPHY011826 | Oleanolic acid | O[C@H]1CC[C@]2([C@H](C1(C)C)CC[C@@]1([C@@H]2CC=C2[C@@]1(C)CC[C@@]1([C@H]2CC(C)(C)CC1)C(=O)O)C)C | 10494 |
| 41 | IMPHY011957 | (+)-delta-Cadinene | CC1=C[C@@H]2C(=C(C)CC[C@H]2C(C)C)CC1 | 441005 |
| 42 | IMPHY012342 | 8-(3-Heptyloxiran-2-yl)oct-1-en-4,6-diyn-3-yl acetate | CCCCCCCC1OC1CC#CC#CC(OC(=O)C)C=C | 129509 |
| 43 | IMPHY012667 | Caryophyllene oxide | C=C1CC[C@H]2O[C@@]2(CC[C@@H]2[C@@H]1CC2(C)C)C | 1742210 |
| 44 | IMPHY014150 | Panaquinquecol 7 | CCCCCCCC1OC1CC#CC#CC(=O)CCO | 9970687 |
| 45 | IMPHY014178 | Panaquinquecol 4 | CCCCCCCC1OC1CC#CC#CC(=O)C1CO1 | 10468587 |
| 46 | IMPHY014448 | Panaquinquecol 6 | CC(=O)OC(C#CC#CC(C=C)O)C1OC1CCCCCCC | 57489663 |
| 47 | IMPHY014690 | (-)-Globulol | C[C@@H]1CC[C@@H]2[C@@H]1[C@H]1[C@H](C1(C)C)CC[C@@]2(C)O | 12304985 |
| 48 | IMPHY014708 | beta-Selinene | C=C1CCC[C@]2([C@H]1C[C@@H](CC2)C(=C)C)C | 442393 |
| 49 | IMPHY014785 | (E)-2-epi-beta-caryophyllene | C/C/1=CCCC(=C)[C@H]2[C@@H](CC1)C(C2)(C)C | 6429274 |
| 50 | IMPHY014831 | beta-Caryophyllene | C/C/1=CCCC(=C)[C@@H]2[C@@H](CC1)C(C2)(C)C | 5281515 |
| 51 | IMPHY015128 | T-Muurolol | CC1=C[C@@H]2[C@H](CC1)[C@@](C)(O)CC[C@H]2C(C)C | 3084331 |
| 52 | IMPHY016919 | Ginsenoside Rb2 | OC[C@H]1O[C@@H](O[C@H]2CC[C@]3([C@H](C2(C)C)CC[C@@]2([C@@H]3C[C@@H](O)[C@H]3[C@@]2(C)CC[C@@H]3[C@@](O[C@@H]2O[C@H](CO[C@@H]3OC[C@@H]([C@@H]([C@H]3O)O)O)[C@H]([C@@H]([C@H]2O)O)O)(CCC=C(C)C)C)C)C)[C@@H]([C@H]([C@@H]1O)O)O[C@@H]1O[C@H](CO)[C@H]([C@@H]([C@H]1O)O)O | 6917976 |
|  |  |  |  |  |
| **PLANT NAME : *Phyllanthus amarus*** | | | | |
| **Serial No** | **IMPPAT Phytochemical Identifier** | **Phytochemical Name** | **SMILES** | **CID** |
| 1 | IMPHY000423 | 4-Methoxynorsecurinine | COC1CN2[C@H](C1)[C@@]13C[C@H]2C=CC3=CC(=O)O1 | 101091319 |
| 2 | IMPHY000494 | Phyllanthenol | CC(=C)CCC[C@@H]([C@@H]1CC[C@]2([C@@]1(C)CC[C@H]1[C@H]2CC[C@@H]2[C@]1(C)[C@H](O)CC(=O)C2(C)C)C)C |  |
| 3 | IMPHY001664 | 24-Isopropyl-cholest-5-en-3beta-ol | O[C@H]1CC[C@]2(C(=CC[C@@H]3[C@@H]2CC[C@]2([C@H]3CC[C@@H]2[C@@H](CCC(C(C)C)C(C)C)C)C)C1)C | 14079080 |
| 4 | IMPHY002413 | Phyllanthol | C[C@@H]1CC[C@]2([C@@H]([C@H]1C)[C@@]13CC[C@H]4[C@@]([C@]3(C1)CC2)(C)CC[C@@H]1[C@]4(C)CC[C@@H](C1(C)C)O)C | 101600095 |
| 5 | IMPHY003349 | Phyllnirurin | OCCCc1cc2c(c(c1)OC)O[C@@H]([C@H]2C)c1ccc2c(c1)OCO2 | 179963 |
| 6 | IMPHY003390 | Hypophyllanthin | COC[C@@H]1Cc2cc(OC)c3c(c2[C@@H]([C@H]1COC)c1ccc(c(c1)OC)OC)OCO3 | 182140 |
| 7 | IMPHY005092 | Dotriacontanoic acid | CCCCCCCCCCCCCCCCCCCCCCCCCCCCCCCC(=O)O | 19255 |
| 8 | IMPHY006086 | Nirphyllin | COC[C@@H]([C@@H](Cc1cc(OC)c(c(c1)OC)O)COC)Cc1cc(OC)c2c(c1)OCO2 | 5491556 |
| 9 | IMPHY006681 | (1R,2R,4R,8S)-4-methoxy-14-oxa-7-azatetracyclo[6.6.1.01,11.02,7]pentadeca-9,11-dien-13-one | CO[C@@H]1CCN2[C@H](C1)[C@@]13OC(=O)C=C3C=C[C@@H]2C1 | 71751660 |
| 10 | IMPHY014836 | beta-Sitosterol | CC[C@@H](C(C)C)CC[C@H]([C@H]1CC[C@@H]2[C@]1(C)CC[C@H]1[C@H]2CC=C2[C@]1(C)CC[C@@H](C2)O)C | 222284 |
| 11 | IMPHY015047 | Rutin | Oc1cc(O)c2c(c1)oc(c(c2=O)O[C@@H]1O[C@H](CO[C@@H]2O[C@@H](C)[C@@H]([C@H]([C@H]2O)O)O)[C@H]([C@@H]([C@H]1O)O)O)c1ccc(c(c1)O)O | 5280805 |
| 12 | IMPHY000014 | Lintetralin | COC[C@H]1Cc2cc(OC)c(cc2[C@H]([C@@H]1COC)c1ccc2c(c1)OCO2)OC | 11361584 |
| 13 | IMPHY000162 | Phyltetralin | COC[C@H]1Cc2cc(OC)c(cc2[C@H]([C@@H]1COC)c1ccc(c(c1)OC)OC)OC | 11223782 |
| 14 | IMPHY002597 | 6-[(2R,3R)-3-[(3,4-dimethoxyphenyl)methyl]-4-methoxy-2-(methoxymethyl)butyl]-4-methoxy-1,3-benzodioxole | COC[C@@H]([C@H](Cc1ccc(c(c1)OC)OC)COC)Cc1cc(OC)c2c(c1)OCO2 | 13989915 |
| 15 | IMPHY003246 | Phyllanthin | COC[C@H]([C@H](Cc1ccc(c(c1)OC)OC)COC)Cc1ccc(c(c1)OC)OC | 358901 |
| 16 | IMPHY003390 | Hypophyllanthin | COC[C@@H]1Cc2cc(OC)c3c(c2[C@@H]([C@H]1COC)c1ccc(c(c1)OC)OC)OCO3 | 182140 |
| 17 | IMPHY006362 | Ascorbic acid | OC[C@@H]([C@H]1OC(=O)C(=C1O)O)O | 54670067 |
| 18 | IMPHY006813 | Nirtetralin | COC[C@@H]1Cc2cc3OCOc3c(c2[C@@H]([C@H]1COC)c1ccc(c(c1)OC)OC)OC | 10477792 |
| 19 | IMPHY010965 | Corilagin | O[C@@H]1[C@H]2COC(=O)c3cc(O)c(c(c3-c3c(C(=O)O[C@@H]1[C@H]([C@@H](O2)OC(=O)c1cc(O)c(c(c1)O)O)O)cc(O)c(c3O)O)O)O | 73568 |
| 20 | IMPHY000381 | Fraternusterol | O[C@@H]1CC/C=C/[C@@H]2/C(=CC[C@H](C)[C@H](CCC2)[C@@H](C(=O)C[C@@H](C(C)C)C)C)/[C@H](CC1)C | 100951228 |
| 21 | IMPHY000383 | Phyllanthosecosteryl ester | CCCCCCCCC(CCCCCCCCC(CCCCCC(=O)O[C@H]1CC[C@H](/C(=C/C[C@@H]2C=CC[C@]3([C@H]2CC[C@@H]3[C@@H](CC[C@H](C(C)C)CC)C)C)/C1)C)O)O | 100951226 |
| 22 | IMPHY000461 | Phthalic acid bis ester | CC(COC(=O)c1ccccc1C(=O)OCC(CCC(C)C)C)CCC(C)C |  |
| 23 | IMPHY005664 | Heptacosanoic acid | CCCCCCCCCCCCCCCCCCCCCCCCCCC(=O)O | 23524 |
| 24 | IMPHY010320 | Phyllanthostigmasterol | CC[C@@H](C(C)C)C/C=C(/[C@H]1CCC[C@H]2[C@H](CC[C@@H]1C)[C@@]1(C)CC[C@@H](CC1=CC2)O)C | 100951227 |
| 25 | IMPHY010331 | Phyllanthosterol | CC[C@@H](C(C)C)C/C=C(/[C@H]1CCC[C@H]2[C@H](CC[C@@H]1C)[C@@]1(C)CC[C@H](CC1=CC2)O)C | 100951225 |
| 26 | IMPHY006466 | Ricinoleic acid | CCCCCC[C@H](C/C=CCCCCCCCC(=O)O)O | 643684 |
| 27 | IMPHY012723 | Linolenic acid | CC/C=CC/C=CC/C=CCCCCCCCC(=O)O | 5280934 |
| 28 | IMPHY014990 | Linoleic acid | CCCCC/C=CC/C=CCCCCCCCC(=O)O | 5280450 |
| 29 | IMPHY000112 | Isophytol | C=CC(CCCC(CCCC(CCCC(C)C)C)C)(O)C | 10453 |
| 30 | IMPHY000308 | Hexadecane | CCCCCCCCCCCCCCCC | 11006 |
| 31 | IMPHY000795 | Octanal | CCCCCCCC=O | 454 |
| 32 | IMPHY001135 | 6,10,14-Trimethylpentadecan-2-one | CC(CCCC(C)C)CCCC(CCCC(=O)C)C | 10408 |
| 33 | IMPHY001896 | Heptacosane | CCCCCCCCCCCCCCCCCCCCCCCCCCC | 11636 |
| 34 | IMPHY001915 | Octadecane | CCCCCCCCCCCCCCCCCC | 11635 |
| 35 | IMPHY003246 | Phyllanthin | COC[C@H]([C@H](Cc1ccc(c(c1)OC)OC)COC)Cc1ccc(c(c1)OC)OC | 358901 |
| 36 | IMPHY003316 | Pentadecanal | CCCCCCCCCCCCCCC=O | 17697 |
| 37 | IMPHY003390 | Hypophyllanthin | COC[C@@H]1Cc2cc(OC)c3c(c2[C@@H]([C@H]1COC)c1ccc(c(c1)OC)OC)OCO3 | 182140 |
| 38 | IMPHY003459 | Pimara-8(14),15-diene | C=C[C@]1(C)CC[C@H]2C(=C1)CC[C@@H]1[C@]2(C)CC | 440909 |
| 39 | IMPHY003484 | 2,5-Dimethylpyrazine | Cc1ncc(nc1)C | 31252 |
| 40 | IMPHY003485 | Myrcene | C=CC(=C)CCC=C(C)C | 31253 |
| 41 | IMPHY003525 | Nonanal | CCCCCCCCC=O | 31289 |
| 42 | IMPHY003689 | cis-3-Hexenal | CC/C=CCC=O | 643941 |
| 43 | IMPHY003982 | gamma-Terpinene | CC1=CCC(=CC1)C(C)C | 7461 |
| 44 | IMPHY004388 | Kaempferol | Oc1ccc(cc1)c1oc2cc(O)cc(c2c(=O)c1O)O | 5280863 |
| 45 | IMPHY004619 | Quercetin | Oc1cc(O)c2c(c1)oc(c(c2=O)O)c1ccc(c(c1)O)O | 5280343 |
| 46 | IMPHY005345 | 1-Octen-3-OL | CCCCCC(C=C)O | 18827 |
| 47 | IMPHY005364 | 2,2,6-Trimethylcyclohexanone | CC1CCCC(C1=O)(C)C | 17000 |
| 48 | IMPHY005537 | Ellagic acid | Oc1cc2c(=O)oc3c4c2c(c1O)oc(=O)c4cc(c3O)O | 5281855 |
| 49 | IMPHY005569 | alpha-Ionone | CC(=O)/C=C/C1C(=CCCC1(C)C)C | 5282108 |
| 50 | IMPHY006039 | 1,2,4-Trimethylbenzene | Cc1ccc(c(c1)C)C | 7247 |
| 51 | IMPHY006145 | p-Cymene | Cc1ccc(cc1)C(C)C | 7463 |
| 52 | IMPHY006322 | Safranal | O=CC1=C(C)C=CCC1(C)C | 61041 |
| 53 | IMPHY006485 | beta-Ionone | CC(=O)/C=C/C1=C(C)CCCC1(C)C | 638014 |
| 54 | IMPHY006550 | Thymol | Cc1ccc(c(c1)O)C(C)C | 6989 |
| 55 | IMPHY006619 | Salicylaldehyde | O=Cc1ccccc1O | 6998 |
| 56 | IMPHY006674 | Isopentyl benzoate | CC(CCOC(=O)c1ccccc1)C | 7193 |
| 57 | IMPHY006845 | 2-Methylnaphthalene | Cc1ccc2c(c1)cccc2 | 7055 |
| 58 | IMPHY006951 | Eicosane | CCCCCCCCCCCCCCCCCCCC | 8222 |
| 59 | IMPHY006971 | Methyl palmitate | CCCCCCCCCCCCCCCC(=O)OC | 8181 |
| 60 | IMPHY007182 | 1-Tetradecanol | CCCCCCCCCCCCCCO | 8209 |
| 61 | IMPHY007213 | Methyl stearate | CCCCCCCCCCCCCCCCCC(=O)OC | 8201 |
| 62 | IMPHY007276 | Nonan-1-ol | CCCCCCCCCO | 8914 |
| 63 | IMPHY007620 | 1-Octanol | CCCCCCCCO | 957 |
| 64 | IMPHY009355 | Tetracosane | CCCCCCCCCCCCCCCCCCCCCCCC | 12592 |
| 65 | IMPHY009368 | Heptadecane | CCCCCCCCCCCCCCCCC | 12398 |
| 66 | IMPHY009375 | Docosane | CCCCCCCCCCCCCCCCCCCCCC | 12405 |
| 67 | IMPHY009377 | Pentacosane | CCCCCCCCCCCCCCCCCCCCCCCCC | 12406 |
| 68 | IMPHY009382 | Heneicosane | CCCCCCCCCCCCCCCCCCCCC | 12403 |
| 69 | IMPHY009389 | Pentadecane | CCCCCCCCCCCCCCC | 12391 |
| 70 | IMPHY009419 | Tridecane | CCCCCCCCCCCCC | 12388 |
| 71 | IMPHY009481 | Octacosane | CCCCCCCCCCCCCCCCCCCCCCCCCCCC | 12408 |
| 72 | IMPHY009482 | Nonacosane | CCCCCCCCCCCCCCCCCCCCCCCCCCCCC | 12409 |
| 73 | IMPHY009490 | Tricosane | CCCCCCCCCCCCCCCCCCCCCCC | 12534 |
| 74 | IMPHY009752 | beta-Cyclocitral | O=CC1=C(C)CCCC1(C)C | 9895 |
| 75 | IMPHY009774 | Benzothiazole | c1ccc2c(c1)scn2 | 7222 |
| 76 | IMPHY009853 | Naphthalene | c1ccc2c(c1)cccc2 | 931 |
| 77 | IMPHY009879 | Hexyl benzoate | CCCCCCOC(=O)c1ccccc1 | 23235 |
| 78 | IMPHY009946 | Benzaldehyde | O=Cc1ccccc1 | 240 |
| 79 | IMPHY010072 | Eucalyptol | CC12CCC(CC1)C(O2)(C)C | 2758 |
| 80 | IMPHY010179 | (-)-beta-Chamigrene | CC1=CC[C@@]2(CC1)C(=C)CCCC2(C)C | 442353 |
| 81 | IMPHY010538 | Farnesylacetone | C/C(=CCC/C(=C/CCC(=O)C)/C)/CCC=C(C)C | 1711945 |
| 82 | IMPHY010965 | Corilagin | O[C@@H]1[C@H]2COC(=O)c3cc(O)c(c(c3-c3c(C(=O)O[C@@H]1[C@H]([C@@H](O2)OC(=O)c1cc(O)c(c(c1)O)O)O)cc(O)c(c3O)O)O)O | 73568 |
| 83 | IMPHY011008 | 1-Heptadecene | CCCCCCCCCCCCCCCC=C | 23217 |
| 84 | IMPHY011473 | Geraniin | O=C(c1cc(O)c(c(c1)O)O)O[C@@H]1O[C@@H]2COC(=O)c3cc(O)c(c(c3-c3c(C(=O)O[C@@H]4[C@H]1OC(=O)c1cc(O)c(c5c1[C@@H]1C(=CC(=O)[C@](O5)(C1(O)O)O)C(=O)O[C@H]24)O)cc(O)c(c3O)O)O)O | 3001497 |
| 85 | IMPHY011588 | cis-3-Hexen-1-ol | OCC/C=CCC | 5281167 |
| 86 | IMPHY011659 | alpha-Muurolene | CC1=C[C@@H]2[C@H](CC1)C(=CC[C@H]2C(C)C)C | 12306047 |
| 87 | IMPHY011696 | cis-3-Hexenyl benzoate | CC/C=CCCOC(=O)c1ccccc1 | 5367706 |
| 88 | IMPHY012021 | Gallic acid | OC(=O)c1cc(O)c(c(c1)O)O | 370 |
| 89 | IMPHY012036 | Camphor | O=C1CC2C(C1(C)CC2)(C)C | 2537 |
| 90 | IMPHY012058 | Linalool | C=CC(CCC=C(C)C)(O)C | 6549 |
| 91 | IMPHY012160 | alpha-Terpineol | CC1=CCC(CC1)C(O)(C)C | 17100 |
| 92 | IMPHY012328 | 1,1,6-Trimethyl-1,2-dihydronaphthalene | Cc1ccc2c(c1)C=CCC2(C)C | 121677 |
| 93 | IMPHY012586 | (-)-alpha-Cadinol | CC1=CC2C(CC1)[C@@](C)(O)CC[C@@H]2C(C)C | 6431302 |
| 94 | IMPHY012654 | Nerol | OC/C=C(CCC=C(C)C)/C | 643820 |
| 95 | IMPHY012712 | Phytol | OC/C=C(/CCC[C@@H](CCC[C@@H](CCCC(C)C)C)C)C | 5280435 |
| 96 | IMPHY012920 | 2-Furanmethanol, 5-ethenyltetrahydro-alpha,alpha,5-trimethyl-, cis- | C=C[C@@]1(C)CC[C@H](O1)C(O)(C)C | 11116492 |
| 97 | IMPHY014396 | Quercetin-3-glucoside | OC[C@H]1O[C@@H](Oc2c(oc3c(c2=O)c(O)cc(c3)O)c2ccc(c(c2)O)[O-])[C@@H]([C@H]([C@@H]1O)O)O | 25203368 |
| 98 | IMPHY014824 | Astragalin | OC[C@H]1O[C@@H](Oc2c(oc3c(c2=O)c(O)cc(c3)O)c2ccc(cc2)O)[C@@H]([C@H]([C@@H]1O)O)O | 5282102 |
| 99 | IMPHY014831 | beta-Caryophyllene | C/C/1=CCCC(=C)[C@@H]2[C@@H](CC1)C(C2)(C)C | 5281515 |
| 100 | IMPHY014923 | Geraniol | OC/C=C(/CCC=C(C)C)C | 637566 |
| 101 | IMPHY014988 | Limonene | CC1=CCC(CC1)C(=C)C | 22311 |
| 102 | IMPHY014989 | trans-Linalool oxide | C=C[C@]1(C)CC[C@H](O1)C(O)(C)C | 6432254 |
| 103 | IMPHY015022 | Nerolidol | C=CC(CC/C=C(/CCC=C(C)C)C)(O)C | 5284507 |
| 104 | IMPHY015047 | Rutin | Oc1cc(O)c2c(c1)oc(c(c2=O)O[C@@H]1O[C@H](CO[C@@H]2O[C@@H](C)[C@@H]([C@H]([C@H]2O)O)O)[C@H]([C@@H]([C@H]1O)O)O)c1ccc(c(c1)O)O | 5280805 |
| 105 | IMPHY015054 | Quercitrin | Oc1cc(O)c2c(c1)oc(c(c2=O)O[C@@H]1O[C@@H](C)[C@@H]([C@H]([C@H]1O)O)O)c1ccc(c(c1)O)O | 5280459 |
| 106 | IMPHY015316 | Bovolide | CCCC/C=C1/OC(=O)C(=C1C)C | 6433214 |
| 107 | IMPHY016041 | Linalool oxide acetate (pyranoid) | C=C[C@@]1(C)CC[C@@H](C(O1)(C)C)OC(=O)C | 6427501 |
| 108 | IMPHY016138 | 1-Ethylnaphthalene | CCc1cccc2c1cccc2 | 14315 |
| 109 | IMPHY016146 | 1-Methylnaphthalene | Cc1cccc2c1cccc2 | 7002 |
| 110 | IMPHY016329 | 2,6-Diisopropylnaphthalene | CC(c1ccc2c(c1)ccc(c2)C(C)C)C | 32241 |
| 111 | IMPHY016888 | beta-Bazzanene | CC1=CC[C@@](CC1)(C)[C@]1(C)CCCC1=C | 12444487 |
| 112 | IMPHY000014 | Lintetralin | OC[C@H]1Cc2cc(OC)c(cc2[C@H]([C@@H]1COC)c1ccc2c(c1)OCO2)OC | 11361584 |
| 113 | IMPHY000162 | Phyltetralin | COC[C@H]1Cc2cc(OC)c(cc2[C@H]([C@@H]1COC)c1ccc(c(c1)OC)OC)OC | 11223782 |
| 114 | IMPHY000465 | Geraniinic acid | O[C@H]1[C@@H](O[C@H]2[C@H]([C@@H]1OC(=O)c1cc(O)c(c(c1-c1c(C(=O)OC2)cc(c(c1O)O)O)O)O)OC(=O)c1cc(O)c(c(c1c1c(cc(c(c1O)O)O)C(=O)O)O)O)OC(=O)c1cc(O)c(c(c1)O)O |  |
| 115 | IMPHY001099 | Furosin | OC[C@H]1O[C@@H](OC(=O)c2cc(O)c(c(c2)O)O)[C@H]2[C@H]([C@@H]1OC(=O)C1=CC(=O)C(C3([C@@H]1c1c(C(=O)O2)cc(c(c1O3)O)O)O)(O)O)O | 10416810 |
| 116 | IMPHY001932 | Epibubbialine | O=C1C=C2[C@]3(O1)C[C@H]([C@H](C2)N1[C@H]3CCC1)O | 11830997 |
| 117 | IMPHY003246 | Phyllanthin | COC[C@H]([C@H](Cc1ccc(c(c1)OC)OC)COC)Cc1ccc(c(c1)OC)OC | 358901 |
| 118 | IMPHY003390 | Hypophyllanthin | COC[C@@H]1Cc2cc(OC)c3c(c2[C@@H]([C@H]1COC)c1ccc(c(c1)OC)OC)OCO3 | 182140 |
| 119 | IMPHY003474 | 1,6-bis-O-galloyl-beta-D-glucose | O[C@@H]1[C@@H](COC(=O)c2cc(O)c(c(c2)O)O)O[C@H]([C@@H]([C@H]1O)O)OC(=O)c1cc(O)c(c(c1)O)O | 440221 |
| 120 | IMPHY005537 | Ellagic acid | Oc1cc2c(=O)oc3c4c2c(c1O)oc(=O)c4cc(c3O)O | 5281855 |
| 121 | IMPHY010965 | Corilagin | O[C@@H]1[C@H]2COC(=O)c3cc(O)c(c(c3-c3c(C(=O)O[C@@H]1[C@H]([C@@H](O2)OC(=O)c1cc(O)c(c(c1)O)O)O)cc(O)c(c3O)O)O)O | 73568 |
| 122 | IMPHY011473 | Geraniin | O=C(c1cc(O)c(c(c1)O)O)O[C@@H]1O[C@@H]2COC(=O)c3cc(O)c(c(c3-c3c(C(=O)O[C@@H]4[C@H]1OC(=O)c1cc(O)c(c5c1[C@@H]1C(=CC(=O)[C@](O5)(C1(O)O)O)C(=O)O[C@H]24)O)cc(O)c(c3O)O)O)O | 3001497 |
| 123 | IMPHY011735 | (+)-Gallocatechin | Oc1cc2O[C@H](c3cc(O)c(c(c3)O)O)[C@H](Cc2c(c1)O)O | 65084 |
|  |  |  |  |  |
| **PLANT NAME : *Phyllanthus emblica*** | | | | |
| **Serial No** | **IMPPAT Phytochemical Identifier** | **Phytochemical Name** | **SMILES** | **CID** |
| 1 | IMPHY000569 | Procyanidin | Oc1cc(O)c2c(c1)OC(C(C2)OC1(Oc2cc(O)cc(c2C(C1O)O)O)c1ccc(c(c1)O)O)c1ccc(c(c1)O)O | 107876 |
| 2 | IMPHY000622 | Proanthocyanidin | COc1c(O)cc(cc1O)C1Oc2c(C[C@H]1O)c(O)cc(c2[C@@H]1[C@@H](O)[C@H](Oc2c1c(O)cc(c2)O)c1ccc(cc1)O)O | 108065 |
| 3 | IMPHY011741 | Tannic acid | O=C(c1cc(O)c(c(c1)OC(=O)c1cc(O)c(c(c1)O)O)O)O[C@@H]1[C@@H](COC(=O)c2cc(O)c(c(c2)OC(=O)c2cc(O)c(c(c2)O)O)O)O[C@H]([C@@H]([C@H]1OC(=O)c1cc(O)c(c(c1)OC(=O)c1cc(O)c(c(c1)O)O)O)OC(=O)c1cc(O)c(c(c1)OC(=O)c1cc(O)c(c(c1)O)O)O)OC(=O)c1cc(O)c(c(c1)OC(=O)c1cc(O)c(c(c1)O)O)O | 16129778 |
| 4 | IMPHY011885 | Leucodelphidin | Oc1cc2O[C@H](c3cc(O)c(c(c3)O)O)[C@H]([C@H](c2c(c1)O)O)O | 440835 |
| 5 | IMPHY012473 | Lupeol | CC(=C)[C@@H]1CC[C@]2([C@H]1[C@H]1CC[C@H]3[C@@]([C@]1(C)CC2)(C)CC[C@@H]1[C@]3(C)CC[C@@H](C1(C)C)O)C | 259846 |
| 6 | IMPHY000103 | Pyrogallol | Oc1c(O)cccc1O | 1057 |
| 7 | IMPHY000783 | 1,3,6-tri-O-galloyl-beta-D-glucose | O[C@@H]1[C@@H](COC(=O)c2cc(O)c(c(c2)O)O)O[C@H]([C@@H]([C@H]1OC(=O)c1cc(O)c(c(c1)O)O)O)OC(=O)c1cc(O)c(c(c1)O)O | 452707 |
| 8 | IMPHY000846 | Riboflavin | OC[C@H]([C@H]([C@H](Cn1c2-c(nc3c1cc(C)c(c3)C)c(=O)[nH]c(=O)n2)O)O)O | 493570 |
| 9 | IMPHY001099 | Furosin | OC[C@H]1O[C@@H](OC(=O)c2cc(O)c(c(c2)O)O)[C@H]2[C@H]([C@@H]1OC(=O)C1=CC(=O)C(C3([C@@H]1c1c(C(=O)O2)cc(c(c1O3)O)O)O)(O)O)O | 10416810 |
| 10 | IMPHY003139 | Terchebin | O=C(c1cc(O)c(c(c1)O)O)OC[C@H]1O[C@@H](OC(=O)c2cc(O)c(c(c2)O)O)[C@H]2[C@H]([C@@H]1OC(=O)C1=CC(=O)C(C3([C@@H]1c1c(C(=O)O2)cc(c(c1O3)O)O)O)(O)O)OC(=O)c1cc(O)c(c(c1)O)O | 3084341 |
| 11 | IMPHY003265 | Phloroglucinol | Oc1cc(O)cc(c1)O | 359 |
| 12 | IMPHY003906 | trans-Zeatin | OC/C(=C/CNc1ncnc2c1[nH]cn2)/C | 449093 |
| 13 | IMPHY004079 | Catechol | Oc1ccccc1O | 289 |
| 14 | IMPHY004619 | Quercetin | Oc1cc(O)c2c(c1)oc(c(c2=O)O)c1ccc(c(c1)O)O | 5280343 |
| 15 | IMPHY004799 | Chebulagic acid | OC(=O)C[C@@H]1C(=O)O[C@@H]2[C@H]3COC(=O)c4cc(O)c(c(c4-c4c(C(=O)O[C@@H]2[C@@H](OC(=O)c2c5[C@H]1[C@H](O)C(=O)Oc5c(c(c2)O)O)[C@@H](O3)OC(=O)c1cc(O)c(c(c1)O)O)cc(O)c(c4O)O)O)O | 442674 |
| 16 | IMPHY005537 | Ellagic acid | Oc1cc2c(=O)oc3c4c2c(c1O)oc(=O)c4cc(c3O)O | 5281855 |
| 17 | IMPHY006139 | Methyl gallate | COC(=O)c1cc(O)c(c(c1)O)O | 7428 |
| 18 | IMPHY006362 | Ascorbic acid | OC[C@@H]([C@H]1OC(=O)C(=C1O)O)O | 54670067 |
| 19 | IMPHY008800 | Kzeyiyxacmutrm-uhfffaoysa- | OC1COC(=O)c2cc(O)c(c(c2-c2c(C(=O)OC1C(C(C(=O)O)OC(=O)c1cc(O)c(c(c1)O)O)OC(=O)c1cc(O)c(c(c1)O)O)cc(O)c(c2O)O)O)O | 44631480 |
| 20 | IMPHY009335 | Phyllantidine | O=C1C=C2[C@@]3(O1)C[C@@H](C=C2)ON1[C@H]3CCCC1 | 12314211 |
| 21 | IMPHY009606 | Ethyl gallate | CCOC(=O)c1cc(O)c(c(c1)O)O | 13250 |
| 22 | IMPHY010070 | beta-Glucogallin | OC[C@H]1O[C@@H](OC(=O)c2cc(O)c(c(c2)O)O)[C@@H]([C@H]([C@@H]1O)O)O | 124021 |
| 23 | IMPHY010404 | Galactaric acid | O[C@H]([C@H](C(=O)O)O)[C@H]([C@@H](C(=O)O)O)O | 3037582 |
| 24 | IMPHY010827 | Chebulinic acid | OC(=O)C[C@@H]1C(=O)O[C@@H]2[C@@H](COC(=O)c3cc(O)c(c(c3)O)O)O[C@H]([C@@H]([C@H]2OC(=O)c2cc(O)c(c(c2)O)O)OC(=O)c2c3[C@H]1[C@H](O)C(=O)Oc3c(c(c2)O)O)OC(=O)c1cc(O)c(c(c1)O)O | 72284 |
| 25 | IMPHY010965 | Corilagin | O[C@@H]1[C@H]2COC(=O)c3cc(O)c(c(c3-c3c(C(=O)O[C@@H]1[C@H]([C@@H](O2)OC(=O)c1cc(O)c(c(c1)O)O)O)cc(O)c(c3O)O)O)O | 73568 |
| 26 | IMPHY010998 | Chebulic acid | OC(=O)C[C@@H]([C@@H]1[C@H](OC(=O)c2c1c(O)c(c(c2)O)O)C(=O)O)C(=O)O | 71308174 |
| 27 | IMPHY011473 | Geraniin | O=C(c1cc(O)c(c(c1)O)O)O[C@@H]1O[C@@H]2COC(=O)c3cc(O)c(c(c3-c3c(C(=O)O[C@@H]4[C@H]1OC(=O)c1cc(O)c(c5c1[C@@H]1C(=CC(=O)[C@](O5)(C1(O)O)O)C(=O)O[C@H]24)O)cc(O)c(c3O)O)O)O | 3001497 |
| 28 | IMPHY011741 | Tannic acid | O=C(c1cc(O)c(c(c1)OC(=O)c1cc(O)c(c(c1)O)O)O)O[C@@H]1[C@@H](COC(=O)c2cc(O)c(c(c2)OC(=O)c2cc(O)c(c(c2)O)O)O)O[C@H]([C@@H]([C@H]1OC(=O)c1cc(O)c(c(c1)OC(=O)c1cc(O)c(c(c1)O)O)O)OC(=O)c1cc(O)c(c(c1)OC(=O)c1cc(O)c(c(c1)O)O)O)OC(=O)c1cc(O)c(c(c1)OC(=O)c1cc(O)c(c(c1)O)O)O | 16129778 |
| 29 | IMPHY012021 | Gallic acid | OC(=O)c1cc(O)c(c(c1)O)O | 370 |
| 30 | IMPHY013560 | Trigalloylglucose | OC[C@H]([C@H]([C@]([C@](C(=O)c1cc(O)c(c(c1)O)O)(C(=O)C(=O)c1cc(O)c(c(c1)O)O)O)(C(=O)c1cc(O)c(c(c1)O)O)O)O)O | 90116889 |
| 31 | IMPHY014049 | Tryptase | CC(=O)O.OC[C@@H](C(=O)N[C@H](C(=O)Nc1cc(C)c2c(c1)oc(=O)cc2)CCCN=C(N)N)NC(=O)[C@H](Cc1ccccc1)NC(=O)OC(C)(C)C | 5311489 |
| 32 | IMPHY000783 | 1,3,6-tri-O-galloyl-beta-D-glucose | O[C@@H]1[C@@H](COC(=O)c2cc(O)c(c(c2)O)O)O[C@H]([C@@H]([C@H]1OC(=O)c1cc(O)c(c(c1)O)O)O)OC(=O)c1cc(O)c(c(c1)O)O | 452707 |
| 33 | IMPHY003139 | Terchebin | O=C(c1cc(O)c(c(c1)O)O)OC[C@H]1O[C@@H](OC(=O)c2cc(O)c(c(c2)O)O)[C@H]2[C@H]([C@@H]1OC(=O)C1=CC(=O)C(C3([C@@H]1c1c(C(=O)O2)cc(c(c1O3)O)O)O)(O)O)OC(=O)c1cc(O)c(c(c1)O)O | 3084341 |
| 34 | IMPHY004388 | Kaempferol | Oc1ccc(cc1)c1oc2cc(O)cc(c2c(=O)c1O)O | 5280863 |
| 35 | IMPHY004799 | Chebulagic acid | OC(=O)C[C@@H]1C(=O)O[C@@H]2[C@H]3COC(=O)c4cc(O)c(c(c4-c4c(C(=O)O[C@@H]2[C@@H](OC(=O)c2c5[C@H]1[C@H](O)C(=O)Oc5c(c(c2)O)O)[C@@H](O3)OC(=O)c1cc(O)c(c(c1)O)O)cc(O)c(c4O)O)O)O | 442674 |
| 36 | IMPHY005537 | Ellagic acid | Oc1cc2c(=O)oc3c4c2c(c1O)oc(=O)c4cc(c3O)O | 5281855 |
| 37 | IMPHY006362 | Ascorbic acid | OC[C@@H]([C@H]1OC(=O)C(=C1O)O)O | 54670067 |
| 38 | IMPHY009335 | Phyllantidine | O=C1C=C2[C@@]3(O1)C[C@@H](C=C2)ON1[C@H]3CCCC1 | 12314211 |
| 39 | IMPHY010404 | Galactaric acid | O[C@H]([C@H](C(=O)O)O)[C@H]([C@@H](C(=O)O)O)O | 3037582 |
| 40 | IMPHY010827 | Chebulinic acid | OC(=O)C[C@@H]1C(=O)O[C@@H]2[C@@H](COC(=O)c3cc(O)c(c(c3)O)O)O[C@H]([C@@H]([C@H]2OC(=O)c2cc(O)c(c(c2)O)O)OC(=O)c2c3[C@H]1[C@H](O)C(=O)Oc3c(c(c2)O)O)OC(=O)c1cc(O)c(c(c1)O)O | 72284 |
| 41 | IMPHY010965 | Corilagin | O[C@@H]1[C@H]2COC(=O)c3cc(O)c(c(c3-c3c(C(=O)O[C@@H]1[C@H]([C@@H](O2)OC(=O)c1cc(O)c(c(c1)O)O)O)cc(O)c(c3O)O)O)O | 73568 |
| 42 | IMPHY010998 | Chebulic acid | OC(=O)C[C@@H]([C@@H]1[C@H](OC(=O)c2c1c(O)c(c(c2)O)O)C(=O)O)C(=O)O | 71308174 |
| 43 | IMPHY012021 | Gallic acid | OC(=O)c1cc(O)c(c(c1)O)O | 370 |
| 44 | IMPHY012473 | Lupeol | CC(=C)[C@@H]1CC[C@]2([C@H]1[C@H]1CC[C@H]3[C@@]([C@]1(C)CC2)(C)CC[C@@H]1[C@]3(C)CC[C@@H](C1(C)C)O)C | 259846 |
| 45 | IMPHY014824 | Astragalin | OC[C@H]1O[C@@H](Oc2c(oc3c(c2=O)c(O)cc(c3)O)c2ccc(cc2)O)[C@@H]([C@H]([C@@H]1O)O)O | 5282102 |
| 46 | IMPHY014836 | beta-Sitosterol | CC[C@@H](C(C)C)CC[C@H]([C@H]1CC[C@@H]2[C@]1(C)CC[C@H]1[C@H]2CC=C2[C@]1(C)CC[C@@H](C2)O)C | 222284 |
| 47 | IMPHY001221 | Oleanolic aldehyde | O=C[C@]12CC[C@@]3(C(=CC[C@H]4[C@@]3(C)CC[C@@H]3[C@]4(C)CC[C@@H](C3(C)C)O)[C@@H]2CC(CC1)(C)C)C | 10321055 |
| 48 | IMPHY005537 | Ellagic acid | Oc1cc2c(=O)oc3c4c2c(c1O)oc(=O)c4cc(c3O)O | 5281855 |
| 49 | IMPHY011670 | Epigallocatechin gallate | Oc1cc(O)c2c(c1)O[C@@H]([C@@H](C2)OC(=O)c1cc(O)c(c(c1)O)O)c1cc(O)c(c(c1)O)O | 65064 |
| 50 | IMPHY011826 | Oleanolic acid | O[C@H]1CC[C@]2([C@H](C1(C)C)CC[C@@]1([C@@H]2CC=C2[C@@]1(C)CC[C@@]1([C@H]2CC(C)(C)CC1)C(=O)O)C)C | 10494 |
| 51 | IMPHY012473 | Lupeol | CC(=C)[C@@H]1CC[C@]2([C@H]1[C@H]1CC[C@H]3[C@@]([C@]1(C)CC2)(C)CC[C@@H]1[C@]3(C)CC[C@@H](C1(C)C)O)C | 259846 |
| 52 | IMPHY000060 | Myristic acid | CCCCCCCCCCCCCC(=O)O | 11005 |
| 53 | IMPHY000846 | Riboflavin | OC[C@H]([C@H]([C@H](Cn1c2-c(nc3c1cc(C)c(c3)C)c(=O)[nH]c(=O)n2)O)O)O | 493570 |
| 54 | IMPHY004631 | Stearic acid | CCCCCCCCCCCCCCCCCC(=O)O | 5281 |
| 55 | IMPHY006362 | Ascorbic acid | OC[C@@H]([C@H]1OC(=O)C(=C1O)O)O | 54670067 |
| 56 | IMPHY007327 | Palmitic acid | CCCCCCCCCCCCCCCC(=O)O | 985 |
| 57 | IMPHY007357 | Nicotinic acid | OC(=O)c1cccnc1 | 938 |
| 58 | IMPHY010404 | Galactaric acid | O[C@H]([C@H](C(=O)O)O)[C@H]([C@@H](C(=O)O)O)O | 3037582 |
| 59 | IMPHY011609 | alpha-Carotene | C/C(=CC=CC=C(C=CC=C(C=CC1=C(C)CCCC1(C)C)/C)/C)/C=C/C=C(/C=C/[C@H]1C(=CCCC1(C)C)C)C | 6419725 |
| 60 | IMPHY011797 | Oleic acid | CCCCCCCC/C=CCCCCCCCC(=O)O | 445639 |
| 61 | IMPHY012723 | Linolenic acid | CC/C=CC/C=CC/C=CCCCCCCCC(=O)O | 5280934 |
| 62 | IMPHY014836 | beta-Sitosterol | CC[C@@H](C(C)C)CC[C@H]([C@H]1CC[C@@H]2[C@]1(C)CC[C@H]1[C@H]2CC=C2[C@]1(C)CC[C@@H](C2)O)C | 222284 |
| 63 | IMPHY014893 | D-Glucose | OC[C@H]1OC(O)[C@@H]([C@H]([C@@H]1O)O)O | 5793 |
| 64 | IMPHY014919 | D-Galacturonic Acid | OC1O[C@H](C(=O)O)[C@@H]([C@@H]([C@H]1O)O)O | 439215 |
| 65 | IMPHY014990 | Linoleic acid | CCCCC/C=CC/C=CCCCCCCCC(=O)O | 5280450 |
| 66 | IMPHY011264 | Eriodictyol-7-O-glucoside | OC[C@H]1O[C@@H](Oc2cc3O[C@@H](CC(=O)c3c(c2)O)c2ccc(c(c2)O)O)[C@@H]([C@H]([C@@H]1O)O)O | 13254473 |
| 67 | IMPHY012473 | Lupeol | CC(=C)[C@@H]1CC[C@]2([C@H]1[C@H]1CC[C@H]3[C@@]([C@]1(C)CC2)(C)CC[C@@H]1[C@]3(C)CC[C@@H](C1(C)C)O)C | 259846 |
| 68 | IMPHY014836 | beta-Sitosterol | CC[C@@H](C(C)C)CC[C@H]([C@H]1CC[C@@H]2[C@]1(C)CC[C@H]1[C@H]2CC=C2[C@]1(C)CC[C@@H](C2)O)C | 222284 |
| 69 | IMPHY000103 | Pyrogallol | Oc1c(O)cccc1O | 1057 |
| 70 | IMPHY000569 | Procyanidin | Oc1cc(O)c2c(c1)OC(C(C2)OC1(Oc2cc(O)cc(c2C(C1O)O)O)c1ccc(c(c1)O)O)c1ccc(c(c1)O)O | 107876 |
| 71 | IMPHY000622 | Proanthocyanidin | COc1c(O)cc(cc1O)C1Oc2c(C[C@H]1O)c(O)cc(c2[C@@H]1[C@@H](O)[C@H](Oc2c1c(O)cc(c2)O)c1ccc(cc1)O)O | 108065 |
| 72 | IMPHY002390 | 3-6-Di-o-galloyl-glucose | O[C@@H]1[C@@H](COC(=O)c2cc(O)c(c(c2)O)O)OC([C@@H]([C@H]1OC(=O)c1cc(O)c(c(c1)O)O)O)O |  |
| 73 | IMPHY004799 | Chebulagic acid | OC(=O)C[C@@H]1C(=O)O[C@@H]2[C@H]3COC(=O)c4cc(O)c(c(c4-c4c(C(=O)O[C@@H]2[C@@H](OC(=O)c2c5[C@H]1[C@H](O)C(=O)Oc5c(c(c2)O)O)[C@@H](O3)OC(=O)c1cc(O)c(c(c1)O)O)cc(O)c(c4O)O)O)O | 442674 |
| 74 | IMPHY005054 | 2-Methoxycarbonyl-6-oxo-3-[2,3,4-trihydroxy-6-[4-hydroxy-6-(hydroxymethyl)-2-(3,4,5-trihydroxybenzoyl)oxyoxan-3-yl]oxycarbonylphenyl]oxane-4-carboxylic acid | OCC1CC(O)C(C(O1)OC(=O)c1cc(O)c(c(c1)O)O)OC(=O)c1cc(O)c(c(c1C1C(CC(=O)OC1C(=O)OC)C(=O)O)O)O | 5318051 |
| 75 | IMPHY005537 | Ellagic acid | Oc1cc2c(=O)oc3c4c2c(c1O)oc(=O)c4cc(c3O)O | 5281855 |
| 76 | IMPHY006362 | Ascorbic acid | OC[C@@H]([C@H]1OC(=O)C(=C1O)O)O | 54670067 |
| 77 | IMPHY007357 | Nicotinic acid | OC(=O)c1cccnc1 | 938 |
| 78 | IMPHY008252 | Inositol | OC1C(O)C(O)C(C(C1O)O)O | 892 |
| 79 | IMPHY010965 | Corilagin | O[C@@H]1[C@H]2COC(=O)c3cc(O)c(c(c3-c3c(C(=O)O[C@@H]1[C@H]([C@@H](O2)OC(=O)c1cc(O)c(c(c1)O)O)O)cc(O)c(c3O)O)O)O | 73568 |
| 80 | IMPHY011473 | Geraniin | O=C(c1cc(O)c(c(c1)O)O)O[C@@H]1O[C@@H]2COC(=O)c3cc(O)c(c(c3-c3c(C(=O)O[C@@H]4[C@H]1OC(=O)c1cc(O)c(c5c1[C@@H]1C(=CC(=O)[C@](O5)(C1(O)O)O)C(=O)O[C@H]24)O)cc(O)c(c3O)O)O)O | 3001497 |
| 81 | IMPHY011707 | beta-Carotene | C/C(=CC=CC=C(C=CC=C(C=CC1=C(C)CCCC1(C)C)/C)/C)/C=C/C=C(/C=C/C1=C(C)CCCC1(C)C)C | 5280489 |
| 82 | IMPHY011885 | Leucodelphidin | Oc1cc2O[C@H](c3cc(O)c(c(c3)O)O)[C@H]([C@H](c2c(c1)O)O)O | 440835 |
| 83 | IMPHY012021 | Gallic acid | OC(=O)c1cc(O)c(c(c1)O)O | 370 |
| 84 | IMPHY012473 | Lupeol | CC(=C)[C@@H]1CC[C@]2([C@H]1[C@H]1CC[C@H]3[C@@]([C@]1(C)CC2)(C)CC[C@@H]1[C@]3(C)CC[C@@H](C1(C)C)O)C | 259846 |
| 85 | IMPHY014824 | Astragalin | OC[C@H]1O[C@@H](Oc2c(oc3c(c2=O)c(O)cc(c3)O)c2ccc(cc2)O)[C@@H]([C@H]([C@@H]1O)O)O | 5282102 |
| 86 | IMPHY014836 | beta-Sitosterol | CC[C@@H](C(C)C)CC[C@H]([C@H]1CC[C@@H]2[C@]1(C)CC[C@H]1[C@H]2CC=C2[C@]1(C)CC[C@@H](C2)O)C | 222284 |
| 87 | IMPHY014919 | D-Galacturonic Acid | OC1O[C@H](C(=O)O)[C@@H]([C@@H]([C@H]1O)O)O | 439215 |
| 88 | IMPHY014990 | Linoleic acid | CCCCC/C=CC/C=CCCCCCCCC(=O)O | 5280450 |
|  |  |  |  |  |
| **PLANT NAME : *Phyllanthus niruri*** | | | | |
| **Serial No** | **IMPPAT Phytochemical Identifier** | **Phytochemical Name** | **SMILES** | **CID** |
| 1 | IMPHY010965 | Corilagin | O[C@@H]1[C@H]2COC(=O)c3cc(O)c(c(c3-c3c(C(=O)O[C@@H]1[C@H]([C@@H](O2)OC(=O)c1cc(O)c(c(c1)O)O)O)cc(O)c(c3O)O)O)O | 73568 |
| 2 | IMPHY003390 | Hypophyllanthin | COC[C@@H]1Cc2cc(OC)c3c(c2[C@@H]([C@H]1COC)c1ccc(c(c1)OC)OC)OCO3 | 182140 |
| 3 | IMPHY003246 | Phyllanthin | COC[C@H]([C@H](Cc1ccc(c(c1)OC)OC)COC)Cc1ccc(c(c1)OC)OC | 358901 |
| 4 | IMPHY003390 | Hypophyllanthin | COC[C@@H]1Cc2cc(OC)c3c(c2[C@@H]([C@H]1COC)c1ccc(c(c1)OC)OC)OCO3 | 182140 |
|  |  |  |  |  |
| **PLANT NAME : *Phyllanthus urinaria*** | | | | |
| **Serial No** | **IMPPAT Phytochemical Identifier** | **Phytochemical Name** | **SMILES** | **CID** |
| 1 | IMPHY004619 | Quercetin | Oc1cc(O)c2c(c1)oc(c(c2=O)O)c1ccc(c(c1)O)O | 5280343 |
| 2 | IMPHY006362 | Ascorbic acid | OC[C@@H]([C@H]1OC(=O)C(=C1O)O)O | 54670067 |
| 3 | IMPHY012721 | Isoquercitrin | OC[C@H]1O[C@@H](Oc2c(oc3c(c2=O)c(O)cc(c3)O)c2ccc(c(c2)O)O)[C@@H]([C@H]([C@@H]1O)O)O | 5280804 |
| 4 | IMPHY014824 | Astragalin | OC[C@H]1O[C@@H](Oc2c(oc3c(c2=O)c(O)cc(c3)O)c2ccc(cc2)O)[C@@H]([C@H]([C@@H]1O)O)O | 5282102 |
| 5 | IMPHY015047 | Rutin | Oc1cc(O)c2c(c1)oc(c(c2=O)O[C@@H]1O[C@H](CO[C@@H]2O[C@@H](C)[C@@H]([C@H]([C@H]2O)O)O)[C@H]([C@@H]([C@H]1O)O)O)c1ccc(c(c1)O)O | 5280805 |
| 6 | IMPHY015054 | Quercitrin | Oc1cc(O)c2c(c1)oc(c(c2=O)O[C@@H]1O[C@@H](C)[C@@H]([C@H]([C@H]1O)O)O)c1ccc(c(c1)O)O | 5280459 |
| 7 | IMPHY004619 | Quercetin | Oc1cc(O)c2c(c1)oc(c(c2=O)O)c1ccc(c(c1)O)O | 5280343 |
| 8 | IMPHY012721 | Isoquercitrin | OC[C@H]1O[C@@H](Oc2c(oc3c(c2=O)c(O)cc(c3)O)c2ccc(c(c2)O)O)[C@@H]([C@H]([C@@H]1O)O)O | 5280804 |
| 9 | IMPHY014824 | Astragalin | OC[C@H]1O[C@@H](Oc2c(oc3c(c2=O)c(O)cc(c3)O)c2ccc(cc2)O)[C@@H]([C@H]([C@@H]1O)O)O | 5282102 |
| 10 | IMPHY015047 | Rutin | Oc1cc(O)c2c(c1)oc(c(c2=O)O[C@@H]1O[C@H](CO[C@@H]2O[C@@H](C)[C@@H]([C@H]([C@H]2O)O)O)[C@H]([C@@H]([C@H]1O)O)O)c1ccc(c(c1)O)O | 5280805 |
| 11 | IMPHY015054 | Quercitrin | Oc1cc(O)c2c(c1)oc(c(c2=O)O[C@@H]1O[C@@H](C)[C@@H]([C@H]([C@H]1O)O)O)c1ccc(c(c1)O)O | 5280459 |
| 12 | IMPHY003390 | Hypophyllanthin | COC[C@@H]1Cc2cc(OC)c3c(c2[C@@H]([C@H]1COC)c1ccc(c(c1)OC)OC)OCO3 | 182140 |
| 13 | IMPHY010965 | Corilagin | O[C@@H]1[C@H]2COC(=O)c3cc(O)c(c(c3-c3c(C(=O)O[C@@H]1[C@H]([C@@H](O2)OC(=O)c1cc(O)c(c(c1)O)O)O)cc(O)c(c3O)O)O)O | 73568 |
| 14 | IMPHY001915 | Octadecane | CCCCCCCCCCCCCCCCCC | 11635 |
| 15 | IMPHY004388 | Kaempferol | Oc1ccc(cc1)c1oc2cc(O)cc(c2c(=O)c1O)O | 5280863 |
| 16 | IMPHY004619 | Quercetin | Oc1cc(O)c2c(c1)oc(c(c2=O)O)c1ccc(c(c1)O)O | 5280343 |
| 17 | IMPHY005537 | Ellagic acid | Oc1cc2c(=O)oc3c4c2c(c1O)oc(=O)c4cc(c3O)O | 5281855 |
| 18 | IMPHY011619 | alpha-Amyrin | C[C@@H]1CC[C@]2([C@@H]([C@H]1C)C1=CC[C@H]3[C@@]([C@@]1(CC2)C)(C)CC[C@@H]1[C@]3(C)CC[C@@H](C1(C)C)O)C | 73170 |
| 19 | IMPHY011741 | Tannic acid | O=C(c1cc(O)c(c(c1)OC(=O)c1cc(O)c(c(c1)O)O)O)O[C@@H]1[C@@H](COC(=O)c2cc(O)c(c(c2)OC(=O)c2cc(O)c(c(c2)O)O)O)O[C@H]([C@@H]([C@H]1OC(=O)c1cc(O)c(c(c1)OC(=O)c1cc(O)c(c(c1)O)O)O)OC(=O)c1cc(O)c(c(c1)OC(=O)c1cc(O)c(c(c1)O)O)O)OC(=O)c1cc(O)c(c(c1)OC(=O)c1cc(O)c(c(c1)O)O)O | 16129778 |
| 20 | IMPHY012021 | Gallic acid | OC(=O)c1cc(O)c(c(c1)O)O | 370 |
| 21 | IMPHY012473 | Lupeol | CC(=C)[C@@H]1CC[C@]2([C@H]1[C@H]1CC[C@H]3[C@@]([C@]1(C)CC2)(C)CC[C@@H]1[C@]3(C)CC[C@@H](C1(C)C)O)C | 259846 |
| 22 | IMPHY012721 | Isoquercitrin | OC[C@H]1O[C@@H](Oc2c(oc3c(c2=O)c(O)cc(c3)O)c2ccc(c(c2)O)O)[C@@H]([C@H]([C@@H]1O)O)O | 5280804 |
| 23 | IMPHY014824 | Astragalin | OC[C@H]1O[C@@H](Oc2c(oc3c(c2=O)c(O)cc(c3)O)c2ccc(cc2)O)[C@@H]([C@H]([C@@H]1O)O)O | 5282102 |
| 24 | IMPHY014836 | beta-Sitosterol | CC[C@@H](C(C)C)CC[C@H]([C@H]1CC[C@@H]2[C@]1(C)CC[C@H]1[C@H]2CC=C2[C@]1(C)CC[C@@H](C2)O)C | 222284 |
| 25 | IMPHY014838 | Daucosterol | CC[C@@H](C(C)C)CC[C@H]([C@H]1CC[C@@H]2[C@]1(C)CC[C@H]1[C@H]2CC=C2[C@]1(C)CC[C@@H](C2)O[C@@H]1O[C@H](CO)[C@H]([C@@H]([C@H]1O)O)O)C | 5742590 |
| 26 | IMPHY015047 | Rutin | Oc1cc(O)c2c(c1)oc(c(c2=O)O[C@@H]1O[C@H](CO[C@@H]2O[C@@H](C)[C@@H]([C@H]([C@H]2O)O)O)[C@H]([C@@H]([C@H]1O)O)O)c1ccc(c(c1)O)O | 5280805 |
| 27 | IMPHY015054 | Quercitrin | Oc1cc(O)c2c(c1)oc(c(c2=O)O[C@@H]1O[C@@H](C)[C@@H]([C@H]([C@H]1O)O)O)c1ccc(c(c1)O)O | 5280459 |
|  |  |  |  |  |
| **PLANT NAME : *Piper betle*** | | | | |
| **Serial No** | **IMPPAT Phytochemical Identifier** | **Phytochemical Name** | **SMILES** | **CID** |
| 1 | IMPHY000099 | Myrtenol | OCC1=CCC2CC1C2(C)C | 10582 |
| 2 | IMPHY000846 | Riboflavin | OC[C@H]([C@H]([C@H](Cn1c2-c(nc3c1cc(C)c(c3)C)c(=O)[nH]c(=O)n2)O)O)O | 493570 |
| 3 | IMPHY001144 | Dillapiol | C=CCc1cc2OCOc2c(c1OC)OC | 10231 |
| 4 | IMPHY001308 | Retinol | OC/C=C(/C=C/C=C(/C=C/C1=C(C)CCCC1(C)C)C)C | 445354 |
| 5 | IMPHY003129 | Allylpyrocatechol | Oc1c(CC=C)cccc1O | 292101 |
| 6 | IMPHY003270 | Guaiazulene | CC(c1ccc(c2-c(c1)c(C)cc2)C)C | 3515 |
| 7 | IMPHY003485 | Myrcene | C=CC(=C)CCC=C(C)C | 31253 |
| 8 | IMPHY003536 | Eugenol | C=CCc1ccc(c(c1)OC)O | 3314 |
| 9 | IMPHY003616 | Bicyclogermacrene | C/C/1=CCC/C(=C/[C@H]2[C@@H](CC1)C2(C)C)/C | 13894537 |
| 10 | IMPHY003956 | (+)-gamma-Gurjunene | C[C@@H]1CC[C@H]2C1=C[C@@H](CC[C@H]2C)C(=C)C | 15560285 |
| 11 | IMPHY003962 | beta-Guaiene | CC(=C1CC[C@@H](C2=C(C1)[C@@H](C)CC2)C)C | 15560252 |
| 12 | IMPHY003977 | (-)-beta-Bourbonene | CC([C@@H]1CC[C@@]2([C@H]1[C@H]1C(=C)CC[C@@H]21)C)C | 62566 |
| 13 | IMPHY003982 | gamma-Terpinene | CC1=CCC(=CC1)C(C)C | 7461 |
| 14 | IMPHY004135 | Cyclohexene, 6-ethenyl-6-methyl-1-(1-methylethyl)-3-(1-methylethylidene)-, (S | C=C[C@@]1(C)CCC(=C(C)C)C=C1C(C)C | 11019992 |
| 15 | IMPHY004549 | Safrole | C=CCc1ccc2c(c1)OCO2 | 5144 |
| 16 | IMPHY004631 | Stearic acid | CCCCCCCCCCCCCCCCCC(=O)O | 5281 |
| 17 | IMPHY005374 | 4-Allyl-1,2-diacetoxybenzene | C=CCc1ccc(c(c1)OC(=O)C)OC(=O)C | 166872 |
| 18 | IMPHY005541 | 1,4,4-Trimethyl-8-methylene-1,5-cycloundecadiene | C=C1CCC/C(=CCC(/C=CC1)(C)C)/C | 21159064 |
| 19 | IMPHY006145 | p-Cymene | Cc1ccc(cc1)C(C)C | 7463 |
| 20 | IMPHY006297 | Chavibetol | C=CCc1ccc(c(c1)O)OC | 596375 |
| 21 | IMPHY006325 | Myrtenal | O=CC1=CCC2CC1C2(C)C | 61130 |
| 22 | IMPHY006362 | Ascorbic acid | OC[C@@H]([C@H]1OC(=O)C(=C1O)O)O | 54670067 |
| 23 | IMPHY006417 | 2,6-Dimethyl-2,4,6-octatriene | C/C=C(/C=C/C=C(C)C)C | 5368821 |
| 24 | IMPHY006558 | 1-Triacontanol | CCCCCCCCCCCCCCCCCCCCCCCCCCCCCCO | 68972 |
| 25 | IMPHY006593 | 4-Allylbenzene-1,2-diol | C=CCc1ccc(c(c1)O)O | 70775 |
| 26 | IMPHY006696 | Methyleugenol | C=CCc1ccc(c(c1)OC)OC | 7127 |
| 27 | IMPHY006709 | Acetyleugenol | C=CCc1ccc(c(c1)OC)OC(=O)C | 7136 |
| 28 | IMPHY006944 | Estragole | COc1ccc(cc1)CC=C | 8815 |
| 29 | IMPHY006946 | (1aR,4S,4aS,7R,7aS,7bR)-1,1,4,7-tetramethyl-2,3,4,5,6,7,7a,7b-octahydro-1aH-cyclopropa[h]azulen-4a-ol | C[C@@H]1CC[C@]2([C@@H]1[C@H]1[C@H](C1(C)C)CC[C@@H]2C)O | 9794494 |
| 30 | IMPHY006970 | Decanal | CCCCCCCCCC=O | 8175 |
| 31 | IMPHY007317 | 4-Allylphenol | C=CCc1ccc(cc1)O | 68148 |
| 32 | IMPHY007357 | Nicotinic acid | OC(=O)c1cccnc1 | 938 |
| 33 | IMPHY007376 | beta-Cubebene | CC([C@@H]1CC[C@H]([C@]23[C@H]1[C@H]2C(=C)CC3)C)C | 93081 |
| 34 | IMPHY007450 | Oxalic acid | OC(=O)C(=O)O | 971 |
| 35 | IMPHY007520 | Viridiflorene | C[C@@H]1CCC2=C(C)CC[C@@H]3[C@H]([C@H]12)C3(C)C | 10910653 |
| 36 | IMPHY007615 | Allylpyrocatechol diacetate | C=CCc1cccc(c1OC(=O)C)OC(=O)C | 46700759 |
| 37 | IMPHY007840 | Spathulenol | C=C1CC[C@@H]2[C@H]([C@H]3[C@H]1CC[C@]3(C)O)C2(C)C | 92231 |
| 38 | IMPHY008936 | alpha-Guaiene | CC(=C)[C@@H]1CC[C@@H](C2=C(C1)[C@@H](C)CC2)C | 5317844 |
| 39 | IMPHY009708 | 4-Allylphenyl acetate | C=CCc1ccc(cc1)OC(=O)C | 523825 |
| 40 | IMPHY009827 | Artemisiatriene | C=CC(/C=C/C(=C)C)(C)C | 5320377 |
| 41 | IMPHY010072 | Eucalyptol | CC12CCC(CC1)C(O2)(C)C | 2758 |
| 42 | IMPHY010080 | beta-Elemene | C=C[C@]1(C)CC[C@H](C[C@H]1C(=C)C)C(=C)C | 6918391 |
| 43 | IMPHY010603 | beta-Cadinene | CC1=CC[C@@H]2[C@@H](C1)[C@@H](CC=C2C)C(C)C | 10657 |
| 44 | IMPHY011392 | 3-Carene | CC1=CCC2C(C1)C2(C)C | 26049 |
| 45 | IMPHY011396 | 4-Carvomenthenol | CC1=CCC(CC1)(O)C(C)C | 11230 |
| 46 | IMPHY011521 | 2-Undecanone | CCCCCCCCCC(=O)C | 8163 |
| 47 | IMPHY011542 | beta-Eudesmol | C=C1CCC[C@]2([C@H]1C[C@@H](CC2)C(O)(C)C)C | 91457 |
| 48 | IMPHY011558 | Apiole | C=CCc1cc(OC)c2c(c1OC)OCO2 | 10659 |
| 49 | IMPHY011579 | Eremophilene | CC(=C)[C@@H]1CCC2=CCC[C@@H]([C@]2(C1)C)C | 12309744 |
| 50 | IMPHY011581 | alpha-Selinene | CC1=CCC[C@]2([C@H]1C[C@@H](CC2)C(=C)C)C | 10856614 |
| 51 | IMPHY011586 | (S,1Z,6Z)-8-Isopropyl-1-methyl-5-methylenecyclodeca-1,6-diene | C/C/1=C/CCC(=C)/C=C[C@@H](CC1)C(C)C | 91723653 |
| 52 | IMPHY011599 | Terpinolene | CC1=CCC(=C(C)C)CC1 | 11463 |
| 53 | IMPHY011640 | Isoeugenol | C/C=C/c1ccc(c(c1)OC)O | 853433 |
| 54 | IMPHY011643 | alpha-Terpinene | CC1=CC=C(CC1)C(C)C | 7462 |
| 55 | IMPHY011659 | alpha-Muurolene | CC1=C[C@@H]2[C@H](CC1)C(=CC[C@H]2C(C)C)C | 12306047 |
| 56 | IMPHY011660 | (+)-alpha-Cadinene | CC1=C[C@@H]2[C@@H](CC1)C(=CC[C@H]2C(C)C)C | 12306048 |
| 57 | IMPHY011667 | alpha-Gurjunene | C[C@@H]1CC[C@@H]2[C@H](C3=C(CC[C@H]13)C)C2(C)C | 15560276 |
| 58 | IMPHY011707 | beta-Carotene | C/C(=CC=CC=C(C=CC=C(C=CC1=C(C)CCCC1(C)C)/C)/C)/C=C/C=C(/C=C/C1=C(C)CCCC1(C)C)C | 5280489 |
| 59 | IMPHY011761 | Humulene | C/C/1=CCC(C)(C)/C=C/C/C(=C/CC1)/C | 5281520 |
| 60 | IMPHY011792 | gamma-Muurolene | CC1=C[C@@H]2[C@H](CC1)C(=C)CC[C@H]2C(C)C | 12313020 |
| 61 | IMPHY011793 | (+)-gamma-Cadinene | CC1=C[C@@H]2[C@@H](CC1)C(=C)CC[C@H]2C(C)C | 6432404 |
| 62 | IMPHY011896 | Valencene | CC(=C)[C@@H]1CCC2=CCC[C@H]([C@@]2(C1)C)C | 9855795 |
| 63 | IMPHY011957 | (+)-delta-Cadinene | CC1=C[C@@H]2C(=C(C)CC[C@H]2C(C)C)CC1 | 441005 |
| 64 | IMPHY011965 | (+)-beta-Phellandrene | CC([C@@H]1CCC(=C)C=C1)C | 442484 |
| 65 | IMPHY012036 | Camphor | O=C1CC2C(C1(C)CC2)(C)C | 2537 |
| 66 | IMPHY012058 | Linalool | C=CC(CCC=C(C)C)(O)C | 6549 |
| 67 | IMPHY012061 | alpha-Pinene | CC1=CCC2CC1C2(C)C | 6654 |
| 68 | IMPHY012104 | Citronellol | OCCC(CCC=C(C)C)C | 8842 |
| 69 | IMPHY012147 | beta-Pinene | C=C1CCC2CC1C2(C)C | 14896 |
| 70 | IMPHY012160 | alpha-Terpineol | CC1=CCC(CC1)C(O)(C)C | 17100 |
| 71 | IMPHY012261 | alpha-Bergamotene | CC(=CCCC1(C)C2CC=C(C1C2)C)C | 86608 |
| 72 | IMPHY012265 | (1r,3s,5r)-6,6-Dimethyl-2-methylidenebicyclo[3.1.1]heptan-3-ol | C=C1[C@@H](O)C[C@H]2C[C@@H]1C2(C)C | 88302 |
| 73 | IMPHY012305 | beta-Patchoulene | CC1CCC2=C1CC1CCC2(C1(C)C)C | 101731 |
| 74 | IMPHY012464 | Clionasterol | CC[C@H](C(C)C)CC[C@H]([C@H]1CC[C@@H]2[C@]1(C)CC[C@H]1[C@H]2CC=C2[C@]1(C)CC[C@@H](C2)O)C | 457801 |
| 75 | IMPHY012712 | Phytol | OC/C=C(/CCC[C@@H](CCC[C@@H](CCCC(C)C)C)C)C | 5280435 |
| 76 | IMPHY012739 | (Z)-beta-Ocimene | C=C/C(=CCC=C(C)C)/C | 5320250 |
| 77 | IMPHY012921 | gamma-Elemene | C=C[C@]1(C)CCC(=C(C)C)C[C@H]1C(=C)C | 6432312 |
| 78 | IMPHY013093 | delta-Elemene | C=C[C@@]1(C)CCC(=C[C@@H]1C(=C)C)C(C)C | 12309449 |
| 79 | IMPHY013133 | (Z)-p-Menth-2-en-1-ol | CC([C@@H]1CC[C@](C=C1)(C)O)C | 13918681 |
| 80 | IMPHY013835 | (+)-Fenchone | O=C1[C@@]2(C)CC[C@@H](C1(C)C)C2 | 1201521 |
| 81 | IMPHY014708 | beta-Selinene | C=C1CCC[C@]2([C@H]1C[C@@H](CC2)C(=C)C)C | 442393 |
| 82 | IMPHY014806 | Caswell No. 264AB | CC([C@@H]1CC[C@H]([C@]23[C@H]1[C@H]2C(=CC3)C)C)C | 442359 |
| 83 | IMPHY014811 | alpha-Phellandrene | CC1=CCC(C=C1)C(C)C | 7460 |
| 84 | IMPHY014817 | Aromadendrene | CC1CCC2C1C1C(C1(C)C)CCC2=C | 91354 |
| 85 | IMPHY014831 | beta-Caryophyllene | C/C/1=CCCC(=C)[C@@H]2[C@@H](CC1)C(C2)(C)C | 5281515 |
| 86 | IMPHY014842 | Stigmasterol | CC[C@@H](C(C)C)/C=C/[C@H]([C@H]1CC[C@@H]2[C@]1(C)CC[C@H]1[C@H]2CC=C2[C@]1(C)CC[C@@H](C2)O)C | 5280794 |
| 87 | IMPHY014847 | Bornyl acetate | CC(=O)OC1CC2C(C1(C)CC2)(C)C | 6448 |
| 88 | IMPHY014852 | Camphene | C=C1C2CCC(C1(C)C)C2 | 6616 |
| 89 | IMPHY014857 | Cedrol | C[C@@H]1CC[C@@H]2[C@@]31CC[C@@]([C@H](C3)C2(C)C)(C)O | 65575 |
| 90 | IMPHY014865 | Calamenene | CC([C@@H]1CC[C@@H](c2c1cc(C)cc2)C)C | 6429077 |
| 91 | IMPHY014986 | Ledol | C[C@@H]1CC[C@H]2[C@@H]1[C@H]1[C@H](C1(C)C)CC[C@@]2(C)O | 92812 |
| 92 | IMPHY014988 | Limonene | CC1=CCC(CC1)C(=C)C | 22311 |
| 93 | IMPHY015022 | Nerolidol | C=CC(CC/C=C(/CCC=C(C)C)C)(O)C | 5284507 |
| 94 | IMPHY015042 | Piperitone | CC1=CC(=O)C(CC1)C(C)C | 6987 |
| 95 | IMPHY015123 | alpha-Copaene | CC([C@@H]1CC[C@]2([C@@H]3[C@H]1C2C(=CC3)C)C)C | 70678558 |
| 96 | IMPHY015128 | T-Muurolol | CC1=C[C@@H]2[C@H](CC1)[C@@](C)(O)CC[C@H]2C(C)C | 3084331 |
| 97 | IMPHY016012 | Allo-Aromadendrene | C[C@@H]1CC[C@H]2[C@@H]1C1C(C1(C)C)CCC2=C | 42608158 |
| 98 | IMPHY017327 | p-Menthane-1,3-diol | CC(C1CCC(CC1O)(C)O)C | 524005 |
| 99 | IMPHY003681 | Diosgenin | C[C@@H]1CC[C@@]2(OC1)O[C@@H]1[C@H]([C@@H]2C)[C@@]2([C@@H](C1)[C@@H]1CC=C3[C@]([C@H]1CC2)(C)CC[C@@H](C3)O)C | 99474 |
| 100 | IMPHY004631 | Stearic acid | CCCCCCCCCCCCCCCCCC(=O)O | 5281 |
| 101 | IMPHY006558 | 1-Triacontanol | CCCCCCCCCCCCCCCCCCCCCCCCCCCCCCO | 68972 |
| 102 | IMPHY007317 | 4-Allylphenol | C=CCc1ccc(cc1)O | 68148 |
| 103 | IMPHY008910 | Hentriacontane | CCCCCCCCCCCCCCCCCCCCCCCCCCCCCCC | 12410 |
| 104 | IMPHY009485 | Pentatriacontane | CCCCCCCCCCCCCCCCCCCCCCCCCCCCCCCCCCC | 12413 |
| 105 | IMPHY012464 | Clionasterol | CC[C@H](C(C)C)CC[C@H]([C@H]1CC[C@@H]2[C@]1(C)CC[C@H]1[C@H]2CC=C2[C@]1(C)CC[C@@H](C2)O)C | 457801 |
| 106 | IMPHY014836 | beta-Sitosterol | CC[C@@H](C(C)C)CC[C@H]([C@H]1CC[C@@H]2[C@]1(C)CC[C@H]1[C@H]2CC=C2[C@]1(C)CC[C@@H](C2)O)C | 222284 |
| 107 | IMPHY007317 | 4-Allylphenol | C=CCc1ccc(cc1)O | 68148 |
| 108 | IMPHY001073 | Piperbetol | C=CC[C@]12C=C(OC)C(=O)[C@@H]([C@@H]2OC(=O)C)[C@H]([C@@H]1C)c1ccc(c(c1)OC)O | 10385474 |
| 109 | IMPHY001240 | Arecaidine | CN1CC(=CCC1)C(=O)O | 10355 |
| 110 | IMPHY001246 | Carvacrol | CC(c1ccc(c(c1)O)C)C | 10364 |
| 111 | IMPHY001368 | Piperol A | C=CC[C@@]12C[C@H]([C@H]([C@@H]2O)C(=O)C(=C1)OC)c1ccc(c(c1)OC)OC | 102586046 |
| 112 | IMPHY002191 | Piperol B | C=CC[C@]12C=C(OC)C(=O)[C@@H]([C@@H]2O)[C@H]([C@@H]1C)c1ccc(c(c1)OC)OC | 101715614 |
| 113 | IMPHY003129 | Allylpyrocatechol | Oc1c(CC=C)cccc1O | 292101 |
| 114 | IMPHY003536 | Eugenol | C=CCc1ccc(c(c1)OC)O | 3314 |
| 115 | IMPHY005639 | Arecoline | COC(=O)C1=CCCN(C1)C | 2230 |
| 116 | IMPHY006145 | p-Cymene | Cc1ccc(cc1)C(C)C | 7463 |
| 117 | IMPHY006297 | Chavibetol | C=CCc1ccc(c(c1)O)OC | 596375 |
| 118 | IMPHY006362 | Ascorbic acid | OC[C@@H]([C@H]1OC(=O)C(=C1O)O)O | 54670067 |
| 119 | IMPHY006593 | 4-Allylbenzene-1,2-diol | C=CCc1ccc(c(c1)O)O | 70775 |
| 120 | IMPHY006696 | Methyleugenol | C=CCc1ccc(c(c1)OC)OC | 7127 |
| 121 | IMPHY006944 | Estragole | COc1ccc(cc1)CC=C | 8815 |
| 122 | IMPHY007317 | 4-Allylphenol | C=CCc1ccc(cc1)O | 68148 |
| 123 | IMPHY007450 | Oxalic acid | OC(=O)C(=O)O | 971 |
| 124 | IMPHY007528 | Cadinane | C[C@H]1CC[C@@H]2[C@@H](C1)[C@@H](CC[C@@H]2C)C(C)C | 9548708 |
| 125 | IMPHY008892 | Methyl piperbetol | C=CC[C@]12C=C(OC)C(=O)[C@@H]([C@@H]2OC(=O)C)[C@H]([C@@H]1C)c1ccc(c(c1)OC)OC | 101715613 |
| 126 | IMPHY008937 | Vitamin E | C[C@@H](CCC[C@]1(C)CCc2c(O1)c(C)c(c(c2C)O)C)CCC[C@@H](CCCC(C)C)C | 14985 |
| 127 | IMPHY010072 | Eucalyptol | CC12CCC(CC1)C(O2)(C)C | 2758 |
| 128 | IMPHY011519 | alpha-Terpinyl acetate | CC(=O)OC(C1CCC(=CC1)C)(C)C | 111037 |
| 129 | IMPHY011643 | alpha-Terpinene | CC1=CC=C(CC1)C(C)C | 7462 |
| 130 | IMPHY011707 | beta-Carotene | C/C(=CC=CC=C(C=CC=C(C=CC1=C(C)CCCC1(C)C)/C)/C)/C=C/C=C(/C=C/C1=C(C)CCCC1(C)C)C | 5280489 |
| 131 | IMPHY012160 | alpha-Terpineol | CC1=CCC(CC1)C(O)(C)C | 17100 |
| 132 | IMPHY014831 | beta-Caryophyllene | C/C/1=CCCC(=C)[C@@H]2[C@@H](CC1)C(C2)(C)C | 5281515 |
|  |  |  |  |  |
| **PLANT NAME : *Piper cubeba*** | | | | |
| **Serial No** | **IMPPAT Phytochemical Identifier** | **Phytochemical Name** | **SMILES** | **CID** |
| 1 | IMPHY000022 | Myrcenol | C=CC(=C)CCCC(O)(C)C | 10975 |
| 2 | IMPHY000399 | beta-Bisabolene | CC(=CCCC(=C)[C@H]1CCC(=CC1)C)C | 10104370 |
| 3 | IMPHY000402 | 1,4-Cineole | CC(C12CCC(O2)(CC1)C)C | 10106 |
| 4 | IMPHY000602 | M-Cymene | Cc1cccc(c1)C(C)C | 10812 |
| 5 | IMPHY001246 | Carvacrol | CC(c1ccc(c(c1)O)C)C | 10364 |
| 6 | IMPHY001350 | (-)-Cubebinin | COc1c(OC)cc(cc1OC)C[C@H]1C(O)OC[C@@H]1Cc1cc(OC)c(c(c1)OC)OC | 44575401 |
| 7 | IMPHY001351 | Elemicin | C=CCc1cc(OC)c(c(c1)OC)OC | 10248 |
| 8 | IMPHY001516 | Decane | CCCCCCCCCC | 15600 |
| 9 | IMPHY001703 | Crotepoxide | CC(=O)O[C@H]1[C@H](OC(=O)C)[C@H]2O[C@H]2[C@@H]2[C@@]1(COC(=O)c1ccccc1)O2 | 161314 |
| 10 | IMPHY001912 | Cubebin | O[C@H]1OC[C@@H]([C@H]1Cc1ccc2c(c1)OCO2)Cc1ccc2c(c1)OCO2 | 117443 |
| 11 | IMPHY002875 | Undecane | CCCCCCCCCCC | 14257 |
| 12 | IMPHY003050 | Methyl salicylate | COC(=O)c1ccccc1O | 4133 |
| 13 | IMPHY003296 | Piperitenone | CC1=CC(=O)C(=C(C)C)CC1 | 381152 |
| 14 | IMPHY003485 | Myrcene | C=CC(=C)CCC=C(C)C | 31253 |
| 15 | IMPHY003497 | (-)-Dihydroclusin | OC[C@@H]([C@@H](Cc1ccc2c(c1)OCO2)CO)Cc1cc(OC)c(c(c1)OC)OC | 332806 |
| 16 | IMPHY003694 | Germacrene a | C/C/1=CCC/C(=C/C[C@@H](CC1)C(=C)C)/C | 9548705 |
| 17 | IMPHY003719 | beta-Copaene | CC([C@@H]1CC[C@]2([C@@H]3[C@H]1C2C(=C)CC3)C)C | 57339298 |
| 18 | IMPHY003723 | 2,4-Decadienal | CCCCC/C=C/C=C/C=O | 5283349 |
| 19 | IMPHY003760 | 2-Nonenal | CCCCCC/C=C/C=O | 5283335 |
| 20 | IMPHY003798 | (Z)-alpha-Bisabolene | CC(=CC/C=C(C1CCC(=CC1)C)/C)C | 5352653 |
| 21 | IMPHY003822 | Cubebol | CC([C@@H]1CC[C@H]([C@]23[C@H]1[C@H]2[C@@](C)(O)CC3)C)C | 11276107 |
| 22 | IMPHY003955 | (R)-4-Isopropylcyclohex-2-enone | CC([C@H]1CCC(=O)C=C1)C | 642520 |
| 23 | IMPHY003975 | Yatein | COc1cc(C[C@H]2C(=O)OC[C@@H]2Cc2ccc3c(c2)OCO3)cc(c1OC)OC | 442835 |
| 24 | IMPHY003977 | (-)-beta-Bourbonene | CC([C@@H]1CC[C@@]2([C@H]1[C@H]1C(=C)CC[C@@H]21)C)C | 62566 |
| 25 | IMPHY003982 | gamma-Terpinene | CC1=CCC(=CC1)C(C)C | 7461 |
| 26 | IMPHY004067 | Dihydrocarvyl acetate | CC(=O)OC1CC(CCC1C)C(=C)C | 30248 |
| 27 | IMPHY004147 | Isoyatein | COc1cc(C[C@H]2COC(=O)[C@@H]2Cc2ccc3c(c2)OCO3)cc(c1OC)OC | 10787153 |
| 28 | IMPHY004281 | Guaiol | C[C@H]1CC[C@H](CC2=C1CC[C@@H]2C)C(O)(C)C | 227829 |
| 29 | IMPHY004549 | Safrole | C=CCc1ccc2c(c1)OCO2 | 5144 |
| 30 | IMPHY005484 | 2,4,5-Trimethoxybenzaldehyde | COc1cc(C=O)c(cc1OC)OC | 20525 |
| 31 | IMPHY005521 | 1-Hexen-3-OL | CCCC(C=C)O | 20928 |
| 32 | IMPHY005618 | Germacrene B | C/C/1=CCC/C(=C/CC(=C(C)C)CC1)/C | 5281519 |
| 33 | IMPHY005653 | 2-Octanol | CCCCCCC(O)C | 20083 |
| 34 | IMPHY005801 | Dihydrocubebin | OC[C@@H]([C@@H](Cc1ccc2c(c1)OCO2)CO)Cc1ccc2c(c1)OCO2 | 193042 |
| 35 | IMPHY006145 | p-Cymene | Cc1ccc(cc1)C(C)C | 7463 |
| 36 | IMPHY006550 | Thymol | Cc1ccc(c(c1)O)C(C)C | 6989 |
| 37 | IMPHY006696 | Methyleugenol | C=CCc1ccc(c(c1)OC)OC | 7127 |
| 38 | IMPHY006948 | beta-Terpineol | CC(=C)C1CCC(CC1)(C)O | 8748 |
| 39 | IMPHY006950 | Tricyclene | CC12C3C1CC(C2(C)C)C3 | 79035 |
| 40 | IMPHY007041 | Furfural | O=Cc1ccco1 | 7362 |
| 41 | IMPHY007282 | (3R,4R)-3-((7-Methoxybenzo[d][1,3]dioxol-5-yl)methyl)-4-(3,4,5-trimethoxybenzyl)dihydrofuran-2(3H)-one | COc1cc(C[C@H]2COC(=O)[C@@H]2Cc2cc(OC)c3c(c2)OCO3)cc(c1OC)OC | 91724200 |
| 42 | IMPHY007286 | Umbellulon | CC1=CC(=O)C2(C1C2)C(C)C | 91195 |
| 43 | IMPHY007331 | 6-Methyl-5-hepten-2-one | CC(=O)CCC=C(C)C | 9862 |
| 44 | IMPHY007376 | beta-Cubebene | CC([C@@H]1CC[C@H]([C@]23[C@H]1[C@H]2C(=C)CC3)C)C | 93081 |
| 45 | IMPHY007421 | Citronellyl acetate | CC(CCC=C(C)C)CCOC(=O)C | 9017 |
| 46 | IMPHY007840 | Spathulenol | C=C1CC[C@@H]2[C@H]([C@H]3[C@H]1CC[C@]3(C)O)C2(C)C | 92231 |
| 47 | IMPHY008303 | 1-Hepten-3-OL | CCCCC(C=C)O | 21057 |
| 48 | IMPHY008369 | Methyl isobutyl ketone | CC(CC(=O)C)C | 7909 |
| 49 | IMPHY008903 | epi-Cubebol | CC([C@H]1C=C[C@@H]([C@@]23[C@@H]1C2C(C)(O)CC3)C)C | 91753433 |
| 50 | IMPHY009642 | 2-Nonanone | CCCCCCCC(=O)C | 13187 |
| 51 | IMPHY009739 | 5-Isopropylbicyclo[3.1.0]hexan-2-one | O=C1CCC2(C1C2)C(C)C | 92784 |
| 52 | IMPHY009756 | cis-Muurola-3,5-diene | CC1=CC[C@@H]2C(=C1)[C@H](CC[C@H]2C)C(C)C | 51351708 |
| 53 | IMPHY009765 | beta-Calacorene | CC(C1CCC(=C)c2c1cc(C)cc2)C | 529621 |
| 54 | IMPHY009946 | Benzaldehyde | O=Cc1ccccc1 | 240 |
| 55 | IMPHY009955 | Cyclohexane | C1CCCCC1 | 8078 |
| 56 | IMPHY010072 | Eucalyptol | CC12CCC(CC1)C(O2)(C)C | 2758 |
| 57 | IMPHY010080 | beta-Elemene | C=C[C@]1(C)CC[C@H](C[C@H]1C(=C)C)C(=C)C | 6918391 |
| 58 | IMPHY011002 | 3-Methyl-2-pentanol | CCC(C(O)C)C | 11261 |
| 59 | IMPHY011100 | alpha-Cedrene epoxide | CC1CCC2C31CC1OC1(C(C3)C2(C)C)C | 122510 |
| 60 | IMPHY011392 | 3-Carene | CC1=CCC2C(C1)C2(C)C | 26049 |
| 61 | IMPHY011396 | 4-Carvomenthenol | CC1=CCC(CC1)(O)C(C)C | 11230 |
| 62 | IMPHY011400 | (+)-Piperenol A | O[C@H]1C(=C[C@H]([C@@H]([C@@H]1OC(=O)c1ccccc1)O)O)COC(=O)c1ccccc1 | 14890278 |
| 63 | IMPHY011455 | Cadina-1,4-diene | CC1=CC2C(=CC1)[C@@H](C)CCC2C(C)C | 6427091 |
| 64 | IMPHY011519 | alpha-Terpinyl acetate | CC(=O)OC(C1CCC(=CC1)C)(C)C | 111037 |
| 65 | IMPHY011521 | 2-Undecanone | CCCCCCCCCC(=O)C | 8163 |
| 66 | IMPHY011542 | beta-Eudesmol | C=C1CCC[C@]2([C@H]1C[C@@H](CC2)C(O)(C)C)C | 91457 |
| 67 | IMPHY011552 | (1R)-2-methyl-5-propan-2-ylbicyclo[3.1.0]hex-2-ene | CC1=CCC2([C@@H]1C2)C(C)C | 6451618 |
| 68 | IMPHY011581 | alpha-Selinene | CC1=CCC[C@]2([C@H]1C[C@@H](CC2)C(=C)C)C | 10856614 |
| 69 | IMPHY011586 | (S,1Z,6Z)-8-Isopropyl-1-methyl-5-methylenecyclodeca-1,6-diene | C/C/1=C/CCC(=C)/C=C[C@@H](CC1)C(C)C | 91723653 |
| 70 | IMPHY011588 | cis-3-Hexen-1-ol | OCC/C=CCC | 5281167 |
| 71 | IMPHY011590 | d-Borneol | O[C@@H]1C[C@H]2C([C@@]1(C)CC2)(C)C | 61060 |
| 72 | IMPHY011599 | Terpinolene | CC1=CCC(=C(C)C)CC1 | 11463 |
| 73 | IMPHY011614 | beta-Cedrene | C=C1CC[C@@]23C[C@@H]1C(C)(C)[C@@H]2CC[C@H]3C | 11106485 |
| 74 | IMPHY011630 | cis,cis-Farnesol | OC/C=C(CC/C=C(CCC=C(C)C)/C)/C | 1549107 |
| 75 | IMPHY011631 | (E,Z)-farnesol | OC/C=C(/CC/C=C(CCC=C(C)C)/C)C | 1549109 |
| 76 | IMPHY011632 | Farnesol | OC/C=C(/CC/C=C(/CCC=C(C)C)C)C | 445070 |
| 77 | IMPHY011633 | (2Z,6E)-Farnesol | OC/C=C(CC/C=C(/CCC=C(C)C)C)/C | 1549108 |
| 78 | IMPHY011643 | alpha-Terpinene | CC1=CC=C(CC1)C(C)C | 7462 |
| 79 | IMPHY011658 | beta-Farnesene | C=CC(=C)CC/C=C(/CCC=C(C)C)C | 5281517 |
| 80 | IMPHY011659 | alpha-Muurolene | CC1=C[C@@H]2[C@H](CC1)C(=CC[C@H]2C(C)C)C | 12306047 |
| 81 | IMPHY011660 | (+)-alpha-Cadinene | CC1=C[C@@H]2[C@@H](CC1)C(=CC[C@H]2C(C)C)C | 12306048 |
| 82 | IMPHY011761 | Humulene | C/C/1=CCC(C)(C)/C=C/C/C(=C/CC1)/C | 5281520 |
| 83 | IMPHY011790 | Neral | O=C/C=C(CCC=C(C)C)/C | 643779 |
| 84 | IMPHY011792 | gamma-Muurolene | CC1=C[C@@H]2[C@H](CC1)C(=C)CC[C@H]2C(C)C | 12313020 |
| 85 | IMPHY011793 | (+)-gamma-Cadinene | CC1=C[C@@H]2[C@@H](CC1)C(=C)CC[C@H]2C(C)C | 6432404 |
| 86 | IMPHY011890 | Elemol | C=C[C@]1(C)CC[C@H](C[C@H]1C(=C)C)C(O)(C)C | 92138 |
| 87 | IMPHY011957 | (+)-delta-Cadinene | CC1=C[C@@H]2C(=C(C)CC[C@H]2C(C)C)CC1 | 441005 |
| 88 | IMPHY011988 | (-)-trans-Carveol | CC(=C)[C@@H]1CC=C([C@H](C1)O)C | 94221 |
| 89 | IMPHY012036 | Camphor | O=C1CC2C(C1(C)CC2)(C)C | 2537 |
| 90 | IMPHY011965 | (+)-beta-Phellandrene | CC([C@@H]1CCC(=C)C=C1)C | 442484 |
| 91 | IMPHY012058 | Linalool | C=CC(CCC=C(C)C)(O)C | 6549 |
| 92 | IMPHY012061 | alpha-Pinene | CC1=CCC2CC1C2(C)C | 6654 |
| 93 | IMPHY012147 | beta-Pinene | C=C1CCC2CC1C2(C)C | 14896 |
| 94 | IMPHY012160 | alpha-Terpineol | CC1=CCC(CC1)C(O)(C)C | 17100 |
| 95 | IMPHY012165 | Sabinene | C=C1CCC2(C1C2)C(C)C | 18818 |
| 96 | IMPHY012261 | alpha-Bergamotene | CC(=CCCC1(C)C2CC=C(C1C2)C)C | 86608 |
| 97 | IMPHY012279 | alpha-Curcumene | CC(=CCCC(c1ccc(cc1)C)C)C | 92139 |
| 98 | IMPHY012289 | epi-Globulol | C[C@H]1CC[C@@H]2[C@H]1[C@@H]1[C@@H](C1(C)C)CC[C@@]2(C)O | 7308311 |
| 99 | IMPHY012337 | Heterotropan | COc1cc(OC)c(cc1C1C(C)C(C1c1cc(OC)c(cc1OC)OC)C)OC | 126324 |
| 100 | IMPHY012586 | (-)-alpha-Cadinol | CC1=CC2C(CC1)[C@@](C)(O)CC[C@@H]2C(C)C | 6431302 |
| 101 | IMPHY012596 | Selin-11-en-4alpha-ol | CC(=C)[C@@H]1CC[C@@]2([C@@H](C1)[C@](C)(O)CCC2)C | 15560330 |
| 102 | IMPHY012654 | Nerol | OC/C=C(CCC=C(C)C)/C | 643820 |
| 103 | IMPHY012665 | Levomenol | CC(=CCC[C@@]([C@H]1CCC(=CC1)C)(O)C)C | 442343 |
| 104 | IMPHY012667 | Caryophyllene oxide | C=C1CC[C@H]2O[C@@]2(CC[C@@H]2[C@@H]1CC2(C)C)C | 1742210 |
| 105 | IMPHY012910 | trans-Calamenene | CC([C@H]1CC[C@@H](c2c1cc(C)cc2)C)C | 6429022 |
| 106 | IMPHY012921 | gamma-Elemene | C=C[C@]1(C)CCC(=C(C)C)C[C@H]1C(=C)C | 6432312 |
| 107 | IMPHY013080 | alpha-Calacorene | CC([C@@H]1CC=C(c2c1cc(C)cc2)C)C | 12302243 |
| 108 | IMPHY013093 | delta-Elemene | C=C[C@@]1(C)CCC(=C[C@@H]1C(=C)C)C(C)C | 12309449 |
| 109 | IMPHY013190 | (+)-Zeylenol | O=C(c1ccccc1)OCC1(O)C(O)C=CC(C1O)OC(=O)c1ccccc1 | 14283260 |
| 110 | IMPHY013246 | Piperenol B | O=C(c1ccccc1)OCC1(O)C=CC(C(C1O)O)OC(=O)c1ccccc1 | 14890283 |
| 111 | IMPHY013838 | 3,7-Dimethyloct-6-en-3-ol | CCC(CCC=C(C)C)(O)C | 86749 |
| 112 | IMPHY013971 | epi-Cubenol | CC1=C[C@@H]2[C@@](CC1)(O)[C@H](C)CC[C@H]2C(C)C | 12046149 |
| 113 | IMPHY013972 | 1,10-Di-epcubenol | CC1=C[C@@H]2[C@](CC1)(O)[C@H](C)CC[C@@H]2C(C)C | 91748749 |
| 114 | IMPHY014690 | (-)-Globulol | C[C@@H]1CC[C@@H]2[C@@H]1[C@H]1[C@H](C1(C)C)CC[C@@]2(C)O | 12304985 |
| 115 | IMPHY014708 | beta-Selinene | C=C1CCC[C@]2([C@H]1C[C@@H](CC2)C(=C)C)C | 442393 |
| 116 | IMPHY014805 | Cedr-8-ene | CC1=CC[C@@]23C[C@@H]1C(C)(C)[C@@H]2CC[C@H]3C | 6431015 |
| 117 | IMPHY014806 | Caswell No. 264AB | CC([C@@H]1CC[C@H]([C@]23[C@H]1[C@H]2C(=CC3)C)C)C | 442359 |
| 118 | IMPHY014811 | alpha-Phellandrene | CC1=CCC(C=C1)C(C)C | 7460 |
| 119 | IMPHY014831 | beta-Caryophyllene | C/C/1=CCCC(=C)[C@@H]2[C@@H](CC1)C(C2)(C)C | 5281515 |
| 120 | IMPHY014833 | beta-Himachalene | CC1=C[C@H]2C(=C(C)CCCC2(C)C)CC1 | 11586487 |
| 121 | IMPHY014835 | (E)-beta-ocimene | C=C/C(=C/CC=C(C)C)/C | 5281553 |
| 122 | IMPHY014847 | Bornyl acetate | CC(=O)OC1CC2C(C1(C)CC2)(C)C | 6448 |
| 123 | IMPHY014852 | Camphene | C=C1C2CCC(C1(C)C)C2 | 6616 |
| 124 | IMPHY014857 | Cedrol | C[C@@H]1CC[C@@H]2[C@@]31CC[C@@]([C@H](C3)C2(C)C)(C)O | 65575 |
| 125 | IMPHY014863 | cis-alpha-Bergamotene | CC(=CCCC1(C)[C@@H]2CC=C([C@H]1C2)C)C | 91753502 |
| 126 | IMPHY014865 | Calamenene | CC([C@@H]1CC[C@@H](c2c1cc(C)cc2)C)C | 6429077 |
| 127 | IMPHY014873 | 2-Cyclohexen-1-ol, 3-methyl-6-(1-methylethyl)-, (1R,6S)-rel- | CC1=C[C@@H]([C@@H](CC1)C(C)C)O | 85567 |
| 128 | IMPHY014874 | cis-Sabinene hydrate | C[C@@H]1CC[C@@]2(C1C2)C(C)C | 101629835 |
| 129 | IMPHY014877 | (S)-cis-Verbenol | CC1=C[C@H](O)[C@H]2C[C@@H]1C2(C)C | 87839 |
| 130 | IMPHY014881 | Copaene | CC(C1CCC2(C3C1C2C(=CC3)C)C)C | 19725 |
| 131 | IMPHY014906 | Cedrelanol | CC1=C[C@@H]2[C@@H](CC1)[C@@](C)(O)CC[C@H]2C(C)C | 160799 |
| 132 | IMPHY014907 | 6-Epi-beta-bisabolol | CC(=CCC[C@@H]([C@@]1(O)CCC(=CC1)C)C)C | 12300148 |
| 133 | IMPHY014914 | Fenchol | OC1C2(C)CCC(C1(C)C)C2 | 15406 |
| 134 | IMPHY014923 | Geraniol | OC/C=C(/CCC=C(C)C)C | 637566 |
| 135 | IMPHY014986 | Ledol | C[C@@H]1CC[C@H]2[C@@H]1[C@H]1[C@H](C1(C)C)CC[C@@]2(C)O | 92812 |
| 136 | IMPHY014988 | Limonene | CC1=CCC(CC1)C(=C)C | 22311 |
| 137 | IMPHY015016 | alpha-Muurolol | CC1=C[C@@H]2[C@H](CC1)[C@](C)(O)CC[C@@H]2C(C)C | 91753440 |
| 138 | IMPHY015022 | Nerolidol | C=CC(CC/C=C(/CCC=C(C)C)C)(O)C | 5284507 |
| 139 | IMPHY015062 | (+)-cis-Sabinol | CC([C@]12C[C@@H]2C(=C)[C@@H](C1)O)C | 94147 |
| 140 | IMPHY015063 | Sabinyl acetate | CC(=O)OC1CC2(C(C1=C)C2)C(C)C | 94266 |
| 141 | IMPHY015120 | ((1R,2S,5R,6S)-5-(Benzoyloxy)-1,2,6-trihydroxycyclohex-3-en-1-yl)methyl benzoate | O=C(c1ccccc1)OC[C@@]1(O)[C@@H](O)C=C[C@H]([C@@H]1O)OC(=O)c1ccccc1 | 10992619 |
| 142 | IMPHY015123 | alpha-Copaene | CC([C@@H]1CC[C@]2([C@@H]3[C@H]1C2C(=CC3)C)C)C | 70678558 |
| 143 | IMPHY015128 | T-Muurolol | CC1=C[C@@H]2[C@H](CC1)[C@@](C)(O)CC[C@H]2C(C)C | 3084331 |
| 144 | IMPHY015380 | 4-Methyl-2-pentanol | CC(CC(O)C)C | 7910 |
| 145 | IMPHY015411 | 5-Methylheptan-3-one | CCC(CC(=O)CC)C | 7822 |
| 146 | IMPHY015752 | Isoaromadendrene epoxide | CC1CCC2C1C1C(C1(C)C)CC1C2(C)O1 | 534398 |
| 147 | IMPHY016012 | Allo-Aromadendrene | C[C@@H]1CC[C@H]2[C@@H]1C1C(C1(C)C)CCC2=C | 42608158 |
| 148 | IMPHY016027 | trans-Sabinene hydrate | CC([C@@]12CC[C@](C2C1)(C)O)C | 12315151 |
| 149 | IMPHY016053 | Viridiflorol | C[C@@H]1CC[C@H]2[C@@H]1[C@H]1[C@H](C1(C)C)CC[C@]2(C)O | 11996452 |
| 150 | IMPHY016054 | trans-alpha-Bergamotene | CC(=CCC[C@]1(C)[C@H]2CC=C([C@@H]1C2)C)C | 6429302 |
| 151 | IMPHY016229 | 2-Ethyl-4-pentenal | C=CCC(C=O)CC | 21264 |
| 152 | IMPHY016281 | 2-Methylundecanal | CCCCCCCCCC(C=O)C | 61031 |
| 153 | IMPHY016575 | cis-Muurol-5-en-4-beta-ol | CC1CCC2C(=C1)[C@](O)(CC[C@@H]2C)C(C)C | 6428412 |
| 154 | IMPHY016597 | Cycloisolongifolene | CC1(C)CCCC23C41CC(CC24)C3(C)C | 563197 |
| 155 | IMPHY016895 | gamma-Gurjunene epoxide | CC([C@@H]1CC[C@H]([C@@H]2C3(C1O3)C(C)CC2)C)C | 91750423 |
| 156 | IMPHY017044 | Dihydroocimene | CCC(/C=C/C=C(C)C)C | 129640025 |
| 157 | IMPHY017124 | beta Farnesene | CCC(=C)CC/C=C(/CCC=C(C)C)C | 15228937 |
| 158 | IMPHY017645 | Cadina-1,4-diene-3-ol | CC(C1CCC(C2=CC(C(=CC12)C)O)C)C | 6431196 |
| 159 | IMPHY000602 | M-Cymene | Cc1cccc(c1)C(C)C | 10812 |
| 160 | IMPHY001516 | Decane | CCCCCCCCCC | 15600 |
| 161 | IMPHY002875 | Undecane | CCCCCCCCCCC | 14257 |
| 162 | IMPHY003050 | Methyl salicylate | COC(=O)c1ccccc1O | 4133 |
| 163 | IMPHY003485 | Myrcene | C=CC(=C)CCC=C(C)C | 31253 |
| 164 | IMPHY003723 | 2,4-Decadienal | CCCCC/C=C/C=C/C=O | 5283349 |
| 165 | IMPHY003760 | 2-Nonenal | CCCCCC/C=C/C=O | 5283335 |
| 166 | IMPHY003798 | (Z)-alpha-Bisabolene | CC(=CC/C=C(C1CCC(=CC1)C)/C)C | 5352653 |
| 167 | IMPHY003822 | Cubebol | CC([C@@H]1CC[C@H]([C@]23[C@H]1[C@H]2[C@@](C)(O)CC3)C)C | 11276107 |
| 168 | IMPHY003955 | (R)-4-Isopropylcyclohex-2-enone | CC([C@H]1CCC(=O)C=C1)C | 642520 |
| 169 | IMPHY003977 | (-)-beta-Bourbonene | CC([C@@H]1CC[C@@]2([C@H]1[C@H]1C(=C)CC[C@@H]21)C)C | 62566 |
| 170 | IMPHY003982 | gamma-Terpinene | CC1=CCC(=CC1)C(C)C | 7461 |
| 171 | IMPHY004281 | Guaiol | C[C@H]1CC[C@H](CC2=C1CC[C@@H]2C)C(O)(C)C | 227829 |
| 172 | IMPHY005618 | Germacrene B | C/C/1=CCC/C(=C/CC(=C(C)C)CC1)/C | 5281519 |
| 173 | IMPHY005653 | 2-Octanol | CCCCCCC(O)C | 20083 |
| 174 | IMPHY006145 | p-Cymene | Cc1ccc(cc1)C(C)C | 7463 |
| 175 | IMPHY006950 | Tricyclene | CC12C3C1CC(C2(C)C)C3 | 79035 |
| 176 | IMPHY007286 | Umbellulon | CC1=CC(=O)C2(C1C2)C(C)C | 91195 |
| 177 | IMPHY007331 | 6-Methyl-5-hepten-2-one | CC(=O)CCC=C(C)C | 9862 |
| 178 | IMPHY007376 | beta-Cubebene | CC([C@@H]1CC[C@H]([C@]23[C@H]1[C@H]2C(=C)CC3)C)C | 93081 |
| 179 | IMPHY007840 | Spathulenol | C=C1CC[C@@H]2[C@H]([C@H]3[C@H]1CC[C@]3(C)O)C2(C)C | 92231 |
| 180 | IMPHY008903 | epi-Cubebol | CC([C@H]1C=C[C@@H]([C@@]23[C@@H]1C2C(C)(O)CC3)C)C | 91753433 |
| 181 | IMPHY009642 | 2-Nonanone | CCCCCCCC(=O)C | 13187 |
| 182 | IMPHY009739 | 5-Isopropylbicyclo[3.1.0]hexan-2-one | O=C1CCC2(C1C2)C(C)C | 92784 |
| 183 | IMPHY009756 | cis-Muurola-3,5-diene | CC1=CC[C@@H]2C(=C1)[C@H](CC[C@H]2C)C(C)C | 51351708 |
| 184 | IMPHY009765 | beta-Calacorene | CC(C1CCC(=C)c2c1cc(C)cc2)C | 529621 |
| 185 | IMPHY009946 | Benzaldehyde | O=Cc1ccccc1 | 240 |
| 186 | IMPHY010072 | Eucalyptol | CC12CCC(CC1)C(O2)(C)C | 2758 |
| 187 | IMPHY010080 | beta-Elemene | C=C[C@]1(C)CC[C@H](C[C@H]1C(=C)C)C(=C)C | 6918391 |
| 188 | IMPHY011392 | 3-Carene | CC1=CCC2C(C1)C2(C)C | 26049 |
| 189 | IMPHY011396 | 4-Carvomenthenol | CC1=CCC(CC1)(O)C(C)C | 11230 |
| 190 | IMPHY011521 | 2-Undecanone | CCCCCCCCCC(=O)C | 8163 |
| 191 | IMPHY011542 | beta-Eudesmol | C=C1CCC[C@]2([C@H]1C[C@@H](CC2)C(O)(C)C)C | 91457 |
| 192 | IMPHY011552 | (1R)-2-methyl-5-propan-2-ylbicyclo[3.1.0]hex-2-ene | CC1=CCC2([C@@H]1C2)C(C)C | 6451618 |
| 193 | IMPHY011586 | (S,1Z,6Z)-8-Isopropyl-1-methyl-5-methylenecyclodeca-1,6-diene | C/C/1=C/CCC(=C)/C=C[C@@H](CC1)C(C)C | 91723653 |
| 194 | IMPHY011590 | d-Borneol | O[C@@H]1C[C@H]2C([C@@]1(C)CC2)(C)C | 61060 |
| 195 | IMPHY011599 | Terpinolene | CC1=CCC(=C(C)C)CC1 | 11463 |
| 196 | IMPHY011632 | Farnesol | OC/C=C(/CC/C=C(/CCC=C(C)C)C)C | 445070 |
| 197 | IMPHY011633 | (2Z,6E)-Farnesol | OC/C=C(CC/C=C(/CCC=C(C)C)C)/C | 1549108 |
| 198 | IMPHY011643 | alpha-Terpinene | CC1=CC=C(CC1)C(C)C | 7462 |
| 199 | IMPHY011658 | beta-Farnesene | C=CC(=C)CC/C=C(/CCC=C(C)C)C | 5281517 |
| 200 | IMPHY011659 | alpha-Muurolene | CC1=C[C@@H]2[C@H](CC1)C(=CC[C@H]2C(C)C)C | 12306047 |
| 201 | IMPHY011660 | (+)-alpha-Cadinene | CC1=C[C@@H]2[C@@H](CC1)C(=CC[C@H]2C(C)C)C | 12306048 |
| 202 | IMPHY011761 | Humulene | C/C/1=CCC(C)(C)/C=C/C/C(=C/CC1)/C | 5281520 |
| 203 | IMPHY011792 | gamma-Muurolene | CC1=C[C@@H]2[C@H](CC1)C(=C)CC[C@H]2C(C)C | 12313020 |
| 204 | IMPHY011793 | (+)-gamma-Cadinene | CC1=C[C@@H]2[C@@H](CC1)C(=C)CC[C@H]2C(C)C | 6432404 |
| 205 | IMPHY011890 | Elemol | C=C[C@]1(C)CC[C@H](C[C@H]1C(=C)C)C(O)(C)C | 92138 |
| 206 | IMPHY011957 | (+)-delta-Cadinene | CC1=C[C@@H]2C(=C(C)CC[C@H]2C(C)C)CC1 | 441005 |
| 207 | IMPHY011965 | (+)-beta-Phellandrene | CC([C@@H]1CCC(=C)C=C1)C | 442484 |
| 208 | IMPHY012036 | Camphor | O=C1CC2C(C1(C)CC2)(C)C | 2537 |
| 209 | IMPHY012058 | Linalool | C=CC(CCC=C(C)C)(O)C | 6549 |
| 210 | IMPHY012061 | alpha-Pinene | CC1=CCC2CC1C2(C)C | 6654 |
| 211 | IMPHY012147 | beta-Pinene | C=C1CCC2CC1C2(C)C | 14896 |
| 212 | IMPHY012160 | alpha-Terpineol | CC1=CCC(CC1)C(O)(C)C | 17100 |
| 213 | IMPHY012165 | Sabinene | C=C1CCC2(C1C2)C(C)C | 18818 |
| 214 | IMPHY012289 | epi-Globulol | C[C@H]1CC[C@@H]2[C@H]1[C@@H]1[C@@H](C1(C)C)CC[C@@]2(C)O | 7308311 |
| 215 | IMPHY012586 | (-)-alpha-Cadinol | CC1=CC2C(CC1)[C@@](C)(O)CC[C@@H]2C(C)C | 6431302 |
| 216 | IMPHY012596 | Selin-11-en-4alpha-ol | CC(=C)[C@@H]1CC[C@@]2([C@@H](C1)[C@](C)(O)CCC2)C | 15560330 |
| 217 | IMPHY012654 | Nerol | OC/C=C(CCC=C(C)C)/C | 643820 |
| 218 | IMPHY012665 | Levomenol | CC(=CCC[C@@]([C@H]1CCC(=CC1)C)(O)C)C | 442343 |
| 219 | IMPHY012910 | trans-Calamenene | CC([C@H]1CC[C@@H](c2c1cc(C)cc2)C)C | 6429022 |
| 220 | IMPHY012921 | gamma-Elemene | C=C[C@]1(C)CCC(=C(C)C)C[C@H]1C(=C)C | 6432312 |
| 221 | IMPHY013080 | alpha-Calacorene | CC([C@@H]1CC=C(c2c1cc(C)cc2)C)C | 12302243 |
| 222 | IMPHY013971 | epi-Cubenol | CC1=C[C@@H]2[C@@](CC1)(O)[C@H](C)CC[C@H]2C(C)C | 12046149 |
| 223 | IMPHY013972 | 1,10-Di-epcubenol | CC1=C[C@@H]2[C@](CC1)(O)[C@H](C)CC[C@@H]2C(C)C | 91748749 |
| 224 | IMPHY014690 | (-)-Globulol | C[C@@H]1CC[C@@H]2[C@@H]1[C@H]1[C@H](C1(C)C)CC[C@@]2(C)O | 12304985 |
| 225 | IMPHY014806 | Caswell No. 264AB | CC([C@@H]1CC[C@H]([C@]23[C@H]1[C@H]2C(=CC3)C)C)C | 442359 |
| 226 | IMPHY014811 | alpha-Phellandrene | CC1=CCC(C=C1)C(C)C | 7460 |
| 227 | IMPHY014831 | beta-Caryophyllene | C/C/1=CCCC(=C)[C@@H]2[C@@H](CC1)C(C2)(C)C | 5281515 |
| 228 | IMPHY014833 | beta-Himachalene | CC1=C[C@H]2C(=C(C)CCCC2(C)C)CC1 | 11586487 |
| 229 | IMPHY014835 | (E)-beta-ocimene | C=C/C(=C/CC=C(C)C)/C | 5281553 |
| 230 | IMPHY014852 | Camphene | C=C1C2CCC(C1(C)C)C2 | 6616 |
| 231 | IMPHY014863 | cis-alpha-Bergamotene | CC(=CCCC1(C)[C@@H]2CC=C([C@H]1C2)C)C | 91753502 |
| 232 | IMPHY014865 | Calamenene | CC([C@@H]1CC[C@@H](c2c1cc(C)cc2)C)C | 6429077 |
| 233 | IMPHY014873 | 2-Cyclohexen-1-ol, 3-methyl-6-(1-methylethyl)-, (1R,6S)-rel- | CC1=C[C@@H]([C@@H](CC1)C(C)C)O | 85567 |
| 234 | IMPHY014874 | cis-Sabinene hydrate | C[C@@H]1CC[C@@]2(C1C2)C(C)C | 101629835 |
| 235 | IMPHY014877 | (S)-cis-Verbenol | CC1=C[C@H](O)[C@H]2C[C@@H]1C2(C)C | 87839 |
| 236 | IMPHY014906 | Cedrelanol | CC1=C[C@@H]2[C@@H](CC1)[C@@](C)(O)CC[C@H]2C(C)C | 160799 |
| 237 | IMPHY014907 | 6-Epi-beta-bisabolol | CC(=CCC[C@@H]([C@@]1(O)CCC(=CC1)C)C)C | 12300148 |
| 238 | IMPHY014923 | Geraniol | OC/C=C(/CCC=C(C)C)C | 637566 |
| 239 | IMPHY014986 | Ledol | C[C@@H]1CC[C@H]2[C@@H]1[C@H]1[C@H](C1(C)C)CC[C@@]2(C)O | 92812 |
| 240 | IMPHY014988 | Limonene | CC1=CCC(CC1)C(=C)C | 22311 |
| 241 | IMPHY015016 | alpha-Muurolol | CC1=C[C@@H]2[C@H](CC1)[C@](C)(O)CC[C@@H]2C(C)C | 91753440 |
| 242 | IMPHY015022 | Nerolidol | C=CC(CC/C=C(/CCC=C(C)C)C)(O)C | 5284507 |
| 243 | IMPHY015123 | alpha-Copaene | CC([C@@H]1CC[C@]2([C@@H]3[C@H]1C2C(=CC3)C)C)C | 70678558 |
| 244 | IMPHY015128 | T-Muurolol | CC1=C[C@@H]2[C@H](CC1)[C@@](C)(O)CC[C@H]2C(C)C | 3084331 |
| 245 | IMPHY015411 | 5-Methylheptan-3-one | CCC(CC(=O)CC)C | 7822 |
| 246 | IMPHY015752 | Isoaromadendrene epoxide | CC1CCC2C1C1C(C1(C)C)CC1C2(C)O1 | 534398 |
| 247 | IMPHY016012 | Allo-Aromadendrene | C[C@@H]1CC[C@H]2[C@@H]1C1C(C1(C)C)CCC2=C | 42608158 |
| 248 | IMPHY016027 | trans-Sabinene hydrate | CC([C@@]12CC[C@](C2C1)(C)O)C | 12315151 |
| 249 | IMPHY016053 | Viridiflorol | C[C@@H]1CC[C@H]2[C@@H]1[C@H]1[C@H](C1(C)C)CC[C@]2(C)O | 11996452 |
| 250 | IMPHY016054 | trans-alpha-Bergamotene | CC(=CCC[C@]1(C)[C@H]2CC=C([C@@H]1C2)C)C | 6429302 |
| 251 | IMPHY016229 | 2-Ethyl-4-pentenal | C=CCC(C=O)CC | 21264 |
| 252 | IMPHY016281 | 2-Methylundecanal | CCCCCCCCCC(C=O)C | 61031 |
| 253 | IMPHY016575 | cis-Muurol-5-en-4-beta-ol | CC1CCC2C(=C1)[C@](O)(CC[C@@H]2C)C(C)C | 6428412 |
| 254 | IMPHY016597 | Cycloisolongifolene | CC1(C)CCCC23C41CC(CC24)C3(C)C | 563197 |
| 255 | IMPHY016895 | gamma-Gurjunene epoxide | CC([C@@H]1CC[C@H]([C@@H]2C3(C1O3)C(C)CC2)C)C | 91750423 |
| 256 | IMPHY001912 | Cubebin | O[C@H]1OC[C@@H]([C@H]1Cc1ccc2c(c1)OCO2)Cc1ccc2c(c1)OCO2 | 117443 |
| 257 | IMPHY004631 | Stearic acid | CCCCCCCCCCCCCCCCCC(=O)O | 5281 |
| 258 | IMPHY007212 | Docosanoic acid | CCCCCCCCCCCCCCCCCCCCCC(=O)O | 8215 |
| 259 | IMPHY007327 | Palmitic acid | CCCCCCCCCCCCCCCC(=O)O | 985 |
| 260 | IMPHY011394 | Arachidic acid | CCCCCCCCCCCCCCCCCCCC(=O)O | 10467 |
| 261 | IMPHY011797 | Oleic acid | CCCCCCCC/C=CCCCCCCCC(=O)O | 445639 |
| 262 | IMPHY014990 | Linoleic acid | CCCCC/C=CC/C=CCCCCCCCC(=O)O | 5280450 |
| 263 | IMPHY000018 | (1alpha,2beta,5alpha,8alpha,10alpha)-1,10-Epoxy-2-hydroxy-3,7(11)-guaiadien-12,8-olide | CC1=C2C[C@H]3C(=C[C@H]([C@]43[C@@](C[C@@H]2OC1=O)(O4)C)O)C | 11299991 |
| 264 | IMPHY000527 | Cubebinic ether | CC(=O)OC[C@@H]1Cc2cc3OCOc3cc2C=C1Cc1ccc2c(c1)OCO2 |  |
| 265 | IMPHY000529 | Cubebinol | OC[C@@H]1Cc2cc3OCOc3cc2C=C1Cc1ccc2c(c1)OCO2 |  |
| 266 | IMPHY000534 | Isocubebinic ether | C1Oc2c(O1)ccc(c2)C[C@@H]1[C@H]2COC1c1c(C2)cc2c(c1)OCO2 |  |
| 267 | IMPHY001410 | Ethoxyclusin | CCOc1cc(C[C@H]2COC([C@@H]2Cc2cc(OC)c(c(c2)OC)OC)O)cc2c1OCO2 | 44575400 |
| 268 | IMPHY001912 | Cubebin | O[C@H]1OC[C@@H]([C@H]1Cc1ccc2c(c1)OCO2)Cc1ccc2c(c1)OCO2 | 117443 |
| 269 | IMPHY002997 | 2(3H)-Furanone, dihydro-3-(3,4,5-trimethoxybenzyl)-4-veratryl-, trans- | COc1ccc(cc1OC)C[C@H]1COC(=O)[C@@H]1Cc1cc(OC)c(c(c1)OC)OC | 384878 |
| 270 | IMPHY003497 | (-)-Dihydroclusin | OC[C@@H]([C@@H](Cc1ccc2c(c1)OCO2)CO)Cc1cc(OC)c(c(c1)OC)OC | 332806 |
| 271 | IMPHY003822 | Cubebol | CC([C@@H]1CC[C@H]([C@]23[C@H]1[C@H]2[C@@](C)(O)CC3)C)C | 11276107 |
| 272 | IMPHY003975 | Yatein | COc1cc(C[C@H]2C(=O)OC[C@@H]2Cc2ccc3c(c2)OCO3)cc(c1OC)OC | 442835 |
| 273 | IMPHY004073 | (5alpha,8alpha)-2-Oxo-1(10),3,7(11)-guaiatrien-12,8-olide | CC1=C2C[C@H]3C(=CC(=O)C3=C(C[C@@H]2OC1=O)C)C | 3013843 |
| 274 | IMPHY004147 | Isoyatein | COc1cc(C[C@H]2COC(=O)[C@@H]2Cc2ccc3c(c2)OCO3)cc(c1OC)OC | 10787153 |
| 275 | IMPHY005484 | 2,4,5-Trimethoxybenzaldehyde | COc1cc(C=O)c(cc1OC)OC | 20525 |
| 276 | IMPHY005562 | Hexadecenoic acid | CCCCCCCCCCCCC/C=C/C(=O)O | 5282743 |
| 277 | IMPHY005801 | Dihydrocubebin | OC[C@@H]([C@@H](Cc1ccc2c(c1)OCO2)CO)Cc1ccc2c(c1)OCO2 | 193042 |
| 278 | IMPHY007188 | Piperidine | C1CCCNC1 | 8082 |
| 279 | IMPHY007282 | (3R,4R)-3-((7-Methoxybenzo[d][1,3]dioxol-5-yl)methyl)-4-(3,4,5-trimethoxybenzyl)dihydrofuran-2(3H)-one | COc1cc(C[C@H]2COC(=O)[C@@H]2Cc2cc(OC)c3c(c2)OCO3)cc(c1OC)OC | 91724200 |
| 280 | IMPHY008944 | Cubenene | C12C3C4C2=C2C1C3=C42 | 57357909 |
| 281 | IMPHY010049 | Artesin[sesquiterpene] | O=C1O[C@H]2[C@H]([C@@H]1C)CC[C@@]1(C2=C(C)CC[C@H]1O)C | 10422228 |
| 282 | IMPHY011659 | alpha-Muurolene | CC1=C[C@@H]2[C@H](CC1)C(=CC[C@H]2C(C)C)C | 12306047 |
| 283 | IMPHY011920 | Isohinokinin | O=C1OC[C@@H]([C@@H]1Cc1ccc2c(c1)OCO2)Cc1ccc2c(c1)OCO2 | 10871927 |
| 284 | IMPHY011931 | Hinokinin | O=C1OC[C@@H]([C@H]1Cc1ccc2c(c1)OCO2)Cc1ccc2c(c1)OCO2 | 442879 |
| 285 | IMPHY011957 | (+)-delta-Cadinene | CC1=C[C@@H]2C(=C(C)CC[C@H]2C(C)C)CC1 | 441005 |
| 286 | IMPHY012638 | Epizonarene | CC1=CC2=C(CCC(C2CC1)C)C(C)C | 595385 |
| 287 | IMPHY014721 | 1-epi-Bicyclosesquiphellandrene | C=C1CCC2C(=C1)C(CCC2C)C(C)C | 521496 |
| 288 | IMPHY014865 | Calamenene | CC([C@@H]1CC[C@@H](c2c1cc(C)cc2)C)C | 6429077 |
| 289 | IMPHY014895 | 5-[3-(1,3-Benzodioxol-5-yl)-1,3,3a,4,6,6a-hexahydrofuro[3,4-c]furan-6-yl]-1,3-benzodioxole | C1Oc2c(O1)cc(cc2)C1OCC2C1COC2c1ccc2c(c1)OCO2 | 5204 |
| 290 | IMPHY000402 | 1,4-Cineole | CC(C12CCC(O2)(CC1)C)C | 10106 |
| 291 | IMPHY001350 | (-)-Cubebinin | COc1c(OC)cc(cc1OC)C[C@H]1C(O)OC[C@@H]1Cc1cc(OC)c(c(c1)OC)OC | 44575401 |
| 292 | IMPHY001410 | Ethoxyclusin | CCOc1cc(C[C@H]2COC([C@@H]2Cc2cc(OC)c(c(c2)OC)OC)O)cc2c1OCO2 | 44575400 |
| 293 | IMPHY001703 | Crotepoxide | CC(=O)O[C@H]1[C@H](OC(=O)C)[C@H]2O[C@H]2[C@@H]2[C@@]1(COC(=O)c1ccccc1)O2 | 161314 |
| 294 | IMPHY001912 | Cubebin | O[C@H]1OC[C@@H]([C@H]1Cc1ccc2c(c1)OCO2)Cc1ccc2c(c1)OCO2 | 117443 |
| 295 | IMPHY002997 | 2(3H)-Furanone, dihydro-3-(3,4,5-trimethoxybenzyl)-4-veratryl-, trans- | COc1ccc(cc1OC)C[C@H]1COC(=O)[C@@H]1Cc1cc(OC)c(c(c1)OC)OC | 384878 |
| 296 | IMPHY003497 | (-)-Dihydroclusin | OC[C@@H]([C@@H](Cc1ccc2c(c1)OCO2)CO)Cc1cc(OC)c(c(c1)OC)OC | 332806 |
| 297 | IMPHY003822 | Cubebol | CC([C@@H]1CC[C@H]([C@]23[C@H]1[C@H]2[C@@](C)(O)CC3)C)C | 11276107 |
| 298 | IMPHY003975 | Yatein | COc1cc(C[C@H]2C(=O)OC[C@@H]2Cc2ccc3c(c2)OCO3)cc(c1OC)OC | 442835 |
| 299 | IMPHY004147 | Isoyatein | COc1cc(C[C@H]2COC(=O)[C@@H]2Cc2ccc3c(c2)OCO3)cc(c1OC)OC | 10787153 |
| 300 | IMPHY004192 | Piperine | O=C(N1CCCCC1)/C=C/C=C/c1ccc2c(c1)OCO2 | 638024 |
| 301 | IMPHY005484 | 2,4,5-Trimethoxybenzaldehyde | COc1cc(C=O)c(cc1OC)OC | 20525 |
| 302 | IMPHY005801 | Dihydrocubebin | OC[C@@H]([C@@H](Cc1ccc2c(c1)OCO2)CO)Cc1ccc2c(c1)OCO2 | 193042 |
| 303 | IMPHY007282 | (3R,4R)-3-((7-Methoxybenzo[d][1,3]dioxol-5-yl)methyl)-4-(3,4,5-trimethoxybenzyl)dihydrofuran-2(3H)-one | COc1cc(C[C@H]2COC(=O)[C@@H]2Cc2cc(OC)c3c(c2)OCO3)cc(c1OC)OC | 91724200 |
| 304 | IMPHY010072 | Eucalyptol | CC12CCC(CC1)C(O2)(C)C | 2758 |
| 305 | IMPHY010603 | beta-Cadinene | CC1=CC[C@@H]2[C@@H](C1)[C@@H](CC=C2C)C(C)C | 10657 |
| 306 | IMPHY011313 | (-)-Car-4-ene | C[C@@H]1C=C[C@@H]2[C@H](C1)C2(C)C | 16211587 |
| 307 | IMPHY011931 | Hinokinin | O=C1OC[C@@H]([C@H]1Cc1ccc2c(c1)OCO2)Cc1ccc2c(c1)OCO2 | 442879 |
| 308 | IMPHY012337 | Heterotropan | COc1cc(OC)c(cc1C1C(C)C(C1c1cc(OC)c(cc1OC)OC)C)OC | 126324 |
| 309 | IMPHY013190 | (+)-Zeylenol | O=C(c1ccccc1)OCC1(O)C(O)C=CC(C1O)OC(=O)c1ccccc1 | 14283260 |
| 310 | IMPHY013463 | Clusin | COc1cc(CC2C(O)OCC2Cc2ccc3c(c2)OCO3)cc(c1OC)OC | 44575537 |
| 311 | IMPHY014721 | 1-epi-Bicyclosesquiphellandrene | C=C1CCC2C(=C1)C(CCC2C)C(C)C | 521496 |
| 312 | IMPHY014895 | 5-[3-(1,3-Benzodioxol-5-yl)-1,3,3a,4,6,6a-hexahydrofuro[3,4-c]furan-6-yl]-1,3-benzodioxole | C1Oc2c(O1)cc(cc2)C1OCC2C1COC2c1ccc2c(c1)OCO2 | 5204 |
|  |  |  |  |  |
| **PLANT NAME : *Piper longum*** | | | | |
| **Serial No** | **IMPPAT Phytochemical Identifier** | **Phytochemical Name** | **SMILES** | **CID** |
| 1 | IMPHY001915 | Octadecane | CCCCCCCCCCCCCCCCCC | 5320621 |
| 2 | IMPHY003053 | 1-Pentadecene | CCCCCCCCCCCCCC=C | 637858 |
| 3 | IMPHY003329 | Norcepharadione B | COc1cc2C(=O)C(=O)Nc3c2c(c1OC)c1ccccc1c3 | 5204 |
| 4 | IMPHY003485 | Myrcene | C=CC(=C)CCC=C(C)C | 11006 |
| 5 | IMPHY004341 | Piperlonguminine | CC(CNC(=O)/C=C/C=C/c1ccc2c(c1)OCO2)C | 44453654 |
| 6 | IMPHY006342 | Piperlongumine | COc1cc(/C=C/C(=O)N2CCC=CC2=O)cc(c1OC)OC | 11622 |
| 7 | IMPHY014895 | 5-[3-(1,3-Benzodioxol-5-yl)-1,3,3a,4,6,6a-hexahydrofuro[3,4-c]furan-6-yl]-1,3-benzodioxole | C1Oc2c(O1)cc(cc2)C1OCC2C1COC2c1ccc2c(c1)OCO2 | 11635 |
| 8 | IMPHY000308 | Hexadecane | CCCCCCCCCCCCCCCC | 25913 |
| 9 | IMPHY001313 | Piperundecalidine | O=C(N1CCCCC1)/C=C/C=C/CCCC/C=C/c1ccc2c(c1)OCO2 | 189168 |
| 10 | IMPHY001881 | 2-Tridecanone | CCCCCCCCCCCC(=O)C | 31253 |
| 11 | IMPHY006279 | 2-Phenylethanol | OCCc1ccccc1 | 6054 |
| 12 | IMPHY005260 | Aristolodione | COc1c(O)cc2c3c1c1ccccc1cc3N(C(=O)C2=O)C | 184116 |
| 13 | IMPHY005360 | Aristolactam BII | COc1c(OC)cc2c3c1c1ccccc1cc3NC2=O | 162739 |
| 14 | IMPHY006145 | p-Cymene | Cc1ccc(cc1)C(C)C | 7463 |
| 15 | IMPHY006149 | 4'-Methoxyacetophenone | COc1ccc(cc1)C(=O)C | 7476 |
| 16 | IMPHY006184 | Dehydropipernonaline | O=C(N1CCCCC1)/C=C/C=C/CC/C=C/c1ccc2c(c1)OCO2 | 6439947 |
| 17 | IMPHY006266 | Guineensine | CC(CNC(=O)/C=C/C=C/CCCCCC/C=C/c1ccc2c(c1)OCO2)C | 6442405 |
| 18 | IMPHY004192 | Piperine | O=C(N1CCCCC1)/C=C/C=C/c1ccc2c(c1)OCO2 | 638024 |
| 19 | IMPHY006342 | Piperlongumine | COc1cc(/C=C/C(=O)N2CCC=CC2=O)cc(c1OC)OC | 637858 |
| 20 | IMPHY006624 | Pluviatilol | COc1cc(ccc1O)[C@H]1OC[C@@H]2[C@H]1CO[C@H]2c1ccc2c(c1)OCO2 | 70695727 |
| 21 | IMPHY006951 | Eicosane | CCCCCCCCCCCCCCCCCCCC | 8222 |
| 22 | IMPHY006970 | Decanal | CCCCCCCCCC=O | 8175 |
| 23 | IMPHY006992 | 1-Methylhexyl acetate | CCCCCC(OC(=O)C)C | 80018 |
| 24 | IMPHY007204 | Dodecanal | CCCCCCCCCCCC=O | 8194 |
| 25 | IMPHY007376 | beta-Cubebene | CC([C@@H]1CC[C@H]([C@]23[C@H]1[C@H]2C(=C)CC3)C)C | 93081 |
| 26 | IMPHY007528 | Cadinane | C[C@H]1CC[C@@H]2[C@@H](C1)[C@@H](CC[C@@H]2C)C(C)C | 9548708 |
| 27 | IMPHY007559 | Pipernonaline | O=C(N1CCCCC1)/C=C/CCCC/C=C/c1ccc2c(c1)OCO2 | 9974595 |
| 28 | IMPHY007632 | Aristololactam | COc1cccc2c1cc1NC(=O)c3c1c2c1OCOc1c3 | 96710 |
| 29 | IMPHY009368 | Heptadecane | CCCCCCCCCCCCCCCCC | 12398 |
| 30 | IMPHY009369 | Nonadecane | CCCCCCCCCCCCCCCCCCC | 12401 |
| 31 | IMPHY009382 | Heneicosane | CCCCCCCCCCCCCCCCCCCCC | 12403 |
| 32 | IMPHY009389 | Pentadecane | CCCCCCCCCCCCCCC | 12391 |
| 33 | IMPHY009419 | Tridecane | CCCCCCCCCCCCC | 12388 |
| 34 | IMPHY009642 | 2-Nonanone | CCCCCCCC(=O)C | 13187 |
| 35 | IMPHY010072 | Eucalyptol | CC12CCC(CC1)C(O2)(C)C | 2758 |
| 36 | IMPHY010080 | beta-Elemene | C=C[C@]1(C)CC[C@H](C[C@H]1C(=C)C)C(=C)C | 6918391 |
| 37 | IMPHY011464 | Isobutyramide | CC(C(=O)N)C | 68424 |
| 38 | IMPHY011519 | alpha-Terpinyl acetate | CC(=O)OC(C1CCC(=CC1)C)(C)C | 111037 |
| 39 | IMPHY011521 | 2-Undecanone | CCCCCCCCCC(=O)C | 8163 |
| 40 | IMPHY011542 | beta-Eudesmol | C=C1CCC[C@]2([C@H]1C[C@@H](CC2)C(O)(C)C)C | 91457 |
| 41 | IMPHY011552 | (1R)-2-methyl-5-propan-2-ylbicyclo[3.1.0]hex-2-ene | CC1=CCC2([C@@H]1C2)C(C)C | 6451618 |
| 42 | IMPHY011567 | Fargesin | COc1cc(ccc1OC)[C@@H]1OC[C@H]2[C@@H]1CO[C@@H]2c1ccc2c(c1)OCO2 | 10926754 |
| 43 | IMPHY011593 | Pellitorine | CCCCC/C=C/C=C/C(=O)NCC(C)C | 5318516 |
| 44 | IMPHY011599 | Terpinolene | CC1=CCC(=C(C)C)CC1 | 11463 |
| 45 | IMPHY011641 | (+)-alpha-Gurjunene | C[C@H]1CC[C@H]2[C@@H](C3=C(CC[C@@H]13)C)C2(C)C | 15560275 |
| 46 | IMPHY011659 | alpha-Muurolene | CC1=C[C@@H]2[C@H](CC1)C(=CC[C@H]2C(C)C)C | 12306047 |
| 47 | IMPHY011709 | alpha-Eudesmol | CC1=CCC[C@]2([C@H]1C[C@@H](CC2)C(O)(C)C)C | 92762 |
| 48 | IMPHY011745 | Zingiberene | CC(=CCC[C@@H]([C@H]1CC=C(C=C1)C)C)C | 92776 |
| 49 | IMPHY011761 | Humulene | C/C/1=CCC(C)(C)/C=C/C/C(=C/CC1)/C | 5281520 |
| 50 | IMPHY011792 | gamma-Muurolene | CC1=C[C@@H]2[C@H](CC1)C(=C)CC[C@H]2C(C)C | 12313020 |
| 51 | IMPHY011839 | (Z)-gamma-bisabolene | CC(=CCC/C(=C1/CCC(=CC1)C)/C)C | 3033866 |
| 52 | IMPHY011890 | Elemol | C=C[C@]1(C)CC[C@H](C[C@H]1C(=C)C)C(O)(C)C | 92138 |
| 53 | IMPHY011965 | (+)-beta-Phellandrene | CC([C@@H]1CCC(=C)C=C1)C | 442484 |
| 54 | IMPHY012058 | Linalool | C=CC(CCC=C(C)C)(O)C | 6549 |
| 55 | IMPHY012061 | alpha-Pinene | CC1=CCC2CC1C2(C)C | 6654 |
| 56 | IMPHY012130 | Dihydrocarveol | CC(=C)C1CCC(C(C1)O)C | 12072 |
| 57 | IMPHY012147 | beta-Pinene | C=C1CCC2CC1C2(C)C | 14896 |
| 58 | IMPHY012160 | alpha-Terpineol | CC1=CCC(CC1)C(O)(C)C | 17100 |
| 59 | IMPHY012305 | beta-Patchoulene | CC1CCC2=C1CC1CCC2(C1(C)C)C | 101731 |
| 60 | IMPHY012353 | Aristolactam AII | COc1c(O)cc2c3c1c1ccccc1cc3NC2=O | 148657 |
| 61 | IMPHY012585 | delta-Cadinol | CC1=C[C@@H]2[C@H](CC1)[C@](C)(O)CC[C@H]2C(C)C | 3084311 |
| 62 | IMPHY012586 | (-)-alpha-Cadinol | CC1=CC2C(CC1)[C@@](C)(O)CC[C@@H]2C(C)C | 6431302 |
| 63 | IMPHY012615 | 9-Eicosyne | CCCCCCCCCCC#CCCCCCCCC | 557019 |
| 64 | IMPHY012667 | Caryophyllene oxide | C=C1CC[C@H]2O[C@@]2(CC[C@@H]2[C@@H]1CC2(C)C)C | 1742210 |
| 65 | IMPHY012739 | (Z)-beta-Ocimene | C=C/C(=CCC=C(C)C)/C | 5320250 |
| 66 | IMPHY012921 | gamma-Elemene | C=C[C@]1(C)CCC(=C(C)C)C[C@H]1C(=C)C | 6432312 |
| 67 | IMPHY013093 | delta-Elemene | C=C[C@@]1(C)CCC(=C[C@@H]1C(=C)C)C(C)C | 12309449 |
| 68 | IMPHY014811 | alpha-Phellandrene | CC1=CCC(C=C1)C(C)C | 7460 |
| 69 | IMPHY014831 | beta-Caryophyllene | C/C/1=CCCC(=C)[C@@H]2[C@@H](CC1)C(C2)(C)C | 5281515 |
| 70 | IMPHY014835 | (E)-beta-ocimene | C=C/C(=C/CC=C(C)C)/C | 5281553 |
| 71 | IMPHY014847 | Bornyl acetate | CC(=O)OC1CC2C(C1(C)CC2)(C)C | 6448 |
| 72 | IMPHY014852 | Camphene | C=C1C2CCC(C1(C)C)C2 | 6616 |
| 73 | IMPHY014881 | Copaene | CC(C1CCC2(C3C1C2C(=CC3)C)C)C | 19725 |
| 74 | IMPHY014895 | 5-[3-(1,3-Benzodioxol-5-yl)-1,3,3a,4,6,6a-hexahydrofuro[3,4-c]furan-6-yl]-1,3-benzodioxole | C1Oc2c(O1)cc(cc2)C1OCC2C1COC2c1ccc2c(c1)OCO2 | 5204 |
| 75 | IMPHY014988 | Limonene | CC1=CCC(CC1)C(=C)C | 22311 |
| 76 | IMPHY015022 | Nerolidol | C=CC(CC/C=C(/CCC=C(C)C)C)(O)C | 5284507 |
| 77 | IMPHY001881 | 2-Tridecanone | CCCCCCCCCCCC(=O)C | 11622 |
| 78 | IMPHY003053 | 1-Pentadecene | CCCCCCCCCCCCCC=C | 25913 |
| 79 | IMPHY003485 | Myrcene | C=CC(=C)CCC=C(C)C | 31253 |
| 80 | IMPHY006970 | Decanal | CCCCCCCCCC=O | 31253 |
| 81 | IMPHY006992 | 1-Methylhexyl acetate | CCCCCC(OC(=O)C)C | 80018 |
| 82 | IMPHY007204 | Dodecanal | CCCCCCCCCCCC=O | 8194 |
| 83 | IMPHY007376 | beta-Cubebene | CC([C@@H]1CC[C@H]([C@]23[C@H]1[C@H]2C(=C)CC3)C)C | 93081 |
| 84 | IMPHY007528 | Cadinane | C[C@H]1CC[C@@H]2[C@@H](C1)[C@@H](CC[C@@H]2C)C(C)C | 9548708 |
| 85 | IMPHY009368 | Heptadecane | CCCCCCCCCCCCCCCCC | 12398 |
| 86 | IMPHY009389 | 2-Nonanone | CCCCCCCCCCCCCCC | 12391 |
| 87 | IMPHY009419 | Tridecane | CCCCCCCCCCCCC | 12388 |
| 88 | IMPHY009642 | 2-Nonanone | CCCCCCCC(=O)C | 13187 |
| 89 | IMPHY010072 | Eucalyptol | CC12CCC(CC1)C(O2)(C)C | 2758 |
| 90 | IMPHY010080 | beta-Elemene | C=C[C@]1(C)CC[C@H](C[C@H]1C(=C)C)C(=C)C | 6918391 |
| 91 | IMPHY011519 | alpha-Terpinyl acetate | CC(=O)OC(C1CCC(=CC1)C)(C)C | 111037 |
| 92 | IMPHY011521 | 2-Undecanone | CC(=O)OC(C1CCC(=CC1)C)(C)C | 8163 |
| 93 | IMPHY011542 | beta-Eudesmol | C=C1CCC[C@]2([C@H]1C[C@@H](CC2)C(O)(C)C)C | 91457 |
| 94 | IMPHY011599 | Terpinolene | CC1=CCC(=C(C)C)CC1 | 11463 |
| 95 | IMPHY011641 | (+)-alpha-Gurjunene | C[C@H]1CC[C@H]2[C@@H](C3=C(CC[C@@H]13)C)C2(C)C | 15560275 |
| 96 | IMPHY011659 | alpha-Muurolene | CC1=C[C@@H]2[C@H](CC1)C(=CC[C@H]2C(C)C)C | 12306047 |
| 97 | IMPHY011709 | alpha-Eudesmol | CC1=CCC[C@]2([C@H]1C[C@@H](CC2)C(O)(C)C)C | 92762 |
| 98 | IMPHY011761 | Humulene | C/C/1=CCC(C)(C)/C=C/C/C(=C/CC1)/C | 5281520 |
| 99 | IMPHY011792 | gamma-Muurolene | CC1=C[C@@H]2[C@H](CC1)C(=C)CC[C@H]2C(C)C | 12313020 |
| 100 | IMPHY011839 | (Z)-gamma-bisabolene | CC(=CCC/C(=C1/CCC(=CC1)C)/C)C | 3033866 |
| 101 | IMPHY011890 | Elemol | C=C[C@]1(C)CC[C@H](C[C@H]1C(=C)C)C(O)(C)C | 92138 |
| 102 | IMPHY011965 | (+)-beta-Phellandrene | CC([C@@H]1CCC(=C)C=C1)C | 442484 |
| 103 | IMPHY012058 | Linalool | C=CC(CCC=C(C)C)(O)C | 6549 |
| 104 | IMPHY012061 | alpha-Pinene | CC1=CCC2CC1C2(C)C | 6654 |
| 105 | IMPHY012147 | beta-Pinene | C=C1CCC2CC1C2(C)C | 14896 |
| 106 | IMPHY012160 | alpha-Terpineol | CC1=CCC(CC1)C(O)(C)C | 17100 |
| 107 | IMPHY012305 | beta-Patchoulene | CC1CCC2=C1CC1CCC2(C1(C)C)C | 101731 |
| 108 | IMPHY012585 | delta-Cadinol | CC1=C[C@@H]2[C@H](CC1)[C@](C)(O)CC[C@H]2C(C)C | 3084311 |
| 109 | IMPHY012586 | (-)-alpha-Cadinol | CC1=CC2C(CC1)[C@@](C)(O)CC[C@@H]2C(C)C | 6431302 |
| 110 | IMPHY012615 | 9-Eicosyne | CCCCCCCCCCC#CCCCCCCCC | 557019 |
| 111 | IMPHY012667 | Caryophyllene oxide | C=C1CC[C@H]2O[C@@]2(CC[C@@H]2[C@@H]1CC2(C)C)C | 1742210 |
| 112 | IMPHY012739 | (Z)-beta-Ocimene | C=C/C(=CCC=C(C)C)/C | 5320250 |
| 113 | IMPHY012921 | gamma-Elemene | C=C[C@]1(C)CCC(=C(C)C)C[C@H]1C(=C)C | 6432312 |
| 114 | IMPHY013093 | delta-Elemene | C=C[C@@]1(C)CCC(=C[C@@H]1C(=C)C)C(C)C | 12309449 |
| 115 | IMPHY014811 | alpha-Phellandrene | CC1=CCC(C=C1)C(C)C | 7460 |
| 116 | IMPHY014835 | (E)-beta-ocimene | C=C/C(=C/CC=C(C)C)/C | 5281553 |
| 117 | MPHY014847 | Bornyl acetate | CC(=O)OC1CC2C(C1(C)CC2)(C)C | 6448 |
| 118 | IMPHY014852 | Camphene | C=C1C2CCC(C1(C)C)C2 | 6616 |
| 119 | IMPHY014881 | Copaene | CC(C1CCC2(C3C1C2C(=CC3)C)C)C | 19725 |
| 120 | IMPHY014831 | beta-Caryophyllene | C/C/1=CCCC(=C)[C@@H]2[C@@H](CC1)C(C2)(C)C | 5281515 |
| 121 | IMPHY014988 | Limonene | CC1=CCC(CC1)C(=C)C | 22311 |
| 122 | IMPHY015022 | Nerolidol | C=CC(CC/C=C(/CCC=C(C)C)C)(O)C | 5284507 |
| 123 | IMPHY001881 | 2-Tridecanone | CCCCCCCCCCCC(=O)C | 11622 |
| 124 | IMPHY003053 | 1-Pentadecene | CCCCCCCCCCCCCC=C | 25913 |
| 125 | IMPHY003328 | Cepharadione B | COc1cc2C(=O)C(=O)N(c3c2c(c1OC)c1ccccc1c3)C | 189151 |
| 126 | IMPHY003329 | Norcepharadione B | COc1cc2C(=O)C(=O)Nc3c2c(c1OC)c1ccccc1c3 | 189168 |
| 127 | IMPHY003485 | Myrcene | C=CC(=C)CCC=C(C)C | 31253 |
| 128 | IMPHY004192 | Piperine | O=C(N1CCCCC1)/C=C/C=C/c1ccc2c(c1)OCO2 | 638024 |
| 129 | IMPHY004341 | Piperlonguminine | CC(CNC(=O)/C=C/C=C/c1ccc2c(c1)OCO2)C | 5320621 |
| 130 | IMPHY005260 | Aristolodione | COc1c(O)cc2c3c1c1ccccc1cc3N(C(=O)C2=O)C | 184116 |
| 131 | IMPHY005360 | Aristolactam BII | COc1c(OC)cc2c3c1c1ccccc1cc3NC2=O | 162739 |
| 132 | IMPHY006342 | Piperlongumine | COc1cc(/C=C/C(=O)N2CCC=CC2=O)cc(c1OC)OC | 637858 |
| 133 | IMPHY006970 | Decanal | CCCCCCCCCC=O | 8175 |
| 134 | IMPHY006992 | 1-Methylhexyl acetate | CCCCCC(OC(=O)C)C | 80018 |
| 135 | IMPHY007017 | 5-[3-(1,3-Benzodioxol-5-yl)-1,3,3a,4,6,6a-hexahydrofuro[3,4-c]furan-6-yl]-1,3-benzodioxole | COC(=O)/C=C/c1cc(OC)c(c(c1)OC)OC | 735846 |
| 136 | IMPHY007204 | Dodecanal | CCCCCCCCCCCC=O | 8194 |
| 137 | IMPHY007371 | Cepharadione A | O=C1N(C)c2cc3ccccc3c3c2c(C1=O)cc1c3OCO1 | 94577 |
| 138 | IMPHY007376 | beta-Cubebene | CC([C@@H]1CC[C@H]([C@]23[C@H]1[C@H]2C(=C)CC3)C)C | 93081 |
| 139 | IMPHY007528 | Cadinane | C[C@H]1CC[C@@H]2[C@@H](C1)[C@@H](CC[C@@H]2C)C(C)C | 9548708 |
| 140 | IMPHY009368 | Heptadecane | CCCCCCCCCCCCCCCCC | 12398 |
| 141 | IMPHY009389 | Pentadecane | CCCCCCCCCCCCCCC | 12391 |
| 142 | IMPHY009413 | Triacontane | CCCCCCCCCCCCCCCCCCCCCCCCCCCCCC | 12535 |
| 143 | IMPHY009419 | Tridecane | CCCCCCCCCCCCC | 12388 |
| 144 | IMPHY009642 | 2-Nonanone | CCCCCCCC(=O)C | 13187 |
| 145 | IMPHY010072 | Eucalyptol | CC12CCC(CC1)C(O2)(C)C | 2758 |
| 146 | IMPHY010080 | beta-Elemene | C=C[C@]1(C)CC[C@H](C[C@H]1C(=C)C)C(=C)C | 6918391 |
| 147 | IMPHY011519 | alpha-Terpinyl acetate | CC(=O)OC(C1CCC(=CC1)C)(C)C | 111037 |
| 148 | IMPHY011521 | 2-Undecanone | CCCCCCCCCC(=O)C | 8163 |
| 149 | IMPHY011542 | beta-Eudesmol | C=C1CCC[C@]2([C@H]1C[C@@H](CC2)C(O)(C)C)C | 91457 |
| 150 | IMPHY011599 | Terpinolene | CC1=CCC(=C(C)C)CC1 | 11463 |
| 151 | IMPHY011641 | (+)-alpha-Gurjunene | C[C@H]1CC[C@H]2[C@@H](C3=C(CC[C@@H]13)C)C2(C)C | 15560275 |
| 152 | IMPHY011659 | alpha-Muurolene | CC1=C[C@@H]2[C@H](CC1)C(=CC[C@H]2C(C)C)C | 12306047 |
| 153 | IMPHY011709 | alpha-Eudesmol | CC1=CCC[C@]2([C@H]1C[C@@H](CC2)C(O)(C)C)C | 92762 |
| 154 | IMPHY011761 | Humulene | C/C/1=CCC(C)(C)/C=C/C/C(=C/CC1)/C | 5281520 |
| 155 | IMPHY011792 | gamma-Muurolene | CC1=C[C@@H]2[C@H](CC1)C(=C)CC[C@H]2C(C)C | 12313020 |
| 156 | IMPHY011839 | (Z)-gamma-bisabolene | CC(=CCC/C(=C1/CCC(=CC1)C)/C)C | 3033866 |
| 157 | IMPHY011890 | Elemol | C=C[C@]1(C)CC[C@H](C[C@H]1C(=C)C)C(O)(C)C | 92138 |
| 158 | IMPHY011965 | (+)-beta-Phellandrene | CC([C@@H]1CCC(=C)C=C1)C | 442484 |
| 159 | IMPHY012058 | Linalool | C=CC(CCC=C(C)C)(O)C | 6549 |
| 160 | IMPHY012061 | alpha-Pinene | CC1=CCC2CC1C2(C)C | 6654 |
| 161 | IMPHY012147 | beta-Pinene | C=C1CCC2CC1C2(C)C | 14896 |
| 162 | IMPHY012160 | alpha-Terpineol | CC1=CCC(CC1)C(O)(C)C | 17100 |
| 163 | IMPHY012305 | beta-Patchoulene | CC1CCC2=C1CC1CCC2(C1(C)C)C | 101731 |
| 164 | IMPHY012585 | delta-Cadinol | CC1=C[C@@H]2[C@H](CC1)[C@](C)(O)CC[C@H]2C(C)C | 3084311 |
| 165 | IMPHY012586 | (-)-alpha-Cadinol | CC1=CC2C(CC1)[C@@](C)(O)CC[C@@H]2C(C)C | 6431302 |
| 166 | IMPHY012615 | 9-Eicosyne | CCCCCCCCCCC#CCCCCCCCC | 557019 |
| 167 | IMPHY012667 | Caryophyllene oxide | C=C1CC[C@H]2O[C@@]2(CC[C@@H]2[C@@H]1CC2(C)C)C | 1742210 |
| 168 | IMPHY012679 | Piperolactam A | COc1cc2C(=O)Nc3c2c(c1O)c1ccccc1c3 | 3081016 |
| 169 | IMPHY012739 | (Z)-beta-Ocimene | C=C/C(=CCC=C(C)C)/C | 5320250 |
| 170 | IMPHY012921 | gamma-Elemene | C=C[C@]1(C)CCC(=C(C)C)C[C@H]1C(=C)C | 6432312 |
| 171 | IMPHY013093 | delta-Elemene | C=C[C@@]1(C)CCC(=C[C@@H]1C(=C)C)C(C)C | 12309449 |
| 172 | IMPHY014811 | alpha-Phellandrene | CC1=CCC(C=C1)C(C)C | 7460 |
| 173 | IMPHY014831 | beta-Caryophyllene | C/C/1=CCCC(=C)[C@@H]2[C@@H](CC1)C(C2)(C)C | 5281515 |
| 174 | IMPHY014835 | (E)-beta-ocimene | C=C/C(=C/CC=C(C)C)/C | 5281553 |
| 175 | IMPHY014847 | Bornyl acetate | CC(=O)OC1CC2C(C1(C)CC2)(C)C | 6448 |
| 176 | IMPHY014852 | Camphene | C=C1C2CCC(C1(C)C)C2 | 6616 |
| 177 | IMPHY014881 | Copaene | CC(C1CCC2(C3C1C2C(=CC3)C)C)C | 19725 |
| 178 | IMPHY014895 | 5-[3-(1,3-Benzodioxol-5-yl)-1,3,3a,4,6,6a-hexahydrofuro[3,4-c]furan-6-yl]-1,3-benzodioxole | C1Oc2c(O1)cc(cc2)C1OCC2C1COC2c1ccc2c(c1)OCO2 | 5204 |
| 179 | IMPHY014988 | Limonene | CC1=CCC(CC1)C(=C)C | 22311 |
| 180 | IMPHY015022 | Nerolidol | C=CC(CC/C=C(/CCC=C(C)C)C)(O)C | 5284507 |
| 181 | IMPHY011462 | Sylvatine | CC(CCC/C=C/CCCCNC(=O)/C=C/C=C/c1ccc2c(c1)OCO2)C | 90472536 |
| 182 | IMPHY012967 | Diaeudesmin | COc1cc(ccc1OC)[C@@H]1OC[C@H]2[C@@H]1CO[C@H]2c1ccc(c(c1)OC)OC | 6992053 |
| 183 | IMPHY014895 | 5-[3-(1,3-Benzodioxol-5-yl)-1,3,3a,4,6,6a-hexahydrofuro[3,4-c]furan-6-yl]-1,3-benzodioxole | C1Oc2c(O1)cc(cc2)C1OCC2C1COC2c1ccc2c(c1)OCO2 | 5204 |
| 184 | IMPHY001881 | 2-Tridecanone | CCCCCCCCCCCC(=O)C | 11622 |
| 185 | IMPHY003053 | 1-Pentadecene | CCCCCCCCCCCCCC=C | 25913 |
| 186 | IMPHY003485 | Myrcene | C=CC(=C)CCC=C(C)C | 31253 |
| 187 | IMPHY004192 | Piperine | O=C(N1CCCCC1)/C=C/C=C/c1ccc2c(c1)OCO2 | 638024 |
| 188 | IMPHY004341 | Piperlonguminine | CC(CNC(=O)/C=C/C=C/c1ccc2c(c1)OCO2)C | 5320621 |
| 189 | IMPHY006342 | Piperlongumine | COc1cc(/C=C/C(=O)N2CCC=CC2=O)cc(c1OC)OC | 637858 |
| 190 | IMPHY006970 | Decanal | CCCCCCCCCC=O | 8175 |
| 191 | IMPHY006992 | 1-Methylhexyl acetate | CCCCCC(OC(=O)C)C | 80018 |
| 192 | IMPHY007204 | Dodecanal | CCCCCCCCCCCC=O | 8194 |
| 193 | IMPHY007376 | beta-Cubebene | CC([C@@H]1CC[C@H]([C@]23[C@H]1[C@H]2C(=C)CC3)C)C | 93081 |
| 194 | IMPHY007528 | Cadinane | C[C@H]1CC[C@@H]2[C@@H](C1)[C@@H](CC[C@@H]2C)C(C)C | 9548708 |
| 195 | IMPHY009368 | Heptadecane | CCCCCCCCCCCCCCCCC | 12398 |
| 196 | IMPHY009389 | Pentadecane | CCCCCCCCCCCCCCC | 12391 |
| 197 | IMPHY009419 | Tridecane | CCCCCCCCCCCCC | 12388 |
| 198 | IMPHY009642 | 2-Nonanone | CCCCCCCC(=O)C | 13187 |
| 199 | IMPHY010072 | Eucalyptol | CC12CCC(CC1)C(O2)(C)C | 2758 |
| 200 | IMPHY010080 | beta-Elemene | C=C[C@]1(C)CC[C@H](C[C@H]1C(=C)C)C(=C)C | 6918391 |
| 201 | IMPHY011519 | alpha-Terpinyl acetate | CC(=O)OC(C1CCC(=CC1)C)(C)C | 111037 |
| 202 | IMPHY011521 | 2-Undecanone | CCCCCCCCCC(=O)C | 8163 |
| 203 | IMPHY011542 | beta-Eudesmol | C=C1CCC[C@]2([C@H]1C[C@@H](CC2)C(O)(C)C)C | 91457 |
| 204 | IMPHY011599 | Terpinolene | CC1=CCC(=C(C)C)CC1 | 11463 |
| 205 | IMPHY011641 | (+)-alpha-Gurjunene | C[C@H]1CC[C@H]2[C@@H](C3=C(CC[C@@H]13)C)C2(C)C | 15560275 |
| 206 | IMPHY011659 | alpha-Muurolene | CC1=C[C@@H]2[C@H](CC1)C(=CC[C@H]2C(C)C)C | 12306047 |
| 207 | IMPHY011709 | alpha-Eudesmol | CC1=CCC[C@]2([C@H]1C[C@@H](CC2)C(O)(C)C)C | 92762 |
| 208 | IMPHY011761 | Humulene | C/C/1=CCC(C)(C)/C=C/C/C(=C/CC1)/C | 5281520 |
| 209 | IMPHY011792 | gamma-Muurolene | CC1=C[C@@H]2[C@H](CC1)C(=C)CC[C@H]2C(C)C | 12313020 |
| 210 | IMPHY011839 | (Z)-gamma-bisabolene | CC(=CCC/C(=C1/CCC(=CC1)C)/C)C | 3033866 |
| 211 | IMPHY011890 | Elemol | C=C[C@]1(C)CC[C@H](C[C@H]1C(=C)C)C(O)(C)C | 92138 |
| 212 | IMPHY011965 | (+)-beta-Phellandrene | CC([C@@H]1CCC(=C)C=C1)C | 442484 |
| 213 | IMPHY012058 | Linalool | C=CC(CCC=C(C)C)(O)C | 6549 |
| 214 | IMPHY012061 | alpha-Pinene | CC1=CCC2CC1C2(C)C | 6654 |
| 215 | IMPHY012147 | beta-Pinene | C=C1CCC2CC1C2(C)C | 14896 |
| 216 | IMPHY012160 | alpha-Terpineol | CC1=CCC(CC1)C(O)(C)C | 17100 |
| 217 | IMPHY012305 | beta-Patchoulene | CC1CCC2=C1CC1CCC2(C1(C)C)C | 101731 |
| 218 | IMPHY012585 | delta-Cadinol | CC1=C[C@@H]2[C@H](CC1)[C@](C)(O)CC[C@H]2C(C)C | 3084311 |
| 219 | IMPHY012586 | (-)-alpha-Cadinol | CC1=CC2C(CC1)[C@@](C)(O)CC[C@@H]2C(C)C | 6431302 |
| 220 | IMPHY012615 | 9-Eicosyne | CCCCCCCCCCC#CCCCCCCCC | 557019 |
| 221 | IMPHY012667 | Caryophyllene oxide | C=C1CC[C@H]2O[C@@]2(CC[C@@H]2[C@@H]1CC2(C)C)C | 1742210 |
| 222 | IMPHY012739 | (Z)-beta-Ocimene | C=C/C(=CCC=C(C)C)/C | 5320250 |
| 223 | IMPHY012921 | gamma-Elemene | C=C[C@]1(C)CCC(=C(C)C)C[C@H]1C(=C)C | 6432312 |
| 224 | IMPHY013093 | delta-Elemene | C=C[C@@]1(C)CCC(=C[C@@H]1C(=C)C)C(C)C | 12309449 |
| 225 | IMPHY014811 | alpha-Phellandrene | CC1=CCC(C=C1)C(C)C | 7460 |
| 226 | IMPHY014831 | beta-Caryophyllene | C/C/1=CCCC(=C)[C@@H]2[C@@H](CC1)C(C2)(C)C | 5281515 |
| 227 | IMPHY014835 | (E)-beta-ocimene | C=C/C(=C/CC=C(C)C)/C | 5281553 |
| 228 | IMPHY014847 | Bornyl acetate | CC(=O)OC1CC2C(C1(C)CC2)(C)C | 6448 |
| 229 | IMPHY014852 | Camphene | C=C1C2CCC(C1(C)C)C2 | 6616 |
| 230 | IMPHY014881 | Copaene | CC(C1CCC2(C3C1C2C(=CC3)C)C)C | 19725 |
| 231 | IMPHY012667 | Caryophyllene oxide | C=C1CC[C@H]2O[C@@]2(CC[C@@H]2[C@@H]1CC2(C)C)C | 1742210 |
| 232 | IMPHY012739 | (Z)-beta-Ocimene | C=C/C(=CCC=C(C)C)/C | 5320250 |
| 233 | IMPHY012921 | gamma-Elemene | C=C[C@]1(C)CCC(=C(C)C)C[C@H]1C(=C)C | 6432312 |
| 234 | IMPHY013093 | delta-Elemene | C=C[C@@]1(C)CCC(=C[C@@H]1C(=C)C)C(C)C | 12309449 |
| 235 | IMPHY014811 | alpha-Phellandrene | CC1=CCC(C=C1)C(C)C | 7460 |
| 236 | IMPHY014831 | beta-Caryophyllene | C/C/1=CCCC(=C)[C@@H]2[C@@H](CC1)C(C2)(C)C | 5281515 |
| 237 | IMPHY014835 | (E)-beta-ocimene | C=C/C(=C/CC=C(C)C)/C | 5281553 |
| 238 | IMPHY014847 | Bornyl acetate | CC(=O)OC1CC2C(C1(C)CC2)(C)C | 6448 |
| 239 | IMPHY014852 | Camphene | C=C1C2CCC(C1(C)C)C2 | 6616 |
| 240 | IMPHY014881 | Copaene | CC(C1CCC2(C3C1C2C(=CC3)C)C)C | 19725 |
| 241 | IMPHY014988 | Limonene | CC1=CCC(CC1)C(=C)C | 22311 |
| 242 | IMPHY015022 | Nerolidol | C=CC(CC/C=C(/CCC=C(C)C)C)(O)C | 5284507 |
| 243 | IMPHY000399 | beta-Bisabolene | CC(=CCCC(=C)[C@H]1CCC(=CC1)C)C | 10104370 |
| 244 | IMPHY001313 | Piperundecalidine | O=C(N1CCCCC1)/C=C/C=C/CCCC/C=C/c1ccc2c(c1)OCO2 | 44453654 |
| 245 | IMPHY003328 | Cepharadione B | COc1cc2C(=O)C(=O)N(c3c2c(c1OC)c1ccccc1c3)C | 189151 |
| 246 | IMPHY003329 | Norcepharadione B | COc1cc2C(=O)C(=O)Nc3c2c(c1OC)c1ccccc1c3 | 189168 |
| 247 | IMPHY004192 | Piperine | O=C(N1CCCCC1)/C=C/C=C/c1ccc2c(c1)OCO2 | 638024 |
| 248 | IMPHY004217 | Chavicine | O=C(N1CCCCC1)/C=CC=C/c1ccc2c(c1)OCO2 | 1548912 |
| 249 | IMPHY004341 | Piperlonguminine | CC(CNC(=O)/C=C/C=C/c1ccc2c(c1)OCO2)C | 5320621 |
| 250 | IMPHY005360 | Aristolactam BII | COc1c(OC)cc2c3c1c1ccccc1cc3NC2=O | 162739 |
| 251 | IMPHY005815 | Tetrahydropiperine | O=C(N1CCCCC1)CCCCc1ccc2c(c1)OCO2 | 581676 |
| 252 | IMPHY005944 | Pipercide | CC(CNC(=O)/C=C/C=C/CCCC/C=C/c1ccc2c(c1)OCO2)C | 5372162 |
| 253 | IMPHY006266 | Guineensine | CC(CNC(=O)/C=C/C=C/CCCCCC/C=C/c1ccc2c(c1)OCO2)C | 6442405 |
| 254 | IMPHY006342 | Piperlongumine | COc1cc(/C=C/C(=O)N2CCC=CC2=O)cc(c1OC)OC | 637858 |
| 255 | IMPHY006624 | Pluviatilol | COc1cc(ccc1O)[C@H]1OC[C@@H]2[C@H]1CO[C@H]2c1ccc2c(c1)OCO2 | 70695727 |
| 256 | IMPHY007269 | Trichostachine | O=C(N1CCCC1)/C=C/C=C/c1ccc2c(c1)OCO2 | 636537 |
| 257 | IMPHY007371 | Cepharadione A | O=C1N(C)c2cc3ccccc3c3c2c(C1=O)cc1c3OCO1 | 94577 |
| 258 | IMPHY007559 | Pipernonaline | O=C(N1CCCCC1)/C=C/CCCC/C=C/c1ccc2c(c1)OCO2 | 9974595 |
| 259 | IMPHY009389 | Pentadecane | CCCCCCCCCCCCCCC | 12391 |
| 260 | IMPHY011593 | Pellitorine | CCCCC/C=C/C=C/C(=O)NCC(C)C | 5318516 |
| 261 | IMPHY012058 | Linalool | C=CC(CCC=C(C)C)(O)C | 6549 |
| 262 | IMPHY012165 | Sabinene | C=C1CCC2(C1C2)C(C)C | 18818 |
| 263 | IMPHY013823 | Bamipine | CN1CCC(CC1)N(c1ccccc1)Cc1ccccc1 | 72075 |
| 264 | IMPHY014811 | alpha-Phellandrene | CC1=CCC(C=C1)C(C)C | 7460 |
| 265 | IMPHY014831 | beta-Caryophyllene | C/C/1=CCCC(=C)[C@@H]2[C@@H](CC1)C(C2)(C)C | 5281515 |
| 266 | IMPHY014836 | beta-Sitosterol | CC[C@@H](C(C)C)CC[C@H]([C@H]1CC[C@@H]2[C@]1(C)CC[C@H]1[C@H]2CC=C2[C@]1(C)CC[C@@H](C2)O)C | 222284 |
| 267 | IMPHY014895 | 5-[3-(1,3-Benzodioxol-5-yl)-1,3,3a,4,6,6a-hexahydrofuro[3,4-c]furan-6-yl]-1,3-benzodioxole | C1Oc2c(O1)cc(cc2)C1OCC2C1COC2c1ccc2c(c1)OCO2 | 5204 |
| 268 | IMPHY014988 | Limonene | CC1=CCC(CC1)C(=C)C | 22311 |
| 269 | IMPHY015047 | Rutin | Oc1cc(O)c2c(c1)oc(c(c2=O)O[C@@H]1O[C@H](CO[C@@H]2O[C@@H](C)[C@@H]([C@H]([C@H]2O)O)O)[C@H]([C@@H]([C@H]1O)O)O)c1ccc(c(c1)O)O | 5280805 |
|  |  |  |  |  |
| **PLANT NAME : *Piper nigrum*** | | | | |
| **Serial No** | **IMPPAT Phytochemical Identifier** | **Phytochemical Name** | **SMILES** | **CID** |
| 1 | IMPHY000022 | Myrcenol | C=CC(=C)CCCC(O)(C)C | 10975 |
| 2 | IMPHY000027 | (E)-Piperolein A | O=C(N1CCCCC1)CCCC/C=C/c1ccc2c(c1)OCO2 | 11141599 |
| 3 | IMPHY000099 | Myrtenol | OCC1=CCC2CC1C2(C)C | 10582 |
| 4 | IMPHY000399 | beta-Bisabolene | CC(=CCCC(=C)[C@H]1CCC(=CC1)C)C | 10104370 |
| 5 | IMPHY000402 | 1,4-Cineole | CC(C12CCC(O2)(CC1)C)C | 10106 |
| 6 | IMPHY000491 | Pinene | CC1CCC2CC1C2(C)C |  |
| 7 | IMPHY000602 | M-Cymene | Cc1cccc(c1)C(C)C | 10812 |
| 8 | IMPHY000711 | Bornylene | CC1(C)C2CCC1(C)C=C2 | 10047 |
| 9 | IMPHY001316 | (2E,4E,6E)-7-(2H-1,3-benzodioxol-5-yl)-1-(piperidin-1-yl)hepta-2,4,6-trien-1-one | O=C(N1CCCCC1)/C=C/C=C/C=C/c1ccc2c(c1)OCO2 | 10244953 |
| 10 | IMPHY001351 | Elemicin | C=CCc1cc(OC)c(c(c1)OC)OC | 10248 |
| 11 | IMPHY002263 | Pipericide | CC(NC(=O)/C=C/C=C/CCCC/C=C/c1ccc2c(c1)OCO2)C | 101422868 |
| 12 | IMPHY002825 | 2-(4-Methylphenyl)propan-2-ol | Cc1ccc(cc1)C(O)(C)C | 14529 |
| 13 | IMPHY002915 | Benzyl Alcohol | OCc1ccccc1 | 244 |
| 14 | IMPHY003398 | Myristicin | C=CCc1cc(OC)c2c(c1)OCO2 | 4276 |
| 15 | IMPHY003485 | Myrcene | C=CC(=C)CCC=C(C)C | 31253 |
| 16 | IMPHY003513 | Isoamyl alcohol | OCCC(C)C | 31260 |
| 17 | IMPHY003525 | Nonanal | CCCCCCCCC=O | 31289 |
| 18 | IMPHY003536 | Eugenol | C=CCc1ccc(c(c1)OC)O | 3314 |
| 19 | IMPHY003616 | Bicyclogermacrene | C/C/1=CCC/C(=C/[C@H]2[C@@H](CC1)C2(C)C)/C | 13894537 |
| 20 | IMPHY003694 | Germacrene a | C/C/1=CCC/C(=C/C[C@@H](CC1)C(=C)C)/C | 9548705 |
| 21 | IMPHY003719 | beta-Copaene | CC([C@@H]1CC[C@]2([C@@H]3[C@H]1C2C(=C)CC3)C)C | 57339298 |
| 22 | IMPHY003798 | (Z)-alpha-Bisabolene | CC(=CC/C=C(C1CCC(=CC1)C)/C)C | 5352653 |
| 23 | IMPHY003807 | (E)-alpha-bisabolene | CC(=CC/C=C(/C1CCC(=CC1)C)C)C | 5315468 |
| 24 | IMPHY003955 | (R)-4-Isopropylcyclohex-2-enone | CC([C@H]1CCC(=O)C=C1)C | 642520 |
| 25 | IMPHY003956 | (+)-gamma-Gurjunene | C[C@@H]1CC[C@H]2C1=C[C@@H](CC[C@H]2C)C(=C)C | 15560285 |
| 26 | IMPHY003962 | beta-Guaiene | CC(=C1CC[C@@H](C2=C(C1)[C@@H](C)CC2)C)C | 15560252 |
| 27 | IMPHY003977 | (-)-beta-Bourbonene | CC([C@@H]1CC[C@@]2([C@H]1[C@H]1C(=C)CC[C@@H]21)C)C | 62566 |
| 28 | IMPHY003982 | gamma-Terpinene | CC1=CCC(=CC1)C(C)C | 7461 |
| 29 | IMPHY004022 | Tricyclo(6.3.1.02,5)dodecan-1-ol, 4,4,8-trimethyl-, (1R,2S,5R,8S) | C[C@]12CCC[C@](C2)(O)[C@@H]2[C@@H](CC1)C(C2)(C)C | 11746218 |
| 30 | IMPHY004135 | Cyclohexene, 6-ethenyl-6-methyl-1-(1-methylethyl)-3-(1-methylethylidene)-, (S)- | C=C[C@@]1(C)CCC(=C(C)C)C=C1C(C)C | 11019992 |
| 31 | IMPHY004151 | Geranyl formate | O=COC/C=C(/CCC=C(C)C)C | 5282109 |
| 32 | IMPHY004184 | Cinnamyl acetate | CC(=O)OC/C=C/c1ccccc1 | 5282110 |
| 33 | IMPHY004192 | Piperine | O=C(N1CCCCC1)/C=C/C=C/c1ccc2c(c1)OCO2 | 638024 |
| 34 | IMPHY004217 | Chavicine | O=C(N1CCCCC1)/C=CC=C/c1ccc2c(c1)OCO2 | 1548912 |
| 35 | IMPHY004536 | Geranic acid | CC(=CCC/C(=C/C(=O)O)/C)C | 5275520 |
| 36 | IMPHY004646 | Vomifoliol | C[C@H](/C=C/[C@@]1(O)C(=CC(=O)CC1(C)C)C)O | 5280462 |
| 37 | IMPHY004889 | Cinnamyl alcohol | OC/C=C/c1ccccc1 | 5315892 |
| 38 | IMPHY005345 | 1-Octen-3-OL | CCCCCC(C=C)O | 18827 |
| 39 | IMPHY005653 | 2-Octanol | CCCCCCC(O)C | 20083 |
| 40 | IMPHY005720 | Piperolein B | O=C(N1CCCCC1)CCCCCC/C=C/c1ccc2c(c1)OCO2 | 21580213 |
| 41 | IMPHY005836 | Methyl caffeic acid | OC(=O)/C(=C/c1ccc(c(c1)O)O)/C | 55255958 |
| 42 | IMPHY005944 | Pipercide | CC(CNC(=O)/C=C/C=C/CCCC/C=C/c1ccc2c(c1)OCO2)C | 5372162 |
| 43 | IMPHY006007 | Citronellyl formate | O=COCCC(CCC=C(C)C)C | 7778 |
| 44 | IMPHY006145 | p-Cymene | Cc1ccc(cc1)C(C)C | 7463 |
| 45 | IMPHY006266 | Guineensine | CC(CNC(=O)/C=C/C=C/CCCCCC/C=C/c1ccc2c(c1)OCO2)C | 6442405 |
| 46 | IMPHY006324 | Linalyl propionate | CCC(=O)OC(CCC=C(C)C)(C=C)C | 61098 |
| 47 | IMPHY006414 | Geranyl tiglate | C/C=C(/C(=O)OC/C=C(/CCC=C(C)C)C)C | 5367785 |
| 48 | IMPHY006516 | 3,4-Methylenedioxycinnamic acid | OC(=O)/C=C/c1ccc2c(c1)OCO2 | 643181 |
| 49 | IMPHY006550 | Thymol | Cc1ccc(c(c1)O)C(C)C | 6989 |
| 50 | IMPHY006696 | Methyleugenol | C=CCc1ccc(c(c1)OC)OC | 7127 |
| 51 | IMPHY006719 | (1aR,7S,7aS,7bR)-1,1,4,7-tetramethyl-2,3,5,6,7a,7b-hexahydro-1aH-cyclopropa[h]azulen-7-ol | CC1=C2CC[C@]([C@H]2[C@H]2[C@@H](CC1)C2(C)C)(C)O | 102303030 |
| 52 | IMPHY006932 | Cuparene | Cc1ccc(cc1)[C@]1(C)CCCC1(C)C | 86895 |
| 53 | IMPHY006944 | Estragole | COc1ccc(cc1)CC=C | 8815 |
| 54 | IMPHY006948 | beta-Terpineol | CC(=C)C1CCC(CC1)(C)O | 8748 |
| 55 | IMPHY006965 | alpha,alpha-Dimethyl-4-methylenecyclohexanemethanol | CC(C1CCC(=C)CC1)(O)C | 81722 |
| 56 | IMPHY007055 | Piperonal | O=Cc1ccc2c(c1)OCO2 | 8438 |
| 57 | IMPHY007171 | 1-Hexanol | CCCCCCO | 8103 |
| 58 | IMPHY007188 | Piperidine | C1CCCNC1 | 8082 |
| 59 | IMPHY007202 | Nonane | CCCCCCCCC | 8141 |
| 60 | IMPHY007269 | Trichostachine | O=C(N1CCCC1)/C=C/C=C/c1ccc2c(c1)OCO2 | 636537 |
| 61 | IMPHY007307 | (1S,2S,3S,6S)-3-ethenyl-3,7,7-trimethyl-2-prop-1-en-2-ylbicyclo[4.1.0]heptane | C=C[C@]1(C)CC[C@H]2[C@@H]([C@H]1C(=C)C)C2(C)C | 91746561 |
| 62 | IMPHY007327 | Palmitic acid | CCCCCCCCCCCCCCCC(=O)O | 985 |
| 63 | IMPHY007376 | beta-Cubebene | CC([C@@H]1CC[C@H]([C@]23[C@H]1[C@H]2C(=C)CC3)C)C | 93081 |
| 64 | IMPHY007421 | Citronellyl acetate | CC(CCC=C(C)C)CCOC(=O)C | 9017 |
| 65 | IMPHY007445 | (Z)-beta-Ocimenol | C=C/C(=CC(C=C(C)C)O)/C | 91753567 |
| 66 | IMPHY007528 | Cadinane | C[C@H]1CC[C@@H]2[C@@H](C1)[C@@H](CC[C@@H]2C)C(C)C | 9548708 |
| 67 | IMPHY007840 | Spathulenol | C=C1CC[C@@H]2[C@H]([C@H]3[C@H]1CC[C@]3(C)O)C2(C)C | 92231 |
| 68 | IMPHY007852 | Azulene | c1ccc2-c(cc1)ccc2 | 9231 |
| 69 | IMPHY008146 | 1-Pentanol | CCCCCO | 6276 |
| 70 | IMPHY008303 | 1-Hepten-3-OL | CCCCC(C=C)O | 21057 |
| 71 | IMPHY008773 | (2E,4E)-N-(2-methylpropyl)octadeca-2,4-dienamide | CCCCCCCCCCCCC/C=C/C=C/C(=O)NCC(C)C | 9974234 |
| 72 | IMPHY008936 | alpha-Guaiene | CC(=C)[C@@H]1CC[C@@H](C2=C(C1)[C@@H](C)CC2)C | 5317844 |
| 73 | IMPHY008946 | delta-Guaiene | CC(=C)[C@@H]1CCC(=C2[C@@H](C1)[C@@H](C)CC2)C | 94275 |
| 74 | IMPHY009604 | Dihydroferuperine | COC1=CC(=CCC1O)/C=CC=CC(=O)N1CCCCC1 | 131752910 |
| 75 | IMPHY009605 | Feruperine | COc1cc(/C=CC=CC(=O)N2CCCCC2)ccc1O | 131752909 |
| 76 | IMPHY009618 | Zingiberenol | CC(=CCCC(C1CCC(C=C1)(C)O)C)C | 13213649 |
| 77 | IMPHY009626 | 2-Nonanol | CCCCCCCC(O)C | 12367 |
| 78 | IMPHY009840 | Cyclosativene | CC(C1CCC2(C3C1C1C(C21C)C3)C)C | 519960 |
| 79 | IMPHY009853 | Naphthalene | c1ccc2c(c1)cccc2 | 931 |
| 80 | IMPHY009946 | Benzaldehyde | O=Cc1ccccc1 | 240 |
| 81 | IMPHY009966 | Eucarvone | CC1=CC=CC(CC1=O)(C)C | 136330 |
| 82 | IMPHY010072 | Eucalyptol | CC12CCC(CC1)C(O2)(C)C | 2758 |
| 83 | IMPHY010080 | beta-Elemene | C=C[C@]1(C)CC[C@H](C[C@H]1C(=C)C)C(=C)C | 6918391 |
| 84 | IMPHY010097 | Benzyl benzoate | O=C(c1ccccc1)OCc1ccccc1 | 2345 |
| 85 | IMPHY010603 | beta-Cadinene | CC1=CC[C@@H]2[C@@H](C1)[C@@H](CC=C2C)C(C)C | 10657 |
| 86 | IMPHY010986 | 2-Methyl cinnamyl alcohol | OCC=Cc1ccccc1C | 54031279 |
| 87 | IMPHY011050 | 3-Buten-2-OL | CC(C=C)O | 11716 |
| 88 | IMPHY011058 | 3-Cyclohexen-1-ol, 4-methyl-1-(1-methylethyl)-, acetate | CC(=O)OC1(CCC(=CC1)C)C(C)C | 20960 |
| 89 | IMPHY011392 | 3-Carene | CC1=CCC2C(C1)C2(C)C | 26049 |
| 90 | IMPHY011396 | 4-Carvomenthenol | CC1=CCC(CC1)(O)C(C)C | 11230 |
| 91 | IMPHY011409 | Guaiacol | COc1ccccc1O | 460 |
| 92 | IMPHY011519 | alpha-Terpinyl acetate | CC(=O)OC(C1CCC(=CC1)C)(C)C | 111037 |
| 93 | IMPHY011542 | beta-Eudesmol | C=C1CCC[C@]2([C@H]1C[C@@H](CC2)C(O)(C)C)C | 91457 |
| 94 | IMPHY011552 | (1R)-2-methyl-5-propan-2-ylbicyclo[3.1.0]hex-2-ene | CC1=CCC2([C@@H]1C2)C(C)C | 6451618 |
| 95 | IMPHY011557 | 4-Isopropylbenzyl alcohol | OCc1ccc(cc1)C(C)C | 325 |
| 96 | IMPHY011562 | 2-Hexenal | CCC/C=C/C=O | 5281168 |
| 97 | IMPHY011581 | alpha-Selinene | CC1=CCC[C@]2([C@H]1C[C@@H](CC2)C(=C)C)C | 10856614 |
| 98 | IMPHY011586 | (1R)-2-methyl-5-propan-2-ylbicyclo[3.1.0]hex-2-ene | C/C/1=C/CCC(=C)/C=C[C@@H](CC1)C(C)C | 91723653 |
| 99 | IMPHY011588 | cis-3-Hexen-1-ol | OCC/C=CCC | 5281167 |
| 100 | IMPHY011590 | d-Borneol | O[C@@H]1C[C@H]2C([C@@]1(C)CC2)(C)C | 61060 |
| 101 | IMPHY011593 | Pellitorine | CCCCC/C=C/C=C/C(=O)NCC(C)C | 5318516 |
| 102 | IMPHY011599 | Terpinolene | CC1=CCC(=C(C)C)CC1 | 11463 |
| 103 | IMPHY011630 | cis,cis-Farnesol | OC/C=C(CC/C=C(CCC=C(C)C)/C)/C | 1549107 |
| 104 | IMPHY011631 | (E,Z)-farnesol | OC/C=C(/CC/C=C(CCC=C(C)C)/C)C | 1549109 |
| 105 | IMPHY011632 | Farnesol | OC/C=C(/CC/C=C(/CCC=C(C)C)C)C | 445070 |
| 106 | IMPHY011633 | (2Z,6E)-Farnesol | OC/C=C(CC/C=C(/CCC=C(C)C)C)/C | 1549108 |
| 107 | IMPHY011643 | alpha-Terpinene | CC1=CC=C(CC1)C(C)C | 7462 |
| 108 | IMPHY011647 | Geranyl acetate | C/C(=CCOC(=O)C)/CCC=C(C)C | 1549026 |
| 109 | IMPHY011648 | Neryl acetate | C/C(=C/COC(=O)C)/CCC=C(C)C | 1549025 |
| 110 | IMPHY011658 | beta-Farnesene | C=CC(=C)CC/C=C(/CCC=C(C)C)C | 5281517 |
| 111 | IMPHY011659 | alpha-Muurolene | CC1=C[C@@H]2[C@H](CC1)C(=CC[C@H]2C(C)C)C | 12306047 |
| 112 | IMPHY011667 | alpha-Gurjunene | C[C@@H]1CC[C@@H]2[C@H](C3=C(CC[C@H]13)C)C2(C)C | 15560276 |
| 113 | IMPHY011709 | alpha-Eudesmol | CC1=CCC[C@]2([C@H]1C[C@@H](CC2)C(O)(C)C)C | 92762 |
| 114 | IMPHY011745 | Zingiberene | CC(=CCC[C@@H]([C@H]1CC=C(C=C1)C)C)C | 92776 |
| 115 | IMPHY011749 | Humulene epoxide II | C/C/1=CCC(C)(C)/C=C/C[C@@]2([C@@H](CC1)O2)C | 10704181 |
| 116 | IMPHY011761 | Humulene | C/C/1=CCC(C)(C)/C=C/C/C(=C/CC1)/C | 5281520 |
| 117 | IMPHY011789 | Citral | O=C/C=C(/CCC=C(C)C)C | 638011 |
| 118 | IMPHY011790 | Neral | O=C/C=C(CCC=C(C)C)/C | 643779 |
| 119 | IMPHY011792 | gamma-Muurolene | CC1=C[C@@H]2[C@H](CC1)C(=C)CC[C@H]2C(C)C | 12313020 |
| 120 | IMPHY011793 | (+)-gamma-Cadinene | CC1=C[C@@H]2[C@@H](CC1)C(=C)CC[C@H]2C(C)C | 6432404 |
| 121 | IMPHY011817 | alpha-Farnesene | C=C/C(=C/C/C=C(/CCC=C(C)C)C)/C | 5281516 |
| 122 | IMPHY011872 | (4E,7E)-1,5,9,9-Tetramethyl-12-oxabicyclo[9.1.0]dodeca-4,7-diene | C/C/1=C/CCC2(C)OC2CC(/C=CC1)(C)C | 22559443 |
| 123 | IMPHY011879 | Moupinamide | COc1cc(/C=C/C(=O)NCCc2ccc(cc2)O)ccc1O | 5280537 |
| 124 | IMPHY011882 | Cinnamaldehyde | O=C/C=C/c1ccccc1 | 637511 |
| 125 | IMPHY011890 | Elemol | C=C[C@]1(C)CC[C@H](C[C@H]1C(=C)C)C(O)(C)C | 92138 |
| 126 | IMPHY011901 | Thujone | O=C1C[C@]2([C@@H]([C@H]1C)C2)C(C)C | 261491 |
| 127 | IMPHY011902 | beta-Thujone | O=C1C[C@]2([C@@H]([C@@H]1C)C2)C(C)C | 91456 |
| 128 | IMPHY011938 | gamma-Eudesmol | CC1=C2C[C@@H](CC[C@]2(CCC1)C)C(O)(C)C | 6432005 |
| 129 | IMPHY011939 | 10-epi-gamma-Eudesmol | CC1=C2C[C@@H](CC[C@@]2(CCC1)C)C(O)(C)C | 6430754 |
| 130 | IMPHY011957 | (+)-delta-Cadinene | CC1=C[C@@H]2C(=C(C)CC[C@H]2C(C)C)CC1 | 441005 |
| 131 | IMPHY011965 | (+)-beta-Phellandrene | CC([C@@H]1CCC(=C)C=C1)C | 442484 |
| 132 | IMPHY011973 | (-)-cis-Carveol | CC(=C)[C@@H]1CC=C([C@@H](C1)O)C | 330573 |
| 133 | IMPHY011983 | cis-Cinnamaldehyde | O=C/C=Cc1ccccc1 | 6428995 |
| 134 | IMPHY011988 | (-)-trans-Carveol | CC(=C)[C@@H]1CC=C([C@H](C1)O)C | 94221 |
| 135 | IMPHY012036 | Camphor | O=C1CC2C(C1(C)CC2)(C)C | 2537 |
| 136 | IMPHY012058 | Linalool | C=CC(CCC=C(C)C)(O)C | 6549 |
| 137 | IMPHY012061 | alpha-Pinene | CC1=CCC2CC1C2(C)C | 6654 |
| 138 | IMPHY012086 | Citronellal | O=CCC(CCC=C(C)C)C | 7794 |
| 139 | IMPHY012095 | Cyclohexene | C1CCC=CC1 | 8079 |
| 140 | IMPHY012104 | Citronellol | OCCC(CCC=C(C)C)C | 8842 |
| 141 | IMPHY012107 | Adamantane | C1C2CC3CC1CC(C2)C3 | 9238 |
| 142 | IMPHY012130 | Dihydrocarveol | CC(=C)C1CCC(C(C1)O)C | 12072 |
| 143 | IMPHY012147 | beta-Pinene | C=C1CCC2CC1C2(C)C | 14896 |
| 144 | IMPHY012160 | alpha-Terpineol | CC1=CCC(CC1)C(O)(C)C | 17100 |
| 145 | IMPHY012165 | Sabinene | C=C1CCC2(C1C2)C(C)C | 18818 |
| 146 | IMPHY012168 | (1S,2S,6S,7R,8R)-1,3-dimethyl-8-propan-2-yltricyclo[4.4.0.02,7]dec-3-ene | CC([C@H]1CC[C@]2([C@@H]3[C@@H]1[C@H]2C(=CC3)C)C)C | 101607926 |
| 147 | IMPHY012178 | p-Menthan-3-one | CC1CCC(C(=O)C1)C(C)C | 6986 |
| 148 | IMPHY012279 | alpha-Curcumene | CC(=CCCC(c1ccc(cc1)C)C)C | 92139 |
| 149 | IMPHY012305 | beta-Patchoulene | CC1CCC2=C1CC1CCC2(C1(C)C)C | 101731 |
| 150 | IMPHY012585 | delta-Cadinol | CC1=C[C@@H]2[C@H](CC1)[C@](C)(O)CC[C@H]2C(C)C | 3084311 |
| 151 | IMPHY012586 | (-)-alpha-Cadinol | CC1=CC2C(CC1)[C@@](C)(O)CC[C@@H]2C(C)C | 6431302 |
| 152 | IMPHY012589 | 3-(1,5-Dimethyl-4-hexenyl)-6-methylene-1-cyclohexene | CC(C1CCC(=C)C=C1)CCC=C(C)C | 519764 |
| 153 | IMPHY012654 | Nerol | OC/C=C(CCC=C(C)C)/C | 643820 |
| 154 | IMPHY012667 | Caryophyllene oxide | C=C1CC[C@H]2O[C@@]2(CC[C@@H]2[C@@H]1CC2(C)C)C | 1742210 |
| 155 | IMPHY012739 | (Z)-beta-Ocimene | C=C/C(=CCC=C(C)C)/C | 5320250 |
| 156 | IMPHY012823 | 1,6-Cyclodecadiene | C1C/C=CCCC/C=CC1 | 5365639 |
| 157 | IMPHY012920 | 2-Furanmethanol, 5-ethenyltetrahydro-alpha,alpha,5-trimethyl-, cis- | C=C[C@@]1(C)CC[C@H](O1)C(O)(C)C | 11116492 |
| 158 | IMPHY012921 | gamma-Elemene | C=C[C@]1(C)CCC(=C(C)C)C[C@H]1C(=C)C | 6432312 |
| 159 | IMPHY013093 | delta-Elemene | C=C[C@@]1(C)CCC(=C[C@@H]1C(=C)C)C(C)C | 12309449 |
| 160 | IMPHY013133 | (Z)-p-Menth-2-en-1-ol | CC([C@@H]1CC[C@](C=C1)(C)O)C | 13918681 |
| 161 | IMPHY014161 | Coumaperine | Oc1ccc(cc1)/C=C/C=C/C(=O)N1CCCCC1 | 10131321 |
| 162 | IMPHY014708 | beta-Selinene | C=C1CCC[C@]2([C@H]1C[C@@H](CC2)C(=C)C)C | 442393 |
| 163 | IMPHY014801 | Zizanene | CC1=C[C@@H]2[C@H](CC1)C(=CC[C@@H]2C(C)C)C | 12306046 |
| 164 | IMPHY014806 | Caswell No. 264AB | CC([C@@H]1CC[C@H]([C@]23[C@H]1[C@H]2C(=CC3)C)C)C | 442359 |
| 165 | IMPHY014811 | alpha-Phellandrene | CC1=CCC(C=C1)C(C)C | 7460 |
| 166 | IMPHY014817 | Aromadendrene | CC1CCC2C1C1C(C1(C)C)CCC2=C | 91354 |
| 167 | IMPHY014831 | beta-Caryophyllene | C/C/1=CCCC(=C)[C@@H]2[C@@H](CC1)C(C2)(C)C | 5281515 |
| 168 | IMPHY014835 | (E)-beta-ocimene | C=C/C(=C/CC=C(C)C)/C | 5281553 |
| 169 | IMPHY014852 | Camphene | C=C1C2CCC(C1(C)C)C2 | 6616 |
| 170 | IMPHY014857 | Cedrol | C[C@@H]1CC[C@@H]2[C@@]31CC[C@@]([C@H](C3)C2(C)C)(C)O | 65575 |
| 171 | IMPHY014871 | cis-Nerolidol | C=CC(CC/C=C(CCC=C(C)C)/C)(O)C | 5320128 |
| 172 | IMPHY014881 | Copaene | CC(C1CCC2(C3C1C2C(=CC3)C)C)C | 19725 |
| 173 | IMPHY014884 | Cubebene | CC([C@H]1CC[C@@H](C23[C@@H]1C2C(=CC3)C)C)C | 91747196 |
| 174 | IMPHY014906 | Cedrelanol | CC1=C[C@@H]2[C@@H](CC1)[C@@](C)(O)CC[C@H]2C(C)C | 160799 |
| 175 | IMPHY014907 | 6-Epi-beta-bisabolol | CC(=CCC[C@@H]([C@@]1(O)CCC(=CC1)C)C)C | 12300148 |
| 176 | IMPHY014923 | Geraniol | OC/C=C(/CCC=C(C)C)C | 637566 |
| 177 | IMPHY014988 | Limonene | CC1=CCC(CC1)C(=C)C | 22311 |
| 178 | IMPHY014989 | trans-Linalool oxide | C=C[C@]1(C)CC[C@H](O1)C(O)(C)C | 6432254 |
| 179 | IMPHY015003 | Menthol | CC1CCC(C(C1)O)C(C)C | 1254 |
| 180 | IMPHY015004 | Menthone | C[C@@H]1CC[C@H](C(=O)C1)C(C)C | 26447 |
| 181 | IMPHY015016 | alpha-Muurolol | CC1=C[C@@H]2[C@H](CC1)[C@](C)(O)CC[C@@H]2C(C)C | 91753440 |
| 182 | IMPHY015022 | Nerolidol | C=CC(CC/C=C(/CCC=C(C)C)C)(O)C | 5284507 |
| 183 | IMPHY015042 | Piperitone | CC1=CC(=O)C(CC1)C(C)C | 6987 |
| 184 | IMPHY015094 | (+)-trans-Limonene oxide | CC(=C)[C@@H]1CC[C@]2([C@@H](C1)O2)C | 449290 |
| 185 | IMPHY015095 | 2-Cyclohexen-1-ol, 1-methyl-4-(1-methylethyl)-, trans- | CC([C@@H]1CC[C@@](C=C1)(C)O)C | 122484 |
| 186 | IMPHY015123 | alpha-Copaene | CC([C@@H]1CC[C@]2([C@@H]3[C@H]1C2C(=CC3)C)C)C | 70678558 |
| 187 | IMPHY016027 | trans-Sabinene hydrate | CC([C@@]12CC[C@](C2C1)(C)O)C | 12315151 |
| 188 | IMPHY016054 | trans-alpha-Bergamotene | CC(=CCC[C@]1(C)[C@H]2CC=C([C@@H]1C2)C)C | 6429302 |
| 189 | IMPHY000022 | Myrcenol | C=CC(=C)CCCC(O)(C)C | 10975 |
| 190 | IMPHY000399 | beta-Bisabolene | CC(=CCCC(=C)[C@H]1CCC(=CC1)C)C | 10104370 |
| 191 | IMPHY000402 | 1,4-Cineole | CC(C12CCC(O2)(CC1)C)C | 10106 |
| 192 | IMPHY001246 | Carvacrol | CC(c1ccc(c(c1)O)C)C | 10364 |
| 193 | IMPHY001351 | Elemicin | C=CCc1cc(OC)c(c(c1)OC)OC | 10248 |
| 194 | IMPHY001516 | Decane | CCCCCCCCCC | 15600 |
| 195 | IMPHY001912 | Cubebin | O[C@H]1OC[C@@H]([C@H]1Cc1ccc2c(c1)OCO2)Cc1ccc2c(c1)OCO2 | 117443 |
| 196 | IMPHY003296 | Piperitenone | CC1=CC(=O)C(=C(C)C)CC1 | 381152 |
| 197 | IMPHY003485 | Myrcene | C=CC(=C)CCC=C(C)C | 31253 |
| 198 | IMPHY003719 | beta-Copaene | CC([C@@H]1CC[C@]2([C@@H]3[C@H]1C2C(=C)CC3)C)C | 57339298 |
| 199 | IMPHY003822 | Cubebol | CC([C@@H]1CC[C@H]([C@]23[C@H]1[C@H]2[C@@](C)(O)CC3)C)C | 11276107 |
| 200 | IMPHY003977 | (-)-beta-Bourbonene | CC([C@@H]1CC[C@@]2([C@H]1[C@H]1C(=C)CC[C@@H]21)C)C | 62566 |
| 201 | IMPHY003982 | gamma-Terpinene | CC1=CCC(=CC1)C(C)C | 7461 |
| 202 | IMPHY004067 | Dihydrocarvyl acetate | CC(=O)OC1CC(CCC1C)C(=C)C | 30248 |
| 203 | IMPHY004281 | Guaiol | C[C@H]1CC[C@H](CC2=C1CC[C@@H]2C)C(O)(C)C | 227829 |
| 204 | IMPHY004549 | Safrole | C=CCc1ccc2c(c1)OCO2 | 5144 |
| 205 | IMPHY005521 | 1-Hexen-3-OL | CCCC(C=C)O | 20928 |
| 206 | IMPHY005618 | Germacrene B | C/C/1=CCC/C(=C/CC(=C(C)C)CC1)/C | 5281519 |
| 207 | IMPHY006145 | p-Cymene | Cc1ccc(cc1)C(C)C | 7463 |
| 208 | IMPHY006177 | Methyl geranate | COC(=O)/C=C(/CCC=C(C)C)C | 5365910 |
| 209 | IMPHY006550 | Thymol | Cc1ccc(c(c1)O)C(C)C | 6989 |
| 210 | IMPHY006696 | Methyleugenol | C=CCc1ccc(c(c1)OC)OC | 7127 |
| 211 | IMPHY006948 | beta-Terpineol | CC(=C)C1CCC(CC1)(C)O | 8748 |
| 212 | IMPHY007041 | Furfural | O=Cc1ccco1 | 7362 |
| 213 | IMPHY007376 | beta-Cubebene | CC([C@@H]1CC[C@H]([C@]23[C@H]1[C@H]2C(=C)CC3)C)C | 93081 |
| 214 | IMPHY007421 | Citronellyl acetate | CC(CCC=C(C)C)CCOC(=O)C | 9017 |
| 215 | IMPHY007840 | Spathulenol | C=C1CC[C@@H]2[C@H]([C@H]3[C@H]1CC[C@]3(C)O)C2(C)C | 92231 |
| 216 | IMPHY008150 | 1-Methyl-4-(prop-1-en-2-yl)benzene | Cc1ccc(cc1)C(=C)C | 62385 |
| 217 | IMPHY008303 | 1-Hepten-3-OL | CCCCC(C=C)O | 21057 |
| 218 | IMPHY008369 | Methyl isobutyl ketone | CC(CC(=O)C)C | 7909 |
| 219 | IMPHY008936 | alpha-Guaiene | CC(=C)[C@@H]1CC[C@@H](C2=C(C1)[C@@H](C)CC2)C | 5317844 |
| 220 | IMPHY008946 | delta-Guaiene | CC(=C)[C@@H]1CCC(=C2[C@@H](C1)[C@@H](C)CC2)C | 94275 |
| 221 | IMPHY009642 | 2-Nonanone | CCCCCCCC(=O)C | 13187 |
| 222 | IMPHY010080 | beta-Elemene | C=C[C@]1(C)CC[C@H](C[C@H]1C(=C)C)C(=C)C | 6918391 |
| 223 | IMPHY011002 | 3-Methyl-2-pentanol | CCC(C(O)C)C | 11261 |
| 224 | IMPHY011100 | alpha-Cedrene epoxide | CC1CCC2C31CC1OC1(C(C3)C2(C)C)C | 122510 |
| 225 | IMPHY011392 | 3-Carene | CC1=CCC2C(C1)C2(C)C | 26049 |
| 226 | IMPHY011396 | 4-Carvomenthenol | CC1=CCC(CC1)(O)C(C)C | 11230 |
| 227 | IMPHY011455 | Cadina-1,4-diene | CC1=CC2C(=CC1)[C@@H](C)CCC2C(C)C | 6427091 |
| 228 | IMPHY011519 | alpha-Terpinyl acetate | CC(=O)OC(C1CCC(=CC1)C)(C)C | 111037 |
| 229 | IMPHY011521 | 2-Undecanone | CCCCCCCCCC(=O)C | 8163 |
| 230 | IMPHY011542 | beta-Eudesmol | C=C1CCC[C@]2([C@H]1C[C@@H](CC2)C(O)(C)C)C | 91457 |
| 231 | IMPHY011552 | (1R)-2-methyl-5-propan-2-ylbicyclo[3.1.0]hex-2-ene | CC1=CCC2([C@@H]1C2)C(C)C | 6451618 |
| 232 | IMPHY011581 | alpha-Selinene | CC1=CCC[C@]2([C@H]1C[C@@H](CC2)C(=C)C)C | 10856614 |
| 233 | IMPHY011586 | (S,1Z,6Z)-8-Isopropyl-1-methyl-5-methylenecyclodeca-1,6-diene | C/C/1=C/CCC(=C)/C=C[C@@H](CC1)C(C)C | 91723653 |
| 234 | IMPHY011588 | cis-3-Hexen-1-ol | OCC/C=CCC | 5281167 |
| 235 | IMPHY011590 | d-Borneol | O[C@@H]1C[C@H]2C([C@@]1(C)CC2)(C)C | 61060 |
| 236 | IMPHY011599 | Terpinolene | CC1=CCC(=C(C)C)CC1 | 11463 |
| 237 | IMPHY011614 | beta-Cedrene | C=C1CC[C@@]23C[C@@H]1C(C)(C)[C@@H]2CC[C@H]3C | 11106485 |
| 238 | IMPHY011630 | cis,cis-Farnesol | OC/C=C(CC/C=C(CCC=C(C)C)/C)/C | 1549107 |
| 239 | IMPHY011631 | (E,Z)-farnesol | OC/C=C(/CC/C=C(CCC=C(C)C)/C)C | 1549109 |
| 240 | IMPHY011632 | Farnesol | OC/C=C(/CC/C=C(/CCC=C(C)C)C)C | 445070 |
| 241 | IMPHY011633 | (2Z,6E)-Farnesol | OC/C=C(CC/C=C(/CCC=C(C)C)C)/C | 1549108 |
| 242 | IMPHY011643 | alpha-Terpinene | CC1=CC=C(CC1)C(C)C | 7462 |
| 243 | IMPHY011659 | alpha-Muurolene | CC1=C[C@@H]2[C@H](CC1)C(=CC[C@H]2C(C)C)C | 12306047 |
| 244 | IMPHY011761 | Humulene | C/C/1=CCC(C)(C)/C=C/C/C(=C/CC1)/C | 5281520 |
| 245 | IMPHY011789 | Citral | O=C/C=C(/CCC=C(C)C)C | 638011 |
| 246 | IMPHY011790 | Neral | O=C/C=C(CCC=C(C)C)/C | 643779 |
| 247 | IMPHY011890 | Elemol | C=C[C@]1(C)CC[C@H](C[C@H]1C(=C)C)C(O)(C)C | 92138 |
| 248 | IMPHY011957 | (+)-delta-Cadinene | CC1=C[C@@H]2C(=C(C)CC[C@H]2C(C)C)CC1 | 441005 |
| 249 | IMPHY011965 | (+)-beta-Phellandrene | CC([C@@H]1CCC(=C)C=C1)C | 442484 |
| 250 | IMPHY011988 | (-)-trans-Carveol | CC(=C)[C@@H]1CC=C([C@H](C1)O)C | 94221 |
| 251 | IMPHY012036 | Camphor | O=C1CC2C(C1(C)CC2)(C)C | 2537 |
| 252 | IMPHY012058 | Linalool | C=CC(CCC=C(C)C)(O)C | 6549 |
| 253 | IMPHY012061 | alpha-Pinene | CC1=CCC2CC1C2(C)C | 6654 |
| 254 | IMPHY012086 | Citronellal | O=CCC(CCC=C(C)C)C | 7794 |
| 255 | IMPHY012104 | Citronellol | OCCC(CCC=C(C)C)C | 8842 |
| 256 | IMPHY012147 | beta-Pinene | C=C1CCC2CC1C2(C)C | 14896 |
| 257 | IMPHY012160 | alpha-Terpineol | CC1=CCC(CC1)C(O)(C)C | 17100 |
| 258 | IMPHY012165 | Sabinene | C=C1CCC2(C1C2)C(C)C | 18818 |
| 259 | IMPHY012168 | (1S,2S,6S,7R,8R)-1,3-dimethyl-8-propan-2-yltricyclo[4.4.0.02,7]dec-3-ene | CC([C@H]1CC[C@]2([C@@H]3[C@@H]1[C@H]2C(=CC3)C)C)C | 101607926 |
| 260 | IMPHY012255 | (+)-trans-Piperitenol | CC1=C[C@@H]([C@H](CC1)C(C)C)O | 85568 |
| 261 | IMPHY012261 | alpha-Bergamotene | CC(=CCCC1(C)C2CC=C(C1C2)C)C | 86608 |
| 262 | IMPHY012279 | alpha-Curcumene | CC(=CCCC(c1ccc(cc1)C)C)C | 92139 |
| 263 | IMPHY012586 | (-)-alpha-Cadinol | CC1=CC2C(CC1)[C@@](C)(O)CC[C@@H]2C(C)C | 6431302 |
| 264 | IMPHY012654 | Nerol | OC/C=C(CCC=C(C)C)/C | 643820 |
| 265 | IMPHY012667 | Caryophyllene oxide | C=C1CC[C@H]2O[C@@]2(CC[C@@H]2[C@@H]1CC2(C)C)C | 1742210 |
| 266 | IMPHY012910 | trans-Calamenene | CC([C@H]1CC[C@@H](c2c1cc(C)cc2)C)C | 6429022 |
| 267 | IMPHY013080 | alpha-Calacorene | CC([C@@H]1CC=C(c2c1cc(C)cc2)C)C | 12302243 |
| 268 | IMPHY013093 | delta-Elemene | C=C[C@@]1(C)CCC(=C[C@@H]1C(=C)C)C(C)C | 12309449 |
| 269 | IMPHY013836 | Fenchone | O=C1C2(C)CCC(C1(C)C)C2 | 14525 |
| 270 | IMPHY013838 | 3,7-Dimethyloct-6-en-3-ol | CCC(CCC=C(C)C)(O)C | 86749 |
| 271 | IMPHY014690 | (-)-Globulol | C[C@@H]1CC[C@@H]2[C@@H]1[C@H]1[C@H](C1(C)C)CC[C@@]2(C)O | 12304985 |
| 272 | IMPHY014708 | beta-Selinene | C=C1CCC[C@]2([C@H]1C[C@@H](CC2)C(=C)C)C | 442393 |
| 273 | IMPHY014805 | Cedr-8-ene | CC1=CC[C@@]23C[C@@H]1C(C)(C)[C@@H]2CC[C@H]3C | 6431015 |
| 274 | IMPHY014806 | Caswell No. 264AB | CC([C@@H]1CC[C@H]([C@]23[C@H]1[C@H]2C(=CC3)C)C)C | 442359 |
| 275 | IMPHY014811 | alpha-Phellandrene | CC1=CCC(C=C1)C(C)C | 7460 |
| 276 | IMPHY014831 | beta-Caryophyllene | C/C/1=CCCC(=C)[C@@H]2[C@@H](CC1)C(C2)(C)C | 5281515 |
| 277 | IMPHY014835 | (E)-beta-ocimene | C=C/C(=C/CC=C(C)C)/C | 5281553 |
| 278 | IMPHY014847 | Bornyl acetate | CC(=O)OC1CC2C(C1(C)CC2)(C)C | 6448 |
| 279 | IMPHY014852 | Camphene | C=C1C2CCC(C1(C)C)C2 | 6616 |
| 280 | IMPHY014857 | Cedrol | C[C@@H]1CC[C@@H]2[C@@]31CC[C@@]([C@H](C3)C2(C)C)(C)O | 65575 |
| 281 | IMPHY014865 | Calamenene | CC([C@@H]1CC[C@@H](c2c1cc(C)cc2)C)C | 6429077 |
| 282 | IMPHY014874 | cis-Sabinene hydrate | C[C@@H]1CC[C@@]2(C1C2)C(C)C | 101629835 |
| 283 | IMPHY014907 | 6-Epi-beta-bisabolol | CC(=CCC[C@@H]([C@@]1(O)CCC(=CC1)C)C)C | 12300148 |
| 284 | IMPHY014914 | Fenchol | OC1C2(C)CCC(C1(C)C)C2 | 15406 |
| 285 | IMPHY014923 | Geraniol | OC/C=C(/CCC=C(C)C)C | 637566 |
| 286 | IMPHY014988 | Limonene | CC1=CCC(CC1)C(=C)C | 22311 |
| 287 | IMPHY014989 | trans-Linalool oxide | C=C[C@]1(C)CC[C@H](O1)C(O)(C)C | 6432254 |
| 288 | IMPHY015062 | (+)-cis-Sabinol | CC([C@]12C[C@@H]2C(=C)[C@@H](C1)O)C | 94147 |
| 289 | IMPHY015063 | Sabinyl acetate | CC(=O)OC1CC2(C(C1=C)C2)C(C)C | 94266 |
| 290 | IMPHY015123 | alpha-Copaene | CC([C@@H]1CC[C@]2([C@@H]3[C@H]1C2C(=CC3)C)C)C | 70678558 |
| 291 | IMPHY015128 | T-Muurolol | CC1=C[C@@H]2[C@H](CC1)[C@@](C)(O)CC[C@H]2C(C)C | 3084331 |
| 292 | IMPHY015380 | 4-Methyl-2-pentanol | CC(CC(O)C)C | 7910 |
| 293 | IMPHY016012 | Allo-Aromadendrene | C[C@@H]1CC[C@H]2[C@@H]1C1C(C1(C)C)CCC2=C | 42608158 |
| 294 | IMPHY017044 | Dihydroocimene | CCC(/C=C/C=C(C)C)C | 129640025 |
| 295 | IMPHY017124 | beta Farnesene | CCC(=C)CC/C=C(/CCC=C(C)C)C | 15228937 |
| 296 | IMPHY017645 | Cadina-1,4-diene-3-ol | CC(C1CCC(C2=CC(C(=CC12)C)O)C)C | 6431196 |
| 297 | IMPHY000491 | Pinene | CC1CCC2CC1C2(C)C |  |
| 298 | IMPHY002915 | Benzyl Alcohol | OCc1ccccc1 | 244 |
| 299 | IMPHY003694 | Germacrene a | C/C/1=CCC/C(=C/C[C@@H](CC1)C(=C)C)/C | 9548705 |
| 300 | IMPHY004135 | Cyclohexene, 6-ethenyl-6-methyl-1-(1-methylethyl)-3-(1-methylethylidene)-, (S)- | C=C[C@@]1(C)CCC(=C(C)C)C=C1C(C)C | 11019992 |
| 301 | IMPHY007840 | Spathulenol | C=C1CC[C@@H]2[C@H]([C@H]3[C@H]1CC[C@]3(C)O)C2(C)C | 92231 |
| 302 | IMPHY007852 | Azulene | c1ccc2-c(cc1)ccc2 | 9231 |
| 303 | IMPHY009853 | Naphthalene | c1ccc2c(c1)cccc2 | 931 |
| 304 | IMPHY009966 | Eucarvone | CC1=CC=CC(CC1=O)(C)C | 136330 |
| 305 | IMPHY011392 | 3-Carene | CC1=CCC2C(C1)C2(C)C | 26049 |
| 306 | IMPHY011643 | alpha-Terpinene | CC1=CC=C(CC1)C(C)C | 7462 |
| 307 | IMPHY011761 | Humulene | C/C/1=CCC(C)(C)/C=C/C/C(=C/CC1)/C | 5281520 |
| 308 | IMPHY012061 | alpha-Pinene | CC1=CCC2CC1C2(C)C | 6654 |
| 309 | IMPHY012095 | Cyclohexene | C1CCC=CC1 | 8079 |
| 310 | IMPHY012107 | Adamantane | C1C2CC3CC1CC(C2)C3 | 9238 |
| 311 | IMPHY012147 | beta-Pinene | C=C1CCC2CC1C2(C)C | 14896 |
| 312 | IMPHY012667 | Caryophyllene oxide | C=C1CC[C@H]2O[C@@]2(CC[C@@H]2[C@@H]1CC2(C)C)C | 1742210 |
| 313 | IMPHY012823 | 1,6-Cyclodecadiene | C1C/C=CCCC/C=CC1 | 5365639 |
| 314 | IMPHY014806 | Caswell No. 264AB | CC([C@@H]1CC[C@H]([C@]23[C@H]1[C@H]2C(=CC3)C)C)C | 442359 |
| 315 | IMPHY014831 | beta-Caryophyllene | C/C/1=CCCC(=C)[C@@H]2[C@@H](CC1)C(C2)(C)C | 5281515 |
| 316 | IMPHY014881 | Copaene | CC(C1CCC2(C3C1C2C(=CC3)C)C)C | 19725 |
| 317 | IMPHY014988 | Limonene | CC1=CCC(CC1)C(=C)C | 22311 |
| 318 | IMPHY003485 | Myrcene | C=CC(=C)CCC=C(C)C | 31253 |
| 319 | IMPHY003982 | gamma-Terpinene | CC1=CCC(=CC1)C(C)C | 7461 |
| 320 | IMPHY004192 | Piperine | O=C(N1CCCCC1)/C=C/C=C/c1ccc2c(c1)OCO2 | 638024 |
| 321 | IMPHY005618 | Germacrene B | C/C/1=CCC/C(=C/CC(=C(C)C)CC1)/C | 5281519 |
| 322 | IMPHY006145 | p-Cymene | Cc1ccc(cc1)C(C)C | 7463 |
| 323 | IMPHY006177 | Methyl geranate | COC(=O)/C=C(/CCC=C(C)C)C | 5365910 |
| 324 | IMPHY007840 | Spathulenol | C=C1CC[C@@H]2[C@H]([C@H]3[C@H]1CC[C@]3(C)O)C2(C)C | 92231 |
| 325 | IMPHY008150 | 1-Methyl-4-(prop-1-en-2-yl)benzene | Cc1ccc(cc1)C(=C)C | 62385 |
| 326 | IMPHY008910 | Hentriacontane | CCCCCCCCCCCCCCCCCCCCCCCCCCCCCCC | 12410 |
| 327 | IMPHY008930 | 16-Hentriacontanone | CCCCCCCCCCCCCCCC(=O)CCCCCCCCCCCCCCC | 94741 |
| 328 | IMPHY008936 | alpha-Guaiene | CC(=C)[C@@H]1CC[C@@H](C2=C(C1)[C@@H](C)CC2)C | 5317844 |
| 329 | IMPHY008946 | delta-Guaiene | CC(=C)[C@@H]1CCC(=C2[C@@H](C1)[C@@H](C)CC2)C | 94275 |
| 330 | IMPHY009642 | 2-Nonanone | CCCCCCCC(=O)C | 13187 |
| 331 | IMPHY010080 | beta-Elemene | C=C[C@]1(C)CC[C@H](C[C@H]1C(=C)C)C(=C)C | 6918391 |
| 332 | IMPHY011392 | 3-Carene | CC1=CCC2C(C1)C2(C)C | 26049 |
| 333 | IMPHY011396 | 4-Carvomenthenol | CC1=CCC(CC1)(O)C(C)C | 11230 |
| 334 | IMPHY011455 | Cadina-1,4-diene | CC1=CC2C(=CC1)[C@@H](C)CCC2C(C)C | 6427091 |
| 335 | IMPHY011521 | 2-Undecanone | CCCCCCCCCC(=O)C | 8163 |
| 336 | IMPHY011586 | (S,1Z,6Z)-8-Isopropyl-1-methyl-5-methylenecyclodeca-1,6-diene | C/C/1=C/CCC(=C)/C=C[C@@H](CC1)C(C)C | 91723653 |
| 337 | IMPHY011590 | d-Borneol | O[C@@H]1C[C@H]2C([C@@]1(C)CC2)(C)C | 61060 |
| 338 | IMPHY011599 | Terpinolene | CC1=CCC(=C(C)C)CC1 | 11463 |
| 339 | IMPHY011643 | alpha-Terpinene | CC1=CC=C(CC1)C(C)C | 7462 |
| 340 | IMPHY011761 | Humulene | C/C/1=CCC(C)(C)/C=C/C/C(=C/CC1)/C | 5281520 |
| 341 | IMPHY011789 | Citral | O=C/C=C(/CCC=C(C)C)C | 638011 |
| 342 | IMPHY011790 | Neral | O=C/C=C(CCC=C(C)C)/C | 643779 |
| 343 | IMPHY011890 | Elemol | C=C[C@]1(C)CC[C@H](C[C@H]1C(=C)C)C(O)(C)C | 92138 |
| 344 | IMPHY011957 | (+)-delta-Cadinene | CC1=C[C@@H]2C(=C(C)CC[C@H]2C(C)C)CC1 | 441005 |
| 345 | IMPHY011965 | (+)-beta-Phellandrene | CC([C@@H]1CCC(=C)C=C1)C | 442484 |
| 346 | IMPHY011988 | (-)-trans-Carveol | CC(=C)[C@@H]1CC=C([C@H](C1)O)C | 94221 |
| 347 | IMPHY012036 | Camphor | O=C1CC2C(C1(C)CC2)(C)C | 2537 |
| 348 | IMPHY012058 | Linalool | C=CC(CCC=C(C)C)(O)C | 6549 |
| 349 | IMPHY012061 | alpha-Pinene | CC1=CCC2CC1C2(C)C | 6654 |
| 350 | IMPHY012086 | Citronellal | O=CCC(CCC=C(C)C)C | 7794 |
| 351 | IMPHY012104 | Citronellol | OCCC(CCC=C(C)C)C | 8842 |
| 352 | IMPHY012147 | beta-Pinene | C=C1CCC2CC1C2(C)C | 14896 |
| 353 | IMPHY012160 | alpha-Terpineol | CC1=CCC(CC1)C(O)(C)C | 17100 |
| 354 | IMPHY012165 | Sabinene | C=C1CCC2(C1C2)C(C)C | 18818 |
| 355 | IMPHY012168 | (1S,2S,6S,7R,8R)-1,3-dimethyl-8-propan-2-yltricyclo[4.4.0.02,7]dec-3-ene | CC([C@H]1CC[C@]2([C@@H]3[C@@H]1[C@H]2C(=CC3)C)C)C | 101607926 |
| 356 | IMPHY012255 | (+)-trans-Piperitenol | CC1=C[C@@H]([C@H](CC1)C(C)C)O | 85568 |
| 357 | IMPHY012910 | trans-Calamenene | CC([C@H]1CC[C@@H](c2c1cc(C)cc2)C)C | 6429022 |
| 358 | IMPHY013080 | alpha-Calacorene | CC([C@@H]1CC=C(c2c1cc(C)cc2)C)C | 12302243 |
| 359 | IMPHY013093 | delta-Elemene | C=C[C@@]1(C)CCC(=C[C@@H]1C(=C)C)C(C)C | 12309449 |
| 360 | IMPHY013836 | Fenchone | O=C1C2(C)CCC(C1(C)C)C2 | 14525 |
| 361 | IMPHY014690 | (-)-Globulol | C[C@@H]1CC[C@@H]2[C@@H]1[C@H]1[C@H](C1(C)C)CC[C@@]2(C)O | 12304985 |
| 362 | IMPHY014806 | Caswell No. 264AB | CC([C@@H]1CC[C@H]([C@]23[C@H]1[C@H]2C(=CC3)C)C)C | 442359 |
| 363 | IMPHY014811 | alpha-Phellandrene | CC1=CCC(C=C1)C(C)C | 7460 |
| 364 | IMPHY014831 | beta-Caryophyllene | C/C/1=CCCC(=C)[C@@H]2[C@@H](CC1)C(C2)(C)C | 5281515 |
| 365 | IMPHY014835 | (E)-beta-ocimene | C=C/C(=C/CC=C(C)C)/C | 5281553 |
| 366 | IMPHY014836 | beta-Sitosterol | CC[C@@H](C(C)C)CC[C@H]([C@H]1CC[C@@H]2[C@]1(C)CC[C@H]1[C@H]2CC=C2[C@]1(C)CC[C@@H](C2)O)C | 222284 |
| 367 | IMPHY014847 | Bornyl acetate | CC(=O)OC1CC2C(C1(C)CC2)(C)C | 6448 |
| 368 | IMPHY014852 | Camphene | C=C1C2CCC(C1(C)C)C2 | 6616 |
| 369 | IMPHY014874 | cis-Sabinene hydrate | C[C@@H]1CC[C@@]2(C1C2)C(C)C | 101629835 |
| 370 | IMPHY014988 | Limonene | CC1=CCC(CC1)C(=C)C | 22311 |
| 371 | IMPHY014989 | trans-Linalool oxide | C=C[C@]1(C)CC[C@H](O1)C(O)(C)C | 6432254 |
| 372 | IMPHY015123 | alpha-Copaene | CC([C@@H]1CC[C@]2([C@@H]3[C@H]1C2C(=CC3)C)C)C | 70678558 |
| 373 | IMPHY002333 | N-isobutyl-2e,4e,8z-eicosatrienamide | CCCCCCCCCCC/C=CCC/C=C/C=C/C(=O)NCC(C)C |  |
| 374 | IMPHY003955 | (R)-4-Isopropylcyclohex-2-enone | CC([C@H]1CCC(=O)C=C1)C | 642520 |
| 375 | IMPHY004192 | Piperine | O=C(N1CCCCC1)/C=C/C=C/c1ccc2c(c1)OCO2 | 638024 |
| 376 | IMPHY004217 | Chavicine | O=C(N1CCCCC1)/C=CC=C/c1ccc2c(c1)OCO2 | 1548912 |
| 377 | IMPHY006362 | Ascorbic acid | OC[C@@H]([C@H]1OC(=O)C(=C1O)O)O | 54670067 |
| 378 | IMPHY007055 | Piperonal | O=Cc1ccc2c(c1)OCO2 | 8438 |
| 379 | IMPHY007188 | Piperidine | C1CCCNC1 | 8082 |
| 380 | IMPHY007269 | Trichostachine | O=C(N1CCCC1)/C=C/C=C/c1ccc2c(c1)OCO2 | 636537 |
| 381 | IMPHY011593 | Pellitorine | CCCCC/C=C/C=C/C(=O)NCC(C)C | 5318516 |
| 382 | IMPHY011609 | alpha-Carotene | C/C(=CC=CC=C(C=CC=C(C=CC1=C(C)CCCC1(C)C)/C)/C)/C=C/C=C(/C=C/[C@H]1C(=CCCC1(C)C)C)C | 6419725 |
| 383 | IMPHY011879 | Moupinamide | COc1cc(/C=C/C(=O)NCCc2ccc(cc2)O)ccc1O | 5280537 |
| 384 | IMPHY012104 | Citronellol | OCCC(CCC=C(C)C)C | 8842 |
| 385 | IMPHY014852 | Camphene | C=C1C2CCC(C1(C)C)C2 | 6616 |
| 386 | IMPHY000027 | (E)-Piperolein A | O=C(N1CCCCC1)CCCC/C=C/c1ccc2c(c1)OCO2 | 11141599 |
| 387 | IMPHY000056 | 1-[(2E,4E)-2,4-dodecadienoyl]pyrrolidine | CCCCCCC/C=C/C=C/C(=O)N1CCCC1 | 10999431 |
| 388 | IMPHY000099 | Myrtenol | OCC1=CCC2CC1C2(C)C | 10582 |
| 389 | IMPHY000289 | Retrofractamide A | CC(CNC(=O)/C=C/C=C/CC/C=C/c1ccc2c(c1)OCO2)C | 11012859 |
| 390 | IMPHY000399 | beta-Bisabolene | CC(=CCCC(=C)[C@H]1CCC(=CC1)C)C | 10104370 |
| 391 | IMPHY000545 | O-Cymene | CC(c1ccccc1C)C | 10703 |
| 392 | IMPHY001316 | (2E,4E,6E)-7-(2H-1,3-benzodioxol-5-yl)-1-(piperidin-1-yl)hepta-2,4,6-trien-1-one | O=C(N1CCCCC1)/C=C/C=C/C=C/c1ccc2c(c1)OCO2 | 10703 |
| 393 | IMPHY001565 | Capsaicin | COc1cc(CNC(=O)CCCC/C=C/C(C)C)ccc1O | 1548943 |
| 394 | IMPHY001912 | Cubebin | O[C@H]1OC[C@@H]([C@H]1Cc1ccc2c(c1)OCO2)Cc1ccc2c(c1)OCO2 | 117443 |
| 395 | IMPHY002872 | Piperolactam D | COc1c(OC)c2C(=O)Nc3c2c(c1O)c1ccccc1c3 | 14039008 |
| 396 | IMPHY002913 | Sesquisabinene | CC(=CCCC(C12CCC(=C)C2C1)C)C | 25202482 |
| 397 | IMPHY002950 | 2-Ethylpyrazine | CCc1cnccn1 | 26331 |
| 398 | IMPHY002958 | 2,3,5-Trimethylpyrazine | Cc1cnc(c(n1)C)C | 26808 |
| 399 | IMPHY002969 | Retrofractamide C | CC(CNC(=O)/C=C/CCCC/C=C/c1ccc2c(c1)OCO2)C | 25255091 |
| 400 | IMPHY003392 | Butylated hydroxytoluene | Cc1cc(c(c(c1)C(C)(C)C)O)C(C)(C)C | 31404 |
| 401 | IMPHY003484 | 2,5-Dimethylpyrazine | Cc1ncc(nc1)C | 31252 |
| 402 | IMPHY003485 | Myrcene | C=CC(=C)CCC=C(C)C | 31253 |
| 403 | IMPHY003536 | Eugenol | C=CCc1ccc(c(c1)OC)O | 3314 |
| 404 | IMPHY003616 | Bicyclogermacrene | C/C/1=CCC/C(=C/[C@H]2[C@@H](CC1)C2(C)C)/C | 13894537 |
| 405 | IMPHY003982 | gamma-Terpinene | CC1=CCC(=CC1)C(C)C | 7461 |
| 406 | IMPHY004022 | Tricyclo(6.3.1.02,5)dodecan-1-ol, 4,4,8-trimethyl-, (1R,2S,5R,8S)- | C[C@]12CCC[C@](C2)(O)[C@@H]2[C@@H](CC1)C(C2)(C)C | 11746218 |
| 407 | IMPHY004079 | Catechol | Oc1ccccc1O | 289 |
| 408 | IMPHY004192 | Piperine | O=C(N1CCCCC1)/C=C/C=C/c1ccc2c(c1)OCO2 | 638024 |
| 409 | IMPHY004531 | p-Mentha-1,8-dien-4-ol | CC1=CCC(CC1)(O)C(=C)C | 527428 |
| 410 | IMPHY004549 | Safrole | C=CCc1ccc2c(c1)OCO2 | 5144 |
| 411 | IMPHY005670 | ,3-Dimethylpyrazine | Cc1nccnc1C | 22201 |
| 412 | IMPHY005720 | Pipercide | O=C(N1CCCCC1)CCCCCC/C=C/c1ccc2c(c1)OCO2 | 21580213 |
| 413 | IMPHY006145 | p-Cymene | Cc1ccc(cc1)C(C)C | 7463 |
| 414 | IMPHY006184 | Dehydropipernonaline | O=C(N1CCCCC1)/C=C/C=C/CC/C=C/c1ccc2c(c1)OCO2 | 6439947 |
| 415 | IMPHY006207 | Wisanine | COc1cc2OCOc2cc1/C=C/C=C/C(=O)N1CCCCC1 | 6441085 |
| 416 | IMPHY006215 | (2E,4E)-1-(Pyrrolidin-1-yl)deca-2,4-dien-1-one | CCCCC/C=C/C=C/C(=O)N1CCCC1 | 6440616 |
| 417 | IMPHY006266 | Guineensine | CC(CNC(=O)/C=C/C=C/CCCCCC/C=C/c1ccc2c(c1)OCO2)C | 6442405 |
| 418 | IMPHY006292 | Kakuol | CCC(=O)c1cc2OCOc2cc1O | 596894 |
| 419 | IMPHY006362 | Ascorbic acid | OC[C@@H]([C@H]1OC(=O)C(=C1O)O)O | 54670067 |
| 420 | IMPHY005944 | Pipercide | CC(CNC(=O)/C=C/C=C/CCCC/C=C/c1ccc2c(c1)OCO2)C | 5372162 |
| 421 | IMPHY006515 | Hedycaryol | C/C/1=C/CC/C(=CC[C@H](CC1)C(O)(C)C)/C | 6432240 |
| 422 | IMPHY006932 | Cuparene | Cc1ccc(cc1)[C@]1(C)CCCC1(C)C | 86895 |
| 423 | IMPHY006953 | 2,6-Dimethylpyrazine | Cc1cncc(n1)C | 7938 |
| 424 | IMPHY006978 | 2-Methylpyrazine | Cc1cnccn1 | 7976 |
| 425 | IMPHY007055 | Piperonal | O=Cc1ccc2c(c1)OCO2 | 8438 |
| 426 | IMPHY007188 | Piperidine | C1CCCNC1 | 8082 |
| 427 | IMPHY007202 | Nonane | CCCCCCCCC | 8141 |
| 428 | IMPHY007269 | Trichostachine | O=C(N1CCCC1)/C=C/C=C/c1ccc2c(c1)OCO2 | 636537 |
| 429 | IMPHY007371 | Cepharadione A | O=C1N(C)c2cc3ccccc3c3c2c(C1=O)cc1c3OCO1 | 94577 |
| 430 | IMPHY007442 | 5-Methyl-2-propan-2-ylcyclohexa-2,4-dien-1-ol | CC1=CC=C(C(C1)O)C(C)C | 91752234 |
| 431 | IMPHY007445 | (Z)-beta-Ocimenol | C=C/C(=CC(C=C(C)C)O)/C | 91753567 |
| 432 | IMPHY007559 | Pipernonaline | O=C(N1CCCCC1)/C=C/CCCC/C=C/c1ccc2c(c1)OCO2 | 9974595 |
| 433 | IMPHY007574 | Curcumin | COc1cc(/C=C/C(=O)CC(=O)/C=C/c2ccc(c(c2)OC)O)ccc1O | 969516 |
| 434 | IMPHY007840 | Spathulenol | C=C1CC[C@@H]2[C@H]([C@H]3[C@H]1CC[C@]3(C)O)C2(C)C | 92231 |
| 435 | IMPHY008686 | 2-Ethyl-5-methylpyrazine | CCc1cnc(cn1)C | 25915 |
| 436 | IMPHY008936 | alpha-Guaiene | CC(=C)[C@@H]1CC[C@@H](C2=C(C1)[C@@H](C)CC2)C | 5317844 |
| 437 | IMPHY008946 | delta-Guaiene | CC(=C)[C@@H]1CCC(=C2[C@@H](C1)[C@@H](C)CC2)C | 94275 |
| 438 | IMPHY009604 | Dihydroferuperine | COC1=CC(=CCC1O)/C=CC=CC(=O)N1CCCCC1 | 131752910 |
| 439 | IMPHY009605 | Feruperine | COc1cc(/C=CC=CC(=O)N2CCCCC2)ccc1O | 131752909 |
| 440 | IMPHY010072 | Eucalyptol | CC12CCC(CC1)C(O2)(C)C | 2758 |
| 441 | IMPHY010080 | beta-Elemene | C=C[C@]1(C)CC[C@H](C[C@H]1C(=C)C)C(=C)C | 6918391 |
| 442 | IMPHY010205 | Pipecolic acid | OC(=O)C1CCCCN1 | 849 |
| 443 | IMPHY010244 | (1S,5S,8S)-4,4,8-Trimethyltricyclo[6.3.1.01,5]dodec-2-ene | C[C@]12CCC[C@@]3(C2)[C@@H](CC1)C(C=C3)(C)C | 10102 |
| 444 | IMPHY011392 | 3-Carene | CC1=CCC2C(C1)C2(C)C | 26049 |
| 445 | IMPHY011396 | 4-Carvomenthenol | CC1=CCC(CC1)(O)C(C)C | 11230 |
| 446 | IMPHY011464 | Isobutyramide | CC(C(=O)N)C | 68424 |
| 447 | IMPHY011519 | alpha-Terpinyl acetate | CC(=O)OC(C1CCC(=CC1)C)(C)C | 111037 |
| 448 | IMPHY011542 | beta-Eudesmol | C=C1CCC[C@]2([C@H]1C[C@@H](CC2)C(O)(C)C)C | 91457 |
| 449 | IMPHY011552 | (1R)-2-methyl-5-propan-2-ylbicyclo[3.1.0]hex-2-ene | CC1=CCC2([C@@H]1C2)C(C)C | 6451618 |
| 450 | IMPHY011581 | alpha-Selinene | CC1=CCC[C@]2([C@H]1C[C@@H](CC2)C(=C)C)C | 10856614 |
| 451 | IMPHY011586 | (S,1Z,6Z)-8-Isopropyl-1-methyl-5-methylenecyclodeca-1,6-diene | C/C/1=C/CCC(=C)/C=C[C@@H](CC1)C(C)C | 91723653 |
| 452 | IMPHY011590 | d-Borneol | O[C@@H]1C[C@H]2C([C@@]1(C)CC2)(C)C | 61060 |
| 453 | IMPHY011593 | Pellitorine | CCCCC/C=C/C=C/C(=O)NCC(C)C | 5318516 |
| 454 | IMPHY011599 | Terpinolene | CC1=CCC(=C(C)C)CC1 | 11463 |
| 455 | IMPHY011609 | alpha-Carotene | C/C(=CC=CC=C(C=CC=C(C=CC1=C(C)CCCC1(C)C)/C)/C)/C=C/C=C(/C=C/[C@H]1C(=CCCC1(C)C)C)C | 6419725 |
| 456 | IMPHY011643 | alpha-Terpinene | CC1=CC=C(CC1)C(C)C | 7462 |
| 457 | IMPHY011647 | Geranyl acetate | C/C(=CCOC(=O)C)/CCC=C(C)C | 1549026 |
| 458 | IMPHY011648 | Neryl acetate | C/C(=C/COC(=O)C)/CCC=C(C)C | 1549025 |
| 459 | IMPHY011658 | beta-Farnesene | C=CC(=C)CC/C=C(/CCC=C(C)C)C | 5281517 |
| 460 | IMPHY011741 | Tannic acid | O=C(c1cc(O)c(c(c1)OC(=O)c1cc(O)c(c(c1)O)O)O)O[C@@H]1[C@@H](COC(=O)c2cc(O)c(c(c2)OC(=O)c2cc(O)c(c(c2)O)O)O)O[C@H]([C@@H][C@H]1OC(=O)c1cc(O)c(c(c1)OC(=O)c1cc(O)c(c(c1)O)O)O)OC(=O)c1cc(O)c(c(c1)OC(=O)c1cc(O)c(c(c1)O)O)O)OC(=O)c1cc(O)c(c(c1)OC(=O)c1cc(O)c(c(c1)O)O)O | 16129778 |
| 461 | IMPHY011761 | Humulene | C/C/1=CCC(C)(C)/C=C/C/C(=C/CC1)/C | 5281520 |
| 462 | IMPHY011792 | gamma-Muurolene | CC1=C[C@@H]2[C@H](CC1)C(=C)CC[C@H]2C(C)C | 12313020 |
| 463 | IMPHY011817 | alpha-Farnesene | C=C/C(=C/C/C=C(/CCC=C(C)C)C)/C | 5281516 |
| 464 | IMPHY011879 | Moupinamide | COc1cc(/C=C/C(=O)NCCc2ccc(cc2)O)ccc1O | 5280537 |
| 465 | IMPHY011890 | Elemol | C=C[C@]1(C)CC[C@H](C[C@H]1C(=C)C)C(O)(C)C | 92138 |
| 466 | IMPHY011957 | (+)-delta-Cadinene | CC1=C[C@@H]2C(=C(C)CC[C@H]2C(C)C)CC1 | 441005 |
| 467 | IMPHY011988 | (-)-trans-Carveol | CC(=C)[C@@H]1CC=C([C@H](C1)O)C | 94221 |
| 468 | IMPHY012012 | (E,E)-2,4-Decadienoic isobutylamide | CCCCC/C=CC=CC(=O)NCC(C)C | 11368078 |
| 469 | IMPHY012058 | Linalool | C=CC(CCC=C(C)C)(O)C | 6549 |
| 470 | IMPHY012061 | alpha-Pinene | CC1=CCC2CC1C2(C)C | 6654 |
| 471 | IMPHY012086 | Citronellal | O=CCC(CCC=C(C)C)C | 7794 |
| 472 | IMPHY012104 | Citronellol | OCCC(CCC=C(C)C)C | 8842 |
| 473 | IMPHY012130 | Dihydrocarveol | CC(=C)C1CCC(C(C1)O)C | 12072 |
| 474 | IMPHY012147 | beta-Pinene | C=C1CCC2CC1C2(C)C | 14896 |
| 475 | IMPHY012160 | alpha-Terpineol | CC1=CCC(CC1)C(O)(C)C | 17100 |
| 476 | IMPHY012165 | Sabinene | C=C1CCC2(C1C2)C(C)C | 18818 |
| 477 | IMPHY012175 | D-Limonene | CC1=CC[C@@H](CC1)C(=C)C | 440917 |
| 478 | IMPHY012279 | alpha-Curcumene | CC(=CCCC(c1ccc(cc1)C)C)C | 92139 |
| 479 | IMPHY012667 | Caryophyllene oxide | C=C1CC[C@H]2O[C@@]2(CC[C@@H]2[C@@H]1CC2(C)C)C | 1742210 |
| 480 | IMPHY014708 | beta-Selinene | C=C1CCC[C@]2([C@H]1C[C@@H](CC2)C(=C)C)C | 442393 |
| 481 | IMPHY014801 | Zizanene | CC1=C[C@@H]2[C@H](CC1)C(=CC[C@@H]2C(C)C)C | 12306046 |
| 482 | IMPHY014806 | Caswell No. 264AB | CC([C@@H]1CC[C@H]([C@]23[C@H]1[C@H]2C(=CC3)C)C)C | 442359 |
| 483 | IMPHY014811 | alpha-Phellandrene | CC1=CCC(C=C1)C(C)C | 7460 |
| 484 | IMPHY014831 | beta-Caryophyllene | C/C/1=CCCC(=C)[C@@H]2[C@@H](CC1)C(C2)(C)C | 5281515 |
| 485 | IMPHY014835 | (E)-beta-ocimene | C=C/C(=C/CC=C(C)C)/C | 5281553 |
| 486 | IMPHY014852 | Camphene | C=C1C2CCC(C1(C)C)C2 | 6616 |
| 487 | IMPHY014857 | Cedrol | C[C@@H]1CC[C@@H]2[C@@]31CC[C@@]([C@H](C3)C2(C)C)(C)O | 65575 |
| 488 | IMPHY014871 | cis-Nerolidol | C=CC(CC/C=C(CCC=C(C)C)/C)(O)C | 5320128 |
| 489 | IMPHY014907 | 6-Epi-beta-bisabolol | CC(=CCC[C@@H]([C@@]1(O)CCC(=CC1)C)C)C | 12300148 |
| 490 | IMPHY014988 | Limonene | CC1=CCC(CC1)C(=C)C | 22311 |
| 491 | IMPHY014989 | trans-Linalool oxide | C=C[C@]1(C)CC[C@H](O1)C(O)(C)C | 6432254 |
| 492 | IMPHY015042 | Piperitone | CC1=CC(=O)C(CC1)C(C)C | 6987 |
| 493 | IMPHY015095 | 2-Cyclohexen-1-ol, 1-methyl-4-(1-methylethyl)-, trans- | CC([C@@H]1CC[C@@](C=C1)(C)O)C | 122484 |
| 494 | IMPHY015123 | alpha-Copaene | CC([C@@H]1CC[C@]2([C@@H]3[C@H]1C2C(=CC3)C)C)C | 70678558 |
| 495 | IMPHY015919 | 2-Ethyl-6-methylpyrazine | CCc1cncc(n1)C | 26332 |
| 496 | IMPHY016027 | trans-Sabinene hydrate | CC([C@@]12CC[C@](C2C1)(C)O)C | 12315151 |
| 497 | IMPHY016054 | trans-alpha-Bergamotene | CC(=CCC[C@]1(C)[C@H]2CC=C([C@@H]1C2)C)C | 6429302 |
| 498 | IMPHY016453 | 5-Ethyl-2-methylpyridine | CCc1ccc(nc1)C | 7728 |
| 499 | IMPHY017003 | 1-Acetylpiperidine | CC(=O)N1CCCCC1 | 12058 |
| 500 | IMPHY017167 | N-Formylpiperidine | O=CN1CCCCC1 | 17429 |
| 501 | IMPHY017242 | 3-Ethyl-2,5-dimethylpyrazine | CCc1nc(C)cnc1C | 25916 |
| 502 | IMPHY017245 | 2-Ethyl-3,5-dimethylpyrazine | CCc1ncc(nc1C)C | 26334 |
| 503 | IMPHY017248 | 2-Ethyl-3-methylpyrazine | CCc1nccnc1C | 27457 |
| 504 | IMPHY017258 | 3,5-Diethyl-2-methylpyrazine | CCc1cnc(c(n1)CC)C | 28906 |
| 505 | IMPHY017687 | 2-Isopropylpyridine | CC(c1ccccn1)C | 69523 |
| 506 | IMPHY017708 | 2-Heptylpyridine | CCCCCCCc1ccccn1 | 72919 |
| 507 | IMPHY017728 | 2-Ethylpyridine | CCc1ccccn1 | 7523 |
| 508 | IMPHY017744 | 2-Butylpyridine | CCCCc1ccccn1 | 78750 |
| 509 | IMPHY017751 | 2,6-Dimethylpyridine | Cc1cccc(n1)C | 7937 |
|  |  |  |  |  |
| **PLANT NAME : *Plantago ovata*** | | | | |
| **Serial No** | **IMPPAT Phytochemical Identifier** | **Phytochemical Name** | **SMILES** | **CID** |
| 1 | IMPHY012053 | Sucrose | OC[C@H]1O[C@@]([C@H]([C@@H]1O)O)(CO)O[C@H]1O[C@H](CO)[C@H]([C@@H]([C@H]1O)O)O | 5988 |
| 2 | IMPHY000165 | Tetracosanoic acid | CCCCCCCCCCCCCCCCCCCCCCCC(=O)O | 11197 |
| 3 | IMPHY004187 | L-(+)-Arabinose | OC[C@@H]([C@@H]([C@H](C=O)O)O)O | 5460291 |
| 4 | IMPHY004631 | Stearic acid | CCCCCCCCCCCCCCCCCC(=O)O | 5281 |
| 5 | IMPHY006362 | Ascorbic acid | OC[C@@H]([C@H]1OC(=O)C(=C1O)O)O | 54670067 |
| 6 | IMPHY006415 | 12-Octadecenoic acid, 9-oxo-, (Z)- | CCCCCC=CCCC(=O)CCCCCCCC(=O)O | 53686719 |
| 7 | IMPHY007327 | Palmitic acid | CCCCCCCCCCCCCCCC(=O)O | 985 |
| 8 | IMPHY011516 | Aucubin | OCC1=C[C@H]([C@H]2[C@@H]1[C@@H](OC=C2)O[C@@H]1O[C@H](CO)[C@H]([C@@H]([C@H]1O)O)O)O | 91458 |
| 9 | IMPHY011741 | Tannic acid | O=C(c1cc(O)c(c(c1)OC(=O)c1cc(O)c(c(c1)O)O)O)O[C@@H]1[C@@H](COC(=O)c2cc(O)c(c(c2)OC(=O)c2cc(O)c(c(c2)O)O)O)O[C@H]([C@@H]([C@H]1OC(=O)c1cc(O)c(c(c1)OC(=O)c1cc(O)c(c(c1)O)O)O)OC(=O)c1cc(O)c(c(c1)OC(=O)c1cc(O)c(c(c1)O)O)O)OC(=O)c1cc(O)c(c(c1)OC(=O)c1cc(O)c(c(c1)O)O)O | 16129778 |
| 10 | IMPHY011797 | Oleic acid | CCCCCCCC/C=CCCCCCCCC(=O)O | 445639 |
| 11 | IMPHY012050 | D-Galactose | OC[C@H]1OC(O)[C@@H]([C@H]([C@H]1O)O)O | 6036 |
| 12 | IMPHY012723 | Linolenic acid | CC/C=CC/C=CC/C=CCCCCCCCC(=O)O | 5280934 |
| 13 | IMPHY014836 | beta-Sitosterol | CC[C@@H](C(C)C)CC[C@H]([C@H]1CC[C@@H]2[C@]1(C)CC[C@H]1[C@H]2CC=C2[C@]1(C)CC[C@@H](C2)O)C | 222284 |
| 14 | IMPHY014919 | D-Galacturonic Acid | OC1O[C@H](C(=O)O)[C@@H]([C@@H]([C@H]1O)O)O | 439215 |
| 15 | IMPHY014990 | Linoleic acid | CCCCC/C=CC/C=CCCCCCCCC(=O)O | 5280450 |
| 16 | IMPHY015056 | L-Rhamnose | O[C@H]1[C@H](C)OC([C@@H]([C@@H]1O)O)O | 25310 |
| 17 | IMPHY015116 | D-Xylose | O[C@@H]1COC([C@@H]([C@H]1O)O)O | 135191 |
| 18 | IMPHY012053 | Sucrose | OC[C@H]1O[C@@]([C@H]([C@@H]1O)O)(CO)O[C@H]1O[C@H](CO)[C@H]([C@@H]([C@H]1O)O)O | 5988 |
| 19 | IMPHY000165 | Tetracosanoic acid | CCCCCCCCCCCCCCCCCCCCCCCC(=O)O | 11197 |
| 20 | IMPHY001045 | Boschniakine | O=Cc1cncc2c1CC[C@H]2C | 442507 |
| 21 | IMPHY002137 | Boschniakinic acid | C[C@@H]1CCc2c1cncc2C(=O)O | 12300212 |
| 22 | IMPHY003358 | Stachyose | OC[C@H]1O[C@@]([C@H]([C@@H]1O)O)(CO)O[C@H]1O[C@H](CO[C@H]2O[C@H](CO[C@H]3O[C@H](CO)[C@@H]([C@@H]([C@H]3O)O)O)[C@@H]([C@@H]([C@H]2O)O)O)[C@H]([C@@H]([C@H]1O)O)O | 439531 |
| 23 | IMPHY003438 | Planteose | OC[C@H]1O[C@H](OC[C@H]2O[C@@]([C@H]([C@@H]2O)O)(CO)O[C@H]2O[C@H](CO)[C@H]([C@@H]([C@H]2O)O)O)[C@@H]([C@H]([C@H]1O)O)O | 440140 |
| 24 | IMPHY004167 | beta-D-Xylopyranose | O[C@@H]1CO[C@H]([C@@H]([C@H]1O)O)O | 125409 |
| 25 | IMPHY004187 | L-(+)-Arabinose | OC[C@@H]([C@@H]([C@H](C=O)O)O)O | 5460291 |
| 26 | IMPHY004631 | Stearic acid | CCCCCCCCCCCCCCCCCC(=O)O | 5281 |
| 27 | IMPHY006362 | Ascorbic acid | OC[C@@H]([C@H]1OC(=O)C(=C1O)O)O | 54670067 |
| 28 | IMPHY006415 | 12-Octadecenoic acid, 9-oxo-, (Z)- | CCCCCC=CCCC(=O)CCCCCCCC(=O)O | 53686719 |
| 29 | IMPHY007212 | Docosanoic acid | CCCCCCCCCCCCCCCCCCCCCC(=O)O | 8215 |
| 30 | IMPHY007327 | Palmitic acid | CCCCCCCCCCCCCCCC(=O)O | 985 |
| 31 | IMPHY008709 | 4-O-Methylglucuronic acid | O=C[C@@H]([C@H]([C@@H]([C@@H](C(=O)O)O)OC)O)O | 151010 |
| 32 | IMPHY011516 | Aucubin | OCC1=C[C@H]([C@H]2[C@@H]1[C@@H](OC=C2)O[C@@H]1O[C@H](CO)[C@H]([C@@H]([C@H]1O)O)O)O | 91458 |
| 33 | IMPHY011597 | Aldobiouronic acid | O=C[C@@H]([C@H]([C@H]([C@@H](CO[C@@H]1O[C@H](C(=O)O)[C@H]([C@@H]([C@H]1O)O)O)O)O)O)O | 101286238 |
| 34 | IMPHY011619 | alpha-Amyrin | C[C@@H]1CC[C@]2([C@@H]([C@H]1C)C1=CC[C@H]3[C@@]([C@@]1(CC2)C)(C)CC[C@@H]1[C@]3(C)CC[C@@H](C1(C)C)O)C | 73170 |
| 35 | IMPHY011797 | Oleic acid | CCCCCCCC/C=CCCCCCCCC(=O)O | 445639 |
| 36 | IMPHY012050 | D-Galactose | OC[C@H]1OC(O)[C@@H]([C@H]([C@H]1O)O)O | 6036 |
| 37 | IMPHY012223 | beta-Amyrin | O[C@H]1CC[C@]2([C@H](C1(C)C)CC[C@@]1([C@@H]2CC=C2[C@@]1(C)CC[C@@]1([C@H]2CC(C)(C)CC1)C)C)C | 73145 |
| 38 | IMPHY012320 | Raffinose | OC[C@H]1O[C@@]([C@H]([C@@H]1O)O)(CO)O[C@H]1O[C@H](CO[C@H]2O[C@H](CO)[C@@H]([C@@H]([C@H]2O)O)O)[C@H]([C@@H]([C@H]1O)O)O | 439242 |
| 39 | IMPHY012402 | Campesterol | O[C@H]1CC[C@]2(C(=CC[C@@H]3[C@@H]2CC[C@]2([C@H]3CC[C@@H]2[C@@H](CC[C@H](C(C)C)C)C)C)C1)C | 173183 |
| 40 | IMPHY012723 | Linolenic acid | CC/C=CC/C=CC/C=CCCCCCCCC(=O)O | 5280934 |
| 41 | IMPHY014836 | beta-Sitosterol | CC[C@@H](C(C)C)CC[C@H]([C@H]1CC[C@@H]2[C@]1(C)CC[C@H]1[C@H]2CC=C2[C@]1(C)CC[C@@H](C2)O)C | 222284 |
| 42 | IMPHY014893 | D-Glucose | OC[C@H]1OC(O)[C@@H]([C@H]([C@@H]1O)O)O | 5793 |
| 43 | IMPHY014916 | D-Fructose | OCC1(O)OC[C@H]([C@H]([C@@H]1O)O)O | 2723872 |
| 44 | IMPHY014919 | D-Galacturonic Acid | OC1O[C@H](C(=O)O)[C@@H]([C@@H]([C@H]1O)O)O | 439215 |
| 45 | IMPHY014990 | Linoleic acid | CCCCC/C=CC/C=CCCCCCCCC(=O)O | 5280450 |
| 46 | IMPHY015056 | L-Rhamnose | O[C@H]1[C@H](C)OC([C@@H]([C@@H]1O)O)O | 25310 |
| 47 | IMPHY015116 | D-Xylose | O[C@@H]1COC([C@@H]([C@H]1O)O)O | 135191 |
|  |  |  |  |  |
| **PLANT NAME : *Quercus infectoria*** | | | | |
| **Serial No** | **IMPPAT Phytochemical Identifier** | **Phytochemical Name** | **SMILES** | **CID** |
| 1 | IMPHY003399 | 8-[5-(5,7-Dimethoxy-4-oxochromen-2-yl)-2-methoxyphenyl]-5,7-dimethoxy-2-(4-methoxyphenyl)chromen-4-one | COc1ccc(cc1)c1cc(=O)c2c(o1)c(c(cc2OC)OC)c1cc(ccc1OC)c1cc(=O)c2c(o1)cc(cc2OC)OC | 3336566 |
| 2 | IMPHY004995 | 6-[4-(5,7-Dihydroxy-4-oxochromen-2-yl)phenoxy]-5-hydroxy-2-(4-hydroxyphenyl)-7-methoxychromen-4-one | COc1cc2oc(cc(=O)c2c(c1Oc1ccc(cc1)c1cc(=O)c2c(o1)cc(cc2O)O)O)c1ccc(cc1)O | 5318537 |
| 3 | IMPHY005599 | Amentoflavone | Oc1ccc(cc1)c1cc(=O)c2c(o1)c(c(cc2O)O)c1cc(ccc1O)c1cc(=O)c2c(o1)cc(cc2O)O | 5281600 |
| 4 | IMPHY014836 | beta-Sitosterol | CC[C@@H](C(C)C)CC[C@H]([C@H]1CC[C@@H]2[C@]1(C)CC[C@H]1[C@H]2CC=C2[C@]1(C)CC[C@@H](C2)O)C | 222284 |
| 5 | IMPHY000687 | Syringic acid | COc1cc(cc(c1O)OC)C(=O)O | 10742 |
| 6 | IMPHY002588 | Flavylium | c1ccc(cc1)c1ccc2c([o+]1)cccc2 | 145858 |
| 7 | IMPHY004995 | 6-[4-(5,7-Dihydroxy-4-oxochromen-2-yl)phenoxy]-5-hydroxy-2-(4-hydroxyphenyl)-7-methoxychromen-4-one | COc1cc2oc(cc(=O)c2c(c1Oc1ccc(cc1)c1cc(=O)c2c(o1)cc(cc2O)O)O)c1ccc(cc1)O | 5318537 |
| 8 | IMPHY005537 | Ellagic acid | Oc1cc2c(=O)oc3c4c2c(c1O)oc(=O)c4cc(c3O)O | 5281855 |
| 9 | IMPHY005599 | Amentoflavone | Oc1ccc(cc1)c1cc(=O)c2c(o1)c(c(cc2O)O)c1cc(ccc1O)c1cc(=O)c2c(o1)cc(cc2O)O | 5281600 |
| 10 | IMPHY009338 | Nyctanthic acid | OC(=O)CC[C@@]1(C)[C@@H](CC[C@@]2([C@@H]1CC=C1[C@@]2(C)CC[C@@]2([C@H]1CC(C)(C)CC2)C)C)C(=C)C | 12313631 |
| 11 | IMPHY011461 | Methyl oleanolate | COC(=O)[C@@]12CCC(C[C@H]2C2=CC[C@H]3[C@@]([C@@]2(CC1)C)(C)CC[C@@H]1[C@]3(C)CC[C@@H](C1(C)C)O)(C)C | 92900 |
| 12 | IMPHY011741 | Tannic acid | O=C(c1cc(O)c(c(c1)OC(=O)c1cc(O)c(c(c1)O)O)O)O[C@@H]1[C@@H](COC(=O)c2cc(O)c(c(c2)OC(=O)c2cc(O)c(c(c2)O)O)O)O[C@H]([C@@H]([C@H]1OC(=O)c1cc(O)c(c(c1)OC(=O)c1cc(O)c(c(c1)O)O)O)OC(=O)c1cc(O)c(c(c1)OC(=O)c1cc(O)c(c(c1)O)O)O)OC(=O)c1cc(O)c(c(c1)OC(=O)c1cc(O)c(c(c1)O)O)O | 16129778 |
| 13 | IMPHY012021 | Gallic acid | OC(=O)c1cc(O)c(c(c1)O)O | 370 |
| 14 | IMPHY012231 | Methyl betulate | COC(=O)C12CCC(C2C2C(CC1)(C)C1(C)CCC3C(C1CC2)(C)CCC(C3(C)C)O)C(=C)C | 423298 |
| 15 | IMPHY014836 | beta-Sitosterol | CC[C@@H](C(C)C)CC[C@H]([C@H]1CC[C@@H]2[C@]1(C)CC[C@H]1[C@H]2CC=C2[C@]1(C)CC[C@@H](C2)O)C | 222284 |
| 16 | IMPHY000659 | 4-Glucogallic acid | OC[C@H]1O[C@@H](Oc2c(O)cc(cc2O)C(=O)O)[C@@H]([C@H]([C@@H]1O)O)O | 10088114 |
| 17 | IMPHY000687 | Syringic acid | COc1cc(cc(c1O)OC)C(=O)O | 10742 |
| 18 | IMPHY003474 | 1,6-bis-O-galloyl-beta-D-glucose | O[C@@H]1[C@@H](COC(=O)c2cc(O)c(c(c2)O)O)O[C@H]([C@@H]([C@H]1O)O)OC(=O)c1cc(O)c(c(c1)O)O | 440221 |
| 19 | IMPHY003557 | Digallic acid | O=C(c1cc(O)c(c(c1)O)O)Oc1cc(cc(c1O)O)C(=O)O | 341 |
| 20 | IMPHY003996 | Anacardic acid | CCCCCCCCCCCCCCCc1cccc(c1C(=O)O)O | 167551 |
| 21 | IMPHY004619 | Quercetin | Oc1cc(O)c2c(c1)oc(c(c2=O)O)c1ccc(c(c1)O)O | 5280343 |
| 22 | IMPHY005537 | Ellagic acid | Oc1cc2c(=O)oc3c4c2c(c1O)oc(=O)c4cc(c3O)O | 5281855 |
| 23 | IMPHY006658 | Pentagalloylglucose | O=C(c1cc(O)c(c(c1)O)O)O[C@@H]1O[C@H](COC(=O)c2cc(O)c(c(c2)O)O)[C@H]([C@@H]([C@H]1OC(=O)c1cc(O)c(c(c1)O)O)OC(=O)c1cc(O)c(c(c1)O)O)OC(=O)c1cc(O)c(c(c1)O)O | 65238 |
| 24 | IMPHY006939 | [(2R,3R,4S,5R)-6,7-dioxo-2,3,4,5-tetrakis[(3,4,5-trihydroxybenzoyl)oxy]-7-(3,4,5-trihydroxyphenyl)heptyl] 3,4,5-trihydroxybenzoate | O=C(c1cc(O)c(c(c1)O)O)O[C@@H]([C@@H]([C@H](C(=O)C(=O)c1cc(O)c(c(c1)O)O)OC(=O)c1cc(O)c(c(c1)O)O)OC(=O)c1cc(O)c(c(c1)O)O)[C@H](OC(=O)c1cc(O)c(c(c1)O)O)COC(=O)c1cc(O)c(c(c1)O)O | 87574482 |
| 25 | IMPHY010070 | beta-Glucogallin | OC[C@H]1O[C@@H](OC(=O)c2cc(O)c(c(c2)O)O)[C@@H]([C@H]([C@@H]1O)O)O | 124021 |
| 26 | IMPHY010353 | 2-O,3-O,4-O,6-O-Tetragalloyl-D-glucopyranose | OC1O[C@H](COC(=O)c2cc(O)c(c(c2)O)O)[C@H]([C@@H]([C@H]1OC(=O)c1cc(O)c(c(c1)O)O)OC(=O)c1cc(O)c(c(c1)O)O)OC(=O)c1cc(O)c(c(c1)O)O | 13888122 |
| 27 | IMPHY011740 | Gallotannin | O=C(c1cc(O)c(c(c1)OC(=O)c1cc(O)c(c(c1)O)O)O)O[C@H]1[C@H](COC(=O)c2cc(O)c(c(c2)OC(=O)c2cc(O)c(c(c2)O)O)O)O[C@@H]([C@H][C@@H]1OC(=O)c1cc(O)c(c(c1)OC(=O)c1cc(O)c(c(c1)O)O)O)OC(=O)c1cc(O)c(c(c1)OC(=O)c1cc(O)c(c(c1)O)O)O)OC(=O)c1cc(O)c(c(c1)OC(=O)c1cc(O)c(c(c1)O)O)O | 16133892 |
| 28 | IMPHY011741 | Tannic acid | O=C(c1cc(O)c(c(c1)OC(=O)c1cc(O)c(c(c1)O)O)O)O[C@@H]1[C@@H](COC(=O)c2cc(O)c(c(c2)OC(=O)c2cc(O)c(c(c2)O)O)O)O[C@H]([C@@H]([C@H]1OC(=O)c1cc(O)c(c(c1)OC(=O)c1cc(O)c(c(c1)O)O)O)OC(=O)c1cc(O)c(c(c1)OC(=O)c1cc(O)c(c(c1)O)O)O)OC(=O)c1cc(O)c(c(c1)OC(=O)c1cc(O)c(c(c1)O)O)O | 16129778 |
| 29 | IMPHY012021 | Gallic acid | OC(=O)c1cc(O)c(c(c1)O)O | 370 |
|  |  |  |  |  |
| **PLANT NAME : *Rosa centifolia*** | | | | |
| Serial No | IMPPAT Phytochemical Identifier | Phytochemical Name | SMILES | CID |
| 1 | IMPHY000308 | Hexadecane | CCCCCCCCCCCCCCCC | 11006 |
| 2 | IMPHY001846 | Dimethyl disulfide | CSSC | 12232 |
| 3 | IMPHY001854 | 3-Methylbutanal | O=CCC(C)C | 11552 |
| 4 | IMPHY001896 | Heptacosane | CCCCCCCCCCCCCCCCCCCCCCCCCCC | 11636 |
| 5 | IMPHY001915 | Octadecane | CCCCCCCCCCCCCCCCCC | 11635 |
| 6 | IMPHY002434 | beta-Maaliene | CC1=C2[C@H]3[C@H](C3(C)C)CC[C@@]2(CCC1)C | 101596917 |
| 7 | IMPHY002875 | Undecane | CCCCCCCCCCC | 14257 |
| 8 | IMPHY002915 | Benzyl Alcohol | OCc1ccccc1 | 244 |
| 9 | IMPHY002949 | 1-Butanol | CCCCO | 263 |
| 10 | IMPHY003050 | Methyl salicylate | COC(=O)c1ccccc1O | 4133 |
| 11 | IMPHY003482 | 4-Methoxybenzaldehyde | COc1ccc(cc1)C=O | 31244 |
| 12 | IMPHY003485 | Myrcene | C=CC(=C)CCC=C(C)C | 31253 |
| 13 | IMPHY003519 | Isoamyl acetate | CC(CCOC(=O)C)C | 31276 |
| 14 | IMPHY003536 | Eugenol | C=CCc1ccc(c(c1)OC)O | 3314 |
| 15 | IMPHY004751 | 3-(Methylthio)propionaldehyde | CSCCC=O | 18635 |
| 16 | IMPHY006037 | 2-Methylbutyraldehyde | CC(C=O)CC | 7284 |
| 17 | IMPHY006138 | Anisole | COc1ccccc1 | 7519 |
| 18 | IMPHY006145 | p-Cymene | Cc1ccc(cc1)C(C)C | 7463 |
| 19 | IMPHY006177 | Methyl geranate | COC(=O)/C=C(/CCC=C(C)C)C | 5365910 |
| 20 | IMPHY006279 | 2-Phenylethanol | OCCc1ccccc1 | 6054 |
| 21 | IMPHY006347 | Hexanal | CCCCCC=O | 6184 |
| 22 | IMPHY006352 | Nerol oxide | CC(=CC1OCC=C(C1)C)C | 61275 |
| 23 | IMPHY006404 | Damascenone | C/C=C/C(=O)C1=C(C)C=CCC1(C)C | 5366074 |
| 24 | IMPHY006485 | beta-Ionone | CC(=O)/C=C/C1=C(C)CCCC1(C)C | 638014 |
| 25 | IMPHY006574 | Ethyl benzoate | CCOC(=O)c1ccccc1 | 7165 |
| 26 | IMPHY006696 | Methyleugenol | C=CCc1ccc(c(c1)OC)OC | 7127 |
| 27 | IMPHY006700 | Methyl benzoate | COC(=O)c1ccccc1 | 7150 |
| 28 | IMPHY006934 | 2-Methyl-1-butanol | CCC(CO)C | 8723 |
| 29 | IMPHY006951 | Eicosane | CCCCCCCCCCCCCCCCCCCC | 8222 |
| 30 | IMPHY006989 | 2-Pentanone | CCCC(=O)C | 7895 |
| 31 | IMPHY007041 | Furfural | O=Cc1ccco1 | 7362 |
| 32 | IMPHY007083 | Methionine sulfoxide | CS(=O)CCC(C(=O)O)N | 847 |
| 33 | IMPHY007131 | Perillene | CC(=CCCc1cocc1)C | 68316 |
| 34 | IMPHY007171 | 1-Hexanol | CCCCCCO | 8103 |
| 35 | IMPHY007186 | Heptanal | CCCCCCC=O | 8130 |
| 36 | IMPHY007214 | Octyl acetate | CCCCCCCCOC(=O)C | 8164 |
| 37 | IMPHY007302 | Hexyl acetate | CCCCCCOC(=O)C | 8908 |
| 38 | IMPHY007331 | 6-Methyl-5-hepten-2-one | CC(=O)CCC=C(C)C | 9862 |
| 39 | IMPHY007421 | Citronellyl acetate | CC(CCC=C(C)C)CCOC(=O)C | 9017 |
| 40 | IMPHY008146 | 1-Pentanol | CCCCCO | 6276 |
| 41 | IMPHY008946 | delta-Guaiene | CC(=C)[C@@H]1CCC(=C2[C@@H](C1)[C@@H](C)CC2)C | 94275 |
| 42 | IMPHY008972 | Butyl acetate | CCCCOC(=O)C | 31272 |
| 43 | IMPHY008991 | Benzyl acetate | CC(=O)OCc1ccccc1 | 8785 |
| 44 | IMPHY009355 | Tetracosane | CCCCCCCCCCCCCCCCCCCCCCCC | 12592 |
| 45 | IMPHY009359 | Hexacosane | CCCCCCCCCCCCCCCCCCCCCCCCCC | 12407 |
| 46 | IMPHY009368 | Heptadecane | CCCCCCCCCCCCCCCCC | 12398 |
| 47 | IMPHY009369 | Nonadecane | CCCCCCCCCCCCCCCCCCC | 12401 |
| 48 | IMPHY009375 | Docosane | CCCCCCCCCCCCCCCCCCCCCC | 12405 |
| 49 | IMPHY009377 | Pentacosane | CCCCCCCCCCCCCCCCCCCCCCCCC | 12406 |
| 50 | IMPHY009382 | Heneicosane | CCCCCCCCCCCCCCCCCCCCC | 12403 |
| 51 | IMPHY009389 | Pentadecane | CCCCCCCCCCCCCCC | 12391 |
| 52 | IMPHY009490 | Tricosane | CCCCCCCCCCCCCCCCCCCCCCC | 12534 |
| 53 | IMPHY009596 | Pentyl acetate | CCCCCOC(=O)C | 12348 |
| 54 | IMPHY009879 | Hexyl benzoate | CCCCCCOC(=O)c1ccccc1 | 23235 |
| 55 | IMPHY009946 | Benzaldehyde | O=Cc1ccccc1 | 240 |
| 56 | IMPHY009955 | Cyclohexane | C1CCCCC1 | 8078 |
| 57 | IMPHY010072 | Eucalyptol | CC12CCC(CC1)C(O2)(C)C | 2758 |
| 58 | IMPHY010086 | 6-Methylhept-5-en-2-ol | CC(CCC=C(C)C)O | 20745 |
| 59 | IMPHY010097 | Benzyl benzoate | O=C(c1ccccc1)OCc1ccccc1 | 2345 |
| 60 | IMPHY010995 | Toluene | Cc1ccccc1 | 1140 |
| 61 | IMPHY011010 | 1-Nonadecene | CCCCCCCCCCCCCCCCCC=C | 29075 |
| 62 | IMPHY011396 | 4-Carvomenthenol | CC1=CCC(CC1)(O)C(C)C | 11230 |
| 63 | IMPHY011586 | (S,1Z,6Z)-8-Isopropyl-1-methyl-5-methylenecyclodeca-1,6-diene | C/C/1=C/CCC(=C)/C=C[C@@H](CC1)C(C)C | 91723653 |
| 64 | IMPHY011599 | Terpinolene | CC1=CCC(=C(C)C)CC1 | 11463 |
| 65 | IMPHY011632 | Farnesol | OC/C=C(/CC/C=C(/CCC=C(C)C)C)C | 445070 |
| 66 | IMPHY011647 | Geranyl acetate | C/C(=CCOC(=O)C)/CCC=C(C)C | 1549026 |
| 67 | IMPHY011648 | Neryl acetate | C/C(=C/COC(=O)C)/CCC=C(C)C | 1549025 |
| 68 | IMPHY011659 | alpha-Muurolene | CC1=C[C@@H]2[C@H](CC1)C(=CC[C@H]2C(C)C)C | 12306047 |
| 69 | IMPHY011761 | Humulene | C/C/1=CCC(C)(C)/C=C/C/C(=C/CC1)/C | 5281520 |
| 70 | IMPHY011763 | Anethole | C/C=C/c1ccc(cc1)OC | 637563 |
| 71 | IMPHY011789 | Citral | O=C/C=C(/CCC=C(C)C)C | 638011 |
| 72 | IMPHY011790 | Neral | O=C/C=C(CCC=C(C)C)/C | 643779 |
| 73 | IMPHY011804 | cis-3-Hexenyl acetate | CC/C=CCCOC(=O)C | 5363388 |
| 74 | IMPHY011884 | Pulegone | C[C@@H]1CCC(=C(C)C)C(=O)C | 442495 |
| 75 | IMPHY011938 | gamma-Eudesmol | CC1=C2C[C@@H](CC[C@]2(CCC1)C)C(O)(C)C | 6432005 |
| 76 | IMPHY011957 | (+)-delta-Cadinene | CC1=C[C@@H]2C(=C(C)CC[C@H]2C(C)C)CC1 | 441005 |
| 77 | IMPHY011965 | (+)-beta-Phellandrene | CC([C@@H]1CCC(=C)C=C1)C | 442484 |
| 78 | IMPHY012036 | Camphor | O=C1CC2C(C1(C)CC2)(C)C | 2537 |
| 79 | IMPHY012058 | Linalool | C=CC(CCC=C(C)C)(O)C | 6549 |
| 80 | IMPHY012061 | alpha-Pinene | CC1=CCC2CC1C2(C)C | 6654 |
| 81 | IMPHY012070 | o-Xylene | Cc1ccccc1C | 7237 |
| 82 | IMPHY012104 | Citronellol | OCCC(CCC=C(C)C)C | 8842 |
| 83 | IMPHY012147 | beta-Pinene | C=C1CCC2CC1C2(C)C | 14896 |
| 84 | IMPHY012160 | alpha-Terpineol | CC1=CCC(CC1)C(O)(C)C | 17100 |
| 85 | IMPHY012165 | Sabinene | C=C1CCC2(C1C2)C(C)C | 18818 |
| 86 | IMPHY012179 | (2S,4R)-4-methyl-2-(2-methylprop-1-en-1-yl)tetrahydro-2H-pyran | C[C@@H]1CCO[C@@H](C1)C=C(C)C | 1712087 |
| 87 | IMPHY012654 | Nerol | OC/C=C(CCC=C(C)C)/C | 643820 |
| 88 | IMPHY013836 | Fenchone | O=C1C2(C)CCC(C1(C)C)C2 | 14525 |
| 89 | IMPHY014817 | Aromadendrene | CC1CCC2C1C1C(C1(C)C)CCC2=C | 91354 |
| 90 | IMPHY014831 | beta-Caryophyllene | C/C/1=CCCC(=C)[C@@H]2[C@@H](CC1)C(C2)(C)C | 5281515 |
| 91 | IMPHY014835 | (E)-beta-ocimene | C=C/C(=C/CC=C(C)C)/C | 5281553 |
| 92 | IMPHY014923 | Geraniol | OC/C=C(/CCC=C(C)C)C | 637566 |
| 93 | IMPHY014988 | Limonene | CC1=CCC(CC1)C(=C)C | 22311 |
| 94 | IMPHY015003 | Menthol | CC1CCC(C(C1)O)C(C)C | 1254 |
| 95 | IMPHY015004 | Menthone | C[C@@H]1CC[C@H](C(=O)C1)C(C)C | 26447 |
| 96 | IMPHY015022 | Nerolidol | C=CC(CC/C=C(/CCC=C(C)C)C)(O)C | 5284507 |
| 97 | IMPHY015123 | alpha-Copaene | CC([C@@H]1CC[C@]2([C@@H]3[C@H]1C2C(=CC3)C)C)C | 70678558 |
| 98 | IMPHY015376 | 4-(Prop-1-en-2-yl)benzaldehyde | O=Cc1ccc(cc1)C(=C)C | 14597914 |
| 99 | IMPHY015751 | 3-Methylbutyl pentanoate | CCCCC(=O)OCCC(C)C | 74901 |
| 100 | IMPHY017272 | Chlorodibromomethane | ClC(Br)Br | 31296 |
| 101 | IMPHY017472 | Bromoform | BrC(Br)Br | 5558 |
| 102 | IMPHY007083 | Methionine sulfoxide | CS(=O)CCC(C(=O)O)N | 847 |
| 103 | IMPHY002915 | Benzyl Alcohol | OCc1ccccc1 | 244 |
| 104 | IMPHY003485 | Myrcene | C=CC(=C)CCC=C(C)C | 31253 |
| 105 | IMPHY003536 | Eugenol | C=CCc1ccc(c(c1)OC)O | 3314 |
| 106 | IMPHY004536 | Geranic acid | CC(=CCC/C(=C/C(=O)O)/C)C | 5275520 |
| 107 | IMPHY006279 | 2-Phenylethanol | OCCc1ccccc1 | 6054 |
| 108 | IMPHY007421 | Citronellyl acetate | CC(CCC=C(C)C)CCOC(=O)C | 9017 |
| 109 | IMPHY011647 | Geranyl acetate | C/C(=CCOC(=O)C)/CCC=C(C)C | 1549026 |
| 110 | IMPHY011789 | Citral | O=C/C=C(/CCC=C(C)C)C | 638011 |
| 111 | IMPHY012058 | Linalool | C=CC(CCC=C(C)C)(O)C | 6549 |
| 112 | IMPHY012061 | alpha-Pinene | CC1=CCC2CC1C2(C)C | 6654 |
| 113 | IMPHY012104 | Citronellol | OCCC(CCC=C(C)C)C | 8842 |
| 114 | IMPHY012147 | beta-Pinene | C=C1CCC2CC1C2(C)C | 14896 |
| 115 | IMPHY012524 | Cyanin | OC[C@H]1O[C@@H](Oc2cc(O)cc3c2cc(O[C@@H]2O[C@H](CO)[C@H]([C@@H] | 441688 |
| 116 | IMPHY012654 | Nerol | OC/C=C(CCC=C(C)C)/C | 643820 |
| 117 | IMPHY014923 | Geraniol | OC/C=C(/CCC=C(C)C)C | 637566 |
| 118 | IMPHY015054 | Quercitrin | Oc1cc(O)c2c(c1)oc(c(c2=O)O[C@@H]1O[C@@H](C)[C@@H]([C@H]([C@H]1O)O)O)c1ccc(c(c1)O)O | 5280459 |
| **PLANT NAME : *Rosmarinus officinalis*** | | | | |
| **Serial No** | **IMPPAT Phytochemical Identifier** | **Phytochemical Name** | **SMILES** | **CID** |
| 1 | IMPHY000060 | Myristic acid | CCCCCCCCCCCCCC(=O)O | 11005 |
| 2 | IMPHY000099 | Myrtenol | OCC1=CCC2CC1C2(C)C | 10582 |
| 3 | IMPHY000308 | Hexadecane | CCCCCCCCCCCCCCCC | 11006 |
| 4 | IMPHY000399 | beta-Bisabolene | CC(=CCCC(=C)[C@H]1CCC(=CC1)C)C | 10104370 |
| 5 | IMPHY000812 | Triterpenoid | O[C@H]1CC[C@]2([C@H]([C@]1(C)COS(=O)(=O)O)CC[C@@]1([C@@H]2CC=C2[C@@]1(C)CC[C@@]1([C@H]2CC(C)(C)CC1)C(=O)O)C)C | 451674 |
| 6 | IMPHY000915 | Chrysanthenone | CC1=CCC2C(=O)C1C2(C)C | 442463 |
| 7 | IMPHY001144 | Dillapiol | C=CCc1cc2OCOc2c(c1OC)OC | 10231 |
| 8 | IMPHY001246 | Carvacrol | CC(c1ccc(c(c1)O)C)C | 10364 |
| 9 | IMPHY001351 | Elemicin | C=CCc1cc(OC)c(c(c1)OC)OC | 10248 |
| 10 | IMPHY001548 | Geranylacetone | C/C=C(/C)C, CC(C)=O, CC=C(C)C | 1549778 |
| 11 | IMPHY001658 | Thymol methyl ether | COc1cc(C)ccc1C(C)C | 14104 |
| 12 | IMPHY001828 | 3-Octano | CCCCCC(CC)O | 11527 |
| 13 | IMPHY001915 | Octadecane | CCCCCCCCCCCCCCCCCC | 11635 |
| 14 | IMPHY002072 | Pinocarvone | C=C1C(=O)CC2CC1C2(C)C | 121719 |
| 15 | IMPHY002825 | 2-(4-Methylphenyl)propan-2-ol | Cc1ccc(cc1)C(O)(C)C | 14529 |
| 16 | IMPHY002983 | 1-Hexadecanol | CCCCCCCCCCCCCCCCO | 2682 |
| 17 | IMPHY003296 | Piperitenone | CC1=CC(=O)C(=C(C)C)CC1 | 381152 |
| 18 | IMPHY003485 | Myrcene | C=CC(=C)CCC=C(C)C | 31253 |
| 19 | IMPHY003536 | Eugenol | C=CCc1ccc(c(c1)OC)O | 3314 |
| 20 | IMPHY003545 | 4-Isopropylbenzaldehyde | O=Cc1ccc(cc1)C(C)C | 326 |
| 21 | IMPHY003559 | beta-Amyrone | O=C1CC[C@]2([C@H](C1(C)C)CC[C@@]1([C@@H]2CC=C2[C@@]1(C)CC[C@@]1([C@H]2CC(C)(C)CC1)C)C)C | 12306160 |
| 22 | IMPHY003649 | 2-Heptenal | CCCC/C=C/C=O | 5283316 |
| 23 | IMPHY003691 | Methyl jasmonate | CC/C=CC[C@@H]1[C@H](CCC1=O)CC(=O)OC | 5281929 |
| 24 | IMPHY003956 | (+)-gamma-Gurjunene | C[C@@H]1CC[C@H]2C1=C[C@@H](CC[C@H]2C)C(=C)C | 15560285 |
| 25 | IMPHY003977 | (-)-beta-Bourbonene | CC([C@@H]1CC[C@@]2([C@H]1[C@H]1C(=C)CC[C@@H]21)C)C | 62566 |
| 26 | IMPHY003982 | gamma-Terpinene | CC1=CCC(=CC1)C(C)C | 7461 |
| 27 | IMPHY004017 | Manool | C=C[C@@](CC[C@H]1C(=C)CC[C@@H]2[C@]1(C)CCCC2(C)C)(O)C | 3034394 |
| 28 | IMPHY004077 | Verbenone | CC1=CC(=O)C2CC1C2(C)C | 29025 |
| 29 | IMPHY005196 | 7-O-Methylrosmanol | CO[C@@H]1[C@H]2OC(=O)[C@]3([C@@H]2C(C)(C)CCC3)c2c1cc(C(C)C)c(c2O)O | 23243692 |
| 30 | IMPHY005327 | Cirsimaritin | COc1cc2oc(cc(=O)c2c(c1OC)O)c1ccc(cc1)O | 188323 |
| 31 | IMPHY005345 | 1-Octen-3-OL | CCCCCC(C=C)O | 18827 |
| 32 | IMPHY005400 | Sclareol | C=C[C@@](CC[C@H]1[C@](C)(O)CC[C@@H]2[C@]1(C)CCCC2(C)C)(O)C | 163263 |
| 33 | IMPHY006138 | Anisole | COc1ccccc1 | 7519 |
| 34 | IMPHY006145 | p-Cymene | Cc1ccc(cc1)C(C)C | 7463 |
| 35 | IMPHY006300 | Cholesterol | CC(CCC[C@H]([C@H]1CC[C@@H]2[C@]1(C)CC[C@H]1[C@H]2CC=C2[C@]1(C)CC[C@@H](C2)O)C)C | 5997 |
| 36 | IMPHY006324 | Linalyl propionate | CCC(=O)OC(CCC=C(C)C)(C=C)C | 61098 |
| 37 | IMPHY006325 | Myrtenal | O=CC1=CCC2CC1C2(C)C | 61130 |
| 38 | IMPHY006337 | 1-Octen-3-one | CCCCCC(=O)C=C | 61346 |
| 39 | IMPHY006550 | Thymol | Cc1ccc(c(c1)O)C(C)C | 6989 |
| 40 | IMPHY006696 | Methyleugenol | C=CCc1ccc(c(c1)OC)OC | 7127 |
| 41 | IMPHY006898 | Stearyl alcohol | CCCCCCCCCCCCCCCCCCO | 8221 |
| 42 | IMPHY006925 | 1-Octadecene | CCCCCCCCCCCCCCCCC=C | 8217 |
| 43 | IMPHY006944 | Estragole | COc1ccc(cc1)CC=C | 8815 |
| 44 | IMPHY006948 | beta-Terpineol | CC(=C)C1CCC(CC1)(C)O | 8748 |
| 45 | IMPHY006950 | Tricyclene | CC12C3C1CC(C2(C)C)C3 | 79035 |
| 46 | IMPHY006951 | Eicosane | CCCCCCCCCCCCCCCCCCCC | 8222 |
| 47 | IMPHY007067 | Linalyl acetate | C=CC(OC(=O)C)(CCC=C(C)C)C | 8294 |
| 48 | IMPHY007201 | Carvacrol methyl ether | COc1cc(ccc1C)C(C)C | 80790 |
| 49 | IMPHY007276 | Nonan-1-ol | CCCCCCCCCO | 8914 |
| 50 | IMPHY007327 | Palmitic acid | CCCCCCCCCCCCCCCC(=O)O | 985 |
| 51 | IMPHY007539 | Phenylacetaldehyde | O=CCc1ccccc1 | 998 |
| 52 | IMPHY007747 | Carvacryl acetate | CC(=O)Oc1cc(ccc1C)C(C)C | 80792 |
| 53 | IMPHY007840 | Spathulenol | C=C1CC[C@@H]2[C@H]([C@H]3[C@H]1CC[C@]3(C)O)C2(C)C | 92231 |
| 54 | IMPHY008150 | 1-Methyl-4-(prop-1-en-2-yl)benzene | Cc1ccc(cc1)C(=C)C | 62385 |
| 55 | IMPHY009212 | Rofficerone | C[C@@H]1[C@H]2C3=CC[C@H]4[C@@]([C@]3(C)CC[C@@]2(C)CC[C@]1(C)O)(C)CC[C@@H]1[C@]4(C)CCC(=O)C1(C)C | 101763940 |
| 56 | IMPHY009355 | Tetracosane | CCCCCCCCCCCCCCCCCCCCCCCC | 12592 |
| 57 | IMPHY009367 | 1-Pentadecanol | CCCCCCCCCCCCCCCO | 12397 |
| 58 | IMPHY009368 | Heptadecane | CCCCCCCCCCCCCCCCC | 12398 |
| 59 | IMPHY009369 | Nonadecane | CCCCCCCCCCCCCCCCCCC | 12401 |
| 60 | IMPHY009377 | Pentacosane | CCCCCCCCCCCCCCCCCCCCCCCCC | 12406 |
| 61 | IMPHY009490 | Tricosane | CCCCCCCCCCCCCCCCCCCCCCC | 12534 |
| 62 | IMPHY009742 | beta-Oplopenone | CC([C@@H]1CCC(=C)[C@H]2[C@H]1[C@H](CC2)C(=O)C)C | 14038847 |
| 63 | IMPHY009743 | beta-Gurjunene | C[C@@H]1CC[C@@H]2[C@H]([C@H]3[C@@H]1CCC3=C)C2(C)C | 6450812 |
| 64 | IMPHY009763 | Bornyl formate | O=COC1CC2C(C1(C)CC2)(C)C | 518472 |
| 65 | IMPHY009778 | 1-Isopropyl-4-methylenebicyclo[3.1.0]hex-2-ene | CC(C12C=CC(=C)C2C1)C | 524198 |
| 66 | IMPHY010000 | Dodecane | CCCCCCCCCCCC | 8182 |
| 67 | IMPHY010072 | Eucalyptol | CC12CCC(CC1)C(O2)(C)C | 2758 |
| 68 | IMPHY010080 | beta-Elemene | C=C[C@]1(C)CC[C@H](C[C@H]1C(=C)C)C(=C)C | 6918391 |
| 69 | IMPHY010579 | 1-Eicosene | CCCCCCCCCCCCCCCCCCC=C | 18936 |
| 70 | IMPHY010781 | Limonene oxide, cis-(-)- | CC(=C)[C@H]1CC[C@]2([C@@H](C1)O2)C | 6452061 |
| 71 | IMPHY010887 | Thymol acetate | CC(=O)Oc1cc(C)ccc1C(C)C | 68252 |
| 72 | IMPHY011051 | Abietatriene | CC(c1ccc2c(c1)CC[C@@H]1[C@]2(C)CCCC1(C)C)C | 6432211 |
| 73 | IMPHY011079 | 4,8-Methanoazulen-9-ol, decahydro-2,2,4,8-tetramethyl-, stereoisomer | OC1C2(C)CCCC1(C)C1C2CC(C1)(C)C | 521185 |
| 74 | IMPHY011381 | (4S)-1-Methyl-4-(prop-1-EN-2-YL)-7-oxabicyclo[4.1.0]heptane | CC(=C)[C@H]1CCC2(C(C1)O2)C | 10953718 |
| 75 | IMPHY011392 | 3-Carene | CC1=CCC2C(C1)C2(C)C | 26049 |
| 76 | IMPHY011396 | 4-Carvomenthenol | CC1=CCC(CC1)(O)C(C)C | 11230 |
| 77 | IMPHY011407 | alpha-Campholenal | O=CC[C@H]1CC=C(C1(C)C)C | 1252759 |
| 78 | IMPHY011519 | alpha-Terpinyl acetate | CC(=O)OC(C1CCC(=CC1)C)(C)C | 111037 |
| 79 | IMPHY011542 | beta-Eudesmol | C=C1CCC[C@]2([C@H]1C[C@@H](CC2)C(O)(C)C)C | 91457 |
| 80 | IMPHY011552 | (1R)-2-methyl-5-propan-2-ylbicyclo[3.1.0]hex-2-ene | CC1=CCC2([C@@H]1C2)C(C)C | 6451618 |
| 81 | IMPHY011562 | 2-Hexenal | CCC/C=C/C=O | 5281168 |
| 82 | IMPHY011568 | alpha-Fenchyl acetate | CC(=O)O[C@@H]1[C@]2(C)CC[C@H](C1(C)C)C2 | 7530950 |
| 83 | IMPHY011586 | (S,1Z,6Z)-8-Isopropyl-1-methyl-5-methylenecyclodeca-1,6-diene | C/C/1=C/CCC(=C)/C=C[C@@H](CC1)C(C)C | 91723653 |
| 84 | IMPHY011590 | d-Borneol | O[C@@H]1C[C@H]2C([C@@]1(C)CC2)(C)C | 61060 |
| 85 | IMPHY011599 | Terpinolene | CC1=CCC(=C(C)C)CC1 | 11463 |
| 86 | IMPHY011643 | alpha-Terpinene | CC1=CC=C(CC1)C(C)C | 7462 |
| 87 | IMPHY011647 | Geranyl acetate | C/C(=CCOC(=O)C)/CCC=C(C)C | 1549026 |
| 88 | IMPHY011648 | Neryl acetate | C/C(=C/COC(=O)C)/CCC=C(C)C | 1549025 |
| 89 | IMPHY011659 | alpha-Muurolene | CC1=C[C@@H]2[C@H](CC1)C(=CC[C@H]2C(C)C)C | 12306047 |
| 90 | IMPHY011660 | (+)-alpha-Cadinene | CC1=C[C@@H]2[C@@H](CC1)C(=CC[C@H]2C(C)C)C | 12306048 |
| 91 | IMPHY011709 | alpha-Eudesmol | CC1=CCC[C@]2([C@H]1C[C@@H](CC2)C(O)(C)C)C | 92762 |
| 92 | IMPHY011714 | Methyl cinnamate | COC(=O)/C=C/c1ccccc1 | 637520 |
| 93 | IMPHY011761 | Humulene | C/C/1=CCC(C)(C)/C=C/C/C(=C/CC1)/C | 5281520 |
| 94 | IMPHY011778 | (2Z,6Z)-Farnesyl acetate | C/C(=C/CC/C(=CCOC(=O)C)/C)/CCC=C(C)C | 1551479 |
| 95 | IMPHY011789 | Citral | O=C/C=C(/CCC=C(C)C)C | 638011 |
| 96 | IMPHY011790 | Neral | O=C/C=C(CCC=C(C)C)/C | 643779 |
| 97 | IMPHY011792 | gamma-Muurolene | CC1=C[C@@H]2[C@H](CC1)C(=C)CC[C@H]2C(C)C | 12313020 |
| 98 | IMPHY011793 | (+)-gamma-Cadinene | CC1=C[C@@H]2[C@@H](CC1)C(=C)CC[C@H]2C(C)C | 6432404 |
| 99 | IMPHY011811 | (Z)-Methyl cinnamate | COC(=O)/C=Cc1ccccc1 | 6428458 |
| 100 | IMPHY011817 | alpha-Farnesene | C=C/C(=C/C/C=C(/CCC=C(C)C)C)/C | 5281516 |
| 101 | IMPHY011839 | (Z)-gamma-bisabolene | CC(=CCC/C(=C1/CCC(=CC1)C)/C)C | 3033866 |
| 102 | IMPHY011884 | Pulegone | C[C@@H]1CCC(=C(C)C)C(=O)C1 | 442495 |
| 103 | IMPHY011896 | Valencene | CC(=C)[C@@H]1CCC2=CCC[C@H]([C@@]2(C1)C)C | 9855795 |
| 104 | IMPHY011901 | Thujone | O=C1C[C@]2([C@@H]([C@H]1C)C2)C(C)C | 261491 |
| 105 | IMPHY011938 | gamma-Eudesmol | CC1=C2C[C@@H](CC[C@]2(CCC1)C)C(O)(C)C | 6432005 |
| 106 | IMPHY011957 | (+)-delta-Cadinene | CC1=C[C@@H]2C(=C(C)CC[C@H]2C(C)C)CC1 | 441005 |
| 107 | IMPHY011965 | (+)-beta-Phellandrene | CC([C@@H]1CCC(=C)C=C1)C | 442484 |
| 108 | IMPHY012036 | Camphor | O=C1CC2C(C1(C)CC2)(C)C | 2537 |
| 109 | IMPHY012058 | Linalool | C=CC(CCC=C(C)C)(O)C | 6549 |
| 110 | IMPHY012061 | alpha-Pinene | CC1=CCC2CC1C2(C)C | 6654 |
| 111 | IMPHY012075 | Carvone | CC(=C)C1CC=C(C(=O)C1)C | 7439 |
| 112 | IMPHY012086 | Citronellal | O=CCC(CCC=C(C)C)C | 7794 |
| 113 | IMPHY012104 | Citronellol | OCCC(CCC=C(C)C)C | 8842 |
| 114 | IMPHY012130 | Dihydrocarveol | CC(=C)C1CCC(C(C1)O)C | 12072 |
| 115 | IMPHY012147 | beta-Pinene | C=C1CCC2CC1C2(C)C | 14896 |
| 116 | IMPHY012152 | alpha-Fenchol | O[C@H]1[C@@]2(C)CC[C@@H](C1(C)C)C2 | 439711 |
| 117 | IMPHY012160 | alpha-Terpineol | CC1=CCC(CC1)C(O)(C)C | 17100 |
| 118 | IMPHY012165 | Sabinene | C=C1CCC2(C1C2)C(C)C | 18818 |
| 119 | IMPHY012168 | (1S,2S,6S,7R,8R)-1,3-dimethyl-8-propan-2-yltricyclo[4.4.0.02,7]dec-3-ene | CC([C@H]1CC[C@]2([C@@H]3[C@@H]1[C@H]2C(=CC3)C)C)C | 101607926 |
| 120 | IMPHY012205 | Sabinene hydrate | CC(C12CCC(C2C1)(C)O)C | 62367 |
| 121 | IMPHY012249 | 1-Nonadecanol | CCCCCCCCCCCCCCCCCCCO | 80281 |
| 122 | IMPHY012255 | (+)-trans-Piperitenol | CC1=C[C@@H]([C@H](CC1)C(C)C)O | 85568 |
| 123 | IMPHY012265 | (1r,3s,5r)-6,6-Dimethyl-2-methylidenebicyclo[3.1.1]heptan-3-ol | C=C1[C@@H](O)C[C@H]2C[C@@H]1C2(C)C | 88302 |
| 124 | IMPHY012402 | Campesterol | O[C@H]1CC[C@]2(C(=CC[C@@H]3[C@@H]2CC[C@]2([C@H]3CC[C@@H]2[C@@H](CC[C@H](C(C)C)C)C)C)C1)C | 173183 |
| 125 | IMPHY012473 | Lupeol | CC(=C)[C@@H]1CC[C@]2([C@H]1[C@H]1CC[C@H]3[C@@]([C@]1(C)CC2)(C)CC[C@@H]1[C@]3(C)CC[C@@H](C1(C)C)O)C | 259846 |
| 126 | IMPHY012585 | delta-Cadinol | CC1=C[C@@H]2[C@H](CC1)[C@](C)(O)CC[C@H]2C(C)C | 3084311 |
| 127 | IMPHY012654 | Nerol | OC/C=C(CCC=C(C)C)/C | 643820 |
| 128 | IMPHY012665 | Levomenol | CC(=CCC[C@@]([C@H]1CCC(=CC1)C)(O)C)C | 442343 |
| 129 | IMPHY012667 | Caryophyllene oxide | C=C1CC[C@H]2O[C@@]2(CC[C@@H]2[C@@H]1CC2(C)C)C | 1742210 |
| 130 | IMPHY012712 | Phytol | OC/C=C(/CCC[C@@H](CCC[C@@H](CCCC(C)C)C)C)C | 5280435 |
| 131 | IMPHY012739 | (Z)-beta-Ocimene | C=C/C(=CCC=C(C)C)/C | 5320250 |
| 132 | IMPHY012920 | 2-Furanmethanol, 5-ethenyltetrahydro-alpha,alpha,5-trimethyl-, cis- | C=C[C@@]1(C)CC[C@H](O1)C(O)(C)C | 11116492 |
| 133 | IMPHY012921 | gamma-Elemene | C=C[C@]1(C)CCC(=C(C)C)C[C@H]1C(=C)C | 6432312 |
| 134 | IMPHY013080 | alpha-Calacorene | CC([C@@H]1CC=C(c2c1cc(C)cc2)C)C | 12302243 |
| 135 | IMPHY013133 | (Z)-p-Menth-2-en-1-ol | CC([C@@H]1CC[C@](C=C1)(C)O)C | 13918681 |
| 136 | IMPHY013836 | Fenchone | O=C1C2(C)CCC(C1(C)C)C2 | 14525 |
| 137 | IMPHY014690 | (-)-Globulol | C[C@@H]1CC[C@@H]2[C@@H]1[C@H]1[C@H](C1(C)C)CC[C@@]2(C)O | 12304985 |
| 138 | IMPHY014708 | beta-Selinene | C=C1CCC[C@]2([C@H]1C[C@@H](CC2)C(=C)C)C | 442393 |
| 139 | IMPHY014801 | Zizanene | CC1=C[C@@H]2[C@H](CC1)C(=CC[C@@H]2C(C)C)C | 12306046 |
| 140 | IMPHY014806 | Caswell No. 264AB | CC([C@@H]1CC[C@H]([C@]23[C@H]1[C@H]2C(=CC3)C)C)C | 442359 |
| 141 | IMPHY014811 | alpha-Phellandrene | CC1=CCC(C=C1)C(C)C | 7460 |
| 142 | IMPHY014817 | Aromadendrene | CC1CCC2C1C1C(C1(C)C)CCC2=C | 91354 |
| 143 | IMPHY014831 | beta-Caryophyllene | C/C/1=CCCC(=C)[C@@H]2[C@@H](CC1)C(C2)(C)C | 5281515 |
| 144 | IMPHY014835 | (E)-beta-ocimen | C=C/C(=C/CC=C(C)C)/C | 5281553 |
| 145 | IMPHY014842 | Stigmasterol | CC[C@@H](C(C)C)/C=C/[C@H]([C@H]1CC[C@@H]2[C@]1(C)CC[C@H]1[C@H]2CC=C2[C@]1(C)CC[C@@H](C2)O)C | 5280794 |
| 146 | IMPHY014847 | Bornyl acetate | CC(=O)OC1CC2C(C1(C)CC2)(C)C | 6448 |
| 147 | IMPHY014852 | Camphene | C=C1C2CCC(C1(C)C)C2 | 6616 |
| 148 | IMPHY014865 | Calamenene | CC([C@@H]1CC[C@@H](c2c1cc(C)cc2)C)C | 6429077 |
| 149 | IMPHY014869 | Isomenthone | C[C@H]1CC[C@H](C(=O)C1)C(C)C | 6432469 |
| 150 | IMPHY014873 | 2-Cyclohexen-1-ol, 3-methyl-6-(1-methylethyl)-, (1R,6S)-rel- | CC1=C[C@@H]([C@@H](CC1)C(C)C)O | 85567 |
| 151 | IMPHY014874 | cis-Sabinene hydrate | C[C@@H]1CC[C@@]2(C1C2)C(C)C | 101629835 |
| 152 | IMPHY014877 | (S)-cis-Verbenol | CC1=C[C@H](O)[C@H]2C[C@@H]1C2(C)C | 87839 |
| 153 | IMPHY014885 | 1-Isopropyl-4,7-dimethyl-1,3,4,5,6,8a-hexahydro-4a(2H)-naphthalenol | CC1=CC2C(CC1)(O)C(C)CCC2C(C)C | 519857 |
| 154 | IMPHY014906 | Cedrelanol | CC1=C[C@@H]2[C@@H](CC1)[C@@](C)(O)CC[C@H]2C(C)C | 160799 |
| 155 | IMPHY014923 | Geraniol | OC/C=C(/CCC=C(C)C)C | 637566 |
| 156 | IMPHY014938 | iso-Isopulegol | C[C@@H]1CC[C@@H]([C@H](C1)O)C(=C)C | 11008146 |
| 157 | IMPHY014986 | Ledol | C[C@@H]1CC[C@H]2[C@@H]1[C@H]1[C@H](C1(C)C)CC[C@@]2(C)O | 92812 |
| 158 | IMPHY014988 | Limonene | CC1=CCC(CC1)C(=C)C | 22311 |
| 159 | IMPHY014989 | trans-Linalool oxide | C=C[C@]1(C)CC[C@H](O1)C(O)(C)C | 6432254 |
| 160 | IMPHY015003 | Menthol | CC1CCC(C(C1)O)C(C)C | 1254 |
| 161 | IMPHY015004 | Menthone | C[C@@H]1CC[C@H](C(=O)C1)C(C)C | 26447 |
| 162 | IMPHY015011 | Methylisoeugenol | C/C=C/c1ccc(c(c1)OC)OC | 637776 |
| 163 | IMPHY015038 | (-)-Phyllocladene | C=C1C[C@]23C[C@@H]1CC[C@H]3[C@]1([C@H](CC2)C(C)(C)CCC1)C | 44559813 |
| 164 | IMPHY015040 | 3-Pinanone | O=C1CC2CC(C1C)C2(C)C | 11038 |
| 165 | IMPHY015042 | Piperitone | CC1=CC(=O)C(CC1)C(C)C | 6987 |
| 166 | IMPHY015081 | Taraxasterol | C=C1CC[C@]2([C@H]([C@@H]1C)[C@H]1CC[C@H]3[C@@]([C@@]1(CC2)C)(C)CC[C@@H]1[C@]3(C)CC[C@@H](C1(C)C)O)C | 115250 |
| 167 | IMPHY015098 | trans-Verbenol | CC1=C[C@H](O)[C@@H]2C[C@H]1C2(C)C | 89664 |
| 168 | IMPHY015123 | alpha-Copaene | CC([C@@H]1CC[C@]2([C@@H]3[C@H]1C2C(=CC3)C)C)C | 70678558 |
| 169 | IMPHY015128 | T-Muurolol | CC1=C[C@@H]2[C@H](CC1)[C@@](C)(O)CC[C@H]2C(C)C | 3084331 |
| 170 | IMPHY016012 | Allo-Aromadendrene | C[C@@H]1CC[C@H]2[C@@H]1C1C(C1(C)C)CCC2=C | 42608158 |
| 171 | IMPHY016014 | Isobornyl acetate | CC(=O)O[C@H]1C[C@@H]2C([C@]1(C)CC2)(C)C | 247573 |
| 172 | IMPHY016027 | trans-Sabinene hydrate | CC([C@@]12CC[C@](C2C1)(C)O)C | 12315151 |
| 173 | IMPHY016054 | trans-alpha-Bergamotene | CC(=CCC[C@]1(C)[C@H]2CC=C([C@@H]1C2)C)C | 6429302 |
| 174 | IMPHY016578 | cis-Piperitol, acetate | CC(=O)O[C@H]1C=C(C)CC[C@H]1C(C)C | 6427492 |
| 175 | IMPHY000070 | Cadalene | Cc1ccc2c(c1)c(ccc2C)C(C)C | 10225 |
| 176 | IMPHY000399 | beta-Bisabolene | CC(=CCCC(=C)[C@H]1CCC(=CC1)C)C | 10104370 |
| 177 | IMPHY001246 | Carvacrol | CC(c1ccc(c(c1)O)C)C | 10364 |
| 178 | IMPHY001548 | Geranylacetone | C/C(=CCCC(=O)C)/CCC=C(C)C | 1549778 |
| 179 | IMPHY002072 | Pinocarvone | C=C1C(=O)CC2CC1C2(C)C | 121719 |
| 180 | IMPHY003179 | 3-Octanone | CCCCCC(=O)CC | 246728 |
| 181 | IMPHY003296 | Piperitenone | CC1=CC(=O)C(=C(C)C)CC1 | 381152 |
| 182 | IMPHY003485 | Myrcene | C=CC(=C)CCC=C(C)C | 31253 |
| 183 | IMPHY003536 | Eugenol | C=CCc1ccc(c(c1)OC)O | 3314 |
| 184 | IMPHY003567 | alpha-Fenchene | C=C1CC2C(C1CC2)(C)C | 28930 |
| 185 | IMPHY003691 | Methyl jasmonate | CC/C=CC[C@@H]1[C@H](CCC1=O)CC(=O)OC | 5281929 |
| 186 | IMPHY003807 | (E)-alpha-bisabolene | CC(=CC/C=C(/C1CCC(=CC1)C)C)C | 5315468 |
| 187 | IMPHY003982 | gamma-Terpinene | CC1=CCC(=CC1)C(C)C | 7461 |
| 188 | IMPHY004077 | Verbenone | CC1=CC(=O)C2CC1C2(C)C | 29025 |
| 189 | IMPHY004971 | alpha-Bourbonene | CC(C1CCC2(C1C1C(=CCC21)C)C)C | 530816 |
| 190 | IMPHY005345 | 1-Octen-3-OL | CCCCCC(C=C)O | 18827 |
| 191 | IMPHY006145 | p-Cymene | Cc1ccc(cc1)C(C)C | 7463 |
| 192 | IMPHY006550 | Thymol | Cc1ccc(c(c1)O)C(C)C | 6989 |
| 193 | IMPHY006696 | Methyleugenol | C=CCc1ccc(c(c1)OC)OC | 7127 |
| 194 | IMPHY006950 | Tricyclene | CC12C3C1CC(C2(C)C)C3 | 79035 |
| 195 | IMPHY006965 | alpha,alpha-Dimethyl-4-methylenecyclohexanemethanol | CC(C1CCC(=C)CC1)(O)C | 81722 |
| 196 | IMPHY007067 | Linalyl acetate | C=CC(OC(=O)C)(CCC=C(C)C)C | 8294 |
| 197 | IMPHY007376 | beta-Cubebene | CC([C@@H]1CC[C@H]([C@]23[C@H]1[C@H]2C(=C)CC3)C)C | 93081 |
| 198 | IMPHY007520 | Viridiflorene | C[C@@H]1CCC2=C(C)CC[C@@H]3[C@H]([C@H]12)C3(C)C | 10910653 |
| 199 | IMPHY007840 | Spathulenol | C=C1CC[C@@H]2[C@H]([C@H]3[C@H]1CC[C@]3(C)O)C2(C)C | 92231 |
| 200 | IMPHY009729 | Verbenene | C=C1C=CC2CC1C2(C)C | 6427476 |
| 201 | IMPHY010072 | Eucalyptol | CC12CCC(CC1)C(O2)(C)C | 2758 |
| 202 | IMPHY011392 | 3-Carene | CC1=CCC2C(C1)C2(C)C | 26049 |
| 203 | IMPHY011396 | 4-Carvomenthenol | CC1=CCC(CC1)(O)C(C)C | 11230 |
| 204 | IMPHY011455 | Cadina-1,4-diene | CC1=CC2C(=CC1)[C@@H](C)CCC2C(C)C | 6427091 |
| 205 | IMPHY011542 | beta-Eudesmol | C=C1CCC[C@]2([C@H]1C[C@@H](CC2)C(O)(C)C)C | 91457 |
| 206 | IMPHY011552 | (1R)-2-methyl-5-propan-2-ylbicyclo[3.1.0]hex-2-ene | CC1=CCC2([C@@H]1C2)C(C)C | 6451618 |
| 207 | IMPHY011586 | (S,1Z,6Z)-8-Isopropyl-1-methyl-5-methylenecyclodeca-1,6-diene | C/C/1=C/CCC(=C)/C=C[C@@H](CC1)C(C)C | 91723653 |
| 208 | IMPHY011590 | d-Borneol | O[C@@H]1C[C@H]2C([C@@]1(C)CC2)(C)C | 61060 |
| 209 | IMPHY011599 | Terpinolene | CC1=CCC(=C(C)C)CC1 | 11463 |
| 210 | IMPHY011643 | alpha-Terpinene | CC1=CC=C(CC1)C(C)C | 7462 |
| 211 | IMPHY011647 | Geranyl acetate | C/C(=CCOC(=O)C)/CCC=C(C)C | 1549026 |
| 212 | IMPHY011658 | beta-Farnesene | C=CC(=C)CC/C=C(/CCC=C(C)C)C | 5281517 |
| 213 | IMPHY011659 | alpha-Muurolene | CC1=C[C@@H]2[C@H](CC1)C(=CC[C@H]2C(C)C)C | 12306047 |
| 214 | IMPHY011761 | Humulene | C/C/1=CCC(C)(C)/C=C/C/C(=C/CC1)/C | 5281520 |
| 215 | IMPHY011792 | gamma-Muurolene | CC1=C[C@@H]2[C@H](CC1)C(=C)CC[C@H]2C(C)C | 12313020 |
| 216 | IMPHY011793 | (+)-gamma-Cadinene | CC1=C[C@@H]2[C@@H](CC1)C(=C)CC[C@H]2C(C)C | 6432404 |
| 217 | IMPHY011817 | alpha-Farnesene | C=C/C(=C/C/C=C(/CCC=C(C)C)C)/C | 5281516 |
| 218 | IMPHY011902 | beta-Thujone | O=C1C[C@]2([C@@H]([C@@H]1C)C2)C(C)C | 91456 |
| 219 | IMPHY011957 | (+)-delta-Cadinene | CC1=C[C@@H]2C(=C(C)CC[C@H]2C(C)C)CC1 | 441005 |
| 220 | IMPHY011965 | (+)-beta-Phellandrene | CC([C@@H]1CCC(=C)C=C1)C | 442484 |
| 221 | IMPHY011999 | Carveol | CC(=C)C1CC=C(C(C1)O)C | 7438 |
| 222 | IMPHY012036 | Camphor | O=C1CC2C(C1(C)CC2)(C)C | 2537 |
| 223 | IMPHY012058 | Linalool | C=CC(CCC=C(C)C)(O)C | 6549 |
| 224 | IMPHY012061 | alpha-Pinene | CC1=CCC2CC1C2(C)C | 6654 |
| 225 | IMPHY012075 | Carvone | CC(=C)C1CC=C(C(=O)C1)C | 7439 |
| 226 | IMPHY012147 | beta-Pinene | C=C1CCC2CC1C2(C)C | 14896 |
| 227 | IMPHY012160 | alpha-Terpineol | CC1=CCC(CC1)C(O)(C)C | 17100 |
| 228 | IMPHY012165 | Sabinene | C=C1CCC2(C1C2)C(C)C | 18818 |
| 229 | IMPHY012168 | (1S,2S,6S,7R,8R)-1,3-dimethyl-8-propan-2-yltricyclo[4.4.0.02,7]dec-3-ene | CC([C@H]1CC[C@]2([C@@H]3[C@@H]1[C@H]2C(=CC3)C)C)C | 101607926 |
| 230 | IMPHY012198 | Verbenol | CC1=CC(O)C2CC1C2(C)C | 61126 |
| 231 | IMPHY012254 | Isopinocamphone | O=C1C[C@H]2C[C@@H]([C@@H]1C)C2(C)C | 84532 |
| 232 | IMPHY012279 | alpha-Curcumene | CC(=CCCC(c1ccc(cc1)C)C)C | 92139 |
| 233 | IMPHY012586 | (-)-alpha-Cadinol | CC1=CC2C(CC1)[C@@](C)(O)CC[C@@H]2C(C)C | 6431302 |
| 234 | IMPHY012589 | 3-(1,5-Dimethyl-4-hexenyl)-6-methylene-1-cyclohexene | CC(C1CCC(=C)C=C1)CCC=C(C)C | 519764 |
| 235 | IMPHY012665 | Levomenol | CC(=CCC[C@@]([C@H]1CCC(=CC1)C)(O)C)C | 442343 |
| 236 | IMPHY012667 | Caryophyllene oxide | C=C1CC[C@H]2O[C@@]2(CC[C@@H]2[C@@H]1CC2(C)C)C | 1742210 |
| 237 | IMPHY012739 | (Z)-beta-Ocimene | C=C/C(=CCC=C(C)C)/C | 5320250 |
| 238 | IMPHY014241 | 4,7,7-Trimethylbicyclo[3.2.0]hept-3-en-6-one | CC1=CCC2C1C(=O)C2(C)C | 12309890 |
| 239 | IMPHY014806 | Caswell No. 264AB | CC([C@@H]1CC[C@H]([C@]23[C@H]1[C@H]2C(=CC3)C)C)C | 442359 |
| 240 | IMPHY014811 | alpha-Phellandrene | CC1=CCC(C=C1)C(C)C | 7460 |
| 241 | IMPHY014817 | Aromadendrene | CC1CCC2C1C1C(C1(C)C)CCC2=C | 91354 |
| 242 | IMPHY014831 | beta-Caryophyllene | C/C/1=CCCC(=C)[C@@H]2[C@@H](CC1)C(C2)(C)C | 5281515 |
| 243 | IMPHY014835 | (E)-beta-ocimene | C=C/C(=C/CC=C(C)C)/C | 5281553 |
| 244 | IMPHY014847 | Bornyl acetate | CC(=O)OC1CC2C(C1(C)CC2)(C)C | 6448 |
| 245 | IMPHY014852 | Camphene | C=C1C2CCC(C1(C)C)C2 | 6616 |
| 246 | IMPHY014865 | Calamenene | CC([C@@H]1CC[C@@H](c2c1cc(C)cc2)C)C | 6429077 |
| 247 | IMPHY014874 | cis-Sabinene hydrate | C[C@@H]1CC[C@@]2(C1C2)C(C)C | 101629835 |
| 248 | IMPHY014914 | Fenchol | OC1C2(C)CCC(C1(C)C)C2 | 15406 |
| 249 | IMPHY014923 | Geraniol | OC/C=C(/CCC=C(C)C)C | 637566 |
| 250 | IMPHY014988 | Limonene | CC1=CCC(CC1)C(=C)C | 22311 |
| 251 | IMPHY015003 | Menthol | CC1CCC(C(C1)O)C(C)C | 1254 |
| 252 | IMPHY015040 | 3-Pinanone | O=C1CC2CC(C1C)C2(C)C | 11038 |
| 253 | IMPHY015123 | alpha-Copaene | CC([C@@H]1CC[C@]2([C@@H]3[C@H]1C2C(=CC3)C)C)C | 70678558 |
| 254 | IMPHY016012 | Allo-Aromadendrene | C[C@@H]1CC[C@H]2[C@@H]1C1C(C1(C)C)CCC2=C | 42608158 |
| 255 | IMPHY016027 | trans-Sabinene hydrate | CC([C@@]12CC[C@](C2C1)(C)O)C | 12315151 |
| 256 | IMPHY000099 | Myrtenol | OCC1=CCC2CC1C2(C)C | 10582 |
| 257 | IMPHY000399 | beta-Bisabolene | CC(=CCCC(=C)[C@H]1CCC(=CC1)C)C | 10104370 |
| 258 | IMPHY000545 | O-Cymene | CC(c1ccccc1C)C | 10703 |
| 259 | IMPHY000602 | M-Cymene | Cc1cccc(c1)C(C)C | 10812 |
| 260 | IMPHY000915 | Chrysanthenone | CC1=CCC2C(=O)C1C2(C)C | 442463 |
| 261 | IMPHY001218 | Thymoquinone | CC(C1=CC(=O)C(=CC1=O)C)C | 10281 |
| 262 | IMPHY001246 | Carvacrol | CC(c1ccc(c(c1)O)C)C | 10364 |
| 263 | IMPHY001548 | Geranylacetone | C/C(=CCCC(=O)C)/CCC=C(C)C | 1549778 |
| 264 | IMPHY001767 | Rosmadial | O=C[C@H]1C(C)(C)CCC[C@]21C(=O)Oc1c2c(C=O)cc(c1O)C(C)C | 15801061 |
| 265 | IMPHY001915 | Octadecane | CCCCCCCCCCCCCCCCCC | 11635 |
| 266 | IMPHY002072 | Pinocarvone | C=C1C(=O)CC2CC1C2(C)C | 121719 |
| 267 | IMPHY002825 | 2-(4-Methylphenyl)propan-2-ol | Cc1ccc(cc1)C(O)(C)C | 14529 |
| 268 | IMPHY003179 | 3-Octanone | CCCCCC(=O)CC | 246728 |
| 269 | IMPHY003238 | Isopulegone | CC1CCC(C(=O)C1)C(=C)C | 34645 |
| 270 | IMPHY003296 | Piperitenone | CC1=CC(=O)C(=C(C)C)CC1 | 381152 |
| 271 | IMPHY003459 | Pimara-8(14),15-diene | C=C[C@]1(C)CC[C@H]2C(=C1)CC[C@@H]1[C@]2(C)CCCC1(C)C | 440909 |
| 272 | IMPHY003485 | Myrcene | C=CC(=C)CCC=C(C)C | 31253 |
| 273 | IMPHY003525 | Nonanal | CCCCCCCCC=O | 31289 |
| 274 | IMPHY003533 | 2-Methylanisole | COc1ccccc1C | 33637 |
| 275 | IMPHY003536 | Eugenol | C=CCc1ccc(c(c1)OC)O | 3314 |
| 276 | IMPHY003537 | Tetradecanal | CCCCCCCCCCCCCC=O | 31291 |
| 277 | IMPHY003616 | Bicyclogermacrene | C/C/1=CCC/C(=C/[C@H]2[C@@H](CC1)C2(C)C)/C | 13894537 |
| 278 | IMPHY003710 | (-)-Isopulegol | C[C@@H]1CC[C@H]([C@@H](C1)O)C(=C)C | 170833 |
| 279 | IMPHY003977 | (-)-beta-Bourbonene | CC([C@@H]1CC[C@@]2([C@H]1[C@H]1C(=C)CC[C@@H]21)C)C | 62566 |
| 280 | IMPHY003982 | gamma-Terpinene | CC1=CCC(=CC1)C(C)C | 7461 |
| 281 | IMPHY003992 | Hesperidin | COc1ccc(cc1O)[C@@H]1CC(=O)c2c(O1)cc(cc2O)O[C@@H]1O[C@H](CO[C@@H]2O[C@@H](C)[C@@H]([C@H]([C@H]2O)O)O)[C@H]([C@@H]([C@H]1O)O)O | 10621 |
| 282 | IMPHY004003 | Neoisopulegol | C[C@@H]1CC[C@H]([C@H](C1)O)C(=C)C | 6553885 |
| 283 | IMPHY004077 | Verbenone | CC1=CC(=O)C2CC1C2(C)C | 29025 |
| 284 | IMPHY004216 | (1S,2S,7S,8S)-2,6,6,9-tetramethyltricyclo[5.4.0.02,8]undec-9-ene | CC1=CC[C@H]2[C@H]3[C@@H]1[C@@]2(C)CCCC3(C)C | 91753627 |
| 285 | IMPHY004225 | Methyl linoleate | CCCCC/C=CC/C=CCCCCCCCC(=O)OC | 5284421 |
| 286 | IMPHY004326 | Rosmanol | O=C1O[C@H]2[C@@H]3[C@]1(CCCC3(C)C)c1c([C@@H]2O)cc(c(c1O)O)C(C)C | 13966122 |
| 287 | IMPHY004332 | (1R,8R,9S,10S)-3,4,8-trihydroxy-11,11-dimethyl-5-propan-2-yl-16-oxatetracyclo[7.5.2.01,10.02,7]hexadeca-2,4,6-trien-15-one | O=C1O[C@H]2[C@@H]3[C@]1(CCCC3(C)C)c1c([C@H]2O)cc(c(c1O)O)C(C)C | 23243694 |
| 288 | IMPHY004631 | Stearic acid | CCCCCCCCCCCCCCCCCC(=O)O | 5281 |
| 289 | IMPHY004660 | Luteolin | Oc1cc(O)c2c(c1)oc(cc2=O)c1ccc(c(c1)O)O | 5280445 |
| 290 | IMPHY005345 | 1-Octen-3-OL | CCCCCC(C=C)O | 18827 |
| 291 | IMPHY005390 | 1-Octen-3-yl acetate | CCCCCC(OC(=O)C)C=C | 17121 |
| 292 | IMPHY005432 | Diosmin | COc1ccc(cc1O)c1cc(=O)c2c(o1)cc(cc2O)O[C@@H]1O[C@H](CO[C@@H]2O[C@@H](C)[C@@H]([C@H]([C@H]2O)O)O)[C@H]([C@@H]([C@H]1O)O)O | 5281613 |
| 293 | IMPHY005609 | beta-Sinensal | C=CC(=C)CC/C=C(/CC/C=C(/C=O)C)C | 5281535 |
| 294 | IMPHY005618 | Germacrene B | C/C/1=CCC/C(=C/CC(=C(C)C)CC1)/C | 5281519 |
| 295 | IMPHY006145 | p-Cymene | Cc1ccc(cc1)C(C)C | 7463 |
| 296 | IMPHY006325 | Myrtenal | O=CC1=CCC2CC1C2(C)C | 61130 |
| 297 | IMPHY006428 | (R)-Lavandulol | OC[C@@H](C(=C)C)CC=C(C)C | 5464156 |
| 298 | IMPHY006519 | Linalool oxide, pyrane, (Z)- | C=C[C@]1(C)OC(C)(C)CC[C@@H]1O | 6431477 |
| 299 | IMPHY006550 | Thymol | Cc1ccc(c(c1)O)C(C)C | 6989 |
| 300 | IMPHY006653 | Carnosic acid | OC(=O)[C@@]12CCCC([C@@H]2CCc2c1c(O)c(c(c2)C(C)C)O)(C)C | 65126 |
| 301 | IMPHY006696 | Methyleugenol | C=CCc1ccc(c(c1)OC)OC | 7127 |
| 302 | IMPHY006944 | Estragole | COc1ccc(cc1)CC=C | 8815 |
| 303 | IMPHY006950 | Tricyclene | CC12C3C1CC(C2(C)C)C3 | 79035 |
| 304 | IMPHY006965 | alpha,alpha-Dimethyl-4-methylenecyclohexanemethanol | CC(C1CCC(=C)CC1)(O)C | 81722 |
| 305 | IMPHY006970 | Decanal | CCCCCCCCCC=O | 8175 |
| 306 | IMPHY007067 | Linalyl acetate | C=CC(OC(=O)C)(CCC=C(C)C)C | 8294 |
| 307 | IMPHY007204 | Dodecanal | CCCCCCCCCCCC=O | 8194 |
| 308 | IMPHY007276 | Nonan-1-ol | CCCCCCCCCO | 8914 |
| 309 | IMPHY007327 | Palmitic acid | CCCCCCCCCCCCCCCC(=O)O | 985 |
| 310 | IMPHY007366 | alpha-Santalene | CC(=CCCC1(C)C2CC3C1(C)C3C2)C | 94164 |
| 311 | IMPHY007367 | Widdrol | C[C@@]1(O)CC=C2[C@@](CC1)(C)CCCC2(C)C | 94334 |
| 312 | IMPHY007376 | beta-Cubebene | CC([C@@H]1CC[C@H]([C@]23[C@H]1[C@H]2C(=C)CC3)C)C | 93081 |
| 313 | IMPHY007378 | (2S,3R,4R,5S,6S)-2-(hydroxymethyl)-6-(((2S,3R,4R,5S,6R)-3,4,5-trihydroxy-6-(((4aR,8aS)-4-isopropyl-1,7-dimethyl-1,2,3,4,4a,5,8,8a-octahydronaphthalen-1-yl)oxy)tetrahydro-2H-pyran-2-yl)methoxy)tetrahyd | OC[C@@H]1O[C@H](OC[C@@H]2O[C@H](OC3(C)CCC([C@@H]4[C@@H]3CC(=CC4)C)C(C)C)[C@H]([C@@H]([C@H]2O)O)O)[C@H]([C@@H]([C@H]1O)O)O | 91754221 |
| 314 | IMPHY007421 | Citronellyl acetate | CC(CCC=C(C)C)CCOC(=O)C | 9017 |
| 315 | IMPHY007606 | Thymohydroquinone | CC(c1cc(O)c(cc1O)C)C | 95779 |
| 316 | IMPHY007620 | 1-Octanol | CCCCCCCCO | 957 |
| 317 | IMPHY007840 | Spathulenol | C=C1CC[C@@H]2[C@H]([C@H]3[C@H]1CC[C@]3(C)O)C2(C)C | 92231 |
| 318 | IMPHY008024 | Isobutyl isobutyrate | CC(COC(=O)C(C)C)C | 7351 |
| 319 | IMPHY008150 | 1-Methyl-4-(prop-1-en-2-yl)benzene | Cc1ccc(cc1)C(=C)C | 62385 |
| 320 | IMPHY008936 | alpha-Guaiene | CC(=C)[C@@H]1CC[C@@H](C2=C(C1)[C@@H](C)CC2)C | 5317844 |
| 321 | IMPHY009171 | Miltirone | CC(C1=Cc2ccc3c(c2C(=O)C1=O)CCCC3(C)C)C | 160142 |
| 322 | IMPHY009729 | Verbenene | C=C1C=CC2CC1C2(C)C | 6427476 |
| 323 | IMPHY009778 | 1-Isopropyl-4-methylenebicyclo[3.1.0]hex-2-ene | CC(C12C=CC(=C)C2C1)C | 524198 |
| 324 | IMPHY009832 | Lavandulyl acetate | CC(=O)OCC(C(=C)C)CC=C(C)C | 30247 |
| 325 | IMPHY009871 | Isoterpinolene | CC1CCC(=C(C)C)C=C1 | 102443 |
| 326 | IMPHY009874 | Myrtenyl acetate | CC(=O)OCC1=CCC2CC1C2(C)C | 61262 |
| 327 | IMPHY010005 | Humulane-1,6-dien-3-ol | CC1/C=C/C(C)(C)CCC/C(=CC(C1)O)/C | 5353015 |
| 328 | IMPHY010072 | Eucalyptol | CC12CCC(CC1)C(O2)(C)C | 2758 |
| 329 | IMPHY010080 | beta-Elemene | C=C[C@]1(C)CC[C@H](C[C@H]1C(=C)C)C(=C)C | 6918391 |
| 330 | IMPHY010097 | Benzyl benzoate | O=C(c1ccccc1)OCc1ccccc1 | 2345 |
| 331 | IMPHY010972 | Homoplantaginin | OC[C@H]1O[C@@H](Oc2cc3oc(cc(=O)c3c(c2OC)O)c2ccc(cc2)O)[C@@H]([C@H]([C@@H]1O)O)O | 5318083 |
| 332 | IMPHY011004 | p-Mentha-1,3,8-triene | CC1=CC=C(CC1)C(=C)C | 176983 |
| 333 | IMPHY011007 | 1-Decene | CCCCCCCCC=C | 13381 |
| 334 | IMPHY011051 | Abietatriene | CC(c1ccc2c(c1)CC[C@@H]1[C@]2(C)CCCC1(C)C)C | 6432211 |
| 335 | IMPHY011239 | Carnosol | O=C1O[C@H]2C[C@@H]3[C@]1(CCCC3(C)C)c1c2cc(c(c1O)O)C(C)C | 442009 |
| 336 | IMPHY011354 | trans-Sabinene hydrate acetate | CC(=O)O[C@@]1(C)CC[C@@]2(C1C2)C(C)C | 6427504 |
| 337 | IMPHY011392 | 3-Carene | CC1=CCC2C(C1)C2(C)C | 26049 |
| 338 | IMPHY011396 | 4-Carvomenthenol | CC1=CCC(CC1)(O)C(C)C | 11230 |
| 339 | IMPHY011505 | Apigenin 7,4'-dimethyl ether | COc1ccc(cc1)c1cc(=O)c2c(o1)cc(cc2O)OC | 5281601 |
| 340 | IMPHY011519 | alpha-Terpinyl acetate | CC(=O)OC(C1CCC(=CC1)C)(C)C | 111037 |
| 341 | IMPHY011552 | (1R)-2-methyl-5-propan-2-ylbicyclo[3.1.0]hex-2-ene | CC1=CCC2([C@@H]1C2)C(C)C | 6451618 |
| 342 | IMPHY011558 | Apiole | C=CCc1cc(OC)c2c(c1OC)OCO2 | 10659 |
| 343 | IMPHY011562 | 2-Hexenal | CCC/C=C/C=O | 5281168 |
| 344 | IMPHY011564 | Germacra-1(10),5-dien-4-ol | C/C/1=C/CC[C@@](C)(O)/C=C[C@@H](CC1)C(C)C | 101589676 |
| 345 | IMPHY011568 | alpha-Fenchyl acetate | CC(=O)O[C@@H]1[C@]2(C)CC[C@H](C1(C)C)C2 | 7530950 |
| 346 | IMPHY011581 | alpha-Selinene | CC1=CCC[C@]2([C@H]1C[C@@H](CC2)C(=C)C)C | 10856614 |
| 347 | IMPHY011586 | (S,1Z,6Z)-8-Isopropyl-1-methyl-5-methylenecyclodeca-1,6-diene | C/C/1=C/CCC(=C)/C=C[C@@H](CC1)C(C)C | 91723653 |
| 348 | IMPHY011589 | 7-epi-alpha-Eudesmol | CC1=CCC[C@]2([C@H]1C[C@H](CC2)C(O)(C)C)C | 12304196 |
| 349 | IMPHY011590 | d-Borneol | O[C@@H]1C[C@H]2C([C@@]1(C)CC2)(C)C | 61060 |
| 350 | IMPHY011599 | Terpinolene | CC1=CCC(=C(C)C)CC1 | 11463 |
| 351 | IMPHY011643 | alpha-Terpinene | CC1=CC=C(CC1)C(C)C | 7462 |
| 352 | IMPHY011647 | Geranyl acetate | C/C(=CCOC(=O)C)/CCC=C(C)C | 1549026 |
| 353 | IMPHY011648 | Neryl acetate | C/C(=C/COC(=O)C)/CCC=C(C)C | 1549025 |
| 354 | IMPHY011658 | beta-Farnesene | C=CC(=C)CC/C=C(/CCC=C(C)C)C | 5281517 |
| 355 | IMPHY011659 | alpha-Muurolene | CC1=C[C@@H]2[C@H](CC1)C(=CC[C@H]2C(C)C)C | 12306047 |
| 356 | IMPHY011660 | (+)-alpha-Cadinene | CC1=C[C@@H]2[C@@H](CC1)C(=CC[C@H]2C(C)C)C | 12306048 |
| 357 | IMPHY011667 | alpha-Gurjunene | C[C@@H]1CC[C@@H]2[C@H](C3=C(CC[C@H]13)C)C2(C)C | 15560276 |
| 358 | IMPHY011749 | Humulene epoxide II | C/C/1=CCC(C)(C)/C=C/C[C@@]2([C@@H](CC1)O2)C | 10704181 |
| 359 | IMPHY011761 | Humulene | C/C/1=CCC(C)(C)/C=C/C/C(=C/CC1)/C | 5281520 |
| 360 | IMPHY011763 | Anethole | C/C=C/c1ccc(cc1)OC | 637563 |
| 361 | IMPHY011789 | Citral | O=C/C=C(/CCC=C(C)C)C | 638011 |
| 362 | IMPHY011790 | Neral | O=C/C=C(CCC=C(C)C)/C | 643779 |
| 363 | IMPHY011792 | gamma-Muurolene | CC1=C[C@@H]2[C@H](CC1)C(=C)CC[C@H]2C(C)C | 12313020 |
| 364 | IMPHY011793 | (+)-gamma-Cadinene | CC1=C[C@@H]2[C@@H](CC1)C(=C)CC[C@H]2C(C)C | 6432404 |
| 365 | IMPHY011817 | alpha-Farnesene | C=C/C(=C/C/C=C(/CCC=C(C)C)C)/C | 5281516 |
| 366 | IMPHY011880 | Ursolic acid | C[C@@H]1CC[C@]2([C@@H]([C@H]1C)C1=CC[C@H]3[C@@]([C@@]1(CC2)C)(C)CC[C@@H]1[C@]3(C)CC[C@@H](C1(C)C)O)C(=O)O | 64945 |
| 367 | IMPHY011884 | Pulegone | C[C@@H]1CCC(=C(C)C)C(=O)C1 | 442495 |
| 368 | IMPHY011896 | Valencene | CC(=C)[C@@H]1CCC2=CCC[C@H]([C@@]2(C1)C)C | 9855795 |
| 369 | IMPHY011901 | Thujone | O=C1C[C@]2([C@@H]([C@H]1C)C2)C(C)C | 261491 |
| 370 | IMPHY011902 | beta-Thujone | O=C1C[C@]2([C@@H]([C@@H]1C)C2)C(C)C | 91456 |
| 371 | IMPHY011957 | (+)-delta-Cadinene | CC1=C[C@@H]2C(=C(C)CC[C@H]2C(C)C)CC1 | 441005 |
| 372 | IMPHY011965 | (+)-beta-Phellandrene | CC([C@@H]1CCC(=C)C=C1)C | 442484 |
| 373 | IMPHY011999 | Carveol | CC(=C)C1CC=C(C(C1)O)C | 7438 |
| 374 | IMPHY012036 | Camphor | O=C1CC2C(C1(C)CC2)(C)C | 2537 |
| 375 | IMPHY012058 | Linalool | C=CC(CCC=C(C)C)(O)C | 6549 |
| 376 | IMPHY012061 | alpha-Pinene | CC1=CCC2CC1C2(C)C | 6654 |
| 377 | IMPHY012075 | Carvone | CC(=C)C1CC=C(C(=O)C1)C | 7439 |
| 378 | IMPHY012086 | Citronellal | O=CCC(CCC=C(C)C)C | 7794 |
| 379 | IMPHY012104 | Citronellol | OCCC(CCC=C(C)C)C | 8842 |
| 380 | IMPHY012130 | Dihydrocarveol | CC(=C)C1CCC(C(C1)O)C | 12072 |
| 381 | IMPHY012145 | 1-Tetradecene | CCCCCCCCCCCCC=C | 14260 |
| 382 | IMPHY012147 | beta-Pinene | C=C1CCC2CC1C2(C)C | 14896 |
| 383 | IMPHY012152 | alpha-Fenchol | O[C@H]1[C@@]2(C)CC[C@@H](C1(C)C)C2 | 439711 |
| 384 | IMPHY012160 | alpha-Terpineol | CC1=CCC(CC1)C(O)(C)C | 17100 |
| 385 | IMPHY012165 | Sabinene | C=C1CCC2(C1C2)C(C)C | 18818 |
| 386 | IMPHY012168 | (1S,2S,6S,7R,8R)-1,3-dimethyl-8-propan-2-yltricyclo[4.4.0.02,7]dec-3-ene | CC([C@H]1CC[C@]2([C@@H]3[C@@H]1[C@H]2C(=CC3)C)C)C | 101607926 |
| 387 | IMPHY012178 | p-Menthan-3-one | CC1CCC(C(=O)C1)C(C)C | 6986 |
| 388 | IMPHY012198 | Verbenol | CC1=CC(O)C2CC1C2(C)C | 61126 |
| 389 | IMPHY012205 | Sabinene hydrate | CC(C12CCC(C2C1)(C)O)C | 62367 |
| 390 | IMPHY012254 | Isopinocamphone | O=C1C[C@H]2C[C@@H]([C@@H]1C)C2(C)C | 84532 |
| 391 | IMPHY012265 | (1r,3s,5r)-6,6-Dimethyl-2-methylidenebicyclo[3.1.1]heptan-3-ol | C=C1[C@@H](O)C[C@H]2C[C@@H]1C2(C)C | 88302 |
| 392 | IMPHY012279 | alpha-Curcumene | CC(=CCCC(c1ccc(cc1)C)C)C | 92139 |
| 393 | IMPHY012285 | Nepetalactone | CC1CCC2C1C(=O)OC=C2C | 92770 |
| 394 | IMPHY012286 | Totarol | CC(c1c(O)ccc2c1CC[C@@H]1[C@]2(C)CCCC1(C)C)C | 92783 |
| 395 | IMPHY012326 | Nepitrin | OC[C@H]1O[C@@H](Oc2cc3oc(cc(=O)c3c(c2OC)O)c2ccc(c(c2)O)O)[C@@H]([C@H]([C@@H]1O)O)O | 120742 |
| 396 | IMPHY012328 | 1,1,6-Trimethyl-1,2-dihydronaphthalene | Cc1ccc2c(c1)C=CCC2(C)C | 121677 |
| 397 | IMPHY012379 | Cirsimarin | OC[C@H]1O[C@@H](Oc2ccc(cc2)c2cc(=O)c3c(o2)cc(c(c3O)OC)OC)[C@@H]([C@H]([C@@H]1O)O)O | 159460 |
| 398 | IMPHY012586 | (-)-alpha-Cadinol | CC1=CC2C(CC1)[C@@](C)(O)CC[C@@H]2C(C)C | 6431302 |
| 399 | IMPHY012589 | 3-(1,5-Dimethyl-4-hexenyl)-6-methylene-1-cyclohexene | CC(C1CCC(=C)C=C1)CCC=C(C)C | 519764 |
| 400 | IMPHY012598 | Aristolene | CC1CCCC2=CCC3C(C12C)C3(C)C | 530421 |
| 401 | IMPHY012654 | Nerol | OC/C=C(CCC=C(C)C)/C | 643820 |
| 402 | IMPHY012665 | Levomenol | CC(=CCC[C@@]([C@H]1CCC(=CC1)C)(O)C)C | 442343 |
| 403 | IMPHY012667 | Caryophyllene oxide | C=C1CC[C@H]2O[C@@]2(CC[C@@H]2[C@@H]1CC2(C)C)C | 1742210 |
| 404 | IMPHY012712 | Phytol | OC/C=C(/CCC[C@@H](CCC[C@@H](CCCC(C)C)C)C)C | 5280435 |
| 405 | IMPHY012719 | Cosmosiin | OC[C@H]1O[C@@H](Oc2cc(O)c3c(c2)oc(cc3=O)c2ccc(cc2)O)[C@@H]([C@H]([C@@H]1O)O)O | 5280704 |
| 406 | IMPHY012737 | (1S,4E,9S)-4,11,11-trimethyl-8-methylidenebicyclo[7.2.0]undec-4-ene | C/C/1=CCCC(=C)[C@@H]2[C@H](CC1)C(C2)(C)C | 6429301 |
| 407 | IMPHY012739 | (Z)-beta-Ocimene | C=C/C(=CCC=C(C)C)/C | 5320250 |
| 408 | IMPHY012910 | trans-Calamenene | CC([C@H]1CC[C@@H](c2c1cc(C)cc2)C)C | 6429022 |
| 409 | IMPHY012914 | trans-Sabinyl acetate | CC(=O)O[C@H]1C[C@@]2(C(C1=C)C2)C(C)C | 6430313 |
| 410 | IMPHY012921 | gamma-Elemene | C=C[C@]1(C)CCC(=C(C)C)C[C@H]1C(=C)C | 6432312 |
| 411 | IMPHY014811 | alpha-Phellandrene | CC1=CCC(C=C1)C(C)C | 7460 |
| 412 | IMPHY014817 | Aromadendrene | CC1CCC2C1C1C(C1(C)C)CCC2=C | 91354 |
| 413 | IMPHY014831 | beta-Caryophyllene | C/C/1=CCCC(=C)[C@@H]2[C@@H](CC1)C(C2)(C)C | 5281515 |
| 414 | IMPHY014835 | (E)-beta-ocimene | C=C/C(=C/CC=C(C)C)/C | 5281553 |
| 415 | IMPHY014847 | Bornyl acetate | CC(=O)OC1CC2C(C1(C)CC2)(C)C | 6448 |
| 416 | IMPHY014852 | Camphene | C=C1C2CCC(C1(C)C)C2 | 6616 |
| 417 | IMPHY014865 | Calamenene | CC([C@@H]1CC[C@@H](c2c1cc(C)cc2)C)C | 6429077 |
| 418 | IMPHY014873 | 2-Cyclohexen-1-ol, 3-methyl-6-(1-methylethyl)-, (1R,6S)-rel- | CC1=C[C@@H]([C@@H](CC1)C(C)C)O | 85567 |
| 419 | IMPHY014874 | cis-Sabinene hydrate | C[C@@H]1CC[C@@]2(C1C2)C(C)C | 101629835 |
| 420 | IMPHY014877 | (S)-cis-Verbenol | CC1=C[C@H](O)[C@H]2C[C@@H]1C2(C)C | 87839 |
| 421 | IMPHY012969 | salicylic acid 2-beta-D-glucoside | OC[C@H]1O[C@@H](Oc2ccccc2C(=O)[O-])[C@@H]([C@H]([C@@H]1O)O)O | 7099939 |
| 422 | IMPHY012997 | Luteolin 3'-o-glucuronide | Oc1cc(O)c2c(c1)oc(cc2=O)c1ccc(c(c1)O[C@@H]1O[C@H](C(=O)O)[C@H]([C@@H]([C@H]1O)O)O)O | 10253785 |
| 423 | IMPHY013080 | alpha-Calacorene | CC([C@@H]1CC=C(c2c1cc(C)cc2)C)C | 12302243 |
| 424 | IMPHY013133 | (Z)-p-Menth-2-en-1-ol | CC([C@@H]1CC[C@](C=C1)(C)O)C | 13918681 |
| 425 | IMPHY013805 | 2-Phenylethyl hexanoate | CCCCCC(=O)OCCc1ccccc1 | 61384 |
| 426 | IMPHY013836 | Fenchone | O=C1C2(C)CCC(C1(C)C)C2 | 14525 |
| 427 | IMPHY014241 | 4,7,7-Trimethylbicyclo[3.2.0]hept-3-en-6-one | CC1=CCC2C1C(=O)C2(C)C | 12309890 |
| 428 | IMPHY014708 | beta-Selinene | C=C1CCC[C@]2([C@H]1C[C@@H](CC2)C(=C)C)C | 442393 |
| 429 | IMPHY014801 | Zizanene | CC1=C[C@@H]2[C@H](CC1)C(=CC[C@@H]2C(C)C)C | 12306046 |
| 430 | IMPHY014806 | Caswell No. 264AB | CC([C@@H]1CC[C@H]([C@]23[C@H]1[C@H]2C(=CC3)C)C)C | 442359 |
| 431 | IMPHY014885 | 1-Isopropyl-4,7-dimethyl-1,3,4,5,6,8a-hexahydro-4a(2H)-naphthalenol | CC1=CC2C(CC1)(O)C(C)CCC2C(C)C | 519857 |
| 432 | IMPHY014923 | Geraniol | OC/C=C(/CCC=C(C)C)C | 637566 |
| 433 | IMPHY014942 | Bicyclo[2.2.1]heptan-2-ol, 1,7,7-trimethyl-, formate, (1R,2R,4R)-rel- | O=CO[C@@H]1C[C@@H]2C([C@@]1(C)CC2)(C)C | 23623868 |
| 434 | IMPHY014986 | Ledol | C[C@@H]1CC[C@H]2[C@@H]1[C@H]1[C@H](C1(C)C)CC[C@@]2(C)O | 92812 |
| 435 | IMPHY014988 | Limonene | CC1=CCC(CC1)C(=C)C | 22311 |
| 436 | IMPHY014990 | Linoleic acid | CCCCC/C=CC/C=CCCCCCCCC(=O)O | 5280450 |
| 437 | IMPHY015004 | Menthone | C[C@@H]1CC[C@H](C(=O)C1)C(C)C | 26447 |
| 438 | IMPHY015016 | alpha-Muurolol | CC1=C[C@@H]2[C@H](CC1)[C@](C)(O)CC[C@@H]2C(C)C | 91753440 |
| 439 | IMPHY015022 | Nerolidol | C=CC(CC/C=C(/CCC=C(C)C)C)(O)C | 5284507 |
| 440 | IMPHY015040 | 3-Pinanone | O=C1CC2CC(C1C)C2(C)C | 11038 |
| 441 | IMPHY015042 | Piperitone | CC1=CC(=O)C(CC1)C(C)C | 6987 |
| 442 | IMPHY015094 | (+)-trans-Limonene oxide | CC(=C)[C@@H]1CC[C@]2([C@@H](C1)O2)C | 449290 |
| 443 | IMPHY015095 | 2-Cyclohexen-1-ol, 1-methyl-4-(1-methylethyl)-, trans- | CC([C@@H]1CC[C@@](C=C1)(C)O)C | 122484 |
| 444 | IMPHY015123 | alpha-Copaene | CC([C@@H]1CC[C@]2([C@@H]3[C@H]1C2C(=CC3)C)C)C | 70678558 |
| 445 | IMPHY015330 | 3-Eicosyne | CCCCCCCCCCCCCCCCC#CCC | 549159 |
| 446 | IMPHY016012 | Allo-Aromadendrene | C[C@@H]1CC[C@H]2[C@@H]1C1C(C1(C)C)CCC2=C | 42608158 |
| 447 | IMPHY016014 | Isobornyl acetate | CC(=O)O[C@H]1C[C@@H]2C([C@]1(C)CC2)(C)C | 247573 |
| 448 | IMPHY016027 | trans-Sabinene hydrate | CC([C@@]12CC[C@](C2C1)(C)O)C | 12315151 |
| 449 | IMPHY016053 | Viridiflorol | C[C@@H]1CC[C@H]2[C@@H]1[C@H]1[C@H](C1(C)C)CC[C@]2(C)O | 11996452 |
| 450 | IMPHY016956 | 2-Phenylethyl pivalate | O=C(C(C)(C)C)OCCc1ccccc1 | 105516 |
| 451 | IMPHY017035 | gamma-Thujaplicin | CC(c1ccc(=O)c(cc1)O)C | 12649 |
| 452 | IMPHY017239 | Phenethyl 2-furoate | O=C(c1ccco1)OCCc1ccccc1 | 251531 |
| 453 | IMPHY017423 | (3E)-2,6-dimethylhepta-3,5-dien-2-ol | CC(=C/C=C/C(O)(C)C)C | 5369969 |
| 454 | IMPHY017663 | alpha-Santalyl acetate | CC(=O)OC/C(=CCC[C@]1(C)C2C[C@@H]3C1(C)[C@@H]3C2)/C | 6445771 |
| 455 | IMPHY017689 | 2'-Hydroxy-5'-methoxyacetophenone | COc1ccc(c(c1)C(=O)C)O | 69714 |
| 456 | IMPHY017957 | Cedranediol | C[C@@H]1CC[C@@]23C[C@@H]1C(C)(C)[C@@H]2C(C[C@H]3C)(O)O |  |
| 457 | IMPHY001086 | Royleanone | O=C1C(=O)C(=C(C2=C1[C@@]1(C)CCCC([C@@H]1CC2)(C)C)O)C(C)C | 442084 |
| 458 | IMPHY002870 | Horminone | O[C@@H]1C[C@H]2C(C)(C)CCC[C@@]2(C2=C1C(=C(C(C)C)C(=O)C2=O)O)C | 2751795 |
| 459 | IMPHY007798 | Cryptotanshinone | C[C@H]1COC2=C1C(=O)C(=O)c1c2ccc2c1CCCC2(C)C | 160254 |
| 460 | IMPHY010854 | Taxodione | CC(C1=CC2=CC(=O)[C@@H]3[C@](C2=C(C1=O)O)(C)CCCC3(C)C)C | 73588 |
| 461 | IMPHY014776 | 7alpha-Acetoxyroyleanone | CC(=O)O[C@@H]1C[C@H]2C(C)(C)CCC[C@@]2(C2=C1C(=C(C(C)C)C(=O)C2=O)O)C | 2751796 |
| 462 | IMPHY017250 | 6,7-Dehydroroyleanone | O=C1C(=O)C(=C(C2=C1[C@@]1(C)CCCC([C@@H]1C=C2)(C)C)O)C(C)C | 2751794 |
| 463 | IMPHY003485 | Myrcene | C=CC(=C)CCC=C(C)C | 31253 |
| 464 | IMPHY006145 | p-Cymene | Cc1ccc(cc1)C(C)C | 7463 |
| 465 | IMPHY010072 | Eucalyptol | CC12CCC(CC1)C(O2)(C)C | 2758 |
| 466 | IMPHY011396 | 4-Carvomenthenol | CC1=CCC(CC1)(O)C(C)C | 11230 |
| 467 | IMPHY011590 | d-Borneol | O[C@@H]1C[C@H]2C([C@@]1(C)CC2)(C)C | 61060 |
| 468 | IMPHY012036 | Camphor | O=C1CC2C(C1(C)CC2)(C)C | 2537 |
| 469 | IMPHY012061 | alpha-Pinene | CC1=CCC2CC1C2(C)C | 6654 |
| 470 | IMPHY012147 | beta-Pinene | C=C1CCC2CC1C2(C)C | 14896 |
| 471 | IMPHY012160 | alpha-Terpineol | CC1=CCC(CC1)C(O)(C)C | 17100 |
| 472 | IMPHY012165 | Sabinene | C=C1CCC2(C1C2)C(C)C | 18818 |
| 473 | IMPHY012175 | D-Limonene | CC1=CC[C@@H](CC1)C(=C)C | 440917 |
| 474 | IMPHY014831 | beta-Caryophyllene | C/C/1=CCCC(=C)[C@@H]2[C@@H](CC1)C(C2)(C)C | 5281515 |
| 475 | IMPHY014847 | Bornyl acetate | CC(=O)OC1CC2C(C1(C)CC2)(C)C | 6448 |
| 476 | IMPHY014852 | Camphene | C=C1C2CCC(C1(C)C)C2 | 6616 |
| 477 | IMPHY014923 | Geraniol | OC/C=C(/CCC=C(C)C)C | 637566 |
| 478 | IMPHY001246 | Carvacrol | CC(c1ccc(c(c1)O)C)C | 10364 |
| 479 | IMPHY003179 | 3-Octanone | CCCCCC(=O)CC | 246728 |
| 480 | IMPHY003485 | Myrcene | C=CC(=C)CCC=C(C)C | 31253 |
| 481 | IMPHY003531 | Rosmaricine | O=C1OC2C3C1(CCCC3(C)C)c1c(C2N)cc(c(c1O)O)C(C)C | 330877 |
| 482 | IMPHY003536 | Eugenol | C=CCc1ccc(c(c1)OC)O | 3314 |
| 483 | IMPHY003710 | (-)-Isopulegol | C[C@@H]1CC[C@H]([C@@H](C1)O)C(=C)C | 170833 |
| 484 | IMPHY003982 | gamma-Terpinene | CC1=CCC(=CC1)C(C)C | 7461 |
| 485 | IMPHY004003 | Neoisopulegol | C[C@@H]1CC[C@H]([C@H](C1)O)C(=C)C | 6553885 |
| 486 | IMPHY004077 | Verbenone | CC1=CC(=O)C2CC1C2(C)C | 29025 |
| 487 | IMPHY004194 | (1S,2R,5S)-2-isopropyl-5-methylcyclohexyl acetate | C[C@H]1CC[C@@H]([C@H](C1)OC(=O)C)C(C)C | 62335 |
| 488 | IMPHY004597 | Rosmarinic acid | O=C(O[C@@H](C(=O)O)Cc1ccc(c(c1)O)O)/C=C/c1ccc(c(c1)O)O | 5281792 |
| 489 | IMPHY004661 | Apigenin | Oc1ccc(cc1)c1cc(=O)c2c(o1)cc(cc2O)O | 5280443 |
| 490 | IMPHY005431 | Diosmetin | COc1ccc(cc1O)c1cc(=O)c2c(o1)cc(cc2O)O | 5281612 |
| 491 | IMPHY005432 | Diosmin | COc1ccc(cc1O)c1cc(=O)c2c(o1)cc(cc2O)O[C@@H]1O[C@H](CO[C@@H]2O[C@@H](C)[C@@H]([C@H]([C@H]2O)O)O)[C@H]([C@@H]([C@H]1O)O)O | 5281613 |
| 492 | IMPHY005940 | Thunbergol | C/C/1=C/CC/C(=C/CCC(/C=C/C(CC1)C(C)C)(C)O)/C | 5363523 |
| 493 | IMPHY006145 | p-Cymene | Cc1ccc(cc1)C(C)C | 7463 |
| 494 | IMPHY006550 | Thymol | Cc1ccc(c(c1)O)C(C)C | 6989 |
| 495 | IMPHY006944 | Estragole | COc1ccc(cc1)CC=C | 8815 |
| 496 | IMPHY007067 | Linalyl acetate | C=CC(OC(=O)C)(CCC=C(C)C)C | 8294 |
| 497 | IMPHY007219 | Isobutyl acetate | CC(COC(=O)C)C | 8038 |
| 498 | IMPHY007317 | 4-Allylphenol | C=CCc1ccc(cc1)O | 68148 |
| 499 | IMPHY009832 | Lavandulyl acetate | CC(=O)OCC(C(=C)C)CC=C(C)C | 30247 |
| 500 | IMPHY010072 | Eucalyptol | CC12CCC(CC1)C(O2)(C)C | 2758 |
| 501 | IMPHY011396 | 4-Carvomenthenol | CC1=CCC(CC1)(O)C(C)C | 11230 |
| 502 | IMPHY011519 | alpha-Terpinyl acetate | CC(=O)OC(C1CCC(=CC1)C)(C)C | 111037 |
| 503 | IMPHY011590 | d-Borneol | O[C@@H]1C[C@H]2C([C@@]1(C)CC2)(C)C | 61060 |
| 504 | IMPHY011599 | Terpinolene | CC1=CCC(=C(C)C)CC1 | 11463 |
| 505 | IMPHY011643 | alpha-Terpinene | CC1=CC=C(CC1)C(C)C | 7462 |
| 506 | IMPHY011647 | Geranyl acetate | C/C(=CCOC(=O)C)/CCC=C(C)C | 1549026 |
| 507 | IMPHY011648 | Neryl acetate | C/C(=C/COC(=O)C)/CCC=C(C)C | 1549025 |
| 508 | IMPHY011761 | Humulene | C/C/1=CCC(C)(C)/C=C/C/C(=C/CC1)/C | 5281520 |
| 509 | IMPHY011826 | Oleanolic acid | O[C@H]1CC[C@]2([C@H](C1(C)C)CC[C@@]1([C@@H]2CC=C2[C@@]1(C)CC[C@@]1([C@H]2CC(C)(C)CC1)C(=O)O)C)C | 10494 |
| 510 | IMPHY011880 | Ursolic acid | C[C@@H]1CC[C@]2([C@@H]([C@H]1C)C1=CC[C@H]3[C@@]([C@@]1(CC2)C)(C)CC[C@@H]1[C@]3(C)CC[C@@H](C1(C)C)O)C(=O)O | 64945 |
| 511 | IMPHY011884 | Pulegone | C[C@@H]1CCC(=C(C)C)C(=O)C1 | 442495 |
| 512 | IMPHY011901 | Thujone | O=C1C[C@]2([C@@H]([C@H]1C)C2)C(C)C | 261491 |
| 513 | IMPHY011902 | beta-Thujone | O=C1C[C@]2([C@@H]([C@@H]1C)C2)C(C)C | 91456 |
| 514 | IMPHY012036 | Camphor | O=C1CC2C(C1(C)CC2)(C)C | 2537 |
| 515 | IMPHY012058 | Linalool | C=CC(CCC=C(C)C)(O)C | 6549 |
| 516 | IMPHY012061 | alpha-Pinene | CC1=CCC2CC1C2(C)C | 6654 |
| 517 | IMPHY012147 | beta-Pinene | C=C1CCC2CC1C2(C)C | 14896 |
| 518 | IMPHY012160 | alpha-Terpineol | CC1=CCC(CC1)C(O)(C)C | 17100 |
| 519 | IMPHY012165 | Sabinene | C=C1CCC2(C1C2)C(C)C | 18818 |
| 520 | IMPHY012178 | p-Menthan-3-one | CC1CCC(C(=O)C1)C(C)C | 6986 |
| 521 | IMPHY012198 | Verbenol | CC1=CC(O)C2CC1C2(C)C | 61126 |
| 522 | IMPHY012739 | (Z)-beta-Ocimene | C=C/C(=CCC=C(C)C)/C | 5320250 |
| 523 | IMPHY013835 | (+)-Fenchone | O=C1[C@@]2(C)CC[C@@H](C1(C)C)C2 | 1201521 |
| 524 | IMPHY014831 | beta-Caryophyllene | C/C/1=CCCC(=C)[C@@H]2[C@@H](CC1)C(C2)(C)C | 5281515 |
| 525 | IMPHY014835 | (E)-beta-ocimene | C=C/C(=C/CC=C(C)C)/C | 5281553 |
| 526 | IMPHY014847 | Bornyl acetate | CC(=O)OC1CC2C(C1(C)CC2)(C)C | 6448 |
| 527 | IMPHY014852 | Camphene | C=C1C2CCC(C1(C)C)C2 | 6616 |
| 528 | IMPHY014923 | Geraniol | OC/C=C(/CCC=C(C)C)C | 637566 |
| 529 | IMPHY014988 | Limonene | CC1=CCC(CC1)C(=C)C | 22311 |
| 530 | IMPHY015003 | Menthol | CC1CCC(C(C1)O)C(C)C | 1254 |
| 531 | IMPHY015004 | Menthone | C[C@@H]1CC[C@H](C(=O)C1)C(C)C | 26447 |
| 532 | IMPHY015042 | Piperitone | CC1=CC(=O)C(CC1)C(C)C | 6987 |
| 533 | IMPHY015047 | Rutin | Oc1cc(O)c2c(c1)oc(c(c2=O)O[C@@H]1O[C@H](CO[C@@H]2O[C@@H](C)[C@@H]([C@H]([C@H]2O)O)O)[C@H]([C@@H]([C@H]1O)O)O)c1ccc(c(c1)O)O | 5280805 |
| 534 | IMPHY015123 | alpha-Copaene | CC([C@@H]1CC[C@]2([C@@H]3[C@H]1C2C(=CC3)C)C)C | 70678558 |
| 535 | IMPHY016027 | trans-Sabinene hydrate | CC([C@@]12CC[C@](C2C1)(C)O)C | 12315151 |
| 536 | IMPHY003179 | 3-Octanone | CCCCCC(=O)CC | 246728 |
| 537 | IMPHY003485 | Myrcene | C=CC(=C)CCC=C(C)C | 31253 |
| 538 | IMPHY006145 | p-Cymene | Cc1ccc(cc1)C(C)C | 7463 |
| 539 | IMPHY010072 | Eucalyptol | CC12CCC(CC1)C(O2)(C)C | 2758 |
| 540 | IMPHY011599 | Terpinolene | CC1=CCC(=C(C)C)CC1 | 11463 |
| 541 | IMPHY011761 | Humulene | C/C/1=CCC(C)(C)/C=C/C/C(=C/CC1)/C | 5281520 |
| 542 | IMPHY012036 | Camphor | O=C1CC2C(C1(C)CC2)(C)C | 2537 |
| 543 | IMPHY012058 | Linalool | C=CC(CCC=C(C)C)(O)C | 6549 |
| 544 | IMPHY012061 | alpha-Pinene | CC1=CCC2CC1C2(C)C | 6654 |
| 545 | IMPHY012279 | alpha-Curcumene | CC(=CCCC(c1ccc(cc1)C)C)C | 92139 |
| 546 | IMPHY014835 | (E)-beta-ocimene | C=C/C(=C/CC=C(C)C)/C | 5281553 |
| 547 | IMPHY014847 | Bornyl acetate | CC(=O)OC1CC2C(C1(C)CC2)(C)C | 6448 |
| 548 | IMPHY014874 | cis-Sabinene hydrate | C[C@@H]1CC[C@@]2(C1C2)C(C)C | 101629835 |
| 549 | IMPHY003485 | Myrcene | C=CC(=C)CCC=C(C)C | 31253 |
| 550 | IMPHY004077 | Verbenone | CC1=CC(=O)C2CC1C2(C)C | 29025 |
| 551 | IMPHY010072 | Eucalyptol | CC12CCC(CC1)C(O2)(C)C | 2758 |
| 552 | IMPHY011396 | 4-Carvomenthenol | CC1=CCC(CC1)(O)C(C)C | 11230 |
| 553 | IMPHY011590 | d-Borneol | O[C@@H]1C[C@H]2C([C@@]1(C)CC2)(C)C | 61060 |
| 554 | IMPHY012036 | Camphor | O=C1CC2C(C1(C)CC2)(C)C | 2537 |
| 555 | IMPHY012058 | Linalool | C=CC(CCC=C(C)C)(O)C | 6549 |
| 556 | IMPHY012061 | alpha-Pinene | CC1=CCC2CC1C2(C)C | 6654 |
| 557 | IMPHY012147 | beta-Pinene | C=C1CCC2CC1C2(C)C | 14896 |
| 558 | IMPHY012160 | alpha-Terpineol | CC1=CCC(CC1)C(O)(C)C | 17100 |
| 559 | IMPHY014847 | Bornyl acetate | CC(=O)OC1CC2C(C1(C)CC2)(C)C | 6448 |
| 560 | IMPHY014852 | Camphene | C=C1C2CCC(C1(C)C)C2 | 6616 |
| 561 | IMPHY000060 | Myristic acid | CCCCCCCCCCCCCC(=O)O | 11005 |
| 562 | IMPHY000070 | Cadalene | Cc1ccc2c(c1)c(ccc2C)C(C)C | 10225 |
| 563 | IMPHY000099 | Myrtenol | OCC1=CCC2CC1C2(C)C | 10582 |
| 564 | IMPHY000399 | beta-Bisabolene | CC(=CCCC(=C)[C@H]1CCC(=CC1)C)C | 10104370 |
| 565 | IMPHY000491 | Pinene | CC1CCC2CC1C2(C)C |  |
| 566 | IMPHY000545 | O-Cymene | CC(c1ccccc1C)C | 10703 |
| 567 | IMPHY000757 | rosmaquinone B | CO[C@H]1C2=C(C(=O)C(=O)C(=C2)C(C)C)[C@@]23[C@@H]([C@@H]1OC2=O)C(C)(C)CCC3 | 46883407 |
| 568 | IMPHY000915 | Chrysanthenone | CC1=CCC2C(=O)C1C2(C)C | 442463 |
| 569 | IMPHY001071 | [(2R,3S,4R,5R,6S)-6-[[(2S,3S,4S)-3,4-dihydroxy-4-(hydroxymethyl)oxolan-2-yl]oxymethyl]-3,4,5-trihydroxyoxan-2-yl] 3,4-dihydroxybenzoate | OC[C@]1(O)CO[C@@H]([C@H]1O)OC[C@@H]1O[C@H](OC(=O)c2ccc(c(c2)O)O)[C@H]([C@@H]([C@H]1O)O)O | 44558991 |
| 570 | IMPHY001072 | [(2R,3S,4R,5R,6S)-6-[[(2S,3S,4S)-3,4-dihydroxy-4-(hydroxymethyl)oxolan-2-yl]oxymethyl]-3,4,5-trihydroxyoxan-2-yl] (E)-3-(3,4-dihydroxyphenyl)prop-2-enoate | OC[C@]1(O)CO[C@@H]([C@H]1O)OC[C@@H]1O[C@H](OC(=O)/C=C/c2ccc(c(c2)O)O)[C@H]([C@@H]([C@H]1O)O)O | 44558989 |
| 571 | IMPHY001246 | Carvacrol | CC(c1ccc(c(c1)O)C)C | 10364 |
| 572 | IMPHY001315 | Flavanone | O=C1CC(Oc2c1cccc2)c1ccccc1 | 10251 |
| 573 | IMPHY001516 | Decane | CCCCCCCCCC | 15600 |
| 574 | IMPHY001555 | Jasmone | CC/C=CCC1=C(C)CCC1=O | 1549018 |
| 575 | IMPHY001658 | Thymol methyl ether | COc1cc(C)ccc1C(C)C | 14104 |
| 576 | IMPHY001767 | Rosmadial | O=C[C@H]1C(C)(C)CCC[C@]21C(=O)Oc1c2c(C=O)cc(c1O)C(C)C | 15801061 |
| 577 | IMPHY001816 | gamma-Terpineol | CC(=C1CCC(CC1)(C)O)C | 11467 |
| 578 | IMPHY001817 | p-Menth-3-en-1-ol | CC(C1=CCC(CC1)(C)O)C | 11468 |
| 579 | IMPHY001931 | Vanillin | COc1cc(C=O)ccc1O | 1183 |
| 580 | IMPHY002072 | Pinocarvone | C=C1C(=O)CC2CC1C2(C)C | 121719 |
| 581 | IMPHY002533 | (-)-beta-Curcumene | CC(=CCC[C@H](C1=CCC(=CC1)C)C)C | 14014430 |
| 582 | IMPHY002825 | 2-(4-Methylphenyl)propan-2-ol | Cc1ccc(cc1)C(O)(C)C | 14529 |
| 583 | IMPHY003179 | 3-Octanone | CCCCCC(=O)CC | 246728 |
| 584 | IMPHY003296 | Piperitenone | CC1=CC(=O)C(=C(C)C)CC1 | 381152 |
| 585 | IMPHY003401 | Nopol | OCCC1=CCC2CC1C2(C)C | 31408 |
| 586 | IMPHY003485 | Myrcene | C=CC(=C)CCC=C(C)C | 31253 |
| 587 | IMPHY003512 | Ethyl hexanoate | CCCCCC(=O)OCC | 31265 |
| 588 | IMPHY003525 | Nonanal | CCCCCCCCC=O | 31289 |
| 589 | IMPHY003536 | Eugenol | C=CCc1ccc(c(c1)OC)O | 3314 |
| 590 | IMPHY003545 | 4-Isopropylbenzaldehyde | O=Cc1ccc(cc1)C(C)C | 326 |
| 591 | IMPHY003552 | 2,3-Dimethylstyrene | C=Cc1cccc(c1C)C | 33936 |
| 592 | IMPHY003559 | beta-Amyrone | O=C1CC[C@]2([C@H](C1(C)C)CC[C@@]1([C@@H]2CC=C2[C@@]1(C)CC[C@@]1([C@H]2CC(C)(C)CC1)C)C)C | 12306160 |
| 593 | IMPHY003616 | Bicyclogermacrene | C/C/1=CCC/C(=C/[C@H]2[C@@H](CC1)C2(C)C)/C | 13894537 |
| 594 | IMPHY003691 | Methyl jasmonate | CC/C=CC[C@@H]1[C@H](CCC1=O)CC(=O)OC | 5281929 |
| 595 | IMPHY003710 | (-)-Isopulegol | C[C@@H]1CC[C@H]([C@@H](C1)O)C(=C)C | 170833 |
| 596 | IMPHY003977 | (-)-beta-Bourbonene | CC([C@@H]1CC[C@@]2([C@H]1[C@H]1C(=C)CC[C@@H]21)C)C | 62566 |
| 597 | IMPHY003982 | gamma-Terpinene | CC1=CCC(=CC1)C(C)C | 7461 |
| 598 | IMPHY003992 | Hesperidin | COc1ccc(cc1O)[C@@H]1CC(=O)c2c(O1)cc(cc2O)O[C@@H]1O[C@H](CO[C@@H]2O[C@@H](C)[C@@H]([C@H]([C@H]2O)O)O)[C@H]([C@@H]([C@H]1O)O)O | 10621 |
| 599 | IMPHY004049 | alpha-Santalol | OC/C(=CCC[C@]1(C)C2C[C@@H]3C1(C)[C@@H]3C2)/C | 11085337 |
| 600 | IMPHY004077 | Verbenone | CC1=CC(=O)C2CC1C2(C)C | 29025 |
| 601 | IMPHY004151 | Geranyl formate | O=COC/C=C(/CCC=C(C)C)C | 5282109 |
| 602 | IMPHY004194 | (1S,2R,5S)-2-isopropyl-5-methylcyclohexyl acetate | C[C@H]1CC[C@@H]([C@H](C1)OC(=O)C)C(C)C | 62335 |
| 603 | IMPHY004271 | Betulin | OC[C@@]12CC[C@H]([C@@H]2[C@@H]2[C@](CC1)(C)[C@]1(C)CC[C@@H]3[C@]([C@H]1CC2)(C)CC[C@@H](C3(C)C)O)C(=C)C | 72326 |
| 604 | IMPHY004281 | Guaiol | C[C@H]1CC[C@H](CC2=C1CC[C@@H]2C)C(O)(C)C | 227829 |
| 605 | IMPHY004286 | Longifolene | C=C1C2CCC3C1(C)CCCC(C23)(C)C | 289151 |
| 606 | IMPHY004326 | Rosmanol | O=C1O[C@H]2[C@@H]3[C@]1(CCCC3(C)C)c1c([C@@H]2O)cc(c(c1O)O)C(C)C | 13966122 |
| 607 | IMPHY004332 | (1R,8R,9S,10S)-3,4,8-trihydroxy-11,11-dimethyl-5-propan-2-yl-16-oxatetracyclo[7.5.2.01,10.02,7]hexadeca-2,4,6-trien-15-one | O=C1O[C@H]2[C@@H]3[C@]1(CCCC3(C)C)c1c([C@H]2O)cc(c(c1O)O)C(C)C | 23243694 |
| 608 | IMPHY004388 | Kaempferol | Oc1ccc(cc1)c1oc2cc(O)cc(c2c(=O)c1O)O | 5280863 |
| 609 | IMPHY004438 | Geranyl butyrate | CCCC(=O)OC/C=C(/CCC=C(C)C)C | 5355856 |
| 610 | IMPHY004555 | 1,3,3-Trimethyl-2-oxabicyclo[2.2.2]oct-5-ene | CC12CCC(C=C1)C(O2)(C)C | 523035 |
| 611 | IMPHY004597 | Rosmarinic acid | O=C(O[C@@H](C(=O)O)Cc1ccc(c(c1)O)O)/C=C/c1ccc(c(c1)O)O | 5281792 |
| 612 | IMPHY004619 | Quercetin | Oc1cc(O)c2c(c1)oc(c(c2=O)O)c1ccc(c(c1)O)O | 5280343 |
| 613 | IMPHY004660 | Luteolin | Oc1cc(O)c2c(c1)oc(cc2=O)c1ccc(c(c1)O)O | 5280445 |
| 614 | IMPHY004889 | Cinnamyl alcohol | OC/C=C/c1ccccc1 | 5315892 |
| 615 | IMPHY005127 | Hexyl butyrate | CCCCCCOC(=O)CCC | 17525 |
| 616 | IMPHY005196 | 7-O-Methylrosmanol | CO[C@@H]1[C@H]2OC(=O)[C@]3([C@@H]2C(C)(C)CCC3)c2c1cc(C(C)C)c(c2O)O | 23243692 |
| 617 | IMPHY005206 | Rosmaquinone | O[C@H]1C2=C(C(=O)C(=O)C(=C2)C(C)C)[C@@]23[C@@H]([C@@H]1OC2=O)C(C)(C)CCC3 | 23243691 |
| 618 | IMPHY005327 | Cirsimaritin | COc1cc2oc(cc(=O)c2c(c1OC)O)c1ccc(cc1)O | 188323 |
| 619 | IMPHY005344 | Pisiferic acid | OC(=O)[C@@]12CCCC([C@@H]2CCc2c1cc(O)c(c2)C(C)C)(C)C | 162209 |
| 620 | IMPHY005345 | 1-Octen-3-OL | CCCCCC(C=C)O | 18827 |
| 621 | IMPHY005432 | Diosmin | COc1ccc(cc1O)c1cc(=O)c2c(o1)cc(cc2O)O[C@@H]1O[C@H](CO[C@@H]2O[C@@H](C)[C@@H]([C@H]([C@H]2O)O)O)[C@H]([C@@H]([C@H]1O)O)O | 5281613 |
| 622 | IMPHY005434 | Galangin | Oc1cc(O)c2c(c1)oc(c(c2=O)O)c1ccccc1 | 5281616 |
| 623 | IMPHY005435 | Genkwanin | COc1cc(O)c2c(c1)oc(cc2=O)c1ccc(cc1)O | 5281617 |
| 624 | IMPHY005442 | Hispidulin | COc1c(O)cc2c(c1O)c(=O)cc(o2)c1ccc(cc1)O | 5281628 |
| 625 | IMPHY005461 | Isoscutellarein | Oc1ccc(cc1)c1cc(=O)c2c(o1)c(O)c(cc2O)O | 5281665 |
| 626 | IMPHY005501 | Allantoin | NC(=O)NC1NC(=O)NC1=O | 204 |
| 627 | IMPHY006020 | Kaempferitrin | Oc1ccc(cc1)c1oc2cc(O[C@@H]3O[C@@H](C)[C@@H]([C@H]([C@H]3O)O)O)cc(c2c(=O)c1O[C@@H]1O[C@@H](C)[C@@H]([C@H]([C@H]1O)O)O)O | 5486199 |
| 628 | IMPHY006145 | p-Cymene | Cc1ccc(cc1)C(C)C | 7463 |
| 629 | IMPHY006148 | 4-Hydroxyacetophenone | CC(=O)c1ccc(cc1)O | 7469 |
| 630 | IMPHY006325 | Myrtenal | O=CC1=CCC2CC1C2(C)C | 61130 |
| 631 | IMPHY006347 | Hexanal | CCCCCC=O | 6184 |
| 632 | IMPHY006417 | 2,6-Dimethyl-2,4,6-octatriene | C/C=C(/C=C/C=C(C)C)C | 5368821 |
| 633 | IMPHY006428 | (R)-Lavandulol | OC[C@@H](C(=C)C)CC=C(C)C | 5464156 |
| 634 | IMPHY006550 | Thymol | Cc1ccc(c(c1)O)C(C)C | 6989 |
| 635 | IMPHY006653 | Carnosic acid | OC(=O)[C@@]12CCCC([C@@H]2CCc2c1c(O)c(c(c2)C(C)C)O)(C)C | 65126 |
| 636 | IMPHY006696 | Methyleugenol | C=CCc1ccc(c(c1)OC)OC | 7127 |
| 637 | IMPHY006700 | Methyl benzoate | COC(=O)c1ccccc1 | 7150 |
| 638 | IMPHY006709 | Acetyleugenol | C=CCc1ccc(c(c1)OC)OC(=O)C | 7136 |
| 639 | IMPHY006750 | Hesperetin | COc1ccc(cc1O)[C@@H]1CC(=O)c2c(O1)cc(cc2O)O | 72281 |
| 640 | IMPHY006940 | 3,4-Dihydroxybenzaldehyde | O=Cc1ccc(c(c1)O)O | 8768 |
| 641 | IMPHY006944 | Estragole | COc1ccc(cc1)CC=C | 8815 |
| 642 | IMPHY006948 | beta-Terpineol | CC(=C)C1CCC(CC1)(C)O | 8748 |
| 643 | IMPHY006950 | Tricyclene | CC12C3C1CC(C2(C)C)C3 | 79035 |
| 644 | IMPHY006965 | alpha,alpha-Dimethyl-4-methylenecyclohexanemethanol | CC(C1CCC(=C)CC1)(O)C | 81722 |
| 645 | IMPHY007067 | Linalyl acetate | C=CC(OC(=O)C)(CCC=C(C)C)C | 8294 |
| 646 | IMPHY007200 | 2-Octanone | CCCCCCC(=O)C | 8093 |
| 647 | IMPHY007201 | Carvacrol methyl ether | COc1cc(ccc1C)C(C)C | 80790 |
| 648 | IMPHY007327 | Palmitic acid | CCCCCCCCCCCCCCCC(=O)O | 985 |
| 649 | IMPHY007331 | 6-Methyl-5-hepten-2-one | CC(=O)CCC=C(C)C | 9862 |
| 650 | IMPHY007366 | alpha-Santalene | CC(=CCCC1(C)C2CC3C1(C)C3C2)C | 94164 |
| 651 | IMPHY007376 | beta-Cubebene | CC([C@@H]1CC[C@H]([C@]23[C@H]1[C@H]2C(=C)CC3)C)C | 93081 |
| 652 | IMPHY007421 | Citronellyl acetate | CC(CCC=C(C)C)CCOC(=O)C | 9017 |
| 653 | IMPHY007520 | Viridiflorene | C[C@@H]1CCC2=C(C)CC[C@@H]3[C@H]([C@H]12)C3(C)C | 10910653 |
| 654 | IMPHY007528 | Cadinane | C[C@H]1CC[C@@H]2[C@@H](C1)[C@@H](CC[C@@H]2C)C(C)C | 9548708 |
| 655 | IMPHY007747 | Carvacryl acetate | CC(=O)Oc1cc(ccc1C)C(C)C | 80792 |
| 656 | IMPHY007840 | Spathulenol | C=C1CC[C@@H]2[C@H]([C@H]3[C@H]1CC[C@]3(C)O)C2(C)C | 92231 |
| 657 | IMPHY008150 | 1-Methyl-4-(prop-1-en-2-yl)benzene | Cc1ccc(cc1)C(=C)C | 62385 |
| 658 | IMPHY008724 | Isorhamnetin | COc1cc(ccc1O)c1oc2cc(O)cc(c2c(=O)c1O)O | 5281654 |
| 659 | IMPHY008991 | Benzyl acetate | CC(=O)OCc1ccccc1 | 8785 |
| 660 | IMPHY009729 | Verbenene | C=C1C=CC2CC1C2(C)C | 6427476 |
| 661 | IMPHY009743 | beta-Gurjunene | C[C@@H]1CC[C@@H]2[C@H]([C@H]3[C@@H]1CCC3=C)C2(C)C | 6450812 |
| 662 | IMPHY009757 | 6,6-Dimethyl-2-methylenebicyclo[3.1.1]hept-3-yl acetate | CC(=O)OC1CC2CC(C1=C)C2(C)C | 102553 |
| 663 | IMPHY009770 | Caryophyllenyl alcohol | CC1CCC(O)/C(=CCC2C1CC2(C)C)/C | 91704770 |
| 664 | IMPHY009832 | Lavandulyl acetate | CC(=O)OCC(C(=C)C)CC=C(C)C | 30247 |
| 665 | IMPHY009853 | Naphthalene | c1ccc2c(c1)cccc2 | 931 |
| 666 | IMPHY009871 | Isoterpinolene | CC1CCC(=C(C)C)C=C1 | 102443 |
| 667 | IMPHY009946 | Benzaldehyde | O=Cc1ccccc1 | 240 |
| 668 | IMPHY009985 | Hexyl tiglate | CCCCCCOC(=O)/C(=C/C)/C | 637523 |
| 669 | IMPHY010072 | Eucalyptol | CC12CCC(CC1)C(O2)(C)C | 2758 |
| 670 | IMPHY010080 | beta-Elemene | C=C[C@]1(C)CC[C@H](C[C@H]1C(=C)C)C(=C)C | 6918391 |
| 671 | IMPHY010097 | Benzyl benzoate | O=C(c1ccccc1)OCc1ccccc1 | 2345 |
| 672 | IMPHY010550 | Naringetol | Oc1ccc(cc1)[C@@H]1CC(=O)c2c(O1)cc(cc2O)O | 439246 |
| 673 | IMPHY010781 | Limonene oxide, cis-(-)- | CC(=C)[C@H]1CC[C@]2([C@@H](C1)O2)C | 6452061 |
| 674 | IMPHY010840 | cis-Myrtanol | OC[C@H]1CC[C@@H]2C[C@H]1C2(C)C | 117419 |
| 675 | IMPHY010887 | Thymol acetate | CC(=O)Oc1cc(C)ccc1C(C)C | 68252 |
| 676 | IMPHY011004 | p-Mentha-1,3,8-triene | CC1=CC=C(CC1)C(=C)C | 176983 |
| 677 | IMPHY011058 | 3-Cyclohexen-1-ol, 4-methyl-1-(1-methylethyl)-, acetate | CC(=O)OC1(CCC(=CC1)C)C(C)C | 20960 |
| 678 | IMPHY011187 | (+)-trans-Myrtanol | OC[C@@H]1CC[C@@H]2C[C@H]1C2(C)C | 84880 |
| 679 | IMPHY011239 | Carnosol | O=C1O[C@H]2C[C@@H]3[C@]1(CCCC3(C)C)c1c2cc(c(c1O)O)C(C)C | 442009 |
| 680 | IMPHY011273 | alpha-Amyrenone | C[C@@H]1CC[C@]2([C@@H]([C@H]1C)C1=CC[C@H]3[C@@]([C@@]1(CC2)C)(C)CC[C@@H]1[C@]3(C)CCC(=O)C1(C)C)C | 12306155 |
| 681 | IMPHY011392 | 3-Carene | CC1=CCC2C(C1)C2(C)C | 26049 |
| 682 | IMPHY011396 | 4-Carvomenthenol | CC1=CCC(CC1)(O)C(C)C | 11230 |
| 683 | IMPHY011407 | alpha-Campholenal | O=CC[C@H]1CC=C(C1(C)C)C | 1252759 |
| 684 | IMPHY011435 | Isorosmanol | O[C@H]1[C@@H]2OC(=O)[C@]3([C@@H]1C(C)(C)CCC3)c1c2cc(C(C)C)c(c1O)O | 13820511 |
| 685 | IMPHY011455 | Cadina-1,4-diene | CC1=CC2C(=CC1)[C@@H](C)CCC2C(C)C | 6427091 |
| 686 | IMPHY011519 | alpha-Terpinyl acetate | CC(=O)OC(C1CCC(=CC1)C)(C)C | 111037 |
| 687 | IMPHY011552 | (1R)-2-methyl-5-propan-2-ylbicyclo[3.1.0]hex-2-ene | CC1=CCC2([C@@H]1C2)C(C)C | 6451618 |
| 688 | IMPHY011562 | 2-Hexenal | CCC/C=C/C=O | 5281168 |
| 689 | IMPHY011564 | Germacra-1(10),5-dien-4-ol | C/C/1=C/CC[C@@](C)(O)/C=C[C@@H](CC1)C(C)C | 101589676 |
| 690 | IMPHY011581 | alpha-Selinene | CC1=CCC[C@]2([C@H]1C[C@@H](CC2)C(=C)C)C | 10856614 |
| 691 | IMPHY011586 | (S,1Z,6Z)-8-Isopropyl-1-methyl-5-methylenecyclodeca-1,6-diene | C/C/1=C/CCC(=C)/C=C[C@@H](CC1)C(C)C | 91723653 |
| 692 | IMPHY011590 | d-Borneol | O[C@@H]1C[C@H]2C([C@@]1(C)CC2)(C)C | 61060 |
| 693 | IMPHY011599 | Terpinolene | CC1=CCC(=C(C)C)CC1 | 11463 |
| 694 | IMPHY011643 | alpha-Terpinene | CC1=CC=C(CC1)C(C)C | 7462 |
| 695 | IMPHY011647 | Geranyl acetate | C/C(=CCOC(=O)C)/CCC=C(C)C | 1549026 |
| 696 | IMPHY011648 | Neryl acetate | C/C(=C/COC(=O)C)/CCC=C(C)C | 1549025 |
| 697 | IMPHY011657 | cis-beta-Farnesene | C=CC(=C)CC/C=C(CCC=C(C)C)/C | 5317319 |
| 698 | IMPHY011658 | beta-Farnesene | C=CC(=C)CC/C=C(/CCC=C(C)C)C | 5281517 |
| 699 | IMPHY011659 | alpha-Muurolene | CC1=C[C@@H]2[C@H](CC1)C(=CC[C@H]2C(C)C)C | 12306047 |
| 700 | IMPHY011660 | (+)-alpha-Cadinene | CC1=C[C@@H]2[C@@H](CC1)C(=CC[C@H]2C(C)C)C | 12306048 |
| 701 | IMPHY011667 | alpha-Gurjunene | C[C@@H]1CC[C@@H]2[C@H](C3=C(CC[C@H]13)C)C2(C)C | 15560276 |
| 702 | IMPHY011709 | alpha-Eudesmol | CC1=CCC[C@]2([C@H]1C[C@@H](CC2)C(O)(C)C)C | 92762 |
| 703 | IMPHY011749 | Humulene epoxide II | C/C/1=CCC(C)(C)/C=C/C[C@@]2([C@@H](CC1)O2)C | 10704181 |
| 704 | IMPHY011761 | Humulene | C/C/1=CCC(C)(C)/C=C/C/C(=C/CC1)/C | 5281520 |
| 705 | IMPHY011762 | cis-Anethole | C/C=Cc1ccc(cc1)OC | 1549040 |
| 706 | IMPHY011763 | Anethole | C/C=C/c1ccc(cc1)OC | 637563 |
| 707 | IMPHY011789 | Citral | O=C/C=C(/CCC=C(C)C)C | 638011 |
| 708 | IMPHY011790 | Neral | O=C/C=C(CCC=C(C)C)/C | 643779 |
| 709 | IMPHY011792 | gamma-Muurolene | CC1=C[C@@H]2[C@H](CC1)C(=C)CC[C@H]2C(C)C | 12313020 |
| 710 | IMPHY011793 | (+)-gamma-Cadinene | CC1=C[C@@H]2[C@@H](CC1)C(=C)CC[C@H]2C(C)C | 6432404 |
| 711 | IMPHY011817 | alpha-Farnesene | C=C/C(=C/C/C=C(/CCC=C(C)C)C)/C | 5281516 |
| 712 | IMPHY011826 | Oleanolic acid | O[C@H]1CC[C@]2([C@H](C1(C)C)CC[C@@]1([C@@H]2CC=C2[C@@]1(C)CC[C@@]1([C@H]2CC(C)(C)CC1)C(=O)O)C)C | 10494 |
| 713 | IMPHY011844 | Chlorogenic acid | O=C(O[C@@H]1C[C@@](O)(C[C@H]([C@H]1O)O)C(=O)O)/C=C/c1ccc(c(c1)O)O | 1794427 |
| 714 | IMPHY011872 | (4E,7E)-1,5,9,9-Tetramethyl-12-oxabicyclo[9.1.0]dodeca-4,7-diene | C/C/1=C/CCC2(C)OC2CC(/C=CC1)(C)C | 22559443 |
| 715 | IMPHY011873 | Humulene epoxide | C/C/1=CCCC2(C)OC2CC(/C=C/C1)(C)C | 5352470 |
| 716 | IMPHY011879 | Moupinamide | COc1cc(/C=C/C(=O)NCCc2ccc(cc2)O)ccc1O | 5280537 |
| 717 | IMPHY011880 | Ursolic acid | C[C@@H]1CC[C@]2([C@@H]([C@H]1C)C1=CC[C@H]3[C@@]([C@@]1(CC2)C)(C)CC[C@@H]1[C@]3(C)CC[C@@H](C1(C)C)O)C(=O)O | 64945 |
| 718 | IMPHY011882 | Cinnamaldehyde | O=C/C=C/c1ccccc1 | 637511 |
| 719 | IMPHY011884 | Pulegone | C[C@@H]1CCC(=C(C)C)C(=O)C1 | 442495 |
| 720 | IMPHY011890 | Elemol | C=C[C@]1(C)CC[C@H](C[C@H]1C(=C)C)C(O)(C)C | 92138 |
| 721 | IMPHY011896 | Valencene | CC(=C)[C@@H]1CCC2=CCC[C@H]([C@@]2(C1)C)C | 9855795 |
| 722 | IMPHY011901 | Thujone | O=C1C[C@]2([C@@H]([C@H]1C)C2)C(C)C | 261491 |
| 723 | IMPHY011902 | beta-Thujone | O=C1C[C@]2([C@@H]([C@@H]1C)C2)C(C)C | 91456 |
| 724 | IMPHY011933 | Caffeic acid | OC(=O)/C=C/c1ccc(c(c1)O)O | 689043 |
| 725 | IMPHY011957 | (+)-delta-Cadinene | CC1=C[C@@H]2C(=C(C)CC[C@H]2C(C)C)CC1 | 441005 |
| 726 | IMPHY011965 | (+)-beta-Phellandrene | CC([C@@H]1CCC(=C)C=C1)C | 442484 |
| 727 | IMPHY011973 | (-)-cis-Carveol | CC(=C)[C@@H]1CC=C([C@@H](C1)O)C | 330573 |
| 728 | IMPHY011983 | cis-Cinnamaldehyde | O=C/C=Cc1ccccc1 | 6428995 |
| 729 | IMPHY011988 | (-)-trans-Carveol | CC(=C)[C@@H]1CC=C([C@H](C1)O)C | 94221 |
| 730 | IMPHY012020 | Acetylcholine | CC(=O)OCC[N+](C)(C)C | 187 |
| 731 | IMPHY012036 | Camphor | O=C1CC2C(C1(C)CC2)(C)C | 2537 |
| 732 | IMPHY012058 | Linalool | C=CC(CCC=C(C)C)(O)C | 6549 |
| 733 | IMPHY012061 | alpha-Pinene | CC1=CCC2CC1C2(C)C | 6654 |
| 734 | IMPHY012075 | Carvone | CC(=C)C1CC=C(C(=O)C1)C | 7439 |
| 735 | IMPHY012086 | Citronellal | O=CCC(CCC=C(C)C)C | 7794 |
| 736 | IMPHY012104 | Citronellol | OCCC(CCC=C(C)C)C | 8842 |
| 737 | IMPHY012147 | beta-Pinene | C=C1CCC2CC1C2(C)C | 14896 |
| 738 | IMPHY012152 | alpha-Fenchol | O[C@H]1[C@@]2(C)CC[C@@H](C1(C)C)C2 | 439711 |
| 739 | IMPHY012160 | alpha-Terpineol | CC1=CCC(CC1)C(O)(C)C | 17100 |
| 740 | IMPHY012165 | Sabinene | C=C1CCC2(C1C2)C(C)C | 18818 |
| 741 | IMPHY012168 | (1S,2S,6S,7R,8R)-1,3-dimethyl-8-propan-2-yltricyclo[4.4.0.02,7]dec-3-ene | CC([C@H]1CC[C@]2([C@@H]3[C@@H]1[C@H]2C(=CC3)C)C)C | 101607926 |
| 742 | IMPHY012175 | D-Limonene | CC1=CC[C@@H](CC1)C(=C)C | 440917 |
| 743 | IMPHY012178 | p-Menthan-3-one | CC1CCC(C(=O)C1)C(C)C | 6986 |
| 744 | IMPHY012198 | Verbenol | CC1=CC(O)C2CC1C2(C)C | 61126 |
| 745 | IMPHY012252 | Eriocitrin | O[C@@H]1[C@@H](O)[C@H](Oc2cc(O)c3c(c2)O[C@@H](CC3=O)c2ccc(c(c2)O)O)O[C@@H]([C@H]1O)CO[C@@H]1O[C@@H](C)[C@@H]([C@H]([C@H]1O)O)O | 83489 |
| 746 | IMPHY012254 | Isopinocamphone | O=C1C[C@H]2C[C@@H]([C@@H]1C)C2(C)C | 84532 |
| 747 | IMPHY012265 | (1r,3s,5r)-6,6-Dimethyl-2-methylidenebicyclo[3.1.1]heptan-3-ol | C=C1[C@@H](O)C[C@H]2C[C@@H]1C2(C)C | 88302 |
| 748 | IMPHY012278 | Lyral | O=CC1CCC(=CC1)CCCC(O)(C)C | 91604 |
| 749 | IMPHY012279 | alpha-Curcumene | CC(=CCCC(c1ccc(cc1)C)C)C | 92139 |
| 750 | IMPHY012292 | trans-Sabinol | CC([C@]12CC2C(=C)[C@@H](C1)O)C | 6429076 |
| 751 | IMPHY012487 | Menthofuran | CC1CCc2c(C1)occ2C | 329983 |
| 752 | IMPHY012585 | delta-Cadinol | CC1=C[C@@H]2[C@H](CC1)[C@](C)(O)CC[C@H]2C(C)C | 3084311 |
| 753 | IMPHY012586 | (-)-alpha-Cadinol | CC1=CC2C(CC1)[C@@](C)(O)CC[C@@H]2C(C)C | 6431302 |
| 754 | IMPHY012589 | 3-(1,5-Dimethyl-4-hexenyl)-6-methylene-1-cyclohexene | CC(C1CCC(=C)C=C1)CCC=C(C)C | 519764 |
| 755 | IMPHY012654 | Nerol | OC/C=C(CCC=C(C)C)/C | 643820 |
| 756 | IMPHY012665 | Levomenol | CC(=CCC[C@@]([C@H]1CCC(=CC1)C)(O)C)C | 442343 |
| 757 | IMPHY012667 | Caryophyllene oxide | C=C1CC[C@H]2O[C@@]2(CC[C@@H]2[C@@H]1CC2(C)C)C | 1742210 |
| 758 | IMPHY012739 | (Z)-beta-Ocimene | C=C/C(=CCC=C(C)C)/C | 5320250 |
| 759 | IMPHY012914 | trans-Sabinyl acetate | CC(=O)O[C@H]1C[C@@]2(C(C1=C)C2)C(C)C | 6430313 |
| 760 | IMPHY012920 | 2-Furanmethanol, 5-ethenyltetrahydro-alpha,alpha,5-trimethyl-, cis- | C=C[C@@]1(C)CC[C@H](O1)C(O)(C)C | 11116492 |
| 761 | IMPHY013080 | alpha-Calacorene | CC([C@@H]1CC=C(c2c1cc(C)cc2)C)C | 12302243 |
| 762 | IMPHY013093 | delta-Elemene | C=C[C@@]1(C)CCC(=C[C@@H]1C(=C)C)C(C)C | 12309449 |
| 763 | IMPHY013133 | (Z)-p-Menth-2-en-1-ol | CC([C@@H]1CC[C@](C=C1)(C)O)C | 13918681 |
| 764 | IMPHY013836 | Fenchone | O=C1C2(C)CCC(C1(C)C)C2 | 14525 |
| 765 | IMPHY013838 | 3,7-Dimethyloct-6-en-3-ol | CCC(CCC=C(C)C)(O)C | 86749 |
| 766 | IMPHY014115 | 2-Isopropyl-1,4-hexadiene | C/C=C/CC(=C)C(C)C | 6429324 |
| 767 | IMPHY014708 | beta-Selinene | C=C1CCC[C@]2([C@H]1C[C@@H](CC2)C(=C)C)C | 442393 |
| 768 | IMPHY014721 | 1-epi-Bicyclosesquiphellandrene | C=C1CCC2C(=C1)C(CCC2C)C(C)C | 521496 |
| 769 | IMPHY014732 | 14-Hydroxy-9-epi-beta-Caryophyllene | OCC1(C)C[C@@H]2[C@H]1CC/C(=CCCC2=C)/C | 91747230 |
| 770 | IMPHY014801 | Zizanene | CC1=C[C@@H]2[C@H](CC1)C(=CC[C@@H]2C(C)C)C | 12306046 |
| 771 | IMPHY014806 | Caswell No. 264AB | CC([C@@H]1CC[C@H]([C@]23[C@H]1[C@H]2C(=CC3)C)C)C | 442359 |
| 772 | IMPHY014811 | alpha-Phellandrene | CC1=CCC(C=C1)C(C)C | 7460 |
| 773 | IMPHY014817 | Aromadendrene | CC1CCC2C1C1C(C1(C)C)CCC2=C | 91354 |
| 774 | IMPHY014831 | beta-Caryophyllene | C/C/1=CCCC(=C)[C@@H]2[C@@H](CC1)C(C2)(C)C | 5281515 |
| 775 | IMPHY014835 | (E)-beta-ocimene | C=C/C(=C/CC=C(C)C)/C | 5281553 |
| 776 | IMPHY014847 | Bornyl acetate | CC(=O)OC1CC2C(C1(C)CC2)(C)C | 6448 |
| 777 | IMPHY014852 | Camphene | C=C1C2CCC(C1(C)C)C2 | 6616 |
| 778 | IMPHY014857 | Cedrol | C[C@@H]1CC[C@@H]2[C@@]31CC[C@@]([C@H](C3)C2(C)C)(C)O | 65575 |
| 779 | IMPHY014863 | cis-alpha-Bergamotene | CC(=CCCC1(C)[C@@H]2CC=C([C@H]1C2)C)C | 91753502 |
| 780 | IMPHY014865 | Calamenene | CC([C@@H]1CC[C@@H](c2c1cc(C)cc2)C)C | 6429077 |
| 781 | IMPHY014874 | cis-Sabinene hydrate | C[C@@H]1CC[C@@]2(C1C2)C(C)C | 101629835 |
| 782 | IMPHY014877 | (S)-cis-Verbenol | CC1=C[C@H](O)[C@H]2C[C@@H]1C2(C)C | 87839 |
| 783 | IMPHY014885 | 1-Isopropyl-4,7-dimethyl-1,3,4,5,6,8a-hexahydro-4a(2H)-naphthalenol | CC1=CC2C(CC1)(O)C(C)CCC2C(C)C | 519857 |
| 784 | IMPHY014906 | Cedrelanol | CC1=C[C@@H]2[C@@H](CC1)[C@@](C)(O)CC[C@H]2C(C)C | 160799 |
| 785 | IMPHY014914 | Fenchol | OC1C2(C)CCC(C1(C)C)C2 | 15406 |
| 786 | IMPHY014923 | Geraniol | OC/C=C(/CCC=C(C)C)C | 637566 |
| 787 | IMPHY014960 | (+)-Longifolene | C=C1[C@H]2CC[C@H]3[C@]1(C)CCCC([C@@H]23)(C)C | 1796220 |
| 788 | IMPHY014988 | Limonene | CC1=CCC(CC1)C(=C)C | 22311 |
| 789 | IMPHY015003 | Menthol | CC1CCC(C(C1)O)C(C)C | 1254 |
| 790 | IMPHY015004 | Menthone | C[C@@H]1CC[C@H](C(=O)C1)C(C)C | 26447 |
| 791 | IMPHY015022 | Nerolidol | C=CC(CC/C=C(/CCC=C(C)C)C)(O)C | 5284507 |
| 792 | IMPHY015040 | 3-Pinanone | O=C1CC2CC(C1C)C2(C)C | 11038 |
| 793 | IMPHY015042 | Piperitone | CC1=CC(=O)C(CC1)C(C)C | 6987 |
| 794 | IMPHY015063 | Sabinyl acetate | CC(=O)OC1CC2(C(C1=C)C2)C(C)C | 94266 |
| 795 | IMPHY015095 | 2-Cyclohexen-1-ol, 1-methyl-4-(1-methylethyl)-, trans- | CC([C@@H]1CC[C@@](C=C1)(C)O)C | 122484 |
| 796 | IMPHY015098 | trans-Verbenol | CC1=C[C@H](O)[C@@H]2C[C@H]1C2(C)C | 89664 |
| 797 | IMPHY015123 | alpha-Copaene | CC([C@@H]1CC[C@]2([C@@H]3[C@H]1C2C(=CC3)C)C)C | 70678558 |
| 798 | IMPHY015128 | T-Muurolol | CC1=C[C@@H]2[C@H](CC1)[C@@](C)(O)CC[C@H]2C(C)C | 3084331 |
| 799 | IMPHY015233 | 2,5-Dimethylstyrene | C=Cc1cc(C)ccc1C | 16265 |
| 800 | IMPHY015011 | Methylisoeugenol | C/C=C/c1ccc(c(c1)OC)OC | 637776 |
| 801 | IMPHY015466 | Tricyclo(3.3.1.13,7)decan-1-acetamide | NC(=O)CC12CC3CC(C2)CC(C1)C3 | 86819 |
| 802 | IMPHY015510 | Bicyclo[3.1.0]hex-3-EN-2-OL | OC1C=CC2C1C2 | 14909256 |
| 803 | IMPHY015584 | Cyclodecasiloxane, eicosamethyl- | C[Si]1(C)O[Si](C)(C)O[Si](C)(C)O[Si](C)(C)O[Si](C)(C)O[Si](O[Si](O[Si](O[Si](O[Si](O1)(C)C)(C)C)(C)C)(C)C)(C)C | 519601 |
| 804 | IMPHY015591 | Octadecamethylcyclononasiloxane | C[Si]1(C)O[Si](C)(C)O[Si](C)(C)O[Si](C)(C)O[Si](C)(C)O[Si](O[Si](O[Si](O[Si](O1)(C)C)(C)C)(C)C)(C)C | 11172 |
| 805 | IMPHY015718 | Hexadecamethylheptasiloxane | C[Si](O[Si](O[Si](O[Si](C)(C)C)(C)C)(C)C)(O[Si](O[Si](O[Si](C)(C)C)(C)C)(C)C)C | 10912 |
| 806 | IMPHY015749 | Isoamyl isovalerate | CC(CCOC(=O)CC(C)C)C | 12613 |
| 807 | IMPHY016012 | Allo-Aromadendrene | C[C@@H]1CC[C@H]2[C@@H]1C1C(C1(C)C)CCC2=C | 42608158 |
| 808 | IMPHY016027 | trans-Sabinene hydrate | CC([C@@]12CC[C@](C2C1)(C)O)C | 12315151 |
| 809 | IMPHY016054 | trans-alpha-Bergamotene | CC(=CCC[C@]1(C)[C@H]2CC=C([C@@H]1C2)C)C | 6429302 |
| 810 | IMPHY016950 | Norcamphor | O=C1CC2CC1CC2 | 10345 |
| 811 | IMPHY017040 | Bicyclo[3.1.1]heptan-3-one | O=C1CC2CC(C1)C2 | 12852568 |
| 812 | IMPHY017669 | p-Menthan-8-yl acetate | CC1CCC(CC1)C(OC(=O)C)(C)C | 6631 |
| 813 | IMPHY017702 | Bicyclo[3.1.1]hept-3-en-2-one | O=C1C=CC2CC1C2 | 71360359 |
|  |  |  |  |  |
|  |  |  |  |  |
|  |  |  |  |  |
|  |  |  |  |  |
| **PLANT NAME : *Salvia officinalis*** | | | | |
| **Serial No** | **IMPPAT Phytichemical identifier** | **Phytochemical Name** | **SMILES:** | **CID** |
| 1 | IMPHY000060 | Myristic acid | CCCCCCCCCCCCCC(=O)O | 11005 |
| 2 | IMPHY000099 | Myrtenol | OCC1=CCC2CC1C2(C)C | 10582 |
| 3 | IMPHY000112 | Isophyto | C=CC(CCCC(CCCC(CCCC(C)C)C)C)(O)C | 10453 |
| 4 | IMPHY000308 | Hexadecane | CCCCCCCCCCCCCCCC | 11006 |
| 5 | IMPHY000399 | beta-Bisabolene | CC(=CCCC(=C)[C@H]1CCC(=CC1)C)C | 10104370 |
| 6 | IMPHY001246 | Carvacrol | CC(c1ccc(c(c1)O)C)C | 10364 |
| 7 | IMPHY001658 | Thymol methyl ether | COc1cc(C)ccc1C(C)C | 14104 |
| 8 | IMPHY001828 | 3-Octanol | CCCCCC(CC)O | 11527 |
| 9 | IMPHY001915 | Octadecane | CCCCCCCCCCCCCCCCCC | 11635 |
| 10 | IMPHY002072 | Pinocarvone | C=C1C(=O)CC2CC1C2(C)C | 121719 |
| 11 | IMPHY002652 | (1R,4aS,5R,8aS)-3-methyl-8-methylidene-5-propan-2-yl-2,4a,5,6,7,8a-hexahydro-1H-naphthalen-1-ol | CC1=C[C@H]2[C@H]([C@@H](C1)O)C(=C)CC[C@@H]2C(C)C | 13874489 |
| 12 | IMPHY002825 | 2-(4-Methylphenyl)propan-2-ol | Cc1ccc(cc1)C(O)(C)C | 14529 |
| 13 | IMPHY003485 | Myrcene | C=CC(=C)CCC=C(C)C | 31253 |
| 14 | IMPHY003536 | Eugenol | C=CCc1ccc(c(c1)OC)O | 3314 |
| 15 | IMPHY003616 | Bicyclogermacrene | C/C/1=CCC/C(=C/[C@H]2[C@@H](CC1)C2(C)C)/C | 13894537 |
| 16 | IMPHY003956 | (+)-gamma-Gurjunene | C[C@@H]1CC[C@H]2C1=C[C@@H](CC[C@H]2C)C(=C)C | 15560285 |
| 17 | IMPHY003977 | (-)-beta-Bourbonene | CC([C@@H]1CC[C@@]2([C@H]1[C@H]1C(=C)CC[C@@H]21)C)C | 62566 |
| 18 | IMPHY003982 | gamma-Terpinene | CC1=CCC(=CC1)C(C)C | 7461 |
| 19 | IMPHY004017 | Manool | C=C[C@@](CC[C@H]1C(=C)CC[C@@H]2[C@]1(C)CCCC2(C)C)(O)C | 3034394 |
| 20 | IMPHY004077 | Verbenone | CC1=CC(=O)C2CC1C2(C)C | 29025 |
| 21 | IMPHY004669 | Santolina triene | C=CC(C(=C)C)C=C(C)C | 519872 |
| 22 | IMPHY004961 | (E)-2,5,5-Trimethylhepta-3,6-dien-2-ol | C=CC(/C=C/C(O)(C)C)(C)C | 5315406 |
| 23 | IMPHY005345 | 1-Octen-3-OL | CCCCCC(C=C)O | 18827 |
| 24 | IMPHY005400 | Sclareol | C=C[C@@](CC[C@H]1[C@](C)(O)CC[C@@H]2[C@]1(C)CCCC2(C)C)(O)C | 163263 |
| 25 | IMPHY005618 | Germacrene B | C/C/1=CCC/C(=C/CC(=C(C)C)CC1)/C | 5281519 |
| 26 | IMPHY005784 | Oxacyclohexadecan-2-one | O=C1CCCCCCCCCCCCCCO1 | 235414 |
| 27 | IMPHY006145 | p-Cymene | Cc1ccc(cc1)C(C)C | 7463 |
| 28 | IMPHY006550 | Thymol | Cc1ccc(c(c1)O)C(C)C | 6989 |
| 29 | IMPHY006696 | Methyleugenol | C=CCc1ccc(c(c1)OC)OC | 7127 |
| 30 | IMPHY006719 | (1aR,7S,7aS,7bR)-1,1,4,7-tetramethyl-2,3,5,6,7a,7b-hexahydro-1aH-cyclopropa[h]azulen-7-ol | CC1=C2CC[C@]([C@H]2[C@H]2[C@@H](CC1)C2(C)C)(C)O | 102303030 |
| 31 | IMPHY006944 | Estragole | COc1ccc(cc1)CC=C | 8815 |
| 32 | IMPHY006948 | beta-Terpineol | CC(=C)C1CCC(CC1)(C)O | 8748 |
| 33 | IMPHY006950 | Tricyclene | CC12C3C1CC(C2(C)C)C3 | 79035 |
| 34 | IMPHY007067 | Linalyl acetate | C=CC(OC(=O)C)(CCC=C(C)C)C | 8294 |
| 35 | IMPHY007201 | Carvacrol methyl ether | COc1cc(ccc1C)C(C)C | 80790 |
| 36 | IMPHY007276 | Nonan-1-ol | CCCCCCCCCO | 8914 |
| 37 | IMPHY007327 | Palmitic acid | CCCCCCCCCCCCCCCC(=O)O | 985 |
| 38 | IMPHY007376 | beta-Cubebene | CC([C@@H]1CC[C@H]([C@]23[C@H]1[C@H]2C(=C)CC3)C)C | 93081 |
| 39 | IMPHY007520 | Viridiflorene | C[C@@H]1CCC2=C(C)CC[C@@H]3[C@H]([C@H]12)C3(C)C | 10910653 |
| 40 | IMPHY007747 | Carvacryl acetate | CC(=O)Oc1cc(ccc1C)C(C)C | 80792 |
| 41 | IMPHY007840 | Spathulenol | C=C1CC[C@@H]2[C@H]([C@H]3[C@H]1CC[C@]3(C)O)C2(C)C | 92231 |
| 42 | IMPHY007978 | Hexyl 2-methylbutanoate | CCCCCCOC(=O)C(CC)C | 24838 |
| 43 | IMPHY008150 | 1-Methyl-4-(prop-1-en-2-yl)benzene | Cc1ccc(cc1)C(=C)C | 62385 |
| 44 | IMPHY008701 | 3,3,6-Trimethylhepta-1,5-dien-4-ol | C=CC(C(C=C(C)C)O)(C)C | 100197 |
| 45 | IMPHY008920 | Artemisyl acetate | C=CC(C(C=C(C)C)OC(=O)C)(C)C | 524254 |
| 46 | IMPHY009186 | Safficinolide | O=CC12CCCC(C2C(=O)Oc2c1c(C=O)cc(c2O)C(C)C)(C)C | 85152699 |
| 47 | IMPHY009296 | Sageone | O=C1CCC(C2=C1c1c(CC2)cc(c(c1O)O)C(C)C)(C)C | 6481824 |
| 48 | IMPHY009355 | Tetracosane | CCCCCCCCCCCCCCCCCCCCCCCC | 12592 |
| 49 | IMPHY009377 | Pentacosane | CCCCCCCCCCCCCCCCCCCCCCCCC | 12406 |
| 50 | IMPHY009389 | Pentadecane | CCCCCCCCCCCCCCC | 12391 |
| 51 | IMPHY009490 | Tricosane | CCCCCCCCCCCCCCCCCCCCCCC | 12534 |
| 52 | IMPHY009687 | Sclarene | C=CC(=C)CC[C@H]1C(=C)CC[C@@H]2[C@]1(C)CCCC2(C)C | 11323257 |
| 53 | IMPHY009699 | trans-Isolongifolanone | O=C1CCC([C@]23C1C(C)(C)[C@@H](C3)CC2)(C)C | 6427070 |
| 54 | IMPHY009707 | Santolina alcohol | C=CC(C(O)(C)C)C=C(C)C | 519843 |
| 55 | IMPHY009743 | beta-Gurjunene | C[C@@H]1CC[C@@H]2[C@H]([C@H]3[C@@H]1CCC3=C)C2(C)C | 6450812 |
| 56 | IMPHY009759 | cis-Verbenyl acetate | CC(=O)O[C@@H]1C=C(C)[C@@H]2C[C@H]1C2(C)C | 6427494 |
| 57 | IMPHY009765 | beta-Calacorene | CC(C1CCC(=C)c2c1cc(C)cc2)C | 529621 |
| 58 | IMPHY009872 | p-Mentha-3,8-diene | CC1CCC(=CC1)C(=C)C | 521851 |
| 59 | IMPHY010072 | Eucalyptol | CC12CCC(CC1)C(O2)(C)C | 2758 |
| 60 | IMPHY010080 | beta-Elemene | C=C[C@]1(C)CC[C@H](C[C@H]1C(=C)C)C(=C)C | 6918391 |
| 61 | IMPHY010887 | Thymol acetate | CC(=O)Oc1cc(C)ccc1C(C)C | 68252 |
| 62 | IMPHY010977 | Hexadecan-2-one | CCCCCCCCCCCCCCC(=O)C | 29251 |
| 63 | IMPHY011051 | Abietatriene | CC(c1ccc2c(c1)CC[C@@H]1[C@]2(C)CCCC1(C)C)C | 6432211 |
| 64 | IMPHY011073 | 7-epi-Silphiperfol-5-ene | CC1CCC23C1CCC3(C)C=C(C2C)C | 74819460 |
| 65 | IMPHY011223 | iso-3-Thujyl acetate | CC(=O)OC1CC2(C(C1C)C2)C(C)C | 524251 |
| 66 | IMPHY011392 | 3-Carene | CC1=CCC2C(C1)C2(C)C | 26049 |
| 67 | IMPHY011396 | 4-Carvomenthenol | CC1=CCC(CC1)(O)C(C)C | 11230 |
| 68 | IMPHY011407 | alpha-Campholenal | O=CC[C@H]1CC=C(C1(C)C)C | 1252759 |
| 69 | IMPHY011455 | Cadina-1,4-diene | CC1=CC2C(=CC1)[C@@H](C)CCC2C(C)C | 6427091 |
| 70 | IMPHY011519 | alpha-Terpinyl acetate | CC(=O)OC(C1CCC(=CC1)C)(C)C | 111037 |
| 71 | IMPHY011552 | (1R)-2-methyl-5-propan-2-ylbicyclo[3.1.0]hex-2-ene | CC1=CCC2([C@@H]1C2)C(C)C | 6451618 |
| 72 | IMPHY011568 | alpha-Fenchyl acetate | CC(=O)O[C@@H]1[C@]2(C)CC[C@H](C1(C)C)C2 | 7530950 |
| 73 | IMPHY011586 | (S,1Z,6Z)-8-Isopropyl-1-methyl-5-methylenecyclodeca-1,6-diene | C/C/1=C/CCC(=C)/C=C[C@@H](CC1)C(C)C | 91723653 |
| 74 | IMPHY011588 | cis-3-Hexen-1-ol | OCC/C=CCC | 5281167 |
| 75 | IMPHY011590 | d-Borneol | O[C@@H]1C[C@H]2C([C@@]1(C)CC2)(C)C | 61060 |
| 76 | IMPHY011599 | Terpinolene | CC1=CCC(=C(C)C)CC1 | 11463 |
| 77 | IMPHY011643 | alpha-Terpinene | CC1=CC=C(CC1)C(C)C | 7462 |
| 78 | IMPHY011647 | Geranyl acetate | C/C(=CCOC(=O)C)/CCC=C(C)C | 1549026 |
| 79 | IMPHY011658 | beta-Farnesene | C=CC(=C)CC/C=C(/CCC=C(C)C)C | 5281517 |
| 80 | IMPHY011659 | alpha-Muurolene | CC1=C[C@@H]2[C@H](CC1)C(=CC[C@H]2C(C)C)C | 12306047 |
| 81 | IMPHY011660 | (+)-alpha-Cadinene | CC1=C[C@@H]2[C@@H](CC1)C(=CC[C@H]2C(C)C)C | 12306048 |
| 82 | IMPHY011667 | alpha-Gurjunene | C[C@@H]1CC[C@@H]2[C@H](C3=C(CC[C@H]13)C)C2(C)C | 15560276 |
| 83 | IMPHY011749 | Humulene epoxide II | C/C/1=CCC(C)(C)/C=C/C[C@@]2([C@@H](CC1)O2)C | 10704181 |
| 84 | IMPHY011761 | Humulene | C/C/1=CCC(C)(C)/C=C/C/C(=C/CC1)/C | 5281520 |
| 85 | IMPHY011789 | Citral | O=C/C=C(/CCC=C(C)C)C | 638011 |
| 86 | IMPHY011790 | Neral | O=C/C=C(CCC=C(C)C)/C | 643779 |
| 87 | IMPHY011792 | gamma-Muurolene | CC1=C[C@@H]2[C@H](CC1)C(=C)CC[C@H]2C(C)C | 12313020 |
| 88 | IMPHY011793 | (+)-gamma-Cadinene | CC1=C[C@@H]2[C@@H](CC1)C(=C)CC[C@H]2C(C)C | 6432404 |
| 89 | IMPHY011817 | alpha-Farnesene | C=C/C(=C/C/C=C(/CCC=C(C)C)C)/C | 5281516 |
| 90 | IMPHY011872 | (4E,7E)-1,5,9,9-Tetramethyl-12-oxabicyclo[9.1.0]dodeca-4,7-diene | C/C/1=C/CCC2(C)OC2CC(/C=CC1)(C)C | 22559443 |
| 91 | IMPHY011884 | Pulegone | C[C@@H]1CCC(=C(C)C)C(=O)C1 | 442495 |
| 92 | IMPHY011890 | Elemol | C=C[C@]1(C)CC[C@H](C[C@H]1C(=C)C)C(O)(C)C | 92138 |
| 93 | IMPHY011901 | Thujone | O=C1C[C@]2([C@@H]([C@H]1C)C2)C(C)C | 261491 |
| 94 | IMPHY011902 | beta-Thujone | O=C1C[C@]2([C@@H]([C@@H]1C)C2)C(C)C | 91456 |
| 95 | IMPHY011903 | (+)-alpha-Thujone | O=C1C[C@@]2([C@H]([C@@H]1C)C2)C(C)C | 12304612 |
| 96 | IMPHY011904 | cis-Thujone | O=C1C[C@]2([C@H]([C@@H]1C)C2)C(C)C | 249286 |
| 97 | IMPHY011957 | (+)-delta-Cadinene | CC1=C[C@@H]2C(=C(C)CC[C@H]2C(C)C)CC1 | 441005 |
| 98 | IMPHY011965 | (+)-beta-Phellandrene | CC([C@@H]1CCC(=C)C=C1)C | 442484 |
| 99 | IMPHY012036 | Camphor | O=C1CC2C(C1(C)CC2)(C)C | 2537 |
| 100 | IMPHY012058 | Linalool | C=CC(CCC=C(C)C)(O)C | 6549 |
| 101 | IMPHY012061 | alpha-Pinene | CC1=CCC2CC1C2(C)C | 6654 |
| 102 | IMPHY012075 | Carvone | CC(=C)C1CC=C(C(=O)C1)C | 7439 |
| 103 | IMPHY012147 | beta-Pinene | C=C1CCC2CC1C2(C)C | 14896 |
| 104 | IMPHY012152 | alpha-Fenchol | O[C@H]1[C@@]2(C)CC[C@@H](C1(C)C)C2 | 439711 |
| 105 | IMPHY012160 | alpha-Terpineol | CC1=CCC(CC1)C(O)(C)C | 17100 |
| 106 | IMPHY012165 | Sabinene | C=C1CCC2(C1C2)C(C)C | 18818 |
| 107 | IMPHY012168 | (1S,2S,6S,7R,8R)-1,3-dimethyl-8-propan-2-yltricyclo[4.4.0.02,7]dec-3-ene | CC([C@H]1CC[C@]2([C@@H]3[C@@H]1[C@H]2C(=CC3)C)C)C | 101607926 |
| 108 | IMPHY012178 | p-Menthan-3-one | CC1CCC(C(=O)C1)C(C)C | 6986 |
| 109 | IMPHY012205 | Sabinene hydrate | CC(C12CCC(C2C1)(C)O)C | 62367 |
| 110 | IMPHY012586 | (-)-alpha-Cadinol | CC1=CC2C(CC1)[C@@](C)(O)CC[C@@H]2C(C)C | 6431302 |
| 111 | IMPHY012654 | Nerol | OC/C=C(CCC=C(C)C)/C | 643820 |
| 112 | IMPHY012667 | Caryophyllene oxide | C=C1CC[C@H]2O[C@@]2(CC[C@@H]2[C@@H]1CC2(C)C)C | 1742210 |
| 113 | IMPHY012712 | Phytol | OC/C=C(/CCC[C@@H](CCC[C@@H](CCCC(C)C)C)C)C | 5280435 |
| 114 | IMPHY012737 | (1S,4E,9S)-4,11,11-trimethyl-8-methylidenebicyclo[7.2.0]undec-4-ene | C/C/1=CCCC(=C)[C@@H]2[C@H](CC1)C(C2)(C)C | 6429301 |
| 115 | IMPHY012739 | (Z)-beta-Ocimene | C=C/C(=CCC=C(C)C)/C | 5320250 |
| 116 | IMPHY012910 | trans-Calamenene | CC([C@H]1CC[C@@H](c2c1cc(C)cc2)C)C | 6429022 |
| 117 | IMPHY012921 | gamma-Elemene | C=C[C@]1(C)CCC(=C(C)C)C[C@H]1C(=C)C | 6432312 |
| 118 | IMPHY013080 | alpha-Calacorene | CC([C@@H]1CC=C(c2c1cc(C)cc2)C)C | 12302243 |
| 119 | IMPHY013574 | 14-Hydroxy-9-epi-(E)-caryophyllene | OCC1(C)CC2C1CC/C(=CCCC2=C)/C | 5352484 |
| 120 | IMPHY013707 | 12-Methoxy-8,11,13-abietatrien-20,11-olide | COc1c2OC(=O)C3C4c2c(cc1C(C)C)CCC4C(CC3)(C)C | 131752355 |
| 121 | IMPHY013836 | Fenchone | O=C1C2(C)CCC(C1(C)C)C2 | 14525 |
| 122 | IMPHY014115 | 2-Isopropyl-1,4-hexadiene | C/C=C/CC(=C)C(C)C | 6429324 |
| 123 | IMPHY014690 | (-)-Globulol | C[C@@H]1CC[C@@H]2[C@@H]1[C@H]1[C@H](C1(C)C)CC[C@@]2(C)O | 12304985 |
| 124 | IMPHY014708 | beta-Selinene | C=C1CCC[C@]2([C@H]1C[C@@H](CC2)C(=C)C)C | 442393 |
| 125 | IMPHY014806 | Caswell No. 264AB | CC([C@@H]1CC[C@H]([C@]23[C@H]1[C@H]2C(=CC3)C)C)C | 442359 |
| 126 | IMPHY014811 | alpha-Phellandrene | CC1=CCC(C=C1)C(C)C | 7460 |
| 127 | IMPHY014817 | Aromadendrene | CC1CCC2C1C1C(C1(C)C)CCC2=C | 91354 |
| 128 | IMPHY014831 | beta-Caryophyllene | C/C/1=CCCC(=C)[C@@H]2[C@@H](CC1)C(C2)(C)C | 5281515 |
| 129 | IMPHY014835 | (E)-beta-ocimene | C=C/C(=C/CC=C(C)C)/C | 5281553 |
| 130 | IMPHY014847 | Bornyl acetate | CC(=O)OC1CC2C(C1(C)CC2)(C)C | 6448 |
| 131 | IMPHY014852 | Camphene | C=C1C2CCC(C1(C)C)C2 | 6616 |
| 132 | IMPHY014869 | Isomenthone | C[C@H]1CC[C@H](C(=O)C1)C(C)C | 6432469 |
| 133 | IMPHY014870 | cis-Muurola-4(14),5-diene | C=C1CC[C@@H]2C(=C1)[C@H](CC[C@H]2C)C(C)C | 51351709 |
| 134 | IMPHY014871 | cis-Nerolidol | C=CC(CC/C=C(CCC=C(C)C)/C)(O)C | 5320128 |
| 135 | IMPHY014874 | cis-Sabinene hydrate | C[C@@H]1CC[C@@]2(C1C2)C(C)C | 101629835 |
| 136 | IMPHY014906 | Cedrelanol | CC1=C[C@@H]2[C@@H](CC1)[C@@](C)(O)CC[C@H]2C(C)C | 160799 |
| 137 | IMPHY014986 | Ledol | C[C@@H]1CC[C@H]2[C@@H]1[C@H]1[C@H](C1(C)C)CC[C@@]2(C)O | 92812 |
| 138 | IMPHY014988 | Limonene | CC1=CCC(CC1)C(=C)C | 22311 |
| 139 | IMPHY015004 | Menthone | C[C@@H]1CC[C@H](C(=O)C1)C(C)C | 26447 |
| 140 | IMPHY015042 | Piperitone | CC1=CC(=O)C(CC1)C(C)C | 6987 |
| 141 | IMPHY015123 | alpha-Copaene | CC([C@@H]1CC[C@]2([C@@H]3[C@H]1C2C(=CC3)C)C)C | 70678558 |
| 142 | IMPHY015128 | T-Muurolol | CC1=C[C@@H]2[C@H](CC1)[C@@](C)(O)CC[C@H]2C(C)C | 3084331 |
| 143 | IMPHY016012 | Allo-Aromadendrene | C[C@@H]1CC[C@H]2[C@@H]1C1C(C1(C)C)CCC2=C | 42608158 |
| 144 | IMPHY016014 | Isobornyl acetate | CC(=O)O[C@H]1C[C@@H]2C([C@]1(C)CC2)(C)C | 247573 |
| 145 | IMPHY016053 | Viridiflorol | C[C@@H]1CC[C@H]2[C@@H]1[C@H]1[C@H](C1(C)C)CC[C@]2(C)O | 11996452 |
| 146 | IMPHY016054 | trans-alpha-Bergamotene | CC(=CCC[C@]1(C)[C@H]2CC=C([C@@H]1C2)C)C | 6429302 |
| 147 | IMPHY016314 | 2,4-Dimethylstyrene | C=Cc1ccc(cc1C)C | 16694 |
| 148 | IMPHY016607 | Decahydronaphthalene | C1CCC2C(C1)CCCC2 | 7044 |
| 149 | IMPHY016898 | cis-Dihydrocarvone | CC(=C)[C@H]1CC[C@H](C(=O)C1)C | 443181 |
| 150 | IMPHY016922 | 14-Hydroxi-δ-cadinene | OCC1=CC2C(=C(C)CCC2C(C)C)CC1 |  |
| 151 | IMPHY000099 | Myrtenol | OCC1=CCC2CC1C2(C)C | 10582 |
| 152 | IMPHY001246 | Carvacrol | CC(c1ccc(c(c1)O)C)C | 10364 |
| 153 | IMPHY001828 | 3-Octanol | CCCCCC(CC)O | 11527 |
| 154 | IMPHY003485 | Myrcene | C=CC(=C)CCC=C(C)C | 31253 |
| 155 | IMPHY003536 | Eugenol | C=CCc1ccc(c(c1)OC)O | 3314 |
| 156 | IMPHY003977 | (-)-beta-Bourbonene | CC([C@@H]1CC[C@@]2([C@H]1[C@H]1C(=C)CC[C@@H]21)C)C | 62566 |
| 157 | IMPHY003982 | gamma-Terpinene | CC1=CCC(=CC1)C(C)C | 7461 |
| 158 | IMPHY005400 | Sclareol | C=C[C@@](CC[C@H]1[C@](C)(O)CC[C@@H]2[C@]1(C)CCCC2(C)C)(O)C | 163263 |
| 159 | IMPHY006145 | p-Cymene | Cc1ccc(cc1)C(C)C | 7463 |
| 160 | IMPHY006653 | Carnosic acid | OC(=O)[C@@]12CCCC([C@@H]2CCc2c1c(O)c(c(c2)C(C)C)O)(C)C | 65126 |
| 161 | IMPHY006950 | Tricyclene | CC12C3C1CC(C2(C)C)C3 | 79035 |
| 162 | IMPHY007067 | Linalyl acetate | C=CC(OC(=O)C)(CCC=C(C)C)C | 8294 |
| 163 | IMPHY009771 | Caryophyllene acetate | CC(=O)O[C@]12CCC[C@@](C1)(C)CC[C@@H]1[C@@H]2CC1(C)C | 90473619 |
| 164 | IMPHY010072 | Eucalyptol | CC12CCC(CC1)C(O2)(C)C | 2758 |
| 165 | IMPHY011356 | alpha-Bergamotenol | OC/C(=CCCC1(C)C2CC=C(C1C2)C)/C | 5368743 |
| 166 | IMPHY011396 | 4-Carvomenthenol | CC1=CCC(CC1)(O)C(C)C | 11230 |
| 167 | IMPHY011552 | (1R)-2-methyl-5-propan-2-ylbicyclo[3.1.0]hex-2-ene | CC1=CCC2([C@@H]1C2)C(C)C | 6451618 |
| 168 | IMPHY011568 | alpha-Fenchyl acetate | CC(=O)O[C@@H]1[C@]2(C)CC[C@H](C1(C)C)C2 | 7530950 |
| 169 | IMPHY011570 | (2Z,6E)-Farnesyl acetate | C/C(=CCC/C(=CCOC(=O)C)/C)/CCC=C(C)C | 1551480 |
| 170 | IMPHY011590 | d-Borneol | O[C@@H]1C[C@H]2C([C@@]1(C)CC2)(C)C | 61060 |
| 171 | IMPHY011599 | Terpinolene | CC1=CCC(=C(C)C)CC1 | 11463 |
| 172 | IMPHY011630 | cis,cis-Farnesol | OC/C=C(CC/C=C(CCC=C(C)C)/C)/C | 1549107 |
| 173 | IMPHY011633 | (2Z,6E)-Farnesol | OC/C=C(CC/C=C(/CCC=C(C)C)C)/C | 1549108 |
| 174 | IMPHY011643 | alpha-Terpinene | CC1=CC=C(CC1)C(C)C | 7462 |
| 175 | IMPHY011659 | alpha-Muurolene | CC1=C[C@@H]2[C@H](CC1)C(=CC[C@H]2C(C)C)C | 12306047 |
| 176 | IMPHY011761 | Humulene | C/C/1=CCC(C)(C)/C=C/C/C(=C/CC1)/C | 5281520 |
| 177 | IMPHY011792 | gamma-Muurolene | CC1=C[C@@H]2[C@H](CC1)C(=C)CC[C@H]2C(C)C | 12313020 |
| 178 | IMPHY011793 | (+)-gamma-Cadinene | CC1=C[C@@H]2[C@@H](CC1)C(=C)CC[C@H]2C(C)C | 6432404 |
| 179 | IMPHY011901 | Thujone | O=C1C[C@]2([C@@H]([C@H]1C)C2)C(C)C | 261491 |
| 180 | IMPHY011902 | beta-Thujone | O=C1C[C@]2([C@@H]([C@@H]1C)C2)C(C)C | 91456 |
| 181 | IMPHY011957 | (+)-delta-Cadinene | CC1=C[C@@H]2C(=C(C)CC[C@H]2C(C)C)CC1 | 441005 |
| 182 | IMPHY012036 | Camphor | O=C1CC2C(C1(C)CC2)(C)C | 2537 |
| 183 | IMPHY012058 | Linalool | C=CC(CCC=C(C)C)(O)C | 6549 |
| 184 | IMPHY012061 | alpha-Pinene | CC1=CCC2CC1C2(C)C | 6654 |
| 185 | IMPHY012147 | beta-Pinene | C=C1CCC2CC1C2(C)C | 14896 |
| 186 | IMPHY012165 | Sabinene | C=C1CCC2(C1C2)C(C)C | 18818 |
| 187 | IMPHY012168 | (1S,2S,6S,7R,8R)-1,3-dimethyl-8-propan-2-yltricyclo[4.4.0.02,7]dec-3-ene | CC([C@H]1CC[C@]2([C@@H]3[C@@H]1[C@H]2C(=CC3)C)C)C | 101607926 |
| 188 | IMPHY012667 | Caryophyllene oxide | C=C1CC[C@H]2O[C@@]2(CC[C@@H]2[C@@H]1CC2(C)C)C | 1742210 |
| 189 | IMPHY012739 | (Z)-beta-Ocimene | C=C/C(=CCC=C(C)C)/C | 5320250 |
| 190 | IMPHY012910 | trans-Calamenene | CC([C@H]1CC[C@@H](c2c1cc(C)cc2)C)C | 6429022 |
| 191 | IMPHY013080 | alpha-Calacorene | CC([C@@H]1CC=C(c2c1cc(C)cc2)C)C | 12302243 |
| 192 | IMPHY013836 | Fenchone | O=C1C2(C)CCC(C1(C)C)C2 | 14525 |
| 193 | IMPHY014806 | Caswell No. 264AB | CC([C@@H]1CC[C@H]([C@]23[C@H]1[C@H]2C(=CC3)C)C)C | 442359 |
| 194 | IMPHY014811 | alpha-Phellandrene | CC1=CCC(C=C1)C(C)C | 7460 |
| 195 | IMPHY014817 | Aromadendrene | CC1CCC2C1C1C(C1(C)C)CCC2=C | 91354 |
| 196 | IMPHY014831 | beta-Caryophyllene | C/C/1=CCCC(=C)[C@@H]2[C@@H](CC1)C(C2)(C)C | 5281515 |
| 197 | IMPHY014835 | (E)-beta-ocimene | C=C/C(=C/CC=C(C)C)/C | 5281553 |
| 198 | IMPHY014847 | Bornyl acetate | CC(=O)OC1CC2C(C1(C)CC2)(C)C | 6448 |
| 199 | IMPHY014852 | Camphene | C=C1C2CCC(C1(C)C)C2 | 6616 |
| 200 | IMPHY014907 | 6-Epi-beta-bisabolol | CC(=CCC[C@@H]([C@@]1( | 12300148 |
| 201 | IMPHY014988 | Limonene | CC1=CCC(CC1)C(=C)C | 22311 |
| 202 | IMPHY015040 | 3-Pinanone | O=C1CC2CC(C1C)C2(C)C | 11038 |
| 203 | IMPHY015123 | alpha-Copaene | CC([C@@H]1CC[C@]2([C@@H]3[C@H]1C2C(=CC3)C)C)C | 70678558 |
| 204 | IMPHY015257 | 2-Ethylhexanoic acid | CCCCC(C(=O)O)CC | 8697 |
| 205 | IMPHY016005 | (2E)-2-methyl-6-[(1S)-4-methylcyclohex-3-en-1-yl]hepta-2,6-dien-1-ol | OC/C(=C/CCC(=C)[C@H]1CCC(=CC1)C)/C | 15560069 |
| 206 | IMPHY016012 | Allo-Aromadendrene | C[C@@H]1CC[C@H]2[C@@H]1C1C(C1(C)C)CCC2=C | 42608158 |
| 207 | IMPHY016053 | Viridiflorol | C[C@@H]1CC[C@H]2[C@@H]1[C@H]1[C@H](C1(C)C)CC[C@]2(C)O | 11996452 |
| 208 | IMPHY000060 | Myristic acid | CCCCCCCCCCCCCC(=O)O | 11005 |
| 209 | IMPHY000099 | Myrtenol | OCC1=CCC2CC1C2(C)C | 10582 |
| 210 | IMPHY000173 | Thujyl alcohol | OC1CC2(C(C1C)C2)C(C)C | 10550 |
| 211 | IMPHY000545 | O-Cymene | CC(c1ccccc1C)C | 10703 |
| 212 | IMPHY001135 | 6,10,14-Trimethylpentadecan-2-one | CC(CCCC(C)C)CCCC(CCCC(=O)C)C | 10408 |
| 213 | IMPHY001246 | Carvacrol | CC(c1ccc(c(c1)O)C)C | 10364 |
| 214 | IMPHY001711 | Salvigenin | COc1ccc(cc1)c1cc(=O)c2c(o1)cc(c(c2O)OC)OC | 161271 |
| 215 | IMPHY001828 | 3-Octanol | CCCCCC(CC)O | 11527 |
| 216 | IMPHY002072 | Pinocarvone | C=C1C(=O)CC2CC1C2(C)C | 121719 |
| 217 | IMPHY002825 | 2-(4-Methylphenyl)propan-2-ol | Cc1ccc(cc1)C(O)(C)C | 14529 |
| 218 | IMPHY003016 | Lauric acid | CCCCCCCCCCCC(=O)O | 3893 |
| 219 | IMPHY003301 | Octanoic acid | CCCCCCCC(=O)O | 379 |
| 220 | IMPHY003485 | Myrcene | C=CC(=C)CCC=C(C)C | 31253 |
| 221 | IMPHY003536 | Eugenol | C=CCc1ccc(c(c1)OC)O | 3314 |
| 222 | IMPHY003631 | Valeranone | CC([C@H]1CC[C@@]2([C@@](C1)(C)C(=O)CCC2)C)C | 171455 |
| 223 | IMPHY003710 | (-)-Isopulegol | C[C@@H]1CC[C@H]([C@@H](C1)O)C(=C)C | 170833 |
| 224 | IMPHY003966 | 13-Epi-manool | C=C[C@](CC[C@H]1C(=C)CC[C@@H]2[C@]1(C)CCCC2(C)C)(O)C | 10891602 |
| 225 | IMPHY003977 | (-)-beta-Bourbonene | CC([C@@H]1CC[C@@]2([C@H]1[C@H]1C(=C)CC[C@@H]21)C)C | 62566 |
| 226 | IMPHY003982 | gamma-Terpinene | CC1=CCC(=CC1)C(C)C | 7461 |
| 227 | IMPHY004003 | Neoisopulegol | C[C@@H]1CC[C@H]([C@H](C1)O)C(=C)C | 6553885 |
| 228 | IMPHY004017 | Manool | C=C[C@@](CC[C@H]1C(=C)CC[C@@H]2[C@]1(C)CCCC2(C)C)(O)C | 3034394 |
| 229 | IMPHY004077 | Verbenone | CC1=CC(=O)C2CC1C2(C)C | 29025 |
| 230 | IMPHY004194 | (1S,2R,5S)-2-isopropyl-5-methylcyclohexyl acetate | C[C@H]1CC[C@@H]([C@H](C1)OC(=O)C)C(C)C | 62335 |
| 231 | IMPHY004597 | Rosmarinic acid | O=C(O[C@@H](C(=O)O)Cc1ccc(c(c1)O)O)/C=C/c1ccc(c(c1)O)O | 5281792 |
| 232 | IMPHY004660 | Luteolin | Oc1cc(O)c2c(c1)oc(cc2=O)c1ccc(c(c1)O)O | 5280445 |
| 233 | IMPHY004877 | Nepetin | COc1c(O)cc2c(c1O)c(=O)cc(o2)c1ccc(c(c1)O)O | 5317284 |
| 234 | IMPHY005327 | Cirsimaritin | COc1cc2oc(cc(=O)c2c(c1OC)O)c1ccc(cc1)O | 188323 |
| 235 | IMPHY005345 | 1-Octen-3-OL | CCCCCC(C=C)O | 18827 |
| 236 | IMPHY005390 | 1-Octen-3-yl acetate | CCCCCC(OC(=O)C)C=C | 17121 |
| 237 | IMPHY005400 | Sclareol | C=C[C@@](CC[C@H]1[C@](C)(O)CC[C@@H]2[C@]1(C)CCCC2(C)C)(O)C | 163263 |
| 238 | IMPHY005435 | Genkwanin | COc1cc(O)c2c(c1)oc(cc2=O)c1ccc(cc1)O | 5281617 |
| 239 | IMPHY005442 | Hispidulin | COc1c(O)cc2c(c1O)c(=O)cc(o2)c1ccc(cc1)O | 5281628 |
| 240 | IMPHY005940 | Thunbergol | C/C/1=C/CC/C(=C/CCC(/C=C/C(CC1)C(C)C)(C)O)/C | 5363523 |
| 241 | IMPHY006145 | p-Cymene | Cc1ccc(cc1)C(C)C | 7463 |
| 242 | IMPHY006325 | Myrtenal | O=CC1=CCC2CC1C2(C)C | 61130 |
| 243 | IMPHY006550 | Thymol | Cc1ccc(c(c1)O)C(C)C | 6989 |
| 244 | IMPHY006612 | Corosolic acid | C[C@@H]1CC[C@]2([C@@H]([C@H]1C)C1=CC[C@H]3[C@@]([C@@]1(CC2)C)(C)CC[C@@H]1[C@]3(C)C[C@@H](O)[C@@H](C1(C)C)O)C(=O)O | 6918774 |
| 245 | IMPHY006696 | Methyleugenol | C=CCc1ccc(c(c1)OC)OC | 7127 |
| 246 | IMPHY006944 | Estragole | COc1ccc(cc1)CC=C | 8815 |
| 247 | IMPHY006950 | Tricyclene | CC12C3C1CC(C2(C)C)C3 | 79035 |
| 248 | IMPHY007067 | Linalyl acetate | C=CC(OC(=O)C)(CCC=C(C)C)C | 8294 |
| 249 | IMPHY007317 | 4-Allylphenol | C=CCc1ccc(cc1)O | 68148 |
| 250 | IMPHY007327 | Palmitic acid | CCCCCCCCCCCCCCCC(=O)O | 985 |
| 251 | IMPHY007376 | beta-Cubebene | CC([C@@H]1CC[C@H]([C@]23[C@H]1[C@H]2C(=C)CC3)C)C | 93081 |
| 252 | IMPHY007421 | Citronellyl acetate | CC(CCC=C(C)C)CCOC(=O)C | 9017 |
| 253 | IMPHY007520 | Viridiflorene | C[C@@H]1CCC2=C(C)CC[C@@H]3[C@H]([C@H]12)C3(C)C | 10910653 |
| 254 | IMPHY007840 | Spathulenol | C=C1CC[C@@H]2[C@H]([C@H]3[C@H]1CC[C@]3(C)O)C2(C)C | 92231 |
| 255 | IMPHY008024 | Isobutyl isobutyrat | CC(COC(=O)C(C)C)C | 7351 |
| 256 | IMPHY008150 | 1-Methyl-4-(prop-1-en-2-yl)benzene | Cc1ccc(cc1)C(=C)C | 62385 |
| 257 | IMPHY009688 | 3,7(11)-Eudesmadiene | CC1=CCCC2(C1CC(=C(C)C)CC2)C | 522296 |
| 258 | IMPHY009771 | Caryophyllene acetate | CC(=O)O[C@]12CCC[C@@](C1)(C)CC[C@@H]1[C@@H]2CC1(C)C | 90473619 |
| 259 | IMPHY009832 | Lavandulyl acetate | CC(=O)OCC(C(=C)C)CC=C(C)C | 30247 |
| 260 | IMPHY009874 | Myrtenyl acetate | CC(=O)OCC1=CCC2CC1C2(C)C | 61262 |
| 261 | IMPHY010072 | Eucalyptol | CC12CCC(CC1)C(O2)(C)C | 2758 |
| 262 | IMPHY010097 | Benzyl benzoate | O=C(c1ccccc1)OCc1ccccc1 | 2345 |
| 263 | IMPHY011239 | Carnosol | O=C1O[C@H]2C[C@@H]3[C@]1(CCCC3(C)C)c1c2cc(c(c1O)O)C(C)C | 442009 |
| 264 | IMPHY011356 | alpha-Bergamotenol | OC/C(=CCCC1(C)C2CC=C(C1C2)C)/C | 5368743 |
| 265 | IMPHY011392 | 3-Carene | CC1=CCC2C(C1)C2(C)C | 26049 |
| 266 | IMPHY011396 | 4-Carvomenthenol | CC1=CCC(CC1)(O)C(C)C | 11230 |
| 267 | IMPHY011455 | Cadina-1,4-diene | CC1=CC2C(=CC1)[C@@H](C)CCC2C(C)C | 6427091 |
| 268 | IMPHY011519 | alpha-Terpinyl acetate | CC(=O)OC(C1CCC(=CC1)C)(C)C | 111037 |
| 269 | IMPHY011552 | (1R)-2-methyl-5-propan-2-ylbicyclo[3.1.0]hex-2-ene | CC1=CCC2([C@@H]1C2)C(C)C | 6451618 |
| 270 | IMPHY011568 | alpha-Fenchyl acetate | CC(=O)O[C@@H]1[C@]2(C)CC[C@H](C1(C)C)C2 | 7530950 |
| 271 | IMPHY011570 | (2Z,6E)-Farnesyl acetate | C/C(=CCC/C(=CCOC(=O)C)/C)/CCC=C(C)C | 1551480 |
| 272 | IMPHY011581 | alpha-Selinene | CC1=CCC[C@]2([C@H]1C[C@@H](CC2)C(=C)C)C | 10856614 |
| 273 | IMPHY011586 | (S,1Z,6Z)-8-Isopropyl-1-methyl-5-methylenecyclodeca-1,6-diene | C/C/1=C/CCC(=C)/C=C[C@@H](CC1)C(C)C | 91723653 |
| 274 | IMPHY011590 | d-Borneol | O[C@@H]1C[C@H]2C([C@@]1(C)CC2)(C)C | 61060 |
| 275 | IMPHY011599 | Terpinolene | CC1=CCC(=C(C)C)CC1 | 11463 |
| 276 | IMPHY011630 | cis,cis-Farnesol | OC/C=C(CC/C=C(CCC=C(C)C)/C)/C | 1549107 |
| 277 | IMPHY011633 | (2Z,6E)-Farnesol | OC/C=C(CC/C=C(/CCC=C(C)C)C)/C | 1549108 |
| 278 | IMPHY011643 | alpha-Terpinene | CC1=CC=C(CC1)C(C)C | 7462 |
| 279 | IMPHY011646 | Cynaroside | OC[C@H]1O[C@@H](Oc2cc(O)c3c(c2)oc(cc3=O)c2ccc(c(c2)O)O)[C@@H]([C@H]([C@@H]1O)O)O | 5280637 |
| 280 | IMPHY011647 | Geranyl acetate | C/C(=CCOC(=O)C)/CCC=C(C)C | 1549026 |
| 281 | IMPHY011648 | Neryl acetate | C/C(=C/COC(=O)C)/CCC=C(C)C | 1549025 |
| 282 | IMPHY011658 | beta-Farnesene | C=CC(=C)CC/C=C(/CCC=C(C)C)C | 5281517 |
| 283 | IMPHY011659 | alpha-Muurolene | CC1=C[C@@H]2[C@H](CC1)C(=CC[C@H]2C(C)C)C | 12306047 |
| 284 | IMPHY011660 | (+)-alpha-Cadinene | CC1=C[C@@H]2[C@@H](CC1)C(=CC[C@H]2C(C)C)C | 12306048 |
| 285 | IMPHY011761 | Humulene | C/C/1=CCC(C)(C)/C=C/C/C(=C/CC1)/C | 5281520 |
| 286 | IMPHY011792 | gamma-Muurolene | CC1=C[C@@H]2[C@H](CC1)C(=C)CC[C@H]2C(C)C | 12313020 |
| 287 | IMPHY011793 | (+)-gamma-Cadinene | CC1=C[C@@H]2[C@@H](CC1)C(=C)CC[C@H]2C(C)C | 6432404 |
| 288 | IMPHY011826 | Oleanolic acid | O[C@H]1CC[C@]2([C@H](C1(C)C)CC[C@@]1([C@@H]2CC=C2[C@@]1(C)CC[C@@]1([C@H]2CC(C)(C)CC1)C(=O)O)C)C | 10494 |
| 289 | IMPHY011872 | (4E,7E)-1,5,9,9-Tetramethyl-12-oxabicyclo[9.1.0]dodeca-4,7-diene | C/C/1=C/CCC2(C)OC2CC(/C=CC1)(C)C | 22559443 |
| 290 | IMPHY011880 | Ursolic acid | C[C@@H]1CC[C@]2([C@@H]([C@H]1C)C1=CC[C@H]3[C@@]([C@@]1(CC2)C)(C)CC[C@@H]1[C@]3(C)CC[C@@H](C1(C)C)O)C(=O)O | 64945 |
| 291 | IMPHY011884 | Pulegone | C[C@@H]1CCC(=C(C)C)C(=O)C1 | 442495 |
| 292 | IMPHY011901 | Thujone | O=C1C[C@]2([C@@H]([C@H]1C)C2)C(C)C | 261491 |
| 293 | IMPHY011902 | beta-Thujone | O=C1C[C@]2([C@@H]([C@@H]1C)C2)C(C)C | 91456 |
| 294 | IMPHY011933 | Caffeic acid | OC(=O)/C=C/c1ccc(c(c1)O)O | 689043 |
| 295 | IMPHY011957 | (+)-delta-Cadinene | CC1=C[C@@H]2C(=C(C)CC[C@H]2C(C)C)CC1 | 441005 |
| 296 | IMPHY011965 | (+)-beta-Phellandrene | CC([C@@H]1CCC(=C)C=C1)C | 442484 |
| 297 | IMPHY011988 | (-)-trans-Carveol | CC(=C)[C@@H]1CC=C([C@H](C1)O)C | 94221 |
| 298 | IMPHY012036 | Camphor | O=C1CC2C(C1(C)CC2)(C)C | 2537 |
| 299 | IMPHY012053 | Sucrose | OC[C@H]1O[C@@]([C@H]([C@@H]1O)O)(CO)O[C@H]1O[C@H](CO)[C@H]([C@@H]([C@H]1O)O)O | 5988 |
| 300 | IMPHY012056 | (-)-Bornyl acetate | CC(=O)O[C@@H]1C[C@H]2C([C@]1(C)CC2)(C)C | 93009 |
| 301 | IMPHY011884 | Pulegone | C[C@@H]1CCC(=C(C)C)C(=O)C1 | 442495 |
| 302 | IMPHY011901 | Thujone | O=C1C[C@]2([C@@H]([C@H]1C)C2)C(C)C | 261491 |
| 303 | IMPHY011902 | beta-Thujone | O=C1C[C@]2([C@@H]([C@@H]1C)C2)C(C)C | 91456 |
| 304 | IMPHY011933 | Caffeic acid | OC(=O)/C=C/c1ccc(c(c1)O)O | 689043 |
| 305 | IMPHY011957 | (+)-delta-Cadinene | CC1=C[C@@H]2C(=C(C)CC[C@H]2C(C)C)CC1 | 441005 |
| 306 | IMPHY011965 | (+)-beta-Phellandrene | CC([C@@H]1CCC(=C)C=C1)C | 442484 |
| 307 | IMPHY011988 | (-)-trans-Carveol | CC(=C)[C@@H]1CC=C([C@H](C1)O)C | 94221 |
| 308 | IMPHY012036 | Camphor | O=C1CC2C(C1(C)CC2)(C)C | 2537 |
| 309 | IMPHY012053 | Sucrose | OC[C@H]1O[C@@]([C@H]([C@@H]1O)O)(CO)O[C@H]1O[C@H](CO)[C@H]([C@@H]([C@H]1O)O)O | 598 |
| 310 | IMPHY012056 | (-)-Bornyl acetate | CC(=O)O[C@@H]1C[C@H]2C([C@]1(C)CC2)(C)C | 93009 |
| 311 | IMPHY012585 | delta-Cadinol | CC1=C[C@@H]2[C@H](CC1)[C@](C)(O)CC[C@H]2C(C)C | 3084311 |
| 312 | IMPHY012586 | (-)-alpha-Cadinol | CC1=CC2C(CC1)[C@@](C)(O)CC[C@@H]2C(C)C | 6431302 |
| 313 | IMPHY012667 | Caryophyllene oxide | C=C1CC[C@H]2O[C@@]2(CC[C@@H]2[C@@H]1CC2(C)C)C | 1742210 |
| 314 | IMPHY012712 | Phytol | OC/C=C(/CCC[C@@H](CCC[C@@H](CCCC(C)C)C)C)C | 5280435 |
| 315 | IMPHY012719 | Cosmosiin | OC[C@H]1O[C@@H](Oc2cc(O)c3c(c2)oc(cc3=O)c2ccc(cc2)O)[C@@H]([C@H]([C@@H]1O)O)O | 5280704 |
| 316 | IMPHY012737 | (1S,4E,9S)-4,11,11-trimethyl-8-methylidenebicyclo[7.2.0]undec-4-ene | C/C/1=CCCC(=C)[C@@H]2[C@H](CC1)C(C2)(C)C | 6429301 |
| 317 | IMPHY012738 | Isocaryophyllene | C/C/1=C/CCC(=C)[C@@H]2[C@@H](CC1)C(C2)(C)C | 5281522 |
| 318 | IMPHY012739 | (Z)-beta-Ocimene | C=C/C(=CCC=C(C)C)/C | 5320250 |
| 319 | IMPHY012910 | trans-Calamenene | CC([C@H]1CC[C@@H](c2c1cc(C)cc2)C)C | 6429022 |
| 320 | IMPHY013080 | alpha-Calacorene | CC([C@@H]1CC=C(c2c1cc(C)cc2)C)C | 12302243 |
| 321 | IMPHY013836 | Fenchone | O=C1C2(C)CCC(C1(C)C)C2 | 14525 |
| 322 | IMPHY014115 | 2-Isopropyl-1,4-hexadiene | C/C=C/CC(=C)C(C)C | 6429324 |
| 323 | IMPHY014690 | (-)-Globulol | C[C@@H]1CC[C@@H]2[C@@H]1[C@H]1[C@H](C1(C)C)CC[C@@]2(C)O | 12304985 |
| 324 | IMPHY014708 | beta-Selinene | C=C1CCC[C@]2([C@H]1C[C@@H](CC2)C(=C)C)C | 442393 |
| 325 | IMPHY014801 | Zizanene | CC1=C[C@@H]2[C@H](CC1)C(=CC[C@@H]2C(C)C)C | 12306046 |
| 326 | IMPHY014805 | Cedr-8-ene | CC1=CC[C@@]23C[C@@H]1C(C)(C)[C@@H]2CC[C@H]3C | 6431015 |
| 327 | IMPHY014806 | Caswell No. 264AB | CC([C@@H]1CC[C@H]([C@]23[C@H]1[C@H]2C(=CC3)C)C)C | 442359 |
| 328 | IMPHY014811 | alpha-Phellandrene | CC1=CCC(C=C1)C(C)C | 7460 |
| 329 | IMPHY014817 | Aromadendrene | CC1CCC2C1C1C(C1(C)C)CCC2=C | 91354 |
| 330 | IMPHY014831 | beta-Caryophyllene | C/C/1=CCCC(=C)[C@@H]2[C@@H](CC1)C(C2)(C)C | 5281515 |
| 331 | IMPHY014835 | (E)-beta-ocimene | C=C/C(=C/CC=C(C)C)/C | 5281553 |
| 332 | IMPHY014836 | beta-Sitosterol | CC[C@@H](C(C)C)CC[C@H]([C@H]1CC[C@@H]2[C@]1(C)CC[C@H]1[C@H]2CC=C2[C@]1(C)CC[C@@H](C2)O)C | 222284 |
| 333 | IMPHY014847 | Bornyl acetate | CC(=O)OC1CC2C(C1(C)CC2)(C)C | 6448 |
| 334 | IMPHY014852 | Camphene | C=C1C2CCC(C1(C)C)C2 | 6616 |
| 335 | IMPHY014874 | cis-Sabinene hydrate | C[C@@H]1CC[C@@]2(C1C2)C(C)C | 101629835 |
| 336 | IMPHY014907 | 6-Epi-beta-bisabo | CC(=CCC[C@@H]([C@@]1(O)CCC(=CC1)C)C)C | 12300148 |
| 337 | IMPHY014923 | Geraniol | OC/C=C(/CCC=C(C)C)C | 637566 |
| 338 | IMPHY014986 | Ledol | C[C@@H]1CC[C@H]2[C@@H]1[C@H]1[C@H](C1(C)C)CC[C@@]2(C)O | 92812 |
| 339 | IMPHY014988 | Limonene | CC1=CCC(CC1)C(=C)C | 22311 |
| 340 | IMPHY015003 | Menthol | CC1CCC(C(C1)O)C(C)C | 1254 |
| 341 | IMPHY015004 | Menthone | C[C@@H]1CC[C@H](C(=O)C1)C(C)C | 26447 |
| 342 | IMPHY015040 | 3-Pinanone | O=C1CC2CC(C1C)C2(C)C | 11038 |
| 343 | IMPHY015042 | Piperitone | CC1=CC(=O)C(CC1)C(C)C | 6987 |
| 344 | IMPHY015063 | Sabinyl acetate | CC(=O)OC1CC2(C(C1=C)C2)C(C)C | 94266 |
| 345 | IMPHY015123 | alpha-Copaene | CC([C@@H]1CC[C@]2([C@@H]3[C@H]1C2C(=CC3)C)C)C | 70678558 |
| 346 | IMPHY015257 | 2-Ethylhexanoic acid | CCCCC(C(=O)O)CC | 8697 |
| 347 | IMPHY016005 | (2E)-2-methyl-6-[(1S)-4-methylcyclohex-3-en-1-yl]hepta-2,6-dien-1-ol | OC/C(=C/CCC(=C)[C@H]1CCC(=CC1)C)/C | 15560069 |
| 348 | IMPHY016012 | Allo-Aromadendrene | C[C@@H]1CC[C@H]2[C@@H]1C1C(C1(C)C)CCC2=C | 42608158 |
| 349 | IMPHY016027 | trans-Sabinene hydrate | CC([C@@]12CC[C@](C2C1)(C)O)C | 12315151 |
| 350 | IMPHY016050 | Umbellulol | CC1=CC(C2(C1C2)C(C)C)O | 561871 |
| 351 | IMPHY016053 | Viridiflorol | C[C@@H]1CC[C@H]2[C@@H]1[C@H]1[C@H](C1(C)C)CC[C@]2(C)O | 11996452 |
| 352 | IMPHY017147 | Cedrol formate | O=CO[C@]1(C)CC[C@@]23C[C@@H]1C(C)(C)[C@@H]2CC[C@H]3C | 162002 |
| 353 | IMPHY017650 | 1,2-Epoxyhumulene | CC1CCCC2(C)OC2CC(/C=C/C1)(C)C | 6432846 |
| 354 | IMPHY001086 | Royleanone | O=C1C(=O)C(=C(C2=C1[C@@]1(C)CCCC([C@@H]1CC2)(C)C)O)C(C)C | 442084 |
| 355 | IMPHY004608 | Salvianolic acid A | O=C(O[C@@H](C(=O)O)Cc1ccc(c(c1)O)O)/C=C/c1ccc(c(c1/C=C/c1ccc(c(c1)O)O)O)O | 5281793 |
| 356 | IMPHY006793 | Lithospermic acid | O=C(O[C@@H](C(=O)O)Cc1ccc(c(c1)O)O)/C=C/c1ccc(c2c1[C@H](C(=O)O)[C@H](O2)c1ccc(c(c1)O)O)O | 6441498 |
| 357 | IMPHY011901 | Thujone | O=C1C[C@]2([C@@H]([C@H]1C)C2)C(C)C | 261491 |
| 358 | IMPHY011902 | beta-Thujone | O=C1C[C@]2([C@@H]([C@@H]1C)C2)C(C)C | 91456 |
| 359 | IMPHY012036 | Camphor | O=C1CC2C(C1(C)CC2)(C)C | 2537 |
| 360 | IMPHY014776 | 7alpha-Acetoxyroyleanone | CC(=O)O[C@@H]1C[C@H]2C(C)(C)CCC[C@@]2(C2=C1C(=C(C(C)C)C(=O)C2=O)O)C | 2751796 |
| 361 | IMPHY017250 | 6,7-Dehydroroyleanone | O=C1C(=O)C(=C(C2=C1[C@@]1(C)CCCC([C@@H]1C=C2)(C)C)O)C(C)C | 2751794 |
| 362 | IMPHY011619 | alpha-Amyrin | C[C@@H]1CC[C@]2([C@@H]([C@H]1C)C1=CC[C@H]3[C@@]([C@@]1(CC2)C)(C)CC[C@@H]1[C@]3(C)CC[C@@H](C1(C)C)O)C | 73170 |
| 363 | IMPHY012061 | alpha-Pinene | CC1=CCC2CC1C2(C)C | 6654 |
| 364 | IMPHY012223 | beta-Amyrin | O[C@H]1CC[C@]2([C@H](C1(C)C)CC[C@@]1([C@@H]2CC=C2[C@@]1(C)CC[C@@]1([C@H]2CC(C)(C)CC1)C)C)C | 73145 |
| 365 | IMPHY014836 | beta-Sitosterol | CC[C@@H](C(C)C)CC[C@H]([C@H]1CC[C@@H]2[C@]1(C)CC[C@H]1[C@H]2CC=C2[C@]1(C)CC[C@@H](C2)O)C | 222284 |
| 366 | IMPHY000099 | Myrtenol | OCC1=CCC2CC1C2(C)C | 10582 |
| 367 | IMPHY001246 | Carvacrol | CC(c1ccc(c(c1)O)C)C | 10364 |
| 368 | IMPHY001828 | 3-Octanol | CCCCCC(CC)O | 11527 |
| 369 | IMPHY003485 | Myrcene | C=CC(=C)CCC=C(C)C | 31253 |
| 370 | IMPHY003536 | Eugenol | C=CCc1ccc(c(c1)OC)O | 3314 |
| 371 | IMPHY003977 | (-)-beta-Bourbonene | CC([C@@H]1CC[C@@]2([C@H]1[C@H]1C(=C)CC[C@@H]21)C)C | 62566 |
| 372 | IMPHY003982 | gamma-Terpinene | CC1=CCC(=CC1)C(C)C | 7461 |
| 373 | IMPHY005400 | Sclareol | C=C[C@@](CC[C@H]1[C@](C)(O)CC[C@@H]2[C@]1(C)CCCC2(C)C)(O)C | 163263 |
| 374 | IMPHY006145 | p-Cymene | Cc1ccc(cc1)C(C)C | 7463 |
| 375 | IMPHY006950 | Tricyclene | CC12C3C1CC(C2(C)C)C3 | 79035 |
| 376 | IMPHY007067 | Linalyl acetate | C=CC(OC(=O)C)(CCC=C(C)C)C | 8294 |
| 377 | IMPHY009771 | Caryophyllene acetate | CC(=O)O[C@]12CCC[C@@](C1)(C)CC[C@@H]1[C@@H]2CC1(C)C | 90473619 |
| 378 | IMPHY010072 | Eucalyptol | CC12CCC(CC1)C(O2)(C)C | 2758 |
| 379 | IMPHY011356 | alpha-Bergamotenol | OC/C(=CCCC1(C)C2CC=C(C1C2)C)/C | 5368743 |
| 380 | IMPHY011396 | 4-Carvomenthenol | CC1=CCC(CC1)(O)C(C)C | 11230 |
| 381 | IMPHY011552 | (1R)-2-methyl-5-propan-2-ylbicyclo[3.1.0]hex-2-ene | CC1=CCC2([C@@H]1C2)C(C)C | 6451618 |
| 382 | IMPHY011568 | alpha-Fenchyl acetate | CC(=O)O[C@@H]1[C@]2(C)CC[C@H](C1(C)C)C2 | 7530950 |
| 383 | IMPHY011570 | (2Z,6E)-Farnesyl acetate | C/C(=CCC/C(=CCOC(=O)C)/C)/CCC=C(C)C | 1551480 |
| 384 | IMPHY011590 | d-Borneol | O[C@@H]1C[C@H]2C([C@@]1(C)CC2)(C)C | 61060 |
| 385 | IMPHY011599 | Terpinolene | CC1=CCC(=C(C)C)CC1 | 11463 |
| 386 | IMPHY011630 | cis,cis-Farnesol | OC/C=C(CC/C=C(CCC=C(C)C)/C)/C | 1549107 |
| 387 | IMPHY011633 | (2Z,6E)-Farnesol | OC/C=C(CC/C=C(/CCC=C(C)C)C)/C | 1549108 |
| 388 | IMPHY011643 | alpha-Terpinene | CC1=CC=C(CC1)C(C)C | 7462 |
| 389 | IMPHY011659 | alpha-Muurolene | CC1=C[C@@H]2[C@H](CC1)C(=CC[C@H]2C(C)C)C | 12306047 |
| 390 | IMPHY011761 | Humulene | C/C/1=CCC(C)(C)/C=C/C/C(=C/CC1)/C | 18818 |
| 391 | IMPHY011792 | gamma-Muurolene | CC1=C[C@@H]2[C@H](CC1)C(=C)CC[C@H]2C(C)C | 12313020 |
| 392 | IMPHY011793 | (+)-gamma-Cadinene | CC1=C[C@@H]2[C@@H](CC1)C(=C)CC[C@H]2C(C)C | 6432404 |
| 393 | IMPHY011901 | Thujone | O=C1C[C@]2([C@@H]([C@H]1C)C2)C(C)C | 261491 |
| 394 | IMPHY011902 | beta-Thujone | O=C1C[C@]2([C@@H]([C@@H]1C)C2)C(C)C | 91456 |
| 395 | IMPHY011957 | (+)-delta-Cadinene | CC1=C[C@@H]2C(=C(C)CC[C@H]2C(C)C)CC1 | 441005 |
| 396 | IMPHY012036 | Camphor | O=C1CC2C(C1(C)CC2)(C)C | 2537 |
| 397 | IMPHY012058 | Linalool | C=CC(CCC=C(C)C)(O)C | 6549 |
| 398 | IMPHY012061 | alpha-Pinene | CC1=CCC2CC1C2(C)C | 6654 |
| 399 | IMPHY012147 | beta-Pinene | C=C1CCC2CC1C2(C)C | 14896 |
| 400 | IMPHY012165 | Sabinene | C=C1CCC2(C1C2)C(C)C | 18818 |
| 401 | IMPHY012168 | (1S,2S,6S,7R,8R)-1,3-dimethyl-8-propan-2-yltricyclo[4.4.0.02,7]dec-3-ene | CC([C@H]1CC[C@]2([C@@H]3[C@@H]1[C@H]2C(=CC3)C)C)C | 101607926 |
| 402 | IMPHY012667 | Caryophyllene oxide | C=C1CC[C@H]2O[C@@]2(CC[C@@H]2[C@@H]1CC2(C)C)C | 1742210 |
| 403 | IMPHY012739 | (Z)-beta-Ocimene | C=C/C(=CCC=C(C)C)/C | 5320250 |
| 404 | IMPHY012910 | trans-Calamenene | CC([C@H]1CC[C@@H](c2c1cc(C)cc2)C)C | 6429022 |
| 405 | IMPHY013080 | alpha-Calacorene | CC([C@@H]1CC=C(c2c1cc(C)cc2)C)C | 12302243 |
| 406 | IMPHY013836 | Fenchone | O=C1C2(C)CCC(C1(C)C)C2 | 14525 |
| 407 | IMPHY014806 | Caswell No. 264AB | CC([C@@H]1CC[C@H]([C@]23[C@H]1[C@H]2C(=CC3)C)C)C | 442359 |
| 408 | MPHY014811 | alpha-Phellandrene | CC1=CCC(C=C1)C(C)C | 7460 |
| 409 | IMPHY014817 | Aromadendrene | CC1CCC2C1C1C(C1(C)C)CCC2=C | 91354 |
| 410 | IMPHY014831 | beta-Caryophyllene | C/C/1=CCCC(=C)[C@@H]2[C@@H](CC1)C(C2)(C)C | 5281515 |
| 411 | IMPHY014835 | (E)-beta-ocimene | C=C/C(=C/CC=C(C)C)/C | 5281553 |
| 412 | IMPHY014847 | Bornyl acetate | CC(=O)OC1CC2C(C1(C)CC2)(C)C | 6448 |
| 413 | IMPHY014852 | Camphene | C=C1C2CCC(C1(C)C)C2 | 6616 |
| 414 | IMPHY014907 | 6-Epi-beta-bisabolol | CC(=CCC[C@@H]([C@@]1(O)CCC(=CC1)C)C)C | 12300148 |
| 415 | IMPHY014988 | Limonene | CC1=CCC(CC1)C(=C)C | 22311 |
| 416 | IMPHY015040 | 3-Pinanone | O=C1CC2CC(C1C)C2(C)C | 11038 |
| 417 | IMPHY015123 | alpha-Copaene | CC([C@@H]1CC[C@]2([C@@H]3[C@H]1C2C(=CC3)C)C)C | 70678558 |
| 418 | IMPHY015257 | 2-Ethylhexanoic acid | CCCCC(C(=O)O)CC | 8697 |
| 419 | IMPHY016005 | (2E)-2-methyl-6-[(1S)-4-methylcyclohex-3-en-1-yl]hepta-2,6-dien-1-ol | OC/C(=C/CCC(=C)[C@H]1CCC(=CC1)C)/C | 15560069 |
| 420 | IMPHY016012 | Allo-Aromadendrene | C[C@@H]1CC[C@H]2[C@@H]1C1C(C1(C)C)CCC2=C | 42608158 |
| 421 | IMPHY016053 | Viridiflorol | C[C@@H]1CC[C@H]2[C@@H]1[C@H]1[C@H](C1(C)C)CC[C@]2(C)O | 11996452 |
| 422 | IMPHY000099 | Myrtenol | OCC1=CCC2CC1C2(C)C | 10582 |
| 423 | IMPHY002179 | Humuladienone | C/C/1=C/CC(C)(C)/C=CCC(C(=O)CC1)C | 101297706 |
| 424 | IMPHY003485 | Myrcene | C=CC(=C)CCC=C(C)C | 31253 |
| 425 | IMPHY003536 | Eugenol | C=CCc1ccc(c(c1)OC)O | 3314 |
| 426 | IMPHY003982 | IMPHY003982 | CC1=CCC(=CC1)C(C)C | 7461 |
| 427 | IMPHY004017 | Manool | C=C[C@@](CC[C@H]1C(=C)CC[C@@H]2[C@]1(C)CCCC2(C)C)(O)C | 3034394 |
| 428 | IMPHY004077 | Verbenone | CC1=CC(=O)C2CC1C2(C)C | 29025 |
| 429 | IMPHY005345 | 1-Octen-3-OL | CCCCCC(C=C)O | 18827 |
| 430 | IMPHY006145 | p-Cymene | Cc1ccc(cc1)C(C)C | 7463 |
| 431 | IMPHY006950 | Tricyclene | CC12C3C1CC(C2(C)C)C3 | 79035 |
| 432 | IMPHY007067 | Linalyl acetate | C=CC(OC(=O)C)(CCC=C(C)C)C | 8294 |
| 433 | IMPHY007376 | beta-Cubebene | CC([C@@H]1CC[C@H]([C@]23[C@H]1[C@H]2C(=C)CC3)C)C | 93081 |
| 434 | IMPHY007520 | Viridiflorene | C[C@@H]1CCC2=C(C)CC[C@@H]3[C@H]([C@H]12)C3(C)C | 10910653 |
| 435 | IMPHY007840 | Spathulenol | C=C1CC[C@@H]2[C@H]([C@H]3[C@H]1CC[C@]3(C)O)C2(C)C | 92231 |
| 436 | IMPHY010072 | Eucalyptol | CC12CCC(CC1)C(O2)(C)C | 2758 |
| 437 | IMPHY011049 | Abieta-7,13-diene | CC(C1=CC2=CC[C@@H]3[C@]([C@H]2CC1)(C)CCCC3(C)C)C | 443470 |
| 438 | IMPHY011392 | 3-Carene | CC1=CCC2C(C1)C2(C)C | 26049 |
| 439 | IMPHY011396 | 4-Carvomenthenol | CC1=CCC(CC1)(O)C(C)C | 11230 |
| 440 | IMPHY011519 | alpha-Terpinyl acetate | CC(=O)OC(C1CCC(=CC1)C)(C)C | 111037 |
| 441 | IMPHY011542 | beta-Eudesmol | C=C1CCC[C@]2([C@H]1C[C@@H](CC2)C(O)(C)C)C | 91457 |
| 442 | IMPHY011552 | (1R)-2-methyl-5-propan-2-ylbicyclo[3.1.0]hex-2-ene | CC1=CCC2([C@@H]1C2)C(C)C | 6451618 |
| 443 | IMPHY011586 | (S,1Z,6Z)-8-Isopropyl-1-methyl-5-methylenecyclodeca-1,6-diene | C/C/1=C/CCC(=C)/C=C[C@@H](CC1)C(C)C | 91723653 |
| 444 | IMPHY011590 | d-Borneol | O[C@@H]1C[C@H]2C([C@@]1(C)CC2)(C)C | 61060 |
| 445 | IMPHY011599 | Terpinolene | CC1=CCC(=C(C)C)CC1 | 11463 |
| 446 | IMPHY011643 | alpha-Terpinene | CC1=CC=C(CC1)C(C)C | 7462 |
| 447 | IMPHY011647 | Geranyl acetate | C/C(=CCOC(=O)C)/CCC=C(C)C | 1549026 |
| 448 | IMPHY011659 | alpha-Muurolene | CC1=C[C@@H]2[C@H](CC1)C(=CC[C@H]2C(C)C)C | 12306047 |
| 449 | IMPHY011709 | alpha-Eudesmol | CC1=CCC[C@]2([C@H]1C[C@@H](CC2)C(O)(C)C)C | 92762 |
| 450 | IMPHY011761 | Humulene | C/C/1=CCC(C)(C)/C=C/C/C(=C/CC1)/C | 5281520 |
| 451 | IMPHY011790 | Neral | O=C/C=C(CCC=C(C)C)/C | 643779 |
| 452 | IMPHY011792 | gamma-Muurolene | CC1=C[C@@H]2[C@H](CC1)C(=C)CC[C@H]2C(C)C | 12313020 |
| 453 | IMPHY011826 | Oleanolic acid | O[C@H]1CC[C@]2([C@H](C1(C)C)CC[C@@]1([C@@H]2CC=C2[C@@]1(C)CC[C@@]1([C@H]2CC(C)(C)CC1)C(=O)O)C)C | 10494 |
| 454 | IMPHY011872 | (4E,7E)-1,5,9,9-Tetramethyl-12-oxabicyclo[9.1.0]dodeca-4,7-diene | C/C/1=C/CCC2(C)OC2CC(/C=CC1)(C)C | 22559443 |
| 455 | IMPHY011873 | Humulene epoxide | C/C/1=CCCC2(C)OC2CC(/C=C/C1)(C)C | 5352470 |
| 456 | IMPHY011890 | Elemol | C=C[C@]1(C)CC[C@H](C[C@H]1C(=C)C)C(O)(C)C | 92138 |
| 457 | IMPHY011901 | Thujone | O=C1C[C@]2([C@@H]([C@H]1C)C2)C(C)C | 261491 |
| 458 | IMPHY011902 | beta-Thujone | O=C1C[C@]2([C@@H]([C@@H]1C)C2)C(C)C | 91456 |
| 459 | IMPHY011904 | cis-Thujone | O=C1C[C@]2([C@H]([C@@H]1C)C2)C(C)C | 249286 |
| 460 | IMPHY011957 | (+)-delta-Cadinene | CC1=C[C@@H]2C(=C(C)CC[C@H]2C(C)C)CC1 | 441005 |
| 461 | IMPHY011973 | (-)-cis-Carveol | CC(=C)[C@@H]1CC=C([C@@H](C1)O)C | 330573 |
| 462 | IMPHY011988 | (-)-trans-Carveol | CC(=C)[C@@H]1CC=C([C@H](C1)O)C | 94221 |
| 463 | IMPHY011999 | Carveol | CC(=C)C1CC=C(C(C1)O)C | 7438 |
| 464 | IMPHY012036 | Camphor | O=C1CC2C(C1(C)CC2)(C)C | 2537 |
| 465 | IMPHY012058 | Linalool | C=CC(CCC=C(C)C)(O)C | 6549 |
| 466 | IMPHY012061 | alpha-Pinene | CC1=CCC2CC1C2(C)C | 6654 |
| 467 | IMPHY012147 | beta-Pinene | C=C1CCC2CC1C2(C)C | 14896 |
| 468 | IMPHY012160 | alpha-Terpineol | CC1=CCC(CC1)C(O)(C)C | 17100 |
| 469 | IMPHY012165 | Sabinene | C=C1CCC2(C1C2)C(C)C | 18818 |
| 470 | IMPHY012255 | (+)-trans-Piperitenol | CC1=C[C@@H]([C@H](CC1)C(C)C)O | 85568 |
| 471 | IMPHY012586 | (-)-alpha-Cadinol | CC1=CC2C(CC1)[C@@](C)(O)CC[C@@H]2C(C)C | 6431302 |
| 472 | IMPHY012589 | 3-(1,5-Dimethyl-4-hexenyl)-6-methylene-1-cyclohexene | CC(C1CCC(=C)C=C1)CCC=C(C)C | 519764 |
| 473 | IMPHY012667 | Caryophyllene oxide | C=C1CC[C@H]2O[C@@]2(CC[C@@H]2[C@@H]1CC2(C)C)C | 1742210 |
| 474 | IMPHY012719 | Cosmosiin | OC[C@H]1O[C@@H](Oc2cc(O)c3c(c2)oc(cc3=O)c2ccc(cc2)O)[C@@H]([C@H]([C@@H]1O)O)O | 5280704 |
| 475 | IMPHY012739 | (Z)-beta-Ocimene | C=C/C(=CCC=C(C)C)/C | 5320250 |
| 476 | IMPHY014708 | beta-Selinene | C=C1CCC[C@]2([C@H]1C[C@@H](CC2)C(=C)C)C | 442393 |
| 477 | IMPHY014811 | alpha-Phellandrene | CC1=CCC(C=C1)C(C)C | 7460 |
| 478 | IMPHY014817 | Aromadendrene | CC1CCC2C1C1C(C1(C)C)CCC2=C | 91354 |
| 479 | IMPHY014831 | beta-Caryophyllene | C/C/1=CCCC(=C)[C@@H]2[C@@H](CC1)C(C2)(C)C | 5281515 |
| 480 | IMPHY014835 | (E)-beta-ocimene | C=C/C(=C/CC=C(C)C)/C | 5281553 |
| 481 | IMPHY014847 | Bornyl acetate | CC(=O)OC1CC2C(C1(C)CC2)(C)C | 6448 |
| 482 | IMPHY014852 | Camphene | C=C1C2CCC(C1(C)C)C2 | 6616 |
| 483 | IMPHY014873 | 2-Cyclohexen-1-ol, 3-methyl-6-(1-methylethyl)-, (1R,6S)-rel- | CC1=C[C@@H]([C@@H](CC1)C(C)C)O | 85567 |
| 484 | IMPHY014874 | cis-Sabinene hydrate | C[C@@H]1CC[C@@]2(C1C2)C(C)C | 101629835 |
| 485 | IMPHY014923 | Geraniol | OC/C=C(/CCC=C(C)C)C | 637566 |
| 486 | IMPHY014988 | Limonene | CC1=CCC(CC1)C(=C)C | 22311 |
| 487 | IMPHY015040 | 3-Pinanone | O=C1CC2CC(C1C)C2(C)C | 11038 |
| 488 | IMPHY015123 | alpha-Copaene | CC([C@@H]1CC[C@]2([C@@H]3[C@H]1C2C(=CC3)C)C)C | 70678558 |
| 489 | IMPHY016012 | Allo-Aromadendrene | C[C@@H]1CC[C@H]2[C@@H]1C1C(C1(C)C)CCC2=C | 42608158 |
| 490 | IMPHY016053 | Viridiflorol | C[C@@H]1CC[C@H]2[C@@H]1[C@H]1[C@H](C1(C)C)CC[C@]2(C)O | 11996452 |
| 491 | IMPHY000070 | Cadalene | Cc1ccc2c(c1)c(ccc2C)C(C)C | 10225 |
| 492 | IMPHY000099 | Myrtenol | OCC1=CCC2CC1C2(C)C | 10582 |
| 493 | IMPHY000308 | Hexadecane | CCCCCCCCCCCCCCCC | 11006 |
| 494 | IMPHY000491 | Pinene | CC1CCC2CC1C2(C)C |  |
| 495 | IMPHY000573 | Chamazulene | CCc1ccc(c2-c(c1)c(C)cc2)C | 10719 |
| 496 | IMPHY000915 | Chrysanthenone | CC1=CCC2C(=O)C1C2(C)C | 442463 |
| 497 | IMPHY001086 | Royleanone | O=C1C(=O)C(=C(C2=C1[C@@]1(C)CCCC([C@@H]1CC2)(C)C)O)C(C)C | 442084 |
| 498 | IMPHY001223 | 2,3-Dihydrobenzofuran | c1ccc2c(c1)OCC2 | 10329 |
| 499 | IMPHY001246 | Carvacrol | CC(c1ccc(c(c1)O)C)C | 10364 |
| 500 | IMPHY001493 | Methyl tridecanoate | CCCCCCCCCCCCC(=O)OC | 15608 |
| 501 | IMPHY001548 | Geranylacetone | C/C(=CCCC(=O)C)/CCC=C(C)C | 1549778 |
| 502 | IMPHY001721 | Cirsiliol | COc1cc2oc(cc(=O)c2c(c1OC)O)c1ccc(c(c1)O)O | 160237 |
| 503 | IMPHY001767 | Rosmadial | O=C[C@H]1C(C)(C)CCC[C@]21C(=O)Oc1c2c(C=O)cc(c1O)C(C)C | 15801061 |
| 504 | IMPHY001828 | 3-Octanol | CCCCCC(CC)O | 11527 |
| 505 | IMPHY001905 | Bisabolol oxide B | CC1=CCC(CC1)C1(C)CCC(O1)C(O)(C)C | 117301 |
| 506 | IMPHY002518 | Galdosol | O=C1O[C@H]2[C@@H]3[C@]1(CCCC3(C)C)c1c(C2=O)cc(c(c1O)O)C(C)C | 13966127 |
| 507 | IMPHY002684 | Z-harpagoside | OC[C@H]1O[C@@H](O[C@@H]2OC=C[C@@]3([C@H]2[C@@](C)(OC(=O)/C=Cc2ccccc2)C[C@H]3O)O)[C@@H]([C@H]([C@@H]1O)O)O | 13889700 |
| 508 | IMPHY002870 | Horminone | O[C@@H]1C[C@H]2C(C)(C)CCC[C@@]2(C2=C1C(=C(C(C)C)C(=O)C2=O)O)C | 2751795 |
| 509 | IMPHY002915 | Benzyl Alcohol | OCc1ccccc1 | 244 |
| 510 | IMPHY003174 | 5-Hydroxymethylfurfural | OCc1ccc(o1)C=O | 237332 |
| 511 | IMPHY003296 | Piperitenone | CC1=CC(=O)C(=C(C)C)CC1 | 381152 |
| 512 | IMPHY003485 | Myrcene | C=CC(=C)CCC=C(C)C | 31253 |
| 513 | IMPHY003490 | Coumarin | O=c1ccc2c(o1)cccc2 | 323 |
| 514 | IMPHY003495 | 2-Methoxy-4-vinylphenol | COc1cc(C=C)ccc1O | 332 |
| 515 | IMPHY003525 | Nonanal | CCCCCCCCC=O | 31289 |
| 516 | IMPHY003536 | Eugenol | C=CCc1ccc(c(c1)OC)O | 3314 |
| 517 | IMPHY003616 | Bicyclogermacrene | C/C/1=CCC/C(=C/[C@H]2[C@@H](CC1)C2(C)C)/C | 13894537 |
| 518 | IMPHY003631 | Valeranone | CC([C@H]1CC[C@@]2([C@@](C1)(C)C(=O)CCC2)C)C | 171455 |
| 519 | IMPHY003695 | (-)-Germacrene A | C/C/1=CCC/C(=C/C[C@H](CC1)C(=C)C)/C | 9548706 |
| 520 | IMPHY003710 | (-)-Isopulegol | C[C@@H]1CC[C@H]([C@@H](C1)O)C(=C)C | 170833 |
| 521 | IMPHY003719 | beta-Copaene | CC([C@@H]1CC[C@]2([C@@H]3[C@H]1C2C(=C)CC3)C)C | 57339298 |
| 522 | IMPHY003977 | (-)-beta-Bourbonene | CC([C@@H]1CC[C@@]2([C@H]1[C@H]1C(=C)CC[C@@H]21)C)C | 62566 |
| 523 | IMPHY003982 | gamma-Terpinene | CC1=CCC(=CC1)C(C)C | 7461 |
| 524 | IMPHY004017 | Manool | C=C[C@@](CC[C@H]1C(=C)CC[C@@H]2[C@]1(C)CCCC2(C)C)(O)C | 3034394 |
| 525 | IMPHY004049 | alpha-Santalol | OC/C(=CCC[C@]1(C)C2C[C@@H]3C1(C)[C@@H]3C2)/C | 11085337 |
| 526 | IMPHY004077 | Verbenone | CC1=CC(=O)C2CC1C2(C)C | 29025 |
| 527 | IMPHY004194 | (1S,2R,5S)-2-isopropyl-5-methylcyclohexyl acetate | C[C@H]1CC[C@@H]([C@H](C1)OC(=O)C)C(C)C | 62335 |
| 528 | IMPHY004281 | Guaiol | C[C@H]1CC[C@H](CC2=C1CC[C@@H]2C)C(O)(C)C | 227829 |
| 529 | IMPHY004302 | Arachidonic acid | CCCCC/C=CC/C=CC/C=CC/C=CCCCC(=O)O | 444899 |
| 530 | IMPHY004326 | Rosmanol | O=C1O[C@H]2[C@@H]3[C@]1(CCCC3(C)C)c1c([C@@H]2O)cc(c(c1O)O)C(C)C | 13966122 |
| 531 | IMPHY004332 | (1R,8R,9S,10S)-3,4,8-trihydroxy-11,11-dimethyl-5-propan-2-yl-16-oxatetracyclo[7.5.2.01,10.02,7]hexadeca-2,4,6-trien-15-one | O=C1O[C@H]2[C@@H]3[C@]1(CCCC3(C)C)c1c([C@H]2O)cc(c(c1O)O)C(C)C | 23243694 |
| 532 | IMPHY004399 | Methyl linolenate | CC/C=CC/C=CC/C=CCCCCCCCC(=O)OC | 5319706 |
| 533 | IMPHY004555 | 1,3,3-Trimethyl-2-oxabicyclo[2.2.2]oct-5-ene | CC12CCC(C=C1)C(O2)(C)C | 523035 |
| 534 | IMPHY004597 | Rosmarinic acid | O=C(O[C@@H](C(=O)O)Cc1ccc(c(c1)O)O)/C=C/c1ccc(c(c1)O)O | 5281792 |
| 535 | IMPHY004608 | Salvianolic acid A | O=C(O[C@@H](C(=O)O)Cc1ccc(c(c1)O)O)/C=C/c1ccc(c(c1/C=C/c1ccc(c(c1)O)O)O)O | 5281793 |
| 536 | IMPHY004660 | Luteolin | Oc1cc(O)c2c(c1)oc(cc2=O)c1ccc(c(c1)O)O | 5280445 |
| 537 | IMPHY004661 | Apigenin | Oc1ccc(cc1)c1cc(=O)c2c(o1)cc(cc2O)O | 5280443 |
| 538 | IMPHY004971 | alpha-Bourbonene | CC(C1CCC2(C1C1C(=CCC21)C)C)C | 530816 |
| 539 | IMPHY005084 | beta-Boswellic acid | C[C@@H]1CC[C@]2([C@@H]([C@H]1C)C1=CC[C@H]3[C@@]([C@@]1(CC2)C)(C)CC[C@@H]1[C@]3(C)CC[C@H]([C@]1(C)C(=O)O)O)C | 168928 |
| 540 | IMPHY005196 | 7-O-Methylrosmanol | CO[C@@H]1[C@H]2OC(=O)[C@]3([C@@H]2C(C)(C)CCC3)c2c1cc(C(C)C)c(c2O)O | 23243692 |
| 541 | IMPHY005327 | Cirsimaritin | COc1cc2oc(cc(=O)c2c(c1OC)O)c1ccc(cc1)O | 188323 |
| 542 | IMPHY005345 | 1-Octen-3-OL | CCCCCC(C=C)O | 18827 |
| 543 | IMPHY005400 | Sclareol | C=C[C@@](CC[C@H]1[C@](C)(O)CC[C@@H]2[C@]1(C)CCCC2(C)C)(O)C | 163263 |
| 544 | IMPHY005442 | Hispidulin | COc1c(O)cc2c(c1O)c(=O)cc(o2)c1ccc(cc1)O | 5281628 |
| 545 | IMPHY005447 | 6-Hydroxyluteolin | Oc1ccc(cc1O)c1cc(=O)c2c(o1)cc(c(c2O)O)O | 5281642 |
| 546 | IMPHY005811 | 2-Pentylfuran | CCCCCc1ccco1 | 19602 |
| 547 | IMPHY005846 | 1-Hepten-5-yne, 2-methyl-3-methylene- | CC#CCC(=C)C(=C)C | 569164 |
| 548 | IMPHY005966 | Usnic acid | CC(=O)C1C(=O)C=C2C(C1=O)(C)c1c(O)c(C)c(c(c1O2)C(=O)C)O | 5646 |
| 549 | IMPHY006026 | 4-Methylbenzaldehyde | O=Cc1ccc(cc1)C | 7725 |
| 550 | IMPHY006145 | p-Cymene | Cc1ccc(cc1)C(C)C | 7463 |
| 551 | IMPHY006148 | 4-Hydroxyacetophenone | CC(=O)c1ccc(cc1)O | 7469 |
| 552 | IMPHY006279 | 2-Phenylethanol | OCCc1ccccc1 | 6054 |
| 553 | IMPHY006325 | Myrtenal | O=CC1=CCC2CC1C2(C)C | 61130 |
| 554 | IMPHY006347 | Hexanal | CCCCCC=O | 6184 |
| 555 | IMPHY006419 | 6-Methyl-3,5-heptadien-2-one | CC(=C/C=C/C(=O)C)C | 5370101 |
| 556 | IMPHY006485 | beta-Ionone | CC(=O)/C=C/C1=C(C)CCCC1(C)C | 638014 |
| 557 | IMPHY006486 | Squalene | C/C(=CCC/C=C(/CC/C=C(/CCC=C(C)C)C)C)/CC/C=C(/CCC=C(C)C)C | 638072 |
| 558 | IMPHY006519 | Linalool oxide, pyrane, (Z)- | C=C[C@]1(C)OC(C)(C)CC[C@@H]1O | 6431477 |
| 559 | IMPHY006550 | Thymol | Cc1ccc(c(c1)O)C(C)C | 6989 |
| 560 | IMPHY006653 | Carnosic acid | OC(=O)[C@@]12CCCC([C@@H]2CCc2c1c(O)c(c(c2)C(C)C)O)(C)C | 65126 |
| 561 | IMPHY006668 | Isophorone | CC1=CC(=O)CC(C1)(C)C | 6544 |
| 562 | IMPHY006696 | Methyleugenol | C=CCc1ccc(c(c1)OC)OC | 7127 |
| 563 | IMPHY006718 | 5-Hydroxymaltol | Cc1occ(c(=O)c1O)O | 70627 |
| 564 | IMPHY006793 | Lithospermic acid | O=C(O[C@@H](C(=O)O)Cc1ccc(c(c1)O)O)/C=C/c1ccc(c2c1[C@H](C(=O)O)[C@H](O2)c1ccc(c(c1)O)O)O | 6441498 |
| 565 | IMPHY006948 | beta-Terpineol | CC(=C)C1CCC(CC1)(C)O | 8748 |
| 566 | IMPHY006950 | Tricyclene | CC12C3C1CC(C2(C)C)C3 | 79035 |
| 567 | IMPHY006953 | 2,6-Dimethylpyrazine | Cc1cncc(n1)C | 7938 |
| 568 | IMPHY006965 | alpha,alpha-Dimethyl-4-methylenecyclohexanemethanol | CC(C1CCC(=C)CC1)(O)C | 81722 |
| 569 | IMPHY006971 | Methyl palmitate | CCCCCCCCCCCCCCCC(=O)OC | 8181 |
| 570 | IMPHY006981 | Indole | c1ccc2c(c1)[nH]cc2 | 798 |
| 571 | IMPHY007067 | Linalyl acetate | C=CC(OC(=O)C)(CCC=C(C)C)C | 8294 |
| 572 | IMPHY007138 | Dehydrovomifoliol | CC(=O)/C=C/[C@@]1(O)C(=CC(=O)CC1(C)C)C | 688492 |
| 573 | IMPHY007201 | Carvacrol methyl ether | COc1cc(ccc1C)C(C)C | 80790 |
| 574 | IMPHY007215 | Methyl dodecanoate | CCCCCCCCCCCC(=O)OC | 8139 |
| 575 | IMPHY007238 | Abietane | CC([C@H]1CC[C@H]2[C@H](C1)CC[C@@H]1[C@]2(C)CCCC1(C)C)C | 6857485 |
| 576 | IMPHY007331 | 6-Methyl-5-hepten-2-one | CC(=O)CCC=C(C)C | 9862 |
| 577 | IMPHY007355 | Gentiopicroside | C=C[C@H]1[C@@H](OC=C2C1=CCOC2=O)O[C@@H]1O[C@H](CO)[C@H]([C@@H]([C@H]1O)O)O | 88708 |
| 578 | IMPHY007376 | beta-Cubebene | CC([C@@H]1CC[C@H]([C@]23[C@H]1[C@H]2C(=C)CC3)C)C | 93081 |
| 579 | IMPHY007520 | Viridiflorene | C[C@@H]1CCC2=C(C)CC[C@@H]3[C@H]([C@H]12)C3(C)C | 10910653 |
| 580 | IMPHY007598 | Phenylacetic acid | OC(=O)Cc1ccccc1 | 999 |
| 581 | IMPHY007747 | Carvacryl acetate | CC(=O)Oc1cc(ccc1C)C(C)C | 80792 |
| 582 | IMPHY007840 | Spathulenol | C=C1CC[C@@H]2[C@H]([C@H]3[C@H]1CC[C@]3(C)O)C2(C)C | 92231 |
| 583 | IMPHY007853 | Camphane | CC1(C)C2CCC1(C)CC2 | 92108 |
| 584 | IMPHY008065 | Ethyl dodecanoate | CCCCCCCCCCCC(=O)OCC | 7800 |
| 585 | IMPHY008144 | 2,6,6-Trimethyl-2-cyclohexene-1,4-dione | O=C1C=C(C)C(=O)C(C1)(C)C | 62374 |
| 586 | IMPHY008150 | 1-Methyl-4-(prop-1-en-2-yl)benzene | Cc1ccc(cc1)C(=C)C | 62385 |
| 587 | IMPHY008395 | Methyl Carnosate | COC(=O)[C@@]12CCCC([C@@H]2CCc2c1c(O)c(c(c2)C(C)C)O)(C)C | 11336941 |
| 588 | IMPHY008937 | Vitamin E | C[C@@H](CCC[C@]1(C)CCc2c(O1)c(C)c(c(c2C)O)C)CCC[C@@H](CCCC(C)C)C | 14985 |
| 589 | IMPHY008998 | Phenanthrene | c1ccc2c(c1)c1ccccc1cc2 | 995 |
| 590 | IMPHY009268 | Oplopanone | CC([C@@H]1CC[C@@]([C@H]2[C@H]1[C@H](CC2)C(=O)C)(C)O)C | 10466745 |
| 591 | IMPHY009359 | Hexacosane | CCCCCCCCCCCCCCCCCCCCCCCCCC | 12407 |
| 592 | IMPHY009368 | Heptadecane | CCCCCCCCCCCCCCCCC | 12398 |
| 593 | IMPHY009413 | Triacontane | CCCCCCCCCCCCCCCCCCCCCCCCCCCCCC | 12535 |
| 594 | IMPHY009419 | Tridecane | CCCCCCCCCCCCC | 12388 |
| 595 | IMPHY009624 | Ethyl palmitate | CCCCCCCCCCCCCCCC(=O)OCC | 12366 |
| 596 | IMPHY009729 | Verbenene | C=C1C=CC2CC1C2(C)C | 6427476 |
| 597 | IMPHY009752 | beta-Cyclocitral | O=CC1=C(C)CCCC1(C)C | 9895 |
| 598 | IMPHY009757 | 6,6-Dimethyl-2-methylenebicyclo[3.1.1]hept-3-yl acetate | CC(=O)OC1CC2CC(C1=C)C2(C)C | 102553 |
| 599 | IMPHY009765 | beta-Calacorene | CC(C1CCC(=C)c2c1cc(C)cc2)C | 529621 |
| 600 | IMPHY009832 | Lavandulyl acetate | CC(=O)OCC(C(=C)C)CC=C(C)C | 30247 |
| 601 | IMPHY009874 | Myrtenyl acetate | CC(=O)OCC1=CCC2CC1C2(C)C | 61262 |
| 602 | IMPHY009889 | Hexyl isovalerate | CCCCCCOC(=O)CC(C)C | 61455 |
| 603 | IMPHY009946 | Benzaldehyde | O=Cc1ccccc1 | 240 |
| 604 | IMPHY009962 | Ethyl tetradecanoate | CCCCCCCCCCCCCC(=O)OCC | 31283 |
| 605 | IMPHY009996 | 2,6,6-Trimethylbicyclo[3.1.1]heptan-3-ol | OC1CC2CC(C1C)C2(C)C | 99038 |
| 606 | IMPHY010000 | Dodecane | CCCCCCCCCCCC | 8182 |
| 607 | IMPHY010072 | Eucalyptol | CC12CCC(CC1)C(O2)(C)C | 2758 |
| 608 | IMPHY010080 | beta-Elemene | C=C[C@]1(C)CC[C@H](C[C@H]1C(=C)C)C(=C)C | 6918391 |
| 609 | IMPHY010083 | 4-Hydroxybenzoic acid | Oc1ccc(cc1)C(=O)O | 135 |
| 610 | IMPHY010097 | Benzyl benzoate | O=C(c1ccccc1)OCc1ccccc1 | 2345 |
| 611 | IMPHY010179 | (-)-beta-Chamigrene | CC1=CC[C@@]2(CC1)C(=C)CCCC2(C)C | 442353 |
| 612 | IMPHY010189 | Thunbergene | C/C/1=C/CC/C(=C/C/C=C(C=C[C@@H](CC1)C(C)C)/C)/C | 11747713 |
| 613 | IMPHY010603 | beta-Cadinene | CC1=CC[C@@H]2[C@@H](C1)[C@@H](CC=C2C)C(C)C | 10657 |
| 614 | IMPHY010781 | Limonene oxide, cis-(-)- | CC(=C)[C@H]1CC[C@]2([C@@H](C1)O2)C | 6452061 |
| 615 | IMPHY010887 | Thymol acetate | CC(=O)Oc1cc(C)ccc1C(C)C | 68252 |
| 616 | IMPHY011051 | Abietatriene | CC(c1ccc2c(c1)CC[C@@H]1[C@]2(C)CCCC1(C)C)C | 6432211 |
| 617 | IMPHY011215 | Tetradecane | CCCCCCCCCCCCCC | 12389 |
| 618 | IMPHY011223 | iso-3-Thujyl acetate | CC(=O)OC1CC2(C(C1C)C2)C(C)C | 524251 |
| 619 | IMPHY011239 | Carnosol | O=C1O[C@H]2C[C@@H]3[C@]1(CCCC3(C)C)c1c2cc(c(c1O)O)C(C)C | 442009 |
| 620 | IMPHY011321 | (E,E)-3,5-octadien-2-one | CC/C=C/C=C/C(=O)C | 5352876 |
| 621 | IMPHY011347 | (E)-Sesquilavandulol | OCC(C(=C)C)C/C=C(/CCC=C(C)C)C | 5352145 |
| 622 | IMPHY011392 | 3-Carene | CC1=CCC2C(C1)C2(C)C | 26049 |
| 623 | IMPHY011396 | 4-Carvomenthenol | CC1=CCC(CC1)(O)C(C)C | 11230 |
| 624 | IMPHY011407 | alpha-Campholenal | O=CC[C@H]1CC=C(C1(C)C)C | 1252759 |
| 625 | IMPHY011519 | alpha-Terpinyl acetate | CC(=O)OC(C1CCC(=CC1)C)(C)C | 111037 |
| 626 | IMPHY011534 | luteolin 7-O-beta-d-glucopyranoside | OC[C@H]1O[C@@H](Oc2cc(O)c3c(c2)oc(cc3=O)c2ccc(c(c2)O)O)[C@@H]([C@@H]([C@H]1O)O)O | 13093777 |
| 627 | IMPHY011552 | (1R)-2-methyl-5-propan-2-ylbicyclo[3.1.0]hex-2-ene | CC1=CCC2([C@@H]1C2)C(C)C | 6451618 |
| 628 | IMPHY011562 | 2-Hexenal | CCC/C=C/C=O | 5281168 |
| 629 | IMPHY011568 | alpha-Fenchyl acetate | CC(=O)O[C@@H]1[C@]2(C)CC[C@H](C1(C)C)C2 | 7530950 |
| 630 | IMPHY011586 | (S,1Z,6Z)-8-Isopropyl-1-methyl-5-methylenecyclodeca-1,6-diene | C/C/1=C/CCC(=C)/C=C[C@@H](CC1)C(C)C | 91723653 |
| 631 | IMPHY011590 | d-Borneol | O[C@@H]1C[C@H]2C([C@@]1(C)CC2)(C)C | 61060 |
| 632 | IMPHY011599 | Terpinolene | CC1=CCC(=C(C)C)CC1 | 11463 |
| 633 | IMPHY011632 | Farnesol | OC/C=C(/CC/C=C(/CCC=C(C)C)C)C | 445070 |
| 634 | IMPHY011643 | alpha-Terpinene | CC1=CC=C(CC1)C(C)C | 7462 |
| 635 | IMPHY011646 | Cynaroside | OC[C@H]1O[C@@H](Oc2cc(O)c3c(c2)oc(cc3=O)c2ccc(c(c2)O)O)[C@@H]([C@H]([C@@H]1O)O)O | 5280637 |
| 636 | IMPHY011647 | Geranyl acetate | C/C(=CCOC(=O)C)/CCC=C(C)C | 1549026 |
| 637 | IMPHY011648 | Neryl acetate | C/C(=C/COC(=O)C)/CCC=C(C)C | 1549025 |
| 638 | IMPHY011657 | cis-beta-Farnesene | C=CC(=C)CC/C=C(CCC=C(C)C)/C | 5317319 |
| 639 | IMPHY011659 | alpha-Muurolene | CC1=C[C@@H]2[C@H](CC1)C(=CC[C@H]2C(C)C)C | 12306047 |
| 640 | IMPHY011660 | (+)-alpha-Cadinene | CC1=C[C@@H]2[C@@H](CC1)C(=CC[C@H]2C(C)C)C | 12306048 |
| 641 | IMPHY011667 | alpha-Gurjunene | C[C@@H]1CC[C@@H]2[C@H](C3=C(CC[C@H]13)C)C2(C)C | 15560276 |
| 642 | IMPHY011707 | beta-Carotene | C/C(=CC=CC=C(C=CC=C(C=CC1=C(C)CCCC1(C)C)/C)/C)/C=C/C=C(/C=C/C1=C(C)CCCC1(C)C)C | 5280489 |
| 643 | IMPHY011709 | alpha-Eudesmol | CC1=CCC[C@]2([C@H]1C[C@@H](CC2)C(O)(C)C)C | 92762 |
| 644 | IMPHY011749 | Humulene epoxide II | C/C/1=CCC(C)(C)/C=C/C[C@@]2([C@@H](CC1)O2)C | 10704181 |
| 645 | IMPHY011752 | beta-SANTALOL | OC/C(=CCC[C@]1(C)[C@H]2CC[C@@H](C1=C)C2)/C | 6857681 |
| 646 | IMPHY011761 | Humulene | C/C/1=CCC(C)(C)/C=C/C/C(=C/CC1)/C | 5281520 |
| 647 | IMPHY011762 | cis-Anethole | C/C=Cc1ccc(cc1)OC | 1549040 |
| 648 | IMPHY011763 | Anethole | C/C=C/c1ccc(cc1)OC | 637563 |
| 649 | IMPHY011789 | Citral | O=C/C=C(/CCC=C(C)C)C | 638011 |
| 650 | IMPHY011790 | Neral | O=C/C=C(CCC=C(C)C)/C | 643779 |
| 651 | IMPHY011792 | gamma-Muurolene | CC1=C[C@@H]2[C@H](CC1)C(=C)CC[C@H]2C(C)C | 12313020 |
| 652 | IMPHY011797 | Oleic acid | CCCCCCCC/C=CCCCCCCCC(=O)O | 445639 |
| 653 | IMPHY011817 | alpha-Farnesene | C=C/C(=C/C/C=C(/CCC=C(C)C)C)/C | 5281516 |
| 654 | IMPHY011825 | 3-Epioleanolic acid | O[C@@H]1CC[C@]2([C@H](C1(C)C)CC[C@@]1([C@@H]2CC=C2[C@@]1(C)CC[C@@]1([C@H]2CC(C)(C)CC1)C(=O)O)C)C | 11869658 |
| 655 | IMPHY011826 | Oleanolic acid | O[C@H]1CC[C@]2([C@H](C1(C)C)CC[C@@]1([C@@H]2CC=C2[C@@]1(C)CC[C@@]1([C@H]2CC(C)(C)CC1)C(=O)O)C)C | 10494 |
| 656 | IMPHY011872 | (4E,7E)-1,5,9,9-Tetramethyl-12-oxabicyclo[9.1.0]dodeca-4,7-diene | C/C/1=C/CCC2(C)OC2CC(/C=CC1)(C)C | 22559443 |
| 657 | IMPHY011873 | Humulene epoxide | C/C/1=CCCC2(C)OC2CC(/C=C/C1)(C)C | 5352470 |
| 658 | IMPHY011880 | Ursolic acid | C[C@@H]1CC[C@]2([C@@H]([C@H]1C)C1=CC[C@H]3[C@@]([C@@]1(CC2)C)(C)CC[C@@H]1[C@]3(C)CC[C@@H](C1(C)C)O)C(=O)O | 64945 |
| 659 | IMPHY011884 | Pulegone | C[C@@H]1CCC(=C(C)C)C(=O)C1 | 442495 |
| 660 | IMPHY011901 | Thujone | O=C1C[C@]2([C@@H]([C@H]1C)C2)C(C)C | 261491 |
| 661 | IMPHY011902 | beta-Thujone | O=C1C[C@]2([C@@H]([C@@H]1C)C2)C(C)C | 91456 |
| 662 | IMPHY011903 | (+)-alpha-Thujone | O=C1C[C@@]2([C@H]([C@@H]1C)C2)C(C)C | 12304612 |
| 663 | IMPHY011904 | cis-Thujone | O=C1C[C@]2([C@H]([C@@H]1C)C2)C(C)C | 249286 |
| 664 | IMPHY011933 | Caffeic acid | OC(=O)/C=C/c1ccc(c(c1)O)O | 689043 |
| 665 | IMPHY011957 | (+)-delta-Cadinene | CC1=C[C@@H]2C(=C(C)CC[C@H]2C(C)C)CC1 | 441005 |
| 666 | IMPHY011965 | (+)-beta-Phellandrene | CC([C@@H]1CCC(=C)C=C1)C | 442484 |
| 667 | IMPHY011971 | 2alpha,3alpha-Dihydroxyolean-12-en-28-oic acid | O[C@@H]1C[C@@]2(C)C(C([C@@H]1O)(C)C)CC[C@@]1([C@@H]2CC=C2[C@@]1(C)CC[C@@]1([C@@H]2CC(C)(C)CC1)C(=O)O)C | 179482 |
| 668 | IMPHY011973 | (-)-cis-Carveol | CC(=C)[C@@H]1CC=C([C@@H](C1)O)C | 330573 |
| 669 | IMPHY011988 | (-)-trans-Carveol | CC(=C)[C@@H]1CC=C([C@H](C1)O)C | 94221 |
| 670 | IMPHY012036 | Camphor | O=C1CC2C(C1(C)CC2)(C)C | 2537 |
| 671 | IMPHY012058 | Linalool | C=CC(CCC=C(C)C)(O)C | 6549 |
| 672 | IMPHY012061 | alpha-Pinene | CC1=CCC2CC1C2(C)C | 6654 |
| 673 | IMPHY012075 | Carvone | CC(=C)C1CC=C(C(=O)C1)C | 7439 |
| 674 | IMPHY012104 | Citronellol | OCCC(CCC=C(C)C)C | 8842 |
| 675 | IMPHY012147 | beta-Pinene | C=C1CCC2CC1C2(C)C | 14896 |
| 676 | IMPHY012160 | alpha-Terpineol | CC1=CCC(CC1)C(O)(C)C | 17100 |
| 677 | IMPHY012165 | Sabinene | C=C1CCC2(C1C2)C(C)C | 18818 |
| 678 | IMPHY012168 | (1S,2S,6S,7R,8R)-1,3-dimethyl-8-propan-2-yltricyclo[4.4.0.02,7]dec-3-ene | CC([C@H]1CC[C@]2([C@@H]3[C@@H]1[C@H]2C(=CC3)C)C)C | 101607926 |
| 679 | IMPHY012178 | p-Menthan-3-one | CC1CCC(C(=O)C1)C(C)C | 6986 |
| 680 | IMPHY012278 | Lyral | O=CC1CCC(=CC1)CCCC(O)(C)C | 91604 |
| 681 | IMPHY012292 | trans-Sabinol | CC([C@]12CC2C(=C)[C@@H](C1)O)C | 6429076 |
| 682 | IMPHY012487 | Menthofuran | CC1CCc2c(C1)occ2C | 329983 |
| 683 | IMPHY012550 | Vicenin-2 | OC[C@H]1O[C@H]([C@@H]([C@H]([C@@H]1O)O)O)c1c(O)c([C@@H]2O[C@H](CO)[C@H]([C@@H]([C@H]2O)O)O)c(c2c1oc(cc2=O)c1ccc(cc1)O)O | 442664 |
| 684 | IMPHY012586 | (-)-alpha-Cadinol | CC1=CC2C(CC1)[C@@](C)(O)CC[C@@H]2C(C)C | 6431302 |
| 685 | IMPHY012589 | 3-(1,5-Dimethyl-4-hexenyl)-6-methylene-1-cyclohexene | CC(C1CCC(=C)C=C1)CCC=C(C)C | 519764 |
| 686 | IMPHY012654 | Nerol | OC/C=C(CCC=C(C)C)/C | 643820 |
| 687 | IMPHY012667 | Caryophyllene oxide | C=C1CC[C@H]2O[C@@]2(CC[C@@H]2[C@@H]1CC2(C)C)C | 1742210 |
| 688 | IMPHY012712 | Phytol | OC/C=C(/CCC[C@@H](CCC[C@@H](CCCC(C)C)C)C)C | 5280435 |
| 689 | IMPHY012723 | Linolenic acid | CC/C=CC/C=CC/C=CCCCCCCCC(=O)O | 5280934 |
| 690 | IMPHY012738 | Isocaryophyllene | C/C/1=C/CCC(=C)[C@@H]2[C@@H](CC1)C(C2)(C)C | 5281522 |
| 691 | IMPHY012739 | (Z)-beta-Ocimene | C=C/C(=CCC=C(C)C)/C | 5320250 |
| 692 | IMPHY012767 | 9,12,15-Octadecatrienal | O=CCCCCCCC/C=C/C/C=C/C/C=C/CC | 5283384 |
| 693 | IMPHY012910 | trans-Calamenene | CC([C@H]1CC[C@@H](c2c1cc(C)cc2)C)C | 6429022 |
| 694 | IMPHY012914 | trans-Sabinyl acetate | CC(=O)O[C@H]1C[C@@]2(C(C1=C)C2)C(C)C | 6430313 |
| 695 | IMPHY012915 | allo-Aromadendrene epoxide | CC1CCC2C1[C@@H]1[C@@H](C1(C)C)CCC12OC1 | 91746712 |
| 696 | IMPHY012920 | 2-Furanmethanol, 5-ethenyltetrahydro-alpha,alpha,5-trimethyl-, cis- | C=C[C@@]1(C)CC[C@H](O1)C(O)(C)C | 11116492 |
| 697 | IMPHY013080 | alpha-Calacorene | CC([C@@H]1CC=C(c2c1cc(C)cc2)C)C | 12302243 |
| 698 | IMPHY013133 | (Z)-p-Menth-2-en-1-ol | CC([C@@H]1CC[C@](C=C1)(C)O)C | 13918681 |
| 699 | IMPHY014115 | 2-Isopropyl-1,4-hexadiene | C/C=C/CC(=C)C(C)C | 6429324 |
| 700 | IMPHY014732 | 14-Hydroxy-9-epi-beta-Caryophyllene | OCC1(C)C[C@@H]2[C@H]1CC/C(=CCCC2=C)/C | 91747230 |
| 701 | IMPHY014801 | Zizanene | CC1=C[C@@H]2[C@H](CC1)C(=CC[C@@H]2C(C)C)C | 12306046 |
| 702 | IMPHY014803 | Tetrahydro-2,2,6-trimethyl-6-(4-methyl-3-cyclohexen-1-yl)-2H-pyran-3-ol | CC1=CCC(CC1)C1(C)CCC(C(O1)(C)C)O | 90806 |
| 703 | IMPHY014806 | Caswell No. 264AB | CC([C@@H]1CC[C@H]([C@]23[C@H]1[C@H]2C(=CC3)C)C)C | 442359 |
| 704 | IMPHY014811 | alpha-Phellandrene | CC1=CCC(C=C1)C(C)C | 7460 |
| 705 | IMPHY014817 | Aromadendrene | CC1CCC2C1C1C(C1(C)C)CCC2=C | 91354 |
| 706 | IMPHY014831 | beta-Caryophyllene | C/C/1=CCCC(=C)[C@@H]2[C@@H](CC1)C(C2)(C)C | 5281515 |
| 707 | IMPHY014835 | (E)-beta-ocimene | C=C/C(=C/CC=C(C)C)/C | 5281553 |
| 708 | IMPHY014836 | beta-Sitosterol | CC[C@@H](C(C)C)CC[C@H]([C@H]1CC[C@@H]2[C@]1(C)CC[C@H]1[C@H]2CC=C2[C@]1(C)CC[C@@H](C2)O)C | 222284 |
| 709 | IMPHY014847 | Bornyl acetate | CC(=O)OC1CC2C(C1(C)CC2)(C)C | 6448 |
| 710 | IMPHY014852 | Camphene | C=C1C2CCC(C1(C)C)C2 | 6616 |
| 711 | IMPHY014874 | cis-Sabinene hydrate | C[C@@H]1CC[C@@]2(C1C2)C(C)C | 101629835 |
| 712 | IMPHY014923 | Geraniol | OC/C=C(/CCC=C(C)C)C | 637566 |
| 713 | IMPHY014988 | Limonene | CC1=CCC(CC1)C(=C)C | 22311 |
| 714 | IMPHY014990 | Linoleic acid | CCCCC/C=CC/C=CCCCCCCCC(=O)O | 5280450 |
| 715 | IMPHY015003 | Menthol | CC1CCC(C(C1)O)C(C)C | 1254 |
| 716 | IMPHY015004 | Menthone | C[C@@H]1CC[C@H](C(=O)C1)C(C)C | 26447 |
| 717 | IMPHY015011 | Methylisoeugenol | C/C=C/c1ccc(c(c1)OC)OC | 637776 |
| 718 | IMPHY015022 | Nerolidol | C=CC(CC/C=C(/CCC=C(C)C)C)(O)C | 5284507 |
| 719 | IMPHY015040 | 3-Pinanone | O=C1CC2CC(C1C)C2(C)C | 11038 |
| 720 | IMPHY015042 | Piperitone | CC1=CC(=O)C(CC1)C(C)C | 6987 |
| 721 | IMPHY015095 | 2-Cyclohexen-1-ol, 1-methyl-4-(1-methylethyl)-, trans- | CC([C@@H]1CC[C@@](C=C1)(C)O)C | 122484 |
| 722 | IMPHY015123 | alpha-Copaene | CC([C@@H]1CC[C@]2([C@@H]3[C@H]1C2C(=CC3)C)C)C | 70678558 |
| 723 | IMPHY015153 | Dihydropyridine | N1CC=CC=C1 | 407038 |
| 724 | IMPHY015185 | 1H-Cycloprop[e]azulene | c1cc2-c(c3c(c1)C3)ccc2 | 15549266 |
| 725 | IMPHY015197 | 3-(Naphthalen-1-yl)propan-1-ol | OCCCc1cccc2c1cccc2 | 14711518 |
| 726 | IMPHY015221 | 2(3H)-Furanone | O=C1CC=CO1 | 140765 |
| 727 | IMPHY015511 | Bicyclo[3.1.1]heptane | C1CC2CC(C1)C2 | 638057 |
| 728 | IMPHY015512 | Bicyclo[6.1.0]nonane | C1CCCC2C(CC1)C2 | 136124 |
| 729 | IMPHY015591 | Octadecamethylcyclononasiloxane | C[Si]1(C)O[Si](C)(C)O[Si](C)(C)O[Si](C)(C)O[Si](C)(C)O[Si](O[Si](O[Si](O[Si](O1)(C)C)(C)C)(C)C)(C)C | 11172 |
| 730 | IMPHY015675 | Tetracosamethyl-cyclododecasiloxane | C[Si]1(C)O[Si](C)(C)O[Si](C)(C)O[Si](C)(C)O[Si](C)(C)O[Si](C)(C)O[Si](O[Si](O[Si](O[Si](O[Si](O[Si](O1)(C)C)(C)C)(C)C)(C)C)(C)C)(C)C | 167767 |
| 731 | IMPHY015731 | Tetradecamethylhexasiloxane | C[Si](O[Si](O[Si](C)(C)C)(C)C)(O[Si](O[Si](O[Si](C)(C)C)(C)C)(C)C)C | 7875 |
| 732 | IMPHY015971 | 6-methyl-3-[(2Z)-6-methylhepta-2,5-dien-2-yl]-7-oxabicyclo[4.1.0]heptane | CC(=CC/C=C(C1CCC2(C(C1)O2)C)/C)C | 5363099 |
| 733 | IMPHY016012 | Allo-Aromadendrene | C[C@@H]1CC[C@H]2[C@@H]1C1C(C1(C)C)CCC2=C | 42608158 |
| 734 | IMPHY016014 | Isobornyl acetate | CC(=O)O[C@H]1C[C@@H]2C([C@]1(C)CC2)(C)C | 247573 |
| 735 | IMPHY016047 | trans-Verbenyl acetate | CC(=O)O[C@H]1C=C(C)[C@H]2C[C@@H]1C2(C)C | 6428417 |
| 736 | IMPHY016053 | Viridiflorol | C[C@@H]1CC[C@H]2[C@@H]1[C@H]1[C@H](C1(C)C)CC[C@]2(C)O | 11996452 |
| 737 | IMPHY016054 | trans-alpha-Bergamotene | CC(=CCC[C@]1(C)[C@H]2CC=C([C@@H]1C2)C)C | 6429302 |
| 738 | IMPHY016950 | Norcamphor | O=C1CC2CC1CC2 | 10345 |
| 739 | IMPHY017019 | 2-Palmitoylglycerol | CCCCCCCCCCCCCCCC(=O)OC(CO)CO | 123409 |
| 740 | IMPHY017055 | 1-Naphthaleneethanol | OCCc1cccc2c1cccc2 | 13047 |
| 741 | IMPHY017333 | 3-Thujanol | CC(C12CCC(C2(C1)O)C)C | 527259 |
| 742 | IMPHY017650 | 1,2-Epoxyhumulene | CC1CCCC2(C)OC2CC(/C=C/C1)(C)C | 6432846 |
| 743 | IMPHY017655 | 1,5,9-Trimethyl-13-oxabicyclo[10.1.0]trideca-4,8-diene | C/C/1=CCCC2(C)OC2CC/C(=C/CC1)/C | 6436727 |
| 744 | IMPHY017656 | 2-Butenoic acid, 2-methyl-, 3-methylpentyl ester, (2Z)- | CCC(CCOC(=O)/C(=CC)/C)C | 6437020 |
| 745 | IMPHY017672 | Acetic acid;1,7,7-trimethylbicyclo[2.2.1]heptan-2-ol | OC1CC2C(C1(C)CC2)(C)C.CC(=O)O | 67173459 |
| 746 | IMPHY017747 | Nortricyclene | C1C2CC3C1C3C2 | 78962 |
| 747 | IMPHY017844 | Labd-7,13-dien-15-ol | OC/C=C(/CCC1C(=CCC2[C@]1(C)CCCC2(C)C)C)C | 91747910 |
| 748 | IMPHY017920 | 14-Hydroxy-z-caryophyllene | OCC1CCCC(C2C(/C=C/1)C(C2)(C)C)C |  |
| 749 | IMPHY017952 | 5, 7-Dimethoxy-1-naphthol | COc1cc(OC)cc2c1cccc2O |  |
|  |  |  |  |  |
| **PLANT NAME : Sambucus nigra** | | | | |
| **Serial No** | **IMPPAT Phytochemical Identifier** | **Phytochemical Name** | **SMILES** | **CID** |
| 1 | IMPHY001896 | Heptacosane | CCCCCCCCCCCCCCCCCCCCCCCCCCC | 11636 |
| 2 | IMPHY004055 | Choline | OCC[N+](C)(C)C | 305 |
| 3 | IMPHY004165 | Sambunigrin | OC[C@H]1O[C@@H](O[C@@H](c2ccccc2)C#N)[C@@H]([C@H]([C@@H]1O)O)O | 91434 |
| 4 | IMPHY004271 | Betulin | OC[C@@]12CC[C@H]([C@@H]2[C@@H]2[C@](CC1)(C)[C@]1(C)CC[C@@H]3[C@]([C@H]1CC2)(C)CC[C@@H](C3(C)C)O)C(=C)C | 72326 |
| 5 | IMPHY007273 | 1-Hexacosanol | CCCCCCCCCCCCCCCCCCCCCCCCCCO | 68171 |
| 6 | IMPHY011273 | alpha-Amyrenone | C[C@@H]1CC[C@]2([C@@H]([C@H]1C)C1=CC[C@H]3[C@@]([C@@]1(CC2)C)(C)CC[C@@H]1[C@]3(C)CCC(=O)C1(C)C)C | 12306155 |
| 7 | IMPHY011619 | alpha-Amyrin | C[C@@H]1CC[C@]2([C@@H]([C@H]1C)C1=CC[C@H]3[C@@]([C@@]1(CC2)C)(C)CC[C@@H]1[C@]3(C)CC[C@@H](C1(C)C)O)C | 73170 |
| 8 | IMPHY011826 | Oleanolic acid | O[C@H]1CC[C@]2([C@H](C1(C)C)CC[C@@]1([C@@H]2CC=C2[C@@]1(C)CC[C@@]1([C@H]2CC(C)(C)CC1)C(=O)O)C)C | 10494 |
| 9 | IMPHY011880 | Ursolic acid | C[C@@H]1CC[C@]2([C@@H]([C@H]1C)C1=CC[C@H]3[C@@]([C@@]1(CC2)C)(C)CC[C@@H]1[C@]3(C)CC[C@@H](C1(C)C)O)C(=O)O | 64945 |
| 10 | IMPHY014836 | beta-Sitosterol | CC[C@@H](C(C)C)CC[C@H]([C@H]1CC[C@@H]2[C@]1(C)CC[C@H]1[C@H]2CC=C2[C@]1(C)CC[C@@H](C2)O)C | 222284 |
| 11 | IMPHY006279 | 2-Phenylethanol | OCCc1ccccc1 | 6054 |
| 12 | IMPHY006300 | Cholesterol | CC(CCC[C@H]([C@H]1CC[C@@H]2[C@]1(C)CC[C@H]1[C@H]2CC=C2[C@]1(C)CC[C@@H](C2)O)C)C | 5997 |
| 13 | IMPHY007327 | Palmitic acid | CCCCCCCCCCCCCCCC(=O)O | 985 |
| 14 | IMPHY011393 | 24-Methylenecycloartanol | C=C(C(C)C)CC[C@H]([C@H]1CC[C@@]2([C@]1(C)CC[C@@]13[C@H]2CC[C@@H]2[C@]3(C1)CC[C@@H](C2(C)C)O)C)C | 94204 |
| 15 | IMPHY011619 | alpha-Amyrin | C[C@@H]1CC[C@]2([C@@H]([C@H]1C)C1=CC[C@H]3[C@@]([C@@]1(CC2)C)(C)CC[C@@H]1[C@]3(C)CC[C@@H](C1(C)C)O)C | 73170 |
| 16 | IMPHY011642 | Cycloartenol | CC(=CCC[C@H]([C@H]1CC[C@@]2([C@]1(C)CC[C@@]13[C@H]2CC[C@@H]2[C@]3(C1)CC[C@@H](C2(C)C)O)C)C)C | 92110 |
| 17 | IMPHY011748 | isorhamnetin-3-O-glucoside | OC[C@H]1O[C@@H](Oc2c(oc3c(c2=O)c(O)cc(c3)O)c2ccc(c(c2)OC)O)[C@@H]([C@H]([C@@H]1O)O)O | 5318645 |
| 18 | IMPHY011757 | Isorhamnetin 3-glucoside | OC[C@H]1O[C@H](Oc2c(oc3c(c2=O)c(O)cc(c3)O)c2ccc(c(c2)OC)O)C(C([C@@H]1O)O)O | 44258009 |
| 19 | IMPHY011797 | Oleic acid | CCCCCCCC/C=CCCCCCCCC(=O)O | 445639 |
| 20 | IMPHY011844 | Chlorogenic acid | O=C(O[C@@H]1C[C@@](O)(C[C@H]([C@H]1O)O)C(=O)O)/C=C/c1ccc(c(c1)O)O | 1794427 |
| 21 | IMPHY011880 | Ursolic acid | C[C@@H]1CC[C@]2([C@@H]([C@H]1C)C1=CC[C@H]3[C@@]([C@@]1(CC2)C)(C)CC[C@@H]1[C@]3(C)CC[C@@H](C1(C)C)O)C(=O)O | 64945 |
| 22 | IMPHY012058 | Linalool | C=CC(CCC=C(C)C)(O)C | 6549 |
| 23 | IMPHY012223 | beta-Amyrin | O[C@H]1CC[C@]2([C@H](C1(C)C)CC[C@@]1([C@@H]2CC=C2[C@@]1(C)CC[C@@]1([C@H]2CC(C)(C)CC1)C)C)C | 73145 |
| 24 | IMPHY012473 | Lupeol | CC(=C)[C@@H]1CC[C@]2([C@H]1[C@H]1CC[C@H]3[C@@]([C@]1(C)CC2)(C)CC[C@@H]1[C@]3(C)CC[C@@H](C1(C)C)O)C | 259846 |
| 25 | IMPHY012721 | Isoquercitrin | OC[C@H]1O[C@@H](Oc2c(oc3c(c2=O)c(O)cc(c3)O)c2ccc(c(c2)O)O)[C@@H]([C@H]([C@@H]1O)O)O | 5280804 |
| 26 | IMPHY012723 | Linolenic acid | CC/C=CC/C=CC/C=CCCCCCCCC(=O)O | 5280934 |
| 27 | IMPHY012868 | Narcissin | COc1cc(ccc1O)c1oc2cc(O)cc(c2c(=O)c1O[C@@H]1O[C@H](CO[C@@H]2O[C@@H](C)[C@@H]([C@H]([C@H]2O)O)O)[C@H]([C@@H]([C@H]1O)O)O)O | 5481663 |
| 28 | IMPHY014842 | Stigmasterol | CC[C@@H](C(C)C)/C=C/[C@H]([C@H]1CC[C@@H]2[C@]1(C)CC[C@H]1[C@H]2CC=C2[C@]1(C)CC[C@@H](C2)O)C | 5280794 |
| 29 | IMPHY014955 | 5,7-dihydroxy-2-(4-hydroxy-3-methoxyphenyl)-4-oxo-4H-chromen-3-yl 6-O-(6-deoxy-alpha-D-mannopyranosyl)-beta-D-glucopyranoside | COc1cc(ccc1O)c1oc2cc(O)cc(c2c(=O)c1O[C@@H]1O[C@H](CO[C@H]2O[C@H](C)[C@H]([C@@H]([C@@H]2O)O)O)[C@H]([C@@H]([C@H]1O)O)O)O | 17751019 |
| 30 | IMPHY014990 | Linoleic acid | CCCCC/C=CC/C=CCCCCCCCC(=O)O | 5280450 |
| 31 | IMPHY015047 | Rutin | Oc1cc(O)c2c(c1)oc(c(c2=O)O[C@@H]1O[C@H](CO[C@@H]2O[C@@H](C)[C@@H]([C@H]([C@H]2O)O)O)[C@H]([C@@H]([C@H]1O)O)O)c1ccc(c(c1)O)O | 5280805 |
| 32 | IMPHY002588 | Flavylium | c1ccc(cc1)c1ccc2c([o+]1)cccc2 | 145858 |
| 33 | IMPHY007160 | Cyanidin 3-O-beta-D-sambubioside | OC[C@H]1O[C@@H](Oc2cc3c(O)cc(cc3[o+]c2c2ccc(c(c2)O)O)O)[C@@H]([C@H]([C@@H]1O)O)O[C@@H]1OC[C@H]([C@@H]([C@H]1O)O)O | 6602304 |
| 34 | IMPHY007327 | Palmitic acid | CCCCCCCCCCCCCCCC(=O)O | 985 |
| 35 | IMPHY011797 | Oleic acid | CCCCCCCC/C=CCCCCCCCC(=O)O | 445639 |
| 36 | IMPHY012723 | Linolenic acid | CC/C=CC/C=CC/C=CCCCCCCCC(=O)O | 5280934 |
| 37 | IMPHY014990 | Linoleic acid | CCCCC/C=CC/C=CCCCCCCCC(=O)O | 5280450 |
| 38 | IMPHY001081 | alpha-Amyrin palmitate | CCCCCCCCCCCCCCCC(=O)O[C@H]1CC[C@]2([C@H](C1(C)C)CC[C@@]1([C@@H]2CC=C2[C@@]1(C)CC[C@@]1([C@H]2[C@@H](C)[C@@H](CC1)C)C)C)C | 10394654 |
| 39 | IMPHY001308 | Retinol | OC/C=C(/C=C/C=C(/C=C/C1=C(C)CCCC1(C)C)C)C | 445354 |
| 40 | IMPHY002539 | beta-Amyrin palmitate | CCCCCCCCCCCCCCCC(=O)O[C@H]1CC[C@]2([C@H](C1(C)C)CC[C@@]1([C@@H]2CC=C2[C@@]1(C)CC[C@@]1([C@H]2CC(C)(C)CC1)C)C)C | 13915599 |
| 41 | IMPHY002588 | Flavylium | c1ccc(cc1)c1ccc2c([o+]1)cccc2 | 145858 |
| 42 | IMPHY002915 | Benzyl Alcohol | OCc1ccccc1 | 244 |
| 43 | IMPHY003138 | Keracyanin | Oc1cc(O)c2c(c1)[o+]c(c(c2)O[C@@H]1O[C@H](CO[C@@H]2O[C@@H](C)[C@@H]([C@H]([C@H]2O)O)O)[C@H]([C@@H]([C@H]1O)O)O)c1ccc(c(c1)O)O.[Cl-] | 29231 |
| 44 | IMPHY004055 | Choline | OCC[N+](C)(C)C | 305 |
| 45 | IMPHY004165 | Sambunigrin | OC[C@H]1O[C@@H](O[C@@H](c2ccccc2)C#N)[C@@H]([C@H]([C@@H]1O)O)O | 91434 |
| 46 | IMPHY004619 | Quercetin | Oc1cc(O)c2c(c1)oc(c(c2=O)O)c1ccc(c(c1)O)O | 5280343 |
| 47 | IMPHY006362 | Ascorbic acid | OC[C@@H]([C@H]1OC(=O)C(=C1O)O)O | 54670067 |
| 48 | IMPHY006574 | Ethyl benzoate | CCOC(=O)c1ccccc1 | 7165 |
| 49 | IMPHY006981 | Indole | c1ccc2c(c1)[nH]cc2 | 798 |
| 50 | IMPHY007450 | Oxalic acid | OC(=O)C(=O)O | 971 |
| 51 | IMPHY009946 | Benzaldehyde | O=Cc1ccccc1 | 240 |
| 52 | IMPHY011826 | Oleanolic acid | O[C@H]1CC[C@]2([C@H](C1(C)C)CC[C@@]1([C@@H]2CC=C2[C@@]1(C)CC[C@@]1([C@H]2CC(C)(C)CC1)C(=O)O)C)C | 10494 |
| 53 | IMPHY011880 | Ursolic acid | C[C@@H]1CC[C@]2([C@@H]([C@H]1C)C1=CC[C@H]3[C@@]([C@@]1(CC2)C)(C)CC[C@@H]1[C@]3(C)CC[C@@H](C1(C)C)O)C(=O)O | 64945 |
| 54 | IMPHY012402 | Campesterol | O[C@H]1CC[C@]2(C(=CC[C@@H]3[C@@H]2CC[C@]2([C@H]3CC[C@@H]2[C@@H](CC[C@H](C(C)C)C)C)C)C1)C | 173183 |
| 55 | IMPHY014836 | beta-Sitosterol | CC[C@@H](C(C)C)CC[C@H]([C@H]1CC[C@@H]2[C@]1(C)CC[C@H]1[C@H]2CC=C2[C@]1(C)CC[C@@H](C2)O)C | 222284 |
| 56 | IMPHY014842 | Stigmasterol | CC[C@@H](C(C)C)/C=C/[C@H]([C@H]1CC[C@@H]2[C@]1(C)CC[C@H]1[C@H]2CC=C2[C@]1(C)CC[C@@H](C2)O)C | 5280794 |
| 57 | IMPHY000060 | Myristic acid | CCCCCCCCCCCCCC(=O)O | 11005 |
| 58 | IMPHY003016 | Lauric acid | CCCCCCCCCCCC(=O)O | 3893 |
| 59 | IMPHY004631 | Stearic acid | CCCCCCCCCCCCCCCCCC(=O)O | 5281 |
| 60 | IMPHY007327 | Palmitic acid | CCCCCCCCCCCCCCCC(=O)O | 985 |
| 61 | IMPHY008306 | Heptadecenoic acid | CCCCCCCCCCCCCC/C=C/C(=O)O | 5282747 |
| 62 | IMPHY009924 | Tetradecenic acid | CCCCCCCCCCCC=CC(=O)O | 162384 |
| 63 | IMPHY011797 | Oleic acid | CCCCCCCC/C=CCCCCCCCC(=O)O | 445639 |
| 64 | IMPHY012723 | Linolenic acid | CC/C=CC/C=CC/C=CCCCCCCCC(=O)O | 5280934 |
| 65 | IMPHY014990 | Linoleic acid | CCCCC/C=CC/C=CCCCCCCCC(=O)O | 5280450 |
| 66 | IMPHY006889 | methyl (1R,4aS,8S,8aS)-3-hydroxy-1-methyl-8-[(2S,3R,4S,5S,6R)-3,4,5-trihydroxy-6-(hydroxymethyl)oxan-2-yl]oxy-1,3,4,4a,8,8a-hexahydropyrano[3,4-c]pyran-5-carboxylate | OC[C@H]1O[C@@H](O[C@@H]2OC=C([C@@H]3[C@H]2[C@@H](C)OC(C3)O)C(=O)OC)[C@@H]([C@H]([C@@H]1O)O)O | 102004523 |
| 67 | IMPHY001081 | alpha-Amyrin palmitate | CCCCCCCCCCCCCCCC(=O)O[C@H]1CC[C@]2([C@H](C1(C)C)CC[C@@]1([C@@H]2CC=C2[C@@]1(C)CC[C@@]1([C@H]2[C@@H](C)[C@@H](CC1)C)C)C)C | 10394654 |
| 68 | IMPHY001950 | 2-Methylheptane | CCCCCC(C)C | 11594 |
| 69 | IMPHY002516 | 1-O-feruloyl-beta-D-glucose | OC[C@H]1O[C@@H](OC(=O)/C=C/c2ccc(c(c2)OC)O)[C@@H]([C@H]([C@@H]1O)O)O | 13962928 |
| 70 | IMPHY003138 | Keracyanin | Oc1cc(O)c2c(c1)[o+]c(c(c2)O[C@@H]1O[C@H](CO[C@@H]2O[C@@H](C)[C@@H]([C@H]([C@H]2O)O)O)[C@H]([C@@H]([C@H]1O)O)O)c1ccc(c(c1)O)O.[Cl-] | 29231 |
| 71 | IMPHY003986 | Prunasin | OC[C@H]1O[C@@H](O[C@H](c2ccccc2)C#N)[C@@H]([C@H]([C@@H]1O)O)O | 119033 |
| 72 | IMPHY004619 | Quercetin | Oc1cc(O)c2c(c1)oc(c(c2=O)O)c1ccc(c(c1)O)O | 5280343 |
| 73 | IMPHY005106 | 1-Caffeoyl-beta-D-glucose | OC[C@H]1O[C@@H](OC(=O)/C=C/c2ccc(c(c2)O)O)[C@@H]([C@H]([C@@H]1O)O)O | 5281761 |
| 74 | IMPHY005783 | 2-Hydroxydocosanoic acid | CCCCCCCCCCCCCCCCCCCCC(C(=O)O)O | 193484 |
| 75 | IMPHY006279 | 2-Phenylethanol | OCCc1ccccc1 | 6054 |
| 76 | IMPHY007327 | Palmitic acid | CCCCCCCCCCCCCCCC(=O)O | 985 |
| 77 | IMPHY011273 | alpha-Amyrenone | C[C@@H]1CC[C@]2([C@@H]([C@H]1C)C1=CC[C@H]3[C@@]([C@@]1(CC2)C)(C)CC[C@@H]1[C@]3(C)CCC(=O)C1(C)C)C | 12306155 |
| 78 | IMPHY011306 | (R)-(beta-D-Glucopyranosyloxy)(3-hydroxyphenyl)acetonitrile | (R)-(beta-D-Glucopyranosyloxy)(3-hydroxyphenyl)acetonitrile | 92323 |
| 79 | IMPHY011393 | 24-Methylenecycloartanol | C=C(C(C)C)CC[C@H]([C@H]1CC[C@@]2([C@]1(C)CC[C@@]13[C@H]2CC[C@@H]2[C@]3(C1)CC[C@@H](C2(C)C)O)C)C | 94204 |
| 80 | IMPHY011394 | Arachidic acid | CCCCCCCCCCCCCCCCCCCC(=O)O | 10467 |
| 81 | IMPHY011428 | Zierin | OC[C@H]1O[C@@H](O[C@@H](c2cccc(c2)O)C#N)[C@@H]([C@H]([C@@H]1O)O)O | 441473 |
| 82 | IMPHY011619 | alpha-Amyrin | C[C@@H]1CC[C@]2([C@@H]([C@H]1C)C1=CC[C@H]3[C@@]([C@@]1(CC2)C)(C)CC[C@@H]1[C@]3(C)CC[C@@H](C1(C)C)O)C | 73170 |
| 83 | IMPHY012053 | Sucrose | OC[C@H]1O[C@@]([C@H]([C@@H]1O)O)(CO)O[C@H]1O[C@H](CO)[C@H]([C@@H]([C@H]1O)O)O | 5988 |
| 84 | IMPHY012160 | alpha-Terpineol | CC1=CCC(CC1)C(O)(C)C | 17100 |
| 85 | IMPHY012179 | (2S,4R)-4-methyl-2-(2-methylprop-1-en-1-yl)tetrahydro-2H-pyran | C[C@@H]1CCO[C@@H](C1)C=C(C)C | 1712087 |
| 86 | IMPHY014893 | D-Glucose | OC[C@H]1OC(O)[C@@H]([C@H]([C@@H]1O)O)O | 5793 |
| 87 | IMPHY014916 | D-Fructose | OCC1(O)OC[C@H]([C@H]([C@@H]1O)O)O | 2723872 |
| 88 | IMPHY015047 | Rutin | Oc1cc(O)c2c(c1)oc(c(c2=O)O[C@@H]1O[C@H](CO[C@@H]2O[C@@H](C)[C@@H]([C@H]([C@H]2O)O)O)[C@H]([C@@H]([C@H]1O)O)O)c1ccc(c(c1)O)O | 5280805 |
|  |  |  |  |  |
| PLANT NAME : Saxifraga melanocentra | | | | |
| Serial No | IMPPAT Phytochemical Identifier | Phytochemical Name | SMILES | CID |
| 1 | IMPHY011729 | Mannitol | OC[C@H]([C@H]([C@@H]([C@@H](CO)O)O)O)O | 6251 |
|  |  |  |  |  |
| **PLANT NAME : *Schleichera oleosa*** | | | | |
| **Serial No** | **IMPPAT Phytochemical Identifier** | **Phytochemical Name** | **SMILES** | **CID** |
| 1 | IMPHY011541 | Scopoletin | COc1cc2ccc(=O)oc2cc1O | 5280460 |
| 2 | IMPHY011741 | Tannic acid | O=C(c1cc(O)c(c(c1)OC(=O)c1cc(O)c(c(c1)O)O)O)O[C@@H]1[C@@H](COC(=O)c2cc(O)c(c(c2)OC(=O)c2cc(O)c(c(c2)O)O)O)O[C@H]([C@@H]([C@H]1OC(=O)c1cc(O)c(c(c1)OC(=O)c1cc(O)c(c(c1)O)O)O)OC(=O)c1cc(O)c(c(c1)OC(=O)c1cc(O)c(c(c1)O)O)O)OC(=O)c1cc(O)c(c(c1)OC(=O)c1cc(O)c(c(c1)O)O)O | 16129778 |
| 3 | IMPHY012003 | Betulinic acid | CC(=C)[C@@H]1CC[C@]2([C@H]1[C@H]1CC[C@H]3[C@@]([C@]1(C)CC2)(C)CC[C@@H]1[C@]3(C)CC[C@@H](C1(C)C)O)C(=O)O | 64971 |
| 4 | IMPHY012473 | Lupeol | CC(=C)[C@@H]1CC[C@]2([C@H]1[C@H]1CC[C@H]3[C@@]([C@]1(C)CC2)(C)CC[C@@H]1[C@]3(C)CC[C@@H](C1(C)C)O)C | 259846 |
| 5 | IMPHY014836 | beta-Sitosterol | CC[C@@H](C(C)C)CC[C@H]([C@H]1CC[C@@H]2[C@]1(C)CC[C@H]1[C@H]2CC=C2[C@]1(C)CC[C@@H](C2)O)C | 222284 |
| 6 | IMPHY014991 | Lupeol acetate | CC(=O)O[C@H]1CC[C@]2([C@H](C1(C)C)CC[C@@]1([C@@H]2CC[C@H]2[C@@]1(C)CC[C@@]1([C@@H]2[C@@H](CC1)C(=C)C)C)C)C | 92157 |
| 7 | IMPHY003999 | d-Tartaric acid | O[C@@H]([C@@H](C(=O)O)O)C(=O)O | 439655 |
| 8 | IMPHY006300 | Cholesterol | CC(CCC[C@H]([C@H]1CC[C@@H]2[C@]1(C)CC[C@H]1[C@H]2CC=C2[C@]1(C)CC[C@@H](C2)O)C)C | 5997 |
| 9 | IMPHY007450 | Oxalic acid | OC(=O)C(=O)O | 971 |
| 10 | IMPHY011394 | Arachidic acid | CCCCCCCCCCCCCCCCCCCC(=O)O | 10467 |
| 11 | IMPHY012402 | Campesterol | O[C@H]1CC[C@]2(C(=CC[C@@H]3[C@@H]2CC[C@]2([C@H]3CC[C@@H]2[C@@H](CC[C@H](C(C)C)C)C)C)C1)C | 173183 |
| 12 | IMPHY014836 | beta-Sitosterol | CC[C@@H](C(C)C)CC[C@H]([C@H]1CC[C@@H]2[C@]1(C)CC[C@H]1[C@H]2CC=C2[C@]1(C)CC[C@@H](C2)O)C | 222284 |
| 13 | IMPHY014842 | Stigmasterol | CC[C@@H](C(C)C)/C=C/[C@H]([C@H]1CC[C@@H]2[C@]1(C)CC[C@H]1[C@H]2CC=C2[C@]1(C)CC[C@@H](C2)O)C | 5280794 |
| 14 | IMPHY014893 | D-Glucose | OC[C@H]1OC(O)[C@@H]([C@H]([C@@H]1O)O)O | 5793 |
| 15 | IMPHY014916 | D-Fructose | OCC1(O)OC[C@H]([C@H]([C@@H]1O)O)O | 2723872 |
| 16 | IMPHY004631 | Stearic acid | CCCCCCCCCCCCCCCCCC(=O)O | 5281 |
| 17 | IMPHY007327 | Palmitic acid | CCCCCCCCCCCCCCCC(=O)O | 985 |
| 18 | IMPHY011797 | Oleic acid | CCCCCCCC/C=CCCCCCCCC(=O)O | 445639 |
| 19 | IMPHY012723 | Linolenic acid | CC/C=CC/C=CC/C=CCCCCCCCC(=O)O | 5280934 |
| 20 | IMPHY014990 | Linoleic acid | CCCCC/C=CC/C=CCCCCCCCC(=O)O | 5280450 |
| 21 | IMPHY004271 | Betulin | OC[C@@]12CC[C@H]([C@@H]2[C@@H]2[C@](CC1)(C)[C@]1(C)CC[C@@H]3[C@]([C@H]1CC2)(C)CC[C@@H](C3(C)C)O)C(=C)C | 72326 |
| 22 | IMPHY004631 | Stearic acid | CCCCCCCCCCCCCCCCCC(=O)O | 5281 |
| 23 | IMPHY005590 | Brassicasterol | O[C@H]1CC[C@]2(C(=CC[C@@H]3[C@@H]2CC[C@]2([C@H]3CC[C@@H]2[C@@H](/C=C/[C@@H](C(C)C)C)C)C)C1)C | 5281327 |
| 24 | IMPHY006300 | Cholesterol | CC(CCC[C@H]([C@H]1CC[C@@H]2[C@]1(C)CC[C@H]1[C@H]2CC=C2[C@]1(C)CC[C@@H](C2)O)C)C | 5997 |
| 25 | IMPHY007212 | Docosanoic acid | CCCCCCCCCCCCCCCCCCCCCC(=O)O | 8215 |
| 26 | IMPHY007327 | Palmitic acid | CCCCCCCCCCCCCCCC(=O)O | 985 |
| 27 | IMPHY011394 | Arachidic acid | CCCCCCCCCCCCCCCCCCCC(=O)O | 10467 |
| 28 | IMPHY011666 | Palmitoleic acid | CCCCCC/C=CCCCCCCCC(=O)O | 445638 |
| 29 | IMPHY011797 | Oleic acid | CCCCCCCC/C=CCCCCCCCC(=O)O | 445639 |
| 30 | IMPHY012003 | Betulinic acid | CC(=C)[C@@H]1CC[C@]2([C@H]1[C@H]1CC[C@H]3[C@@]([C@]1(C)CC2)(C)CC[C@@H]1[C@]3(C)CC[C@@H](C1(C)C)O)C(=O)O | 64971 |
| 31 | IMPHY012402 | Campesterol | O[C@H]1CC[C@]2(C(=CC[C@@H]3[C@@H]2CC[C@]2([C@H]3CC[C@@H]2[C@@H](CC[C@H](C(C)C)C)C)C)C1)C | 173183 |
| 32 | IMPHY012761 | Gadoleic acid | CCCCCCCCCC/C=CCCCCCCCC(=O)O | 5282767 |
| 33 | IMPHY014836 | beta-Sitosterol | CC[C@@H](C(C)C)CC[C@H]([C@H]1CC[C@@H]2[C@]1(C)CC[C@H]1[C@H]2CC=C2[C@]1(C)CC[C@@H](C2)O)C | 222284 |
| 34 | IMPHY014842 | Stigmasterol | CC[C@@H](C(C)C)/C=C/[C@H]([C@H]1CC[C@@H]2[C@]1(C)CC[C@H]1[C@H]2CC=C2[C@]1(C)CC[C@@H](C2)O)C | 5280794 |
| 35 | IMPHY014893 | D-Glucose | OC[C@H]1OC(O)[C@@H]([C@H]([C@@H]1O)O)O | 5793 |
| 36 | IMPHY014916 | D-Fructose | OCC1(O)OC[C@H]([C@H]([C@@H]1O)O)O | 2723872 |
|  |  |  |  |  |
| **PLANT NAME : *Sesbania grandiflora*** | | | | |
| **Serial No** | **IMPPAT Phytochemical Identifier** | **Phytochemical Name** | **SMILES** | **CID** |
| 1 | IMPHY011741 | Tannic acid | O=C(c1cc(O)c(c(c1)OC(=O)c1cc(O)c(c(c1)O)O)O)O[C@@H]1[C@@H](COC(=O)c2cc(O)c(c(c2)OC(=O)c2cc(O)c(c(c2)O)O)O)O[C@H]([C@@H]([C@H]1OC(=O)c1cc(O)c(c(c1)OC(=O)c1cc(O)c(c(c1)O)O)O)OC(=O)c1cc(O)c(c(c1)OC(=O)c1cc(O)c(c(c1)O)O)O)OC(=O)c1cc(O)c(c(c1)OC(=O)c1cc(O)c(c(c1)O)O)O | 16129778 |
| 2 | IMPHY011826 | Oleanolic acid | O[C@H]1CC[C@]2([C@H](C1(C)C)CC[C@@]1([C@@H]2CC=C2[C@@]1(C)CC[C@@]1([C@H]2CC(C)(C)CC1)C(=O)O)C)C | 10494 |
| 3 | IMPHY011862 | Nonacosan-6-one | CCCCCCCCCCCCCCCCCCCCCCCC(=O)CCCCC | 86011308 |
| 4 | IMPHY004235 | D-Glucuronic Acid | OC1O[C@H](C(=O)O)[C@H]([C@@H]([C@H]1O)O)O | 94715 |
| 5 | IMPHY011572 | (4aR,6aR,6aS,6bR,8aR,10S,12aR,14bS)-10-hydroxy-2,2,6a,6b,9,9,12a-heptamethyl-1,3,4,5,6,6a,7,8,8a,10,11,12,13,14b-tetradecahydropicene-4a-carboxylic acid | O[C@H]1CC[C@]2([C@H](C1(C)C)CC[C@@]1([C@@H]2CC=C2[C@@]1(C)CC[C@]1([C@H]2CC(C)(C)CC1)C(=O)O)C)C | 45483610 |
| 6 | IMPHY011826 | Oleanolic acid | O[C@H]1CC[C@]2([C@H](C1(C)C)CC[C@@]1([C@@H]2CC=C2[C@@]1(C)CC[C@@]1([C@H]2CC(C)(C)CC1)C(=O)O)C)C | 10494 |
| 7 | IMPHY012050 | D-Galactose | OC[C@H]1OC(O)[C@@H]([C@H]([C@H]1O)O)O | 6036 |
| 8 | IMPHY014632 | 5-Methyl-5-pentacosanol | CCCCCCCCCCCCCCCCCCCCC(CCCC)(O)C | 129650615 |
| 9 | IMPHY015056 | L-Rhamnose | O[C@H]1[C@H](C)OC([C@@H]([C@@H]1O)O)O | 25310 |
| 10 | IMPHY003421 | Galactomannan | OC[C@H]1O[C@H](OC[C@H]2O[C@@H](O)[C@H]([C@H]([C@@H]2O[C@@H]2O[C@H](CO)[C@H]([C@@H]([C@@H]2O)O)O)O)O)[C@@H]([C@H]([C@H]1O)O)O | 439336 |
| 11 | IMPHY011966 | (+)-Leucocyanidin | Oc1cc2O[C@H](c3ccc(c(c3)O)O)[C@H]([C@@H](c2c(c1)O)O)O | 155206 |
| 12 | IMPHY011985 | Nicotiflorin | Oc1ccc(cc1)c1oc2cc(O)cc(c2c(=O)c1O[C@@H]1O[C@H](CO[C@@H]2O[C@@H](C)[C@@H]([C@H]([C@H]2O)O)O)[C@H]([C@@H]([C@H]1O)O)O)O | 5318767 |
| 13 | IMPHY014890 | Cyanidin 3-glucoside | OCC1OC(Oc2cc3c(O)cc(cc3[o+]c2c2ccc(c(c2)O)O)O)C(C(C1O)O)O.[Cl-] | 12303220 |
| 14 | IMPHY000165 | Tetracosanoic acid | CCCCCCCCCCCCCCCCCCCCCCCC(=O)O | 11197 |
| 15 | IMPHY004235 | D-Glucuronic Acid | OC1O[C@H](C(=O)O)[C@H]([C@@H]([C@H]1O)O)O | 94715 |
| 16 | IMPHY004631 | Stearic acid | CCCCCCCCCCCCCCCCCC(=O)O | 5281 |
| 17 | IMPHY007327 | Palmitic acid | CCCCCCCCCCCCCCCC(=O)O | 985 |
| 18 | IMPHY011394 | Arachidic acid | CCCCCCCCCCCCCCCCCCCC(=O)O | 10467 |
| 19 | IMPHY011797 | Oleic acid | CCCCCCCC/C=CCCCCCCCC(=O)O | 445639 |
| 20 | IMPHY011826 | Oleanolic acid | O[C@H]1CC[C@]2([C@H](C1(C)C)CC[C@@]1([C@@H]2CC=C2[C@@]1(C)CC[C@@]1([C@H]2CC(C)(C)CC1)C(=O)O)C)C | 10494 |
| 21 | IMPHY011966 | (+)-Leucocyanidin | Oc1cc2O[C@H](c3ccc(c(c3)O)O)[C@H]([C@@H](c2c(c1)O)O)O | 155206 |
| 22 | IMPHY012050 | D-Galactose | OC[C@H]1OC(O)[C@@H]([C@H]([C@H]1O)O)O | 6036 |
| 23 | IMPHY012723 | Linolenic acid | CC/C=CC/C=CC/C=CCCCCCCCC(=O)O | 5280934 |
| 24 | IMPHY012900 | Kaempferol 3,7-diglucoside | OC[C@H]1O[C@@H](Oc2cc(O)c3c(c2)oc(c(c3=O)O[C@@H]2O[C@H](CO)[C@H]([C@@H]([C@H]2O)O)O)c2ccc(cc2)O)[C@@H]([C@H]([C@@H]1O)O)O | 6325460 |
| 25 | IMPHY014890 | Cyanidin 3-glucoside | OCC1OC(Oc2cc3c(O)cc(cc3[o+]c2c2ccc(c(c2)O)O)O)C(C(C1O)O)O.[Cl-] | 12303220 |
| 26 | IMPHY014990 | Linoleic acid | CCCCC/C=CC/C=CCCCCCCCC(=O)O | 5280450 |
| 27 | IMPHY015056 | L-Rhamnose | O[C@H]1[C@H](C)OC([C@@H]([C@@H]1O)O)O | 25310 |
|  |  |  |  |  |
| **PLANT NAME : *Sophora flavescentis*** | | | | |
| **Serial No** | **Serial No** | **Serial No** | **Serial No** | **Serial No** |
| 1 | IMPHY001876 | Oxymatrine | O=C1CCC[C@H]2N1C[C@@H]1CCC[N+]3([C@@H]1[C@@H]2CCC3)[O-] | 114850 |
| 2 | IMPHY004245 | Sophoridine | O=C1CCC[C@H]2N1C[C@H]1CCCN3[C@@H]1[C@@H]2CCC3 | 165549 |
| 3 | IMPHY004247 | Matrine | O=C1CCC[C@H]2N1C[C@@H]1CCCN3[C@@H]1[C@@H]2CCC3 | 91466 |
| 4 | IMPHY009035 | Formononetin | COc1ccc(cc1)c1coc2c(c1=O)ccc(c2)O | 5280378 |
| 5 | IMPHY010912 | Trifolirhizin | OC[C@H]1O[C@@H](Oc2ccc3c(c2)OC[C@@H]2[C@H]3Oc3c2cc2c(c3)OCO2)[C@@H]([C@H]([C@@H]1O)O)O | 442827 |
| 6 | IMPHY011129 | Sophocarpine | O=C1C=CC[C@H]2N1C[C@@H]1CCCN3[C@@H]1[C@@H]2CCC3 | 115269 |
| 7 | IMPHY012797 | Isokurarinone | COc1cc(O)ccc1C1CC(=O)c2c(O1)c(CC(C(=C)C)CC=C(C)C)c(cc2O)O | 5318581 |
| 8 | IMPHY012878 | 4H-1-Benzopyran-4-one, 2-(4-(beta-D-glucopyranosyloxy)phenyl)-7-hydroxy- | OC[C@H]1O[C@@H](Oc2ccc(cc2)c2cc(=O)c3c(o2)cc(cc3)O)[C@@H]([C@H]([C@@H]1O)O)O | 5491513 |
| 9 | IMPHY014389 | Sophocarpidine | O=C1C=CC[C@H]2N1C[C@@H]1CCC[N@@+]3([C@@H]1[C@@H]2CCC3)[O-] | 24721085 |
| 10 | IMPHY001876 | Oxymatrine | O=C1CCC[C@H]2N1C[C@@H]1CCC[N+]3([C@@H]1[C@@H]2CCC3)[O-] | 114850 |
| 11 | IMPHY002048 | 7,2',4'-Trihydroxy-8-lavandulyl-5-methoxyflavanone | COc1cc(O)c(c2c1C(=O)C[C@H](O2)c1ccc(cc1O)O)C[C@H](C(=C)C)CC=C(C)C | 11982640 |
| 12 | IMPHY004247 | Matrine | O=C1CCC[C@H]2N1C[C@@H]1CCCN3[C@@H]1[C@@H]2CCC3 | 91466 |
| 13 | IMPHY006684 | Isoflavone | O=c1c(coc2c1cccc2)c1ccccc1 | 72304 |
| 14 | IMPHY007660 | (1S,9S,10R,12S)-12-hydroxy-7,15-diazatetracyclo[7.7.1.02,7.010,15]heptadeca-2,4-dien-6-one | O[C@H]1CCN2[C@H](C1)[C@H]1C[C@@H](C2)c2n(C1)c(=O)ccc2 | 131676079 |
| 15 | IMPHY010912 | Trifolirhizin | OC[C@H]1O[C@@H](Oc2ccc3c(c2)OC[C@@H]2[C@H]3Oc3c2cc2c(c3)OCO2)[C@@H]([C@H]([C@@H]1O)O)O | 442827 |
| 16 | IMPHY012797 | Isokurarinone | COc1cc(O)ccc1C1CC(=O)c2c(O1)c(CC(C(=C)C)CC=C(C)C)c(cc2O)O | 5318581 |
| 17 | IMPHY012799 | Kushenol D | COc1cc(O)c(c(c1C(=O)/C=C/c1ccc(cc1OC)O)O)CC(C(=C)C)CC=C(C)C | 5318893 |
| 18 | IMPHY012867 | Kushenol C | CC(=CC[C@@H](C(=C)C)Cc1c(O)cc(c2c1oc(c1ccc(cc1O)O)c(c2=O)O)O)C | 5481237 |
| 19 | IMPHY012976 | Sophoraflavone G | CC(=CCC(C(=C)C)Cc1c(O)cc(c2c1O[C@@H](CC2=O)c1ccc(cc1O)O)O)C | 9910234 |
|  |  |  |  |  |
| **Plant Name:*Steculia villosa*** | | | | |
| **Serial No** | **IMPPAT Phytochemical Identifier** | **Phytochemical Name** | **SMILES** | **CID** |
| 1 | IMPHY005431 | Diosmetin | COc1ccc(cc1O)c1cc(=O)c2c(o1)cc(cc2O)O | 5281612 |
| 2 | IMPHY001097 | Malvalic acid | CCCCCCCCC1=C(C1)CCCCCCC(=O)O | 10416 |
| 3 | IMPHY009570 | Sterculic acid | CCCCCCCCC1=C(C1)CCCCCCCC(=O)O | 12921 |
| 4 | IMPHY000082 | Thermopsoside | OC[C@H]1O[C@@H](Oc2cc(O)c3c(c2)oc(cc3=O)c2ccc(c(c2)OC)O)[C@@H]([C@H]([C@@H]1O)O)O | 11294177 |
| 5 | IMPHY004433 | Chrysoeriol | COc1cc(ccc1O)c1cc(=O)c2c(o1)cc(cc2O)O | 5280666 |
| 6 | IMPHY005431 | Diosmetin | COc1ccc(cc1O)c1cc(=O)c2c(o1)cc(cc2O)O | 5281612 |
|  |  |  |  |  |
| **Plant Name:*Swertia angustifolia*** | | | | |
| **Serial No** | **IMPPAT Phytochemical Identifier** | **Phytochemical Name** | **SMILES** | **CID** |
| 1 | IMPHY006836 | Xanthone | O=c1c2ccccc2oc2c1cccc2 | 7020 |
| 2 | IMPHY011880 | Ursolic acid | C[C@@H]1CC[C@]2([C@@H]([C@H]1C)C1=CC[C@H]3[C@@]([C@@]1(CC2)C)(C)CC[C@@H]1[C@]3(C)CC[C@@H](C1(C)C)O)C(=O)O | 64945 |
| 3 | IMPHY014836 | beta-Sitosterol | CC[C@@H](C(C)C)CC[C@H]([C@H]1CC[C@@H]2[C@]1(C)CC[C@H]1[C@H]2CC=C2[C@]1(C)CC[C@@H](C2)O)C | 222284 |
| 4 | IMPHY001708 | Sweroside | C=C[C@H]1[C@@H](OC=C2[C@H]1CCOC2=O)O[C@@H]1O[C@H](CO)[C@H]([C@@H]([C@H]1O)O)O | 161036 |
| 5 | IMPHY010532 | Swertiamarin | C=C[C@H]1[C@@H](OC=C2[C@@]1(O)CCOC2=O)O[C@@H]1O[C@H](CO)[C@H]([C@@H]([C@H]1O)O)O | 442435 |
| 6 | IMPHY005414 | Bellidifolin | COc1cc(O)c2c(c1)oc1c(c2=O)c(O)ccc1O | 5281623 |
| 7 | IMPHY005458 | Norswertianin | Oc1cc(O)c2c(c1)oc1c(c2=O)c(O)c(cc1)O | 5281658 |
| 8 | IMPHY005616 | 1,3,5,8-Tetrahydroxyxanthone | Oc1cc(O)c2c(c1)oc1c(c2=O)c(O)ccc1O | 5281626 |
| 9 | IMPHY005919 | Decussatin | COc1cc(O)c2c(c1)oc1c(c2=O)c(OC)c(cc1)OC | 5378284 |
| 10 | IMPHY006836 | Xanthone | O=c1c2ccccc2oc2c1cccc2 | 7020 |
| 11 | IMPHY011880 | Ursolic acid | C[C@@H]1CC[C@]2([C@@H]([C@H]1C)C1=CC[C@H]3[C@@]([C@@]1(CC2)C)(C)CC[C@@H]1[C@]3(C)CC[C@@H](C1(C)C)O)C(=O)O | 64945 |
| 12 | IMPHY012794 | 1-Hydroxy-2,3,4,7-tetramethoxyxanthone | COc1ccc2c(c1)c(=O)c1c(o2)c(OC)c(c(c1O)OC)OC | 5318358 |
| 13 | IMPHY013244 | 1,3,8-Trihydroxy-7-methoxyxanthone | COc1ccc2c(c1O)c(=O)c1c(o2)cc(cc1O)O | 14839957 |
| 14 | IMPHY014836 | beta-Sitosterol | CC[C@@H](C(C)C)CC[C@H]([C@H]1CC[C@@H]2[C@]1(C)CC[C@H]1[C@H]2CC=C2[C@]1(C)CC[C@@H](C2)O)C | 222284 |
|  |  |  |  |  |
| **PLANT NAME : *Swertia perennis*** | | | | |
| **Serial No** | **IMPPAT Phytochemical Identifier** | **Phytochemical Name** | **SMILES** | **CID** |
| 1 | IMPHY007355 | Gentiopicroside | C=C[C@H]1[C@@H](OC=C2C1=CCOC2=O)O[C@@H]1O[C@H](CO)[C@H]([C@@H]([C@H]1O)O)O | 88708 |
|  |  |  |  |  |
| **PLANT NAME : *Swietenia macrophylla*** | | | | |
| **Serial No** | **IMPPAT Phytochemical Identifier** | **Phytochemical Name** | **SMILES** | **CID** |
| 1 | IMPHY010726 | Swietenolide | COC(=O)[C@@H]([C@@H]1[C@@]2(C)[C@H]3CC[C@@]4(C(=C3C[C@H](C2=O)[C@H](C1(C)C)O)CC(=O)O[C@H]4c1cocc1)C)O |  |
| 2 | IMPHY010726 | Swietenolide | COC(=O)[C@@H]([C@@H]1[C@@]2(C)[C@H]3CC[C@@]4(C(=C3C[C@H](C2=O)[C@H](C1(C)C)O)CC(=O)O[C@H]4c1cocc1)C)O |  |
| 3 | IMPHY011167 | Swietenolide diacetate | COC(=O)C([C@H]1C(C)(C)C(OC(=O)C)[C@H]2C(=O)[C@]1(C)C1CC[C@@]3(C(=C1C2)CC(=O)O[C@H]3c1ccoc1)C)OC(=O)C | 98042567 |
| 4 | IMPHY011174 | Swietenine | COC(=O)[C@@H]([C@H]1C(C)(C)[C@H](OC(=O)/C(=C/C)/C)[C@H]2C(=O)[C@]1(C)[C@H]1CC[C@@]3([C@H](C1=C2)CC(=O)O[C@H]3c1ccoc1)C)O | 14262276 |
| 5 | IMPHY010726 | Swietenolide | COC(=O)[C@@H]([C@@H]1[C@@]2(C)[C@H]3CC[C@@]4(C(=C3C[C@H](C2=O)[C@H](C1(C)C)O)CC(=O)O[C@H]4c1cocc1)C)O |  |
| 6 | IMPHY011167 | Swietenolide diacetate | COC(=O)C([C@H]1C(C)(C)C(OC(=O)C)[C@H]2C(=O)[C@]1(C)C1CC[C@@]3(C(=C1C2)CC(=O)O[C@H]3c1ccoc1)C)OC(=O)C | 98042567 |
|  |  |  |  |  |
| **PLANT NAME : *Syzygium aromaticum*** | | | | |
| **Serial No** | **IMPPAT Phytochemical Identifier** | **Phytochemical Name** | **SMILES** | **CID** |
| 1 | IMPHY000239 | Eugenin | COc1cc(O)c2c(c1)oc(cc2=O)C | 10189 |
| 2 | IMPHY000399 | beta-Bisabolene | CC(=CCCC(=C)[C@H]1CCC(=CC1)C)C | 10104370 |
| 3 | IMPHY001144 | Dillapiol | C=CCc1cc2OCOc2c(c1OC)OC | 10231 |
| 4 | IMPHY001246 | Carvacrol | CC(c1ccc(c(c1)O)C)C | 10364 |
| 5 | IMPHY001351 | Elemicin | C=CCc1cc(OC)c(c(c1)OC)OC | 10248 |
| 6 | IMPHY001816 | gamma-Terpineol | CC(=C1CCC(CC1)(C)O)C | 11467 |
| 7 | IMPHY001861 | 2-Hexanone | CCCCC(=O)C | 11583 |
| 8 | IMPHY001931 | Vanillin | COc1cc(C=O)ccc1O | 1183 |
| 9 | IMPHY003050 | Methyl salicylate | COC(=O)c1ccccc1O | 4133 |
| 10 | IMPHY003398 | Myristicin | C=CCc1cc(OC)c2c(c1)OCO2 | 4276 |
| 11 | IMPHY003485 | Myrcene | C=CC(=C)CCC=C(C)C | 31253 |
| 12 | IMPHY003536 | Eugenol | C=CCc1ccc(c(c1)OC)O | 3314 |
| 13 | IMPHY003537 | Tetradecanal | CCCCCCCCCCCCCC=O | 31291 |
| 14 | IMPHY003545 | Isopropylbenzaldehyde | O=Cc1ccc(cc1)C(C)C | 326 |
| 15 | IMPHY003982 | gamma-Terpinene | CC1=CCC(=CC1)C(C)C | 7461 |
| 16 | IMPHY004121 | 2-Heptanone | CCCCCC(=O)C | 8051 |
| 17 | IMPHY004184 | Cinnamyl acetate | CC(=O)OC/C=C/c1ccccc1 | 5282110 |
| 18 | IMPHY004438 | Geranyl butyrate | CCCC(=O)OC/C=C(/CCC=C(C)C)C | 5355856 |
| 19 | IMPHY004785 | 2,4,6-trimethoxybenzoyl acetone(eugenone), eugenone | COc1cc(OC)cc(c1C(=O)CC(=O)C)OC | 5317271 |
| 20 | IMPHY004846 | Eugeniin | O=C(c1cc(O)c(c(c1)O)O)O[C@@H]1[C@@H](OC(=O)c2cc(O)c(c(c2)O)O)[C@@H](O[C@H]2[C@H]1OC(=O)c1cc(O)c(c(c1-c1c(C(=O)OC2)cc(c(c1O)O)O)O)O)OC(=O)c1cc(O)c(c(c1)O)O | 442679 |
| 21 | IMPHY005057 | Isoeugenitol | Cc1cc(=O)c2c(o1)c(C)c(cc2O)O | 5318562 |
| 22 | IMPHY005080 | Propyl benzoate | CCCOC(=O)c1ccccc1 | 16846 |
| 23 | IMPHY006145 | p-Cymene | Cc1ccc(cc1)C(C)C | 7463 |
| 24 | IMPHY006279 | 2-Phenylethanol | OCCc1ccccc1 | 6054 |
| 25 | IMPHY006347 | Hexanal | CCCCCC=O | 6184 |
| 26 | IMPHY006550 | Thymol | Cc1ccc(c(c1)O)C(C)C | 6989 |
| 27 | IMPHY006574 | Ethyl benzoate | CCOC(=O)c1ccccc1 | 7165 |
| 28 | IMPHY006696 | IMPHY006696 | C=CCc1ccc(c(c1)OC)OC | 7127 |
| 29 | IMPHY006709 | Acetyleugenol | C=CCc1ccc(c(c1)OC)OC(=O)C | 7136 |
| 30 | IMPHY006946 | (1aR,4S,4aS,7R,7aS,7bR)-1,1,4,7-tetramethyl-2,3,4,5,6,7,7a,7b-octahydro-1aH-cyclopropa[h]azulen-4a-ol | C[C@@H]1CC[C@]2([C@@H]1[C@H]1[C@H](C1(C)C)CC[C@@H]2C)O | 9794494 |
| 31 | IMPHY006948 | beta-Terpineol | CC(=C)C1CCC(CC1)(C)O | 8748 |
| 32 | IMPHY006950 | Tricyclene | CC12C3C1CC(C2(C)C)C3 | 79035 |
| 33 | IMPHY006971 | Methyl palmitate | CCCCCCCCCCCCCCCC(=O)OC | 8181 |
| 34 | IMPHY007039 | Acetophenone | CC(=O)c1ccccc1 | 7410 |
| 35 | IMPHY007048 | Benzyl salicylate | O=C(c1ccccc1O)OCc1ccccc1 | 8363 |
| 36 | IMPHY007069 | Methylacetophenone | Cc1ccc(cc1)C(=O)C | 8500 |
| 37 | IMPHY007201 | Carvacrol methyl ether | COc1cc(ccc1C)C(C)C | 80790 |
| 38 | IMPHY007317 | 4-Allylphenol | C=CCc1ccc(cc1)O | 68148 |
| 39 | IMPHY007520 | Viridiflorene | C[C@@H]1CCC2=C(C)CC[C@@H]3[C@H]([C@H]12)C3(C)C | 10910653 |
| 40 | IMPHY008850 | 2-Hydroxy-4,6-dimethoxy-5-methylacetophenone | COc1c(C)c(OC)cc(c1C(=O)C)O |  |
| 41 | IMPHY008944 | IMPHY008944 | C12C3C4C2=C2C1C3=C42 | 57357909 |
| 42 | IMPHY009946 | Benzaldehyde | O=Cc1ccccc1 | 240 |
| 43 | IMPHY010000 | Dodecane | CCCCCCCCCCCC | 8182 |
| 44 | IMPHY010072 | Eucalyptol | CC12CCC(CC1)C(O2)(C)C | 2758 |
| 45 | IMPHY010097 | Benzyl benzoate | O=C(c1ccccc1)OCc1ccccc1 | 2345 |
| 46 | IMPHY010318 | Geranyl propionate | CCC(=O)OC/C=C(/CCC=C(C)C)C | 5355853 |
| 47 | IMPHY010887 | Thymol acetate | CC(=O)Oc1cc(C)ccc1C(C)C | 68252 |
| 48 | IMPHY011392 | 3-Carene | CC1=CCC2C(C1)C2(C)C | 26049 |
| 49 | IMPHY011396 | 4-Carvomenthenol | **CC1=CCC(CC1)(O)C(C)C** | 11230 |
| 50 | IMPHY011519 | alpha-Terpinyl acetate | CC(=O)OC(C1CCC(=CC1)C)(C)C | 111037 |
| 51 | IMPHY011552 | (1R)-2-methyl-5-propan-2-ylbicyclo[3.1.0]hex-2-ene | CC1=CCC2([C@@H]1C2)C(C)C | 6451618 |
| 52 | IMPHY011586 | (S,1Z,6Z)-8-Isopropyl-1-methyl-5-methylenecyclodeca-1,6-diene | C/C/1=C/CCC(=C)/C=C[C@@H](CC1)C(C)C | 91723653 |
| 53 | IMPHY011590 | d-Borneol | O[C@@H]1C[C@H]2C([C@@]1(C)CC2)(C)C | 61060 |
| 54 | IMPHY011599 | Terpinolene | CC1=CCC(=C(C)C)CC1 | 11463 |
| 55 | IMPHY011632 | Farnesol | OC/C=C(/CC/C=C(/CCC=C(C)C)C)C | 445070 |
| 56 | IMPHY011640 | Isoeugenol | C/C=C/c1ccc(c(c1)OC)O | 853433 |
| 57 | IMPHY011643 | alpha-Terpinene | CC1=CC=C(CC1)C(C)C | 7462 |
| 58 | IMPHY011647 | Geranyl acetate | C/C(=CCOC(=O)C)/CCC=C(C)C | 1549026 |
| 59 | IMPHY011659 | alpha-Muurolene | CC1=C[C@@H]2[C@H](CC1)C(=CC[C@H]2C(C)C)C | 12306047 |
| 60 | IMPHY011740 | Gallotannin | O=C(c1cc(O)c(c(c1)OC(=O)c1cc(O)c(c(c1)O)O)O)O[C@H]1[C@H](COC(=O)c2cc(O)c(c(c2)OC(=O)c2cc(O)c(c(c2)O)O)O)O[C@@H]([C@H]([C@@H]1OC(=O)c1cc(O)c(c(c1)OC(=O)c1cc(O)c(c(c1)O)O)O)OC(=O)c1cc(O)c(c(c1)OC(=O)c1cc(O)c(c(c1)O)O)O)OC(=O)c1cc(O)c(c(c1)OC(=O)c1cc(O)c(c(c1)O)O)O | 16133892 |
| 61 | IMPHY011761 | Humulene | C/C/1=CCC(C)(C)/C=C/C/C(=C/CC1)/C | 5281520 |
| 62 | IMPHY011793 | (+)-gamma-Cadinene | CC1=C[C@@H]2[C@@H](CC1)C(=C)CC[C@H]2C(C)C | 6432404 |
| 63 | IMPHY011794 | (1E,4E,8E)-2,6,6,9-tetramethylcycloundeca-1,4,8-triene | C/C/1=C/CC(C)(C)/C=CC/C(=CCC1)/C | 6508206, |
| 64 | IMPHY011817 | alpha-Farnesene | C=C/C(=C/C/C=C(/CCC=C(C)C)C)/C | 5281516 |
| 65 | IMPHY011826 | Oleanolic acid | O[C@H]1CC[C@]2([C@H](C1(C)C)CC[C@@]1([C@@H]2CC=C2[C@@]1(C)CC[C@@]1([C@H]2CC(C)(C)CC1)C(=O)O)C)C | 10494 |
| 66 | IMPHY011882 | Cinnamaldehyde | O=C/C=C/c1ccccc1 | 637511 |
| 67 | IMPHY011896 | Valencene | CC(=C)[C@@H]1CCC2=CCC[C@H]([C@@]2(C1)C)C | 9855795 |
| 68 | IMPHY011938 | gamma-Eudesmol | CC1=C2C[C@@H](CC[C@]2(CCC1)C)C(O)(C)C | 6432005 |
| 69 | IMPHY011957 | (+)-delta-Cadinene | CC1=C[C@@H]2C(=C(C)CC[C@H]2C(C)C)CC1 | 441005 |
| 70 | IMPHY011965 | (+)-beta-Phellandrene | CC([C@@H]1CCC(=C)C=C1)C | 442484 |
| 71 | IMPHY011970 | Maslinic acid | O[C@@H]1C[C@@]2(C)[C@H](C([C@H]1O)(C)C)CC[C@@]1([C@@H]2CC=C2[C@@]1(C)CC[C@@]1([C@H]2CC(C)(C)CC1)C(=O)O)C | 73659 |
| 72 | IMPHY011983 | cis-Cinnamaldehyde | O=C/C=Cc1ccccc1 | 6428995 |
| 73 | IMPHY011992 | alpha-Asarone | C/C=C/c1cc(OC)c(cc1OC)OC | 636822 |
| 74 | IMPHY012036 | Camphor | O=C1CC2C(C1(C)CC2)(C)C | 2537 |
| 75 | IMPHY012058 | Linalool | C=CC(CCC=C(C)C)(O)C | 6549 |
| 76 | IMPHY012061 | alpha-Pinene | CC1=CCC2CC1C2(C)C | 6654 |
| 77 | IMPHY012147 | beta-Pinene | C=C1CCC2CC1C2(C)C | 14896 |
| 78 | IMPHY012160 | alpha-Terpineol | CC1=CCC(CC1)C(O)(C)C | 17100 |
| 79 | IMPHY012165 | Sabinene | C=C1CCC2(C1C2)C(C)C | 18818 |
| 80 | IMPHY012292 | trans-Sabinol | CC([C@]12CC2C(=C)[C@@H](C1)O)C | 6429076 |
| 81 | IMPHY012585 | delta-Cadinol | CC1=C[C@@H]2[C@H](CC1)[C@](C)(O)CC[C@H]2C(C)C | 3084311 |
| 82 | IMPHY012592 | (+)-Endo-beta-bergamotene | CC(=CCC[C@@]1(C)[C@H]2CCC(=C)[C@@H]1C2)C | 12300073 |
| 83 | IMPHY012667 | Caryophyllene oxide | C=C1CC[C@H]2O[C@@]2(CC[C@@H]2[C@@H]1CC2(C)C)C | 1742210 |
| 84 | IMPHY012738 | Isocaryophyllene | C/C/1=C/CCC(=C)[C@@H]2[C@@H](CC1)C(C2)(C)C | 5281522 |
| 85 | IMPHY012910 | trans-Calamenene | CC([C@H]1CC[C@@H](c2c1cc(C)cc2)C)C | 6429022 |
| 86 | IMPHY013836 | Fenchone | O=C1C2(C)CCC(C1(C)C)C2 | 14525, |
| 87 | IMPHY014512 | beta-Duprezianane | C=C1C[C@]23CC[C@@H]1C([C@@H]3CC[C@H]2C)(C)C | 91750164 |
| 88 | IMPHY014708 | beta-Selinene | C=C1CCC[C@]2([C@H]1C[C@@H](CC2)C(=C)C)C | 442393 |
| 89 | IMPHY014806 | Caswell No. 264AB | CC([C@@H]1CC[C@H]([C@]23[C@H]1[C@H]2C(=CC3)C)C)C | 442359 |
| 90 | IMPHY014809 | alpha-Humulene epoxide | C/C/1=CCC[C@@]2(C)O[C@@H]2CC(/C=C/C1)(C)C | 14038843 |
| 91 | IMPHY014811 | alpha-Phellandrene | CC1=CCC(C=C1)C(C)C | 7460 |
| 92 | IMPHY014831 | beta-Caryophyllene | C/C/1=CCCC(=C)[C@@H]2[C@@H](CC1)C(C2)(C)C | 5281515 |
| 93 | IMPHY014833 | beta-Himachalene | CC1=C[C@H]2C(=C(C)CCCC2(C)C)CC1 | 11586487 |
| 94 | IMPHY014852 | Camphene | C=C1C2CCC(C1(C)C)C2 | 6616 |
| 95 | IMPHY014865 | Calamenene | CC([C@@H]1CC[C@@H](c2c1cc(C)cc2)C)C | 6429077 |
| 96 | IMPHY014874 | cis-Sabinene hydrate | C[C@@H]1CC[C@@]2(C1C2)C(C)C | 101629835 |
| 97 | IMPHY014885 | 1-Isopropyl-4,7-dimethyl-1,3,4,5,6,8a-hexahydro-4a(2H)-naphthalenol | CC1=CC2C(CC1)(O)C(C)CCC2C(C)C | 519857 |
| 98 | IMPHY014988 | Limonene | CC1=CCC(CC1)C(=C)C | 22311 |
| 99 | IMPHY015082 | gamma-Cadinene | CC1=CC2C(CC1)C(=C)CCC2C(C)C | 15094 |
| 100 | IMPHY015123 | alpha-Copaene | CC([C@@H]1CC[C@]2([C@@H]3[C@H]1C2C(=CC3)C)C)C | 70678558 |
| 101 | IMPHY016012 | Allo-Aromadendrene | C[C@@H]1CC[C@H]2[C@@H]1C1C(C1(C)C)CCC2=C | 42608158 |
| 102 | IMPHY016027 | trans-Sabinene hydrate | CC([C@@]12CC[C@](C2C1)(C)O)C | 12315151 |
| 103 | IMPHY016158 | Ethylidene diacetate | CC(OC(=O)C)OC(=O)C | 222536 |
| 104 | IMPHY016247 | Phenol, 2-methoxy(2-propenyl)- | COc1c(CC=C)cccc1O | 596373 |
| 105 | IMPHY017585 | 2-Methoxycinnamaldehyde | O=C/C=C/c1ccccc1OC | 641298 |
| 106 | IMPHY000060 | Myristic acid | CCCCCCCCCCCCCC(=O)O | 11005 |
| 107 | IMPHY000070 | Cadalene | Cc1ccc2c(c1)c(ccc2C)C(C)C | 10225 |
| 108 | IMPHY000239 | Eugenin | COc1cc(O)c2c(c1)oc(cc2=O)C | 10189 |
| 109 | IMPHY000883 | Gemin D | O=CC(C(C1OC(=O)c2cc(O)c(c(c2-c2c(C(=O)OCC1O)cc(c(c2O)O)O)O)O)OC(=O)c1cc(O)c(c(c1)O)O)O | 471119 |
| 110 | IMPHY000944 | Biflorin | OC[C@H]1O[C@H]([C@@H]([C@H]([C@@H]1O)O)O)c1c(O)cc2c(c1O)c(=O)cc(o2)C | 441959 |
| 111 | IMPHY001355 | [(10R,11R,12R,13R,15R)-3,4,5,12,13,21,25,26,27-nonahydroxy-8,18-dioxo-9,14,17,23,30-pentaoxahexacyclo[17.12.0.02,7.010,15.022,31.024,29]hentriaconta-1(31),2,4,6,19,21,24(29),25,27-nonaen-11-yl] 3,4,5- | O[C@H]1[C@H](O)O[C@H]2[C@H]([C@@H]1OC(=O)c1cc(O)c(c(c1)O)O)OC(=O)c1cc(O)c(c(c1-c1c(C(=O)OC2)cc(c2c1Oc1cc(O)c(c(c1O2)O)O)O)O)O | 102445430 |
| 112 | IMPHY001356 | 3,4,5-trihydroxy-2-[[(10R,11S,12R,13S,15R)-3,4,5,21,23-pentahydroxy-8,18-dioxo-11,12,13-tris[(3,4,5-trihydroxybenzoyl)oxy]-9,14,17-trioxatetracyclo[17.4.0.02,7.010,15]tricosa-1(23),2,4,6,19,21-hexaen- | O=C(c1cc(O)c(c(c1)O)O)O[C@H]1[C@@H]2OC(=O)c3cc(O)c(c(c3-c3c(C(=O)OC[C@H]2O[C@H]([C@@H]1OC(=O)c1cc(O)c(c(c1)O)O)OC(=O)c1cc(O)c(c(c1)O)O)cc(c(c3O)Oc1c(cc(c(c1O)O)O)C(=O)O)O)O)O | 102445429 |
| 113 | IMPHY001931 | Vanillin | COc1cc(C=O)ccc1O | 1183 |
| 114 | IMPHY002179 | Humuladienone | C/C/1=C/CC(C)(C)/C=CCC(C(=O)CC1)C | 101297706 |
| 115 | IMPHY002342 | Caryophylla-3(12),6-dien-4-ol | C/C/1=C/CC(O)C(=C)C2C(CC1)C(C2)(C)C |  |
| 116 | IMPHY002419 | Ellagitannin | CC(=O)C[C@]1(O)C(=O)C=C2[C@@H]3[C@@]1(O)Oc1c3c(cc(c1O)O)C(=O)O[C@H]1[C@H]3[C@@H](OC2=O)[C@@H](O[C@@H]1COC(=O)c1c(-c2c(C(=O)O3)cc(O)c(c2O)O)c(O)c(c(c1)O)O)OC(=O)c1cc(O)c(c(c1)O)O | 101601927 |
| 117 | IMPHY002915 | Benzyl Alcohol | OCc1ccccc1 | 244 |
| 118 | IMPHY003050 | Methyl salicylate | COC(=O)c1ccccc1O | 4133 |
| 119 | IMPHY003536 | Eugenol | C=CCc1ccc(c(c1)OC)O | 3314 |
| 120 | IMPHY003977 | (-)-beta-Bourbonene | CC([C@@H]1CC[C@@]2([C@H]1[C@H]1C(=C)CC[C@@H]21)C)C | 62566 |
| 121 | IMPHY004121 | 2-Heptanone | CCCCCC(=O)C | 8051 |
| 122 | IMPHY004846 | Eugeniin | O=C(c1cc(O)c(c(c1)O)O)O[C@@H]1[C@@H](OC(=O)c2cc(O)c(c(c2)O)O)[C@@H](O[C@H]2[C@H]1OC(=O)c1cc(O)c(c(c1-c1c(C(=O)OC2)cc(c(c1O)O)O)O)O)OC(=O)c1cc(O)c(c(c1)O)O | 442679 |
| 123 | IMPHY005057 | Isoeugenitol | Cc1cc(=O)c2c(o1)c(C)c(cc2O)O | 5318562 |
| 124 | IMPHY005644 | Pterocarpin | COc1ccc2c(c1)OC[C@@H]1[C@H]2Oc2c1cc1c(c2)OCO1 | 1715306 |
| 125 | IMPHY006145 | p-Cymene | Cc1ccc(cc1)C(C)C | 7463 |
| 126 | IMPHY006696 | Methyleugenol | C=CCc1ccc(c(c1)OC)OC | 7127 |
| 127 | IMPHY006700 | Methyl benzoate | COC(=O)c1ccccc1 | 7150 |
| 128 | IMPHY006709 | Acetyleugenol | C=CCc1ccc(c(c1)OC)OC(=O)C | 7136 |
| 129 | IMPHY006946 | (1aR,4S,4aS,7R,7aS,7bR)-1,1,4,7-tetramethyl-2,3,4,5,6,7,7a,7b-octahydro-1aH-cyclopropa[h]azulen-4a-ol | C[C@@H]1CC[C@]2([C@@H]1[C@H]1[C@H](C1(C)C)CC[C@@H]2C)O | 9794494 |
| 130 | IMPHY006999 | Strictinin | O[C@H]1[C@@H](O[C@H]2[C@H]([C@@H]1O)OC(=O)c1cc(O)c(c(c1-c1c(C(=O)OC2)cc(c(c1O)O)O)O)O)OC(=O)c1cc(O)c(c(c1)O)O | 73330 |
| 131 | IMPHY007067 | Linalyl acetate | C=CC(OC(=O)C)(CCC=C(C)C)C | 8294 |
| 132 | IMPHY007146 | Methyl acetate | COC(=O)C | 6584 |
| 133 | IMPHY007317 | 4-Allylphenol | C=CCc1ccc(cc1)O | 68148 |
| 134 | IMPHY007417 | Ethyl acetate | CCOC(=O)C | 8857 |
| 135 | IMPHY009182 | Rugosin D | Oc1cc(C(=O)O[C@@H]2O[C@@H]3COC(=O)c4cc(O)c(c(c4-c4c(C(=O)O[C@H]3[C@@H]([C@H]2OC(=O)c2cc(O)c(c(c2)O)O)OC(=O)c2cc(O)c(c(c2)O)O)cc(O)c(c4O)O)O)O)c(c(c1O)O)Oc1cc2C(=O)OC[C@H]3O[C@@H](OC(=O)c4cc(O)c(c(c4)O)O)[C@@H]([C@H]([C@@H]3OC(=O)c3c(-c2c(c1O)O)c(O)c(c(c3)O)O)OC(=O)c1cc(O)c(c(c1)O)O)OC(=O)c1cc(O)c(c(c1)O)O | 16129626 |
| 136 | IMPHY009199 | Rugosin A | O=C(c1cc(O)c(c(c1)O)O)O[C@@H]1O[C@@H]2COC(=O)c3cc(Oc4c(cc(c(c4O)O)O)C(=O)O)c(c(c3-c3c(C(=O)O[C@H]2[C@@H]([C@H]1OC(=O)c1cc(O)c(c(c1)O)O)OC(=O)c1cc(O)c(c(c1)O)O)cc(O)c(c3O)O)O)O | 16132354 |
| 137 | IMPHY009368 | Heptadecane | CCCCCCCCCCCCCCCCC | 12398 |
| 138 | IMPHY009411 | Hexadecyl acetate | CCCCCCCCCCCCCCCCOC(=O)C | 12393 |
| 139 | IMPHY009853 | Naphthalene | c1ccc2c(c1)cccc2 | 931 |
| 140 | IMPHY010072 | Eucalyptol | CC12CCC(CC1)C(O2)(C)C | 2758 |
| 141 | IMPHY011396 | 4-Carvomenthenol | CC1=CCC(CC1)(O)C(C)C | 11230 |
| 142 | IMPHY011581 | alpha-Selinene | CC1=CCC[C@]2([C@H]1C[C@@H](CC2)C(=C)C)C | 10856614 |
| 143 | IMPHY011586 | (S,1Z,6Z)-8-Isopropyl-1-methyl-5-methylenecyclodeca-1,6-diene | C/C/1=C/CCC(=C)/C=C[C@@H](CC1)C(C)C | 91723653 |
| 144 | IMPHY011632 | Farnesol | OC/C=C(/CC/C=C(/CCC=C(C)C)C)C | 445070 |
| 145 | IMPHY011734 | Humulene epoxyde | C/C/1=CCC(C)(C)/C=CCC2(C(CC1)O2)C | 5463721 |
| 146 | IMPHY011749 | Humulene epoxide II | C/C/1=CCC(C)(C)/C=C/C[C@@]2([C@@H](CC1)O2)C | 10704181 |
| 147 | IMPHY011761 | Humulene | C/C/1=CCC(C)(C)/C=C/C/C(=C/CC1)/C | 5281520 |
| 148 | IMPHY011763 | Anethole | C/C=C/c1ccc(cc1)OC | 637563 |
| 149 | IMPHY011793 | (+)-gamma-Cadinene | CC1=C[C@@H]2[C@@H](CC1)C(=C)CC[C@H]2C(C)C | 6432404 |
| 150 | IMPHY011797 | Oleic acid | CCCCCCCC/C=CCCCCCCCC(=O)O | 445639 |
| 151 | IMPHY011883 | 3,4-Dihydroxybenzoic acid | OC(=O)c1ccc(c(c1)O)O | 72 |
| 152 | IMPHY011957 | (+)-delta-Cadinene | CC1=C[C@@H]2C(=C(C)CC[C@H]2C(C)C)CC1 | 441005 |
| 153 | IMPHY012058 | Linalool | C=CC(CCC=C(C)C)(O)C | 6549 |
| 154 | IMPHY012061 | alpha-Pinene | CC1=CCC2CC1C2(C)C | 6654 |
| 155 | IMPHY012075 | Carvone | CC(=C)C1CC=C(C(=O)C1)C | 7439 |
| 156 | IMPHY012093 | Isopropyl myristate | CCCCCCCCCCCCCC(=O)OC(C)C | 8042 |
| 157 | IMPHY012103 | Butyl benzoate | CCCCOC(=O)c1ccccc1 | 8698 |
| 158 | IMPHY012147 | beta-Pinene | C=C1CCC2CC1C2(C)C | 14896 |
| 159 | IMPHY012168 | (1S,2S,6S,7R,8R)-1,3-dimethyl-8-propan-2-yltricyclo[4.4.0.02,7]dec-3-ene | CC([C@H]1CC[C@]2([C@@H]3[C@@H]1[C@H]2C(=CC3)C)C)C | 101607926 |
| 160 | IMPHY012585 | delta-Cadinol | CC1=C[C@@H]2[C@H](CC1)[C@](C)(O)CC[C@H]2C(C)C | 3084311 |
| 161 | IMPHY012586 | (-)-alpha-Cadinol | CC1=CC2C(CC1)[C@@](C)(O)CC[C@@H]2C(C)C | 6431302 |
| 162 | IMPHY012654 | Nerol | OC/C=C(CCC=C(C)C)/C | 643820 |
| 163 | IMPHY012667 | Caryophyllene oxide | C=C1CC[C@H]2O[C@@]2(CC[C@@H]2[C@@H]1CC2(C)C)C | 1742210 |
| 164 | IMPHY013080 | alpha-Calacorene | CC([C@@H]1CC=C(c2c1cc(C)cc2)C)C | 12302243 |
| 165 | IMPHY013286 | Rugosin E | O=C[C@@H]([C@H]([C@@H]1OC(=O)c2cc(O)c(c(c2-c2c(C(=O)OC[C@H]1O)cc(c(c2O)O)Oc1c(cc(c(c1O)O)O)C(=O)O[C@@H]1O[C@@H]2COC(=O)c3cc(O)c(c(c3-c3c(C(=O)O[C@H]2[C@@H]([C@H]1OC(=O)c1cc(O)c(c(c1)O)O)OC(=O)c1cc(O)c(c(c1)O)O)cc(O)c(c3O)O)O)O)O)O)OC(=O)c1cc(O)c(c(c1)O)O)OC(=O)c1cc(O)c(c(c1)O)O | 16129738 |
| 166 | IMPHY014806 | Caswell No. 264AB | CC([C@@H]1CC[C@H]([C@]23[C@H]1[C@H]2C(=CC3)C)C)C | 442359 |
| 167 | IMPHY014831 | beta-Caryophyllene | C/C/1=CCCC(=C)[C@@H]2[C@@H](CC1)C(C2)(C)C | 5281515 |
| 168 | IMPHY014835 | (E)-beta-ocimene | C=C/C(=C/CC=C(C)C)/C | 5281553 |
| 169 | IMPHY014865 | Calamenene | CC([C@@H]1CC[C@@H](c2c1cc(C)cc2)C)C | 6429077 |
| 170 | IMPHY014885 | 1-Isopropyl-4,7-dimethyl-1,3,4,5,6,8a-hexahydro-4a(2H)-naphthalenol | CC1=CC2C(CC1)(O)C(C)CCC2C(C)C | 519857 |
| 171 | IMPHY014906 | Cedrelanol | CC1=C[C@@H]2[C@@H](CC1)[C@@](C)(O)CC[C@H]2C(C)C | 160799 |
| 172 | IMPHY014923 | Geraniol | OC/C=C(/CCC=C(C)C)C | 637566 |
| 173 | IMPHY014986 | Ledol | C[C@@H]1CC[C@H]2[C@@H]1[C@H]1[C@H](C1(C)C)CC[C@@]2(C)O | 92812 |
| 174 | IMPHY014988 | Limonene | CC1=CCC(CC1)C(=C)C | 22311 |
| 175 | IMPHY015022 | Nerolidol | C=CC(CC/C=C(/CCC=C(C)C)C)(O)C | 5284507 |
| 176 | IMPHY015123 | alpha-Copaene | CC([C@@H]1CC[C@]2([C@@H]3[C@H]1C2C(=CC3)C)C)C | 70678558 |
| 177 | IMPHY015128 | T-Muurolol | CC1=C[C@@H]2[C@H](CC1)[C@@](C)(O)CC[C@H]2C(C)C | 3084331 |
| 178 | IMPHY016012 | Allo-Aromadendrene | C[C@@H]1CC[C@H]2[C@@H]1C1C(C1(C)C)CCC2=C | 42608158 |
| 179 | IMPHY000060 | Myristic acid | CCCCCCCCCCCCCC(=O)O | 11005 |
| 180 | IMPHY000239 | Eugenin | COc1cc(O)c2c(c1)oc(cc2=O)C | 10189 |
| 181 | IMPHY001931 | Vanillin | COc1cc(C=O)ccc1O | 1183 |
| 182 | IMPHY003050 | Methyl salicylate | COC(=O)c1ccccc1O | 4133 |
| 183 | IMPHY003536 | Eugenol | C=CCc1ccc(c(c1)OC)O | 3314 |
| 184 | IMPHY004121 | 2-Heptanone | CCCCCC(=O)C | 8051 |
| 185 | IMPHY004846 | Eugeniin | O=C(c1cc(O)c(c(c1)O)O)O[C@@H]1[C@@H](OC(=O)c2cc(O)c(c(c2)O)O)[C@@H](O[C@H]2[C@H]1OC(=O)c1cc(O)c(c(c1-c1c(C(=O)OC2)cc(c(c1O)O)O)O)O)OC(=O)c1cc(O)c(c(c1)O)O | 442679 |
| 186 | IMPHY006145 | p-Cymene | Cc1ccc(cc1)C(C)C | 7463 |
| 187 | IMPHY006574 | Ethyl benzoate | CCOC(=O)c1ccccc1 | 7165 |
| 188 | IMPHY006696 | Methyleugenol | C=CCc1ccc(c(c1)OC)OC | 7127 |
| 189 | IMPHY006700 | Methyl benzoate | COC(=O)c1ccccc1 | 7150 |
| 190 | IMPHY006709 | Acetyleugenol | C=CCc1ccc(c(c1)OC)OC(=O)C | 7136 |
| 191 | IMPHY007041 | Furfural | O=Cc1ccco1 | 7362 |
| 192 | IMPHY007067 | Linalyl acetate | C=CC(OC(=O)C)(CCC=C(C)C)C | 8294 |
| 193 | IMPHY007317 | 4-Allylphenol | C=CCc1ccc(cc1)O | 68148 |
| 194 | IMPHY007327 | Palmitic acid | CCCCCCCCCCCCCCCC(=O)O | 985 |
| 195 | IMPHY008991 | Benzyl acetate | CC(=O)OCc1ccccc1 | 8785 |
| 196 | IMPHY009368 | Heptadecane | CCCCCCCCCCCCCCCCC | 12398 |
| 197 | IMPHY009642 | 2-Nonanone | CCCCCCCC(=O)C | 13187 |
| 198 | IMPHY009853 | Naphthalene | c1ccc2c(c1)cccc2 | 931 |
| 199 | IMPHY011581 | alpha-Selinene | CC1=CCC[C@]2([C@H]1C[C@@H](CC2)C(=C)C)C | 10856614 |
| 200 | IMPHY011586 | (S,1Z,6Z)-8-Isopropyl-1-methyl-5-methylenecyclodeca-1,6-diene | C/C/1=C/CCC(=C)/C=C[C@@H](CC1)C(C)C | 91723653 |
| 201 | IMPHY011734 | Humulene epoxyde | C/C/1=CCC(C)(C)/C=CCC2(C(CC1)O2)C | 5463721 |
| 202 | IMPHY011749 | Humulene epoxide II | C/C/1=CCC(C)(C)/C=C/C[C@@]2([C@@H](CC1)O2)C | 10704181 |
| 203 | IMPHY011761 | Humulene | C/C/1=CCC(C)(C)/C=C/C/C(=C/CC1)/C | 5281520 |
| 204 | IMPHY011763 | Anethole | C/C=C/c1ccc(cc1)OC | 637563 |
| 205 | IMPHY011793 | (+)-gamma-Cadinene | CC1=C[C@@H]2[C@@H](CC1)C(=C)CC[C@H]2C(C)C | 6432404 |
| 206 | IMPHY011797 | Oleic acid | CCCCCCCC/C=CCCCCCCCC(=O)O | 445639 |
| 207 | IMPHY011884 | Pulegone | C[C@@H]1CCC(=C(C)C)C(=O)C1 | 442495 |
| 208 | IMPHY011957 | (+)-delta-Cadinene | CC1=C[C@@H]2C(=C(C)CC[C@H]2C(C)C)CC1 | 441005 |
| 209 | IMPHY011970 | Maslinic acid | O[C@@H]1C[C@@]2(C)[C@H](C([C@H]1O)(C)C)CC[C@@]1([C@@H]2CC=C2[C@@]1(C)CC[C@@]1([C@H]2CC(C)(C)CC1)C(=O)O)C | 73659 |
| 210 | IMPHY012058 | Linalool | C=CC(CCC=C(C)C)(O)C | 6549 |
| 211 | IMPHY012061 | alpha-Pinene | CC1=CCC2CC1C2(C)C | 6654 |
| 212 | IMPHY012075 | Carvone | CC(=C)C1CC=C(C(=O)C1)C | 7439 |
| 213 | IMPHY012093 | Isopropyl myristate | CCCCCCCCCCCCCC(=O)OC(C)C | 8042 |
| 214 | IMPHY012103 | Butyl benzoate | CCCCOC(=O)c1ccccc1 | 8698 |
| 215 | IMPHY012147 | beta-Pinene | C=C1CCC2CC1C2(C)C | 14896 |
| 216 | IMPHY012168 | (1S,2S,6S,7R,8R)-1,3-dimethyl-8-propan-2-yltricyclo[4.4.0.02,7]dec-3-ene | CC([C@H]1CC[C@]2([C@@H]3[C@@H]1[C@H]2C(=CC3)C)C)C | 101607926 |
| 217 | IMPHY012586 | (-)-alpha-Cadinol | CC1=CC2C(CC1)[C@@](C)(O)CC[C@@H]2C(C)C | 6431302 |
| 218 | IMPHY012654 | Nerol | OC/C=C(CCC=C(C)C)/C | 643820 |
| 219 | IMPHY012667 | Caryophyllene oxide | C=C1CC[C@H]2O[C@@]2(CC[C@@H]2[C@@H]1CC2(C)C)C | 1742210 |
| 220 | IMPHY014801 | Zizanene | CC1=C[C@@H]2[C@H](CC1)C(=CC[C@@H]2C(C)C)C | 12306046 |
| 221 | IMPHY014806 | Caswell No. 264AB | CC([C@@H]1CC[C@H]([C@]23[C@H]1[C@H]2C(=CC3)C)C)C | 442359 |
| 222 | IMPHY014831 | beta-Caryophyllene | C/C/1=CCCC(=C)[C@@H]2[C@@H](CC1)C(C2)(C)C | 5281515 |
| 223 | IMPHY014835 | (E)-beta-ocimene | C=C/C(=C/CC=C(C)C)/C | 5281553 |
| 224 | IMPHY014865 | Calamenene | CC([C@@H]1CC[C@@H](c2c1cc(C)cc2)C)C | 6429077 |
| 225 | IMPHY014881 | Copaene | CC(C1CCC2(C3C1C2C(=CC3)C)C)C | 19725 |
| 226 | IMPHY014885 | 1-Isopropyl-4,7-dimethyl-1,3,4,5,6,8a-hexahydro-4a(2H)-naphthalenol | CC1=CC2C(CC1)(O)C(C)CCC2C(C)C | 519857 |
| 227 | IMPHY014906 | Cedrelanol | CC1=C[C@@H]2[C@@H](CC1)[C@@](C)(O)CC[C@H]2C(C)C | 160799 |
| 228 | IMPHY014988 | Limonene | CC1=CCC(CC1)C(=C)C | 22311 |
| 229 | IMPHY015022 | Nerolidol | C=CC(CC/C=C(/CCC=C(C)C)C)(O)C | 5284507 |
| 230 | IMPHY015123 | alpha-Copaene | CC([C@@H]1CC[C@]2([C@@H]3[C@H]1C2C(=CC3)C)C)C | 70678558 |
| 231 | IMPHY015128 | T-Muurolol | CC1=C[C@@H]2[C@H](CC1)[C@@](C)(O)CC[C@H]2C(C)C | 3084331 |
| 232 | IMPHY015374 | 3-(4-Hydroxy-2-methoxyphenyl)-2-propenal | O=C/C=C/c1ccc(cc1OC)O | 5374604 |
| 233 | IMPHY016012 | Allo-Aromadendrene | C[C@@H]1CC[C@H]2[C@@H]1C1C(C1(C)C)CCC2=C | 42608158 |
| 234 | IMPHY017714 | Ethylene glycol dimethacrylate | O=C(C(=C)C)OCCOC(=O)C(=C)C | 7355 |
| 235 | IMPHY003536 | Eugenol | C=CCc1ccc(c(c1)OC)O | 3314 |
| 236 | IMPHY000084 | 2-Heptanol | CCCCCC(O)C | 10976 |
| 237 | IMPHY000239 | Eugenin | COc1cc(O)c2c(c1)oc(cc2=O)C | 10189 |
| 238 | IMPHY000685 | 1-(Furan-2-yl)ethanol | CC(c1ccco1)O | 107243 |
| 239 | IMPHY000865 | [(2R,3R,4S,5R,6R)-2,5-dihydroxy-6-(hydroxymethyl)-3-(3,4,5-trihydroxybenzoyl)oxyoxan-4-yl] 3,4,5-trihydroxybenzoate | OC[C@H]1O[C@@H](O)[C@@H]([C@H]([C@@H]1O)OC(=O)c1cc(O)c(c(c1)O)O)OC(=O)c1cc(O)c(c(c1)O)O | 471118 |
| 240 | IMPHY000883 | Gemin D | O=CC(C(C1OC(=O)c2cc(O)c(c(c2-c2c(C(=O)OCC1O)cc(c(c2O)O)O)O)O)OC(=O)c1cc(O)c(c(c1)O)O)O | 471119 |
| 241 | IMPHY000944 | Biflorin | OC[C@H]1O[C@H]([C@@H]([C@H]([C@@H]1O)O)O)c1c(O)cc2c(c1O)c(=O)cc(o2)C | 441959 |
| 242 | IMPHY001246 | Carvacrol | CC(c1ccc(c(c1)O)C)C | 10364 |
| 243 | IMPHY001861 | 2-Hexanone | CCCCC(=O)C | 11583 |
| 244 | IMPHY002003 | 5-Methylfurfural | Cc1ccc(o1)C=O | 12097 |
| 245 | IMPHY002342 | Caryophylla-3(12),6-dien-4-ol | C/C/1=C/CC(O)C(=C)C2C(CC1)C(C2)(C)C |  |
| 246 | IMPHY002671 | Casuariin | O[C@@H]1COC(=O)c2cc(O)c(c(c2-c2c(C(=O)O[C@H]1[C@@H]1OC(=O)c3cc(O)c(c(c3-c3c4C(=O)O[C@H]1[C@H](O)c4c(O)c(c3O)O)O)O)cc(O)c(c2O)O)O)O | 14035442 |
| 247 | IMPHY002915 | Benzyl Alcohol | OCc1ccccc1 | 244 |
| 248 | IMPHY002934 | 2-Heptanol, benzoate | CCCCCC(OC(=O)c1ccccc1)C | 243678 |
| 249 | IMPHY003536 | Eugenol | C=CCc1ccc(c(c1)OC)O | 3314 |
| 250 | IMPHY003927 | Casuarictin | O=C(c1cc(O)c(c(c1)O)O)O[C@@H]1O[C@@H]2COC(=O)c3cc(O)c(c(c3-c3c(C(=O)O[C@H]2[C@H]2[C@H]1OC(=O)c1cc(O)c(c(c1-c1c(C(=O)O2)cc(c(c1O)O)O)O)O)cc(O)c(c3O)O)O)O | 73644 |
| 251 | IMPHY004121 | 2-Heptanone | CCCCCC(=O)C | 8051 |
| 252 | IMPHY004785 | Eugenone | COc1cc(OC)cc(c1C(=O)CC(=O)C)OC | 5317271 |
| 253 | IMPHY004846 | Eugeniin | O=C(c1cc(O)c(c(c1)O)O)O[C@@H]1[C@@H](OC(=O)c2cc(O)c(c(c2)O)O)[C@@H](O[C@H]2[C@H]1OC(=O)c1cc(O)c(c(c1-c1c(C(=O)OC2)cc(c(c1O)O)O)O)O)OC(=O)c1cc(O)c(c(c1)O)O | 442679 |
| 254 | IMPHY005080 | Propyl benzoate | CCCOC(=O)c1ccccc1 | 16846 |
| 255 | IMPHY006347 | Hexanal | CCCCCC=O | 6184 |
| 256 | IMPHY006658 | Pentagalloylglucose | O=C(c1cc(O)c(c(c1)O)O)O[C@@H]1O[C@H](COC(=O)c2cc(O)c(c(c2)O)O)[C@H]([C@@H]([C@H]1OC(=O)c1cc(O)c(c(c1)O)O)OC(=O)c1cc(O)c(c(c1)O)O)OC(=O)c1cc(O)c(c(c1)O)O | 65238 |
| 257 | IMPHY006696 | Methyleugenol | C=CCc1ccc(c(c1)OC)OC | 7127 |
| 258 | IMPHY006700 | Methyl benzoate | COC(=O)c1ccccc1 | 7150 |
| 259 | IMPHY006709 | Acetyleugenol | C=CCc1ccc(c(c1)OC)OC(=O)C | 7136 |
| 260 | IMPHY006946 | (1aR,4S,4aS,7R,7aS,7bR)-1,1,4,7-tetramethyl-2,3,4,5,6,7,7a,7b-octahydro-1aH-cyclopropa[h]azulen-4a-ol | C[C@@H]1CC[C@]2([C@@H]1[C@H]1[C@H](C1(C)C)CC[C@@H]2C)O | 9794494 |
| 261 | IMPHY006971 | Methyl palmitate | CCCCCCCCCCCCCCCC(=O)OC | 8181 |
| 262 | IMPHY006992 | 1-Methylhexyl acetate | CCCCCC(OC(=O)C)C | 80018 |
| 263 | IMPHY006999 | Strictinin | O[C@H]1[C@@H](O[C@H]2[C@H]([C@@H]1O)OC(=O)c1cc(O)c(c(c1-c1c(C(=O)OC2)cc(c(c1O)O)O)O)O)OC(=O)c1cc(O)c(c(c1)O)O | 73330 |
| 264 | IMPHY007006 | Furfuryl alcohol | OCc1ccco1 | 7361 |
| 265 | IMPHY007039 | Acetophenone | CC(=O)c1ccccc1 | 7410 |
| 266 | IMPHY007041 | Furfural | O=Cc1ccco1 | 7362 |
| 267 | IMPHY007048 | Benzyl salicylate | O=C(c1ccccc1O)OCc1ccccc1 | 8363 |
| 268 | IMPHY007222 | Pentanal | CCCCC=O | 8063 |
| 269 | IMPHY008850 | 2-Hydroxy-4,6-dimethoxy-5-methylacetophenone | COc1c(C)c(OC)cc(c1C(=O)C)O |  |
| 270 | IMPHY009182 | Rugosin D | Oc1cc(C(=O)O[C@@H]2O[C@@H]3COC(=O)c4cc(O)c(c(c4-c4c(C(=O)O[C@H]3[C@@H]([C@H]2OC(=O)c2cc(O)c(c(c2)O)O)OC(=O)c2cc(O)c(c(c2)O)O)cc(O)c(c4O)O)O)O)c(c(c1O)O)Oc1cc2C(=O)OC[C@H]3O[C@@H](OC(=O)c4cc(O)c(c(c4)O)O)[C@@H]([C@H]([C@@H]3OC(=O)c3c(-c2c(c1O)O)c(O)c(c(c3)O)O)OC(=O)c1cc(O)c(c(c1)O)O)OC(=O)c1cc(O)c(c(c1)O)O | 16129626 |
| 271 | IMPHY009199 | Rugosin A | O=C(c1cc(O)c(c(c1)O)O)O[C@@H]1O[C@@H]2COC(=O)c3cc(Oc4c(cc(c(c4O)O)O)C(=O)O)c(c(c3-c3c(C(=O)O[C@H]2[C@@H]([C@H]1OC(=O)c1cc(O)c(c(c1)O)O)OC(=O)c1cc(O)c(c(c1)O)O)cc(O)c(c3O)O)O)O | 16132354 |
| 272 | IMPHY009467 | gamma-Decalactone | CCCCCCC1CCC(=O)O1 | 12813 |
| 273 | IMPHY009626 | 2-Nonanol | CCCCCCCC(O)C | 12367 |
| 274 | IMPHY009642 | 2-Nonanone | CCCCCCCC(=O)C | 13187 |
| 275 | IMPHY009946 | Benzaldehyde | O=Cc1ccccc1 | 240 |
| 276 | IMPHY010596 | 1-Methyloctyl acetate | CCCCCCCC(OC(=O)C)C | 85788 |
| 277 | IMPHY011391 | 1,2,3,6-Tetrakis-O-galloyl-beta-D-glucose | O[C@@H]1[C@@H](COC(=O)c2cc(O)c(c(c2)O)O)O[C@H]([C@@H]([C@H]1OC(=O)c1cc(O)c(c(c1)O)O)OC(=O)c1cc(O)c(c(c1)O)O)OC(=O)c1cc(O)c(c(c1)O)O | 73178 |
| 278 | IMPHY011552 | (1R)-2-methyl-5-propan-2-ylbicyclo[3.1.0]hex-2-ene | CC1=CCC2([C@@H]1C2)C(C)C | 6451618 |
| 279 | IMPHY011761 | Humulene | C/C/1=CCC(C)(C)/C=C/C/C(=C/CC1)/C | 5281520 |
| 280 | IMPHY011792 | gamma-Muurolene | CC1=C[C@@H]2[C@H](CC1)C(=C)CC[C@H]2C(C)C | 12313020 |
| 281 | IMPHY012050 | D-Galactose | OC[C@H]1OC(O)[C@@H]([C@H]([C@H]1O)O)O | 6036 |
| 282 | IMPHY012053 | Sucrose | OC[C@H]1O[C@@]([C@H]([C@@H]1O)O)(CO)O[C@H]1O[C@H](CO)[C@H]([C@@H]([C@H]1O)O)O | 5988 |
| 283 | IMPHY012586 | (-)-alpha-Cadinol | CC1=CC2C(CC1)[C@@](C)(O)CC[C@@H]2C(C)C | 6431302 |
| 284 | IMPHY012630 | Tetracyclo[6.3.2.0(2,5).0(1,8)]tridecan-9-ol, 4,4-dimethyl- | OC1CCC23C1(CCC1C3CC1(C)C)CC2 | 585744 |
| 285 | IMPHY012667 | Caryophyllene oxide | C=C1CC[C@H]2O[C@@]2(CC[C@@H]2[C@@H]1CC2(C)C)C | 1742210 |
| 286 | IMPHY013286 | Rugosin E | O=C[C@@H]([C@H]([C@@H]1OC(=O)c2cc(O)c(c(c2-c2c(C(=O)OC[C@H]1O)cc(c(c2O)O)Oc1c(cc(c(c1O)O)O)C(=O)O[C@@H]1O[C@@H]2COC(=O)c3cc(O)c(c(c3-c3c(C(=O)O[C@H]2[C@@H]([C@H]1OC(=O)c1cc(O)c(c(c1)O)O)OC(=O)c1cc(O)c(c(c1)O)O)cc(O)c(c3O)O)O)O)O)O)OC(=O)c1cc(O)c(c(c1)O)O)OC(=O)c1cc(O)c(c(c1)O)O | 16129738 |
| 287 | IMPHY013836 | Fenchone | O=C1C2(C)CCC(C1(C)C)C2 | 14525 |
| 288 | IMPHY014708 | beta-Selinene | C=C1CCC[C@]2([C@H]1C[C@@H](CC2)C(=C)C)C | 442393 |
| 289 | IMPHY014809 | alpha-Humulene epoxide | C/C/1=CCC[C@@]2(C)O[C@@H]2CC(/C=C/C1)(C)C | 14038843 |
| 290 | IMPHY014831 | beta-Caryophyllene | C/C/1=CCCC(=C)[C@@H]2[C@@H](CC1)C(C2)(C)C | 5281515 |
| 291 | IMPHY014893 | D-Glucose | OC[C@H]1OC(O)[C@@H]([C@H]([C@@H]1O)O)O | 5793 |
| 292 | IMPHY014916 | D-Fructose | OCC1(O)OC[C@H]([C@H]([C@@H]1O)O)O | 2723872 |
| 293 | IMPHY015056 | L-Rhamnose | O[C@H]1[C@H](C)OC([C@@H]([C@@H]1O)O)O | 25310 |
| 294 | IMPHY015116 | D-Xylose | O[C@@H]1COC([C@@H]([C@H]1O)O)O | 135191 |
| 295 | IMPHY015123 | alpha-Copaene | CC([C@@H]1CC[C@]2([C@@H]3[C@H]1C2C(=CC3)C)C)C | 70678558 |
|  |  |  |  |  |
| **PLANT NAME : *Syzygium cumini*** | | | | |
| **Serial No** | **IMPPAT Phytochemical Identifier** | **Phytochemical Name** | **SMILES** | **CID** |
| 1 | IMPHY000239 | Eugenin | COc1cc(O)c2c(c1)oc(cc2=O)C | 10189 |
| 2 | IMPHY001947 | (3S)-O-(N-Methoxy-N-D-glucosylglycyl)betulinic acid | OC[C@H]1OC(N(CC(=O)O[C@H]2CC[C@]3(C(C2(C)C)CC[C@@]2(C3CCC3[C@@]2(C)CC[C@@]2(C3C(CC2)C(=C)C)C(=O)O)C)C)OC)[C@@H]([C@H]([C@@H]1O)O)O | 118701750 |
| 3 | IMPHY004388 | Kaempferol | Oc1ccc(cc1)c1oc2cc(O)cc(c2c(=O)c1O)O | 5280863 |
| 4 | IMPHY004619 | Quercetin | Oc1cc(O)c2c(c1)oc(c(c2=O)O)c1ccc(c(c1)O)O | 5280343 |
| 5 | IMPHY005471 | Myricetin | Oc1cc(O)c2c(c1)oc(c(c2=O)O)c1cc(O)c(c(c1)O)O | 5281672 |
| 6 | IMPHY005537 | Ellagic acid | Oc1cc2c(=O)oc3c4c2c(c1O)oc(=O)c4cc(c3O)O | 5281855 |
| 7 | IMPHY009606 | Ethyl gallate | CCOC(=O)c1cc(O)c(c(c1)O)O | 13250 |
| 8 | IMPHY011359 | 1-(2-Hydroxy-4,6-dimethoxy-3-methylphenyl)ethan-1-one | COc1cc(OC)c(c(c1C(=O)C)O)C | 326186 |
| 9 | IMPHY011363 | 2',6'-Dihydroxy-4'-methoxyacetophenone | COc1cc(O)c(c(c1)O)C(=O)C | 24135 |
| 10 | IMPHY011607 | Bergenin | OC[C@H]1O[C@@H]2[C@@H]([C@H]([C@@H]1O)O)OC(=O)c1c2c(O)c(c(c1)O)OC | 66065 |
| 11 | IMPHY011688 | Friedelin | O=C1CC[C@@H]2[C@]([C@H]1C)(C)CC[C@H]1[C@@]2(C)CC[C@@]2([C@]1(C)CC[C@@]1([C@H]2CC(C)(C)CC1)C)C | 91472 |
| 12 | IMPHY011819 | Friedelanol | O[C@@H]1CC[C@@H]2[C@]([C@H]1C)(C)CC[C@H]1[C@@]2(C)CC[C@@]2([C@]1(C)CC[C@@]1([C@H]2CC(C)(C)CC1)C)C | 101341 |
| 13 | IMPHY011859 | Epifriedelanol | O[C@H]1CC[C@@H]2[C@]([C@H]1C)(C)CC[C@H]1[C@@]2(C)CC[C@@]2([C@]1(C)CC[C@@]1([C@H]2CC(C)(C)CC1)C)C | 119242 |
| 14 | IMPHY012003 | Betulinic acid | CC(=C)[C@@H]1CC[C@]2([C@H]1[C@H]1CC[C@H]3[C@@]([C@]1(C)CC2)(C)CC[C@@H]1[C@]3(C)CC[C@@H](C1(C)C)O)C(=O)O | 64971 |
| 15 | IMPHY012021 | Gallic acid | OC(=O)c1cc(O)c(c(c1)O)O | 370 |
| 16 | IMPHY012053 | Sucrose | OC[C@H]1O[C@@]([C@H]([C@@H]1O)O)(CO)O[C@H]1O[C@H](CO)[C@H]([C@@H]([C@H]1O)O)O | 5988 |
| 17 | IMPHY014824 | Astragalin | OC[C@H]1O[C@@H](Oc2c(oc3c(c2=O)c(O)cc(c3)O)c2ccc(cc2)O)[C@@H]([C@H]([C@@H]1O)O)O | 5282102 |
| 18 | IMPHY014836 | beta-Sitosterol | CC[C@@H](C(C)C)CC[C@H]([C@H]1CC[C@@H]2[C@]1(C)CC[C@H]1[C@H]2CC=C2[C@]1(C)CC[C@@H](C2)O)C | 222284, |
| 19 | IMPHY015071 | stigmast-5-en-3beta-yl beta-D-glucopyranoside | CC[C@@H](C(C)C)CC[C@H]([C@H]1CC[C@@H]2[C@]1(C)CC[C@H]1[C@H]2CC=C2[C@]1(C)CC[C@@H](C2)O[C@@H]1OC(CO)[C@H](C(C1O)O)O)C | 70699351 |
| 20 | IMPHY004388 | Kaempferol | Oc1ccc(cc1)c1oc2cc(O)cc(c2c(=O)c1O)O | 5280863 |
| 21 | IMPHY004619 | Quercetin | Oc1cc(O)c2c(c1)oc(c(c2=O)O)c1ccc(c(c1)O)O | 5280343 |
| 22 | IMPHY005438 | Dihydromyricetin | Oc1cc2O[C@H](c3cc(O)c(c(c3)O)O)[C@H](C(=O)c2c(c1)O)O | 161557 |
| 23 | IMPHY005471 | Myricetin | Oc1cc(O)c2c(c1)oc(c(c2=O)O)c1cc(O)c(c(c1)O)O | 5281672 |
| 24 | IMPHY005537 | Ellagic acid | Oc1cc2c(=O)oc3c4c2c(c1O)oc(=O)c4cc(c3O)O | 5281855 |
| 25 | IMPHY011826 | Oleanolic acid | O[C@H]1CC[C@]2([C@H](C1(C)C)CC[C@@]1([C@@H]2CC=C2[C@@]1(C)CC[C@@]1([C@H]2CC(C)(C)CC1)C(=O)O)C)C | 10494 |
| 26 | IMPHY011970 | Maslinic acid | O[C@@H]1C[C@@]2(C)[C@H](C([C@H]1O)(C)C)CC[C@@]1([C@@H]2CC=C2[C@@]1(C)CC[C@@]1([C@H]2CC(C)(C)CC1)C(=O)O)C | 73659 |
| 27 | IMPHY012721 | Isoquercitrin | OC[C@H]1O[C@@H](Oc2c(oc3c(c2=O)c(O)cc(c3)O)c2ccc(c(c2)O)O)[C@@H]([C@H]([C@@H]1O)O)O | 5280804 |
| 28 | IMPHY012958 | 3-O-Acetyloleanolic acid | CC(=O)O[C@H]1CC[C@]2([C@H](C1(C)C)CC[C@@]1([C@@H]2CC=C2[C@@]1(C)CC[C@@]1([C@H]2CC(C)(C)CC1)C(=O)O)C)C | 151202 |
| 29 | IMPHY000919 | Malvidin 3-laminaribioside | OCC1O[C@@H](Oc2cc3c(O)cc(cc3[o+]c2c2cc(OC)c(c(c2)OC)O)O)C(C([C@@H]1O)O[C@@H]1OC(CO)[C@H](C([C@H]1O)O)O)O | 44256979 |
| 30 | IMPHY001031 | Delphinidin 3-gentiobioside | OCC1O[C@@H](OCC2O[C@@H](Oc3cc4c(O)cc(cc4[o+]c3c3cc(O)c(c(c3)O)O)O)C([C@H]([C@@H]2O)O)O)C([C@H]([C@@H]1O)O)O | 44256919 |
| 31 | IMPHY001032 | Petunidin 3-gentiobioside | OCC1O[C@@H](OCC2O[C@@H](Oc3cc4c(O)cc(cc4[o+]c3c3cc(O)c(c(c3)OC)O)O)C(C([C@H]2O)O)O)C(C([C@@H]1O)O)O | 44256956 |
| 32 | IMPHY002588 | Flavylium | c1ccc(cc1)c1ccc2c([o+]1)cccc2 | 145858 |
| 33 | IMPHY003485 | Myrcene | C=CC(=C)CCC=C(C)C | 31253 |
| 34 | IMPHY003500 | Citric acid | OC(=O)C(CC(=O)O)(CC(=O)O)O | 311 |
| 35 | IMPHY004067 | Dihydrocarvyl acetate | CC(=O)OC1CC(CCC1C)C(=C)C | 30248 |
| 36 | IMPHY004438 | Geranyl butyrate | CCCC(=O)OC/C=C(/CCC=C(C)C)C | 5355856 |
| 37 | IMPHY007327 | Palmitic acid | CCCCCCCCCCCCCCCC(=O)O | 985 |
| 38 | IMPHY007367 | Widdrol | C[C@@]1(O)CC=C2[C@@](CC1)(C)CCCC2(C)C | 94334 |
| 39 | IMPHY007450 | Oxalic acid | OC(=O)C(=O)O | 971 |
| 40 | IMPHY009467 | gamma-Decalactone | CCCCCCC1CCC(=O)O1 | 12813 |
| 41 | IMPHY011396 | 4-Carvomenthenol | CC1=CCC(CC1)(O)C(C)C | 11230 |
| 42 | IMPHY011599 | Terpinolene | CC1=CCC(=C(C)C)CC1 | 11463 |
| 43 | IMPHY011657 | cis-beta-Farnesene | C=CC(=C)CC/C=C(CCC=C(C)C)/C | 5317319 |
| 44 | IMPHY011761 | Humulene | C/C/1=CCC(C)(C)/C=C/C/C(=C/CC1)/C | 5281520 |
| 45 | IMPHY011826 | Oleanolic acid | O[C@H]1CC[C@]2([C@H](C1(C)C)CC[C@@]1([C@@H]2CC=C2[C@@]1(C)CC[C@@]1([C@H]2CC(C)(C)CC1)C(=O)O)C)C | 10494 |
| 46 | IMPHY012021 | Gallic acid | OC(=O)c1cc(O)c(c(c1)O)O | 370 |
| 47 | IMPHY012058 | Linalool | C=CC(CCC=C(C)C)(O)C | 6549 |
| 48 | IMPHY012061 | alpha-Pinene | CC1=CCC2CC1C2(C)C | 6654 |
| 49 | IMPHY012147 | beta-Pinene | C=C1CCC2CC1C2(C)C | 14896 |
| 50 | IMPHY012160 | alpha-Terpineol | CC1=CCC(CC1)C(O)(C)C | 17100 |
| 51 | IMPHY012585 | delta-Cadinol | CC1=C[C@@H]2[C@H](CC1)[C@](C)(O)CC[C@H]2C(C)C | 3084311 |
| 52 | IMPHY012739 | (Z)-beta-Ocimene | C=C/C(=CCC=C(C)C)/C | 5320250 |
| 53 | IMPHY014690 | (-)-Globulol | C[C@@H]1CC[C@@H]2[C@@H]1[C@H]1[C@H](C1(C)C)CC[C@@]2(C)O | 12304985 |
| 54 | IMPHY014831 | beta-Caryophyllene | C/C/1=CCCC(=C)[C@@H]2[C@@H](CC1)C(C2)(C)C | 5281515 |
| 55 | IMPHY014835 | (E)-beta-ocimene | C=C/C(=C/CC=C(C)C)/C | 5281553 |
| 56 | IMPHY014847 | Bornyl acetate | CC(=O)OC1CC2C(C1(C)CC2)(C)C | 6448 |
| 57 | IMPHY014871 | cis-Nerolidol | C=CC(CC/C=C(CCC=C(C)C)/C)(O)C | 5320128 |
| 58 | IMPHY014907 | 6-Epi-beta-bisabolol | CC(=CCC[C@@H]([C@@]1(O)CCC(=CC1)C)C)C | 12300148 |
| 59 | IMPHY014986 | Ledol | C[C@@H]1CC[C@H]2[C@@H]1[C@H]1[C@H](C1(C)C)CC[C@@]2(C)O | 92812 |
| 60 | IMPHY016898 | cis-Dihydrocarvone | CC(=C)[C@H]1CC[C@H](C(=O)C1)C | 443181 |
| 61 | IMPHY000545 | O-Cymene | CC(c1ccccc1C)C | 10703 |
| 62 | IMPHY000711 | Bornylene | CC1(C)C2CCC1(C)C=C2 | 10047 |
| 63 | IMPHY001896 | Heptacosane | CCCCCCCCCCCCCCCCCCCCCCCCCCC | 11636 |
| 64 | IMPHY003482 | 4-Methoxybenzaldehyde | COc1ccc(cc1)C=O | 31244 |
| 65 | IMPHY003485 | Myrcene | C=CC(=C)CCC=C(C)C | 31253 |
| 66 | IMPHY003536 | Eugenol | C=CCc1ccc(c(c1)OC)O | 3314 |
| 67 | IMPHY003545 | 4-Isopropylbenzaldehyde | O=Cc1ccc(cc1)C(C)C | 326 |
| 68 | IMPHY003982 | gamma-Terpinene | CC1=CCC(=CC1)C(C)C | 7461 |
| 69 | IMPHY004549 | Safrole | C=CCc1ccc2c(c1)OCO2 | 5144 |
| 70 | IMPHY005438 | Dihydromyricetin | Oc1cc2O[C@H](c3cc(O)c(c(c3)O)O)[C@H](C(=O)c2c(c1)O)O | 161557 |
| 71 | IMPHY006972 | 1-Octacosanol | CCCCCCCCCCCCCCCCCCCCCCCCCCCCO | 68406 |
| 72 | IMPHY007214 | Octyl acetate | CCCCCCCCOC(=O)C | 8164 |
| 73 | IMPHY007276 | Nonan-1-ol | CCCCCCCCCO | 8914 |
| 74 | IMPHY008910 | Hentriacontane | CCCCCCCCCCCCCCCCCCCCCCCCCCCCCCC | 12410 |
| 75 | IMPHY009413 | Triacontane | CCCCCCCCCCCCCCCCCCCCCCCCCCCCCC | 12535 |
| 76 | IMPHY009482 | Nonacosane | CCCCCCCCCCCCCCCCCCCCCCCCCCCCC | 12409 |
| 77 | IMPHY010072 | Eucalyptol | CC12CCC(CC1)C(O2)(C)C | 2758 |
| 78 | IMPHY010781 | Limonene oxide, cis-(-)- | CC(=C)[C@H]1CC[C@]2([C@@H](C1)O2)C | 6452061 |
| 79 | IMPHY011359 | 1-(2-Hydroxy-4,6-dimethoxy-3-methylphenyl)ethan-1-one | COc1cc(OC)c(c(c1C(=O)C)O)C | 326186 |
| 80 | IMPHY011363 | 2',6'-Dihydroxy-4'-methoxyacetophenone | COc1cc(O)c(c(c1)O)C(=O)C | 24135 |
| 81 | IMPHY011396 | 4-Carvomenthenol | CC1=CCC(CC1)(O)C(C)C | 11230 |
| 82 | IMPHY011552 | (1R)-2-methyl-5-propan-2-ylbicyclo[3.1.0]hex-2-ene | CC1=CCC2([C@@H]1C2)C(C)C | 6451618 |
| 83 | IMPHY011586 | (S,1Z,6Z)-8-Isopropyl-1-methyl-5-methylenecyclodeca-1,6-diene | C/C/1=C/CCC(=C)/C=C[C@@H](CC1)C(C)C | 91723653 |
| 84 | IMPHY011590 | d-Borneol | O[C@@H]1C[C@H]2C([C@@]1(C)CC2)(C)C | 61060 |
| 85 | IMPHY011599 | Terpinolene | CC1=CCC(=C(C)C)CC1 | 11463 |
| 86 | IMPHY011647 | Geranyl acetate | C/C(=CCOC(=O)C)/CCC=C(C)C | 1549026 |
| 87 | IMPHY011648 | Neryl acetate | C/C(=C/COC(=O)C)/CCC=C(C)C | 1549025 |
| 88 | IMPHY011714 | Methyl cinnamate | COC(=O)/C=C/c1ccccc1 | 637520 |
| 89 | IMPHY011745 | Zingiberene | CC(=CCC[C@@H]([C@H]1CC=C(C=C1)C)C)C | 92776 |
| 90 | IMPHY011789 | Citral | O=C/C=C(/CCC=C(C)C)C | 638011 |
| 91 | IMPHY011965 | (+)-beta-Phellandrene | CC([C@@H]1CCC(=C)C=C1)C | 442484 |
| 92 | IMPHY011970 | Maslinic acid | O[C@@H]1C[C@@]2(C)[C@H](C([C@H]1O)(C)C)CC[C@@]1([C@@H]2CC=C2[C@@]1(C)CC[C@@]1([C@H]2CC(C)(C)CC1)C(=O)O)C | 73659 |
| 93 | IMPHY012003 | Betulinic acid | CC(=C)[C@@H]1CC[C@]2([C@H]1[C@H]1CC[C@H]3[C@@]([C@]1(C)CC2)(C)CC[C@@H]1[C@]3(C)CC[C@@H](C1(C)C)O)C(=O)O | 64971 |
| 94 | IMPHY012058 | Linalool | C=CC(CCC=C(C)C)(O)C | 6549 |
| 95 | IMPHY012061 | alpha-Pinene | CC1=CCC2CC1C2(C)C | 6654 |
| 96 | IMPHY012147 | beta-Pinene | C=C1CCC2CC1C2(C)C | 14896 |
| 97 | IMPHY012160 | alpha-Terpineol | CC1=CCC(CC1)C(O)(C)C | 17100 |
| 98 | IMPHY012175 | D-Limonene | CC1=CC[C@@H](CC1)C(=C)C | 440917 |
| 99 | IMPHY012667 | Caryophyllene oxide | C=C1CC[C@H]2O[C@@]2(CC[C@@H]2[C@@H]1CC2(C)C)C | 1742210 |
| 100 | IMPHY012739 | (Z)-beta-Ocimene | C=C/C(=CCC=C(C)C)/C | 5320250 |
| 101 | IMPHY012921 | gamma-Elemene | C=C[C@]1(C)CCC(=C(C)C)C[C@H]1C(=C)C | 6432312 |
| 102 | IMPHY014811 | alpha-Phellandrene | CC1=CCC(C=C1)C(C)C | 7460 |
| 103 | IMPHY014831 | beta-Caryophyllene | C/C/1=CCCC(=C)[C@@H]2[C@@H](CC1)C(C2)(C)C | 5281515 |
| 104 | IMPHY014835 | (E)-beta-ocimene | C=C/C(=C/CC=C(C)C)/C | 5281553 |
| 105 | IMPHY014836 | beta-Sitosterol | CC[C@@H](C(C)C)CC[C@H]([C@H]1CC[C@@H]2[C@]1(C)CC[C@H]1[C@H]2CC=C2[C@]1(C)CC[C@@H](C2)O)C | 222284 |
| 106 | IMPHY014847 | Bornyl acetate | CC(=O)OC1CC2C(C1(C)CC2)(C)C | 6448 |
| 107 | IMPHY014852 | Camphene | C=C1C2CCC(C1(C)C)C2 | 6616 |
| 108 | IMPHY014857 | Cedrol | C[C@@H]1CC[C@@H]2[C@@]31CC[C@@]([C@H](C3)C2(C)C)(C)O | 65575 |
| 109 | IMPHY014923 | Geraniol | OC/C=C(/CCC=C(C)C)C | 637566 |
| 110 | IMPHY015042 | Piperitone | CC1=CC(=O)C(CC1)C(C)C | 6987 |
| 111 | IMPHY004619 | Quercetin | Oc1cc(O)c2c(c1)oc(c(c2=O)O)c1ccc(c(c1)O)O | 5280343 |
| 112 | IMPHY005471 | Myricetin | Oc1cc(O)c2c(c1)oc(c(c2=O)O)c1cc(O)c(c(c1)O)O | 5281672 |
| 113 | IMPHY011851 | Myricetin 3-O-glucoside | OC[C@H]1OC(Oc2c(oc3c(c2=O)c(O)cc(c3)O)c2cc(O)c(c(c2)O)O)[C@@H]([C@H]([C@@H]1O)O)O | 22841567 |
| 114 | IMPHY012721 | Isoquercitrin | OC[C@H]1O[C@@H](Oc2c(oc3c(c2=O)c(O)cc(c3)O)c2ccc(c(c2)O)O)[C@@H]([C@H]([C@@H]1O)O)O | 5280804 |
| 115 | IMPHY012868 | Narcissin | COc1cc(ccc1O)c1oc2cc(O)cc(c2c(=O)c1O[C@@H]1O[C@H](CO[C@@H]2O[C@@H](C)[C@@H]([C@H]([C@H]2O)O)O)[C@H]([C@@H]([C@H]1O)O)O)O | 5481663 |
| 116 | IMPHY000060 | Myristic acid | CCCCCCCCCCCCCC(=O)O | 11005 |
| 117 | IMPHY000761 | Pinoresinol 4-O-beta-D-glucopyranoside | OC[C@H]1O[C@@H](Oc2ccc(cc2OC)[C@H]2OC[C@H]3[C@@H]2CO[C@@H]3c2ccc(c(c2)OC)O)[C@@H]([C@H]([C@@H]1O)O)O | 486614 |
| 118 | IMPHY000896 | 2,3-(S)-hexahydroxydiphenoyl-D-glucose | OCC1OC(O)C2C(C1O)OC(=O)c1cc(O)c(c(c1-c1c(C(=O)O2)cc(O)c(c1O)O)O)O | 492390 |
| 119 | IMPHY001097 | Malvalic acid | CCCCCCCCC1=C(C1)CCCCCCC(=O)O | 10416 |
| 120 | IMPHY001816 | gamma-Terpineol | CC(=C1CCC(CC1)(C)O)C | 11467 |
| 121 | IMPHY003719 | beta-Copaene | CC([C@@H]1CC[C@]2([C@@H]3[C@H]1C2C(=C)CC3)C)C | 57339298 |
| 122 | IMPHY003956 | (+)-gamma-Gurjunene | C[C@@H]1CC[C@H]2C1=C[C@@H](CC[C@H]2C)C(=C)C | 15560285 |
| 123 | IMPHY004281 | Guaiol | C[C@H]1CC[C@H](CC2=C1CC[C@@H]2C)C(O)(C)C | 227829 |
| 124 | IMPHY004619 | Quercetin | Oc1cc(O)c2c(c1)oc(c(c2=O)O)c1ccc(c(c1)O)O | 5280343 |
| 125 | IMPHY004631 | Stearic acid | CCCCCCCCCCCCCCCCCC(=O)O | 5281 |
| 126 | IMPHY002419 | Ellagitannin | CC(=O)C[C@]1(O)C(=O)C=C2[C@@H]3[C@@]1(O)Oc1c3c(cc(c1O)O)C(=O)O[C@H]1[C@H]3[C@@H](OC2=O)[C@@H](O[C@@H]1COC(=O)c1c(-c2c(C(=O)O3)cc(O)c(c2O)O)c(O)c(c(c1)O)O)OC(=O)c1cc(O)c(c(c1)O)O | 101601927 |
| 127 | IMPHY003007 | Dihydrodehydrodiconiferyl alcohol | OCCCc1cc2c(c(c1)OC)O[C@@H]([C@H]2CO)c1ccc(c(c1)OC)O | 384679 |
| 128 | IMPHY003016 | Lauric acid | CCCCCCCCCCCC(=O)O | 3893 |
| 129 | IMPHY003174 | Hydroxymethylfurfural | OCc1ccc(o1)C=O | 237332 |
| 130 | IMPHY003485 | Myrcene | C=CC(=C)CCC=C(C)C | 31253 |
| 131 | IMPHY004665 | Resorcinol | Oc1cccc(c1)O | 5054 |
| 132 | IMPHY005537 | Ellagic acid | Oc1cc2c(=O)oc3c4c2c(c1O)oc(=O)c4cc(c3O)O | 5281855 |
| 133 | IMPHY006026 | 4-Methylbenzaldehyde | O=Cc1ccc(cc1)C | 7725 |
| 134 | IMPHY006145 | p-Cymene | Cc1ccc(cc1)C(C)C | 7463 |
| 135 | IMPHY006558 | 1-Triacontanol | CCCCCCCCCCCCCCCCCCCCCCCCCCCCCCO | 68972 |
| 136 | IMPHY006574 | Ethyl benzoate | CCOC(=O)c1ccccc1 | 7165 |
| 137 | IMPHY006840 | 1,2-Dimethoxybenzene | COc1ccccc1OC | 7043 |
| 138 | IMPHY007327 | Palmitic acid | CCCCCCCCCCCCCCCC(=O)O | 985 |
| 139 | IMPHY007419 | 1,3-Dimethoxybenzene | COc1cccc(c1)OC | 9025 |
| 140 | IMPHY009211 | 2-((3,3-Dimethyloxiran-2-yl)methyl)-3-methylfuran | Cc1ccoc1CC1OC1(C)C | 6428926 |
| 141 | IMPHY009570 | Sterculic acid | CCCCCCCCC1=C(C1)CCCCCCCC(=O)O | 12921 |
| 142 | IMPHY009770 | Caryophyllenyl alcohol | CC1CCC(O)/C(=CCC2C1CC2(C)C)/C | 91704770 |
| 143 | Isoterpinolene | Isoterpinolene | CC1CCC(=C(C)C)C=C1 | 102443 |
| 144 | IMPHY009966 | Eucarvone | CC1=CC=CC(CC1=O)(C)C | 136330 |
| 145 | IMPHY010965 | Corilagin | O[C@@H]1[C@H]2COC(=O)c3cc(O)c(c(c3-c3c(C(=O)O[C@@H]1[C@H]([C@@H](O2)OC(=O)c1cc(O)c(c(c1)O)O)O)cc(O)c(c3O)O)O)O | 73568 |
| 146 | IMPHY011359 | 1-(2-Hydroxy-4,6-dimethoxy-3-methylphenyl)ethan-1-one | COc1cc(OC)c(c(c1C(=O)C)O)C | 326186 |
| 147 | IMPHY011409 | Guaiacol | COc1ccccc1O | 460 |
| 148 | IMPHY011581 | alpha-Selinene | CC1=CCC[C@]2([C@H]1C[C@@H](CC2)C(=C)C)C | 10856614 |
| 149 | IMPHY011586 | (S,1Z,6Z)-8-Isopropyl-1-methyl-5-methylenecyclodeca-1,6-diene | C/C/1=C/CCC(=C)/C=C[C@@H](CC1)C(C)C | 91723653 |
| 150 | IMPHY011659 | alpha-Muurolene | CC1=C[C@@H]2[C@H](CC1)C(=CC[C@H]2C(C)C)C | 12306047 |
| 151 | IMPHY011660 | (+)-alpha-Cadinene | CC1=C[C@@H]2[C@@H](CC1)C(=CC[C@H]2C(C)C)C | 12306048 |
| 152 | IMPHY011724 | (9Z)-(12S,13R)-12,13-Epoxyoctadecenoic acid | CCCCC[C@H]1O[C@H]1C/C=CCCCCCCCC(=O)O | 6449780 |
| 153 | IMPHY011741 | Tannic acid | O=C(c1cc(O)c(c(c1)OC(=O)c1cc(O)c(c(c1)O)O)O)O[C@@H]1[C@@H](COC(=O)c2cc(O)c(c(c2)OC(=O)c2cc(O)c(c(c2)O)O)O)O[C@H]([C@@H]([C@H]1OC(=O)c1cc(O)c(c(c1)OC(=O)c1cc(O)c(c(c1)O)O)O)OC(=O)c1cc(O)c(c(c1)OC(=O)c1cc(O)c(c(c1)O)O)O)OC(=O)c1cc(O)c(c(c1)OC(=O)c1cc(O)c(c(c1)O)O)O | 16129778 |
| 154 | IMPHY011749 | Humulene epoxide II | C/C/1=CCC(C)(C)/C=C/C[C@@]2([C@@H](CC1)O2)C | 10704181 |
| 155 | IMPHY011761 | Humulene | C/C/1=CCC(C)(C)/C=C/C/C(=C/CC1)/C | 5281520 |
| 156 | IMPHY011793 | (+)-gamma-Cadinene | CC1=C[C@@H]2[C@@H](CC1)C(=C)CC[C@H]2C(C)C | 6432404 |
| 157 | IMPHY011797 | Oleic acid | CCCCCCCC/C=CCCCCCCCC(=O)O | 445639 |
| 158 | IMPHY011802 | Ferulic acid | COc1cc(/C=C/C(=O)O)ccc1O | 445858 |
| 159 | IMPHY011933 | Caffeic acid | OC(=O)/C=C/c1ccc(c(c1)O)O | 689043 |
| 160 | IMPHY011957 | (+)-delta-Cadinene | CC1=C[C@@H]2C(=C(C)CC[C@H]2C(C)C)CC1 | 441005 |
| 161 | IMPHY011970 | Maslinic acid | O[C@@H]1C[C@@]2(C)[C@H](C([C@H]1O)(C)C)CC[C@@]1([C@@H]2CC=C2[C@@]1(C)CC[C@@]1([C@H]2CC(C)(C)CC1)C(=O)O)C | 73659 |
| 162 | IMPHY012003 | Betulinic acid | CC(=C)[C@@H]1CC[C@]2([C@H]1[C@H]1CC[C@H]3[C@@]([C@]1(C)CC2)(C)CC[C@@H]1[C@]3(C)CC[C@@H](C1(C)C)O)C(=O)O | 64971 |
| 163 | IMPHY012021 | Gallic acid | OC(=O)c1cc(O)c(c(c1)O)O | 370 |
| 164 | IMPHY012061 | alpha-Pinene | CC1=CCC2CC1C2(C)C | 6654 |
| 165 | IMPHY012088 | Mesitylene | Cc1cc(C)cc(c1)C | 7947 |
| 166 | IMPHY012147 | beta-Pinene | C=C1CCC2CC1C2(C)C | 14896 |
| 167 | IMPHY012667 | Caryophyllene oxide | C=C1CC[C@H]2O[C@@]2(CC[C@@H]2[C@@H]1CC2(C)C)C | 1742210 |
| 168 | IMPHY013080 | alpha-Calacorene | CC([C@@H]1CC=C(c2c1cc(C)cc2)C)C | 12302243 |
| 169 | IMPHY013574 | 14-Hydroxy-9-epi-(E)-caryophyllene | OCC1(C)CC2C1CC/C(=CCCC2=C)/C | 5352484 |
| 170 | IMPHY013971 | epi-Cubenol | CC1=C[C@@H]2[C@@](CC1)(O)[C@H](C)CC[C@H]2C(C)C | 12046149 |
| 171 | IMPHY014690 | (-)-Globulol | C[C@@H]1CC[C@@H]2[C@@H]1[C@H]1[C@H](C1(C)C)CC[C@@]2(C)O | 12304985 |
| 172 | IMPHY014785 | (E)-2-epi-beta-caryophyllene | C/C/1=CCCC(=C)[C@H]2[C@@H](CC1)C(C2)(C)C | 6429274 |
| 173 | IMPHY014817 | Aromadendrene | CC1CCC2C1C1C(C1(C)C)CCC2=C | 91354 |
| 174 | IMPHY014836 | beta-Sitosterol | CC[C@@H](C(C)C)CC[C@H]([C@H]1CC[C@@H]2[C@]1(C)CC[C@H]1[C@H]2CC=C2[C@]1(C)CC[C@@H](C2)O)C | 222284 |
| 175 | IMPHY014852 | Camphene | C=C1C2CCC(C1(C)C)C2 | 6616 |
| 176 | IMPHY014988 | Limonene | CC1=CCC(CC1)C(=C)C | 22311 |
| 177 | IMPHY014990 | Linoleic acid | CCCCC/C=CC/C=CCCCCCCCC(=O)O | 5280450 |
| 178 | IMPHY015016 | alpha-Muurolol | CC1=C[C@@H]2[C@H](CC1)[C@](C)(O)CC[C@@H]2C(C)C | 91753440 |
| 179 | IMPHY015123 | alpha-Copaene | CC([C@@H]1CC[C@]2([C@@H]3[C@H]1C2C(=CC3)C)C)C | 70678558 |
| 180 | IMPHY015168 | 14-Hydroxy-alpha-humulene | OCC1(C)/C=C/C/C(=C/CC/C(=C/C1)/C)/C | 5352485 |
| 181 | IMPHY016012 | Allo-Aromadendrene | C[C@@H]1CC[C@H]2[C@@H]1C1C(C1(C)C)CCC2=C | 42608158 |
| 182 | IMPHY017920 | 14-Hydroxy-z-caryophyllene | OCC1CCCC(C2C(/C=C/1)C(C2)(C)C)C |  |
| 183 | IMPHY012061 | alpha-Pinene | CC1=CCC2CC1C2(C)C | 6654 |
| 184 | IMPHY012147 | beta-Pinene | C=C1CCC2CC1C2(C)C | 14896 |
| 185 | IMPHY014847 | Bornyl acetate | CC(=O)OC1CC2C(C1(C)CC2)(C)C | 6448 |
| 186 | IMPHY000711 | Bornylene | CC1(C)C2CCC1(C)C=C2 | 10047 |
| 187 | IMPHY000919 | Malvidin 3-laminaribioside | OCC1O[C@@H](Oc2cc3c(O)cc(cc3[o+]c2c2cc(OC)c(c(c2)OC)O)O)C(C([C@@H]1O)O[C@@H]1OC(CO)[C@H](C([C@H]1O)O)O)O | 44256979 |
| 188 | IMPHY001031 | Delphinidin 3-gentiobioside | OCC1O[C@@H](OCC2O[C@@H](Oc3cc4c(O)cc(cc4[o+]c3c3cc(O)c(c(c3)O)O)O)C([C@H]([C@@H]2O)O)O)C([C@H]([C@@H]1O)O)O | 44256919 |
| 189 | IMPHY001032 | Petunidin 3-gentiobioside | OCC1O[C@@H](OCC2O[C@@H](Oc3cc4c(O)cc(cc4[o+]c3c3cc(O)c(c(c3)OC)O)O)C(C([C@H]2O)O)O)C(C([C@@H]1O)O)O | 44256956 |
| 190 | IMPHY001896 | Heptacosane | CCCCCCCCCCCCCCCCCCCCCCCCCCC | 11636 |
| 191 | IMPHY003016 | Lauric acid | CCCCCCCCCCCC(=O)O | 3893 |
| 192 | IMPHY003485 | Myrcene | C=CC(=C)CCC=C(C)C | 31253 |
| 193 | IMPHY003500 | Citric acid | OC(=O)C(CC(=O)O)(CC(=O)O)O | 311 |
| 194 | IMPHY003536 | Eugenol | C=CCc1ccc(c(c1)OC)O | 3314 |
| 195 | IMPHY003545 | 4-Isopropylbenzaldehyde | O=Cc1ccc(cc1)C(C)C | 326 |
| 196 | IMPHY004619 | Quercetin | Oc1cc(O)c2c(c1)oc(c(c2=O)O)c1ccc(c(c1)O)O | 5280343 |
| 197 | IMPHY004631 | Stearic acid | CCCCCCCCCCCCCCCCCC(=O)O | 5281 |
| 198 | IMPHY005263 | 1-Tricosanol | CCCCCCCCCCCCCCCCCCCCCCCO | 18431 |
| 199 | IMPHY005438 | Dihydromyricetin | Oc1cc2O[C@H](c3cc(O)c(c(c3)O)O)[C@H](C(=O)c2c(c1)O)O | 161557 |
| 200 | IMPHY005537 | Ellagic acid | Oc1cc2c(=O)oc3c4c2c(c1O)oc(=O)c4cc(c3O)O | 5281855 |
| 201 | IMPHY005888 | 3,4'-Di-O-methylellagic acid | COc1cc2c(=O)oc3c4c2c(c1O)oc(=O)c4cc(c3OC)O | 5491816 |
| 202 | IMPHY006174 | Glycolic acid | OCC(=O)O | 757 |
| 203 | IMPHY006310 | Folic | OC(=O)CC[C@@H](C(=O)O)NC(=O)c1ccc(cc1)NCc1cnc2c(n1)c(=O)nc([nH]2)N | 6037 |
| 204 | IMPHY006362 | Ascorbic acid | OC[C@@H]([C@H]1OC(=O)C(=C1O)O)O | 54670067 |
| 205 | IMPHY006558 | 1-Triacontanol | CCCCCCCCCCCCCCCCCCCCCCCCCCCCCCO | 68972 |
| 206 | IMPHY006840 | 1,2-Dimethoxybenzene | COc1ccccc1OC | 7043 |
| 207 | IMPHY006972 | 1-Octacosanol | CCCCCCCCCCCCCCCCCCCCCCCCCCCCO | 68406 |
| 208 | IMPHY007357 | Nicotinic acid | OC(=O)c1cccnc1 | 938 |
| 209 | IMPHY007419 | 1,3-Dimethoxybenzene | COc1cccc(c1)OC | 9025 |
| 210 | IMPHY007450 | Oxalic acid | OC(=O)C(=O)O | 971 |
| 211 | IMPHY008910 | Hentriacontane | CCCCCCCCCCCCCCCCCCCCCCCCCCCCCCC | 12410 |
| 212 | IMPHY009413 | Triacontane | CCCCCCCCCCCCCCCCCCCCCCCCCCCCCC | 12535 |
| 213 | IMPHY009482 | Nonacosane | CCCCCCCCCCCCCCCCCCCCCCCCCCCCC | 12409 |
| 214 | IMPHY009570 | Sterculic acid | CCCCCCCCC1=C(C1)CCCCCCCC(=O)O | 12921 |
| 215 | IMPHY010965 | Corilagin | O[C@@H]1[C@H]2COC(=O)c3cc(O)c(c(c3-c3c(C(=O)O[C@@H]1[C@H]([C@@H](O2)OC(=O)c1cc(O)c(c(c1)O)O)O)cc(O)c(c3O)O)O)O | 73568 |
| 216 | IMPHY011359 | 1-(2-Hydroxy-4,6-dimethoxy-3-methylphenyl)ethan-1-one | COc1cc(OC)c(c(c1C(=O)C)O)C | 326186 |
| 217 | IMPHY011363 | 2',6'-Dihydroxy-4'-methoxyacetophenone | COc1cc(O)c(c(c1)O)C(=O)C | 24135 |
| 218 | IMPHY011409 | Guaiacol | COc1ccccc1O | 460 |
| 219 | IMPHY011590 | d-Borneol | O[C@@H]1C[C@H]2C([C@@]1(C)CC2)(C)C | 61060 |
| 220 | IMPHY011599 | Terpinolene | CC1=CCC(=C(C)C)CC1 | 11463 |
| 221 | IMPHY011643 | alpha-Terpinene | CC1=CC=C(CC1)C(C)C | 7462 |
| 222 | IMPHY011688 | Friedelin | O=C1CC[C@@H]2[C@]([C@H]1C)(C)CC[C@H]1[C@@]2(C)CC[C@@]2([C@]1(C)CC[C@@]1([C@H]2CC(C)(C)CC1)C)C | 91472 |
| 223 | IMPHY011707 | beta-Carotene | C/C(=CC=CC=C(C=CC=C(C=CC1=C(C)CCCC1(C)C)/C)/C)/C=C/C=C(/C=C/C1=C(C)CCCC1(C)C)C | 5280489 |
| 224 | IMPHY011724 | (9Z)-(12S,13R)-12,13-Epoxyoctadecenoic acid | CCCCC[C@H]1O[C@H]1C/C=CCCCCCCCC(=O)O | 6449780 |
| 225 | IMPHY011797 | Oleic acid | CCCCCCCC/C=CCCCCCCCC(=O)O | 445639 |
| 226 | IMPHY011802 | Ferulic acid | COc1cc(/C=C/C(=O)O)ccc1O | 445858 |
| 227 | IMPHY011933 | Caffeic acid | OC(=O)/C=C/c1ccc(c(c1)O)O | 689043 |
| 228 | IMPHY011965 | (+)-beta-Phellandrene | CC([C@@H]1CCC(=C)C=C1)C | 442484 |
| 229 | IMPHY011970 | Maslinic acid | O[C@@H]1C[C@@]2(C)[C@H](C([C@H]1O)(C)C)CC[C@@]1([C@@H]2CC=C2[C@@]1(C)CC[C@@]1([C@H]2CC(C)(C)CC1)C(=O)O)C | 73659 |
| 230 | IMPHY012003 | Betulinic acid | CC(=C)[C@@H]1CC[C@]2([C@H]1[C@H]1CC[C@H]3[C@@]([C@]1(C)CC2)(C)CC[C@@H]1[C@]3(C)CC[C@@H](C1(C)C)O)C(=O)O | 64971 |
| 231 | IMPHY012021 | Gallic acid | OC(=O)c1cc(O)c(c(c1)O)O | 370 |
| 232 | IMPHY012053 | Sucrose | OC[C@H]1O[C@@]([C@H]([C@@H]1O)O)(CO)O[C@H]1O[C@H](CO)[C@H]([C@@H]([C@H]1O)O)O | 5988 |
| 233 | IMPHY012061 | alpha-Pinene | CC1=CCC2CC1C2(C)C | 6654 |
| 234 | IMPHY012147 | beta-Pinene | C=C1CCC2CC1C2(C)C | 14896 |
| 235 | IMPHY012160 | alpha-Terpineol | CC1=CCC(CC1)C(O)(C)C | 17100 |
| 236 | IMPHY012868 | Narcissin | COc1cc(ccc1O)c1oc2cc(O)cc(c2c(=O)c1O[C@@H]1O[C@H](CO[C@@H]2O[C@@H](C)[C@@H]([C@H]([C@H]2O)O)O)[C@H]([C@@H]([C@H]1O)O)O)O | 5481663 |
| 237 | IMPHY014824 | Astragalin | OC[C@H]1O[C@@H](Oc2c(oc3c(c2=O)c(O)cc(c3)O)c2ccc(cc2)O)[C@@H]([C@H]([C@@H]1O)O)O | 5282102 |
| 238 | IMPHY014847 | Bornyl acetate | CC(=O)OC1CC2C(C1(C)CC2)(C)C | 6448 |
| 239 | IMPHY014893 | D-Glucose | OC[C@H]1OC(O)[C@@H]([C@H]([C@@H]1O)O)O | 5793 |
| 240 | IMPHY014916 | D-Fructose | OCC1(O)OC[C@H]([C@H]([C@@H]1O)O)O | 2723872 |
| 241 | IMPHY014979 | (-)-Limonene | CC1=CC[C@H](CC1)C(=C)C | 439250 |
|  |  |  |  |  |
| **PLANT NAME : *Tamarindus indica*** | | | | |
| **Serial No** | **IMPPAT Phytochemical Identifier** | **Phytochemical Name** | **SMILES** | **CID** |
| 1 | IMPHY007130 | Hordenine | CN(CCc1ccc(cc1)O)C | 68313 |
| 2 | IMPHY007130 | Hordenine | CN(CCc1ccc(cc1)O)C | 68313 |
| 3 | IMPHY000037 | 3-Methyl-2-buten-1-OL | OCC=C(C)C | 11173 |
| 4 | IMPHY000042 | 3-Methyl-2-butanone | CC(=O)C(C)C | 11251 |
| 5 | IMPHY000060 | Myristic acid | CCCCCCCCCCCCCC(=O)O | 11005 |
| 6 | IMPHY000120 | Heptadecanoic acid | CCCCCCCCCCCCCCCCC(=O)O | 10465 |
| 7 | IMPHY000550 | 2-Methylfuran | Cc1ccco1 | 10797 |
| 8 | IMPHY000795 | Octanal | CCCCCCCC=O | 454 |
| 9 | IMPHY000846 | Riboflavin | OC[C@H]([C@H]([C@H](Cn1c2-c(nc3c1cc(C)c(c3)C)c(=O)[nH]c(=O)n2)O)O)O | 493570 |
| 10 | IMPHY001223 | 2,3-Dihydrobenzofuran | c1ccc2c(c1)OCC2 | 10329 |
| 11 | IMPHY001246 | Carvacrol | CC(c1ccc(c(c1)O)C)C | 10364 |
| 12 | IMPHY001548 | Geranylacetone | C/C(=CCCC(=O)C)/CCC=C(C)C | 1549778 |
| 13 | IMPHY001577 | Ethyl vinyl ketone | CCC(=O)C=C | 15394 |
| 14 | IMPHY001854 | 3-Methylbutanal | O=CCC(C)C | 11552 |
| 15 | IMPHY001915 | Octadecane | CCCCCCCCCCCCCCCCCC | 11635 |
| 16 | IMPHY001931 | Vanillin | COc1cc(C=O)ccc1O | 1183 |
| 17 | IMPHY001956 | Methyl 2-furoate | COC(=O)c1ccco1 | 11902 |
| 18 | IMPHY001971 | 1-Penten-3-OL | CCC(C=C)O | 12020 |
| 19 | IMPHY002667 | Pentadecanoic acid | CCCCCCCCCCCCCCC(=O)O | 13849 |
| 20 | IMPHY002750 | 2-Acetylfuran | CC(=O)c1ccco1 | 14505 |
| 21 | IMPHY002915 | Benzyl Alcohol | OCc1ccccc1 | 244 |
| 22 | IMPHY002962 | Benzoic acid | OC(=O)c1ccccc1 | 243 |
| 23 | IMPHY003016 | Lauric acid | CCCCCCCCCCCC(=O)O | 3893 |
| 24 | IMPHY003050 | Methyl salicylate | COC(=O)c1ccccc1O | 4133 |
| 25 | IMPHY003104 | Decanoic acid | CCCCCCCCCC(=O)O | 2969 |
| 26 | IMPHY003301 | Octanoic acid | CCCCCCCC(=O)O | 379 |
| 27 | IMPHY003340 | Acetoin | CC(=O)C(O)C | 179 |
| 28 | IMPHY003485 | Myrcene | C=CC(=C)CCC=C(C)C | 31253 |
| 29 | IMPHY003495 | 2-Methoxy-4-vinylphenol | COc1cc(C=C)ccc1O | 332 |
| 30 | IMPHY003499 | Pyrrolidine | C1CCCN1 | 31268 |
| 31 | IMPHY003500 | Citric acid | OC(=O)C(CC(=O)O)(CC(=O)O)O | 311 |
| 32 | IMPHY003513 | Isoamyl alcohol | OCCC(C)C | 31260 |
| 33 | IMPHY003525 | Nonanal | CCCCCCCCC=O | 31289 |
| 34 | IMPHY003536 | Eugenol | C=CCc1ccc(c(c1)OC)O | 3314 |
| 35 | IMPHY003915 | 2-Decenal | CCCCCCC/C=C/C=O | 5283345 |
| 36 | IMPHY003982 | gamma-Terpinene | CC1=CCC(=CC1)C(C)C | 7461 |
| 37 | IMPHY003999 | d-Tartaric acid | O[C@@H]([C@@H](C(=O)O)O)C(=O)O | 439655 |
| 38 | IMPHY004631 | Stearic acid | CCCCCCCCCCCCCCCCCC(=O)O | 5281 |
| 39 | IMPHY004751 | 3-(Methylthio)propionaldehyde | CSCCC=O | 18635 |
| 40 | IMPHY005257 | 2-Ethylfuran | CCc1ccco1 | 18554 |
| 41 | IMPHY005526 | 2-Undecenal | CCCCCCCC/C=C/C=O | 5283356 |
| 42 | IMPHY005551 | 2,4-Heptadienal | CC/C=C/C=C/C=O | 5283321 |
| 43 | IMPHY005670 | 2,3-Dimethylpyrazine | Cc1nccnc1C | 22201 |
| 44 | IMPHY005935 | trans-2-Pentenal | CC/C=C/C=O | 5364752 |
| 45 | IMPHY006026 | 4-Methylbenzaldehyde | O=Cc1ccc(cc1)C | 7725 |
| 46 | IMPHY006037 | 2-Methylbutyraldehyde | CC(C=O)CC | 7284 |
| 47 | IMPHY006039 | 1,2,4-Trimethylbenzene | Cc1ccc(c(c1)C)C | 7247 |
| 48 | IMPHY006145 | p-Cymene | Cc1ccc(cc1)C(C)C | 7463 |
| 49 | IMPHY006279 | 2-Phenylethanol | OCCc1ccccc1 | 6054 |
| 50 | IMPHY006322 | Safranal | O=CC1=C(C)C=CCC1(C)C | 61041 |
| 51 | IMPHY006327 | Lactic acid | OC(=O)C(O)C | 612 |
| 52 | IMPHY006347 | Hexanal | CCCCCC=O | 6184 |
| 53 | IMPHY006362 | Ascorbic acid | OC[C@@H]([C@H]1OC(=O)C(=C1O)O)O | 54670067 |
| 54 | IMPHY006404 | Damascenone | C/C=C/C(=O)C1=C(C)C=CCC1(C)C | 5366074 |
| 55 | IMPHY006499 | Vitispirane | CC1CCC2(O1)C(=C)C=CCC2(C)C | 6450832 |
| 56 | IMPHY006700 | Methyl benzoate | COC(=O)c1ccccc1 | 7150 |
| 57 | IMPHY006934 | 2-Methyl-1-butanol | CCC(CO)C | 8723 |
| 58 | IMPHY006971 | Methyl palmitate | CCCCCCCCCCCCCCCC(=O)OC | 8181 |
| 59 | IMPHY006975 | 3,5-Dimethylphenol | Cc1cc(C)cc(c1)O | 7948 |
| 60 | IMPHY006981 | Indole | c1ccc2c(c1)[nH]cc2 | 798 |
| 61 | IMPHY007006 | Furfuryl alcohol | OCc1ccco1 | 7361 |
| 62 | IMPHY007039 | Acetophenone | CC(=O)c1ccccc1 | 7410 |
| 63 | IMPHY007041 | Furfural | O=Cc1ccco1 | 7362 |
| 64 | IMPHY007045 | 2-Methylbutanoic acid | CC(C(=O)O)CC | 8314 |
| 65 | IMPHY007109 | 2,3-Butanedione | CC(=O)C(=O)C | 650 |
| 66 | IMPHY007162 | 2-Methyl-3-buten-2-OL | C=CC(O)(C)C | 8257 |
| 67 | IMPHY007186 | Heptanal | CCCCCCC=O | 8130 |
| 68 | IMPHY007216 | Pyrrole | c1ccc[nH]1 | 8027 |
| 69 | IMPHY007221 | Methyl decanoate | CCCCCCCCCC(=O)OC | 8050 |
| 70 | IMPHY007327 | Palmitic acid | CCCCCCCCCCCCCCCC(=O)O | 985 |
| 71 | IMPHY007331 | 6-Methyl-5-hepten-2-one | CC(=O)CCC=C(C)C | 9862 |
| 72 | IMPHY007354 | Hexanoic acid | CCCCCC(=O)O | 8892 |
| 73 | IMPHY007357 | Nicotinic acid | OC(=O)c1cccnc1 | 938 |
| 74 | IMPHY007417 | Ethyl acetate | CCOC(=O)C | 8857 |
| 75 | IMPHY007450 | Oxalic acid | OC(=O)C(=O)O | 971 |
| 76 | IMPHY007539 | Phenylacetaldehyde | O=CCc1ccccc1 | 998 |
| 77 | IMPHY007620 | 1-Octanol | CCCCCCCCO | 957 |
| 78 | IMPHY009823 | gamma-Butyrolactone | O=C1CCCO1 | 7302 |
| 79 | IMPHY009946 | Benzaldehyde | O=Cc1ccccc1 | 240 |
| 80 | IMPHY009977 | Cyclohexanol | OC1CCCCC1 | 7966 |
| 81 | IMPHY009980 | Ethylbenzene | CCc1ccccc1 | 7500 |
| 82 | IMPHY010072 | Eucalyptol | CC12CCC(CC1)C(O2)(C)C | 2758 |
| 83 | IMPHY010080 | beta-Elemene | C=C[C@]1(C)CC[C@H](C[C@H]1C(=C)C)C(=C)C | 6918391 |
| 84 | IMPHY010205 | Pipecolic acid | OC(=O)C1CCCCN1 | 849 |
| 85 | IMPHY010547 | Actinidiolide, dihydro- | O=C1C=C2[C@@](O1)(C)CCCC2(C)C | 6432173 |
| 86 | IMPHY010799 | Tamarindienal | OC(=C)/C=C/C(=O)C=O.O=CC(=O)/C=C/C(=O)C |  |
| 87 | IMPHY010841 | Ethyl linoleate | CCCCC/C=CC/C=CCCCCCCCC(=O)OCC | 5282184 |
| 88 | IMPHY010939 | trans-2,cis-6-Nonadienal | CC/C=CCC/C=C/C=O | 643731 |
| 89 | IMPHY010995 | Toluene | Cc1ccccc1 | 1140 |
| 90 | IMPHY011396 | 4-Carvomenthenol | CC1=CCC(CC1)(O)C(C)C | 11230 |
| 91 | IMPHY011409 | Guaiacol | COc1ccccc1O | 460 |
| 92 | IMPHY011562 | 2-Hexenal | CCC/C=C/C=O | 5281168 |
| 93 | IMPHY011599 | Terpinolene | CC1=CCC(=C(C)C)CC1 | 11463 |
| 94 | IMPHY011609 | alpha-Carotene | C/C(=CC=CC=C(C=CC=C(C=CC1=C(C)CCCC1(C)C)/C)/C)/C=C/C=C(/C=C/[C@H]1C(=CCCC1(C)C)C)C | 6419725 |
| 95 | IMPHY011640 | Isoeugenol | C/C=C/c1ccc(c(c1)OC)O | 853433 |
| 96 | IMPHY011643 | alpha-Terpinene | CC1=CC=C(CC1)C(C)C | 7462 |
| 97 | IMPHY011659 | alpha-Muurolene | CC1=C[C@@H]2[C@H](CC1)C(=CC[C@H]2C(C)C)C | 12306047 |
| 98 | IMPHY011666 | Palmitoleic acid | CCCCCC/C=CCCCCCCCC(=O)O | 445638 |
| 99 | IMPHY011761 | Humulene | C/C/1=CCC(C)(C)/C=C/C/C(=C/CC1)/C | 5281520 |
| 100 | IMPHY011763 | Anethole | C/C=C/c1ccc(cc1)OC | 637563 |
| 101 | IMPHY011793 | (+)-gamma-Cadinene | CC1=C[C@@H]2[C@@H](CC1)C(=C)CC[C@H]2C(C)C | 6432404 |
| 102 | IMPHY011797 | Oleic acid | CCCCCCCC/C=CCCCCCCCC(=O)O | 445639 |
| 103 | IMPHY012058 | Linalool | C=CC(CCC=C(C)C)(O)C | 6549 |
| 104 | IMPHY012061 | alpha-Pinene | CC1=CCC2CC1C2(C)C | 6654 |
| 105 | IMPHY012070 | o-Xylene | Cc1ccccc1C | 7237 |
| 106 | IMPHY012100 | Nonanoic acid | CCCCCCCCC(=O)O | 8158 |
| 107 | IMPHY012147 | beta-Pinene | C=C1CCC2CC1C2(C)C | 14896 |
| 108 | IMPHY012160 | alpha-Terpineol | CC1=CCC(CC1)C(O)(C)C | 17100 |
| 109 | IMPHY012163 | 2-Methyltetrahydrofuran-3-one | O=C1CCOC1C | 18522 |
| 110 | IMPHY012165 | Sabinene | C=C1CCC2(C1C2)C(C)C | 18818 |
| 111 | IMPHY012723 | Linolenic acid | CC/C=CC/C=CC/C=CCCCCCCCC(=O)O | 5280934 |
| 112 | IMPHY012920 | 2-Furanmethanol, 5-ethenyltetrahydro-alpha,alpha,5-trimethyl-, cis- | C=C[C@@]1(C)CC[C@H](O1)C(O)(C)C | 11116492 |
| 113 | IMPHY013750 | 1-Methylpyrrole | Cn1cccc1 | 7304 |
| 114 | IMPHY013759 | Hydroxyacetone | CC(=O)CO | 8299 |
| 115 | IMPHY014811 | alpha-Phellandrene | CC1=CCC(C=C1)C(C)C | 7460 |
| 116 | IMPHY014817 | Aromadendrene | CC1CCC2C1C1C(C1(C)C)CCC2=C | 91354 |
| 117 | IMPHY014831 | beta-Caryophyllene | C/C/1=CCCC(=C)[C@@H]2[C@@H](CC1)C(C2)(C)C | 5281515 |
| 118 | IMPHY014988 | Limonene | CC1=CCC(CC1)C(=C)C | 22311 |
| 119 | IMPHY014989 | trans-Linalool oxide | C=C[C@]1(C)CC[C@H](O1)C(O)(C)C | 6432254 |
| 120 | IMPHY014990 | Linoleic acid | CCCCC/C=CC/C=CCCCCCCCC(=O)O | 5280450 |
| 121 | IMPHY015123 | alpha-Copaene | CC([C@@H]1CC[C@]2([C@@H]3[C@H]1C2C(=CC3)C)C)C | 70678558 |
| 122 | IMPHY015223 | 2,3-Pentanedione | CCC(=O)C(=O)C | 11747 |
| 123 | IMPHY015261 | Pyrrole-2-carboxaldehyde | O=Cc1ccc[nH]1 | 13854 |
| 124 | IMPHY015773 | Propane, 2-(ethenyloxy)- | C=COC(C)C | 13557 |
| 125 | IMPHY015802 | Isomaltol | CC(=O)c1occc1O | 18898 |
| 126 | IMPHY015918 | p-Xylene | Cc1ccc(cc1)C | 7809 |
| 127 | IMPHY016134 | N-Ethylpyrrole | CCn1cccc1 | 185226 |
| 128 | IMPHY016213 | 1H-pyrrole | N1[C]=CC=C1 | 12668434 |
| 129 | IMPHY016224 | 4-Cyclopentene-1,3-dione | O=C1C=CC(=O)C1 | 70258 |
| 130 | IMPHY016300 | 2,3-Dimethylmaleic anhydride | O=C1OC(=O)C(=C1C)C | 13010 |
| 131 | IMPHY016413 | 4-(2,3,6-Trimethylphenyl)but-3-en-2-one | CC(=O)C=Cc1c(C)ccc(c1C)C | 53426133 |
| 132 | IMPHY016457 | 5-Methyl-2(5H)-furanone | CC1C=CC(=O)O1 | 11558 |
| 133 | IMPHY017110 | 5-Methyl-1H-pyrrole-2-carbaldehyde | Cc1ccc([nH]1)C=O | 14508 |
| 134 | IMPHY017305 | Furyl hydroxymethyl ketone | OCC(=O)c1ccco1 | 519466 |
| 135 | IMPHY017732 | Phenethyl butyrate | CCCC(=O)OCCc1ccccc1 | 7658 |
| 136 | IMPHY017767 | 2-Butoxyethyl acetate | CCCCOCCOC(=O)C | 8160 |
| 137 | IMPHY017774 | 4-Penten-2-one | CC(=O)CC=C | 83797 |
| 138 | IMPHY001801 | Isoorientin | OC[C@H]1O[C@H]([C@@H]([C@H]([C@@H]1O)O)O)c1c(O)cc2c(c1O)c(=O)cc(o2)c1ccc(c(c1)O)O | 114776 |
| 139 | IMPHY002983 | 1-Hexadecanol | CCCCCCCCCCCCCCCCO | 2682 |
| 140 | IMPHY003999 | d-Tartaric acid | O[C@@H]([C@@H](C(=O)O)O)C(=O)O | 439655 |
| 141 | IMPHY005455 | Orientin | OC[C@H]1O[C@H]([C@@H]([C@H]([C@@H]1O)O)O)c1c(O)cc(c2c1oc(cc2=O)c1ccc(c(c1)O)O)O | 5281675 |
| 142 | IMPHY006145 | p-Cymene | Cc1ccc(cc1)C(C)C | 7463 |
| 143 | IMPHY007130 | Hordenine | CN(CCc1ccc(cc1)O)C | 68313 |
| 144 | IMPHY008937 | Vitamin E | C[C@@H](CCC[C@]1(C)CCc2c(O1)c(C)c(c(c2C)O)C)CCC[C@@H](CCCC(C)C)C | 14985 |
| 145 | IMPHY009367 | 1-Pentadecanol | CCCCCCCCCCCCCCCO | 12397 |
| 146 | IMPHY010097 | Benzyl benzoate | O=C(c1ccccc1)OCc1ccccc1 | 2345 |
| 147 | IMPHY011554 | beta-Tocopherol | C[C@H](CCC[C@@H](CCCC(C)C)C)CCC[C@]1(C)CCc2c(O1)c(C)cc(c2C)O | 6857447 |
| 148 | IMPHY012058 | Linalool | C=CC(CCC=C(C)C)(O)C | 6549 |
| 149 | IMPHY013752 | Propylbenzene | CCCc1ccccc1 | 7668 |
| 150 | IMPHY014805 | Cedr-8-ene | CC1=CC[C@@]23C[C@@H]1C(C)(C)[C@@H]2CC[C@H]3C | 6431015 |
| 151 | IMPHY012061 | alpha-Pinene | CC1=CCC2CC1C2(C)C | 6654 |
| 152 | IMPHY012147 | beta-Pinene | C=C1CCC2CC1C2(C)C | 14896 |
| 153 | IMPHY012160 | alpha-Terpineol | CC1=CCC(CC1)C(O)(C)C | 17100 |
| 154 | IMPHY012654 | Nerol | OC/C=C(CCC=C(C)C)/C | 643820 |
| 155 | IMPHY012713 | Vitexin | OC[C@H]1O[C@H]([C@@H]([C@H]([C@@H]1O)O)O)c1c(O)cc(c2c1oc(cc2=O)c1ccc(cc1)O)O | 5280441 |
| 156 | IMPHY014835 | (E)-beta-ocimene | C=C/C(=C/CC=C(C)C)/C | 5281553 |
| 157 | IMPHY014988 | Limonene | CC1=CCC(CC1)C(=C)C | 22311 |
| 158 | IMPHY001679 | 2,3,4,6-Tetra-O-methyl-D-galactose | COC[C@H]([C@@H]([C@@H]([C@H](C=O)OC)OC)OC)O | 14104336 |
| 159 | IMPHY003112 | Methyl 3,4-dihydroxybenzoate | COC(=O)c1ccc(c(c1)O)O | 287064 |
| 160 | IMPHY004167 | beta-D-Xylopyranose | O[C@@H]1CO[C@H]([C@@H]([C@H]1O)O)O | 125409 |
| 161 | IMPHY005695 | 2,3,4-Tri-o-methyl-d-xylose | CO[C@H]([C@H](C=O)OC)[C@H](OC)CO | 22215468 |
| 162 | IMPHY006386 | Cardenolide | O=C1OCC(=C1)[C@H]1CC[C@H]2[C@]1(C)CC[C@H]1[C@H]2CCC2[C@]1(C)CCCC2 | 53957771 |
| 163 | IMPHY012050 | D-Galactose | OC[C@H]1OC(O)[C@@H]([C@H]([C@H]1O)O)O | 6036 |
| 164 | IMPHY014746 | 2,3,5-tri-O-methylarabinose | COC[C@H]([C@H]([C@@H](C=O)OC)OC)O | 53940505 |
| 165 | IMPHY014893 | D-Glucose | OC[C@H]1OC(O)[C@@H]([C@H]([C@@H]1O)O)O | 5793 |
| 166 | IMPHY014908 | (-)-Epicatechin | Oc1cc2O[C@H](c3ccc(c(c3)O)O)[C@@H](Cc2c(c1)O)O | 72276 |
| 167 | IMPHY014983 | L-Arabinose | O[C@H]1COC([C@@H]([C@H]1O)O)O | 439195 |
| 168 | IMPHY000547 | Gallacetophenone | CC(=O)c1ccc(c(c1O)O)O | 10706 |
| 169 | IMPHY000569 | Procyanidin | Oc1cc(O)c2c(c1)OC(C(C2)OC1(Oc2cc(O)cc(c2C(C1O)O)O)c1ccc(c(c1)O)O)c1ccc(c(c1)O)O | 107876 |
| 170 | IMPHY000622 | Proanthocyanidin | COc1c(O)cc(cc1O)C1Oc2c(C[C@H]1O)c(O)cc(c2[C@@H]1[C@@H](O)[C@H](Oc2c1c(O)cc(c2)O)c1ccc(cc1)O)O | 108065 |
| 171 | IMPHY001956 | Methyl 2-furoate | COC(=O)c1ccco1 | 11902 |
| 172 | IMPHY002003 | 5-Methylfurfural | Cc1ccc(o1)C=O | 12097 |
| 173 | IMPHY002750 | 2-Acetylfuran | CC(=O)c1ccco1 | 14505 |
| 174 | IMPHY003500 | Citric acid | OC(=O)C(CC(=O)O)(CC(=O)O)O | 311 |
| 175 | IMPHY003999 | d-Tartaric acid | O[C@@H]([C@@H](C(=O)O)O)C(=O)O | 439655 |
| 176 | IMPHY004055 | Choline | OCC[N+](C)(C)C | 305 |
| 177 | IMPHY006362 | Ascorbic acid | OC[C@@H]([C@H]1OC(=O)C(=C1O)O)O | 54670067 |
| 178 | IMPHY006429 | (3,4-Dihydroxyphenyl)acetate | [O-]C(=O)Cc1ccc(c(c1)O)O | 5460350 |
| 179 | IMPHY006934 | 2-Methyl-1-butanol | CCC(CO)C | 8723 |
| 180 | IMPHY006975 | 3,5-Dimethylphenol | Cc1cc(C)cc(c1)O | 7948 |
| 181 | IMPHY007025 | Lysophosphatidylethanolamine | [O-]P(=O)(OCCN)OC[C@@H](COC(=O)C)O | 73755142 |
| 182 | IMPHY007036 | 1-(2-Furyl)propane-1,2-dione | CC(=O)C(=O)c1ccco1 | 74038 |
| 183 | IMPHY007041 | Furfural | O=Cc1ccco1 | 7362 |
| 184 | IMPHY007130 | Hordenine | CN(CCc1ccc(cc1)O)C | 68313 |
| 185 | IMPHY007539 | Phenylacetaldehyde | O=CCc1ccccc1 | 998 |
| 186 | IMPHY008252 | Inositol | OC1C(O)C(O)C(C(C1O)O)O | 892 |
| 187 | IMPHY010799 | Tamarindienal | OC(=C)/C=C/C(=O)C=O.O=CC(=O)/C=C/C(=O)C |  |
| 188 | IMPHY011577 | Procyanidin B2 | Oc1cc(O)c2c(c1)O[C@@H]([C@@H]([C@H]2c1c(O)cc(c2c1O[C@@H]([C@@H](C2)O)c1ccc(c(c1)O)O)O)O)c1ccc(c(c1)O)O | 122738 |
| 189 | IMPHY011761 | Humulene | C/C/1=CCC(C)(C)/C=C/C/C(=C/CC1)/C | 5281520 |
| 190 | IMPHY012061 | alpha-Pinene | CC1=CCC2CC1C2(C)C | 6654 |
| 191 | IMPHY014836 | beta-Sitosterol | CC[C@@H](C(C)C)CC[C@H]([C@H]1CC[C@@H]2[C@]1(C)CC[C@H]1[C@H]2CC=C2[C@]1(C)CC[C@@H](C2)O)C | 222284 |
| 192 | IMPHY014854 | Cianidanol | Oc1cc2O[C@H](c3ccc(c(c3)O)O)[C@H](Cc2c(c1)O)O | 9064 |
| 193 | IMPHY014893 | D-Glucose | OC[C@H]1OC(O)[C@@H]([C@H]([C@@H]1O)O)O | 5793 |
| 194 | IMPHY014908 | (-)-Epicatechin | Oc1cc2O[C@H](c3ccc(c(c3)O)O)[C@@H](Cc2c(c1)O)O | 72276 |
| 195 | IMPHY015123 | alpha-Copaene | CC([C@@H]1CC[C@]2([C@@H]3[C@H]1C2C(=CC3)C)C)C | 70678558 |
|  |  |  |  |  |
| **PLANT NAME : *Terminalia arjuna*** | | | | |
| **Serial No** | **IMPPAT Phytochemical Identifier** | **Phytochemical Name** | **SMILES** | **CID** |
| 1 | IMPHY001114 | Arjunin | OC1O[C@@H]2COC(=O)c3cc(O)c(c(c3-c3c(O)c(O)c4c5c3c(=O)oc3c(c(c(-c6c(C(=O)O[C@H]2[C@@H]([C@H]1O)OC(=O)c1cc(O)c(c(c1)O)O)cc(O)c(c6O)O)c(c(=O)o4)c53)O)O)O)O | 102316370 |
| 2 | IMPHY001431 | Arjunic acid | O[C@@H]1C[C@@]2(C)[C@H](C([C@H]1O)(C)C)CC[C@@]1([C@@H]2CC=C2[C@@]1(C)CC[C@@]1([C@H]2[C@H](O)C(C)(C)CC1)C(=O)O)C | 15385516 |
| 3 | IMPHY002418 | Arjunolone | COc1cc2oc(cc(=O)c2cc1O)c1ccc(cc1)O |  |
| 4 | IMPHY002542 | Cerasidin | COc1ccc(c(c1)OC)/C=C/C(=O)c1c(O)cc(cc1OC)OC | 14034812 |
| 5 | IMPHY004079 | Catechol | Oc1ccccc1O | 289 |
| 6 | IMPHY005514 | Arjunetin | OC[C@H]1O[C@@H](OC(=O)[C@@]23CCC([C@H]([C@H]3C3=CC[C@H]4[C@@]([C@@]3(CC2)C)(C)CC[C@@H]2[C@]4(C)C[C@H]([C@@H](C2(C)C)O)O)O)(C)C)[C@@H]([C@H]([C@@H]1O)O)O | 21152828 |
| 7 | IMPHY005537 | Ellagic acid | Oc1cc2c(=O)oc3c4c2c(c1O)oc(=O)c4cc(c3O)O | 5281855 |
| 8 | IMPHY005607 | Baicalein | Oc1cc2oc(cc(=O)c2c(c1O)O)c1ccccc1 | 5281605 |
| 9 | IMPHY005947 | Myristyl oleate | CCCCCCCCCCCCCCOC(=O)CCCCCCC/C=CCCCCCCCC | 5365034 |
| 10 | IMPHY007450 | Oxalic acid | OC(=O)C(=O)O | 971 |
| 11 | IMPHY008675 | Terminic acid | CC(=C)[C@@H]1CC[C@]2([C@H]1[C@@]1(O)CC[C@H]3[C@@]([C@@]1(CC2)C)(C)CC[C@@H]1[C@]3(C)CCC(C1(C)C)O)C(=O)O | 132568257 |
| 12 | IMPHY008910 | Hentriacontane | CCCCCCCCCCCCCCCCCCCCCCCCCCCCCCC | 12410 |
| 13 | IMPHY010319 | [(10R,11R)-10-[(15S,19S)-2,3,4,7,8,9,19-heptahydroxy-12,17-dioxo-13,16-dioxatetracyclo[13.3.1.05,18.06,11]nonadeca-1,3,5(18),6,8,10-hexaen-14-yl]-3,4,5,17,18,19-hexahydroxy-8,14-dioxo-9,13-dioxatricyc | O=C(c1cc(O)c(c(c1)O)O)O[C@@H]1COC(=O)c2cc(O)c(c(c2-c2c(C(=O)O[C@H]1C1OC(=O)c3cc(O)c(c(c3-c3c4C(=O)O[C@H]1[C@@H](O)c4c(O)c(c3O)O)O)O)cc(O)c(c2O)O)O)O | 101601178 |
| 14 | IMPHY011627 | Arjunolic acid | OC[C@]1(C)[C@@H](O)[C@H](O)C[C@]2([C@H]1CC[C@@]1([C@@H]2CC=C2[C@@]1(C)CC[C@@]1([C@H]2CC(CC1)(C)C)C(=O)O)C)C | 73641 |
| 15 | IMPHY011688 | Friedelin | O=C1CC[C@@H]2[C@]([C@H]1C)(C)CC[C@H]1[C@@]2(C)CC[C@@]2([C@]1(C)CC[C@@]1([C@H]2CC(C)(C)CC1)C)C | 91472 |
| 16 | IMPHY011729 | Mannitol | OC[C@H]([C@H]([C@@H]([C@@H](CO)O)O)O)O | 6251 |
| 17 | IMPHY011735 | (+)-Gallocatechin | Oc1cc2O[C@H](c3cc(O)c(c(c3)O)O)[C@H](Cc2c(c1)O)O | 65084 |
| 18 | IMPHY011737 | Epigallocatechin | Oc1cc2O[C@H](c3cc(O)c(c(c3)O)O)[C@@H](Cc2c(c1)O)O | 72277 |
| 19 | IMPHY011741 | Tannic acid | O=C(c1cc(O)c(c(c1)OC(=O)c1cc(O)c(c(c1)O)O)O)O[C@@H]1[C@@H](COC(=O)c2cc(O)c(c(c2)OC(=O)c2cc(O)c(c(c2)O)O)O)O[C@H]([C@@H]([C@H]1OC(=O)c1cc(O)c(c(c1)OC(=O)c1cc(O)c(c(c1)O)O)O)OC(=O)c1cc(O)c(c(c1)OC(=O)c1cc(O)c(c(c1)O)O)O)OC(=O)c1cc(O)c(c(c1)OC(=O)c1cc(O)c(c(c1)O)O)O | 16129778 |
| 20 | IMPHY011826 | Oleanolic acid | O[C@H]1CC[C@]2([C@H](C1(C)C)CC[C@@]1([C@@H]2CC=C2[C@@]1(C)CC[C@@]1([C@H]2CC(C)(C)CC1)C(=O)O)C)C | 10494 |
| 21 | IMPHY011885 | Leucodelphidin | Oc1cc2O[C@H](c3cc(O)c(c(c3)O)O)[C@H]([C@H](c2c(c1)O)O)O | 440835 |
| 22 | IMPHY012649 | Tomentosic acid | OCC1(C)C(O)C(O)CC2(C1CCC1(C2CC=C2C1(C)CCC1(C2C(O)C(CC1)(C)C)C(=O)O)C)C | 622032 |
| 23 | IMPHY012650 | Arjungenin | OC[C@]1(C)[C@@H](O)[C@H](O)C[C@]2([C@H]1CC[C@@]1([C@@H]2CC=C2[C@@]1(C)CC[C@@]1([C@H]2[C@H](O)C(CC1)(C)C)C(=O)O)C)C | 12444386 |
| 24 | IMPHY014836 | beta-Sitosterol | CC[C@@H](C(C)C)CC[C@H]([C@H]1CC[C@@H]2[C@]1(C)CC[C@H]1[C@H]2CC=C2[C@]1(C)CC[C@@H](C2)O)C | 222284 |
| 25 | IMPHY000321 | Arjunglucoside III | OC[C@H]1O[C@@H](OC(=O)[C@@]23CCC([C@H]([C@H]3C3=CC(=O)[C@H]4[C@@]([C@@]3(CC2)C)(C)CC[C@@H]2[C@]4(C)C[C@H]([C@@H](C2(C)C)O)O)O)(C)C)[C@@H]([C@H]([C@@H]1O)O)O | 102117122 |
| 26 | IMPHY001431 | Arjunic acid | O[C@@H]1C[C@@]2(C)[C@H](C([C@H]1O)(C)C)CC[C@@]1([C@@H]2CC=C2[C@@]1(C)CC[C@@]1([C@H]2[C@H](O)C(C)(C)CC1)C(=O)O)C | 15385516 |
| 27 | IMPHY002542 | Cerasidin | COc1ccc(c(c1)OC)/C=C/C(=O)c1c(O)cc(cc1OC)OC | 14034812 |
| 28 | IMPHY005537 | Ellagic acid | Oc1cc2c(=O)oc3c4c2c(c1O)oc(=O)c4cc(c3O)O | 5281855 |
| 29 | IMPHY005947 | Myristyl oleate | CCCCCCCCCCCCCCOC(=O)CCCCCCC/C=CCCCCCCCC | 5365034 |
| 30 | IMPHY008708 | Arjunone | COc1ccc(c(c1)OC)C1CC(=O)c2c(O1)cc(cc2OC)OC | 14034821 |
| 31 | IMPHY008910 | Hentriacontane | CCCCCCCCCCCCCCCCCCCCCCCCCCCCCCC | 12410 |
| 32 | IMPHY011394 | Arachidic acid | CCCCCCCCCCCCCCCCCCCC(=O)O | 10467 |
| 33 | IMPHY011461 | Methyl oleanolate | COC(=O)[C@@]12CCC(C[C@H]2C2=CC[C@H]3[C@@]([C@@]2(CC1)C)(C)CC[C@@H]1[C@]3(C)CC[C@@H](C1(C)C)O)(C)C | 92900 |
| 34 | IMPHY011688 | Friedelin | O=C1CC[C@@H]2[C@]([C@H]1C)(C)CC[C@H]1[C@@]2(C)CC[C@@]2([C@]1(C)CC[C@@]1([C@H]2CC(C)(C)CC1)C)C | 91472 |
| 35 | IMPHY011741 | Tannic acid | O=C(c1cc(O)c(c(c1)OC(=O)c1cc(O)c(c(c1)O)O)O)O[C@@H]1[C@@H](COC(=O)c2cc(O)c(c(c2)OC(=O)c2cc(O)c(c(c2)O)O)O)O[C@H]([C@@H]([C@H]1OC(=O)c1cc(O)c(c(c1)OC(=O)c1cc(O)c(c(c1)O)O)O)OC(=O)c1cc(O)c(c(c1)OC(=O)c1cc(O)c(c(c1)O)O)O)OC(=O)c1cc(O)c(c(c1)OC(=O)c1cc(O)c(c(c1)O)O)O | 16129778 |
| 36 | IMPHY012021 | Gallic acid | OC(=O)c1cc(O)c(c(c1)O)O | 370 |
| 37 | IMPHY011970 | Maslinic acid | O[C@@H]1C[C@@]2(C)[C@H](C([C@H]1O)(C)C)CC[C@@]1([C@@H]2CC=C2[C@@]1(C)CC[C@@]1([C@H]2CC(C)(C)CC1)C(=O)O)C | 73659 |
| 38 | IMPHY001431 | Arjunic acid | O[C@@H]1C[C@@]2(C)[C@H](C([C@H]1O)(C)C)CC[C@@]1([C@@H]2CC=C2[C@@]1(C)CC[C@@]1([C@H]2[C@H](O)C(C)(C)CC1)C(=O)O)C | 15385516 |
| 39 | IMPHY002400 | Arjunoside II | OCC1OC(OC2C(O)CC3(C(C2(C)C)CCC2(C3CC=C3C2(C)CCC2(C3C(O)C(C)(C)CC2)C(=O)O)C)C)C(C(C1O)O)OC1OC(C)C(C(C1O)O)O |  |
| 40 | IMPHY002418 | Arjunolone | COc1cc2oc(cc(=O)c2cc1O)c1ccc(cc1)O |  |
| 41 | IMPHY005514 | Arjunetin | OC[C@H]1O[C@@H](OC(=O)[C@@]23CCC([C@H]([C@H]3C3=CC[C@H]4[C@@]([C@@]3(CC2)C)(C)CC[C@@H]2[C@]4(C)C[C@H]([C@@H](C2(C)C)O)O)O)(C)C)[C@@H]([C@H]([C@@H]1O)O)O | 21152828 |
| 42 | IMPHY005537 | Ellagic acid | Oc1cc2c(=O)oc3c4c2c(c1O)oc(=O)c4cc(c3O)O | 5281855 |
| 43 | IMPHY008675 | Terminic acid | CC(=C)[C@@H]1CC[C@]2([C@H]1[C@@]1(O)CC[C@H]3[C@@]([C@@]1(CC2)C)(C)CC[C@@H]1[C@]3(C)CCC(C1(C)C)O)C(=O)O | 132568257 |
| 44 | IMPHY011461 | Methyl oleanolate | COC(=O)[C@@]12CCC(C[C@H]2C2=CC[C@H]3[C@@]([C@@]2(CC1)C)(C)CC[C@@H]1[C@]3(C)CC[C@@H](C1(C)C)O)(C)C | 92900 |
| 45 | IMPHY011611 | Leucocianidol | Oc1cc2O[C@H](c3ccc(c(c3)O)O)[C@H]([C@H](c2c(c1)O)O)O | 440833 |
| 46 | IMPHY011627 | Arjunolic acid | OC[C@]1(C)[C@@H](O)[C@H](O)C[C@]2([C@H]1CC[C@@]1([C@@H]2CC=C2[C@@]1(C)CC[C@@]1([C@H]2CC(CC1)(C)C)C(=O)O)C)C | 73641 |
| 47 | IMPHY011699 | 8-Hydroxyhexadecanoic acid | CCCCCCCCC(CCCCCCC(=O)O)O | 15569773 |
| 48 | IMPHY011826 | Oleanolic acid | O[C@H]1CC[C@]2([C@H](C1(C)C)CC[C@@]1([C@@H]2CC=C2[C@@]1(C)CC[C@@]1([C@H]2CC(C)(C)CC1)C(=O)O)C)C | 10494 |
| 49 | IMPHY012021 | Gallic acid | OC(=O)c1cc(O)c(c(c1)O)O | 370 |
| 50 | IMPHY013222 | arjunglucoside I | OC[C@H]1O[C@@H](OC(=O)[C@@]23CCC([C@H]([C@H]3C3=CC[C@H]4[C@@]([C@@]3(CC2)C)(C)CC[C@@H]2[C@]4(C)C[C@H]([C@@H]([C@@]2(C)CO)O)O)O)(C)C)[C@@H]([C@H]([C@@H]1O)O)O | 14658050 |
| 51 | IMPHY014836 | beta-Sitosterol | CC[C@@H](C(C)C)CC[C@H]([C@H]1CC[C@@H]2[C@]1(C)CC[C@H]1[C@H]2CC=C2[C@]1(C)CC[C@@H](C2)O)C | 222284 |
| 52 | IMPHY005607 | Baicalein | Oc1cc2oc(cc(=O)c2c(c1O)O)c1ccccc1 | 5281605 |
| 53 | IMPHY011885 | Leucodelphidin | Oc1cc2O[C@H](c3cc(O)c(c(c3)O)O)[C@H]([C@H](c2c(c1)O)O)O | 440835 |
| 54 | IMPHY000321 | Arjunglucoside III | OC[C@H]1O[C@@H](OC(=O)[C@@]23CCC([C@H]([C@H]3C3=CC(=O)[C@H]4[C@@]([C@@]3(CC2)C)(C)CC[C@@H]2[C@]4(C)C[C@H]([C@@H](C2(C)C)O)O)O)(C)C)[C@@H]([C@H]([C@@H]1O)O)O | 102117122 |
| 55 | IMPHY000896 | 2,3-(S)-hexahydroxydiphenoyl-D-glucose | OCC1OC(O)C2C(C1O)OC(=O)c1cc(O)c(c(c1-c1c(C(=O)O2)cc(O)c(c1O)O)O)O | 492390 |
| 56 | IMPHY002542 | Cerasidin | COc1ccc(c(c1)OC)/C=C/C(=O)c1c(O)cc(cc1OC)OC | 14034812 |
| 57 | IMPHY004079 | Catechol | Oc1ccccc1O | 289 |
| 58 | IMPHY004097 | Stearate | CCCCCCCCCCCCCCCCCC(=O)[O-] | 3033836 |
| 59 | IMPHY004562 | Afrormosin | COc1ccc(cc1)c1coc2c(c1=O)cc(c(c2)O)OC | 5281704 |
| 60 | IMPHY005514 | Arjunetin | OC[C@H]1O[C@@H](OC(=O)[C@@]23CCC([C@H]([C@H]3C3=CC[C@H]4[C@@]([C@@]3(CC2)C)(C)CC[C@@H]2[C@]4(C)C[C@H]([C@@H](C2(C)C)O)O)O)(C)C)[C@@H]([C@H]([C@@H]1O)O)O | 21152828 |
| 61 | IMPHY005537 | Ellagic acid | Oc1cc2c(=O)oc3c4c2c(c1O)oc(=O)c4cc(c3O)O | 5281855 |
| 62 | IMPHY005607 | Baicalein | Oc1cc2oc(cc(=O)c2c(c1O)O)c1ccccc1 | 5281605 |
| 63 | IMPHY005947 | Myristyl oleate | CCCCCCCCCCCCCCOC(=O)CCCCCCC/C=CCCCCCCCC | 5365034 |
| 64 | IMPHY006658 | Pentagalloylglucose | O=C(c1cc(O)c(c(c1)O)O)O[C@@H]1O[C@H](COC(=O)c2cc(O)c(c(c2)O)O)[C@H]([C@@H]([C@H]1OC(=O)c1cc(O)c(c(c1)O)O)OC(=O)c1cc(O)c(c(c1)O)O)OC(=O)c1cc(O)c(c(c1)O)O | 65238 |
| 65 | IMPHY008708 | Arjunone | COc1ccc(c(c1)OC)C1CC(=O)c2c(O1)cc(cc2OC)OC | 14034821 |
| 66 | IMPHY008910 | Hentriacontane | CCCCCCCCCCCCCCCCCCCCCCCCCCCCCCC | 12410 |
| 67 | IMPHY008919 | 2alpha,3beta,23-trihydroxyolean-12-en-28-oic acid 28-O-beta-D-glucopynoside | OC[C@H]1O[C@@H](OC(=O)[C@@]23CCC(C[C@H]3C3=CC[C@H]4[C@@]([C@@]3(CC2)C)(C)CC[C@@H]2[C@]4(C)C[C@H]([C@@H]([C@@]2(C)CO)O)O)(C)C)[C@@H]([C@H]([C@@H]1O)O)O | 52951052 |
| 68 | IMPHY010360 | [(2S,3R,4S,5S,6R)-6-hydroxy-3,4,5-tris[(3,4,5-trihydroxybenzoyl)oxy]tetrahydropyran-2-yl]methyl 3,4,5-trihydroxybenzoate | O[C@@H]1O[C@@H](COC(=O)c2cc(O)c(c(c2)O)O)[C@H]([C@@H]([C@@H]1OC(=O)c1cc(O)c(c(c1)O)O)OC(=O)c1 | 49777225 |
| 69 | IMPHY011394 | Arachidic acid | CCCCCCCCCCCCCCCCCCCC(=O)O | 10467 |
| 70 | IMPHY011461 | Methyl oleanolate | COC(=O)[C@@]12CCC(C[C@H]2C2=CC[C@H]3[C@@]([C@@]2(CC1)C)(C)CC[C@@H]1[C@]3(C)CC[C@@H](C1(C)C)O)(C)C | 92900 |
| 71 | IMPHY011627 | Arjunolic acid | OC[C@]1(C)[C@@H](O)[C@H](O)C[C@]2([C@H]1CC[C@@]1([C@@H]2CC=C2[C@@]1(C)CC[C@@]1([C@H]2CC(CC1)(C)C)C(=O)O)C)C | 73641 |
| 72 | IMPHY011699 | 8-Hydroxyhexadecanoic acid | CCCCCCCCC(CCCCCCC(=O)O)O | 15569773 |
| 73 | IMPHY011729 | Mannitol | OC[C@H]([C@H]([C@@H]([C@@H](CO)O)O)O)O | 6251 |
| 74 | IMPHY011735 | (+)-Gallocatechin | Oc1cc2O[C@H](c3cc(O)c(c(c3)O)O)[C@H](Cc2c(c1)O)O | 65084 |
| 75 | IMPHY011885 | Leucodelphidin | Oc1cc2O[C@H](c3cc(O)c(c(c3)O)O)[C@H]([C@H](c2c(c1)O)O)O | 440835 |
| 76 | IMPHY011966 | (+)-Leucocyanidin | Oc1cc2O[C@H](c3ccc(c(c3)O)O)[C@H]([C@@H](c2c(c1)O)O)O | 155206 |
| 77 | IMPHY012021 | Gallic acid | OC(=O)c1cc(O)c(c(c1)O)O | 370 |
| 78 | IMPHY012649 | Tomentosic acid | OCC1(C)C(O)C(O)CC2(C1CCC1(C2CC=C2C1(C)CCC1(C2C(O)C(CC1)(C)C)C(=O)O)C)C | 622032 |
| 79 | IMPHY012650 | Arjungenin | OC[C@]1(C)[C@@H](O)[C@H](O)C[C@]2([C@H]1CC[C@@]1([C@@H]2CC=C2[C@@]1(C)CC[C@@]1([C@H]2[C@H](O)C(CC1)(C)C)C(=O)O)C)C | 12444386 |
| 80 | IMPHY013222 | arjunglucoside I | OC[C@H]1O[C@@H](OC(=O)[C@@]23CCC([C@H]([C@H]3C3=CC[C@H]4[C@@]([C@@]3(CC2)C)(C)CC[C@@H]2[C@]4(C)C[C@H]([C@@H]([C@@]2(C)CO)O)O)O)(C)C)[C@@H]([C@H]([C@@H]1O)O)O | 14658050 |
| 81 | IMPHY014836 | beta-Sitosterol | CC[C@@H](C(C)C)CC[C@H]([C@H]1CC[C@@H]2[C@]1(C)CC[C@H]1[C@H]2CC=C2[C@]1(C)CC[C@@H](C2)O)C | 222284 |
|  |  |  |  |  |
| **PLANT NAME : *Terminalia bellirica*** | | | | |
| **Serial No** | **IMPPAT Phytochemical Identifier** | **Phytochemical Name** | **SMILES** | **CID** |
| 1 | IMPHY000214 | Bellericaside B | OC[C@H]1O[C@@](O)(C[C@@]2(CO)[C@@H](O)[C@H](O)C[C@]3([C@H]2CC[C@@]2([C@@H]3CC=C3[C@@]2(C)CC[C@@]2([C@H]3[C@H](O)C(CC2)(C)C)C(=O)O)C)C)[C@@H](C(C1(O)O)(O)O)O | 102145922 |
| 2 | IMPHY002055 | Celastrol | O=C1C=C2C(=CC=C3[C@@]2(C)CC[C@@]2([C@]3(C)CC[C@@]3([C@H]2C[C@@](C)(CC3)C(=O)O)C)C)C(=C1O)C | 122724 |
| 3 | IMPHY002259 | Bellericoside | OC[C@H]1O[C@@H](OC(=O)[C@@]23CCC(C[C@H]3C3=CC[C@H]4[C@@]([C@@]3(CC2)C)(C)CC[C@@H]2[C@]4(C)C[C@H]([C@@H](C2(CO)CO)O)O)(C)C)[C@@H]([C@H]([C@@H]1O)O)O | 101425822 |
| 4 | IMPHY004799 | Chebulagic acid | OC(=O)C[C@@H]1C(=O)O[C@@H]2[C@H]3COC(=O)c4cc(O)c(c(c4-c4c(C(=O)O[C@@H]2[C@@H](OC(=O)c2c5[C@H]1[C@H](O)C(=O)Oc5c(c(c2)O)O)[C@@H](O3)OC(=O)c1cc(O)c(c(c1)O)O)cc(O)c(c4O)O)O)O | 442674 |
| 5 | IMPHY005161 | Bellericagenin B | OCC1(CO)[C@@H](O)[C@H](O)C[C@]2([C@H]1CC[C@@]1([C@@H]2CC=C2[C@@]1(C)CC[C@@]1([C@H]2[C@H](O)C(CC1)(C)C)C(=O)O)C)C | 21726783 |
| 6 | IMPHY005173 | (4aS,6aR,6aR,6bR,7R,8aR,9R,10R,11R,12aR,14bS)-7,10,11-trihydroxy-9-(hydroxymethyl)-2,2,6a,6b,9,12a-hexamethyl-1,3,4,5,6,6a,7,8,8a,10,11,12,13,14b-tetradecahydropicene-4a-carboxylic acid | OC[C@]1(C)[C@@H](O)[C@H](O)C[C@]2([C@H]1C[C@@H](O)[C@@]1([C@@H]2CC=C2[C@@]1(C)CC[C@@]1([C@H]2CC(CC1)(C)C)C(=O)O)C)C | 21726781 |
| 7 | IMPHY005186 | [(2S,3R,4S,5S,6R)-3,4,5-trihydroxy-6-(hydroxymethyl)oxan-2-yl] (4aS,6aR,6aR,6bR,7R,8aR,9R,10R,11R,12aR,14bS)-7,10,11-trihydroxy-9-(hydroxymethyl)-2,2,6a,6b,9,12a-hexamethyl-1,3,4,5,6,6a,7,8,8a,10,11,1 | OC[C@H]1O[C@@H](OC(=O)[C@@]23CCC(C[C@H]3C3=CC[C@H]4[C@@]([C@@]3(CC2)C)(C)[C@H](O)C[C@@H]2[C@]4(C)C[C@H]([C@@H]([C@@]2(C)CO)O)O)(C)C)[C@@H]([C@H]([C@@H]1O)O)O | 21726782 |
| 8 | IMPHY005537 | Ellagic acid | Oc1cc2c(=O)oc3c4c2c(c1O)oc(=O)c4cc(c3O)O | 5281855 |
| 9 | IMPHY009606 | Ethyl gallate | CCOC(=O)c1cc(O)c(c(c1)O)O | 13250 |
| 10 | IMPHY010070 | beta-Glucogallin | OC[C@H]1O[C@@H](OC(=O)c2cc(O)c(c(c2)O)O)[C@@H]([C@H]([C@@H]1O)O)O | 124021 |
| 11 | IMPHY011729 | Mannitol | OC[C@H]([C@H]([C@@H]([C@@H](CO)O)O)O)O | 6251 |
| 12 | IMPHY012021 | Gallic acid | OC(=O)c1cc(O)c(c(c1)O)O | 370 |
| 13 | IMPHY014836 | beta-Sitosterol | CC[C@@H](C(C)C)CC[C@H]([C@H]1CC[C@@H]2[C@]1(C)CC[C@H]1[C@H]2CC=C2[C@]1(C)CC[C@@H](C2)O)C | 222284 |
| 14 | IMPHY000214 | Bellericaside B | OC[C@H]1O[C@@](O)(C[C@@]2(CO)[C@@H](O)[C@H](O)C[C@]3([C@H]2CC[C@@]2([C@@H]3CC=C3[C@@]2(C)CC[C@@]2([C@H]3[C@H](O)C(CC2)(C)C)C(=O)O)C)C)[C@@H](C(C1(O)O)(O)O)O | 102145922 |
| 15 | IMPHY000782 | 7-Hydroxy-3',4'-methylenedioxyflavan | Oc1ccc2c(c1)O[C@@H](CC2)c1ccc2c(c1)OCO2 | 466078 |
| 16 | IMPHY000800 | Termilignan | COc1ccc(c(c1)O)CC(=C)C(=C)Cc1ccc(cc1)O | 466076 |
| 17 | IMPHY001682 | (4aS,6aR,6aS,6bR,8aR,10R,11R,12aR,14bS)-10,11-dihydroxy-9,9-bis(hydroxymethyl)-2,2,6a,6b,12a-pentamethyl-1,3,4,5,6,6a,7,8,8a,10,11,12,13,14b-tetradecahydropicene-4a-carboxylic acid | OCC1(CO)[C@@H](O)[C@H](O)C[C@]2([C@H]1CC[C@@]1([C@@H]2CC=C2[C@@]1(C)CC[C@@]1([C@H]2CC(CC1)(C)C)C(=O)O)C)C | 14105933 |
| 18 | IMPHY002954 | Tetratriacontane | CCCCCCCCCCCCCCCCCCCCCCCCCCCCCCCCCC | 26519 |
| 19 | IMPHY004799 | Chebulagic acid | OC(=O)C[C@@H]1C(=O)O[C@@H]2[C@H]3COC(=O)c4cc(O)c(c(c4-c4c(C(=O)O[C@@H]2[C@@H](OC(=O)c2c5[C@H]1[C@H](O)C(=O)Oc5c(c(c2)O)O)[C@@H](O3)OC(=O)c1cc(O)c(c(c1)O)O)cc(O)c(c4O)O)O)O | 442674 |
| 20 | IMPHY005161 | Bellericagenin B | OCC1(CO)[C@@H](O)[C@H](O)C[C@]2([C@H]1CC[C@@]1([C@@H]2CC=C2[C@@]1(C)CC[C@@]1([C@H]2[C@H](O)C(CC1)(C)C)C(=O)O)C)C | 21726783 |
| 21 | IMPHY005173 | (4aS,6aR,6aR,6bR,7R,8aR,9R,10R,11R,12aR,14bS)-7,10,11-trihydroxy-9-(hydroxymethyl)-2,2,6a,6b,9,12a-hexamethyl-1,3,4,5,6,6a,7,8,8a,10,11,12,13,14b-tetradecahydropicene-4a-carboxylic acid | (4aS,6aR,6aR,6bR,7R,8aR,9R,10R,11R,12aR,14bS)-7,10,11-trihydroxy-9-(hydroxymethyl)-2,2,6a,6b,9,12a-hexamethyl-1,3,4,5,6,6a,7,8,8a,10,11,12,13,14b-tetradecahydropicene-4a-carboxylic acid | 21726781 |
| 22 | IMPHY005186 | (2S,3R,4S,5S,6R)-3,4,5-trihydroxy-6-(hydroxymethyl)oxan-2-yl] (4aS,6aR,6aR,6bR,7R,8aR,9R,10R,11R,12aR,14bS)-7,10,11-trihydroxy-9-(hydroxymethyl)-2,2,6a,6b,9,12a-hexamethyl-1,3,4,5,6,6a,7,8,8a,10,11,1 | OC[C@H]1O[C@@H](OC(=O)[C@@]23CCC(C[C@H]3C3=CC[C@H]4[C@@]([C@@]3(CC2)C)(C)[C@H](O)C[C@@H]2[C@]4(C)C[C@H]([C@@H]([C@@]2(C)CO)O)O)(C)C)[C@@H]([C@H]([C@@H]1O)O)O | 21726782 |
| 23 | IMPHY005537 | Ellagic acid | Oc1cc2c(=O)oc3c4c2c(c1O)oc(=O)c4cc(c3O)O | 5281855 |
| 24 | IMPHY006963 | Anolignan B | C=C(C(=C)Cc1ccc(cc1)O)Cc1ccc(cc1)O | 72388 |
| 25 | IMPHY008935 | Tritriacontan-9-one | CCCCCCCCCCCCCCCCCCCCCCCCC(=O)CCCCCCCC | 129688132 |
| 26 | IMPHY009483 | Tritriacontane | CCCCCCCCCCCCCCCCCCCCCCCCCCCCCCCCC | 12411 |
| 27 | IMPHY009606 | Ethyl gallate | CCOC(=O)c1cc(O)c(c(c1)O)O | 13250 |
| 28 | IMPHY010070 | beta-Glucogallin | OC[C@H]1O[C@@H](OC(=O)c2cc(O)c(c(c2)O)O)[C@@H]([C@H]([C@@H]1O)O)O | 124021 |
| 29 | IMPHY010965 | Corilagin | O[C@@H]1[C@H]2COC(=O)c3cc(O)c(c(c3-c3c(C(=O)O[C@@H]1[C@H]([C@@H](O2)OC(=O)c1cc(O)c(c(c1)O)O)O)cc(O)c(c3O)O)O)O | 73568 |
| 30 | IMPHY011729 | Mannitol | OC[C@H]([C@H]([C@@H]([C@@H](CO)O)O)O)O | 6251 |
| 31 | IMPHY011741 | Tannic acid | O=C(c1cc(O)c(c(c1)OC(=O)c1cc(O)c(c(c1)O)O)O)O[C@@H]1[C@@H](COC(=O)c2cc(O)c(c(c2)OC(=O)c2cc(O)c(c(c2)O)O)O)O[C@H]([C@@H]([C@H]1OC(=O)c1cc(O)c(c(c1)OC(=O)c1cc(O)c(c(c1)O)O)O)OC(=O)c1cc(O)c(c(c1)OC(=O)c1cc(O)c(c(c1)O)O)O)OC(=O)c1cc(O)c(c(c1)OC(=O)c1cc(O)c(c(c1)O)O)O | 16129778 |
| 32 | IMPHY012021 | Gallic acid | OC(=O)c1cc(O)c(c(c1)O)O | 370 |
| 33 | IMPHY012050 | D-Galactose | OC[C@H]1OC(O)[C@@H]([C@H]([C@H]1O)O)O | 6036 |
| 34 | IMPHY012650 | Arjungenin | OC[C@]1(C)[C@@H](O)[C@H](O)C[C@]2([C@H]1CC[C@@]1([C@@H]2CC=C2[C@@]1(C)CC[C@@]1([C@H]2[C@H](O)C(CC1)(C)C)C(=O)O)C)C | 12444386 |
| 35 | IMPHY014836 | beta-Sitosterol | CC[C@@H](C(C)C)CC[C@H]([C@H]1CC[C@@H]2[C@]1(C)CC[C@H]1[C@H]2CC=C2[C@]1(C)CC[C@@H](C2)O)C | 222284 |
| 36 | IMPHY014893 | D-Glucose | OC[C@H]1OC(O)[C@@H]([C@H]([C@@H]1O)O)O | 5793 |
| 37 | IMPHY014916 | D-Fructose | OCC1(O)OC[C@H]([C@H]([C@@H]1O)O)O | 2723872 |
| 38 | IMPHY015056 | L-Rhamnose | O[C@H]1[C@H](C)OC([C@@H]([C@@H]1O)O)O | 25310 |
| 39 | IMPHY007327 | Palmitic acid | CCCCCCCCCCCCCCCC(=O)O | 985 |
| 40 | IMPHY007417 | Ethyl acetate | CCOC(=O)C | 8857 |
| 41 | IMPHY007450 | Oxalic acid | OC(=O)C(=O)O | 971 |
| 42 | IMPHY008728 | [3-[Hydroxy-(2,3,4,5,6-pentahydroxycyclohexyl)oxyphosphoryl]oxy-2-octadecanoyloxypropyl] octadecanoate | CCCCCCCCCCCCCCCCCC(=O)OCC(OC(=O)CCCCCCCCCCCCCCCCC)COP(=O)(OC1C(O)C(O)C(C(C1O)O)O)O | 44134894 |
| 43 | IMPHY009826 | 1-(11Z-icosenoyl)-2-(9Z,12Z-octadecadienoyl)-sn-glycero-3-phosphoethanolamine | CCCCCCCC/C=CCCCCCCCCCC(=O)OC[C@@H](OC(=O)CCCCCCC/C=CC/C=CCCCCC)COP(=O)(OCC[NH3+])[O-] | 102515444 |
| 44 | IMPHY009953 | 1-Stearoyl-2-linoleoyl-sn-glycero-3-phosphatidylcholine | CCCCCCCCCCCCCCCCCC(=O)OC[C@@H](OC(=O)CCCCCCC/C=CC/C=CCCCCC)COP(=O)(OCC[N+](C)(C)C)[O-] | 6441487 |
| 45 | IMPHY011741 | Tannic acid | O=C(c1cc(O)c(c(c1)OC(=O)c1cc(O)c(c(c1)O)O)O)O[C@@H]1[C@@H](COC(=O)c2cc(O)c(c(c2)OC(=O)c2cc(O)c(c(c2)O)O)O)O[C@H]([C@@H]([C@H]1OC(=O)c1cc(O)c(c(c1)OC(=O)c1cc(O)c(c(c1)O)O)O)OC(=O)c1cc(O)c(c(c1)OC(=O)c1cc(O)c(c(c1)O)O)O)OC(=O)c1cc(O)c(c(c1)OC(=O)c1cc(O)c(c(c1)O)O)O | 16129778 |
| 46 | IMPHY011797 | Oleic acid | CCCCCCCC/C=CCCCCCCCC(=O)O | 445639 |
| 47 | IMPHY012021 | Gallic acid | OC(=O)c1cc(O)c(c(c1)O)O | 370 |
| 48 | IMPHY014990 | Linoleic acid | CCCCC/C=CC/C=CCCCCCCCC(=O)O | 5280450 |
| 49 | IMPHY004799 | Chebulagic acid | OC(=O)C[C@@H]1C(=O)O[C@@H]2[C@H]3COC(=O)c4cc(O)c(c(c4-c4c(C(=O)O[C@@H]2[C@@H](OC(=O)c2c5[C@H]1[C@H](O)C(=O)Oc5c(c(c2)O)O)[C@@H](O3)OC(=O)c | 442674 |
| 50 | IMPHY005537 | Ellagic acid | Oc1cc2c(=O)oc3c4c2c(c1O)oc(=O)c4cc(c3O)O | 5281855 |
| 51 | IMPHY000214 | Bellericaside B | OC[C@H]1O[C@@](O)(C[C@@]2(CO)[C@@H](O)[C@H](O)C[C@]3([C@H]2CC[C@@]2([C@@H]3CC=C3[C@@]2(C)CC[C@@]2([C@H]3[C@H](O)C(CC2)(C)C)C(=O)O)C)C)[C@@H](C(C1(O)O)(O)O)O | 102145922 |
| 52 | IMPHY000782 | 7-Hydroxy-3',4'-methylenedioxyflavan | Oc1ccc2c(c1)O[C@@H](CC2)c1ccc2c(c1)OCO2 | 466078 |
| 53 | IMPHY001682 | (4aS,6aR,6aS,6bR,8aR,10R,11R,12aR,14bS)-10,11-dihydroxy-9,9-bis(hydroxymethyl)-2,2,6a,6b,12a-pentamethyl-1,3,4,5,6,6a,7,8,8a,10,11,12,13,14b-tetradecahydropicene-4a-carboxylic acid | OCC1(CO)[C@@H](O)[C@H](O)C[C@]2([C@H]1CC[C@@]1([C@@H]2CC=C2[C@@]1(C)CC[C@@]1([C@H]2CC(CC1)(C)C)C(=O)O)C)C | 14105933 |
| 54 | IMPHY002259 | Bellericoside | OC[C@H]1O[C@@H](OC(=O)[C@@]23CCC(C[C@H]3C3=CC[C@H]4[C@@]([C@@]3(CC2)C)(C)CC[C@@H]2[C@]4(C)C[C@H]([C@@H](C2(CO)CO)O)O)(C)C)[C@@H]([C@H]([C@@H]1O)O)O | 101425822 |
| 55 | IMPHY004799 | Chebulagic acid | OC(=O)C[C@@H]1C(=O)O[C@@H]2[C@H]3COC(=O)c4cc(O)c(c(c4-c4c(C(=O)O[C@@H]2[C@@H](OC(=O)c2c5[C@H]1[C@H](O)C(=O)Oc5c(c(c2)O)O)[C@@H](O3)OC(=O)c1cc(O)c(c(c1)O)O)cc(O)c(c4O)O)O)O | 442674 |
| 56 | IMPHY005161 | Bellericagenin B | OCC1(CO)[C@@H](O)[C@H](O)C[C@]2([C@H]1CC[C@@]1([C@@H]2CC=C2[C@@]1(C)CC[C@@]1([C@H]2[C@H](O)C(CC1)(C)C)C(=O)O)C)C | 21726783 |
| 57 | IMPHY005173 | (4aS,6aR,6aR,6bR,7R,8aR,9R,10R,11R,12aR,14bS)-7,10,11-trihydroxy-9-(hydroxymethyl)-2,2,6a,6b,9,12a-hexamethyl-1,3,4,5,6,6a,7,8,8a,10,11,12,13,14b-tetradecahydropicene-4a-carboxylic acid | OC[C@]1(C)[C@@H](O)[C@H](O)C[C@]2([C@H]1C[C@@H](O)[C@@]1([C@@H]2CC=C2[C@@]1(C)CC[C@@]1([C@H]2CC(CC1)(C)C)C(=O)O)C)C | 21726781 |
| 58 | IMPHY005186 | [(2S,3R,4S,5S,6R)-3,4,5-trihydroxy-6-(hydroxymethyl)oxan-2-yl] (4aS,6aR,6aR,6bR,7R,8aR,9R,10R,11R,12aR,14bS)-7,10,11-trihydroxy-9-(hydroxymethyl)-2,2,6a,6b,9,12a-hexamethyl-1,3,4,5,6,6a,7,8,8a,10,11,1 | OC[C@H]1O[C@@H](OC(=O)[C@@]23CCC(C[C@H]3C3=CC[C@H]4[C@@]([C@@]3(CC2)C)(C)[C@H](O)C[C@@H]2[C@]4(C)C[C@H]([C@@H]([C@@]2(C)CO)O)O)(C)C)[C@@H]([C@H]([C@@H]1O)O)O | 21726782 |
| 59 | IMPHY005537 | Ellagic acid | Oc1cc2c(=O)oc3c4c2c(c1O)oc(=O)c4cc(c3O)O | 5281855 |
| 60 | IMPHY006963 | Anolignan B | C=C(C(=C)Cc1ccc(cc1)O)Cc1ccc(cc1)O | 72388 |
| 61 | IMPHY007327 | Palmitic acid | CCCCCCCCCCCCCCCC(=O)O | 985 |
| 62 | IMPHY007450 | Oxalic acid | OC(=O)C(=O)O | 971 |
| 63 | IMPHY009606 | Ethyl gallate | CCOC(=O)c1cc(O)c(c(c1)O)O | 13250 |
| 64 | IMPHY010070 | beta-Glucogallin | OC[C@H]1O[C@@H](OC(=O)c2cc(O)c(c(c2)O)O)[C@@H]([C@H]([C@@H]1O)O)O | 124021 |
| 65 | IMPHY010827 | Chebulinic acid | OC(=O)C[C@@H]1C(=O)O[C@@H]2[C@@H](COC(=O)c3cc(O)c(c(c3)O)O)O[C@H]([C@@H]([C@H]2OC(=O)c2cc(O)c(c(c2)O)O)OC(=O)c2c3[C@H]1[C@H](O)C(=O)Oc3c(c(c2)O)O)OC(=O)c1cc(O)c(c(c1)O)O | 72284 |
| 66 | IMPHY010965 | Corilagin | O[C@@H]1[C@H]2COC(=O)c3cc(O)c(c(c3-c3c(C(=O)O[C@@H]1[C@H]([C@@H](O2)OC(=O)c1cc(O)c(c(c1)O)O)O)cc(O)c(c3O)O)O)O | 73568 |
| 67 | IMPHY010998 | Chebulic acid | OC(=O)C[C@@H]([C@@H]1[C@H](OC(=O)c2c1c(O)c(c(c2)O)O)C(=O)O)C(=O)O | 71308174 |
| 68 | IMPHY011741 | Tannic acid | O=C(c1cc(O)c(c(c1)OC(=O)c1cc(O)c(c(c1)O)O)O)O[C@@H]1[C@@H](COC(=O)c2cc(O)c(c(c2)OC(=O)c2cc(O)c(c(c2)O)O)O)O[C@H]([C@@H]([C@H]1OC(=O)c1cc(O)c(c(c1)OC(=O)c1cc(O)c(c(c1)O)O)O)OC(=O)c1cc(O)c(c(c1)OC(=O)c1cc(O)c(c(c1)O)O)O)OC(=O)c1cc(O)c(c(c1)OC(=O)c1cc(O)c(c(c1)O)O)O | 16129778 |
| 69 | IMPHY011797 | Oleic acid | CCCCCCCC/C=CCCCCCCCC(=O)O | 445639 |
| 70 | IMPHY012021 | Gallic acid | OC(=O)c1cc(O)c(c(c1)O)O | 370 |
| 71 | IMPHY012050 | D-Galactose | OC[C@H]1OC(O)[C@@H]([C@H]([C@H]1O)O)O | 6036 |
| 72 | IMPHY012649 | Tomentosic acid | OCC1(C)C(O)C(O)CC2(C1CCC1(C2CC=C2C1(C)CCC1(C2C(O)C(CC1)(C)C)C(=O)O)C)C | 622032 |
| 73 | IMPHY012650 | Arjungenin | OC[C@]1(C)[C@@H](O)[C@H](O)C[C@]2([C@H]1CC[C@@]1([C@@H]2CC=C2[C@@]1(C)CC[C@@]1([C@H]2[C@H](O)C(CC1)(C)C)C(=O)O)C)C | 12444386 |
| 74 | IMPHY013222 | arjunglucoside I | OC[C@H]1O[C@@H](OC(=O)[C@@]23CCC([C@H]([C@H]3C3=CC[C@H]4[C@@]([C@@]3(CC2)C)(C)CC[C@@H]2[C@]4(C)C[C@H]([C@@H]([C@@]2(C)CO)O)O)O)(C)C)[C@@H]([C@H]([C@@H]1O)O)O | 14658050 |
| 75 | IMPHY014836 | beta-Sitosterol | CC[C@@H](C(C)C)CC[C@H]([C@H]1CC[C@@H]2[C@]1(C)CC[C@H]1[C@H]2CC=C2[C@]1(C)CC[C@@H](C2)O)C | 222284 |
| 76 | IMPHY014990 | Linoleic acid | CCCCC/C=CC/C=CCCCCCCCC(=O)O | 5280450 |
|  |  |  |  |  |
| **PLANT NAME : *Terminalia chebula*** | | | | |
| **Serial No** | **IMPPAT Phytochemical Identifier** | **Phytochemical Names** | **SMILES** | **CID** |
| 1 | IMPHY001187 | [(2S,3R,4S,5S,6R)-3,4,5-trihydroxy-6-(hydroxymethyl)oxan-2-yl] (4aS,6aR,6aS,6bR,8R,8aR,9S,10R,11R,12aR,14bS)-8,10,11-trihydroxy-9-(hydroxymethyl)-2,2,6a,6b,9,12a-hexamethyl-1,3,4,5,6,6a,7,8,8a,10,11,1 | OC[C@H]1O[C@@H](OC(=O)[C@@]23CCC(C[C@H]3C3=CC[C@H]4[C@@]([C@@]3(CC2)C)(C)C[C@@H](O)[C@@H]2[C@]4(C)C[C@H]([C@@H]([C@]2(C)CO)O)O)(C)C)[C@@H]([C@H]([C@@H]1O)O)O | 102005082 |
| 2 | IMPHY003139 | Terchebin | O=C(c1cc(O)c(c(c1)O)O)OC[C@H]1O[C@@H](OC(=O)c2cc(O)c(c(c2)O)O)[C@H]2[C@H]([C@@H]1OC(=O)C1=CC(=O)C(C3([C@@H]1c1c(C(=O)O2)cc(c(c1O3)O)O)O)(O)O)OC(=O)c1cc(O)c(c(c1)O)O | 3084341 |
| 3 | IMPHY004631 | Stearic acid | CCCCCCCCCCCCCCCCCC(=O)O | 5281 |
| 4 | IMPHY007327 | Palmitic acid | CCCCCCCCCCCCCCCC(=O)O | 985 |
| 5 | IMPHY011394 | Arachidic acid | CCCCCCCCCCCCCCCCCCCC(=O)O | 10467 |
| 6 | IMPHY011627 | Arjunolic acid | OC[C@]1(C)[C@@H](O)[C@H](O)C[C@]2([C@H]1CC[C@@]1([C@@H]2CC=C2[C@@]1(C)CC[C@@]1([C@H]2CC(CC1)(C)C)C(=O)O)C)C | 73641 |
| 7 | IMPHY011797 | Oleic acid | CCCCCCCC/C=CCCCCCCCC(=O)O | 445639 |
| 8 | IMPHY012021 | Gallic acid | OC(=O)c1cc(O)c(c(c1)O)O | 370 |
| 9 | IMPHY014990 | Linoleic acid | CCCCC/C=CC/C=CCCCCCCCC(=O)O | 5280450 |
| 10 | IMPHY000601 | Triacontanoic acid | CCCCCCCCCCCCCCCCCCCCCCCCCCCCCC(=O)O | 10471 |
| 11 | IMPHY000687 | Syringic acid | COc1cc(cc(c1O)OC)C(=O)O | 10742 |
| 12 | IMPHY002419 | Ellagitannin | CC(=O)C[C@]1(O)C(=O)C=C2[C@@H]3[C@@]1(O)Oc1c3c(cc(c1O)O)C(=O)O[C@H]1[C@H]3[C@@H](OC2=O)[C@@H](O[C@@H]1COC(=O)c1c(-c2c(C(=O)O3)cc(O)c(c2O)O)c(O)c(c(c1)O)O)OC(=O)c1cc(O)c(c(c1)O)O | 101601927 |
| 13 | IMPHY003139 | Terchebin | O=C(c1cc(O)c(c(c1)O)O)OC[C@H]1O[C@@H](OC(=O)c2cc(O)c(c(c2)O)O)[C@H]2[C@H]([C@@H]1OC(=O)C1=CC(=O)C(C3([C@@H]1c1c(C(=O)O2)cc(c(c1O3)O)O)O)(O)O)OC(=O)c1cc(O)c(c(c1)O)O | 3084341 |
| 14 | IMPHY004631 | Stearic acid | CCCCCCCCCCCCCCCCCC(=O)O | 5281 |
| 15 | IMPHY004799 | Chebulagic acid | OC(=O)C[C@@H]1C(=O)O[C@@H]2[C@H]3COC(=O)c4cc(O)c(c(c4-c4c(C(=O)O[C@@H]2[C@@H](OC(=O)c2c5[C@H]1[C@H](O)C(=O)Oc5c(c(c2)O)O)[C@@H](O3)OC(=O)c1cc(O)c(c(c1)O)O)cc(O)c(c4O)O)O)O | 442674 |
[truncated: 95,485 more chars]
